# Genome-Wide Analysis Reveals the Role of Mediator Complex in the Soybean—*Phytophthora sojae* Interaction

**DOI:** 10.3390/ijms20184570

**Published:** 2019-09-15

**Authors:** Dong Xue, Na Guo, Xiao-Li Zhang, Jin-Ming Zhao, Yuan-Peng Bu, Dian-Liang Jiang, Xiao-Ting Wang, Hai-Tang Wang, Rong-Zhan Guan, Han Xing

**Affiliations:** National Center for Soybean Improvement, Key Laboratory of Biology and Genetics and Breeding for Soybean, Ministry of Agriculture, State Key Laboratory for Crop Genetics and Germplasm Enhancement, College of Agriculture, Nanjing Agricultural University, Nanjing 210095, China; xuedongjsrg@126.com (D.X.); xiaolizhang625@163.com (X.-L.Z.); jmz3000@njau.edu.cn (J.-M.Z.); 2015201057@njau.edu.cn (Y.-P.B.); 2016801204@njau.edu.cn (D.-L.J.); 2012201055@njau.edu.cn (X.-T.W.); 2017201074@njau.edu.cn (H.-T.W.)

**Keywords:** *Glycine max* (L.) Merr., *Phytophthora sojae*, mediator complex, *GmMED16-1*, plant defense

## Abstract

The mediator complex is an essential link between transcription factors and RNA polymerase II, and mainly functions in the transduction of diverse signals to genes involved in different pathways. Limited information is available on the role of soybean mediator subunits in growth and development, and their participation in defense response regulation. Here, we performed genome-wide identification of the 95 soybean mediator subunits, which were unevenly localized on the 20 chromosomes and only segmental duplication events were detected. We focused on *GmMED16-1*, which is highly expressed in the roots, for further functional analysis. Transcription of *GmMED16-1* was induced in response to *Phytophthora sojae* infection. *Agrobacterium rhizogenes* mediated soybean hairy root transformation was performed for the silencing of the *GmMED16-1* gene. Silencing of *GmMED16-1* led to an enhanced susceptibility phenotype and increased accumulation of *P. sojae* biomass in hairy roots of transformants. The transcript levels of *NPR1*, *PR1a*, and *PR5* in the salicylic acid defense pathway in roots of *GmMED16-1*-silenced transformants were lower than those of empty-vector transformants. The results provide evidence that *GmMED16-1* may participate in the soybean–*P. sojae* interaction via a salicylic acid-dependent process.

## 1. Introduction

The mediator complex, a component of the RNA polymerase II transcription system, is a crucial regulatory element of the transcriptional machinery and is likely to facilitate a variety of functional interactions. The earliest evidence for Mediator came from biochemical studies in yeast. It was first found as an activator which interferes with the activation of pol II transcription. [1]. The first mammal mediator-like complex purified was the translocon-associated protein complex from humans [2]. A set of approximately 30 consensuses were identified and the sequences of constituent subunits from 70 eukaryotes were analyzed using bioinformatic approaches [3,4,5]. Electron micrographs of the holoenzyme reveal that the mediator complex shows an extended conformation with head, middle, and tail modules [6]. In addition to the three core modules, a fourth separate regulatory module of the mediator complex, termed “cyclin-dependent kinase” (CDK), has been identified [7]. The head and middle modules interact with the promoters of RNA polymerase II and general transcription factors. The tail module binds to the enhancer of sequence-specific transcription factors, whereas the kinase module reversibly associates with the remainder of the mediator complex to regulate transcription [8,9,10].

Mediator complex subunits were first biochemically identified in fungi and metazoans, and in plants were first detected in *Arabidopsis* (*Arabidopsis thaliana*). The plant mediator complex is composed of 32 mediator (MED) subunits, one cyclin-dependent kinase 8 (CDK8), and one cyclin C (CYC) [5]. MED12, MED13, and CYC are located in the kinase module and mutation of these three subunits results in sensitivity or resistance to different pathogens in *Arabidopsis* [11]. Two tail subunits of the mediator complex, *MED14* and *MED15*, have been reported to be critical regulators of the salicylic acid (SA) response as well as the systemic acquired resistance (SAR) signaling pathway, which is similar to *NPR1* [12,13]. *MED18* is the direct target of four plant defense regulation genes, namely *TRX-h5*, *GRXS13*, *GRX480*, and *PTR3* [14]. MED19a is degraded via the proteasome-dependent pathway by interacting with HaRxL44 under pathogen signals [15]. MED21 interacts with the plant defense regulator HUB1 [16]. *MED25* and *MED 8* are involved in the jasmonate (JA)-dependent defense pathway [17,18,19]. Recent research has revealed that *MED25* and *MED30* may regulate flowering time in the model plant *Arabidopsis* [20,21].

Soybean (*Glycine max*) is a commercially important oilseed and vegetable crop grown worldwide. *Phytophthora sojae* is a soil-borne plant pathogen belonging to the class Oomycota that shows a restricted host range, including soybean as its primary host. It causes root and stem rot, and pre- and post-emergence damping-off, which leads to an annual yield loss as high as $200 million in the USA and $1–2 billion worldwide [22]. The pathogen can infect seeds, roots, stems, and leaves in all major soybean growing regions. To protect soybean against the disease, an environmental-friendly method is to breed disease-resistant cultivars. To date, numerous defense-associated genes have been reported to enhance resistance to *P. sojae* in soybean. Silencing of *GmSGT1* contributes to race-specific resistance in soybean [23]. *GmIFR* encodes a NAD(P)H-dependent oxidoreductase and overexpression of *GmIFR* in soybean enhances resistance to *P. sojae* by reducing the accumulation of reactive oxygen species [24]. Overexpression of a soybean ethylene-responsive factor (ERF) transcription factor, *GmERF5*, enhances resistance to *P. sojae* in soybean by interaction with a basic helix–loop–helix (bHLH) transcription factor (*GmbHLH*) and an eukaryotic translation initiation factor (*GmEIF*) [25]. An additional ERF transcription factor, *GmERF113*, is also reported to play a crucial role in the defense of soybean against *P. sojae* infection. Overexpression of *GmERF113* in a susceptible soybean cultivar resulted in increased resistance to *P. sojae* and positively regulated expression of the pathogenesis-related genes *PR1* and *PR10-1* [26]. A soybean dirigent gene, *GmDIR22*, contributes to the regulation of lignan biosynthesis and to resistance to *P. sojae* [27]. *GmPIB1*, a bHLH transcription factor in soybean, has been reported to enhance resistance to *P. sojae* by repressing expression of the promoter *GmSPOD1* [28]. The glycinol 2-dimethylallyl transferase *GmPT01* may be involved in conferring partial resistance to stem and root rot disease in soybean [29]. *GmDAD1*, a conserved defender against cell death 1 (DAD1) from soybean, is reported to play a critical role in defense against *Phytophthora* pathogens and might participate in the endoplasmic reticulum stress signaling pathway [30]. GmBTB/POZ, a novel BTB/POZ domain-containing nuclear protein, plays a positive role in *P. sojae* resistance and the defense response in soybean via a process that might be dependent on SA [31].

The former name for Mediator 16 (*MED16*), a tail subunit in the mediator complex, is Sensitive to Freezing 6 (*SFR6*). Increasing evidence indicates that MED16 plays a crucial role in regulating cold acclimation, drought and osmotic-stress tolerance, development, flowering time, and the circadian clock [32,33]. *SFR6/MED16* also contributes to the control of defense-related gene expression mediated by SA- and JA-responsive pathways [34]. Research on the *med16-1* (*ien1*) mutant, which is insensitive to exogenous NAD+, revealed the role of MED16 in SA-mediated SAR and JA/ethylene-induced defense pathways [35]. MED16 can also interact with WRKY33 and is required for WRKY33-activated transcription of PDF1.2 and ORA59, in which it acts as a central regulator of basal resistance against *Sclerotinia sclerotiorum* in Arabidopsis [36]. In addition, previous studies show that genetic interaction of the mediator complex subunits MED2, MED5, MED16, and MED23 is involved in the regulation of phenylpropanoid biosynthesis [37].

Except for the identification together with other 14 plant species, relatively little information is available on the organization, phylogenetic relationships, and structure of the mediator complex subunits in soybean, and no analysis of the function of a subunit in soybean growth and development has been conducted. Many mediator complex subunits, including *MED16*, have been reported to participate in the plant defense response in other species. In this study, we performed a genome-wide analysis of mediator complex subunits in soybean. The chromosomal distribution, gene duplication, phylogenetic relationships, and gene structures of the soybean mediator subunits were analyzed. Furthermore, we revealed the roles of *GmMED16-1* in soybean–*P. sojae* interaction. The hairy roots of *GmMED16-1*-silenced transformants showed enhanced susceptibility to *P. sojae* compared with that of non-silenced plants. Collectively, the results suggest that *GmMED16-1* positively regulates resistance to *P. sojae* infection in soybean.

## 2. Results

### 2.1. Genome-wide Identification of Soybean Mediator Complex Subunits

Using a bioinformatic approach, 95 mediator complex subunits were identified in soybean based on the domains of mediator proteins predicted with the Pfam database. The nomenclature of the soybean mediator subunits is listed in Table S1. Characteristics of the 95 mediator subunits, including peptide length, molecular weight, number of transmembrane helices, and isoelectric point (pI) were predicted using online database (Table S2). The subunits comprised 115 to 2266 amino acids and 11 of the 95 subunits contained 1–3 transmembrane helices. The predicted molecular weight of the subunits ranged from 13.11 to 251.23 kDa and the predicted pI values ranged between 4.56 and 10.05.

The subcellular localization of the mediator subunits was predicted and included extracellular, endoplasmic reticulum, plasma membrane, chloroplast, cytoplasm, and nucleus (Table S2). The predictions showed that the majority of the mediator subunits were localized in the nucleus (46/95, 48.4%), and few of the subunits were predicted to be localized in the extracellular space, endoplasm reticulum, and plasma membrane (1 (1.1%), 2 (2.1%), and 6 (6.3%), respectively). Thirteen (13.7%) subunits were predicted to be localized in chloroplasts and 27 (28.4%) in the cytoplasm.

### 2.2. Chromosomal Locations and Duplication Patterns of Soybean Mediator Complex Subunits

**Figure 1:**
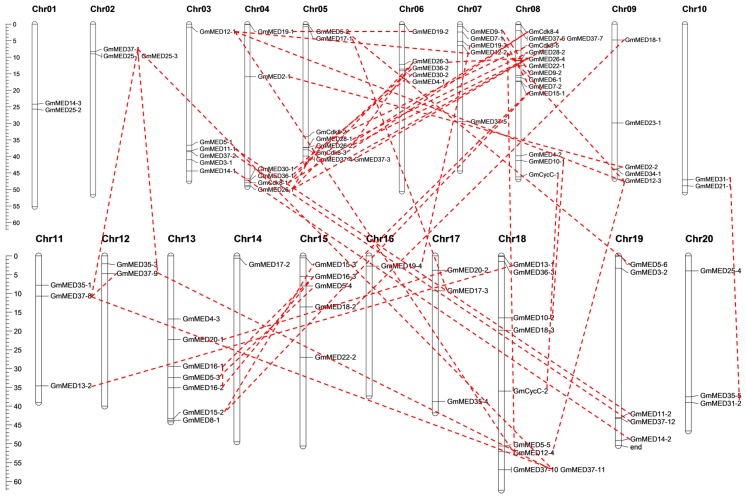
Chromosomal location and duplications of soybean mediator complex subunits. The chromosome number is indicated above each bar and the scale on the left is in MegaBases (Mb). The chromosome size is indicated by its relative length using the information from Phytozome and SoyBase. Each pair of segmental duplication is indicated by the dashed lines.

On the basis of their physical positions, the 95 subunits of the mediator complex were unevenly distributed on the 20 chromosomes of soybean (Figure 1, Table S1). The number of subunits located on the different chromosomes ranged from 1 to 14. Chromosome 8 carried the highest number of subunits, whereas chromosomes 14 and 18 each carried only one of the 95 subunits. Eleven of the 20 chromosomes carried at least five subunits and accounted for more than three-quarters of the total number of subunits. The remaining nine chromosomes carried no more than three subunits on each chromosome.

We investigated the existence of duplications within the soybean mediator complex. Both tandem duplications and segmental duplications were analyzed and only segmental duplications were detected for 65 of the 95 subunits (Table S3). Segmental duplications were detected on all of the soybean chromosomes except chromosomes 1 and 14 (Figure 1). No tandem duplication was identified among the mediator complex subunits.

### 2.3. Phylogenetic Analysis of Soybean Mediator Complex Subunits

**Figure 2:**
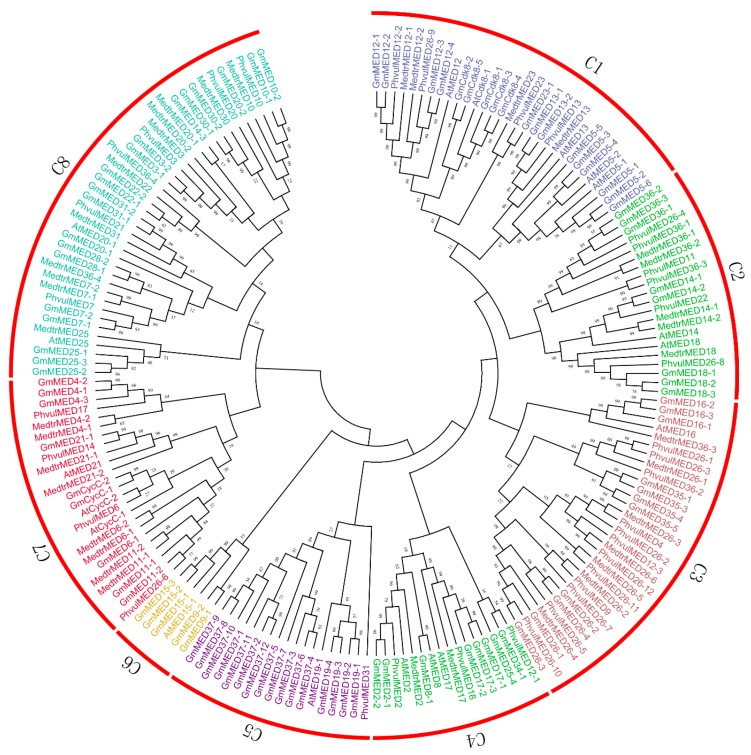
Phylogenetic tree for mediator complex subunits of soybean, *Arabidopsis*, *Phaseolus vulgaris*, and *Medicago truncatula*. The phylogenetic tree was constructed by MEGA 6.0 using the Neighbor-Joining method. The evolutionary distances were computed using the p-distance method. Bootstrap values in percentage (1000 replicates) are indicated on the nodes. Different subfamilies are highlighted using different colors and marked with red arcs outside of the cycle tree.

On the basis of conserved mediator domains reported previously [5], 36 conserved subunits were detected in the genome of *Phaseolus vulgaris* and 37 were identified in the *Medicago truncatula* genome. Among the mediator subunits identified in *Arabidopsis*, 18 are reported to participate in transcription regulation. Together with the 95 subunits from soybean, in total 186 mediator complex subunits from the four species were used to reconstruct a phylogenetic tree (Figure 2, Data sheets S1 and S2).

The mediator complex subunits from the former two plant species were used to analyze sequence conservation in the Leguminosae family, and the subunits selected from *Arabidopsis* were used to predict the putative function of the soybean mediator subunits. The mediator complex subunits were classified into eight groups, which were designated subfamily C1 to C8 and differed in the number of subunits per group (Figure 2). Almost all subfamilies contained subunits from *P. vulgaris* and *M. truncatula* as well as *Arabidopsis*, which thus indicated a high degree of conservation in the evolutionary relationship among leguminous plants, and permitted prediction of the putative function of the mediator complex subunits in soybean with greater confidence.

### 2.4. Gene Structure of Soybean Mediator Complex Subunits

**Figure 3:**
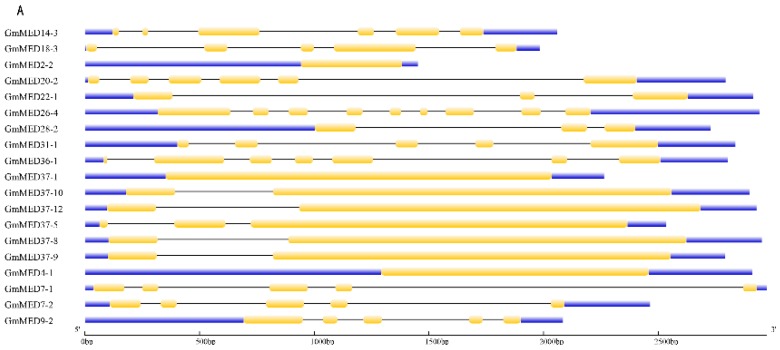

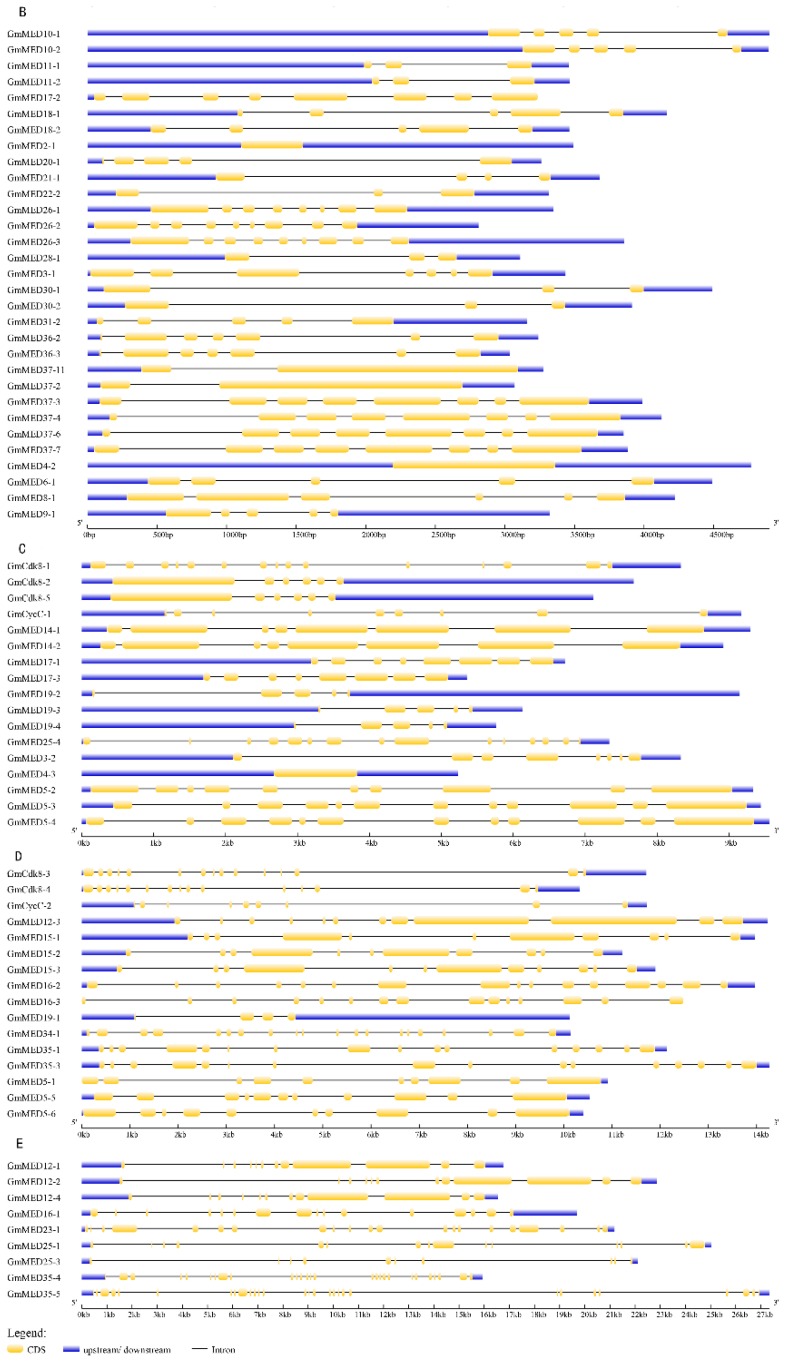
Gene structure of soybean mediator complex subunits. The structures of the 95 soybean mediator subunits were plotted using yellow boxes representing CDS (coding DNA sequence, exons), black lines representing introns and blue boxes indicating upstream/downstream sequences. The genes are listed by the length of the genomic sequence, from A to E represents sequence difference in size. **A**: <3 kb; **B**: 3 kb–5 kb; **C**: 5 kb–10 kb; **D**: 10 kb–15 kb; **E**: >15 kb The scale representing the length of each gene is on the bottom.

For an improved understanding of the characteristics of the soybean mediator complex subunits, the gene structure was analyzed by comparing the full-length genomic sequence with the corresponding coding sequence (Data sheet S1). Exons, introns, and upstream/downstream regions of the 95 mediator subunits were predicted using the GSDS 2.0 database [38]. The number of exons and introns per subunit ranged from 1 to 22, therefore the subunits were grouped by the length of the genomic sequence and the gene structure was analyzed (Figure 3). The gene structure of the soybean mediator subunits, including the length and the numbers of exons and introns, showed highest similarity within the same subunits with the difference in the suffix of -1, -2, -3 et al., and within a group the subunits differed in the length of the introns and the upstream/downstream regions.

### 2.5. Sequence Analysis and Expression Pattern of GmMED16-1 in Soybean

**Figure 4:**
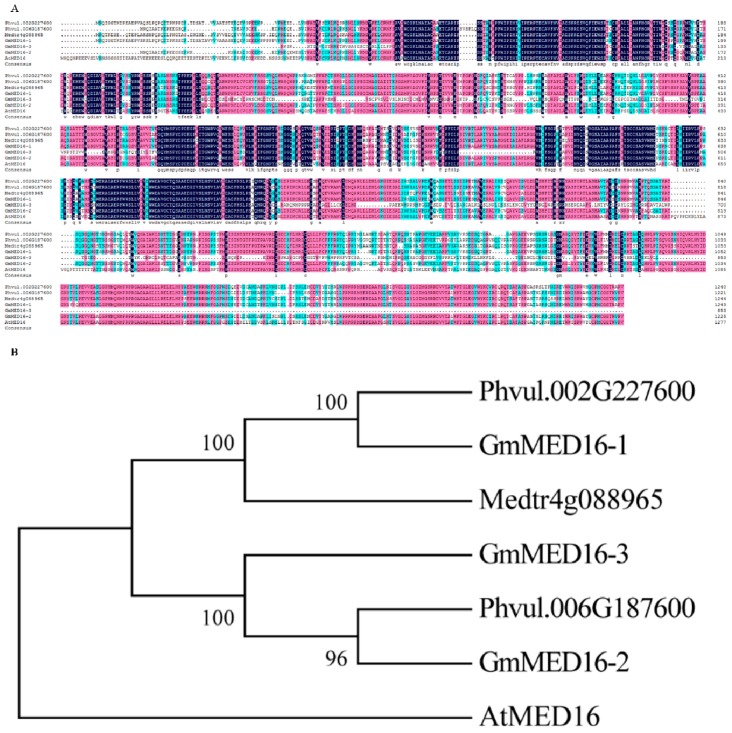
Sequence alignment and phylogenetic analysis of MED16 proteins from soybean, *Arabidopsis*, *Phaseolus vulgaris*, and *Medicago truncatula*. (**A**) Sequence aligment was performed using MED16 proteins from *Arabidopsis*, *Phaseolus vulgaris*, *Medicago truncatula* and *Glycine max*. Amino acid marked with boxes of different colors represents the similarity difference among these sequences. Dark blue represents 100%, pink represents >80%, cyan represents >60%. (**B**) Sequences used for phylogenetics analysis were same as these in alignment. The phylogenetic tree was constructed by MEGA 6.0 using the Neighbor-Joining method. The evolutionary distances were computed using the p-distance method. Bootstrap values in percentage (1000 replicates) are indicated on the nodes.

It was previously reported that *MED16* plays a crucial role in the defense response in *Arabidopsis* [35,36]. Three *MED16* homologs were detected in soybean, namely *GmMED16-1*, *GmMED16-2*, and *GmMED16-3*. On the basis of the sequence similarity of the MED16 protein, two MED16 proteins were identified in *P. vulgaris* and one in *M. truncatula*. Together with AtMED16, in total seven MED16 proteins were used for sequence alignment and phylogenetic analysis (Figure 4). Sequence alignment revealed that, with the exception of GmMED16-3, the remaining six proteins showed high sequence similarity (Figure 4A). Phylogenetic analysis revealed that the sequence of MED16 proteins was highly conserved among legume species (Figure 4B).

**Figure 5:**
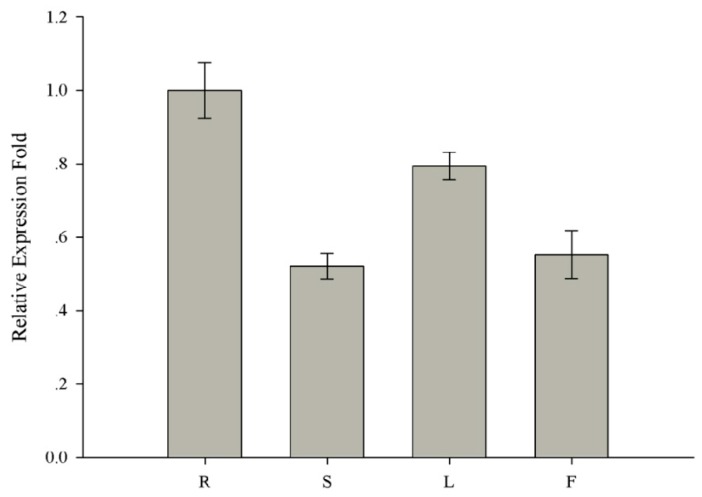
Expression profiles of *GmMED16-1* in various soybean tissues. Expression of *GmMED16-1* was detected in various soybean tissues under field grown conditions. Samples were collected in the flowering stages. The expression levels are normalized to *GmCons4* gene as an endogenous control. R: roots, S: stems, L: leaves, F: flowers.

To investigate the physiological role of soybean *MED16* genes, the expression pattern of *GmMED16-1* gene in the roots, stems, leaves, and flowers of soybean were analyzed by quantitative real-time PCR (qRT-PCR). The relative expression level of *GmMED16-1* was highest in the root (Figure 5). Given that Phytophthora root rot of soybean is a destructive soil-borne disease, *GmMED16-1* (*Glyma.13G181200*) was chosen for further expression analysis in response to *P. sojae* infection.

### 2.6. Expression Profile of GmMED16-1 under Phytophthora sojae Infection

**Figure 6:**
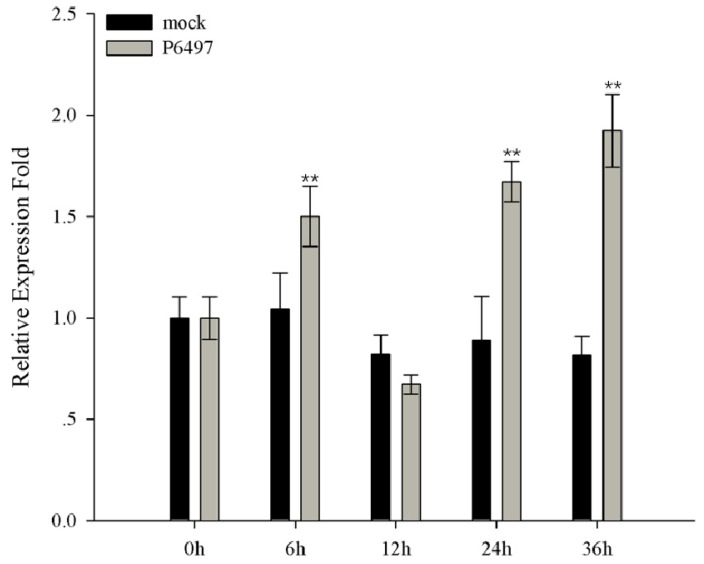
Expression profiles of *GmMED16-1* under *Phytophthora sojae* inoculation. Leaves from the seven days old seedlings were used for *P. sojae* infection. The mock control was treated with the V8 medium without *P. sojae*. Samples were collected from the inoculated area as well as the mock control at 0, 6, 12, 24, and 36 h post-infection (hpi). The expression levels are normalized to *GmCons4* gene as an endogenous control. ** indicate significant difference from control at *p* < 0.01, respectively.

To determine whether *GmMED16-1* is induced in response to *Phytophthora sojae* infection in soybean, the cotyledon of the seven-day-old seedlings was inoculated with *P. sojae* and the transcript level of *GmMED16-1* was analyzed by qRT-PCR. After inoculation, the transcript level of *GmMED16-1* initially increased at 6 h post-inoculation (hpi), then decreased at 12 hpi, and subsequently rebound at 24 hpi. The highest transcript level was recorded at 36 hpi (Figure 6).

### 2.7. GmMED16-1 Silencing Reduced Resistance to Phytophthora sojae in Hairy Roots of Transformants

**Figure 7:**
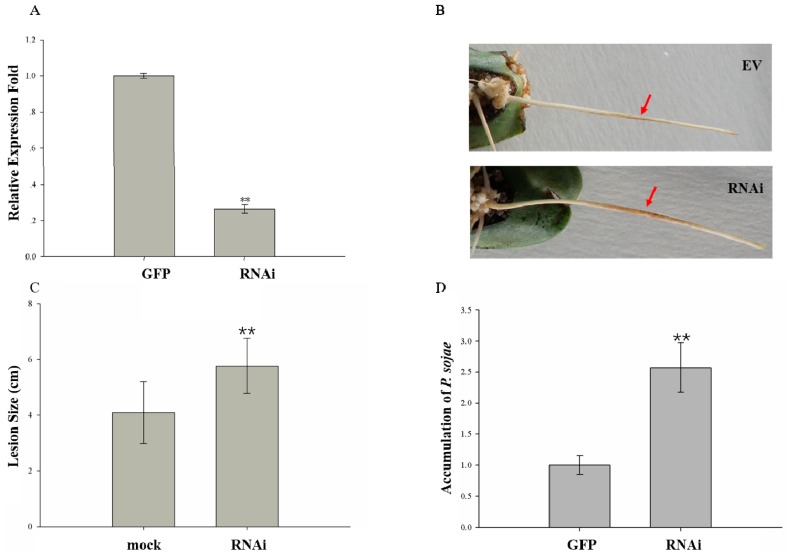
Silencing *GmMED16-1* makes the soybean hairy roots susceptible to *P. sojae*.(**A**) Expression pattern of *GmMED16-1* in the mock and RNAi hairy roots; (**B**) Phenotype of the mock and RNAi hairy roots inoculated by mycelial pellets of *P. sojae*, the red arrow indicates the water-soked lesions after *P. sojae* inoculation of the transgenic hairy roots. EV represents the mock control and RNAi represents the gene silencing ones. (**C**) Lesion size of the mock and RNAi hairy roots inoculated by mycelial pellets of *P. sojae*; (**D**) The biomass accumulation of *P. sojae* zoospores in the mock and RNAi hairy roots treated by the zoospores was quantified by qRT-PCR. *GmCons4* was used as the reference gene. The error bars indicate the standard deviation from three replicates. ** indicate significant difference from control at *p* < 0.01, respectively.

Given that *GmMED16-1* expression was highest in the root (Figure 5), we investigated whether *GmMED16-1* was involved in soybean–*P. sojae* interaction using *Agrobacterium rhizogenes* mediated hairy root transformation using RNA interference (RNAi) in soybean cotyledons. A 365 bp conserved DNA fragment was selected to specifically silence *GmMED16-1*. The pHellsGate12:GFP:*GmMED16-1* RNAi vector was constructed using the Gateway technology and the plasmid was transformed into *Agrobacterium rhizogenes* strain K599 by electroporation. Hairy roots that exhibited GFP fluorescence were first used to detect the RNAi efficiency. The results of qRT-PCR analysis indicated that the RNAi efficiency attained 70%, thus the expression level of the transgene in the RNAi hairy root was only 30% that of the mock roots (Figure 7A).

The RNAi-positive hairy roots were used for a *P. sojae* infection assay. The *GmMED16-1* RNAi roots resulted in long, water-soaked lesions following inoculation with *P. sojae*, whereas hairy roots transformed with the control vector showed dark brown spots at the infection site (Figure 7B). Furthermore, the lesion size on the RNAi hairy roots was significant longer compared with that of hairy roots transformed with the control vector (Figure 7C). We also detected the accumulated biomass of *P. sojae* in the RNAi hairy roots and the hairy roots expressing the control vector. At 48 hpi, the accumulated biomass of *P. sojae* was significantly higher in RNAi hairy roots compared with the hairy roots expressing the control vector (Figure 7D). These results suggested that *GmMED16-1* plays a positive role in soybean–*P. sojae* interaction.

### 2.8. Expression Analysis of Plant Defense-related Genes in Response to Phytophthora sojae Inoculation

**Figure 8:**
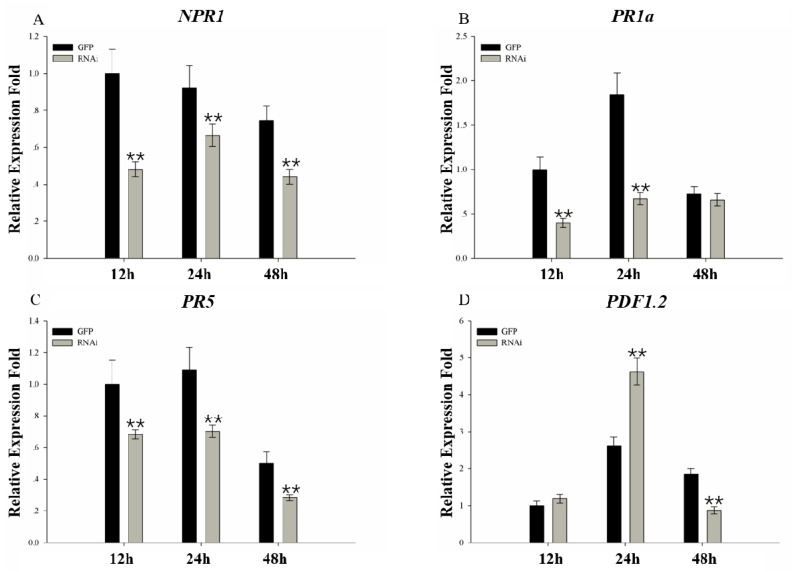
Expression profiles of *NPR1*, *PR1a*, *PR5* and *PDF1.2* in the transgenic hairy roots. Expression of marker genes *NPR1*, *PR1a* and *PR5* in the *SA* signaling pathway and *PDF1.2* in the JA pathway was detected in the RNAi hairy roots as well as the mock ones. The hairy roots were inoculated with zoospore suspension (about 10^4^ zoospores/mL) of *P. sojae* P6497. Samples were collected at 12, 24 and 48 hpi. The expression levels are normalized to *GmCons4* gene as an endogenous control. ** indicate significant difference from control at *p* < 0.01, respectively.

Plant hormones have been reported to play vital roles in the plant defense system. To further examine the function of *GmMED16-1* in soybean in response to *Phytophthora sojae* inoculation, expression level of genes involved in the SA and JA signaling pathways was analyzed. Expression of genes involved in the SA signaling pathway (*NPR1*, *PR1a*, and *PR5*) showed a lower level in the RNAi hairy roots than the mock (Figure 8A–C). Expression of *PDF1.2*, which participates in the JA signaling pathway, was higher in the RNAi hairy roots than the mock at 24 hpi, whereas at 48 hpi the expression level had decreased (Figure 8D). These results suggested that *GmMED16-1* may participate in the soybean–*P. sojae* interaction by means of the SA signaling pathway.

## 3. Discussion

In the present research, 95 mediator complex subunits were identified in soybean. The mediator complex subunits of soybean, together with those of *Arabidopsis*, *P. vulgaris*, and *M. truncatula*, were classified into eight subfamilies, which displayed substantial differences in gene structure. Among the soybean subunits, *GmMED16-1* was chosen for further functional analysis. *GmMED16-1* was induced in response to *P. sojae* infection (Figure 6). We also confirmed that silencing of *GmMED16-1* using RNAi technology led to enhanced susceptibility of the transgenic hairy root to *P. sojae* infection. Expression analysis of genes involved in the SA and JA signaling pathways suggested that *GmMED16-1* may participate in the soybean—*P. sojae* interaction via a SA-dependent process. 

Mathur et al. identified mediator complex genes in 16 plant species and analyzed the structure, phylogenetic relationships, and expression profiles of representative genes in the dicotyledon model plant *Arabidopsis* and the monocotyledon model plant rice during reproduction and under abiotic stress [5]. In the present study, we identified 95 mediator complex subunits based on the current assembly of the soybean genome. Some in silico parameter, like peptide length, molecular weight, numbers of transmembrane helices, isoelectric point and the subcellular localization were predicted using the online database. These results will provide a foundation for the further functional analysis of the soybean mediator subunits. Besides, chromosomal location as well as the duplication patterns were analysis and results showed that the 95 subunits unevenly located on the 20 chromosomes of soybean and only segmental duplications were found. Gene duplications are considered to be one of the primary driving forces in the evolution of genomes and genetic systems [39]. Segmental duplications, one of the main causes of gene family expansion in plant, multiple genes through polyploidy followed by chromosome rearrangements [40]. It occurs most frequently in plants because most plants are diploidized polyploids and retain numerous duplicated chromosomal blocks within their genomes [41]. Researches showed that the soybean expansin gene superfamily has expanded through the duplication events [42].

We analyzed the chromosomal location, duplication, phylogenetic relationships, and gene structure of the mediator complex subunits in soybean in comparison with subunits from *P. vulgaris*, *M. truncatula*, and *Arabidopsis*. The 186 mediator complex subunits analyzed were classified into eight subfamilies. AtMED12 and AtCDK8, which were classified in subfamily C1, are CDK module subunits involved in plant defense pathways and phosphorylation of the CDK module, which is important in defense gene regulation [11]. MED14 belonged to subfamily C2 and has been reported to participate in plant defense via the SA-induced pathogen defense response and SAR signaling pathway [13]. MED18, which was also classified in subfamily C2, is involved in plant sensitivity to necrotrophic fungal pathogens [14]. AtMED16 was classified in subfamily C3 and affects the expression level of defense-related genes in the SA and JA pathways as well as SA-mediated SAR [35]. Recently, it was observed that MED16 regulates resistance to *S. sclerotiorum* by governing both JA/ethylene-mediated and *WRKY33*-activated defense signaling [36]. In addition, MED16 is involved in defense signaling crosstalk together with MED14 and MED15 in *Arabidopsis* [43]. MED19a, a member of subfamily C4, is a positive regulator of plant defense against the oomycete downy mildew pathogen *Hyaloperonospora arabidopsidis* (*Hpa*) and participates in the JA/ethylene signaling pathway as well as SA-triggered immunity in *Arabidopsis* [15]. MED15, which is a critical regulator of SA response in plant defense, also influences the flowering-time phenotype in *Arabidopsis* [12]. The AtCycC-1 and AtCycC-2 genes classified in subfamily C7 are involved in plant defense pathways and phosphorylation of the CYC module, which is important in defense gene regulation [11].

*Phytophthora sojae* is a soil-borne oomycete pathogen and it mainly causes Phytophthora root and stem rot under field conditions. As an essential component of the transcriptional mechanism, the mediator complex plays an important role in plant–pathogen interaction in *Arabidopsis*. Five individual subunits of the mediator complex have been functionally characterized to participate in plant pathogen resistance, namely *MED25/PFT1*, *MED21*, *MED15/NRB4*, *MED16/SFR6*, and *MED8* [32]. Among these genes, *MED16* is a key component of basal resistance against the necrotrophic fungal pathogen *S. sclerotiorum* [36]. In addition, *MED19* is a positive regulator of plant resistance against the oomycete pathogen *Hpa*, leading to proteasome-dependent degradation of MED19a [15]. In the present research, *GmMED16-1* expression was induced in response to *P. sojae* infection. In addition, the lesion size and biomass accumulation of *P. sojae* in RNAi hairy roots showed a significant difference compared with hairy roots transformed with the control vector. These results indicate that *GmMED16-1* is a positive regulator of the soybean—*P. sojae* interaction.

Plant pathogens are often categorized as biotrophs and necrotrophs on the basis of their lifestyles. Biotrophs are pathogens that derive nutrients from living host tissues, whereas necrotrophs are pathogens that derive nutrients from dead or dying cells [44]. Phytohormones, including SA, ethylene, JA, abscisic acid, auxin, brassinosteroids, gibberellic acid, cytokinin, and the recently identified strigolactones, orchestrate effective defense responses by activation of defense gene expression [45]. Among these phytohormones, SA and JA are crucial in the regulation of signaling networks for basal resistance against multiple pathogens [46,47]. The SA signaling pathway positively induces plant defense against biotrophic pathogens that feed and reproduce on living host cells, whereas JA signaling pathways are required for resistance predominantly against necrotrophic pathogens and herbivorous insects, which derive nutrients from living host cells, commonly through specialized feeding structures [44,48,49]. *Phytophthora sojae* is described as a hemibiotrophic pathogen and secretes effector proteins (coded by *Avr* genes) to manipulate and invade living host cells during the initial biotrophic stage of infection [50]. In Arabidopsis, SFR6/MED16 controls SA- and JA-mediated defense gene expression and is essential for resistance to the hemibiotrophic pathogen *Pseudomonas syringae* [34]. The present results showed that the expression level of three genes involved in the SA signaling pathway (*NPR1*, *PR1a*, and *PR5*) was lower in RNAi hairy roots than the mock roots (Figure 8). The expression pattern of plant defense-related genes in the RNAi roots was opposite to that observed in the mock roots in response to *P. sojae* infection. These results suggested that *GmMED16-1* may participate in the soybean–*P. sojae* interaction via the SA signaling pathway. Similar findings have been observed in *Arabidopsis*. MED19a, a positive regulator of resistance to the oomycete *Hpa*, interacts with HaRxL44, which alters the balance of defense transcription from SA-responsive defense to JA/ethylene-signalling, and enhances susceptibility to biotrophs by attenuating SA-dependent gene expression [15].

## 4. Materials and Methods 

### 4.1. Identification of Mediator Subunits in Soybean

Conserved pfam domains have already been predicted [5] and the seed files of these domains were downloaded from the Pfam database (http://pfam.xfam.org/) [51]. They were used to search the soybean genome as well as the *P. vulgaris* and *M. truncatula* by the software hummer 3.0. A total of 95 subunits of the soybean mediator were finally identified from the newest soybean genome database. Besides, 37 conserved subunits were found in *M. truncatula* and 36 *P. vulgaris* were found. Data files containing the information of the final 95 soybean mediator subunits (including their corresponding physical locations on soybean chromosomes, genomic sequences, coding sequences, and protein sequences) were downloaded from *Phytozome* v11.0 (http://phytozome.jgi.doe.gov/pz/portal.html) [52] (Table S1, Data sheet S1). Theoretical isoelectric point (pI) and molecular weight (MW) of soybean mediator subunits were computed by ExPASy “Compute pI/Mw” tool (http://www.expasy.ch/tools/pi_tool.html) [53,54,55]. The subcellular localizations of the mediator subunits were predicted using WoLF PSORT (http://www.genscript.com/wolf-psort.html) [56]. The numbers of transmembrane helices in these proteins were predicted by TMHMM Server v.2.0 (http://www.cbs.dtu.dk/services/TMHMM/) [57].

### 4.2. Phylogenetic and Structural Analysis of Mediator Subunits in Soybean

The protein sequence of 95 soybean mediator subunits (Data sheet S1), 37 conserved subunits in *M. truncatula* and 36 conserved *P. vulgaris* subunits as well as 18 subunits from *Arabidopsis* (Data sheet S2) together were used for multiple sequence alignments by Clustal W. Then the evolutionary analyses were conducted with MEGA 6.0 [58]. The unrooted phylogenetic tree was then constructed using the neighbor-joining method [59]. Phylogeny test using the bootstrap method and the number of bootstrap replications was 1000. The evolutionary distances were computed using the p-distance method [60]. Gene structure analysis was performed in the Gene Structure Display Server (GSDS) database 2.0 (http://gsds.cbi.pku.edu.cn/) [38] with genomic sequences and coding sequences (Data sheet S1) with default settings.

### 4.3. Chromosomal Locations and Gene Duplication Analysis

The chromosomal locations of soybean mediator subunits were illustrated by MapChart [61] based on the physical locations downloaded from *Phytozome* v11.0 [52] (Table S1). Segmental and tandem duplication events of these soybean mediator subunits were identified using the Multiple Collinearity Scan toolkit (MCScan) [62] from the Plant Genome Duplication Database (http://chibba.agtec.uga.edu/duplication/) [63] with relevant parameters: BLASTP was used to search for potential anchors (*E* <1 × 10^−5^, top five matches) between every possible homologous pair, and these pairs were used as the input for MCscan. Syntonic blocks were identified using the *E*-value ≤ 1 × 10^−10^ as a significance cutoff. Tandem duplication was defined as homologous genes with less than ten gene loci in between and > 50% similarity at protein level on a single chromosome [64].

### 4.4. Plant Materials and Phytophthora sojae Inoculation

Soybean (*Glycine max* (L.) Merr.) cultivar Williams 82 was used for the experiment. Soybean seedings were grown in vermiculite substrate in the greenhouse at 25 °C under 16-h-light/8-h-dark cycle for seven days. Then, the leaves were used for *P. sojae* (P6497) infection. The inoculated leaves were placed in the petri dishes, on the surface of the wet filter paper to retain moisture. The petri dishes were cultured in the incubator during the infection of P6497. The mock control was also cultured with the V8 medium without *P. sojae* and samples were collected from the inoculated area as well as the mock control at 0, 6, 12, 24, and 36 h post-infection (hpi). All samples collected above were rapidly frozen in liquid nitrogen and then stored at −70 °C for RNA extraction.

### 4.5. RNA Isolation and Quantitative Real-time PCR

Total RNA of the samples was extracted using the RNAsimple Total RNA Kit (Tiangen, Beijing, China), gDNA elimination and reverse transcription of the first strand cDNA were performed with the PrimeScript^TM^ RT reagent Kit with gDNA Eraser (TaKaRa, Dalian, China). Quantitative real-time PCR was carried out with the LightCycler^®^ 480 II real-time PCR system (Roche, Basel, Switzerland) using the SYBR^®^ Premix Ex Taq^TM^ II (TaKaRa) according to the manufacturers instructions. The relative expression levels of target genes were calculated using the 2^–ΔΔC*T*^ method [65]. The *GmCons4* (GenBank: BU578186.1) gene was selected as the reference for the qRT-PCR. All the nucleic acid sequences of the primers are listed in Table S4.

### 4.6. Construction of pHellsGate12: GFP: GmMED16-1 RNAi Vector

A 365bp conserved fragment of the *GmMED16-1* gene was first cloned into the Gateway entry vector pDONR221, then constructed into pHellsGate12: GFP through an LR recombination reaction between the entry clone and the destination vector (Invitrogen, Shanghai, China). The vector pHellsGate12: GFP which was modified from pHellsGate12 were used for RNAi construction [23,66]. The constructed pHellsGate12: GFP: *GmMED16-1* was validated by PCR as well as sequencing.

### 4.7. Agrobacterium Culture, Soybean Hairy Root Transformation

The plasmid vectors pHellsGate12: GFP: *GmMED16-1* and the empty vector were transformed into *A. rhizogenes* K599 strain by electroporation. The positive clones were selected on the LB medium (with 50 μg/mL kanamycin and 50 μg/mL streptomycin) after 48 h at 28 °C and the individual clones were validated by colony PCR. The single positive colony was shaken in 4 mL at 28 °C, and the bacteria cultures were centrifuged at 4000 rpm for 5 min and resuspended with 10 mM MgCl_2_. The final OD_600_ of bacteria was adjusted to approximately 0.5 to make it suitable for the hair root transformation on the soybean cotyledon.

The soybean hairy root transformation was performed as the described procedures with some modification [23,67]. Soybean cotyledons were removed from the 5-days-old seedlings gently and a circular cut (approximately 0.4 cm in diameter) was made near the petiole end. The cut cotyledons were then place in the Petri dishes containing 0.6% agar medium immediately. Approximately 20 μL of the *A. rhizogenes* suspension were pipetted into the cut surface. These cotyledons were placed in incubator at 25 °C in a dark condition. Seven days later, the callus was emerged at the cut site in the cptyledon. After 3 weeks, transformed hairy roots were seen along a callus ridge on the inoculated cotyledons, and the positive transformed hairy roots were then used for *P. sojae* inoculation and the expression pattern of some genes involved plant hormone signaling pathways.

### 4.8. Assay of P. sojae Infection of the Soybean Hairy Roots

The positive roots were verified by GFP fluorescence and qRT-PCR. Then the positive ones with similar length were for the inoculation with zoospore suspension (about 10^4^ zoospores/mL) of *P. sojae* P6497. Lesion length was measured by venire caliper. The biomass of *P. sojae* was determined by q-PCR using primers from *P. sojae* and soybean. Student’s t test was performed to determine the significance of differences between control and the RNAi roots. For gene involved plant hormone signaling pathways in the transgenic hair roots, the infected roots were mixed and collected at 12, 24 and 48 hpi.

## Supplementary Materials

The following are available online at https://www.mdpi.com/1422-0067/20/18/4570/s1.
