# Supplementary material for: Genome-Wide Analysis Reveals the Role of Mediator Complex in the Soybean—Phytophthora sojae Interaction
Source: Int J Mol Sci. 2019 Sep 15;20(18):4570. doi: 10.3390/ijms20184570 (PMC6770253; doi:10.3390/ijms20184570)
Supplement: Supplementary file 1 [file ijms-20-04570-s001.zip › Supplementary Materials/Data sheet S1.docx]

**Data sheet S1： The genomic sequences, coding sequences and protein sequences of the 95 soybean mediator subunits**

**Note:** The genomic sequences are listed on pages 1-310, the coding sequences are listed on pages 311-388, and the protein sequences are listed on pages 389-415.

>GmMED2-1

AGAACAGGGACAAACTAGTAAGGGGGTTTGTTTCACTTCAGATTTGGTTAATTAGGGTTCCGTTGGCGCGAGCTAAGAACGAAGGAAGTCGAAGCAGTAAAACCCAATCAAAGACCCTCACTCTCAGCCCACTCTTCACGTAAGTGAAACAGTAAGTGTCACCAACACATATGTGCTCTGCATCTTGAATTGCTAATGCTATTTTTCTTAGAGGGTGGATAAGGATAATTTGTATGCCACTGTGAATTGGGATTCATTTCCCTGTGTATTGTCTCAAACCAAGGAAAAAGTTAAAATTAAAATTTAATTTTATTGAGTTTTGATTTTGTTCTTACATGGTTGGGTCAGATAAACTAAACTAATTTGTGCTGGTCTATTGGTTTGTCTCCCTGTGACCTTCCAAGTCTAATTTGCACCTGGTTATCAGAATAATAGGCCAATGAGATTGAGTGAGTTAGTGGTGCCGATCAATCTGGTGGTAACAGTGATATCAATCTGAATGCCTTTTTTTAGTACAAATCTAGTTCAATGTCTATGCCATAAGAATGTACCTCAATAAATTAAAGTTTTAAGGTTATTGTGGAATATGTTCACTATTTCTCATGCAATTCTTGCTTGCTTATTGCTATAAGTTGCGTGCTATCATTCTTCATTGAGTACAATGACAGTTTTGAATTTATAATACCTATGTTTGGTGGATTTTAATTTTTATTAGATTAGATTCATAACCAGCAACTATTTTACTTTATAATATTTATGTTCATGATCAGAAAAACTTTTCTACTTTATAATATTTATATTCCCTATCTGAGATCAAAGTTAATTGCTGGATCTGTGTATCTGTTTATAGGGTGCTTGGTATTAGCAGTGTTTCTCCTTCCACCTTCCCTTTTTATAATGCTCCAATTCATTTTGTGTTCTGTTTCATAGGTTTAGTCTTGCATATTAATTTGTCATGCATTTTCAGGAAACAAGATCAGGCTAGGAGACAAATAGCTTGAAATCTTTTATTGCTAGCTACTGTTCTTGTGCTTTAATTCAATTGGTCTTACTTTCCCTTAGGATCTGTAATTACAAGAAGCTTAGATTTGACACAAGCAGGCACAATGGACAGCATAGTTGATTCTCTGAATAATGCCTATCAAGATTTTGTTGCTGCAGCAGCTAATGTGTTAGAAGCCAAAGAAAATGCTGGTTCCATTAAAACAATGGCAACAGATACTGCTCTAGAAAACTTTAAGCAGAAGTGGGAATTGTTTAGAGTAGCATGTGATCAAGCTGAGGAGTTTGTGGAGTCTGTGAAGCAAAGGATAGGATCTGAGTGTCTGGTGGATGAGGCAACAAGGCCTGTGGCAGGAAAACCTGGACAAGCTACCATGACCGGTCTTCCTCCCATTAGTGCAGTTCGGTTGGAACAGATGAGTAAAGCTGTTCGATGGCTTGTGATTGAATTGCAGCATGGCTCTGGAGCTGGTTCTGCTAATTCAGCTCTTACCCACCCCTCAGCTCCGTTTGATGCCAGGTTTTCTGAAGATGCTGCTCAGTAGCATTAATGTGACTGTGATGATTAATGTTATATATTTTTTTCTCTATGATGATCATGCTTAGACTATATTGTGTTAAACATTTTATACAAGATTAAGGATTAGATTTATGCTAAGCTGTTAAAGAATGTAACTTGTGTGTATTGTGCATGACACAACAATCCTTATTCCTTAATTTTCTGTTGGTGATAATTAATGTATTTTATCCTGCAAAAATGAAAACGCATGAGTTATGCCTGAATTTCCATTATGCATTTATTAACGCTGTAAAATGTCTTCATACAAGTCATGCAATTCCCAAACTGCATTGACATGAAGGCCTTCTTCAAACTATATGTTATGATGCAAAGAAATTGATAACAGGCGAGGTCTACAACAGTTTGTTTAATTAATTACACTCCTAAAACACTGTGAATCACACACACATTCACTTAACTGAATACGAAATAGTTTACTATTGCTCACATGGCGGCCTCTGGAAGGAATGGAGCCCACAGAAGATACTTTAGAAGCTTGGAAGAAAGATCAGAAGTTTCAAGGAAAACAAAGAAATGAGATTTTGATTTGCTTCTTGAAGCTTCATCCTGTGCATCCTACTTGTCGTTTTGGTAAGATCTAGCTATGATATTCAATTAACTTAAATCACATCTTTGTTGACTTGAAACTTTCTTTTTATCTCCATAATGTAACAGTCCTACAGGTCTTGCAATTTAATTAGTTTTTTTTCAACTGCTGCTTGAAAAGTTTTGCCTTTTGGTCATCTTTTTTTCAAGCAAATTAACTTTGTTTGAAATGATTTTGGCTCCTTAGTTTTCATCATCCTGTTGATATGAGAATGGTTGCTTTTATGATAAATTTGTCCATGACCCTCCCTCTTGATTTTTCAACTTTTTCCGTCTTCCTCGTGGATGTTGTAGATTCTACAGGATAGTGGAGCTATAAAGATAGCTGCAACATCTGGGAGGGATATTCAACTGGAGGAAGTATCCTGTGATTAACAATATTAAGCAGCTTGAGAGCGCTGATCTAGAAAGGAATAAGAAGCATGCTCACAAAGGTGGTGGCGATGATGATGAAGATCACGACGATGAGGATGATGACAGTGATGACGGCAACAATAAGATGAAGAAAGGAAAGTGGGATGCCAATGAAAATGAGGAGGAGTATAAAGATGGACACAAAGAATGATTTCAGAGGATGTGCATGCTTACAAGCCATCCCATGTGGATCACAATCACACGAACCTTGAATTGAATGTGTTTTTTCACCCCAAATGATTTAGAAGTTGGGAAAATAATGCCCATCCATTTTTCCAAGAAGAATTCTTCAACATCTCCAAAATTTCTGACTAGAAATTCTTCAAAAGCTACCATTCCTTCTTAAATTCTTCTCTATCTCAAAACACATTCCCCAAGCGAAGGCCATGAATTATACACACAAACAATGTGAATTTGAATCCATGGAAGGGGAGACCAAGTTCCGTGCTACTTCCTTTGTTTGATTTTGCATGTTATCTGTTTCGGTCCAATGCGCAATTCAAAGTTTTGACCATCATCCATCTAACAAACTCCACTGGCCCTTTTATAGAACTATACAATCTCAGAAGGGAAAGTAATTTCAGTTTTAAACATTATAGGATGTCACCACCCTTACACAGTCTTTTACTGCCACAACCAGCAAAGTGGCACCAATCTGTATGAGGGGTTAGGTGAAAATTGCCACAAAGACACCTCTAAGTGGGACTCCCGTTTGTCATTTTCCGCCTCCTGATGTCTGGGTGTCTTTGCCTGTGGCTACATAACTAGATGCTTCAATTTTTAACAAATTAGAGGCTGGAGCTCTGTTTCTTATAGAACTCTTAATATATTTTGGGGTGCTGCTAAGTGCACCCAGCATTATTGCTGATGCACCCAGCAATTTAAGTGAAGTGGCAAAAATACCCTTTAC

>GmMED2-2

AGAAAGGGGGTTTCTTTCATTTCAGATTTGGTTAATTAGGGTTCCGTTGGCGCGAGCTAAGAACGAACGAAGTAGAAGCAGCAAAACCCAATCAAAGACCCTCACTCTCACCCCACACTTCACGTAAGTGAAAACGGTAGGTGTCACCCAACAGATATGTGCTCAGCATCTTGATTTGCTAATGCTATTTTTCTTAGAGGGAGGATAAGGATACAAATTGGGATTCATTTCCCTGTGTGTTGTAGGAAAAATTAAAAAATTAATTTTATTGAATTTTGATTTTGTTCTCATATGTGGCTAGATCAGTTAAACTAAACTAATTTGTGCTGGTCTATTAGTTTGTTTCCCTATGGCCTTCTATGTCTAATTTGCACCTGGCAATCAGAATAATTGGCCATTGAGATTGAGTGAGTAAGTGGTGCCGATCAACTTGGTGGTAACAGTGATATCAATCTGGATGCCTTTTTTTACTAGAAATGTAGTTCAATGTCTATGCCATAAGAATGCACCTCCATCGATTAAAATTTTATGGTTATTGTGGAATATCTTCACTATTTCTTGTGTGATTCTTACTTGCTTATTGTAAGGTGCATGTTATCATTCTTGATTTGAGTACAATGACAGTTTTGAATTTACCAGTGTTTGGTTCTGGATCTGCGTATCTGTTAATAGGGTGCTGGGTATTATCAGTGTTTCTCGTTCCACCTCCCCATTTCGATAATGCTCCAGTTCGTTTTGTGTTGTGCTTCATAGTTTTTGTCTTACATGTTAATATGCCATGTCTTTTCAGTAAAACAAGATCATCAGACTAGGAGACAAATAGCCTCAAATCTTTTTTTGCTAGCTACTGTTCTTGTGCTTTAATTCCAGAGGTCTTACTTTCCTTTAGGATCTGTAATTACTAAAAGCTTAGAATTTCTTTGTATGACACAATCAGGCACCATGGACAGCATAGTTGATTCTCTGAATAATGCCTATCAAGATTTTGTTGTTGCAGCAGCAAATGTGTTGGAAGCCAAAGAAAATGCTGGTTCCATTAAAACAACAGCAACCGATACTGCTCTAGAAAACTTTAAGCAGAAGTGGGAATTGTTTAGAGTAGCATGTGATCAAGCTGAGGAGTTTGTGGAGTCTGTGAAGCAAAGGATAGGATCCGAGTGTCTGGTGGATGAGGCAACAAGGCCTGTAGCAGGAAAACCTGGACAAGCTACCATGACTGGTCTTCCTCCCATCAGTGCAGTTCGGTTGGAACAGATGAGTAAAGCAGTTCGATGGCTTGTGATTGAATTGCAGCATGGCTCTGGAGCTGGTTCTGCTAATTCAGCTCTTACCCACCCCTCAGCTCAGTTTGATGCCAGGTTTTCTGAAGATGCTGCTCAGTAGCATTAACGTGACTGTGATGATTAATGTTATATATTTTTTCTCTATGTTGATCGTGCTTAGACTATATTG

>GmMED3-1

GTGGGCGTGCCACGGGCAAATGCAGATGCAGATGCAACAAACGCAGCAGCAGCTGCAGGCCACGGCGGCACCATTCTCGACCTCCACTCCTCCTCCTCCTCCTCCTTCCTCCGGCGGCTCCGCGTCCGAGGCACCGCCGAAGCAGGTGGCTCAAGCAATGGACAAGCTCGGACAAGCCGAACGAATCATCGCCGACATCCGAATCGGCGCCGACCGCCTCCTCGAAGCGCTCTTCGTCGCGGCGGCGCAGCCTCACCAAGGCAACAAGCCTCTCCAAGTCTTCCTCAAAGAAGACGCATGCATGCGTCAGTATCTTCAAGACCTTCGGTCGCTTGGTAAGCGTGCCTTTTCACTCGCTCCTTTTGTTTTTTAGGTTCCGCGGATTAGGGTTTTGGATTGGACTCTGCGACGTCGTTTGTGTGATTAGTTCTGCGTGGTTTTGGTTTAATAGGTAAGGAGCTAGAAGAGTCTGGAGTTCTCAGCGAATCAGTTCGATCGAGGAAAGACTTTTGGGGCTTGCATATGCCACTGGTTTGTCCAGATGGCGCCGTGGTTGCGTATGCGTGGAAACGACAACTTGCAGGACAAGCGGGTGCTTCTGCCGTTGACCGGACCAGGTTCGATTTGTTCAATTTGGTTCTGTTTCAGTTCTCTCGTTCAAACTTCTGTGTTAGTACATTTAGATAACGGAATAGGATGGAAAACAATGGATTCCAATTCAACTGTCTTTTGTTTTGCTTGGAGTTGATAATTGCTATGGTGTTATTAGCGATTTATTTATTTAGTTATTTTTATGATTTTTGTGCATTCTTAGGTGGATATCTTAATCTTAAGTTTTGGTATTACCTCCTGAATTCGTAGCACATGGTATTAATAATATGAATCTTAGCCAAGTTATGCTGCGATATAGCTGTCTCGTACCTAAGTTGTGTAGAAATAGAGAACTAGTACTACGCCAGAAAACAGTTTTTTTATTGGGTATTGTGGTTTACAATGATCGTGCTGACTTTCGAATCTCAAGTCTGCTAAATTGTGGTACTTTAGTTGCTCACTTAGGCTTTTGAATGATATCAGGTTAGCTCTCAAAGCTTTCACTGATCAGAAAAGGCGTTTTTTCCCACATCTTGATGATGGACTTGAAACTAGTGAATCAGCATCGAAGAAATGTTGTGGGTCTGAAGAAATTGCAGTGGATCCCAAGGAAGGAATTAGTTTTCTAAGGACGCTGCCCGATGTTCTGAAGTCCTTGGAGAAGGATGTACCAAATCTGAAAATATTAACTTTTGAACGATTGGACTGGTTAAAAAGAGCTTCCACACTTACCTTGTCAACAAATGAGAATTATTTGGAACATAACTATCATGGTTCTAATAAGCTAAGGCTAGGATCAGTGGGAACCGTCCCTGCAGAGAAGGTTGCAGTGATAGAATTGTTATTCCCATGTGTCTTCAGGGCTGTTATATCCTTACATCCTGCTGGTTCCATGGATCCAGATGTTGTAGCTTTCTTTGCTCCAGATGAGGTATGGTTGCCAATATTATTGTGATATTTATCATTCTCTTTCCTAATATTCCTTTTGGTGGTGTGAACTGTTTGGTTTTCAGCAATTCAGCATTCAGCATTCTATGGACATACTAGATGATGTCTTAACATCAGTTCTTTGATAGTTCCTGTAATTGATGTGATTTTTGTTTTCCTGTCTCCTGAATCCCTAGTTTCAGATAATGAAGCTCGTATATAAAGCCCATATTAACTCTTGTAGCTGTAAACTTCATTTTCTGATTGCAATGTAAACTTGCTTGTCTACAAGATTCCACTACCTTCTTGAAGCTGTTTTTCTATTCATTTCTTGATTTATTTTCATAACCAAATTGGTTTCAGGGTGAACAGGCTTGTGGTCATCCACCATAGTCATCATATTTTTGTCTTTATCTAATCTAAGATTTCATTTATTTATTCAACCTCACTATACATGTATTTGTTCTGTTAGATAGTCTACTCATAATATATGTGTTTTATTATGATTCTGTAGATGCAAAGTTTTTAAAGTTGTCATTCTTCAAATTGTGTCTATATGGAAATCTAAAATTTTCATAGGGTGAGCCTTGGTGCAATAGTAAGGTTGCTGCCTTGTGACCTGGGGGAGTTTTTAAAGTTCTCTCCAAAATGACTTATATTGTAATAAAAATACTTCAATTTTCTTGAATTTTCCATCTACATATTCCACTGTTTTATAGTTTATTAATACCTCTGGCTCAGTGTATTTAACTGTGTACTACTTAACAGAGCGGAAGTTATGTGCATGCAAGGGGGTTTTCAGTTCATCATGTGTTTAGACATATTACGGTAATGGTCTCTGTGGCTCCTTATTTCATTTTATTCGTTCAGTTACTCTTTGAAGTCTTCATGTATATAGCTTTTTTATCTTGCACTACAGGAGTATGCAGCGACAGCTCTGCAGTATTTTCTTGGGAACCAATCTGAAACGTGTCTGTATTTTCTTCTGGTACTGTAATTATCTATTTCAAATCATAATGCTTTTTTTGGCTTGGAATGTATTAATTCTCGATATACTTGAGGTTTGCTTAGCTTTCTGCCATTTTTGTTGTAGCACTGGATTTGCAGCTACCAGACACTGTTTTCAAAACCTTGCAGGTTCCATTGGCACTTTCTAGAACTTTAAATTTTCCTATCATTTGGTAAAATTACTAATCTCAATTTGTTGGTGGTGCAGCAAATGTTCCCGGCTACTTGCAATGGATAAACAATCAACTTTATTGTTACCTCCAGTCCATCGTCCTTATTGGCAGTTTTCCTTTTCTAAAATTTTGTTAAACATATCCTCAAAGGACCAGAATTCTGATACTACTCAGGCTTATCATATTGACTGCCTCTCTGAGGAAACATAATTGCATCTACCAGTGACTCAGGTATAACCCAAGTGCCCGATTCAATGTGGAACTACCAGGAGCAAGAGTTTCCATTTCATTGATGTATGTATGTTCCCCTTTTCCTGTCATGTATAGGTTGGAAGGTAAGGGGTTGGTGAAGCGATTTGGTTATCTTTGGTCTATATTTTTCATCTCTTAGATTGGAATTGGATCTTCAGGAAATTGACGAGTGCCTTGCTTGTTTTGTAAGCCTCATGAAAGTGGCAGCCATCTGTTCTAAAGATTTCTCAGATATTCATTGAGATTGGTTTTTTGGATACCAAGAAGTTCTCACGTATAATTGCCACTAGACCAAAAAGTACAAGATAGGAACATTATGACCTGCGTATGCTAAATGAAAGTGCCAGCTGACTGTAATTTTTGAAATTTATCAGCATGTGAATACATTATAATGACCGTCTGCAATATTTCTTATTTTAAAATGATGCTGACTACGATGTAGTACACAACTACCTTCTCCGTATAATAGTTATTATATAATCACT

>GmMED3-2

TCAGTCGTGACCTAACAAAGCTGGTGCTTCTTTTTCTCTGCAGCAGCCTCTTCGATCCCGCTGGTGCTCACTTCGATCCCGTTGGCGCTCTCTTCGATTCAGGTTTGTTAATCTATCTTATTTCTCTATTGTTCGATTTCTTGTTGTTGTTGTTGTTGTTGTTGTTGTTGAGTTTAGGGTTCATCCTTTGTTGTTTGGGGGCAAAGGTTGAATCAGTTTTCTTTTGTTTTGATGATGGTGCTCGATTATTAACTTTAATTGTGCAGCCTTTGTTTTGATGATGGGTTATTGGATGATGATCTTGGTTGGTAGCAACGTGGACAGAAGCTATACATTTCAACATTTTATATATGTGGATATGCTACTCTCATCAGGAGCTCGATTGGTTTTTGTGGGAACAATTGCTTAATTTGCATTTATTTGCACAGAGCACTAGTCTTTGGGAGGTTAGGCTGATTGTCTATGGGTACTTCTAGTATGGGTAGGCATGGCAACGGGCCTGAACCCGTGGGGCCCACCCTGGACCCGCCCTGATTTTGACGGGGAAAACCTGAGTTGACCGGGGTCGGGGTCGGGTTTTCCCCAATAGCCAAAGTCGGGTTCGGGGATGACAAACCAATGCAGAAAAATATACTTTCATCTTTGTGTTGACAAACATTATAGAATGTTAATGATAAGAAAACTTTTCGCAAATTGCTGGGATGAAGGGAGAAAAGTTTTTTCAATGCAGATTCAAATTCAATATCTACAGAGTTTCTGTTTGACTTGAATGATAACAAAATAAGAACTTTGACACAAGTTCAAATTATGAATGAAGTAAGAAAAACTTCAAATTCAATATCAATACTAGCTTCTGTTACAGGTTCTAATTCAATATAATGTTACTCATTGATAATAGGAATTATGAATGAAATAAGAAAATTTAAATCATTTCGATTAGCAGAAGATATTACTTCATGCTTACTACAAAGTCTAATGTTACAACATAGTTGTGCTCAGCATAACTAATGTTACAGAAAATTAATGATATTCTTAATTGAATGAACCGTTTTCAAGTAAGTTAACAAAGCAAATAGGAGGAAGATCAAGTGAATTCCAAAGTAACAGTTCCCCATCAACACTTGAAGAATCCATGTCAGGTGATGCATGTTGGTGTTCTTGTTCCTTTTTCCTATCCATTGTTGCCATGAACTCTTTCTCCTCCTTATTCTTCTCAAGCTTGTTTTTGCTACAAGCCATTTGAAGGCTTGTTAATTGATCAGCTAGTACTCTTCCCTTCATGTTTCCTGATTCAAGTTTCAACTTCAAGCATTCAGGTATCCATTGAAGGCATAGAGAGAAGCTGAGAACTTCCTCTCATAAGTCATTCTCTTCATCACAGCTCTAAGCATTGAAGGCTCGTCATCGTAGTTCTCATTCTCAGGCTCCACAGGTTCCTCCTCCATCTCTTCAGCCATCTCAAAGGGAGAATAGTAACTAGGTGGAACAATGTTGTCCAAAAACCTAAAATCATGGACATCCAAATTAGTACTTTGCACACCCAAATCATTTCCTTCTTCTTCACTCTCCACCACTCCTGAATTGCTGTCATTGCTTGAAGTCTGTGGTGTCACATTGCTTGATTTGTATTGCAATTGCATATCCATTTTTGTCCCACCATCTTTTTTAGTTAAGCATGACTCAAAGCTACTAGTAATGTAGTCAATTTGTGCAGTGTTGTTGATGAAGTCATTGTTGCTTGTGCTATAATCTTCAGGGTCATTGAGGAAATCAGTGAATTGGTCAATTGAGAATTGTGTTGAACCTTCTCCATATGCATCAACCTGTTGGTGGTGTTGCATTTGTTGGTGGTTGATGAGAAGTGAGGTGGACGAGGACGAGGACGAGGAACAAGCGTTGTTGTTGTTGTTGTTTCTTTCTTTAAGCCTTTGAAGGAGGAGATTGGTGATCTTTGAGGAAAGAGCTGGACTTGTGGAGGATTGAGAGCAAGGCCAGAAGTTAGTCCTCATGTTGGCGCCGTGAAGCAAGCAAGCAGCCTCATCATAGGCTCTTGCAGCTTCCTCAGCAGTGTCAAAAGTTCCCAACCACACTCTTATCTTCTGAATGGTGTCTTTGATCTCAGCCACCCATCTACCTGATGGCCTTTGCCTTACACCAACAAACCTCTTCCTTGCTCTTTGCACCCCTCCAAGTGCTGCAGCTTGTTTCATCATCTCATCCCAACCCAAGGTTCCCTCACATAGCTCTCCCTCTTCCACCACTTCAACAACCTTTCTCTTCCTTGCCATCTACCCCCTTTGAACTCTTTTTGTTTCTTCAATTGGTTAGGCTTATAAGGTGATTCTTTAAGGGTACTAAACTTGGTTTGTTTGGTTAAACCCTTAAACTTATAAGGTGAGTATTAGAATTTAGAGGACACTGAAGTTTGGTTGCTATGCTTTGCCTTAGCTGGTTGGTGCTTTTATATGTGGTTTAAGGAAGTGAGTGATCCTTTGAACTAGTTGAGTTTTGGATATGACCAAGTCACACTGATTAATTGGTTTCATATATTTGTTTCTTTATGATTTCGGTTTCTATATCCAAACATTTGGCGGTTTCTCAGGCTTTGTAATTAGTGCAGCTTGTTTTGACTTGGCCAAAGGGTACATAGATTTATTTCTCTTGTGAGATCTTGTTTGAGAACTTCAGCATATTCTATACCAAAGTGTAGGCCTGAATTGGTCATTACAACTTCTTTTAATCTTTTCCATGTGATCCAAAGCTAGCTACAGACTAAATTATTGTGATCTATGATCTTTCTTTAATTTCAATTTTTATTTTCTGCCCAAATTCTACCACTTTGTCCAAGGCAAGAAGTGAAATGTGAGACAGAGTTTCAGACTCAGTTGCCAAGAAAGACACCAAGAAAAACAACCCGAGTAGCTAAAACCTATGGGCTTATACAGAAGTAACTAGAATACTTGTCCATCTACTGTTAATGCTTGGATTTTACCTTAATAGTTTGATTTTTATATTTCATTATCAACTCTGATTACCCATAACTAGATTTGTTGGTTGTTTATGAACTTTTTCTACGCATCCTTTCATATATGTTTGGTCTAGCCAATAGAATGAGAGTTAAAAACAAAAGTACATTGAAGATTGGTAAGTTTAGGAAATCAACTAATCAAGGCTCTTAGATACTTATTAACACTTTTCTTATCCTAGTCGAGAATGAGAGATATCTTTTAAGGTATAAAAATCATTTAAGAAACTGATTTCTTTCGACTGATACTCGTTTATACACATGAACTTGAAAATCAAATTTCTAACTATATACTTAAAGAACATGATTATTTATCAATTATATCACACTATATTATACTATATTATTTTTTGATACTTATTAACTAGCAAACAGAAATATTTACTCAACAGTAATAGGAAAAGTCTCTTTCATACGAAAACAAGGTCCCTTCATTATTGTTTATACACCCCCGTTGGGTTTCGGGGGCGGGGACGGGGAGAGAAATGGGGAGTCGGGGGCGGGGGTGGGGTAAGCAAAACCCGACCCCGACCTGCCCCGTTGCCATGCCTAAGTATGGAGCTCTTAACATTTAAGGGAATATTTTAGTTTTTATCTGTTATCTTCTCTGTTTAATTGAGCTTGGTATAAGTTTATTGATATACCTTTCCTTGTAAATGTCTGCCATGAAATTAATATGTTACTCATTTAAAATAGTTTCTTAATTGCCCTTATTTTTTTAAATGATTTAGATGAATGACATATTTTATTATTTTATAATATGCTTATGTTTTTATCATTTAAAATATTAGAATACCTCCCAAAAAGGACTGTGCTAAGTGGGATGACATTGACAACAAAATTGAATTTTTTTGAGAATATTTATCGAAGAGATAGATACTGGGAACAGACTAGGAATGAGTTTTAGTAAAGAAGGGTGGAAGAATTTAGAGAAAAAATTCCAATTTAGAACAAGGTGTGCTTATGACAAGATTCAATTAAAAAACAAATAAGATGCGTTAAAAAAGGGTTTTCAATCTTTTGCTAAGTTGGTTGAGAAAGACACCCGTCTTGGTTGGAATTATGAGAAACAAACCATTGTAGCAGATGAAGAACTTTTGGCATTTGCAAAGCAAGATGGAGAATATTGCAATGCATAACCAATTTTAAACTTGAAACTCAAATCTCTATTATTTTGCTGTAACATGATTTGTTATTGCGATTCAATTTTTTTTATACTAAAATGTCCTTTTTTTTTCAAATGGGTATCATTGGATGCTTTTACTTTTGTCGGATCCGGTTAAATTTTCCTTTTTGATCTTGCCCTTATAAGTGTTTGTGCTTTTTTTTCGTTTTCGAATGGTGGTTTTGCTTTGGAAAAATCTTCTGTGCAGATTTTGGGTGTTAGCATTGAAAGGTGTTGATTGAGTTCCATTTTATATGAGTTTCATCTTTTGCCTTTTGTGGTTTAGGCATGGATCTTGTAGTTCATGACATGTATTTGTGTTGTGTAGTTGCTATGACTATGAGCATTCAACTTTTGTGTTTGTTTGTTTTTGAAAAAGTTTGATGGTTTATTTAGATAATGCAAATTTGGCGTTGTGGGGTGTGTTCTGTTTGGTGGATGATTTTTACTTTAGGCTAATAGGTTTGTGCTTTTGAAAGAAAGAGAGAATCAACGGGGTTTGCAGTGAGGTGAAAGATTTCAATTATGTTTATTCCTAGAAGTGAACTGAATTTTGTGTTAATTTGTGTTTCCACTGTACTCTTTCAGCTTCATATATATTGTGTTCAGTCATTAGTTATAGTCTTGTAGACAATGATACCAACTTTAGTTTTACTTGATTGGTGAGATGAAACAGAACATATATGCTTGCTTTGATTTGTATGCATTTTGTGTCAATAAAGCCCTGCATAAATCTTGAATTCAGTAATGTATACTGTATAATATGTGTTTTTATTATTTTTTAGATATTGACTGGTTTGATATACAACACTGGCTGATTTGAAATGGCTTAACTTGCAAGCATAATTTGTACGTTGGTTTAAGTAATTAATGTGGCCCAATCCGAGTTGTACGAAAATACTAACTGAGTCGAAACCCACTTTCTTTTCTTTGTGGGTGTGCCACGGCCAAATGCAGATGCAGATGCAACAAACGCAGCAGCAGGCCACGGCGACACCATTCTCGACCTGCACTCCTCCTCCTTCCTCCGGTGGCTCCGCGTCCGAGGCACCGCCAAAGCAGGTGGCTCAAGCAATGGACAAGCTCGGACAGGCCGAACGAATCATCGCCGACATCCGAATCGGCGCCGACCGCCTCCTCGAAGCGCTCTTCGTCGCGGCGGCGCAGCCTCACCAAGGCAACAAGCCTCTCCAAGTCTTCCTCAAAGAAGACGCATGCATGCGTCAGTATCTTCAAGACCTTCGATCGCTTGGTAAGCATGCCTTTTCACTCGCCCCTTTTGTTTTTTAGGTTCCGTGGATTAGGGTTTTGGATTGGACTCTGCGACGTCGTTTGTGTGATTAGTTCGTCGTGGTTTTGGTTTAATAGGTAAGGAGCTAGAAGAGTCTGGAGTTCTCAGCGAATCAGTTCGATCGAGGAAAGATTTTTGGGGCTTGCATATGCCACTGGTTTGTCCAGATGGCGCTGTGGTTGCGTATGCGTGGAAACGACAACTTGCAGGACAAGCAGGTGCTTCGGCCGTTGACAGGACCAGGTTCGATTTGTTCAATTTGGTTCTGTTTCAGTTCTCTCGTTCAAATTTCTGTGTAGTACATTTAGATAACGGAATAGGATGGAAAACAATGGATTCCAATTCAACTGTCTTTTCTTGTGCTTAGAGTTGATAATTGCTATGGTGTTATTAGCGATTTATTTATTTAGTTATTTTTATGATTTTTGTGCATTCTTAGGTGGATATCTTAATCTTAAGTTTTGGTATTACCTCCTGAATTCCTAGCACATGGTATTGATAATATGAATCTTAGCCAAGTTATGCTGTGATATAGCTGTCTCGTACCTAAGTTGTGTAGAAATAGAGAACTAGTACTACGCTAGAAAACAGTTTTTTTATTGGGTATTGTGGTTTATAATGATCGTGCTGACTTTCGAATCTCAAGTCTGCTAAATTGTGGTACTTTAGTTGCTCACTTAGGCTTTTGAATGATATCAGGTTAGCTCTCAAAGCTTTCACTGATCAGAAAAGGCGTTTTTTCCCACATCTTGATGATGGACTTGAAACTAGTGAATCAGCATCGAAGAAATGTTGTGGGTCTGAAGAAATTGCAGTGGATCCCAAGGAAGGAATTAGTTTTCTAAGGACGCTGCCGGATGTTCTGAAGTCCTTGGAGAAGGATGTACCAAATCTGAAAATATTAACTTTTGAACGATTGGACTGGTTAAAAAGAGCTTCCACACTTACCTCGTCAACAAATGAGAATTCTTTGGAACATAACTATCATGGTTCGAATAAGCTAAGGCTAGGATCAGTGGGAACCGTCCCTGCAGAGAAGGTTGCAGTGATAGAATTGTTATTCCCGTGTGTCTTCAGGGCTGTTATATCCTTACATCCTGCTGGTTCCATGGATCCAGATGCTGTAGCTTTCTTTGCTCCAGATGAGGTATGCCTGCCAATATTATTGTGATATTTATCATTCTTTTTCCTAATATTCCTTTTGGTGGTGTGAACTGTTTGGTTTTCAGCAATTCAGCATTCTATGGACATACTAGATGATGTCTTAACATCAGTTCTTTGATAGTTCCTGTAATTGATGCGGTTTTTGTTTTCCTGTCTCCTGAATCCCTAGTTTCAGATAATGAAGCTTGTATATAAAGCCCATATTAACTCTTGTAGCTGTAAACTTCATTTTCTGATTGCAATGTAAACTTGCTTGTCTACAAGATTCCACTACCTTCTTGAAGCTGTATGGAAATCTAAAATTTTAATAGGGTGAGCCTTGGTGCAATAGTAAGGTTGCTGCCTTGTGACCTGGGGGAGTTTTTAAAGTTCTCTCCAAAATGACTAATTTTTTAATTGTAATAAAAATACTTCAGTTTTCTTGAATTTTCCATCTACATATTCCACAGTTTTATAGTTTATTAATACCTCTGGCGCAGTGTATTTAACTGTGTACTACTTAACAGAGCGGAAGTTATGTGCATGCAAGGGGGTTTTCAGTTCATCATGTGTTTAGACATATTACGGTAATGGTCTCTGTTGCTCCTTATTTCATTTGATTCGTTCAGTTACTCTTTGAAATCTTCATGTATATAGCTTTTTTTATCTTGCACTACAGGAGTATGCAGCAACAGCTCTGCAGTATTTTCTTGGGAACCAATCTGAAACGTGTCTGTATTTTCTTCTGGTACTGTAGTGATCTATTTCAAATCATAATCCTTTTTTTGGTTTGGAATGTATTAATTCTCGATATACTTGAGGTTTTCTTAGCTTTCTGCCATTTTTTGTTGTAGCACTGGATTTGCAGCTACCAGACACTGTTTTCAAGACCTTGCAGGTTCCATTGGCACTTTCTAGAACTTTAAATTTTCCTATCACTTGGTAAAATTACTAATCTCAATTTGTTGGTGGTGCAGCAAATGTTCCCGGCTACTTGCAATGGATAAACAATCAACTTTATTGTTACCTCCAGTTCATCGTCCTTATTGGCAGTTTTCCTTTTCTAAAATTTTGTTGAACATATCCTCAAAGGACCAGAATTCTGATACTACTCAGGCTTATCATATTGACTGCCTCTCTGAGGAAACATAATTGCATCTACCAGTGACTCAGGTATAACCCAAGTGCCCAATTCAATGTGGAACTACCAGGAGCAAGAGTTTCCATTTCATTGTTGTATGTATGTTCCCCTTTTCCTGTCATGTACAGGTTGGAAGGTAAGGGGTTGGAGAAGCAATTTGGTTATCTTTGGTCTATATTTTCCATCTCTTAGATTGGAATTGGATCTTCAGGAAATTGACGAGTGCCTGGCTTGTTTTGTAAGCCTCATGAAAGTGGCTGCCATCTGTTCTAAAGATTTCTCAGATATTCATTGAGAAGTTCTCACGTATAATTGCCACTAGACCAAAAAGTACAAGATAGGAACATTATGACCTGCGTATGCTAAATGAAAGTGCCAGCTGACTGTAATTTTTGAAATTTATCAGCATGTGAATACATTATAATGACCGTCTGCAATATTTCTTATTTTAAAATGATGCTGACTACAATGTAGTACACGACTACCTTCTCCGTATAATGGTTATTATATAATCACTACAAAATTACAGTGTATTGCACAGCTACCTTCTCCATATGTACTAGTTCT

>GmMED4-1

CCATCATCTAGCAATTTAACTAGTGACTTTTCAATCTCTGATACTCCACAAATTTCTTCCCTCAGGTCTCTCTCTCTCCTCTCACACCGCGTCACCGCCCCCCCACTGTCGCCGCCGCCGCCGCCACCCCCCCTCACAGTTCCATTCTCTTCTCGTTTTCCCTTCGTCTCACTCTCACACGAAAACCAGTTCTCGTCGCGGCGGCGCTCTCGTCAGTCGCTGCCGTCGCCGCCTCCGTTGACGTCGGAGGTTCCTTCAACACCAAATTAAAAGGTTTTTTTGTTTCTTGAGCTCTGATCTTTTGATACAGTGTTTTGATTATTATTTTTTGCTTCTCAGTGTTTTACAGATTTGAGATGAATTGTATGCCTACATTAGTGTATTTCTATAACTTTGATCAATTCATGTGATAATGCATTTATGAATCATGTCTTCATTTTTATATAACTTTTGTTTATGCAAGGGGGCACCCCCTACAAGTTTTCTTTCTCTGCTTAGGTGTTTGTATGGTACAGCAAGCAGTATCCTTCGCTGTCCATGAAATTTTTAGTTTTTAAATTTCATGATCCGATTGACCCAACCTGTTCATGACCAAGATGAGTTGGGTTTGGATGAAAAATCACTGGAACCCGATTCAACCCAACCCGAGGTTTTTTAATTGGGTTGGGTGATGAGTTTTCTCAAACCTGACCCAACTCAACCCTTGTACAGCCCTATAATGGTTAGTTAGTCTTTTCCTCTTAAAATCTGTTAAAAAGAGACCTTAAAAATCATTTTTTTTATTGCAATTGGCTGGTCTCTTTGAAGGACGGTTCTTGCACATAAGAACCTGCGCCAAGAACCTATTAGTGAATTGGGTGGCTGTTGTTAGCTTGTTATTATATTTTATTTTAATATTTCTGAAAGTTACAATGGATGTACTATTTGCCCGGTTTAGTTTAAAGTACAATACAACTGTTGGGCAGCAAGTGTTTGATTCTTTTGCTTGTTAGTTTTTGGACTCTTTTTTATCCATCTTTGTGTTTCCCCTTTGGTTCATTACTTTGTGATTCTTAGTGTGTTGGATTTATTATCTTAGCTCCTGAGAAACTCATATTGTTTTTCTGATGCTTTTCTGCTTTGTGCTTCATTCTTAGTGATGTCTTTTACTTTTATACCACAATGATTCAGTGAAAGGAGATAGTGCTTTGTGTGCCTTGAAGTTCAATTACCCTTCAACCTCTGGACTTAATTTTCCTTTCTTCTTCCCTTCATTCCAATCTGTTACCCATAGTGTTTTCTAGAACCACAAAATGCTTCAACACCAAATTGTTCAGTCCCCTGCTATGCTAGGCCTTGCAAATCCTAACTCCCCATCAATTCCGAACCCTACCCCTCCAAAGCTCCCTCCAACACAGACCCATCACCCCCAAGACCGCAACTCTTCAACCCCTTCTTCAGCTCTCTTGTCTCTCCTCCCACCTCACCTCAGAGCCCAAGCACTTCTTGCCCAAATGGCTTCCTTAGCCTCCAAGCTCTTTGAAGTTTCCCCCAATCGGTCCCTTTGGGTCACTGCCTTTCGTGGATCCCTTCCAACTTTCCTCTCTTCCCATTCGTCTACCCCACTTGACACTTCTCCTTCTGATGGCAAAGAAATCATCTCCCTTTTTACTGTTCTCCAAACCCAAATCTTTGAAGCTATTGCTGAACTCCAAGAGATTCTGGACCTTCAAGATGCCAAGCAAAAGATTGACTGCGAAATTCACTCCCAAGATTCAACACTACTTGCATTTGCCAACAAACTCAAAGAAGCCGAGAGTTGCCTCGACATCCTTGTTGATGATTACTCCGATTACCACCGCTCCAAGCGATCAAAATCTGGAGATGATGATTCAATGACTTCATCAACCATCTCGTCTCAGCTGAAGCTGTCGGATATGTTATCATATGCCCACCGGATAAGTTACACCACCTTTGCACCACCAGAATTCGGAGCTGGCCAAGCTCCTCTCCGTGGCGCACTGCCACCTGCACCACAAGAAGAGCAAATGAGAGCTTCACAGTTGTATAATTTTGCTGACCTTGATGTTGGATTGCCTAAAGAAGTTGAAACCAAGGAGAAAATTGTTGAAGCTATTGTTGAGCCTCCACCACAGGTGGATACTAATACAGTTCCGAATTTGTCTGCATTTCAAGGGATGTTACCTCCGATGCCACCTGGCTGGAAGCCGGGAATGCCTGTGCAATTGCCTATTGATTTGCCTCTTCCCCCGCCTGGGTGGAAACCAGGGGATCCTGTGCCGTTGCCTCCCATGGACTCGCTTCAAATACCGAGATTTGCAGAGCAACAAATGCAACCTCACATTCCTCAGCCCAAGCAACCAGAAGTTATTCAAGTGCAGCCAGTTAATTTGGACCTTGGAGGAAGTGATACTAGTGATTATAGTAGTGATGATGCCAGCTCTGATGATGAAGATTGACAAGTTTGTGAAACTGATCAATCTCAAATTAATAACTCTACAGGGTGAGGGATTTTGCACTTGATTGTTATGTCACTGTCAAGTGTCATGTTATATATACTTCTGTCCATTGTTTACTAAGCATTGAGCTTTAATATGATGTTTTCAGCATCGATAGGCTGAGTTGATTGAAGTAATGACATAGCATTTCATCACCTTGTTGGCCTTTTTAGTATTTTTTGTGTAATGGGGACATATCCTCTGTCTCTGCTGGGGGCTTAGTGTTTCTGCTATAGGCCCTAGAAAGCACAAAGCATATCTGTGCTTTTATAACACTGAAAATATCAATAAGCTTTGCGCCTATACTAGTGGAGGGTTGTGTTTTCCAAGCTAAAATGTATCTGGTTCTGATATAGACAATAATGTAAGCTGCTAATATATTACTATGAAAAAGAATATACATCTTATCGGCC

>GmMED4-2

TAACTTGATGTTTCCTCCATCATCTATTAATTCTATTATCTTCAAAATAATTACACTGTACAATTGAAATTACCTCTCAATAACCTCTTCTAATTTTTTTACACCCGTTAATTGTGGAGTTATTTTAGAAATTTAGCAGTACTTGATTTGGGATTAGAAATAGGAAAAGGCTCGGATCTCAGAAAGAAAAGGAAAGGAAAGATCTAGAAACTTAGGAGTTTTAAACTTTCTCTATCACAGAATACAAAACCCTAAAACTACCAACGTCACCCAACCACCACAACCTCCCTTAATAGCCGCCGCACCACCATAACCACCAACTCCCCACCCGCGACCAACCTCCGACCACTACCGCCGCGCCGCATCGTCTCCGACGCCAACCAGAACCATCTCATCCACCCTTCATCCGCCATCTTCATTCTTAGGAACTTTGGCTTCGAGCATTTATAAAGGTAATATCTCAATTTCAAATTTGTTCTGTCACATTGATACTAGAAGGTATATATATATATATATATATATATATATATATATATATAAAGATAATAAGTCTTAAGAAAGATGTGAGCCATCAGAGATTAGCATATTGTACAGAGATCAAACTTTTATATTTCGTAGTAACCAATTACTATATATTGTCTGGAATAGATATTGAGAAGAGTCTAATTTTTTCTTTTGTTGGGAAGGTAACTGATGTTCTCTGTCATGTAGTGATCTTTCTTTCAATCTTTTTGAATTGCTTTATGGCTTGTGTTGCATTACATGAGTTACTGCAGTGTAGTTTTGGTTAACTTATGGTGCTCCAACTTAAGAGAATTGGTCAGATAAAAGCTTACTGTAAGTTGATGTATGCAAATACTGTTTGGTCCTTTTGCTTTGACATTCAATGTGCTGCTTAGTTTATGAATTGGGATTGCTATTCAATTTCAGAAATCTTTTTATTTTTCACATTCTTTGACCATGTGTGCTACTGATTTCTGTAAAACAGAGTGGGTTTTCCTGTTGTTGACTGAAATTGAACTTTATATCAGTACAATAGCATCTAACTGCATCATCACTGATGCAATTGAGTTTCCATCTAACTGCATCGGTTAATTAGTCTTAATGTCAAATATGTTTAATGCTATTATGGTTTAATTAGCGAATATATGTTGCCTGCAATCAAGCACTTGGATGTAGGAAATAAAGGTCTTGGTTGGTGTAATTTTGTATACAAAACTAAAAGGGAAACATGACATTTATAAAAATTAATAGCTTGCAGGCATATCAATGAGCATGCCTTGGCTATTTATATAAAAGTCTTGAAAGAACATTGAATGAGTATTGATTCTGTGTTTGTCGGTTTGCTTGGGATTAAATGATTCTGAGATTTAATATGAGTTTATTAATCGTTGATTTTCAGTGTTGATTATGTTCTTTGAGAGTTAATTCAGAATGATTTGTTTTACTTTTTTATTCTTAAGAACCAAGTTTGTCGGGATGAACAGTTCTCCACGCTGGCGATCTGAGACCTGCTCTCCAAGGGACGAACACGGACGGGTGGCTTTGTCCTTGTGATGCTTTTAGTTCCTGAGAAACTCTTTTATTTATTTTTATGATGCTTTTCAGCTTTGTGCTTCATTCTTACTTCTTAGTCGTTCGTCTGTTTGATTACTTAGTATGTTTTGGTAGCTATATGTTTTGATAGTGTTTCTGAATTGTGATTTTGTTGATTCTTCAGCATTTCACATTTGTAGATTTGTGATTCTATGATTTCCAGCAATTCATTATGCTACAGTTTTACTAATTTTCTCAATGTTATTGCTTTTTTTTATCGTCTTGCATTATCGTGCTACAATTTGTTACTGGCTTAAACATCAAGGTTGTGTTTCAGCCTTTTAGTTAACATCAAGGTTGTGTTTTCGTTGCAGGTGCAGAGACAGAACATTAAAGCTGCTGGTGCTTTGATTCTGCTATGGAACATTGAGGCAAAGCTCAGCATCTTAAACCCTGTAACTGTGAGAGTACAGTCTCTTGCTTGCGCCTTTGCAGTGAGAGTGATGTCTTTTATATACCATGGTGATTCGGTGAATGGAGATAATGCTTTGTGCGCCTTGAACCACAAAATTAGTATTTTCAACCTCTACACTTAATTTTCCTTTCTTCTTCCCTTCATTGCAATCTGTTACCTATAGTATTTTCTAGAACCACAAAATGCTTCAACACCAAATTGTTCAGTCCCCTGCTAGGCTAGGCCTCGCAAATCCTAACTCCCCATCAATTCCAAACCCTACCCCTCCGAAGCTCCCTCCAACACAGACCCATCACCCCCAAGACCGCAACTCCTCAACCCCTTCTTCAGCTCTCTTGTCTCTCCTCCCACCCCTCCCCAGAGCCCAAGCACTTCTTGCCCAAATGGCTTCCTTAGCTTCCAAGCTCTTTGATGTTTCCCCTAATCGGTCCCTTTGGGTCACCGCCTTCCGTGGATCCCTTCCAACTTTCCTCTCTTCTCATTCGTCTACCCCACTTGACACTTCTCCTTCTAATGCCAAAGAAATCATCTCCCTTTTTACTGTTCTCCAAACCCAAATCTTTGAAGCCGTTGCTGAACTCCAAGAGATTCTTGACCTCCAAGATGCCAAGCAAAAGATTGACCGCGAAATTCGCTCCCAAGATTCAACACTTCTTGCATTTGCCAACAAACTCAAAGAGGCTGAGAGCTGCCTCGATATCCTTGTTGACGATTACTCTGATTACCGCCGCTCCAAGCGATCAAAATCTGGAGATGATGATTCCATGACTTCATCAACTGTCTCGTCTCAGCTGAAGCTGTCGGATATATTATCATATGCCCACCGGATAAGTTACACCACCTTTGCACCACCAGAATTTGGAGCTGGCCAAGCTCCTCTCCGTGGCGCACTGCCACCTGCGCCACAAGAAGAGCAAATGAGAGCTTCGCAATTGTATAATTTTGCTGACCTTGATGTTGGATTGCCTAAAGAAGTTGAAACCAAGGAGAAAATTGTTGAAGCTATTGTTGAGCCTCCACCACAGGTGGATACCAATGCAGTTCCGAATTTGTCTGCATTTCAAGGAATGTTACCTCCGATGCCACCTGGCTGGAAGCCGGGAATGCCTGTGCAACTGCCAATTGATTTGCCACTGCCCCCGCCTGGGTGGAAACCAGGGGATCCTGTGCCATTGCCTCCCATGGACTCGCTTCAAATACCGAGATTTGCAGAGCAACAAATGCAACCTCACATTCCTCAGCCCAAACAACCAGAAGTTATTCAAGTGCAACCAGTTAATTTGGACCTTGGAGGAAGTGATACTAGTGATTATAGTAGTGATGATGCCAGCTCTGATGATGAAGATTGACAAGTTTGCAAAACTGATCAATCTCAAATTAAATACTCTACAGGTGAGGGATTTTGCACTTGATTGTTATGTCACTGTCAAGTGTCATGTTATGAATACTTCTGTCAATTGTTTCCTAAGCATTGAATTTTAATATGATGTTTTCAGCATTGAAAGGTTGAGTTGATTGAAGTAATGACATAACTTTTCATCACCTTGTTGGCCTTTTTAGTATTGTATAATGGGGATATATCCTCTGTCTCTGCTGGGGGCTTAGTGTTTCTGCTATAGGCTCTGGAAAGCACAAAGCATATCTGTGCTTTTATAACCGTGAAAATATCAATAAGCTTTGCACCTATACTAGTGGAGGGTTGTGTTTTCTACCTGGTTCTGATATAGACAATAATGTGAGCTGCTAATATATTACTATGAAAAAGAATATACATCTTATCGGCCTTGTTTACATTTTTTTTGTGTGCCATAAAAATAATTTTTTTTAAAAAAAGCTTTACTTTTTCAATAAATAAAAGAATGTTTTATTCTCTAGAATATTCCTCCTCCTATATGCTCATTCCTCACTTAATAATTCCTCCCTCATCCACACGTCATAAATTTACATGGAGTGGTAAATAGTAATCATTTTAAACCACCATTTTTAATTCTTTCACCCTCTGAAAATGCTGCACTCATTGTATTCTGTTTCACCTTCATTCACCAAGAAACCAATGCACTGCAGTACACCAATGCAGCACACAATTAATTTTTCCTGGTCATCCATTTTTCACACCCTTCTGCATCCATCTTCACTCATACAAATACACATTAACTTTCTCATCACACATATTTTGGACGTGGCACAAATCATGAGACTCTCTCATGATCATATTCATTCGCAGCATACAGCACATGAGCACTCACCTGATTTTTTACTCCCATACTCATTGATGCTTTGATACATACTACACACAGGAGGGCTATTGAGAGAGAAAAGGACTAAGAAGTATGAGCAGTAGGGGCTGGAATTGAAGCATCCCTAGACATCTAGCTACATCAATCAATTTGAGAAAATAATCCATTGCCCATTGGTAACCCACCAAGATTTTGTTTCTTACTTCATTTACTTTTTTGTCCAAACTCAGCCTGTAGAAGCTAAATTTTTAACAAGTTGATTCTGATATTTTTCTGTGTATATATTGTTCTCACTTATTATTACTGCATTCTGTAGAGGTAGCTATTGAGTTCAATGTTTTGATTTGAAACCTCTGAATTAACGGAAAAAATAGGTTCATGGTTCTTCTATTTTATGCTGTTAGTTTGGAAATTTTTATTTGTAATTACAGCATAATGCTGTATCCATAATTACGGTCCTCATTTACGGTGTGTTTGGTAGAGAAAGAGAAATAATATTGGAGAGGAATTTTGTACTTTT

>GmMED4-3

GTTACTTACCACACTTTGTTAAAATTTCTTATGGAACCATAGCCAGTTAAGAAGAGTGATTCATCTACATCTTAGATAATTGTTAGGAATCCAGAGTAGAACATAGAGAAAAGAAAAGAAGGACTTTATTTATGGAAAAAAAATTGGACAGAACAAGGAAAAAAAACTATCTACTAAGGGTTCCCCTTCACAAAGGGTTTCCCTTTCTTGACAAATCTAATTTTCTGAATGGTTCTTTATACTCTGTTACCCCTATTTATCTAATTTATCCCTCTAAATCACTAGTTGCTCTAACTACTGAGATTTTTTCTATTTGACAGCTACAACCATTGAAGGCACGAATTTGATCTGATTTCACGAAGGCCGAATCGAAAACGAGAAATTGTAGACTCCAAATTGGTAAAATCTCAATTCTTAAAATCATCTCAAACCCAATTTCTAAACCCTAAACTCCAAATTGGTAAATCTCTCTTCTTTTTCTGAAGTGGGTTGCTTATGTAGTGGCTTCAGTGGGGGTTTTCCCAATACTACTGTTATAGTCTTGTGAAATTTTCTTTTGTTACAGTCTTGCAGAATTTGTATTATCCATTGATTTTGATTTTAATTTATTATATTCTTAGGTTTCTGACTCTCCTTTTGTTGACATTATATTGAGAGTCTAATTTGTTCTTGTGAATTTTATTTAGAGGTCCAATTGATCTGCATGCTCATTTGTGAAATTCTTGAAGCTTTTTTTTTCTTGGTTGTTTTGTGAAATCTGCTTTGAGGTTCTTAACAGGAATACTTTGACAAAACTTTATTTATTATGGTACACTATTTTGACAAAACTTATCTACCACATTTGGTGTAACAAGTTGTTTCAACACATGTAAGACTGCAAGTCAAATTCCGCAGCAAGGATGGAACAAACAGATACATATCAACATCCACTTTAGATAGGTAACAACAATGGCAGACTTATTTTTTAGCTTCTTTAGTGTCCATGGCAGGAAAACCATAAATCAATGTATTTGGGTGCTCTTTTCCTTCTTTGTTTATGGTACTTGGATGCTCTTTTGCTACATGGTGTCCTTGCGATTTGTTTCCTTGGTTCTACACTCATCAACCAGTCCTGCTTCAGCTGTTCATCCAATGTTTCATAAGAATTCCACTGCAGCTCTTTCTGACCAACAGCTCTTTCTGACCAACCTCCTTGTTACTGACCTGGAATATGATTATTTAATTTTCACCGACTGGGTGAAAGTTGTGTAGTAGCTTGAGCCCAAAGGACTTAAAAGAGCTTCCTTGATTTCCCTAAGGAGTAAGGATTCTACCTCAAGAGTTATGGTGCTGGTTTAAGTTGTTTTGAAATGAGAGTTAAGTTGTCTACACCTGCCCATTATATATATTTATGATTTTTCTTTGATTATATAGTTGAACACTTTGTATATGCGCAAGTTGCTTTACAGTTTTTTATATTGTAGAGGACATCAATCCCATCTTTGCCATGATTCACTGTATTATGTTTATAACAAGCTTTTTTATTTTACTGCATATATATAACAAGTTATTGATTGCCTTCAAGAATTCAAGTTGATTTCGTTTGTGCTCAATTTGAATATTTGTCTCTCTCGTTAAACTCTAAAATCTAACCTTTTTATTAGTTGTTTATCTCTAAGTGATCAAGTTTACACAACTGCGGTTACAGCTTTTAGTAACATTTAATGGGAAAACACATAAAAACGTTGTGGAAAACACATAAAAATGTTGTCAATTATTATTAAAAAATTACTTTTTAATTATGTTATTCACGCAAAGACTTTCAAAATTAAGAAAAGAAAATATTTGTTATTTCAGGTCTTGACTTGTTGTGAGAGAGTGGTGGATGGAAAAATGAATAGATAACAGACTAACATTAACTAACATTACTTCACACCAAACCCATCATCCGCACCGCACTGCCACTCATCATCGACCCACCCTTTCCCAACTTTGCGGAGCAGAGGGAGTTGCCGACACCACCCATCACCTGTTCTCGTTTTGCCTAACGATGGTGTCGTAGGCGACGTTGCCATTATCGTCGACAGTGACAGGAAGGATTTTGGATCCAGGTTGAGAAACTCAAATTTTTTTATGATGCTTTTCAGCTTTGTGCTTCATTCTCAGTCATTCATCTGTTTGATTACCTAGTAAGCTTTGTCAGCAATATGTTTTGATAGTGTTTTTGAATTGTGATTTTGTCAATTCTACAGCATTTCACATTCGTAGATTTGTGATTCTATGACTCTTAGCAGTTCAATATGCTACAGTTTTACTTGTTTTTGTGATCGTCTTGCATTATCATGCTACAATTTGTAACTTGCTTAAACAACTGTTTCAGCCTTTCAGTTAAGATCAGGATGTTTTCATTGCAGGTGCAGAGACAAAACGAGAAAGCTACATGGTGCTTTGATTCTGCTATGGAACATTGAGGCAAAGCTCAGCATCTAAAACCCTGTAACTGTGAGGCTATAGTCTCTTGCTTGCACCTTTGCAGTGAGAGTGATGTCTTTTATACCACGGTGATTCGGTGAACGGAGAAAATGCTCTTTGCGCCTTGAAGTTCAAATACCCTTCAACCTCCTAACTTAGTTCTCCTTTCTTCTTCCCTTCATTCCAATCTGTTACCTATTGTGTTTTCTAGAACCACAAAAATGCTTCAACACCAAATAGTTCTGTCCCCTGCTAGGCTAGGCCTTGCAAATCCTAACTCCCCATCAATTCCAAACCCTACCCCTCCAAAGCTCCCTCCAACACATACTCAAGACCGCAACTCTTCAACCCCTTCTTCAGCTCTCTTGTCTCTCCTCCCACCCCTCCCCAGAGCCCAAGCACTTCTTGTCCAACTGGCTTCCTTAGCTTCCAAGCTCTTTGAAGTTTCCCCCAATCGGTCCCTTTGGGTCACCGCCTTCCGTGGATCCCTTCCAACTTTCCTCTCTTCCCATTCGTCTACCCCACTTTGCGCTTCTTCTAATGCCAAAGAAATCATCTCCCTTTTTACTGTTCTCCAAACCCAAATCTTTGAAGCTGTTGCTGAACTCCAAGAAATTCTTGACCTCCAAGATGCCAAGCAAAAGATTGACCGCGAAATTCACTCCCAAGATTCAACACTACTTTCATTTGCCAACAAACTCAAAGATGCTGAGAGCTGCCTCGACATCCTTGTTGATGATTACTCTGATTACCGCCGCTCCAAGCGACTGAAATTTGGAGATGATGATTCCATGACTTCATCAACTGTCTCGTCTCAGCTGAAGCTGTCAGATATATTATCATACGCCCACCGGATAAGTTACACCACCTTTGCACCACCAGAATTCGGAGCTGGCCAAGCTCCTCTCCGTGGCGCACTGCCACCTGCACCACAAGAAGAGCAAATGAGAGCTTCACAGTTGTATAATTTTGCTGACCTTGATGTTGGATTGCCTAAAGAAGTTGAAACCAAGGAGAAAATTGTTGAAGCTATTGTTGAGCCTCCACCACAGGTGGATACTAATGCAGTTCCGAATTTGTCTGCATTTCAAGGGATGTTACCTCCGATGCCACCTGGCTGGAAGCCGGGAATGCCTGTGCAATTACCTATTGATTTGCCACTGCCCCCACCTGGGTGGAAACCAGGGGATCCTGTGCCGTTGCCTCCCATGGACTCGCTTCAAATACCGAGATTTGCAGAGCAACAAATACAACCTCACATTCCTCAGCCCAAGCAACCAGAAGTTATTCAAGTGCAGCCAGTTAATCTGGACCTTGGAGGAAGTGATACTAGTGATTATAGTAGTGATGATGCCAGCTCTGATGACGAAGATTGACAAGTTTGTGAAACTGATCAATCTCAAATTGATAACTCTACAGGTGAGGGATTTTGCACTTTATTGTTATGCCACTGTCATATGTTATATATACGTCCTGTCTATTGTTTCCTAAGCATTGAGTTTTAATATGATGTTTTAAGCATTGATAGTTTGAGTTGATTGAAATAATTGCATAACTTTTCATCACCTTGTTGGCCTTTTTAGTATTTTGTGTAATGGGACCTATCCTTTGTCTCTGCTGGGGGCTCAGTGTTTCTGCTATAGGCTCTAGAAAGTACAAAGCATATCTGTGCTTTTATAACCGTGAAATAGCAATAAGATTTGCACCTATACTAGTGGAGGGTTGTGTTTTCCAAGCTACAATGTATCTGCTTCTAATATAGACAATAATGTAAGCTGCTAATATATTACAACTATGAAAAAAGAATATACGTCTTATCAGCCTAGTTTGCGTCTGGTTCTAATAATTGCATTTTTTACATTAACAAATGAAATATGCTCTACAAGACAGGTTTGTACCAAATTGTGATGAGGCACCTTGTTTACATCTTTCTTTGTATGCCATAAAAAAAATTAAAAAAGCTTCGCTTCTTCAATAAATAGAATAAAGTTTCATTCTCTAGAATTTGCCTCCTCCTATATGCTTATTCCTCACTTAATCGTTCCTCCCTCATCCACGCGTCATAAATTTACATGGAGTGGTAAACAGTTACCATTTCTCTCTCGTGGATACTTTCATTCTCAGAGATACTCTCATCAGTCATTTTAAACCACCATTTTTCATTCTTTCACCCTCTGAAAATGCAGCACTCATTGTATTCTGTTTCACCTTCATTCACCGAGAAACAAATGCACTGCAGTAGTCAATGCAGCACTTGTGAATTTTGCGTACTAATCCATTTTTCACACCCTTTAGCATCCACCTTCACTCATTTACATATACGCATTAACTTTCTCATCACACACTCATATTTTGGATGTGGCACAAATCATGAGACTCATGATCATATTCATACACAGCAGACAGCACATGAGCATTCACCTGATTTTTTACTCCCATACTTATTGATGCTTTGATACTACACACAGGAGGGCTATAGAGAGAGAAAAGGACTAAGAAGGATGATCAGCTGGGGTTGGAATTGAAGCAATCCTAGACATCTAGCTACAATAATGAATTTGAGAAAATAATCCATTGCCCGTCGATAACCCACCAAGATTTCATTTACTTTTTTCGTCTAAACTCAGCCTGTAGAAGCTAAATTTTTAACAAATTGATTCTGATATTTTTGTGTATATATTGTTCTCAATTCTTATTATTCCTGTATTTTGTAGAGGTAGCTATTGAGTTCAATGTTTTGATTAGAAACCTCTGATTTAAAAGAAAAATAGGTTCATGGTTCTTCT

>GmMED5-1

ATGAAATCAATTGATGAAGTTCTTCAGCTTTCTCAAGTATACAGTCAAAAGGTGTGGGAGCCGGGAGTTGTTCTGGTTGATTTTGTCTTTTCAATCGTATGGCAGTTACTTGCAGCATCATTAGATGATGAAGGGTTATTAGATCACACTGCTGAGAATAAGCCTAGATGGCTAAGCAGGTCACATGATATGAATATTGATGGACCTGACAGCTTTACTGAAAAGAAAACTGAGCAAGCAGAGGGGTTTCAGAAGAAAAATACAGCAATAGCTATTGAGATCATTGCAGAATTTTTGCAACAGAAAATGACTTCAAGGATCCTTTCCTTAGTTCATTGGAACATGTAAGAACTTAATTCTGATATACTTTATATTTGGTAAACCATCTAAATTTGAGTGACTCTCAACTAAGATTTTAGAACATTTTATTGAAGGAAAGTTATCTCTGTAGGTCATCACATTGGGGGTCTTTCATTCACCAAATGCAGCTGCTTGTATCAAATTCTTCAGTTTTTAGGAATCTGAAACATATTACTGCAGAGAGCCTTTTGCGTTGGACGCAAAATATCCATGAGGTTGTTTCTCATGAGTTAAAAAAAAAATCTAAGATGGAGACTAACTTAGTCACACCTGCTGGTTCTCTGATGCCTTTGGCTGGTCAATCTTATGGGGATAGTTGGTCTTCACTCTGGCTTCCTATTGATCTTATCCTGGAAGATGCTTTGGATGGAGCACAAGTGGCAGCATTTAGTGCTATTGAAATTATTACCGGTATGCTTATTATTTTTTTTAAGTCATAATGTTGGGTAAAATTCCTACACAAAGAAGAATATCAAATTATAATCAGAAAACACTGAGAAAATATACATCCAAGAGATCCAATGTCATTTTTAAGTTGCCTAAATCACCACAAAAAATGATTTTATTGAATCCCACAAATAGTAATGAATCTATTCCTTACATGAACCCAAAACCCAAGATTCCCAAATATCATATCACTGATAAATTTTGTAGCAGGCCAGTGTTTTACCTACCAGCTTTCATATTACAACACACCTCTCTGTCAATTATTTTCCTTAGTGGTGGAGGGCCCAAATCCCCACATGCATGCACAATCTCTCATTAATTGGCCTTGTTATAATCTTTGTCACAAACTTGAAGAACATGTGCTCAAGTCAGATCCCCTATTTTCTCTTTGACTTCATTTTCTTTCTTTCCTATGTTCAACACCTCAAAATGTATTTTGGAACTAATTCCTCTTTGCTAAGACATGATCTGTTTACTTCCACGACCAAACTCCTTTTTTTTAATTTATAAATTTAAGAAACTCTTATGAACTTTTATGTAGGACCAAACCATTAGGGTACAAAAGACTCTTTCTTGTGACCTATAGTGAAAGTGAGAAGCAGACTTGTCCTTGACAGACCCCCTCCTTGACTAACATAGATTTTCTTGAAATATTTTCTTCCTTGATTTCTCCAACGTCCAATCCAAGTTTTCCTTTTATAAGGCTATAGATAAGGAAGGGATCCTAATTTATAGGATCATGCTAATAATTACTAACAATGATCCTAATAAAAATCAAAACTGGAACTCAAGGATTCTGTTACACAAAATCTTATCAAGACAAAAACCATAAACAAAATTCAAAATGAAAAACAATATAAAGAAATCCTAAATATAAAAAATAATCCTTATCAGCCAAAAATTATGCTTTTCCTCTGATAATATTGTTTCTCCACAAATGCATCCACATCAGAAATATTCACCGATAATAATTCCAGCAATTAAACCCATTCCAGGATTATTATTGGTGAATATTTCTGATGTGGATGCAATTGTGGGGAAACCAATTAATTGCTGGTCCTAACATAGTATTGATTAATTAATAGTATTCCCACTTGAAAGATTCTACTTGTATTATTTTTCAACATCAGTTTTCTAGTGTGGAGAGAGATGGTAGACTGTGAAGTATCAAATAGTTTGAACAGCACTTACCCTTGAATATGGAGTTTTGAATTAGGACTTGCTTTTGAACTAAAGCACTTGGCAGAAAAGATTCTTGGCAGGGATGGAACAAAAAAAGATTCTTGGGGGGGAGGGGGGGGGGCAAAAATTTAACAACAATGACTAATTTTTTTCTTCAAAATCATGATATTCTTTGGTTCTTGATTTGATAAAACTCTTTCGTAATTTTTTGGGTAATGAATGTCTTTGTAATCTCCTTTTCAATATAAATAGTCAAAGTATTTGCATATCACTGGATGTAATCCATGATCACTATATTAATAGCTTTCATTTATTCTTGTTGAATTCTTCTACATTTTAATGTCGTTCATCAAGCTTAGAATCCCGTGATGAAATATTTGAAGCTTGTGGCATACAAGTAGGTTGAGATGGATCAAAGTTATTGTTTTCTTTCTTTTTTGAATCTTTCAAGGATTTATTTTTGGGCTTCCTTTAGAAACTAATAATTTCAAGATCTTCATTATGGTTTTACACAATAAAATTAATACAGATCTTACTAAACCTTTTGATTCAAGTCAAAGAGAGAGTAGAAACTAGAAACTGATATTCCCTTGCCTAAAAAACATTAACGCCTTCTAACATCACCTTGTTGGAAAGCTTCCTTAATTGCGGACACCCCAAGCCCACCTTAATTATGTCCTCTTTGGACTGGCTTAATTGCAACCTCAAGGGGTGTGTTTATCCCCACGTTGCCTAGAGATATGGGTTCAATAGAGCTAATAAAACTTTCCTCACTTTTGTAAGTGAGTTTTGTGTGGTGTATTTAGGTCCTAAGCCCAAATTCTAAGACATCTAATTGTAGATTGCTAGGATCATGCCAAACCGCACCACAGTGACAGCCTTTCTTGCAGACAGAAAGTATTAAAAAATGACCAGCAAATGATCTAGAGAAATGGCAAAATACTGAATGAGGAAAAGGTTATTACAGAATCAAAAACTTGCCTTGTAACAAAGAGAATTGCTGCTATTTCTATAAAAAAAAATTGTTGGAAGATGAAGGGAAAGATATGTGAATAGTGATCTCAAGTTTTTCCTTGTGAGGATTATGACATGGAAAATCAATCCAAATCAAAATGACATCTTTTGAGTGAGAGTTGAACTGAATACTTGATTTGAATGACAAACTTTCTTTTATTCTATTTTTTTCAAATCAGTGTTATTTATTTGGAGTTGTAATTGCAGGTATGGTGAAGACTTTACATGTAGTTAATGGCACTATGTGGCACAATACATTTTTGGGTTTATGGGTTGCAGCACTGCGTCTGGTTCAAAGGGTTGGAATTATTCTAACAGAAAAGGTATGGAGCTGTATGAATTCTCCCGGAAATATTGCTTGATGAATTTATTTGTGTATGATCATTAATAAGGAAAGACTATACTACAAGTCGTTTACATTTTACTTTGTGTTGTCACATGGCACTTTTACATTTAATTGATTAACTGGTTCTTTAAAAAATGTCTAGTCACACTGGAAAATTGCATGCAATTGAACAGTGCATCTTCACATAACAACAATTATTATGCAACCTGTTATTTTAGGAGAGGGATTTAAAAGAAGGTCCTATACCTCGCCTTGATACCTGCATGTGTATGTTACTGAGCATTACAACCCTCGTGGTAACTAATATTATAGAAGAAGAGGAAGGTCAACTTATTGAAGAACCTGAACATAGCCCTACAAATCAAGGGAAAAATAAACTGGCTCTGGGGAAGTGTCATGGGGAATTGATTACCAGCTTACAGCTATTGGGTGATTATGAGTCCTTACTGACACCACCTCAACTTGTTCTTGTGGAAGCCAATCAAGCTGCTGCCAAAGCTATTGTGTTCTTATCAGGAAACCCTGTTGGTAGTGGATGCTTCAAATATATGAGCACGAATGACATGCCGATGAAATGTTGTGAGTAAATTCTTATGTTGTGGTTATCTGTGAAGTTTTTTTTTCTTTTTCTGTAGCATTTGTACTTCTAATTGATATGTATCCTTTTTATAATTTTATTGTTATGTTCTAACTGAGGCAGAGCTAGGGATTAACTACGTGGAGTGGACCAAAGTTAAAATAATCCAAGCAATCTATGGAAAGATAGAAAATCTGTTGGGCTGATAAACTGTATATGCATTTTACAAACAAAATCCAGGTCTTAAATTTTGGGAAGTTTCACCCTCCTTACTTCCTCAAACAGAAAACATAGGTTTCCCTGCGAACAAACAGTCAACATTGCATATCTATTCAAAAATTTCTCTCTCATTCTCTCCCACTCATCACAATTATAGTTATCAGAAGTTTTATTAGTGGCACTGACACTTTTACCTTGGTACTGTGGTATTTAATCATTTTTTTATACGGCTGTGGTATTCAATCATATTTTCATAATTTGGCCATAATGCAATGCTCTTTTTACCTCTCTGTTATTTACTTTTCGTATAAGGCCATCTCATAAAAGTCTGTTCATATGTTTGTTCTGAATCCCTATCTTTTATGTCTTTGGTGTGCAGGTGACACTTTTTTTTATTTTTTATTTTTGTGTTCATTTTTTTTTTTACTTGCAGCTGGAAACTTGCGACATCTAATTGTTGAGGCTTGTATTGCAAAAAAATTGCTTGATACATCAGCTTATTTATGGCCTGGCTATGTGAATACATGCAGCAATCAAATTCCTTGTAGCATTTCTAATCATGTGTCTGGCTGGTCATCGTTGATGGAGGGATCACAGCTAACTCCTGCATTGGTTAATGTCTTAGTTGCGACTCCAGCTTCCAGGTATTCTAGTGACTCATCATTTCATAGTTCATATGCTCTGTGTCTCCTTCAATCCCCTCTGAAATTTGTTTTCAGAAGGGCAAGATTTGAGGAAGGTGGTTGATTCAGGCAAATTAGATGGAGGGGTTTTTCAAATATAGAATTTTAGTGTTTCCAAAATGCTGTTTTTACCACATGACAAAGCAGCAATGACATGGGATATCAGCTCCTCTAGTTGTTTTGCCATTTTTTTGTTTTTTTTTTCTTCTTTTGGCGGTGGTGGTTTTGGGTAGAACACTATTCATACTTTGAATTGTTGACTGCATTCATATTCCGTCAGAAAATTCGTGAAAAGCCTTGGCTCCCAACATTTATGCTACTGACGTAGAACACTGTTCATACTTCAGTAGTGTGTGTATTTTAACAATTTTGTCTGTATGTTTTCTTTGATCTATTTTAATCAATAGTTTGGGATTTAACAATTGATTTACCGTATCATTTTTAAGGCAGTTACAGTTAAAGGGAAATTATATTTACTTTTTGGGAATGTGTTCCTTCTCTTAAACTGAATATTAAGATATGAAAATAAGTCCCCACTCACATTTCATTCTTTATTTTTCTTCTGTTTTATCTCTAGATATTGTCAAGACAAATAGCTAGTTTATTTCTGAACAGTTTACCCTACTTCATTTTGTGTGAGCCAACACCATGCAGTTTTATTAATTTTGAGAATGCATTGTTGATCATGTTTAATGTCATTGGTGGTTGCCTACTAGGTATTGCACCGTGTTCTTTTGTAATTCAAAAGGACAGCTCAACACGACCTTCTGTTACTAGGGAGTTGAAGAGGATTACATGTAGGCAATCCTATCTGAATGAGCAGAGAGGCTTTTTTATTTTTTTTCCCGTGACTAGAATCTGAGATTACCTGGTCATAGAACAATAACCTTTCCGATACACCAATGCTCTCTATCATTTTTTCAATTTCAATTTCTTTATAGAATACTAACATAGCATGAAATGTAGTCATCCTCAAAACATCCTTAAAAACACAGTCATTGGCTGGCATGTGAATGTATTTGAAGCTTTAATAATGTAAATTAAATATGTTTCTATTATAGATATATAAAAGAGTCTTCAAAATATAATTTATAAAATATTAATGTGGTAAGTAAATTGCATAAAATGCAATAAATTTGTATATAATACTAAAATAGCATGAAATGTAGTCGTCCTTAATTTTCCTTCTCTTTATGTGTCACTCATAAACAGGCACTCACCGGTAAGCATATGAATGTAATCTATGCTTTAATGTAAATTAAATATGTGGGTATTATAGTAATGCATAAAAGAATGTTTAAAATTATTCATGTGGTAATAAATTTCATAATATATAATAAATATGGATAATGGAAATAATTTATATATATATACTACATAGTATGAACGGTTGCCATAGAATACTTTTCTTTAATACAGTTTGGCCAGGTTAGTTCCTAAGCTATGTAGCCTTTATTGCTCCCATAGTTCCTGGATAGAGGGACTTATTCATCTGGAAAATACATGGAGATTCTGGTTTTTTAGATCAAATATAGATTTATAATTTATATATACTAAATATGCATACACATTTATGGAGTCTTCGTGTAACTGAAAATGGCAGCTATCTACTCAATGATAGTATAGCATTATACCTGAACTATAGTATACAGATGCGTCCTTCATTTCATGAAGCCAATATTAGATGCCCTTTTTTGAAAACTTATAAGTCAACTGAATTTATGCCCATGGATTTTGATGGAAGCTTGGCAGAGATTGAGAAAATATATGAAATTGCAATCAATGGTTCAGATGAAGAAAAGATATCTGCTGCTACCATTCTATGTGGGGCATCTCTGGTACGTGGTTGGAATGTACAGGTACTGTTTTTGGCTTTCCAATTATTTCAAAGACTTCTACTTTTTTTTGAAAAAGTTTTTTTTAGCTTGTTTGGTTGTCTTCTGTCTCTTCTTTAATTTTGTCTCTATTTTTTAAGTTTACATTATCAATTTTGTTTGCAGGAGCATACCGTTCTTTTTATAACAAAATTGCTTTCACCTATAGATCCTCCTAATTACTCTGGGGCTGAAAGCCATTTGACCAGCCAAGCTCCATTTTTAAACGTTCTTCTTATTGGAATCTCATCTATGGACTGTGTTCATATATTCTCCCTACATGGTTTGGTATGTTCTGTTTTTAATCCATGTAGTTTGACTGACATGTGTAATTGCCAACATTTACCCTTTTTCTTTTAAAGTGAAGTTTTTATGAAGCGTTTGTTTTGTAGTCTTGGAAGGCTCTATCATCAATTATGTTGTAGAGAGCTTGTAGATGTTGCATGTACCTTCTTTGATTATATGTTTTAATTTTGCCGTTTACTGCAGGTTCCATTACTTGCACCAGGACTAATGCTAATATGTGAGGTTTTTGGATCATGTGTTCCTGATTCCTCGTGGACACTTGCAAGTGGAGAAAAACTCACTCATTGGGAAGTATTCTGTAATGCGTTTACTCTTTTGCTGAGGTTCTGGCGGTTTGATCATCTACCTATTGAACAAGTGAGGAGCGATGCAACAACTCCACCATTTGGATCACTACCTAGTCCTGAATGCCTTTTATTAGTTCGAAACTGTAAATTGGCATCATTTGGAAGAACAGAAAAGGATCAACAGAGGCTCAAAAGATGGCCAAAGATTTTATGTTTTTCCGTAGAACCTGTATTCATGGATTCCTTTCCAAAATTAAACTTTTGGTATCGGAAACATCAAGAATGCATTGCTTCATTCCGCTCTGGTCTTGTGCCTGGAAGGCCTGTTAATCAAATTGTTGATGCTCTACTCAGCATGATGTTTAAGAAGGTAAGTAACGGTGTCAAACCTTCGACACCTACTACTTCAGGAAGCAGCAATTCATCTGGGAATGCATTGGATGATGCTTTAATGAAACTAAAAGTGCCTGCCTGGGATATCCTCGAAGCTATTCCCTTTGTGCTGGATTCTGCTCTGACATCTTGTGCTTATGGGAAAACCTCTACCCGTGAACTGGCTACAGGTATGCCTAGTTCTCTATTTGTCTGCATAATTTTTAACTTGTTACCTATCATATTGAGTCAGGATTATAATGAGGAACTGAGACTGATGTTTTATAATCTGAGAATGTCACGAAATCTAAGGATGTACTGCACATAGCTCTTCCAAGTGCGCAACCACTTGAAAATGCTGGATTATCATCATCATAAATCCTATGTCCTGTTAAAAGCAGAACGCCATTTCTGGGATTTTACGCATGTCCTATAAACCCCATTTCGGGACTATATTATCGTAGTACTAATATATCACTTGTTGTAAAGTGGCGTTAAATTAAATATATATATATATATATATATATATATATATATATATATATATATATATAAACATTAAAAAATTTAAAACTATAATGTTTTTATAACGTTAATAAAAAGTATTATAAAAGATATAATGTATATGCATATACATTTTATGGAAATAATAATTTATCTATAAATAAATATAGATTAAAGTGTAGAGTAAAAAAGCTGTAATTATCGATTATTTTTACATAAAAGCTTATAGTATAATATTTATTCTCAAATAAAATTCAAAAAGCACGGTAAGAAAGAAAATTGTTGAATTATTTCTTGAAAGTTCCACTCGGACATGCTGGTTTTCGGAAATTTAATTCTGCAAAAAAAAAATCCAAAAGGTTTTTTTAATATCCTTCATGGATTTCAATTCTGAATGAAATGACAATTATCTGAATAATAATTGAAAAAATAAAAAGTGATGTAATCAGCGAAAAATGGTGTACAAAATGCAACCAGCAACTTTAAAATAAAAAATAAGAAAATGCAACCACCATTTTAATCTTAAATGGAATAAAGAGTGATATATCTTGATATTGATATACCCCGGAACAATATTGATAGTGAAAAATATATTTGGGAAATTTGCACTGGGTTGTATTGTAATACTGATTTTTCCAACTTTGTTTGGTACGCTTCTTGCTGTGTAATTTGATTTTCACGTTCTTTTTTTGTTTTTTTTTTCTTTCTCAGGTCTCAAAGATCTAGCCGATTTTCTTCCAGCTTCTTTGGTTACCATTGCAAGCTACTTTTCAGCTGAAGTTACACGGGGCATATGGAAGCCATCTTTTATGAATGGAACTGACTGGCCTAGCCCTGCTGCAAATCTAGCACATATTGAGCAACAAATAAAGAAAATCTTAGCAGCCACAGGTGTCAATGTCCCAAGCCTTGATATAGGTAAATTGGTCTTAGTCTCTTATTTTCTATTCTTTTGGATTTTGTTAAAACATTAAGGAGAATTCAAACATTAAGAAAGCATTTAATCAACATTATTATATTGCAAATACAAAAATAAAAGAAGGTAATGGGAAACATTACCTATTTGCAACAGGAAATCCTTAAAAGACATTCTTACAAAGATCCATAATCTCTCTAGAGAGTTGGTCAGGACGAATTATATTAATCAACTTGCTGTATGGTTGACCAGTGAATATTCTCTCGTGCGTACAGGCGTTTATTTTTATGGTGGATAAAATTCGTCATATTTTTGGATCTTTTTAAGTATTAAGTACCTTCAAATGATATTTAAAACCTATTAAACACTTCCAATTACTCAAGAAAAGTAAGTAATAATTTTTCTTAATACAACGGATGCTTTGTTTTTCAGTAAAAGTTATCTACTTTCATTTCATTATCCAGTGTTCTTGGAGTGCTTGATAGCATTTGCATTAATATTATAGTATGTGGAAACAAAAGGTTCATTATTTTGACAGTATTTTTAATTCCCTGTAGATGGGGATTCTCCAGCTACACTTCCTTTTCCTTTGGCAGCTTTTGTAAGCCTCACATTAACATATAAACTCGACAAGGCCACTGAGCCATTCCTTGCACTGATTGCCCCTGCTATGAATGCCGTTGCTTCTGGTTGTCCCTGGCCCTCCTTGCCCATTGTGACCTCTCTGTGGATCCAGAAGGTGAAGCGTTGGAGTAACTACTTTGTGCTCTCTGCTTCCAGCACTGTTTTCCACCACAACAAGGATGCTATAGCTCAACTACTAAAAAGTTGCTTCACATCCACCCTTGGGTTAGGCTATGGCAGCATTTATAATAATGGCGGTGTCAGTGCCCTCCTTGGTGATGGTTCTGTTTCCCGAATATCCAATGGGATCTCTCCAGTGGCTCCAGGAATTCTTTACATAAGAGTGTACCGGTCTATTGGAGATATCACCTTATTGATTAAAGAAATTGTGCCTATTTTAATGCTTTCAGTTACAGACATTGCAAGTAGTGATTTGATGCCCAAGGGGGTTGTAAGGAAACCAAAGAAAACCAAGTTTGGGGTAAAATATGGCCAAGTTTCTCTTGCCAGATCCATGGCACGTGTCAAGCATGCCGCTCTACTCGGGGCTTCATTGGTTTGGATATCAGGTGGACAAAAGTTGATTCAATATTTGATGAGGGAGACTCTGCCTTCCTGGTTTTTATCAGCTACCATGTTCGAGCAAGATGGTGGAGAATCTGGAGTTATGGTTGCTAAGCTCAAAGGTTATGCACTGGCATTTTTTGTTTTTCTTAGTGCAGCATTTGCCTGGGGTATTGACAATTCGTATTCACCAAAACAACGGGCAGAGGTTGTCGGCTTGCATTTGAAATTTCTTGCAAGCACCCTGAACAGGAACGGAGCAATGTTTTCTCGTTGTACTACTTGGAAAGCCTATGTGTCAGGGTTAGTGAGCTTGATGGTGGGTCAAGCTCCATCATGGGTTCGGGAAGTCGATGCTGATTTGTTGAAGAGACTGAGCTGGGGATTAAGTCAGATGGATGAACACAAGTTGGCTCTTCGCCTTTTGGAAATTGGAGGAATAGGTGTCATGGGTGCAGCAGCTGAAATGATAATTGGATTTGAACATAGATCTTAAATCTATCTGAACTTTCAAACTATTCTGTAGTTAGACTAGAAAGATTGGTCAGATTGACTATCATATCTACCAAAATAGAAGAATAATGCAAACAAGATCTTTGCAATGAATTGGTCTACATGCTTTTTTTTACATTTCAATACACTTCT

>GmMED5-2

GGACGGCGTATTGAAAAAGTAAGACGGGGAGGGGAGGGGGAGTTGCCGAAGCGGACTTTTGAAAATTAGAGAGAGAGAGAGAGAGAGAGAGAGAGAGGGGATCTGTTGTTGGAAGGAGGTGAAGTGATGGGGGTGTGGGATGGAATCATGCAAGTAACTAAGTTGGCGCAGGAGAAGAAGACAGACCCTCTTCTATGGTCCATCCAGGTCAGCTCCGCCCTCAACTCCGGCGGCGTCTCCCTCCCTTCCATCGAGCTGGCCCACCGCCTGGTCTCCCACATCTGCTTCGACAATCACCTTCCCATCACCTGGAAGTTCCTCGAGAAAGCCATGTCCCTCCGCCTCCTCCCTCCCTTCCTTGCCCTCTCCCTCCTCTCCTCCCGCGTCCTCCCTCTTCGCCGCCTCCACCCCTCCGCCTACACTCTCTACATGGACCTCCTCAGCCGCCACGCCTTCTCCCTCCTCATCCACTTCCCCAATTACCCCTCCGTCATGTCCTCCATCCATCATCTTCTTCACTTCTCCCAACTCTATTCCTCCCTCGACCCTCATCCCGGCGTCGTTCTCGTTCTCTTTCTCTTCACTCTCGTCTCTCAGCTTCTCGAAGCCTCCTTGTCCGACGAAGGATTGCTCCAGCACTCCCCCCGCTTCCTCCCCGTCGATCCCGCCGATATCGTCATCGACAACACCGATGCCTTGCGCCGGAAAAACACTGCTATGGCCATTCAGATTATTTCTCGCTTTCTTCACCACAAACTCACTTCCCGGATCCTTGCATTGGTTCAGCGAAACATGTATTCAAACCCACACCTTTCATCTTTTTCGCTTTGCTATAACTTTGAAACATATAACTGAATTTCCCTTGTCTGGTTACTCATGATTCATTGGCTGCGTTTAGTATACAGGACAAGACTCATATATTGTTCCTTCCTTACGTGTTCTTCTATCTTAGGAATTTAAGTTTTTAGGTTTTCTCTCATTAGGATGTTCACATTTTTAGTTGGTGGATGGATGTTTATTTGTAGGCCAGCGCATTGGGGACCTTTTCTTCACCAGCTGCAGCGGCTTGCAGCAAACTCCACCTTGTTGAGGAGCTTGAAGCACGTCACTCCTGAGTCCCTCTTGCCTTTGGATTTCAACTCCCCTACTGGGATTAAGCTTCTGTGTTCTGAGTGGAAAACAACACCTACACTGGAACTCAATGCTGTCATGGCCGACTCTTGTGCTGTTCAGTCTCGTCACGATAGTTGGTCTTTGCTTTGGCTTCCTATTGATCTTATCCTTGAGGATGCAATGGATGGCAATCATGTTGCAGAAGCTAGTGCTGTTGAAGCACTTACTGGTATGCAGAAATTTCACTGTTGGAACTGTTACTGAACTTGACTGCATCTAACATTCATAAGACTTTTTGGTTTTTGATGCATGATTGAATTGCTTCGTTGAAGTGGTTGTTGGCTTATTGCAGGGTTGGTTAAGGCTTTGCAAGCTGTTAATGGTACTGCATGGCACAGTGCCTTTTTAGGTTTATGGATTGCAGCACTACGGCTAGTTCAAAGGGTAATATATTGCAGCATATCTATTCTATTTTTATTATGTTTTTAGGTTTACATTTAACTGATTTACTGGTTCTCCACTAAATCCAACCAGTGACTCTAATTTGGATTCTGTTAACTTGTTTTAAGTACATTTTCTCCCCCTTTTTTTAGGAGAGGGATCCAGGAGAGGGGCCTGTACCTCGCCTTGATACCTGCTTGTCTATGTTATTGAGCATTACAACCCTTGTAGTTGCTAACCTTATTGAAGAAGAGGAAGGCGAACTTATTGAAGAAGCTGAACATAGCCCTGCAAATCAAAGGATGGACAAACAGGCTTTGGGAGAACGTCATGGGGAATTGGTTACCAGCTTACAGCTATTGGGTGATTATGAGAATTTACTCACTCCTCCTCAATCTGTTATTTGGGGAGCCAATCAGGCTGCTGCCAAGGCTACCTTGTTTGTATCAGGACATAGTGGATACTTGGAACATACGAATGTGAATGACTTGCCCACAAACTGTTGTAAGTAATTGCTTCTATAATATGAATCTCTAATGGTGTTTGGTTGTAGAAAGAACTCTAAGTTGGAGAGGTCCTAAAGTAAAAATAAATGGACAGTTTTAAGAACAATTAAAAACACTTGTGTCCAATTGTATCTTCTTCATACAGTTATAACTAAAATATACAGCAATTTCATGATAAAAAAATTGACAATATGGTCCACAATATGAGCCTGCTTTCATTTATCCTCATAATTGTTCCTTAATGAATTTCAGGGGCATCCAACTTGACCCTAAGATTGGTCATATGGGATTTAATCATATTTTTGTTGTCAATGATCTATTTTTCTGTTTTCTTCTGTTTATTTTGCTCACAGATGTGGTGGTCTCATAAGGGTATTTTCATGTTTATCTCGGGCTCCTGATTAAACCTCTTTGGTTTGTGAGTGACAGTTCATCATCTTGTGTTTTCTTGCAGCTGGTAACTTAAGGCATCTAATTGTTGAGGCTTGTATTGCAAGACATCTTCTTGATACCTCAGCTTATTTCTGGCCTGGTTATGTGAGCGCACCTTTCAATCAGCTGCCTCATAGTATTCCTAACCATTTACCTAGCTGGTCATCATTGATGAAGGGATCACCACTAACTCCTCCATTGGTTAATGTCTTAGTTGCAACTCCTGCTTCTAGGTATGCTGCTATGTCATGTATCATTTTCTGCTCTCATACATTACATGTGTTCTGTGAGAGTTGCTATTTCCACATGGCAAATCAGCGATGGCAAAACTAAACAATTATTATCTTGTCTAGTTTTTCCATTTCACTTTTTCGTTTCTGAGTCAGTTTCACTGTAATAATCTAATTCCTACTACAGTATTTATCCTGCTTCAATTTATATTACTCAACATCATTTAGTGCTGTACTTTTGGGGAAATCTTTGTTGTTTCTTGTCTAATGTCCATAGTTGTGGTTGTCCTTTTTTTGCAGTTCTGTCTACCTCCCTCACTCCCCCTGAAGGAAAAACAATAATTTTATAGTTATAGTTGTTAGAAAATTGCATTATCATGAAATTTAATAATTTATTCATCCTGAACTGAAGTGGGGTTGAGATGGTTGTTTATTTGGGTATAGGTCCTAATTTCAAGTTGTGGTAACAATTAACAACACGTTATCTGTTATCTCGCTATTGTGTTTTTGGGGTCGGCTGCTACACGCTGCTATCCAGGATTTAAAATATTGGATTGTATGGTTTCCTTTTCTTTTTTTGTTTTTGAAATAGTTGAGTCTTCTCTTTAATAAATAAATATTTTTTTTTATAAAAATAGTTGAATCTTTAAAATATATTATGTTATTAGAAAAATGATAATAAGTGAATGTGATATGCAAAATACAGTTTATGGGTTAATTACTCCCATCCCATGTATCTTGGATAAAGAGGCTTATTTTCATTTAAAGAAAATAGAAAATTACTTGTTTCCAAGTCAAAGTAGAAATATTCGTAGATATTAAATAGAGACACACCGTCAAAGAAATGACTGTGTTTGAAAGTGGTTGCTGTCTAATTGGCTGTAGTATTGCTTAATACCTGGGCTTCAGTTACACCTTTCTGTTTCATGAAGTCAGGTATATCCGATGCTCTATTTTGTATATTTACTAGTGGTAATGCAACTGAATTTAAGCTCATGGATGTAATGGAAGCTTAGCAGAGATTGAGAAAGTATTTGAATTTGCAATCAAAGGCTCGGATGAAGAAAAGATATCTGCTGCTACCATTCTTTGTGGGGCATCTTTAGTACGTGGTTGGAATGTGCAGGTGATCCCAACACCATTTTAATTTGATTTTCTCCATAATTAGAAAATCATACTAGACTACACTTCTTCTTTGTTTTTTTTTAAATAATTTATTTTGTTTGCTTCCCCTTCTTTTTCCTTTTTTTTTTTCTTTCATGATTATAATGATTATCATTGTTTGCAGGAACACATAGTATTTTTCATCATAAAGATGCTTTCACCTCCAGTTCCTCCTAAATATTCTGGGACAGAAAGCTATTTGATTAGCCATGCTCCTTTTTTGAATGTCTTTCTGGTGGGAATTTCATCTGTTGACAGTGTTCAGATATTCTCCCTACATGGCGTGGTATGTATAGCAAGTGGTTTACAATCTTTTTTACTCAAAAGAAAAGGGAAAACTAATATGAAGAAGAAAATGTTTTGGAGAACATCCCTCCCCAATACACACAGACAAGAAAAATTATGTCTTATTCAATTTTAAGGTACTATCAATCAAAACCAATTTTAATATTTTCTTTTCAGAGAAGGGAATGGTTGATAACTAAACCTCACAAAACTGGGTTGTAAGATGAGGACTGGCGAATCATTTTAAGATATGGGATTTCAACACCCCCTCTCATGCAGGATTAGACATCTAGAGCATGGACAAATGTGGGTGGCCCAATGACAACATCCTCTCACACCTCGGAATAGACATTTTGAATGGAGACAAGTATGGGTGGCCCAATGACAGGTCTAGGATAGGTTCTGATATCATCATATAACATGGGATTATGGCAATCTAAGTCAAACCCCACAAAACTAGCTTGTAAAGTGAGGATTGTCCAAGTTTTATAAAAACCACATTGATCATATCTCTAGTTGATGCACACCCCTTCATAAACACTAAGACTGGACATCTAAAGCTTGGGAAAATGTTGGTGGCCCTATAACAACATCCCAACACTTAGGAATAGACAACTAGAGTGTAGATAAATATGGGTGACCCAATAACAAATCTAGAATATGCTCCGATATTGTTTTAGAATTTAAGATTAGGGCCTAACTCAAGCCCATAAAACCGTCTTGTATGGTGAAGACTGCCCAATCCTTATGAGAACTACATTGGTCATATCTCTGGTCAACGTTGGACCTCAATAATAACTATTCCTGCTTTTAGTTTGTTGGCTGATTCTATGCATTTACTTTCTTTCAGGTTCCACTACTTGCAGCTGTCTTAATGCCAATATGTGAAGCTTTTGGATCATCTGTTCCTAATGTCTCATGGACTGCTGTAACCGGTGAAAAACTCACTTGTCATGCAGTGTTCTCTAATGCATTTATTCTCCTGCTGAGATTATGGCGGTTTGATCGTCCACCTGTTGAACATGTGATGGGGGGTGCAGCAACCCCAGCATTAGGATCACAATTAGGTCCTGAGTACCTTTTATTGGTTCGGAATTGTATGTTAGCATCCTATGGGAAATCTCCGAGAGATCGAGTAAGAAGTAGAAGATTTTCAAAAATGATAAGTTTTTCTCTCGAGCCCTTATTTATGGATTCCTTTCCAAAATTGAACATTTGGTATCGGCAACATCAAGAATGTATTGCTTCCACCTGCAATACTCTTGCACCTGGAGGGCCAGTTTCTCAGATCGTTGAAGCACTACTTAGCATGATGTGCAAGAAAATAAATAGAAGTGCTCAGTCATTGACGCCTACAACTTCAGGAAGCAGCAATTCATCTCTTTCTTCATTGGATGATGCTTTGATGAAACTCAAAGTGCCTGCATGGGACATCCTTGAAGCAACTCCATTTGTTCTTGATGCTGCTCTCACTGCTTGTGCTCACGGAAGACTCTCTCCTCGTGAATTGGCTACAGGTTTGCCCAGTTTACTCATGGATATATTCAAGCCTTTTTTAAATTGATTTAATCATTATGTATTATTGAGAACTATTTCAGGATTATACTAAGGCTTTCATGTTTTAAAATGTGAGAATGGCAGAGCTGTGGAATAAAGATGTCCTGTGAAAAGATGAAAGAAAATTGAACATGACCTGTAATTCTTGGAATAACTTGGCAAGTGAAGTGACTATATATATAACAAAAATGATATTATTATACCAAAATCTCATGTAATTGTCTTCTCATTCACAGATTCACTTGATTATCCTTGTTCATTTGTCAGTGAGATAAATACAGTATTTGCCAGGAGTTAGGAAGATAAAGATAGTTAGGACAGTTATTGTAATTAGTTAGTTAGAGGTGATTTGCAAGGTTAATGAGGAGAGGAGGATGTAAGAGAAGAGGAATTCTATCTTGCATGTCTAACTAAAATTGGGGAGGATGTAAGAAAATAGGTATTCTATCTTGATAGGTAGGCATATAGAAGAGATACTCCAAAAGATAATATTGCATGTCTAACTAATGATTGTATTGTTTGGTTCCCAGAGGAAAAATAGTAAATTCCAATTCCAATACTAGACACTTAGGTGGGATCTTGTCACTCCCAGTCATTTTTATTTTTAGCTCACATCAAATTATGAATCATCATTATCATCACCTTCGTCCTCCTCAATTTTAGTTTTCAAGCTCCTTAAGCTTCCTCTCTTCTTTGTTTGGACTGGACACCGCTTTGTTGCAATAGTTTTTATGCCATGGACAAGGACAATTGGTTCAAGATCTTTGTTTTAGGACTTACATTCTCAAATAAGTAGTGTTTGACCTAGCTTTTTTTAGAGATTCTCTTTTCCTTAAAAGGAGAAGTTGATGTAAACATAATTTTAGAGTTTAAAAATAAAAGGAGCATTTTTTTAATGAAAATAAATAAATAAAAGATGGAGCTTTCACATGAGTTTCTGCTTCTCTATTTTGAAAACATACCTCCCTTGTCCATGGCCTGATTGACAACCTCCATGTTTGTCACAAAGCTCCTCTTTGGTGATCTCTTGTTCAACCACCTGCTCTCGCCCAATTTCTATTCCTTCAAAGTTGTGATCTGCTACTATTTCCTCATCAATATGCATGCCCTTGTTTCATTCTATTACCTCATGAGGCTCATTCTTAGTCTCGGCCACGATCCTATCATCCTCAAAGCTTGCTCTTAGTTAAGTGATGTTTTCCTGGGTATCAAGCTCCATATGCATTCTCAAAACCATGCCTCCTGAATCCCAATAGCTTTGTGCTCACTAATCTTGATGATGTGTACTCCAAGTATTGACATGTTTGTAGTGCATGCCAATTGTTTGACAATCACTAGTAATGTAGAAGTTTGCAATTAAGTTAAAAAACCATAAAATACCAAACAACATTAGAATTTTTTTTTAACTTCAACTTAGTAGTAGTATCTTCAAAGATAAAAGCTAGGCCAAACGCTAGGTAAGTTCATGCTCCTTTAAGTTGGCATATATTAACTTATTTGATTAAATGTATGTTAATATAAAAATTGTATGTTTGGGAATTCATTAAACTGGGTTGCCAATGTCTTAACCTTTAAACTTTTCTAAGGCTGTTTGCTTAGGAAGTTATCTGCTTGATTTTATCTTAGGTCTCAAAGATCTTGCTGATTTTCTTCCGGCAACTTTGGGTACCATTGTAAGCTACTTATCATCTGAAGTAACACGTGGCATATGGAAGCCTGCTTTTATGAATGGAACTGATTGGCCAAGCCCTGCTGCAAATTTATCTATTGTTGAGCAACAAATCAAGAAAATTCTAGCGGCCACAGGTGTTGATGTCCCTAGCCTTGCTATAGGTTTGTTAAGTTTACTTTCTTTATTTGTGGAAATATTAATTAAACAGAAATCCTTAGCTAAATCCCTCTTTGCACGTTCATTTTATCCTTTCCTGGGTGATTTTTGCAGCATTGTCATTTTCTCCCTATAGACCAACTAAGTTGTCATGTTTTGCATATTCATATAAATTTTTGGGAGTAATTTGTGGGTGATGAATAAATTCTCAATTAGCTACCTGCTATATGTTGACAGAGAAATATCAACTCACTGTCTTGAGTTTTTGGCAAACCGTGTTTTCATTTTCTATTTATATGGACATCACACATCAGGTTTATTAAATCAATCGGTTGACACTACATTTTTAACTGCTGTAAACAGATGGGAATGCTCCAGCTACACTTCCTTTGCCATTGGCGGCTTTTCTGAGTCTCACAATAACATATAAACTGGATAAATCTTGTGAGCGCTTCGTTGTTTTGGCTGGCCCATCTTTGATTGCCCTTTCTTCTGGTTGCCCCTGGCCATGCATGCCCATTGTAGGTGCTTTGTGGGCTCAGAAGGTGAAGCGCTGGAGTGACTTTTTTGTATTCTCTGCTTCTGCAACTGTATTCCACCATAGCAGGGATGCCGTAGTTCAGCTCTTAAGAAGTTGCTTCGCCTCCACCCTTGGGCTTGGTTCCGCCTGCATTTATAACAATGGTGGTGTTGGCACCCTCCTTGGTCATGGCTTTGGTTCTCACTACTCCGGAGGGTTCACTCCGGTTGCTCCTGGGTTTCTCTACTTAAGAGTTTACAGGTCTATTAGAGATGTGATGTTCTTGACAGATGAAATTGTATCTCTTTTAATGCTTTCAGTTAGAGATATAGCAAATGGTGGGTTGCCCAAGGGTGAAGTGGAGAAGCTAAAGAAGACTAAGTATGGAATGAGATATGGACAGGTTTCTCTTTCTGGATCGATGACACGTGTTAAGCATGCCGCTCTTCTTGGGGCTTCGTTTCTTTGGATATCTGGTGGTTCAGGTTTGGTTCAATCTTTGATTACAGAAACTCTGCCTTCATGGTTTTTATCAGCCCAAGGGTTAGAGCAGGAAGGGGGAGAATCTGGAGTTGTGGTTGCTATGCTGAGAGGTTATGCACTTGCATGTTTTGCGGTACTTGGTGGGACATTTGCCTGGGGTATCGACTCTTCGTCACCAGCATCAAAACGACGGCCAAAGGTCCTTGAGATTCATTTAGAATTTCTTGCAAATGCACTAGATGGAAAAATATCTCTTCGCTGTGATTGTGCTACTTGGCGTGCGTATGTGTCTGGGGTTATGAGCTTGATGGTGAGTTGCACACCACTGTGGATCCAGGAACTTGATGTGGGTATATTGAAGAGAATGAGCAACGGATTAAGACAGTTGAACGAAGAAGACTTGGCTCTGCACCTATTAGAAATTAGAGGGACAAGCGTCATGGGTGAGGTAGCTGAAATGATCTGCCAAACTAGATTATAACCGGGCAGAACTTGGGAGCATGGTGCATATGTGATGATACCAAGACCTTATGGAAAATGACACTTGTTTTGCTAGCTACCTTCTACCATTTCTGTACATAGCAATGTAAATTTCATAATTTTGTATTACCCCCATTGAATTTAGATCAGTTACGTTTAGGTAATAGTATAAACCTTTGATCATAATAAGTATTATAGCAGATATTGTCTTCATGTGTACTTGTAGCGTTAACAGATTTCCTAAACTGTGAGCAGTTGGTTATACATTTAGCAATAGTACTAGTATGTTTTCGGGT

>GmMED5-3

AGATCTTACAACCAAACACTATAGCGATTCCTTCAAAACATTAGGCAATTTATTTGAGTAGGATTACAATTCACAATAAGCGTTAAAACTCTTTTTCTAAATATCAAATGCAACTATATATTTACAATACAAACTAAAAATTACTATCAATTAAGTTGAAACAAGCACAAGTAATAACCAAAGATATAGTAGTGTTAGTGTGTCAACCTAGGTGGAGGCCTCAAAGCGACCACGCAGTGGCGCAAAACTGAAGGGTATTGAGTTGTTTAGTGAGTGGGCGTTTACTTTTTTGTCACGATCCCCGAGGAACTAAATACCGGAAGCCGAAATTCCCGAAACACCCCTCCCTGGTTTGACTTAAACGACATTATGCCGATGCATAGATACACATTGCAGGAAGGGTAACGAATCTGAAGTCGAAATTATAAGTTTCTTTGGATGTTGGGATTGTGGGGGATGGAGTGTGTGGCGGAGATGACGAAGGTGGCGCAGCAGAAGGGGAGTGATCCTCTGCTTTGGGCCGTTCAGATGTATTCCAATTTGAACTCCGCCGGAGAGTCTCTACCCTCCCTCCAACTCGCCGAGTTTTTGGTCTCTTACATTTGCTGGGATAACAATGTCCCTATTCTATGGAAGTTTCTCGAAAAGGCATTGACGCTTCAGATCGTTCCTCCTATGCTTCTCCTCGCTCTCCTTTCTGTCAGGTCCCTTTTTCCTTTCTCAACTCTCTTCTCTTCGTATTTCAATTCTTAAGGCTTCTTCCTTTTCTTACACTTTTTCTATGTTTTCTCTTCATGGACCTCTTTCTTCTTCATCTTGTTTTGTTTACTTTATTCCGTTGCGAAACCAACCTCTTTTCACGTTTGGTCCTATCGCCATAACTTTTTTTTTTTTTTTTTTGCTTTTATGAGCTCTGCAGTGCCTTTTGTTTTTTTTCCAAAGATTTTAACATGGCAAGTCCATAAGTGTTGAGGAATGAGGAGTGAGGTTTTTGTAGTTATTTAGCACTCATTTGTCCTTGCAATATCGGATCGGACTTGGGGTGTTACGAAATAATTATGATTCTGTAAATGGATGTAATTATCAATCTCCTCCGGTTTCTATTAACGCCTTGAAAATCATGGATGGTACAATTTTTTGTCATATGAACGGACTTTTGATTTGTTATACGCTACTTAAAAGTGATGTTTTTCTTCTTCAAGTTAATTCTGCATACAAGGAAAATCTAGCCACTTTTTTTTTCTTCACTTTTTCCATACTATGACTTTCGAGTCATAACCAACTCGCATCTATTTATTTAATGATTTTTGCAGTGTAATTTCTCTTAACAAAAACTACCAGACCTCGCTCCTGCTACATGGGTTTCAGTACACTATTGTCTGTGTCATAGACTCATGTTGTTCCATGCACCCTAAGTACTTCTTAATAGTCTGCCATCTTGTCGGTTTTTGTCTCCCTTTTTTCTCTTTACTGGAGCATTATCTGAATTTTTTTGTGTAGGTCCTCTTCTTTCATGACCAAACTACCTTAAATGGTTTTCCATAATTTTTTCTTTTCTTTTTAGAACCCACTTCAACCTTCTCCCTGATAATCACATCTTAGTCTTATCCATCCAGATATGCCCAGTAATCAATTGCAACATCCTCATTTTTGCAACCTACACCTTTTGCTCATGATTCTGTTCACTGCTCAGCATTTTGATCCAGAGCTTTAGGTTGTTTAATTGTACTACATCATGAACAATAGACCATGATTTTTTATAGTTTTATTTTTTTATTCACTATTACAGTTTGGTGTTGATAAAGACATGTTTTTTCTTCACAATTTGGAACAAAATCTATATGCTTGTGTTCCCTGATGAATTGCTATTGTAATCTGGTTTGCCATGGTATTCTTCCCTGATGTATTGCTGTTTTGGTGTGCTAATGTATCATGTATCTTTATTCTTTTTGGCAGGGTTATTCCATGTAGACATGTCCAACCTGCAGCGTATAGGCTATATTTGGAACTTGTCAAAAGACATGCTTTTGAACTTAAATCTCAGATAAACAGGCCAGATTATCAAAAGTAAGCTTTAATTGATGTAATGTAACACTTCTGCTTCAATAAATTTTTGCTTCATCTTGTTTATACCTTTCAAGTTACGTATGGTTTTAACATCTCTATCACGTTCTTTTCTTTGACAATTTATATGATTTTCTCTAGTACATTAAGGATTGATTACTGAAAACTGTTCTGTAATTGAATATGCTACTACACTGTATCACTATCACATTAGATCCTGAGTTTATGTTGATTAAAATTATCAGCATTTGTACTTCCCTAATTTCTTTATGGTAAAAATGAATGGTTGAGATACATATGAACTGTTAATTTTAATAGTTTATTGTGTTAGTGGTGTGCTTTGGTTGGTCATTTGTTAATTCTGATGCTTATACTTGGACAGGGTCATGAAATCTATAGATGCTGTTCTTCATCTTTCCAACATATTTGGCATGCCACAAAGTGAGCCTGGCATTCTGGTGGTTGAATTTATTTTTTCAATTGTCTGGCAGTTGCTTGATGCATCATTGGATGATGAAGGTTTACTGGAATTTACTCCTGACAAAAAGTCTAGATGGGCTACACTATATCATGACATGGAACTGGATAGGCATGATAATTATAGTGAACAAAGGACTGAACATCATGAGAAATTGCAAAATGCAAATACACTTATGGCTGTTGAGATGATTGGGCAGTTTTTGCAGGATAAAATATCTTCAAGGCTTCTTTACTTGGCTCGCCAAAATTTGTAAGTATATTGCTATGTTTTTATTTGTTCTCCACATTTGCTGTCAACTTTTACTAATAACAATGTCAATCAGAATATATCTGTTCATTCCAAAATTTCCCATAAATTTGGTTTGGCATATCATTTCCTATTTTTTTTATTTCATTTCATTTTTTGGTTTAATAAATGATTTCAAAGTATTCATGAAATTATGTATTCTATAATTATAGTGGGAGTGATTTCATGTGTTTTTTAATGATATGTCAGCCTATCACATCTGATGGGATAAGGCTTGGTTATTGTTGTTGTATTTGCTCAGCATCTTTCTACTGTTTTATCAGGCCTGCACATTGGCTTAGTTTTACTCAGAGGCTGCAGCTGCTGGGTGCAAATTCATTAGCATTGAGAAAATCAAGAACTCTAAGTCCCGAGGCACTTCTGCAATTGACTTCAGATTCTTGTATGGTCTTGTCTCGGGAATGCAAAACAAATTCACAGAAAAAGTTCCAAACAGTTATGTCTTCTGAATATCTTTCTTCTTCTGCCTCACTATGTCATGGTGCAAGTCATTCTGCTCTCTGGATTCCTCTTGATCTGGTGCTAGAGGATTCCATGGATGGCTATCAAGTTAGTGCAACAAGTGCCATTGAAACAATTAGCGGTAAGACTGAGAATAGTTATTGGAATCTGACTGAAATGTAGATATTGTCTTTTCAACTTTAATTTAGGTACCTGTCACATGAAATTTTTTGTGTAGGTTTGATAAAGACCCTGCGAGCAATAAATGGCACCTCTTGGCATGACACATTTTTAGGCCTTTGGTTGGCAACTCTTCGTCTTGTTCAAAGGGTATAACTCTTTCTCTGCAACTCAATCTGTTTTCCTGTGAAGTTGTGGTTTTAAATTTATCTTGTCATCGATTGCTTCTGGTATTCCATTACCTTACACTTTGTAATTAAAGAATTTGCATAAGTGGTTTACTTCTCCAATTTACTTGTTGATTTGTTTATAGGAAAGAGATCCTATTGATGGCCCAATGCCTCATCTTGAAACTCGCTTATGCATGCTCTTGTGTATCATACCTCTTGTTGTTGGCGATCTTATTGAGGAGGAGGAGGAAAGGACACCGGTTGATGAAAAAGACAATGGTCCTACAGGTTTCTGGAAAGAGAAAAAGGTTGCAGGAAAATGTCGTGATGATTTAGTCTCAAGTCTCCAGGTATTGGGTGATTATCAAAGCTTGCTCACTCCACCTCAACCTGTTCTTGCTGCTGCAAATCAAGCTGCTGCAAAAGCAATGCTATTTGTTTCTGGCATCACAATAGGGAGTGCATATTTTGACTGTCTCAACATGACAGAGATGCCAGTTGACTGTTGTAAGTTAGAACAACTTTTCTAAATTTCAGCAGTGCATTATTGAGGATGTTGTTTGCATTTTCTGATTCTCTGTGTGATTTTCTTCCAAATTTGGAGAAGTATATGCCTCATATTTGATATTTCACATGCTGTAATGAAGTGTAACAACCAAAGATGTGTATGATAATGTTTCTGATAGCTGCAAAAGTAATCTGTTATCTAATTTACATAAATCTACACTTTAAAAATTATACTGATTATAAAGCAGCTGGAAAGTATAAAAGTAGAAATGATCTTATTTTGCTAAATATTGTAATAAGCAAAATCATTACATTGTAGAATTATGTAAAATCATGACCCGCTCTCTAAGGGTTGTCCTGTTCCTGTAATCCATCGAATTTTTGGTTTAGCAATTCACTTTTCCTGTAATTGTTGATTCTGTATTTGATATTTGGTGGTTAATTTGGAAAGAACATATATTCTTCACATGGTGCTTCATTCAGAAGCTAAATATTTTTGCAAAGATAAAAATAAAAATTCTTGGTAAAAGTATTTCACATTTAGGAACTTCTTGGCGTCCTACTGATAACAAAATTCAAAGAGCAGTTAATGTGTAACCACATATTGACCTAATTAACCATTTGAGTAGTTACATCTTCCAAAATTGTAATAATTTTACCAAGAATTTCTTGTTTGTAATTTTCTGTATATGTGTGTGTAGAAAACTAAATTAATTAAACTATTTATTCTTCTTCAGCTGGAAACATGCGTCATCTGATAGTTGAGGCTTGTATTGCCCGCAATCTACTGGACACATCCGCATACCTATGGCCAGGTTATGTAAACGGACGTATCAATCAAATACCTCAGTGTATGCCAGCTCAAGTACCTGGCTGGTCATCATTTATGAAGGGAACCCCACTTACTTCAGTGATGGTTAATGCTTTGGTTTCAAGTCCTGCTACAAGGTATGATCTAATTTTGGCTTATTCAGTCAATTGGTTCGCATACATTTGAAAACCTGCAATGTTCTAATTTTTGGCAAAGGTGTTCCAGTATAGTATTTATTGTGCTCCCTCTTTGTAGGCGCATAGCCACAATGCTATGACCTTTACCAAACTATTTTTTTGGAAGTTTTTATATTTTAAACAAAGTCCTAAATAATGCTGGCAGTTGAAAATTATAAGCTTTTTCATGTGGCCAACCTCTACGAATTTGTTTTGGGGATATCTATCATACGGGGTGAAGTGAAGGTTTTGTTTCCAATCTCACTTTTAATGCATCCATCACTGTATGGACAAATTAAAATGCTGAAACATGAGATCATATACTAAATGCAACAATGATTCATCATAAACTGAATTTTATAACATAACAAGGGTGACTAAAAGTGGGTAGAATTTAAATTCGTAAAATTTTATTGCCTGGTCTTCAGTTTTTCTTTTTCTTTCCTGAAGCCAGGTATGTCAGATGCTCTTTTTTGTAAATTTACCAAAAGGTAAAGCAACTGAATTTACGCTCCTGGATTTGATGGAAGCTTAGCAGAACTGGAGAAGATTTTTGAAATTGCAATTGAGGGATCAGAAGATGAAAAGATATCTGCTGCTGCCATTCTTTGCGGGGCATCCCTAATTTGTGGATGGAATATACAGGTAGTGAAGAGTTAGACAGTAGAGCATATAAAAACTATCATTTGTTCAGTTGTGTAAACTGCAGAGCACATCCTTCTTTCTAACCACCTTCCCATCCTAATAGAATTTGGATTTCTGTTTAATGCAGGAACACACTGTACATTTCATTCTTAGGTTGTTATCTCCACCAGTTCCTGCAGAAAACACTGAAGGAAACAACTATTTGATTAATTATGCTCCAATACTGAATGTACTCTTTGTTGGAATTGCATCTGTTGATTGTGTGCAAATATTTTCACTCCATGGCTTGGTAAGTTTAATTCAATCCCTAATTTAAAATGTTATTTTCGCTACTACAACAGAAGAAAACCTTTGCAAACACACCAGAATAATAAAGTTAAAAACTTCTGCAGAAGATAAGATGGCCTAGAGGACTGTCATACACAAACCCTAGTAGGCTAGTACCACTCTCAACCTTTCCACTAATTACTTTAATTTTTTTCAGCTTCCTTCAATTGTCTCCTTTTTTTATTCCCATCTCTGACCCTTGGCGCAACGGTACAATTGTTCCTTAGTGATCGGTTGATCATGGGTTCAAATCTGGAAATAGCCTCTTTGCATAGGCAAGGGGAAGGGCTGCGTACAATGACCCTCCCCTATACCTTTGCGAAGTGAGGAGCCTCTGACACTGGGGTACATTAGGTTTTTTTTCTCTCCCTTCCCTCTCTCCATTCATCCCCAAGTCTTCTCCTCATTCACACCCAAATATTTAGTCCCTTTACTTTAGTCTCATGCCAGAGCTTTGTAATTGGTTACTCCCTAATCTTCTCCTCCAACTGCATCTGGCCTATAACTAAACATCCCAGCATGCTTAGCTCCCATTTTCCTACTATAAACTTGGTTAAAAGGCAGCTATTTAAGAAACATTCAGTAGACAAATTTATTGTGTATGTCTTATATAGATACACGGGCTTGGATTATTATTATTATTATTATTTGAATTAATGCATTTCCCTATTCTCCAACCAGGTTCCACAGCTTGCATGTTCATTGATGCCAATCTGTGAAGTTTTTGGGTCATGTGTACCGAATATCTCATGGACACTAACATCAGGGGAAGAAATATCTGCCCATGCTGTGTTTTCAAATGCATTTATTCTTCTTTTAAAGCTATGGAGATTTAACCGGCCTCCACTTGAATATGGAATTGGAGATGTACCCACAGTTGGTTCTCAATTAACTCCTGAATACTTACTATTAGTACGTAATTCCCACTTAATGTCAGCTGGTAACATCCACAAAGATCGCAACAGGAGGAGACTCTCAGAAATAGCAAGTTTATCATCGCCTAATTCTGTATTTGTTGACTCCTTTCCAAAATTAAAAGTGTGGTATCGGCAGCATCAAGCATGTATAGCTTCAACACTCTCTGGTCTTGTCCATGGAACCCCATTTCATCAGATTGTTGAGGGGCTTCTTAACATGATGTTCAGAAAGATTAATAGAGGAAGCCAGACTACTATTACATCTGGAAGCAGTAGCTCTTCTGGACCTGCAAATGAAGATGCCTCTATAGGTCCAAAGTTGCCTGCTTGGGATATTCTTGAAGCCATTCCCTTTGTGGTTGATGCTGCTCTTACAGCCTGTGCTCATGGAAGACTGTCTCCACGTGAGTTGGCAACAGGTATGTGTGTTTTGATCAGATAAAATCCAATGAAACACACCAAATAGCTAGTAGAACTCTTGATCTCTAAATTACCGAAGAGAATTCAGTTTCAAAATTCTTGTTGATGTATTTTACCTCTAAACATAGAGCAAGAGCAACCAAATTCATGTTCATGTAGAAGTTCCTTGATGTACACCACAATATCATGATACCAAAATGCCTTTCTTTAATATCAGGGCTTAAAGATTTAGCTGATTTTCTTCCTGCATCTCTGGCAACCATAATAAGTTACTTCTCTGCTGAAGTAACTAGAGGAGTTTGGAAACCTGTATTTATGAATGGAACAGATTGGCCTAGCCCCGCTGCGAATCTACTGAATGTTGAGGGACAGATCAGGAAAATTTTGGCTGCGACTGGTGTAGATGTGCCTAGCCTTGCTTCAGGTTTTCCCATTTTTATGACCCTAAATTTTATATACTTTCTAAAATCCTGACTTATAATGCATTTTCAGGAGGGAAAATTACGTTTACAACTCGTGATTCATTACAAATTTTTGTATTAAATTTATCTTTTACACAGCTTGAATGAGTATTCAAACGATTTCCATTTAGGAAATGTCTTCCTCCATCTTCCTATCTCTTTCAACATTATTATCAAGTTGTTATTTAGTATTGTGACCAATGGTCATCATTTTAACAGGAGACAGCTGTCCCGCAACACTTCCATTGCCCTTGGCTGCCTTCACAAGCCTTACCATAACCTACAAAGTTGATAAGGCATCAGAGCGTTTTCTTAATTTGGCTGGCCAAACATTGGAGAGCCTCGCTGCAGGTTGTCCATGGCCTTGCATGCCAATTGTAGCTTCCTTGTGGACCCTAAAGGCAAAACGTTGGAGTGACTTCCTTATCTTCTCAGCATCTAGAACTGTCTTCCTCCACAACAGTGATGCAGCAGTTCAACTTCTAAAGAGCTGCTTCACAGCTACCCTTGGCATGAATAGCTCTCCTATCTCAAGCAGTGGTGGTGTTGGAGCCCTACTTGGACATGGATTTAAGTACCACTTATGTGGAGGGCTTTGCCCTGTTGCTCCAGGAATTCTCTATTTACGTGCGTATGGGTCAATCAGAGATGTTGTTTTCTTGACGGAAGAAATTGTTTCCATCTTGATGCACTCTGTGAGGGAAATTGTATGCAGTGGCCTGCCAAGGGATCGATTGGAAAAGTTGAAAGCAAACAAAGATGGCATAAAATATGGACAGGTTTCCCTCGCTGCTTCAATGACTCGGGTGAAGCTTGCTGCTGCCCTCGGGGCCTCTTTAGTATGGATATCAGGAGGGTTGATGCTGGTTCAATTATTAATAAAAGAAACTTTGCCATCCTGGTTTATATCTGTTCAGAGATTAGACCAAGAAGAAAAGTCTGGTGGAATGGTTGCAATGCTTGGTGGATATGCACTTGCTTACTTTGCAGTTCTTTGTGGTGCTTTTGCTTGGGGGGTTGATTCATCATCATCAGCTTCAAAGCGACGTCCGAAGGTTTTAGGTACCCACATGGAGTTTCTGGCAAGTGCACTTGATGGTAAGATATCACTAGGATGTGATTCAGCTACTTGGCGTGCCTATGTGTCAGGTTTTGTGAGCTTGATGGTAGGTTGCACACCTAACTGGGTGCTGGAAGTTGATGTGCACGTGTTGAAGAGACTGAGCAATGGGTTGAGGCAGTTGAACGAAGAGGAGCTTGCTCTTGCTTTGTTGGGTGTTGGTGGAGTAGGTACAATGGATGCTGCAGCTGAGCTCATCATAGACACTGAAATATAAACATTTTCACTATTCTGAGGGCACCTTTGCCTTTTAGGATGAGTTGTACATTAGTTAATTTTCATGTTTATAAAGTTGTGCAGCATGTTGAATTAACAGTTTTGTTAAAAGTACCACGTATTAGAATGGAAAATTTTGTATCTATAACGTTCATCTAATGTTAGTAACTTGGTATATCCATCCCCAATTCGATGAATGTC

>GmMED5-4

GCGAATCTGAGCTCGCAAATAAAATATTTCTTTCGATGTTGGGACTGAGCTGAGATTGTGGGGATGGAGTGTGTGGCGAAGATGACGAAGGTGGCGCAGCAGAAGGGTAGTGATCCTCTGCTTTGGGCCTTTCAGATGTACTCCAATTTGAACTCCGCGGGAGAGTCTCTTCCCTCCCTCGAACTCGCCGAGTTCTTGGTCTCTTACATTTGCTGGGACAACAATGTCCCTATTCTATGGAAGTTTCTCGAAAAGGCATTGACGCTTCAGATCGTTCCTCCTATGCTCCTCCTCGCTCTCCTTTCCGTCAGGTCCTTTTTTCTTTCTACGTTCTCTGTTCTCTGTTCATGGAGCTCTTTCTTCTTCCTTTTCTTTTCTTTGATTTATTCCTATGCCAAGCCAAGCTCTTTTCACGTTTGATTCTATCGGCATAACTATTTTTGTTACTGTTTTTATGAGCTCAGCAGTGCCTTTTTTTCCCTAAGATTTTTACTTGGCAAGTCCATAAGTGTTGCGGAATGAGGAGTGAGGTTTTGGTGGATATTTAGCACTCATTTGTCCTTGCAACATCTGATCGGACTTGGGGTGTTACGAAATAATTATGATTCTGTAAATGGATGTAATTATGAATCTCCGGTTTCTATTAACGCCCTGGTACAATTGTTGCTATATGAACGGACTTTTGATTTGTTAAACGCTACTTAAAAGTGATATTTTTCTTCTTCAAGTTAATTCTGCATACAGGGAAAATCTAGCTACTGTTGTTTGTTTTTCACTTTTCCATACTATGACTTCCGAGTCATAACCAACTCGCATCTATTTATTTAATGATTTTTGCAGTGTAGTTTCTCTTAACAGATACAACCATACCTGGCTCCTGCTATGTGGGGTTAGGCTATATGGATTTTAGTACACTAATGTCTGTGTCATATATTCATGTAGTTTCATGTACCCTAAATATTTCTTAATAGTCTACCATCATGTTTGCTTTTGTCTCCCTTTTTTTCTCTTTACTGGAGCATTATCTTAAATTTTTTGTGTAGGTCCTCTTCTTTCGTGACCAAACCATCTTAAATGGTTTTCCATAATTTTTTTACTTTTAGAAGCCACTTCAACCTTCTCCCTGTTAAACACATTCTTAGACTTACCCATTCAGATATGCTCAATCATCAATTGCAACAACCTCATTATTGTAACCTTCACCTTTTGCTAATGACTTCTGTTCACTACTCAGCATTTTGATCCAGAGCTTTAGGTTGTCTAATTGTACTACATCATGAACAATAGACCATTATTTTTTATAGTTTTATTTTTTGATTAATTATTACAGTTTGGTGTTGATAAAGACATGTTTTTTCTGCAAAATTTGGAACAAAATCCATATGCTTGTGTTCCCTGATGTATTGCTGTTTTGGTGTGTCAATATATCATGTATCTTTATTCTTTTTGGCAGGGTTATTCCATGTAGACATGTCCAACCTGCAGCGTATAGGCTATATTTGGAACTCGTCAAAAGACATGCTTTTGAACTTAAATCTCAGATAAACAGGCCAGATTATCAAAAGTAAGCTTTAATTGATGTAACACTTCTGTTTCAATAAATTTTTGCTTCATCTTGTTTATACCTTTCAAGTTACATATACTGGGTGATTTTAACACCTCTATCATGTTCTTTTCTTTGACATTTTATGTGATTTTCTCTACTACATTTATAATTGATAACTGAAAACTGTTCAGTAATTGAATATGCTACTACACTGTATCACTATCACATTAGATCCTTTGTTTATGTTGATTAAAATTATCAGCATTTTTACTTCCCTAATTTCTTTATAGTATAAATGAATGGTTGAGATACATATGAACTGTTAATTTTAATAGTTTATTGTGTTAGTGGTGCGCTTTGGTCATTTGTTAATTCTTATGCTTATACTTGGACAGGGTCATGAAATCTATTGATGCTGTTCTTCATCTTTCCAACATATTTGGCATGTCACAAAGTGAGCCTGGCATTCTGGTGGTTGAATTTATTTTTTCAATTGTCTGGCAGTTACTTGATGCATCATTGGATGATGAAGGTTTACTGGAATTTACTCCTGACAAAAAGTCTAGATGGGCTACACTATATCATGACATGGAACTGGATAGGCATGATAATTATAGTGAACAAAGGACTGAACATCATGAGAAATTGCAAAATGCAAATACACTTATGGCTGTTGAGATGATTGGGCAGTTTTTGCAGGATAAAATCTCTTCAAGGCTTCTTTACTTGGCTCGCCAAAATTTGTAAGTATTTGCTATCCTTTTATTTGTTCTCAGCATTTGCTGTCAACTTTTACTAATAACATTGCCAATCAGAATATATCTGTTCATTCCAAAATTTCCCATAAATTTGGTTTGATATATCATTTCCTATTTTTTCATTTCATTTTTTTGGGTTAATAAAATGAATTCAAAGTATTCATGAAATTCTATATTCTACAATTATAAAGGAAGTGATTTCATGAGGTTTTTTTTAAAATATGTCAGCCTATCACATCTGATGGGATAAGGCTTGGTTATTGTTGTTGTATTTGCTCAGCAACTTTCTTCTGTTTTATCAGGCCGGCACATTGGCTTAGTTTTACTCAGCGGCTGCAGCTGCTGGGTGAAAATTCATTAGCATTGAGAAAATCAAGAACTCTAAGTCCTGAGGAACTTTTGGAATTGACTTCAGATTCTTGTATGGTCTTGTCTCGGGAATGCAAAACAAATTCACAGAAAAAGTTCCAAACAGTTATGTCTTTTGAATATCTTTCTTCTTCTGCCTCACTATGTCATGGTGCAAGTCATTCTGCTCTCTGGATTCCTCTTGATCTGGTGCTGGAGGATTCCATGGATGGCTATCAAGTTAGTGCAACAAGTTCTATTGAAACAATTAGTGGTGAGATTGAGAATAGTTATCAGAATCTGACTGAAATAGTTGATTTTGTCTTTTCAAATTTAATTTAGGTACTTGTCACATGGAATTTTTTGTGTAGGTTTGATAAAGACCCTGCGAGCAATAAATGGCACCTCTTGGCATGACACTTTTTTAGGCCTTTGGTTAGCAACTCTTCGTCTTGTTCAGAGGGTATGACTCTTTCTCTGCAACTCAATCTGTTTTCCCTTGAAGTTGTGGTTTTAAATTTATCTTGTCCATTGATTGCTTCTAGTATTCCGTTACCATACACTTTGTAATTAAGGAATTTGCATAAGCAGTTTACTCCTCCAATTTAATTGTTGATTTGTTTATAGGAAAGAGATCCCATTGATGGCCCAATGCCTCATCTTGATACTCGCTTATGCATGCTCTTGTGTATCATACCTCTTGTTGTTGGTGATCTTATTGAGGAGGAGGAGGAAAGGACACCAGTTGATGAAAAAGACAGTGGACTTACAGATTGCTGGAAAGAGAAAAAAGTTGCTGGAAAATGTCATAATGATTTAGTCTCGAGTCTCCAGGTATTGGGTGATTATCAAAGCTTGCTCACTCCACCTCAATCTGTTCTTGCTGCTTCAAATCAAGCTGCTGCAAAAGCAATGCTATTTGTTTCTGGCATCACAATAGGGAGTGCATATTTTGACTGCCTCAACATGACAGAGATGCCAGTTGACTGCTGTAAGTTATATTGCTTTCAAAGTCGAATAGAAATTGTATATGGATCTTCAAAAGTTAGAACAACTTTTCTAAATTTCAGCAGTGCATTATTGAGGATGTTGTTTTTATTTTCTGATTCTCTGTGATTTTCTTCCAAATTTGGAAAAGTATATGCCTCATATTTCGCATGCTGTGATGAAGTGCAACAACACATCAATGTTTCTGATTGCTGCTAAAATAATCTCTGTTATCTAATTTAAGTAAATCTACACTTAAAAAATTATACTGATTATAAAGTAGCTGGAAAGTATCAAAAGCAGGAAGTTGTGCAATGACACACATTTCTTTAACATAATGCTTTCAATCTGCTATTTGTAGTTGAGTCTGATTTTGTTTTTCTGATTCTGTTAAAATTGCATAAGGTTTATAGTGACCTGCTTTCAAAGTAATTTTAAAGGAGCTAATTGTCTATAATGCTATGTGGTCTGATTACCTTGGTCACCCTCTTGTAAAGTCAAATAGATTATGTAATTTGAATCTGGGAAAACTCTTCAAAAGTAAACGTTGTCGAGTGAACTTATACAATATACTCAGAATACCTGTTAAAAAAATGGTGAAGACGCCTCCTTGAACACAGAATATCCTATTTCATCACTTGCAAATTATTATGAGCCTTTTAAGACACTAGTGAGGATACATATCAATGAGTCTGATTTCTACTATATCTGTTGGACTAATTATGAATTTTTCTTTGCTGATAGCAAAACAATTGACTGTATAAAAGTAGAGATGATCTTATTTGGCTAAAGATTGTAATAAGCAAAACCATTACATAGTTGAATTATGTAAAATATGACCCTCTCTCTCTGAGAGTTGTTCCATTCCTGTATTCCATCAAATTTTTGGTCTAGCAATTCACTTTCCTGTAATTATTGATTCTGTATTTGATATTTGGTAGTTAATTTTGGAAAGAACATATATTCTTCACCTGATGCTTCATTCAGAAACTAAAGATTTTTGCAAAGATTAAAATAAAAATTCTTGGTAAAAGTATTTTTCACTTAAGAACTTCTAGCCGCCCTACAGATAACAAAATTCAAAGAGCAGTTAATGTGTAACCACATATTGACCTAATTCAACCATTTGAGTGGTTACATTTTCCTAAATTGTAATAATTTTACCAAGAATTTCTTGTTTGTAATTTTCCATATATATGTGTGTGTGTATGTGTATAAAAATATAAAATTAAATCAATTAAACTATTTCTTTTTCAGCTGGAAACATGCGTCATCTGATAGTTGAGGCTTGTATTGCCCGCAATCTACTGGACACATCTGCATACTTATGGCCAGGTTATGTAAATGGATGTATCAATCAAATACCTCAGTGTATGCCAGCTCAAGTACCTGGCTGGTCATCATTTATGAAGGGAGCCCCACTTACTTCAGTGATGGTTAATGCTTTGGTTTCAAGTCCTGCTACAAGGTATGATCTAATTTTGGCTTATTCAGTCAATTTGGTTCGTGTACATTAGAGAACCTGCAATGTTCTAATTTTTGGCAAAGGTGTTCCAGTATAGTATTTATTGTGCTCCCTCTTTGTAGGTACATAGCCACAATGCTATGACCTTTACCAAACTATTTTTTGGAAGTTTTATATTTTAAACTCAGTCCTAAACAGGCCATAAAAGAAATGCCGGCAGTTGAAAATTGTAAGCTTTTTCATGTGGCCAACCTCTACAAATTTCTTTTGGGGATATCTATCATACCATACGAGTGATGAAGGTTTTGTTCCAATCTCATCTTTAATGCATCCATCACTGTATGGACAAATTAACATGCTGAAACATGAGATCATTTACTAAATGCAACAATAATTCATCATAAACTGAATTTAGAACATAACAAGGGTGGCTATAAGTGGGTAGAATTTAAATTCATAAAATTTGTTTGCCTGGTCTTCAGTTTTTCTTTTTCTTTCCTGAAGCCAGGTATGTCAGATGCTCTTTTTTGTAAATTTACCAAAAGGTAAAGCAACTGAATTTACGCTCCTGGATTTGATGGAAGCTTAGCAGAACTGGAGAAAATTTTTGAAATTGCAATTGGGGGATCAGAAGATGAAAAGATATCTGCTGCTGCCATTCTTTGCGGGGCATCCCTAATTCGTGGATGGAATATACAGGTAGTGAAGAGTTAGACAGTAGAGCATATAAAAGCTAGTCATTTGCTCAGTTGTGTAAACTGTAGAGCACATCCTTCTTTCTAACCACCTTCTTATCCTAATAGAATTTGGATCTCTATTTAATGCAGGAACACACTGTACATTTCATTCTGAGGTTGTTATCTCCACCAGTTCCTGCAGAAAACACTGAAGGAAACAACTATTTGATTAATTATGCTCCAATACTGAATGTACTCTTTGTTGGAATTGCATCTGTTGATTGTGTTCAAATATTTTCGCTCCATGGCTTGGTAAGTTTAACTCAATCCCTAATTTAAAGTGTTATTGATAGGACTTAGAATTTATATAAATGAAAGATGAAGACCAGCTACTGCAACAGAAGAAATCCTTTGCAAACAGAAATACGACCAAAAAAATAAAGTTAAGAACTTCTGCAGAAGATAAGATGGCCTAGAGGACTGTCATACACAAACCCTAGTAGCCTAGTACCACGCTCAACCTTTCCACTAATTACTTGAATTTTTTCTGTCTTCCTTCAATTGGCTCCTTTTTTTAATTCCCATCTCTGAGCCTTGTCACAACGGTACAATTGCTCCTTGGTAATCGATGGGTCATGGGTTCAAATCTGGAAATAGCCTCTTTGCATAGGCAAGGGGAAGGAAGGCTGCATACAATGACCCTCCCCTATACCTTTGTGAAGCGAGGAGCCTCTGGCACTGGGGTACATTATTTAGTTGTTTTTTCTCCCTTCCCTCTCTCCATTCATCCCCAAGTCTTTTCCTCATTCACACCCAAATATTTAGTCCCTTTACTTTAGTCTCATGCCAGAGCTTTGTAATGGGTTACTCCCTAATCTTCTCCTCCAGGCTCCAACTGCATCTGGCCTATTACTAAACATCCCAGCACACTTAGCTCCCATTTTCCTGCTATAAACTTGGTTAAAAGGCAGCTATTTAAGAAACATTCAGTAGACAAATTGATTGTTTATGCCTTATATAGATGTTTACCTTGTATAGGACCCTTGTATATGTACATGAGCTTGGATTATTATTATTATAATTTGAATTAATGCATTTCCCAATTCTCCAACCAGGTTCCACAGCTTGCATGTTCATTGATGCCAATCTGTGAAGTTTTTGGGTCATGTGTACCAAATATCTCATGGACACTAACATCAGGGGAAGAAATATCTGCCCATGCTGTGTTTTCAAATGCATTTATTCTTCTTTTAAAGCTATGGAGATTTAACCGACCTCCTCTTGAATATGGAATTGGAGATGTACCCACTGTTGGTTCTCAATTAACTCCCGAATACTTACTATTAGTACGTAATTCCCACTTAATGTCAGCTGGTAACATCCACAAAGATCGCAACAGGAGGAGACTCTCAGAAATAGCAAGTTTATCATCGCCAAATTCTGTATTTGTTGACTCCTTTCCAAAATTAAAAGTGTGGTATCGGCAGCATCAAGCATGTATAGCTTCAACACTCTCCGGTCTTGTCCATGGAACCCCATTTCATCAGATTGTTGAGGGGCTTCTTAACATGATGTTCACAAAGATTAATAGAGGAAGCCAGACAACTATTACATCTGGAAGCAGTAGCTCTTCTGGACCTGCAAATGAAGATACCTCTATAGGGCCAAAGTTGCCTGCTTGGGATATTCTTGAAGCTATTCCCTTTGTAGTTGATGCTGCTCTTACAGCCTGTGCTCATGGAAGACTGTCTCCACGTGAGTTGGCAACAGGTATGTGTGTTTTGATCAGATAAAATCCAATGGAACACACCAAATAGCTTGTAGAATTCTTGATTTCTAAATTACCGAAGAGAATTCAGTTAGTTTCAAAATTCTTGCTGATGTATTTTACCTCTAAATATAGAGCAAGAGCAACCAAATTCATGTTCATATAGACGTTCCTTGATGCACACAACAATATCATGATACCAAAATGCTTTTCTTAATACCAGGGCTTAAAGATTTAGCTGATTTTCTTCCTGCATCTCTGGCAACCATAATAAGTTACTTCTCTGCTGAAGTAACTAGGGGAGTTTGGAAACCTGTATTTATGAATGGAACAGATTGGCCTAGCCCCGGTGCAAATCTACTGAACGTTGAGGGACAGATCAGGAAAATTTTGGCTGCAACTGGTGTAGATGTGCCAAGCCTTGCTTCAGGTTTTCTCGTTTTTATGACCCTAAATTCTATATACTTTTCTAAATTCCTGACTTATAATGCATTTTCAGGAGAGAAAAATTACATTTACAACTCGTGATTCATTGCAATTTTTTCTATTAAATTTATCTTTTACACGACTTGAACGAGTATTCAAACAAATTCAGTTTAGGAAATGTCTTCCTCCATCCTCCTTTCTCTTTCAACATTATTATTAAGTTGTTATTTAGTATTGTGACCAATGGTCATTTTAACAGGAGACAGCTGTCCCGCGATACTTCCATTACCCTTGGCTGCCTTCACAAGCCTTACCATAACCTACAAAGTTGATAAGACATCAGAGCGTTTTCTTAATTTGGCTGGCCAAACATTGGAGAGCCTTGCTGCAGGTTGTCCATGGCCTTGCATGCCAATTGTAGCTTCCTTGTGGACCCTAAAGGCAAAACGTTGGAGTGACTTCCTTATCTTCTCAGCATCTAGAACTGTCTTCCTCCACAACAGTGATGCAGTAGTTCAACTTATAAAAAGCTGCTTCACGGCTACCCTTGGCATGAATAGCTCTCCTATCTCAAGCAGTGGTGGTGTTGGAGCCCTTCTTGGACAAGGATTTAAGTATCATTTATGTGGAGGGCTTTGCCCTGTTGCTCCAGGAATTCTCTATTTACGTGCGTATCGGTCAATCAGAGATATTGTTTTCTTGACAGAAGAAATTGTTTCCATCTTGATGCACTCTGTGAGGGAAATAGTATGCAGTGGCCTGCCAAGGGAGCGATTGGAAAAGTTGAAAGCAACCAAAGACGGCATAAAATATGGACAGGCTTCACTTGCTGCATCAATGACTCGGGTGAAGCTTGCCGCTGCCCTCGGGGCCTCTTTAGTATGGATATCAGGAGGCTTGATGTTGGTTCAATTATTAATAAAAGAAACTTTGCCATCCTGGTTTATATCTGTTCACAGATTAGACCAGGAAGAAAAGTCTGGTGGAATGGTTGCAATGCTTGGTGGATATGCACTTGCTTACTTTGCGGTTCTTTGTGGTGCTTTTGCTTGGGGAGTTGATTCATCATCAGCAGCTTCAAAGCGACGTCCAAAGGTTTTAGGTACCCACATGGAGTTTCTGGCAAGTGCACTTGATGGTAAGATATCACTAGGCTGTGATTCAGCTACTTGGCGTGCCTATGTGTCAGGTTTTGTGAGCTTGATGGTAGGTTGCACACCTAACTGGGTGCTGGAAGTAGATGTGCACGTGTTGAAGAGACTGAGCAATGGGTTGAGACAGTTGAACGAGGAGGAGCTTGCTCTTGCTTTGTTGGGTGTTGGTGGGGTAGGTACAATGGGTGCTGCAGCTGAGCTCATTATTGACACTGAAATATAAACATTTTCGCTATTCTGAGGGCACCTTTTCCTTTTAGGATGACTTGTACATTAGTTAACTTTCATGTTTATAAAGTTGTGCAGCATGTTGAATTAACAGTTTAGTTTGGAAAGTACCAAGCATTAGAATGGGAAATTTTGTATGTATAACGTTCATCTAATGTTAGTATATCCAGCAGCCCTAATCGATGCATGTCCTCTGTTTAATGGTAA

>GmMED5-5

TAGTAATAGCGAGGATGGGCTTGAAAGCATAACCGGCGAGTTGCGCGAAGGCGTGACCTACCCCCATCAATTTCAATACTACTATCCAACCACATCCCAAAACCCAACAACACTCCCTATTTTATCCTTTTTTGATTCACTTCCAACCCAGTAGTGTGTGTGTCCCCTGATTTACATTCCATTTCTTTCCCCCAAACCCCTTAAGCCAAAAATGACACCCCCACCAAGAAACTAACCCCAAACAGAAGACATGGAATTGGCGTTGGCATTGGAGTGGGACGATCAAGTGCGGGAAGGCGTTCTAAAGAAGCTAAAGCTCTGGCAACAACGCAACAACAAACCCCCAACGGCATGGGTCACAGAGCTAGTGGAGCATTTCAACACACTGGGCATTGCCCTACCCTGCCCCGAGTTAGGGGAACTTCTAGTGTCCCAAATATGCTTCGACAACAACCACCCTTTGATCTGGAAGTTCATCCACCACGCTCTCTCCTCGCGCCTCCTCTTCCCTCTCCAAATACTCTCTCTTCTCTCCTCCAACGTCCTTCGCCACCGCCACTCCCACCCTCACGCCTTCGCCCTCTTCCTCCCTCTCCTCGCGCAACACGCCTTCTCCTTCCTCCCTACCCTCTCCAACAACCTCAAGTAATAAATTAATTTAAACCCTAACCTCTCTCTCTCTCTCACTTCATCAATTTCATACAGTTCCTTGAGTACTGCCAAGGTTTTTAATATAATCACTTTTGTTGATATCGCCGCAAATAAAACACAAATGCGGTTGATAGGGTTTTCAATTGCGGTCGCGGACAGTTTTTTTTAAAAACCTTGAGTACTACTAAGTACTGTGTTTGCGCTGTTCTCCAATCGGAATTAGAGTTTTACTTTTGTAATCCGTGTGTAATAAATGTTTCTGCATTTTAAGAATTATGCAACTTAGCAAGGTATAAGACGTGCTTGTTTGCATGATTTGTTTAATTCTGTAATGCAAACCTTTATCGCGCAGATTACTGTGTGGCCTGGAGTGAAACTGTACTTTTGATTGGAGAACAGCACAAACACCAAGAAGCGCGTGAGGAACTGTGTCTGTCTATGTTCTTTTGTTTACTGAATTCTAGAAAATGGTGGTTCCTTTTGCAGGATGGTGAATTCTGTGGATGCTGTGATGCGATTTTCGGAGACTTATAAGATACGCGATCTGGAGCTGGGGCACGTGTTTGTGCTGTTCTTTTACGATATTGTTGTGGCGTTGATTGATTGCGTGTTGATTGATTGGGGGTTTCAGGTGACGTTTAGTGAGAAATCGCGTTTGGTTACTGGAGGAGGTGGTGGTGATGATGAGGAGGATTATATGGAAATTGATCGGAATATGACGACGATGACACAGAGTTTTGAGAAAAGTGAACAGATAAGGAAGCGGAATTCATTCACCGCCTTAGAGGTATTGGAAAGACTAACCGAAAGCAGAAAGGCTACTATTTTGCTCCAGTCTGTTCTCTTGAACATGTATTGTCTCCCACTTTTATATATATGTATTTTATCTTGAAAGTTATGAGTAATTGTTTCTCAATTAATCAATTGCTATATTATGATTGTCTGTGTAACATATTTTGCCATCAAACCAATCTACAGGCTGAAATAATCTTTAGCCTAATCCGTTGAGCTTGAAAAAATAGATAACATTTTTCGTTATTGCCTGATTAAACAATGTAGATACTAAAATTTGTTCTCTGTTCATAATTTTGTTACTGTCAGATATCTTGGGATCAGTGTGTATACTATGTGTCAGAATGTTGTTTGATTGTGCTATCTTCACTTTAATCAGAAAAAAAAACTATAGATGAGAATGATTCTTATATTGTACATGTGACAGTATGTGAGAGAAAGCATATATAGAAGTGGTGTCCTGGTCTAGTGAATTGTGAATGCTAAAAAGTCTTAGAATGTGGGTTTTGGGTTTTTCCCAATACACCCCCTCACACCCAGCACTTTTGAGTTTGGTGCGTGGATAATATGGTGATGACCCTTTAATGGATCTAAGATAGGCCCTTTAACCCATATGAGTGCAAAATATATCTAAGAAACGATGAGAAAGAGGCTCCAGCTTTTATGGTAGATTGTGCTAGAATGAAAAATCATTTCATATTCCTTCTTTTCATGTTCCTGTCTTGCACCAATTGGTATTAGAGCCAAATGCAATCCAATTGACCATGGCAAGACAGAGCTGACTTGGATGAGTAGGAGTATGCCTGAAAGTTTCTCATGTACAATCATGCTTTAAAGAAACATCACGTGGGAGCCTTCTGCTTCCATGGGGAATCAGGATGGCAACAGTAGTCCAGTGGTAGGCATTTTAAGTCTAGCTTAAAAAAACCATTGTTTATTATAATTCTCCTCCATTTTATAATATCCTCTATTGAATAGTTCAACAAAAAAGAAAAAAACTGTTTTCAGCTTATCAATGAAGAAATCATCTAACAAGTAGGAGATGCCGAGATAATAGAAGATCCAAAATTATTCATATCCTTCAGGATTTTTCCTTCTGAATATTTTTGATGGGTAAACATATTTTGGATTCATTAATTGACTTATCAAGTAAAATCAAAGAAAATTGTTTGCTCTCCATTCAAGTTCAAGTTCCAATATACCTTTATAAGGGCCTGCTTTCCTTGTTTACTGGTAAATGTTGCGTGAAACATAAATTGTTTCCATTATCAAATGGTATGGGAAATTTGATTTTATTCAATAGTACATATTCATATGTCGAAACTCCTAGTAATCCTTGCTCTTCTTGTCTGAGCAGTGAGCACTTGTGAGGATAACCAATTTTAGTTCCTAAAGTATATAAGTTAATTTTATTTATACAACATTTTGCATGAGTTTTATTGTATGCATATATGTACTTGCTCTCTAAGATTTGTGCTGGGAAATTGACTAGGTTTTTCCCATTTTGTTTTTCATTGTGTATGATAGGCCTGAAAAATTCAATTGCCTCCAGCAGAGGCTCCAATTTCTTGAATCCCTTGAGTTGGCCTCATCAGAGTTAAAATCAGTAAACCAAGTCCTAACAAAAGTGTCTGCAAGCATTCGTGGAGTTTCTCGTTTTGATTACTGCCTAAGGAAGCATCAGCTGGTTGGGATGCTTAAGGCATCATTGAGATGCAACTATAGATTTTGTCAGTCTCCTTGTTGGGTTCCTTTTGATATTTATATGGAGAATGCTATGGATTCTAGACAAATTCCTACCAAATCAGCTATTGATGTGCTTACAGGTATATCCTTCGAGAGAGTTTACATTGGATTTACTATTGTTTACTAAGAGGTTATTTTCAGAAAGTTATGAGGATTCAGTTCTTTCCTAATATTCATGAACTATTTGCAGAAGCAATCAAGACACTTCAAATACTAAACCAGGCTAGCTGGCAAGAAACTTTTCTAGCACTTTGGCTTTCAGCCCTTCGGCTTGTGCAGCGAGTTGAGACTGGTTCATAATAATCTTTTGTTTTCATTGTAGTATTAACCAAATATTTAACTTTTCCCCCCGCCCTCATGGTTTATTGCAGGAACGTGATCCTCCTGAAGGCCCTATTCCCCATCTTGTGGCCCGTCTATGTGTACTTTTATGCATTGTCCCCTTGGCAATTGCAAATGTTCTAAGGGATGACTCTGAACATAATTCGTCATCTGTTCAGGTGTCTATGGAATCAGAATACAGGCATGAAATGAAAAGTGGTAGTTCTATGAAACTTGGACTGATTTCATCTGTTCAGGTCCTTGGACACTTTTCTGGCCTCCTATGCCCTCCTACATTAGTTATTGATGCAGCCAACCAGGCAGCTAGAAAAGCAGCAAGCTTCATTTATAATACTATGAATGGAAAGGGTGAATCTGGCACTGGAATTCATGCTAATACCAATACAAAAGCAGGTTGATATTTTGGAACTAAATCTTTACGTCCGTGATTCCATCTGCATTGTTTACAATGAATAAGGATTATAATCATGTGCATACAACTTTCTTTATATTTGTTTTCTGCAAATCTCTATGATAATTCTGAATTAATTATCTTTCAGGCGGGAACTTGAGGCATCTCATAGTGGAAGCTTGCATAGCTAGGAATTTAATGGATACATCAGTGTACTTCTGGCCTGGTTATGTCTCTACATCTGTTTTGTCTTTGTCAGATTCGTCACCACTAGAAAAATCTCCATGGTCAATATTTATGGAAGGAACGCCATTAAACAATACCCTTATAAATTCTCTTACAGTGACCCCTGCCTCAAGGTTTATTTTTGTATCAAATTTGATTCAACTTTGGTGTTATCTATACTGCCGATGATTGATCTTCATGATTTTTCTTCTGTCCTAGCCTGGTGGAGATAGAGAAGTTATACTATATTGCATTAAATGGATCAGATGTGGAAAGGCCAGCAGCTGCAAAGATTCTATGTGGTGCATCCCTCAGCCATGGATGGTATATTCAGGTCACAAATTCTACTCCTACTAGAGATATTGGATATTGCAAGTCCTCCTGCAATACAGTTGGTTAGATGAGCTACAGGTTTTTCTTTGGATGCATATTTCTTAAATTATGAAGTCTCACTCCCACTTTCACCCTAAAGTTTACAAATCTTGTTACCAGAGGAGGTAAAATTGTGAACTTAGGTTTGCAGATTTGTTATAATATAATTATTAAAATTGAGAATGGACTGTGCATAGTTCCACTCTAAAAATTAGCTCAAGGTAGGAGGATTACCCAAGTTATTTTAAAGGGTCTTTTGACTATATACTCAACCAATGTGGGAGCTTAACCATAATAATCTTGTCTTAGTCTCATCTAGGAGCTTTTATGCATACAAGTATGATGGACTGGTTTGGAATCTACTGGATCTATTGGCATGGAATTGAAGCAATCAATTATTGCTGTAATGTAATTATTAGGCTGCTCATTGGTTTCCAACCCTATTTTGAATTATTGTACCCTCTTTTGTTTTTCACTTAAGTATTCTATATCTTATTGTTGACCTGCCCAAATAATAGAATTGAAGAACAGGGTATGCAATACAGGCATTAAGTTATTTGGACCATTAATTGAAAATTTCAATGTATTATTTATTTAGTTTAATTCAATCCTTTGGTTTGGCCTAAATACTTGAAGAAATCATGAACCTGGATTGTGATTTTACTGATTTTGAACCTACTTTTTCTCCTCTTCTATAAACTGGAAAAAAGTTGCCATGCTTTTTGTTCTTTGCAGTGACGACTAATGACAAAACTTGCATTAACTCTACTCCATTCAGATGATAGTTATGTCTTTTGGTTGTACTGGCTTATGGATTAATGTGTGAATACTCAGTTCTTGGTATTGCTTTCTTGAATTTTGTGTCCCTTGTCATTTATTGTTTATGATAATCTGATGTTAAACTTTGTCTCCTTGGCAGGAACATGTGGTCCACCATGTGGTCAAGCTTCTTGCTTCTCCGGTACCTCCTAGTCATTCTGGATCTCAGAGCCCTTTGGTTAATAATATGCCCATGCTGTGTGCTGTCCTCCGTGGCACTTCCTCTATCGATACTATTCATATACTTTCTTTATATGGTGTAGTAAGTGAATCACTTCTATAATTTGTTTGATGGCTGTATGAATATGATACCCCTTGTAGATTCTTTCTCACAAATTTATGAGGTTGGAAATTTTTTGGGTCTACTCAGCAGCAGTTGGCAACTGTTGAAAATGTTAGGCCTTGTTTGGTGCTTGGAATTGAGCTCCAAATCAACCTTGGAATCAGAATTGAACTATTAAATTCTAAATTTCATGTTTGATATGCCTTTTTTAGAATGGGAAAATTCAATTCTTTATAAAAATTCCAATTTCCATGGTTGGAAAACAGTTTTAATATATAACACTTTCCAAGTCTTTACCTAGATGCTCAAAAGTGCAATTCCCAGCTACAATTATGCGCCTATATCCACTTCACCTCCAACTCTGAGTAATACCAAGGAGGAACATTAATTTTATGTTCTTCCAGCAATTGGAATTTCAATTTCAATTTTGTTTTCTGAACTAAGCTTGAAGAGAAATCTTGTAGAGATTGAAATGAAGGTGGAAAAGAGAATTATAGAGAGATGAGGCAACATTGATAGGCTGTAGGATGAAACACCGTGTGTTCTCCCTGCCTACACCTCAATTTATATGAGGATGCCGTAGGAGGAGTATTACAAGCATGTTGTGGCAATTAATCAGTCATCAGCCTTGAAATTCAAAACATTTACTTGAAACCATTAACATTTCTAACAGTAGCTGCAAAGTTTATAGAAGTCCATTTTTATTTCTTTTGTGGAGACGCTAGGTTAAACAAGTTAAAGCAACCTTAGATAGTTTGTCTAGTTCATGCACCATTTCTAAGCTATTAGTGCACAAACTTCCTTTAATTTCTTTTCCAGTCACATGTTAATGTCAAATTTTTTATTAGAAGTTCTTTGTGTAGGTCCCCGCAGTTGCTGCTTCTTTATTACCACTTTGTGAGACATTTGGCTCAATTAAACCGACTTCAAATAGCACTGGCGATGAGTCCTCCTCAACATCGACATCAACATCAACATACATGACCTTTTCTTTAGCCTTTCTTTTCCTTATTCGTTTATGGAAATTCTGTAGACCCCCTCTTGACCTGTGCATCACAGAATTAGGAGTTGCAGTTGGAGGTTTGGAGTATATTTTGTCATTGCACAATAATCGTGCCATGTTTTCCCAAGATAAACTGAAAAGCAATCCAAGTCTCTCTGATTCTGCATCTGTTAAACCTGTATATATTGATTCATTTCCAAAATTACGGGCCTTGTATTGTCAATACAAATCTTGTGTTGCTTCTGCCCTTTCTGGCATTTCTACTGGAAATTCCATTCATCAAACTGCTAATATGATTTTAAGCATGATTTACCAGAAGATAACCAAAGGTGGAATTTCATCAAGTAATTCTTCATCACCGACTACTGCTAGCAGTAACGCGTGTAGTTCTCTCATGAATTCTGGTGAAGATAATTTTCAAAGACCCTTGCTTCCTGCATGGGAAGTACTGGAAGCTCTTCCTTTTGTCCTTGAATCAATTTTAACTGCTTGTGTTCACGGGAGGATTTCATCACGAGAATTGACCACAGGTTGGCTAGATGCTAGCGTTACTTCTCTATAAGCTTTTTGCATCCATGAATTTGGTCCCACCTTTCTTGTGTCTTTTTTAATAGATGATAATTAAATCATGAAAATTAAATAATCTTTCTGGTTCCAACTCTTTTTTGTCAATATTATTTTCTGGATGTTAAAATTTTGCTGGATTGCCAAAGCCATTGTACTAACTCCTGTTTAGTTTCATTAAGCCATCCCTGTTTTTAATTTTAAGATTGTAAAGTTCAAATGCAAAGAGGTCACATCGAATTAGGGTAAATGGGGGGTGCTGATACTTAATAAACTAATAAATACATAAATAAAAAATGTTGGTTGTTGTAGTTAGAAAAAAGTTATTGGACAGAAGCACATGTATTTCTGGTGCTACCTTCATTTAATCACTCTAACTCTTATGTGCCATTCACTTGCAGGTCTGAGAGACCTTGTTGATTTTCTTCCAGCATCGCTTGCTGCCATTATTGATTACTTTTCTTCAGAAGTTACTCGTGGTGTATGGAAACTAGTTCCAATGAATGGAACAGATTGGCCTAGTCCAGCAGCACTTATTCAATCAATTGAATCAGAAATAAAAGCTATATTAACTCATGTTGGCGTTGAGGTTCCAAACCGTTCTTCTGGTACTTACCTTGTTCATTATTTTAATATACCTGTTTGATTTTTATGGATTTCTATCGAGTCTCGCAAAAACATCACACATGATACATGCACACTTATAGATTGTATTTATGAGATTTAGGGTTTAGGGTCTCTTAAAATTGCACTTTATTATGAGATTCAGTTCTTTTTAATTTTGCAAGCCCTGCTCCTTCTTTCCTCTCTTGTGCATTTATATTTTATGTTTGTGATACGTTTGGAGAGTTATATTTGGTAAGAAAATATTTTTCTTGATATAACCTGTTTGTTTTGTGTTTTTTAAAATTATGTTCTGCAGGTTTTTTTAGTATAATTTGCATTTTTTGGCTTGCTGCTATTTTCCATTGACAATACTAGTAGATAGAGAGAAGCAAATCCTGAAAGCCTGAAAATAGCAGTGTTGTCAAATAGTCATGTTTCAAGCTATGCTCCTTCAGTCTCACATCAATGCTGAAATTTGTCATTTTTCATCAATTTAAATCTAGTAATGCCTAACAAAAATGTTTAAATTAAAAAAAAAGTACAAAACACTCATCATATTAGAGGCCATTCAATGTTTTCTAGTCCTTGTTTTAATTATTACACCATAATAGTTTGTTTTAGGGTCTCATTTATTAATAAATGCCACTATTTTCCTTTACTATGCAGTGATGATTTTTCAGTGGTGAACCTCTCAAGTCTCAATTTAATCAAACATGCTCTAAAAATATCGAAGGGTTAAAATTTTTTATATCATTGATTGGCTTTGCTGAGAATGGTGGATATCATAAGTAGCTGGTTCTTCTCTCAGCTAGTGAGATCTTTAAATGCTGTCGGTAACTTTAGTTTGTATCTGATATTTATATTTATTTGTTTTAGACTTTTAGTTTATTACATGAAGGATTTTGGGTTATTCACTTAACATGGTACAAACTGCTAGGTGAGAACACATTTCATCCATATATATGTAATATGTTTGTCAAAGACAGTGTTCTTGAGGTGATGAATTTATCACTATCATTACAGACATAAATCTGCAAACTTAATTGTTTTCTAATCCCTTCTTATAATTAATAGGTATACTTGAGATTAAGCAATCCAGTGATTGTGTATTTATATTGCTTACAAGTTACATTTTTGTTGTATGGTAGGTGGTTCACCGGTAATGCTTCCTCTACCAATGGCAGCTCTGGTCAGTTTATCCATTACTTTTAAACTTGACAAGAGCCAGGAGTACATGCATGCTATTACCGGAGCAGCTTTGGAAAATTGTGCATCAGGTTGCCCTTGGCCTAGTATGCCTGTAATTGGCTCTCTGTGGGCCCAAAAGGTTCGTCGCTGGCACAACTTCATTGTTGTATCAGGTTCCCGTTCTGTGTTTAGACACAGCAATGAGTGTGTAGCTCAGCTGTTGAGAAGTTGTTTCACCTCATTCCTTGGAACATTGTGTGTTTCAACCTCAAAACTGACTGCTGAGTGTAATGTGAATGGCCTGTTGGGTAGCACCATTACTGCCCCTGGTCCCTATCCTTTTGTCGCTCCTGGATTCCTTTTTCTTCGATCTTGTCGAAATATACACAATGTGCAGTATGTAAACGATATAATTGTGGGTCTTGTGACAGAGTACTCTAATGAATTAGCCGGTAGACGGACGGGTGCCAGCTCTCGCCACATAAAATCCAATGAAGTATCACTATCCCTTTCCGCTCAAAGTGCAAAAGAGGTGGCTACACTTGGTGCAAGTCTTCTATGTGCAGCAGGTGGGATACTACTGGTCCAGGAATTGTATAAGGAAACTATTCCTACCTGGCTGCTGTCGTCAAGGGATGTGAAGCAAAACAATGATAGTGTTGGGTCATATATACTTGAGGGTTATGCTATGGCATATCTGTTGATATTGTCTGGGTCAATCATATGGGGTGTCGGGACCAAGTTACCATCAGGGACATTCAATAGAAGGAAACGTACCATTGAGGTCCATTTGGACTTCTTGGCAGAAGTCATGGAGAAAAAAATCTCACTCAGTTGCAATCCAATTACATGGAAAACCTATGTCTGCTGTTTGGTGGGATTGATGGTTAGTCTTGCACCAGCATGGGTTCAAGAAGTGAAGGTGGATACTTTGAGGAAATTGGCGCGCGGATTAAGCAGATGGAATGAACATGAACTGGCACTCTCTCTGCTACATAGAGGGGGCACAGCAGCTATGGGAGCTCTTGCTGAACTTGTCAATGTGATTGAGTCTGAACATATGCTGCCGTGCTCATAATCGAATTTATGAAGACTAAATAATGTAATGACAAAAATTGCTTCAGAGAAAATAGCAAGTCTACTCGTCTATTCTACAAGTGAGGTGGGAATATTGCTATTTTGAGTATTTTCTCCCAATCCCGTTAGTATGGACTTGTGGAGTCGAAATGGTAGTATGCTCCTCCACAACTCTAGTCTTTGATGAAATTCTTTTAAGAAAAAAAAAATATTATTGCTCATGAAAAATTTATATTGCTGTTTTTTGTCTTATATTTATATGTAAATGAGTCTTTCATCAGTTCATTTATTAATCAAATAGCACCAATTGTCATTAAGTTGACATAACATGTCAATTGTAGAAAATTTAAAATTTAAATTATTGTTCAAAATTGAGATTGACTTTGATGTGTATAAATCATTTACGTTGATTTGTAGGATTGATTAATTTTTATTTTTTAAAAAAGTATTTTAAAAGAAACCTTGTATATATAAAA

>GmMED5-6

TTGTTGCATCAGCATAAGGAAAGCGATGGGAGAAGTGTTTCAAAATATGAGCGAAGAAGAAGAAGAAGAAGAAGGAAGTGTGATGGATGGAGTTATGGAAGTAACCAAGTGGGCGCAGGAGAAGAAGACAGACCCTCTGATATGGTCGATCCAGGTCAGCTCCGCCCTGAACTCAGGCGGCGTCTCCCTCCCTTCCGTCGAGCTGGCACAGCGGCTGGTCTCCCACATCTGCTTCGAGAATCACGTGCCCATCACGTGGAAGTTCCTCGAGAAAGCCATGTCCGTCAGACTCCTCCCTCCTCTGCTCGTCCTCTCCCTCCTCTCCGCCCGCGTCGTCCCTCAGCGCCGCCTCCACCCTTCCGCCTACGCTCTCTACATGGACCTCCTCAGCCGCCACGCCTTCTCCCCTCACATCCACTTCCCCAATTACCTCAAAGTCATGGCCTCCATCCATCACTCCCTATCTCTTCCCCCCTCCAACCACCACCCTCATCCCGGCGTCGTTCTCGTTCACTTTCTCTTCTCTATCGTCTCGCAGCTTCTCCAATCTTCCTTGGACGACCAAGGATTTCTCCAACACAGCCCCGATCCCTACAACAACAACGATGCCTTGCACCGGAAAAACACTGCCATGGCCATTGAGATTATTGCGCGCTTTCTTCACCACAAACTCACTTCCAGAATCCTTGCATTGGTTCAACGAAACATGTATGTTAATAAACACCTTCACTTCCCTTCACTTTCATTTTTGCTACCTCCTTATTCCTTAGGATAAAACTTACATTCACTTCCTTGCATTTTTATTTTTTTTTATACTCAGGAATGTAAATATTTTTAATGCTCATTATCTGCAAGAAAAAGATTTTTTGGACAACCTTGAGAGAGGATAGATAAGTGATTGATTTTATTGATGATGTAATGCAATGATAGGAAGAGAGAGAAAAGATAGTTGTTGATTGGTTGTTTGAAATTTTTTGTCATTAAAATATTATCACTCTCTATTTGCAATTAGGATAGGACTAAACTTAGATACAGTTCCTTCTTCTATCAGAATTTTTAAGTTTTTATCTCAGCAATTTCAATATTTATTATGCTGATTATTCAACTCAAACTCCAATTATAATTCAAGAACAAAATCAGAAGCAACTAAAAAACTGCTTTTAGGTTTTCTCTGATTAGGATGTTCACATTTTTAGTTGGTGGATGCTTATTTGTAGGCCAGCGCATTGGGGACCTTTTCTACACCAGCTGCAGCAGCTTGCGGCAAACTCCACAGTGTTGAGGAGCTTGAAGCACGTCACTCCTGAGTCCCTTTTGCCTTTGGATTTCAATTCCACTACTGGAAATGGGATTAAGCTTCTGTCTTCTGACTGGAAAACAACACCTACGCTGGAACTCAATGCTGTCATGGCTGACTCTTGCGCAGTTCAGTCTCGTCACGATAGTTGGTCTTTGCTTTGGCTTCCTATTGATCTTATCCTTGAGGATGCAATGGATGGCAATCACGTCGCAGAAGCTAGTGCTGTTGAAGCACTCACTGGTATGCAGAAATTTCACTGTCGTAACATGGTTACCAGACTTGACTGTTTTCATAAGATTTTTTTTGTTTTTGATGCATGATTGAATTGCTTCGTTGAAGTGGTTGTTGTTGGCTTATTGCAGGGTTGGTTAAGGCTTTGCAAGCAGTTAATGGTACTGCATGGCACAGTGCTTTTTTAGGCTTATGGATTGCAGCTCTACGGCTAGTTCAAAGGGTAAGAACTGATGCAAGGGGAAATTAATTTGTTGTAAGTGGTGATTTCTTTTCATGTGGGAAAAGGGGAATTCCTGAAAGAAAATGCAGATTAGTGATTTCAGATTGTGACGAACTCTTTTTTTTGTGTGTGTGTGTGAGAGAGAAATTGCTTGATGAACTTAGGCATGTGTAAGATGTAGTCACCCAAAAACATAACATATTGCAGCATAGCTATTCTATTTTTATTATGTTTGTAGGTTTGCATTTAACTGATTTACTGGTTCATTACTAAATCCAATCGGTGACTCTAATTTTGGATGCTATTGGACTCTATTCACTTGTTTTAAGTACATTTTCTCCCCCTTTTTATAGGAGAGGGATCCAGGAGAGGGGCCTGTACCTCGCCTTGATACCTGCTTGTCTATGTTATTGTGCATTACAACCCTTGTAGTTGCTAACCTTATTGAAGAAGAGGAAGGGAAACTTATTGAAGAAGCTGAACGTAGCCCTGCAAATCAAAGGATGGACAAGCAGGCTTTGGGAGAGCGTCATGGGGCATTGGTTACCAGCTTACAGCTATTGGGGGACTATGAGAATTTACTCACTCCTCCTCAATCTGTTATTTGGGGAGCCAATCAGGCTGCTGCCAAGGCTACCTTGTTTGTATCTGGACATAGTGGATACTTGGAACATACGAATGTGAATGACTTGCCCACAAACTGTTGTAAGTAATTGCTTTTATAATATGAATCTCTAATGGTGTTTGGTTTTAGAAAGAACTCTAAGTTGGAGGTCCTAAAATAAAAATAAATGGGCAGTTTTAAGAACAATTAAAAACACTTGTGTCCAATTGTATCTTCATACAGTTATAACTAAAATATACAGAAATTTCATGATAAGAAAAAATTGACAATATGGTCCATAATAAGAGCCTGCTTTCATTTATCCTGTTAATTGTTCCTTAATGAATTTCAGGGGCATACAACTTGACCTCTCTCTCTCTCTCCCTACATCTATCTATCTATCATGCTCATCATAATTTAAAACATCAACAGTTTTTTGTTTCCATTTAAGCTTGGTCCTATGGGATTTAATCATATTTTTGTTGTCAATGGTAAATGATCTATTTTTCTGTTTTCTTCTGTTTATTTTTCTCTCAGATGTGTTGGTCTCATACGGGTATTTTCATGTTTGTCTTGGGCCCCTGATTATACCTCTTTGGTTTGTGAGTGACAGTTCATCATCTTGTGTTTTCTTGCAGCTGGTAACTTAAGGCATCTCATTGTTGAGGCTTGTATTGCAAGACATCTGCTTGATACCTCAGCGTATTTCTGGCATGGTTATGTGAGCACACCTTTCAATCAGCTGCCTCATAGTATTCCTAACCATTTACCTAGCTGGTCATCATTGATGAAGGGATCACCACTAACTCCTCCATTGGTTAATGTCTTAGTTGCAACTCCTGCTTCTAGGTATGCTGCTATGTCATGTATCATTTTCTGCTTTCATACATTACATGTGCGTTGTGAGAGTTGCTATTTCCACATGGCAAAGCAGCAATGACAAAACTAAAAAGTTATTATCTTGTCTAGTTTTTCTATTTCACTTTTCTGTTTCTTCCTCATCAGTTTCACTATAACAATCTACTTCCTACTACAGTATTTATCCCGCTTCAATTTATACTACTCAACATCAAGTGCTGTACTTATGGGGAAATCTTTGCTGTTTTGGTTGTGGTTGCATTATCATGAAATTTAATAATTTATTCATCCTGAACTGAAGTGGGGTTGAAATATTTTATATAAAAAAAGTTGAATCTTTAAAATATATTATGTTATTAGAAAAATGATAATAAGTGAACGTGATATGCAAAATACATAGTTATGGGTTTATTACTCCCATCCCATGTATCTTGGATAAAGGGGCTTATTTCCACAACAACAACAACGCCTTATCCCACTAGGTGGGGTCGGCTACATGGATCAACTTCCGCCATAATGTTCTATCAAGTACCATACTTCTATCCAAATCATTAAGTGCGAGATCCTTTTTGATAACCTCTCTTATAGTCTTTTTGGGTCTTTCTCTGCCTCGAATTGTTTGTCTTCTCTCCATCTGGTCTACTCTCCTCACTACAGAGTCTACCGGTCTTCTCTCTACATGCCCAAACCACCTAAGTCTATTTTCCACCATCTTCTCTACAATAGGCGCTACTCCAACCCTCTCTCTAATAGCTTCGTTTCTAATTTTATCCTGTCGAGTCTTACCACACATCCACCGCAACATCCTCATCTCCGCTACACCTACTTTATTCTCATGTTGGCTCTTGACCGCCCAATATTCTGTTCCGTACAAAATCGCCGGTCTTACCGCAGTCCGATAAAACTTTCCCTTTAGCTTGATCGGTACCTTTGCATCACATAACACCCCCGATGCTTTTCTCCATTTCATCCATCCTGCTTGAATGCGATGATTCACATCCCCTTCAATTTCCCCATCATCCTGTATTACAGACCCAAGATATTTAAACCGTGTGACTTGAGGGATAATATGGTCTCCTATTTTCACCTCTGAGTTAGAAACCCTCCTTCTTTTGTTGAACTTACATTCCATATACTCCGATTTGCTTCTGCTTAGGCGAAAATCCTGCCTAGGTTAGCTTTGCTATTCTTAGCCGGTCCCAAGCCCGGATAAAAGGAGAGGGTTGTGTTAGGCTTTCGACAGCCAACGTTAAACTTTGTCGAATCTCTATAATATGGATCAATTACGTAAAAAAGGGGCTTATTTCCATTTAAAGAAAATAGAAAATTACTTGTTCCTGAGTCAAAGTAGAAATGAATATGTAGATATTAAATAGAGACACACAATCAAAGTATGCATGAATGTCTGTGTTTGAAAGTGGTTGCTGTCTAATTGGCTGTAGTATTGCTTAATACCTGGGCTTCAGTTACACTTTTCTGTTTCCGGAAGTCAGGTATATCAGATGCTCTATTTTGTATATTTACTAGTGGTAATGCAACTGAATTTAAGCTCGTGGATGTAATGGAAGCTTAGCAGAGATTGAGAAAATATTTGAATTTGCAATCAATGGCTCGGATGAAGAAAAGATATCTGCTGCTACCATTCTTTGTGGGGCATCTTTAGTACGTGGTTGGAATGTGCAGGTGATCCCAACACCATTTTAATTTGATTTTCTTCATAATTAGAAAATCATACTAGACTATTTTTTTTTTATTCATTTTGTTTGCCTCCCCTTCTTTTTCCATTTTTTGTTTTTTACTTTCATGATTATAATGATTATCATTGTTTGCAGGAACACATAGTTTTTTTCATCATAAATATGCTTTCACCTCCAGTTCCTCCTAAATATTCCGGGACAGAAAGTTATTTGATTAGCCATGCTCCTTTTTTGAATGTCTTTCTGGTGGGAATTTCATCTGTAGACAGTGTTCAGATATTCTCCCTACATGGCGTGGTATGTTCAGATTTAATGTGTATTCTGATTCTTACATGTGCATAGCAAGTGGTTTACAATCTTTTTTACTCAAAAGAAAATGTTTTTGAGAACATCCCTCCCCAATACACATAGACAAGGAAAATTGTGTATTATTCAATTTTAAGGTACTATCAATCAAAACCTATTATAATATTTTCTTTTCAGAGAAGGGAATGGTTGATAACTACTAGAATTTGGACTTAGTGCCTAACTTAACCTCACAAAACTGGGTTGTAAGATGAGGACTGGTGAAGCATTATAAGAACTATATTGACCATATCTCTAATCAATGAGATTTCAACACCCCCCCTCAAATGCAAGACTAGACATATAGAGAACATGGATAAGTGTGGGTGGCCCAATGACAACATCCTCTCACGCCTCAGAATAGACATTTTGAATGGAGACAAATATGGGTGGCCCAATGACGGGTTTAGGATAGGTTCTGATATCATCATATAATATGGGCCTATGGCCACTTAAGTCAAACCCCACGAAACTGACTTGTAAGGTGAGAATTGTCCAAGTCTTATAAAAACTACATTGACCATATCTGTAGTTGATGCGTGCCCCTTCACACCTAGACTGGACATCTAAAGCTTGGGCAAATGATGGTGGCCCTATAACACCCCAAATGCCTAGGAATAGACATCTAGAGTGTAGACAAATATGGGTGACCCAATAACAAATTTAGGATATGCTCCGATACCATTTTAGAATTTAGGATTAGGGCCTAACTCAACCCCATAAAACCGTGTTGTGTTGTGAGATGGTCAATGTTGGACCTCAATAATAACTATTCCTGCTTTTAGTTTGTTGGGTGATTCCTACACAGCTTTTAGTTTGTTGGCTGATTCTATGCATTTACTGTCTTTCAGGTTCCACTACTTGCAGCTGTCTTAATGCCAATATGCGAAGCTTTTGGATCATCTGTTCCTAATGTCTCATGGACTGCTGTAACTGGTGAAAAACTCACTTGTCATGCAGTGTTCTCTAATGCATTTATTCTCCTGCTGAGATTATGGCGGTTTGATCGTCCACCTGTTGAACATGTGATGGGGGGTGCAGCAACTCCAGCATTAGGATCACAATTAGGTCCTGAGTACCTTTTATTGGTTCGGAATTGTATGTTAGCAGCCTTTGGGAAATCACCAAGAGATCGAGTAAGGAGTAGAAGATTTTCAAAAATGATAAGATTTTCTCTCGAGCCTTTATTTATGGATTCCTTTCCTAAATTGAACATTTGGTATCGGCAACATCAAGAATGTATTGCATCCATCTGCAATACTCTTGCACCTGGAGGGCCAGTTTCTCAGATCGTTGAAGCACTACTTACCATGATGTGCAAGAAAATAAATCGAAGTGCTCAGTCATTGACACCTACAACTTCAGGAAGCAGCAATTCATCTCTTCCTTCATTGGATGATGCTTTGATGAAACTCAAAGTACCTGCATGGGACATCCTTGAAGCAACTCCATTTGTTCTTGATGCTGCTCTCACTGCTTGTGCTCATGGAAGTCTCTCTCCCCGTGAATTGGCTACAGGTTTACCCAGTTTACTCATGGATATATTCAAGCCTTATTAAAATAGATTTAATCATTATGTATCATTGAGAACTATTTCAGGATTATACTGAGGCTTTCATGTTTTAAGATGTGAGACTGTCAGCTGTGAAATAAAGATGTCCTGTGAAAAGATGAAACAAAATTGAACATGACCTGTGATTCTTGGAATAACTTAGCAAGTGAAGTGACTGTGGGTGTGTGTGTGTGTGTATATATATATAATAAAAAATGATATTATACTACCAAAATCTCATGTAATTGTCTTCTCATTCACTTGATTATCCTTGTTCATTTGTCAGTGAAATAAATACAGTATTTGCTAGGAGTTAGGAAGATAAAGATAGTTAGGACAGTTATTGTAATTAGTTAGTTAGAGGCAATTTGCAAGGTAAATGAGGAGAGGAGGATCCAAGAGAAGAGGCATTCTATCTTGCATGTCTAACTAAAATTGAGGAGGATGTAAGAATAGAGGTAGCTATCTTTTTAGGTAGGTATATAGAAGAGATACTCCAAAAGATAATATTGCATGTCTAACTAATGATTGTATGGTCCCTAGAGGAAAAATAGTAAATTAGTAAATTCCAATTCCAATACTAGACACTTAGGTGGCATCTTGTCACTCCCAGTCATTTTTAGCTTCAAATCAAATTATGAATCATCATTATCATCACCTTCATCCTCCTCAGTTTTAGTTTTCAAGATCCTTAAGCTTCCTCTCTTCTTTGTTTAGACTGGACACTGCTTTGTCGTAATAGTTTTTTGGCCATGGACAAGGACAATTGGTTCAAGATCTTTTTTTTTAGAACTTGCATTCTCAAATAAGTAGTGTTTTGACCTAGCTTTTTTTAGAGATTCTCTTTTCCTTAAAAGGAGAGGTTGAGGTAAACATAATTTTTGAGTTCAAAAATAAAAGGAGCCTTTTTTTTAATGAAGAAAAATAAATAAAAGATGGAGCTTTCACATGAGTTTCTGCTTCTCTATTTTGAAAACATACCTCCCTCCTTGTCCATGTTTGTCACAGCTCCTCATTGGTGATCTCTTGTTCAACCCTCCCCCAATTTCTATTCCTTCAAAGTTGTGATCTACTACTATTTCCTCAACAATATGCATGCCCTTGTTTCATTCTATCAGTCTCGGCCACAATCCTATCATCCTCAAAGCTTGCTCTTAGTTAAGTGATGTTTTCCTGGGTATCAAGCTCCATATGCATTCTCAAAACCATGCCTCTCGAATCCCAATAGCTTTGTGCTCACTAGTCTTGAGGATGTGTACTCCAAGTATTGACATGTTTGTAGTGCATGCCAATTGTTTGACAAAATCACTAGTAATGTAGAAGTTTGCAATTAAGCTAAAAAACCAGAAAATTACCAAGCAACATTAGAAGTTTTTTATTTATTTTAAACTTCAACTTAGAAGTAGCTTCAAAGATAGAAGCTAGGCCAAATGCTAGGTAAGTCCATGCTTCTTTAAGTTGGCATATATTAAGCTTATTTGATTAAATGTATGTTAGTATAAAAATTGCAAGTTTGGGAATTCATTAAACTGGGTTGCCAATGTCTTAACTTTTAAGCTTTTCTAAGGCTGTTTGCTTAGGAAGTTATCTGCTTGATTTTTATCTTAGGTCTCAAAGATCTTGCTGATTTTCTTCCGGCAACTTTGGGTACCATTGTAAGCTACTTATCATCTGAAGTAACACGTTGCATATGGAAGCCTGCTTTTATGAATGGAACTGATTGGCCTAGCCCTGCTGCAAATTTATCTATTGTTGAGCAACAAATCAAGAAAATTCTAGCGGCCACAGGTGTTGATGTTCCTAGCCTCGCTATAGGTTTGTTAAGTATACTTTCTTTATCTGTGGAAATGTTAATTAAACAGAATAATTAAACAGAAATCCTCTTAGCTAAATCCCTCTTTGCAAGTTCATATTATCCTTTCCTGGGTGATTCTTGCAGCATTGTCATTTTCTCCCGATAGACCAACTAACTTGTCATGTTTTGCATATGCATATTTATTTCTGGAAGTAATTTGTGGGTGATGAATAAATTTTCAATTAGCTACCTGCTATATGTTGACATAGAAATATCAACTCACTGTCTCGAATTTTTGGCAAACCATGTTTTCATTTTCTATTTATATGGACATCACACATCAGGTTTATTAAATCAATCAGTTGACACTGCATTTTAACTGCTGTAAACAGATGGGAATGCTCCAGCTACACTTCCTTTGCCATTGGCGGCTCTTTTGAGTCTCACAATAACATATAAACTGGATAAATCTTGTGAGCGCTTTGTTATTTTGGCTGGCCCATCTTTGATTGCCCTTTCTTCTGGTTGCCCCTGGCCATGCATGCCCATTGTAGGTGCTTTGTGGGCTCAGAAGGTGAAGCGCTGGAGTGACTTTTTTGTATTCTCTGCTTCTGCAACTGTATTCCACCACAGCAGGGATGCCGTAGTTCAGCTTTTAAGAAGTTGCTTCGCCTCCACCCTTGGGCTTGGTTCTGCCTGCATTTATAACAATGGTGGTGTTGGCACCCTCCTTGGTCATGGCTTTGGTTCTCACTACTCTGGAGGGTTCACTCCGGTTGCTCCTGGGTTTCTCTACTTAAGAGTTTACAGGTCTATTAGAGATGTCATGTTCTTGACAGATGAAATTGTATCTCTTTTAATGCTTTCAGTTAGAGATATAGCAAATGGTGGGTTGCCCAAGGGTGAAGTGGAGAAGCTAAAGAAGACTAAGTATGGAATAAGATACGGACAGGTTTCTCTTGCTGCATCGATGACTCGCGTTAAGCACGCCGCTCTTCTTGGGGCTTCGATTCTTTGGATATCTGGTGGTTCAGGTTTGGTTCAATCTTTGATTACAGAAACTCTGCCTTCATGGTTTTTATCAGCCCAAGGGTTAGAGCAGGAAGGGGGAGAATCTGGAGTTGTGGTTGCTATGCTGAGAGGTTATGCACTTGCATGTTTTGCGGTACTTGGTGGGACTTTTGCCTGGGGTATCGACTCTTTGTCACCAGCATCAAAACGACGACCAAAGGTCCTTGAGATTCATTTAGAATTTCTTGCAAATGCACTAGATAGAAAAATATCTCTTCGCTGTGATTGTGCTACTTGGCGTGCATATGTGTCTGGGGTTATGAGCTTGATGGTGAGTTGCACGCCACTGTGGATTCAGGAACTTGATGTGGGTATATTGAAGAGAATGAGCAGCGGATTAAGACAGTTGAACGAAGAAGACTTGGCTCTGCGCCTATTAGAAATTAGAGGGACAAGCGTCATGGGTGAGGCAGCTGAAATGATCTGCCAAACTAGATTATAACCGAGCAGAACTTGGGAGCATGGTGCATATGTGATGATACCAAAGACCTTATGAAAAATGACATGACTTATTTTGCTACCTGCTACCATTTCTGTACATAGCAATGTAAATTTCATAATTTTGTATTACCCGTTGAATTCAGATCAGTTACGTTTAGGTAATAGTATAAACCTTTGATCATAATAAGTATTATAGCATTAGCAGATATTGTCTCCATGTGTACTTGTAGCGTTAACAGATTTCATCGTGGGTCATATGAGGATTTTGTTTTAATATTTTTTTCCTTC

>GmMED6-1

TTGTTTATGAAAAATATTTTTTCAAAAAAATACTTATTTTAAAATTATTTTATTTTTTTAAGTTTAAGCAAATCAATTCTTATTCTTTGTGTTTACACAGTGAAGCCTATGAGATTGTGATCTCCTTGGGTCATCAACTGATCATTGTTTTCGTTCATAGTTTTTACATATATTTATGCACGATGAATCGTTTCAAGAATATCGGGAAGCCCTATATGAGGGCTCTTTTAAGACACGGGAACAATTTGAATGTTCATAAAATATTCAGGAGGTGAAATTAGTAAAAGAAAAAAAAGTTGGGCCCAAATCTTGCGGGTTTTTTTTATTTGTTTCCCCTTTCAGTTTGAGTAGTTATATTGTGATGCAGTGAAGGTATTTGTATTTGTTCCCCAACCGAGTAAAAGAAAAGGCTGTGTCCGTTACTCGGGAGTGGAAGATGGCGACGGCGCCGGGAAATCAAATGTTGGAGGGCGGAGGAGGAGCGCCGCAGCCTCCGGGTACAGACATGACCGGAATATGCTTCCGGGATCAGCTCTGGCTCAATAGCTTCCCTCTGGATCGCAACTTGGTCTTCGATTACTTCGCACTCTCCCCTTTCTACGACTGGACCTGCAACAACGAGCAGCTCCGCATGCGCTCCGTTCACCCCCTCGACATCTCTCAACTCTCGTGAGTAAATAAAAAACCCTAATTTCCTCTCTTTTCGGTTTTGTGGATTGTAGTTTGAGTAATTTTCTGTTGTTCAGGAAAATGACGGGGACGGAGTATATGCTCAGCGAAGTTATGGAGCCGCACCTGTTTGTCATTCGCAAGCAGAAGAGAGATAGCCCCGACAAAGTTACACCTATGCTCGCGTATTACGTCCTCGATGGTTCCATTTACCAGGCACCGCAACTGTGCAATGTTTTTGCCGCCAGAATTGTGAGATCATGCTCTATTTATGTTACATTTCTTCATTGTTTTTAGGTTAGGTAGGGTTTTTTTTTTGTTTGGAATGATTTACTTGCATGAAACTTGAGATATGGTTGTTGGTGATTGTGTGATGTAGAACCATAGAAGATAAAAGCATGTTGCAGGGCTATTGTGTGTGTTATTTAGGGATTGTTATATTATTATAAGTAGAAACTTAAGAGATCTTTCATTATTTTGTGGGAATTTTATATATAAACTTGCTTGCATGAGACTTGAGAATCAGATATGGATGTTGGTGATCGTGTGATGTAGAACAGAGGAGGAGCATTTTGTTTAGGGATTATCCTGCCAAGAGGAGTCATATTATTCTTGGTATCTGTTAATTTAAGGAGTATTTCTGTTTGCCTATCCTACATTATTGTGAAAATTAGAGAAAAGCCTTTTCAATCCCGAAATTTAACATCTTGTTTTCTGTACCCAAACTGTATTAGGTTTTAACTGTTTTGAAATTTGAAGTGTTGAGGCTCACCATTTTGAATTTCCAGCTGCACTACATCAAGATATTTTATGATTAGTTTTTATACTTAAAAGGCTTTATTCCACAAACTTTGTGTTTTGCTTTTAGCCAGTGCTTTTTTATTATGGGAGAGAAGGTTTTGTTCAATATACTGGTTTTGTCATTGATTTTGCAGGGAAGGGCCCTTTATTACATACAAAAGGCTTTTACTACAGCTGCCTCAAAGTTGGAGAAGATTGGATATGGTAAGTGGAGATTATAGTTGTGTTTTATGTTTTGGAAAAGTAAGACACTGTTGCTCCCAGAAATGTTACAGAAACTGGATAGAGTTTGAAAAATGATGTTATGAAAGTTTTATAGCTGTAGTATTTTGTTCTTAATTTCTGATTAAGGCAAGCTGCTATACAGTCTTTGTAGCCTCCTTCCTTTTGTCCTTTTAAAGAGAATAACCTTTCAAAGTGGATAGTGAGATGGGCTAAATTTTGTTTTTTAATTGCTAGAAATCTTAGTCTCAGGTTTATGATATATCATTTAGGGGTGGACATGGTTTGTTTCGGGCTGGAAAACCTTACCAAACCATAAATGTGGTTTGGTTTTTAGAATTGAACTGATTTTAGTGGTTCACGAACTGGTTCATTTTCCATTAAGTTAGTTCAGTTTAGAAAGCAGTTTTAAAACGGGTTTGAAACCAGTTTGGTTCAAAACTGTTTTAAAACAGTTCAGAACCAGTTTTAACTCAAAGGTGGTGTTTAAACTCCCAAAGTGGTTTCTAGTTTCTAAACAGTTCAGAATTGGGTTTGAACCCCCAAACTGATTCAGCTTAGTTCAAATCCATGCACACTGCATTGTTCTTAATGGCTACTTCATACACTACTACTAGCGTACCCCATTGCCCAGAGGCTCTTCGCTATGCGAAGGTATGAGGGAGGGATATTGTACGTAGCCTTACCCTTGCATATGCAAAGAGGCTGTTTCCGGGGCTACTTCATACACTCCTCTTTAAAAATCAACTTAATGAGTCAATAGATTTTAATTATCATATACTAAACATCAAAATGTCAAGCTACACTTAAAGCATGTTACACTCATTTCCTCCCAGATTTCGTACTCTCTTTTATGCCCTTTATCAATAGTTAAAGACTTCATTAACTATTAATATAGCTTATGGAGGCGACCAACCTTGTAGTCTTGCCAGAACTTTTGGCATTTATTGTCTCCTTTGTCTACATTTCATCACTTTTATAATAGGTTCAGTTATTTCTGGTTTAATTCAGTTCGGTTCAATTTTCAGTTTTTATCATCCCTTGTATTATTTAAGCCATTTGTAATGTCAGTATGTCACTTTTGCAACTTGAAAACAGAAGGGAACTCTGCTGCTGTTATTTTACCATCTATTTTTTATACCCATTTTATTCCCCTTACACGTTGGTTCTGCCTGCTGTAAGCCATGTTTTTGGAGGTTTTTAAACACCGAACTGTTGCATTTCATATTTCTGTGCACATTAACTGAATTGTGATTCTTTTAGCTGGGTCAGCTGATTCTGAAAATGAGACTGCATTGTTGGAATCAAAGACTGCTAAGGAGACAATTGACATAAAGGAAGTTAAGCGAGTTGATCATATTCTTGCATCCTTGCAACGCAAGGTAAGTTGAAAAACATATCCTTCCCATGTTTCCCTATTAAACAATTGTGTGCACATTATTAAAATTACAACTATTTTTATATAAAAGAGAGGGGTACAGGGGTTGAACTATAAGTTGTATCTTGAATATCTGTGCACTCAGTTGTGAATACTGGGCTTCTTTACTATCTAAAAAATCTTCTTTCATCTTTTGAATAATTCTTTATTTTCCTATTAAATTATCCTAAACCTAATTATTTGAAACAGACTGCATTACATGAAAAACAGAAGCATTTAATATATTGAGAAGCCAAAATGCATTTAATAATTTGAATAATTAGGAGAAGTATTAACTTCACAAAGAACCAAGAGAATCGTCCGGATCATATTATTAGAGTCTTTTTTTCTTGCTTTTCAATAGGGGCTGGAGAAAAGTGCAATCAATAGGGTTTTCCTAATTATTTCTTCCCAAAAGGCCGAAGAACATGAAATTCTTTTTTCCTTTCCATAGGGATGAGATTGGATCTGGCCATAAGAAAAATATTTTGACTAATTAATAACTTGGATAATTTGCTAGGAGGATCTAAGATTATTTTTTTTCTCCTTGCTGTGTTGTCATAATTTTATTTTGCCGGTAGAAGCATGCTTTAAGAGATGTTGTCCTTTTTGCTCTTTTGTGATGATGCACAAGAGGTTTTTCCTAACAGGCCCAAGTCCCCAATTGAACAAATAGTTGTGTTTTGATTGATAAATATGAACTGTTCACATTATTGGAATTTTATGACATTCTTCTCTCAACTTTTAAGCTCTCATCCTTTATTATTGGTGAGAAATGAGTATTATCTTGCTGCTGCAGTTACCACCAGCTCCTCCTCCACCACCATTTCCAGAAGGTTATGTTCCACCTCCAACTGCAGAAACGGAGAAAGGCACTGAAACTCAAGAAGCAGCAGAAACGCAAGCTCCTACTGCTGATCCGATAATTGATCAAGGACCAGCAAAAAGAATGAAATTTTGATATAAAAGACATACTGCAACGGCTATTAATACTTATACAGGTGGAGGAAAATTATATTATTCCTTAACAAGCGGCCGTTGCTAAAACATGATTAACTTAATGTTATTCTCTGGTCTAAAATGCGTTGCTCTTTTCCTGTCTTCACCATAAATGTAGATATTATCACTGAAATAGGATTAAGTTCTCCTGTTTTTTCATTCTAGACAATGTATTGAGCAATTGTAACTTGTAAGGGTAAACGAAACTAGTGTCCTTTTCGCATTGAACATTTTCTGTATTTTACGTTGACGCAGGGACTTGCATTGATCCTAGTTTTCCCTGAACACCTCGGGAAATTGAATTGCTGATCAATTGAGTGTATAAATATTGCGTACGTCAACTAGGGAATAGATTGAATTGTCTTATAGATTCTTTATTCTTT

>GmMED7-1

TTTCTCTCTCTCTGACTCGAAATTTTGCAGATCTGGAAATGGCGACGGCAACGTACCCTCCACCGCCACCTTATTACAGACTTTACAAAGATTACTTGCAAAACCCTAAGTCTGCTCCCGACCCTCCACCGCCTATTGAAGGCACTTATCTTTGTTTTGGAGCCAATTACACCGTATGTATCACAATCCATGCTTCTTCTAATTAGGGTTTCTTCTTATTAACCTTTTTTCTTCTTTCTTCATATCATAGACTAGTGATGTTCTACCAACCTTGGAAGAACAAGGGGTGCGCCAGCTCTATTCTAAGGGCCCTAATGTTGGTAAGCTTTAACCTTTCAATGCTTCTGCTGATCCTTGCTTCCTAATGTTGGTAAGCTTTGTCTGCTCTTTGTTGCCATAGGTTCGGTATTTTATATTATCATTATTATGTTGCAAAATACTCGGTCACATTTTCATCCTTTTTTGGAAATTCCATTATGGGAAAGTGGAATCACTGAGGCACGAACCTAGAACAGAAACAAATCAATTACAGCTTTGCATGCTCTGCTTTTTCAAATTGGGTATTCATTCCCTTACTTTGTAGGTTGGTATATTACATGTTGAGCTACTGTTGTGATATTGCTCGTTACTCCAGAATGATATATACAACTTACATCCATGTCATAAAGTGAGTGATTTCAAAAGGGTCCAGTGTCCATGATATATTTTCTCTTTTATTATAAGTTGCTTTAGCATAACCATTTCAAGATTCTTTAGTTTTTCTTTTATGTTCCATCCTTTAGTAAAAGATTCTGATCTTGTATAGATTTCAAGAAGGAGCTGAGGTCACTCAACGGAGAGTTGCAACTACACGTTTTGGAGCTTGCTGATATTCTTATTGAGAGACCTTCACAGTATGCAAGGAGAGTAGAAGAAATTTCAACAGTATTCAAAAACTTACACCACCTTTTAAATTCATTGCGTCCTCATCAGGTATAGTATGCATTCTTCCATACAGCTAACTCTTAGGAAATATGCTTGCCTTTGACTGGCAGGCTGTTGTTCTCTTTCCGTCATGTGAGGTTTTAAGTGTATCTTGTATTTACTCTGTAGGCGAGAGCAACATTGATTCATATTTTGGAACTTCAGATACAGCGCCGTAAACAAGCAGTGGAGGACATAAAGAGGTATATTTTATATTTCCTTATTGTTTTCTTAATTTCATATGAACCCTAAGGAATCTAGTGCTCTGGTACCATGATAGTGAAATGTCTCTGTTCTATCAATTTGAGAGCCTTTAACAATTTAGTTGGTTTCTATGGGTGGTAATTGCACCCATTTATCCACTAACCATCCAATTTATCCAATTTCTAGGGGATCAACAATTACTATTGGATGGTTAATAAACCATCAGCCATGTTTTTTAGACCAATGGGTCAAGATATTGGATCAATGATTGTCATAAAGACAGGTTTTTATGGAACAATGCTCATAAGGAGAGAAATATTAAAGTTGTGTTGATAAGTAGAAGAGTATAAAAAGTGAAACTTACAAGTATTTTTGAATGGATGGATTGATTTCAGGGAGAGAAGAGTTCCTGTTTTTCATCAATATTCTAAATGGAATATATTTCATTATAAATTTAAAATTTGATCCTTCAATTTTTAATCACAGAAGGATGCCCTATTAATTGATTGATTTCCTTTGATAACATATGCGACACCTTACAAATAGCCCATAACTTCTTAGAGTTCTCCCTCCTAGGTTCATGTACCCATACCGGCTAACCTTAATAACTGCACTATACCTCTTCAAACGAATTTTAATATACTTAGTAACATCATGCTGTATCCCTTTTAAGTTTCATGCTTTCAGCAATGTAAACTTTACCTTCTTGAAACCATGGAAGACCACAAGAAAGTTGGAACTGTTAGTAATGTTGTTGAAACTGATAATGCAGACATCATCTGGCATTCCTGTGATAATTTCTGTTCCCAAACATTTTCCAATGAAGTACATAGTCATTTTCTGGGGTGTTGGTGGTGGTGGTGGGTCAGGCACTCAAGTCTAATTGATTAATTTTTCTACAGTCCCAATGAAGCAGCACTTGCATTGTGACTACTTTTTTGTGTAAAAGATGTGATTTTGGTCTCCCGCCGCCAAACCAAACAAGTGTTTCATTATCCATTTTATACTGATAACCAGAATTCATAGCATCTCACATTTTTCTTTTTTTACAACCTTCTTATTTCTAAAATTTTGGGACTTACAAGACTATTCTCAATGTATCTTGGTTAAGTTCCATGAGCTATGGGTGAAAAAATGGCTGTGATGCTTTTGAAAATTGATTGGTCTGAATTGTAAGATTTTATAGTTGGTGCACTAAATATGAGGCAAAAGCTCAAATGGGGAAGGAACCATAATTTCTTAACATTTATTAGGTAGTGTTGGCTTTTGGTATTATTTGTGTGGCTAGGAGATAGGTTACCAGATCTTTTACTACCTTCATTTCCCTTGCTTATCTAGTTTTGCTTTGGTGAAAATTAAGCAGCTATTTTTCTTTTATTAAATACTAATGTGTGATTGGGACGGTATTTTATTGTGTTCTTGTCAGAGTAAAACACTATTTTGTTTCCTAAAAGCATTGGGGTCAGTCATGTGAGTCTGTAAAAGATGAAAATCACCTTTCTGTTCTGAAAGATGAAAAGGGCCATCTAGTCCTTTTTAGGGACCAAATTAATCAATATTTTCATTTTTCAGGTTGAAAATGGTGATTTCTATTTTAACGGGACGAATGTGATCAACCCCAAATCTTCTAGGGACCAAAATGATGTTTGACTTGTTCTTGTCATTATTAGTCTTTTCTATTCTAACTTAATCTCTATTTGCAGGAGGAGAGAAGAAGCACGAAGGCTCCTTAACGAGTCCTTGGCAACACTTGATGGCCATTAGAGCATTCTATCTTGTATTCACAGCAAGAATCTACTGAGGAAAT

>GmMED7-2

TAAAAGAAGCCATAATTTGATTTGACACAGGAGGCACGTTGCTATGAGTTGCTCTTTCTCAATTTTGCAATTGCAAGTGAAGAAGCCCTAATTTTTTGCAGATCTGGAAATGGCGACGGCAACATACCCTCCACCGCCACCTTATTACAGGCTTTACAAAGATTACTTGCAAGACCCTAATTCTGCTCCCGACCCTCCACCACCTATTGAAGGCACTTATCTTTGTTTTGGAGCCAATTACACTGTATGAACCATTTTTATTCTTCTTCAAAAATAATTAGGGTTTCTTCTTCTTCGCTCTTATTAGCCTTTTTTCTTTTTTGTACATAGACTAGTGATGTTCTACCTACCTTGGAAGAACAAGGGGTGCGCCAGCTCTATTCTAAGGGCCCTAATGTTGGTAAGCTATTTTCATCTCTGCTTTAACCTTTCAATTCTCAGTCACATTTTCATCATTCTTTTGCAAATTTCAATAAGGCATAAACCCAGAACACAAACAAATCAATTACAACTTAGCATGCTTTGGTTTTTCAAATTTGGTATTGATGCCCTTACTTTGTAGGTTGGTATTGCATGTTGAACTACTGTTGTGATATTGTTCGTTGTAACTTGCATAAATTCTTATTTACATCCATGTCATAAAGTGAGTGATTTCTAAAAGGGTCCAGTGTTCATAATATATATTCACTTTTCATTATAAGTTGCTTTAGCATAACCATTACAAGATTCTTTAAAACAAATTTATCTTCCATCCTTTAGTGAAAGATTCTGATCTTGTATATTCTATAGATTTCAAGAAAGAGCTGAGGTCACTCAACGGAGAGTTGCAACTACATGTTTTGGAGCTTGCTGATATTCTTATTGAGAGGCCTTCACAGTATGCAAGAAGAGTAGAAGAAATTTCAACTGTATTCAAAAACTTACATCACCTTTTAAATTCATTGCGTCCTCATCAGGTATACTTTCTTCTATACAACTAACTATAAGGAGATATGCTTGCCTTTGACTGACAGGCTGTTGTTCTCTTTCCGTTGTGTGGGATTTTAATTGTATCTTGTATTTACCCTGCAGGCGAGAGCAACATTGATTCATATTTTGGAACTTCAGATACAGCGTCGTAAACAAGCAGTGGAAGACATAAAGAAGTATATTTTATATTTTACTTATTGTTTTCTTAATTGCATATGAACCCTAAGGAATCTATTGCTCTGGTACCAACTTACTGGAATGTCATTGTTCTATCAATTTGAGAGCCTTTAACAATTTAGTTGGTTTCTGGGGGTGGTAATTGCATCCATTTATCCACTAACCATCCAATTTGTACATGCATAAATTAAACCAATCTGTCCAATCGTAGGAATAGAACTTAATGGATTGGATCAACAATTATTATTGGATGGTTAATAAACTATAAGCCATATTTTTTAGACCAATGTGTCAAGATCATCAATGATGGTCATAAAGACCCGTTGCAGGTTTTTATGAAAACAATCCTCATAAGGAGAGAAATAATTAGATAGAGAAGTTTTGTTGATAAGTAGAAGAGTATAAAAAGTGAATGGATGGATTGATTTTAGGGAGAGAACCGATTCTGTTTATCACCAATATTCTGCCTTTGACAAGTTCTACATTAGAAGTAACTGAAATAGAATATATTTCATTATAAATTCAAAATTTACTGCTTCAATTTTTTAATCAATGAAGGATGCCCTATTAATAAATTGATTTCCTTTGATAACATATGTACACCTTACAAATAGCCCATAACTTCTGAGTTCTGACAGAGTTCTCCTTCCTAGGTTCATGTACTCATGCTCGCATACCTTAATAATTACGCCATACCTCTAGAATTGTATGTTCAATTGAATTTTATTTTAATGTACTTAGTAACATCATGTATCCCTTTTAAGTTTCATGCTTTCGGTTATGTAAACTTTACCTTCTTGAACCCATGGAAAATCTTCAAGGGACCAAAATGATGTTTAACTTGTTCTTGTCATTAGTCTTTTCTAACTTAGTCTCTATTTGCAGGAGGAGAGAAGAAGCCCGAAGGCTCCTTAACGAGTCCTTGGCAACACTTGATGGCCATTAGAGCATTCTCTATCTTGTATTCACAGCAAGAATCTACCGAGGAAATTAATTTTATTTGAAAAGTGAATTTTGTCCTCTAGATTTTAGCAAATGAAAAGATTAAAACAAGAAGTTGTGCGACTTCCTTGAAAATGGTCACTTGACATAACTGTCTCATGTTCTTGATGAAGGCTGTTTTGCAGCATGTGCAAAGATATTAGAAATAGAAGAATTGTGTTAAGAGTGTTGCTACTGTTCTTAGAAATATTTTTAGTAGTGTATAAAGTCTGAAGTCTGAACTATTAATGGATGGACCTGTCAGTCTGCATTATTATTATTATTATTATTATTATTATTATTATTATTATTATTATTATAGGATAGTGTTACGACGA

>GmMED8-1

TGGCTGGTCCGTATCTGCACTAAAACTCAGCGCTTACCCTTTTTTTCCTAAGTCCTAACCTTCATCTTCTTGACCAAAACACAATAGCGGCCACATACCTCCTTGTCTCGCGGACCACCAAACCTCCGCGCCCCTATCAACCAACAAATCCGTCGCTCCTAGCCACGAATCCAACGAACAACCTGCGACCCCTAACTTTCCGCAAACCCAAGACGCACCACCTACGACCAAACACCGCGCACCCTCGTTGATCCGCATATCTACGAAGAGAAGAGAGGTTGAGCAATGGAGGGGGGCGAGGGAGGAGAGGGGTTGAACCAGGCAGTGCAGCAGCAGCTAAATCTGAAGCAAGTGAAGACCCGCGCTATCAGCCTCTTCAAAGCCATCTCTCGTATTCTTGAAGATTTCGAGGCCTACGGTCGCACCAACTCTACCCCCAAATGGCAAGACATTCTGGGTCAATATTCTATGGTCAACCTTGAGCTTTTCAACATCGTCGATGACATCAAGAAGGTGTCCAAGGCCTTTCTGGTCCATCCCAAGAATGTTAATGCCGACAATGCTACCATACTCCCTGTCATGCTTTCCTCCAAACTACTCCCTGAAATGGAGACCGACGACACCGCTAAGAGAGATCAATTGCTTCTGGGGATGCAAAACCTCCCAATTCCAACGCAAATTGAGAAGCTCAAGGTAATTATTAATGAATGAGCTTTGAAATTTGAATTTCGAATTGCTTCATCCACGAGTCATGACCTATTAACCAACCTAATATGCTTCAGGCCCGTCTTGATCTGATTTCCGCAGCCTGTGAAGGTGCTGAAAAAGTGTTGGCTGACACTCGCAAAGCCTACTGCTTCGGAACTCGTCAAGGCCCAGCCGCCATTGCCCCGGCCACTCTAGACAAGGGTCAGGCTGCCAAAATTCAAGAGCAAGAAAATCTACTCCGTTCTGCTGTCAATGCCGGTGACGGATTACGGATACCAGGAGACCAGAGGCACATCACTCCTGCACAACCTCCATTGCATTTAGCAGATGCACTTCCTGTCGTCATTGCCAATGACCCTACTACTGCACAACCGCAACCACAACTCTCAGCAAACACCATGGGCATGCCTGCCCAGAATTCCTTGTTACAGGCCTCCTCCGCAACCGTATCACAACTTTTGGGAAGATCAGCAGCATCTCCTTCTGCTGCAACCACCACCACCACTTCTTTTGACAATATAACAGCTTCCCCAATCCCATATGCCAATTCACCTAGGTCTTCTACAAACATCATGAACTCTCCATCCCCTCAGCAACAACAAACCCAACAACAGCCACCAGTGCTGCAGCAGCAGCAGCAACGACAGAAATTCATGCAATTACCTCAACAGCAGCAGCAGCACCAAATTCTTGCTCAGCAGCAACAGTTCAGGCAGTCTGCAATGCAAGGACTGGGACAGGTAATATTTTCAAGTTATGTATTAGTGCTATTTGTTGTATCTTTCTATTCTCTTTTTTGGGGGTAACAAGCTTGCTTTCATCATCAGTTGCACGGACAACATCAGATGCAATTTTCTCAACCACTTGGGCACCAGCAATTTCAGGGTAGGCAGCTGCCTTCAGGACATGTTCAGCATGGCATTGGTCAAAGCCAACTCAATCAAGGAAACCAAATGACTCGATTAAGCCAGTTTTCTGGTCCTGCTAACAGTGCGCTATTCAGTGCTGCTCAAACAACACCTAATACTCAAATGGTATGGTATTCATGTTTACAATTTATTCAAATAGACAGGCTCTTTTACTATGACTATATATATATATATATATATATATATATATATATATATATATATATATATATATATATATATTCATGGACAGCTAACTGCAGAAGGAAGTTATAACTAATTTGTTGAATTACTTTGACTATCTGTTGGTGCTGTATTGCTTTCCTCTTGTCCTCCTCAGTCCTTAGTACTTCTTTGAAGGGAAAGCCAACCTCATAAGTAATTAAATAAACTAAAAGGAAGGAAGACAATATTTTGGAGTCCATATCATATAGCACCTGAACTCACTTTTACTTGTATTTTCTTTACCTTTTTCTTGCAGTAAATTTCATCAGTAACTCCATTTTTTGTCCTTTGTGTTTGTTTTTTTTTTTATCGGCAAAAATAAATTACATTTATATATATAATCAGTACCAGCGGTACTATCAGTACAAGTAGTTAGACCACCAGCCACATGGTTCCAAAATCAGACTAAGGTCAGTCCCAAAGGGAACTTGTGTGTTAATATTGTTTGTTTTTGTTTCTTGCAGACCTCTTTTTTTTGTTCCTCCTTTTTGTGGGGGTGGGGTGTGGGGGAGTTAGGGGGGTTGAGTTTATCTTATTTATTTTTTAGTCCTGTGGAGGGCTACCTACTATTTGAACTCTGTATTGCTGCCAGTATATAGAGTAGGTATGGCTTTACAAATGCTTTAACACTCGAGCATTATTTTGCGTTGCAAAAAAAAAAAAAAACACTGGAGAGTCTGTTAGTCTGACAGCAATAAAATCATTCTTTGTTTTAATTGAAATGGAGCTTTCTGAACATAGCACAGTTCTTTTGAGATCAATTTTAACTGTATTCCTGGATTTGATTTATGTAATTTGGGATGTGATTTCTTGTCTTTCCGTTTGAAAAGATTATTTTTCTGGATCTTTGATGATGTGCTGTTGCTGTGTTTCGTTGTGCTACCCTTACTGTTAATGTGCTTTGCTTCAACAATTACTTCTGTGATGATTTTTGCTTCTTAACCTCAGATTCCAAACATTTCCGGCACATTACCTTCACAGTCCCTTCTCCCACGGGTGCAGGTTTGTCATTGATGTTCTCTGCCTATTGGGTTATCCCGATATGTCTCATTGGTTTTATTGAGGTATAAATTGTCAGATTTGAATGAAACAAGAAAGAAGTCAAAGGAATTTTTGTTGACTGATCTGTCTATGGTGTTCTGGGCATGTTTTTGAACTCCACTATTTAGTCCTAGTAAAGTTTAGTCACTTTTTTGTTTGACTCACTCCATTGACATTATACTTCCGGATGATTCAGTCTATCTGTACATTGTTGCAACTATAATAGAAACTAATAGGTGACTTTTAGGGGCCATTCTGATTTGCAAAATTAAGAAATTGATATTTTAATTTTGGCCACTTTTTATTGTATTTTATTTTTTTGAAATTTTATGGCATAATTTTCATTATGACATCTTTCTGTAAGGACCGGCTGGACTACCCTGTGACTCACTTTAAGTTGGTCATTACTTGAACAAAATAATTTCTATCATGCATTTGGTTCATCATCTATATAGACTGAATAGTTGTCTCCGCTTGCTTTTGGTTAACTTTAAATCATGAGGATAACATATTCAAACTTACTATACATTTATTATTTCCTCAGTTAGGATTATCTGGAAACAACCCTCAGCGAAGTCATCCTTCCCAAATGTTGAGTGATCAGGGTATGAACTTTATCCTGAATATCTTTATCTTTTCATTTGTTAGTTGGGGATCATTGTCTGTGTAGTTTGTCCTCCTTGAAAGGGTTGTGATGCAGCCTGGTGTGTCATTTATTCCATCTTCTATATGTGTGTGACTTATTACTGAATCATCACTGTGGAGCTTACCTTGCAGTGTTTAACATGGGAGGTGGCAATCCTGGTGGTATGATGCCCATACAACAGCAGCAGCAGCAGCAGCAGCAGCAACAACATGGTTCACAAGCATTTGGTAGCATGGCAAATGCTCAGAATCTACAATCTGGGTTGGTGACACTTCAAAACACACAACAGAATCACCCCAATTTCTCTCAGCAGAGACAGCAAAATCCACAGTGATTAGGACTCGGCATGGATGGGGTTGGTTGAACCATGTTGCTGTAGAAACATGGTTCACTAAAACTGAACCAAACTTTTAGTTTAATTATTTTTTGGGTTACCAAATTATTTTTAATTTTGTAAAAAAAAACTGAACTGAAAAAAAAAACTGGTTAGTCCAGTTCGGTTTGTGGGTTCATTGGATTTATACGGCAGGCTTGTATAATTACAATATTGGAAACCTATGTTAGAATTTAGAAAATAGTTTGTGAAGAGCGTAAAATGGCTGGTTGATTCAGAACTATTTGTAGGGGCGATAATCGAAAACCACGGCATCGTCGTTCATTGAGTCTTACTATTACCAAACCCAAACATATGA

>GmMED9-1

AAACATACGGATCTTAAACCGGAAAATCCTTTCTCCACCGTGTTTTGCTTCGCTCCACGCTCAGACCTTGGTTGAGTTCTTTCCATTTGTTTCTCTCGTGTATCGTGCTGTGCTTTATTCTCTGCCTCTCTCAATCGTTCTACTTCGCTTCGCTCTCTCACTCTCTCTGTGAATTCCATGAAGCCTAATGCTTTTATTCTTTTACTTTTACTTTTTTTTTCTAATCTTTTCCCTAATTTTGTATCTCTGACTCTGTGTGCAGGTTTGATTCTTCTCCCTGATTTCGCTGTAAATAGCTGAATCTAGCTTCTCTCAATATCTAAGGTAATGATAGTTTGAATAGCCGAATTGAATTCCCGAGTTTTGGTGTCATATATTAATTTCCCTTTGTTGACATTGGTAGTATGACATATTATAATATTAGGACAATCTGTAGTTGTTTAAGGGCCCCTTTGTGTTCACATTTTTTTAGTTGATATATTAATCTATGGTAGGATATAGAATCTGAACCTGAAGAAGGAGTTAATCGTTTTGCAGCAGCAGTATAATTGTAGTGAGTGAGATGGATCATTATGGTTCTTCTGGAGGAAGCTGGACGATGATCCCAACCCACAATTCAAGTATTCAATCCCAATCCCAATCGAACCAGGACCCTAACCTCTTTCTTCAACAACAACAACAACAGTTTCTCCAGTCACAACCTTTTCAGCAAGCACTACCACCCCAATCTCCATTTCAACAGCACCACCACCTTTATCAGCAGCAGCAACAACAACAACAACAGCAGCGTCTTCTACCGCAACCGCAACAGCAACAGCAACAGCAGCAACAACAACAACAGCCGCAGAACCTTCACCAGTCACTGGCCTCTCACTACCATCTTTTACATGTAATGAGACCATTTTCAATATATTCTTTTATTTTTCTTTTCAATAAAGGAATTTCTTGTTATGGGCACAGTTGGTGGAGAATTTGGCTGAGGTTATCGAACATGGAACCCCGGATCAGCAGTCAGATGCATTGGTTTGAATTTTCCTCTTTTCACTGTTTTGATTAATCCATTTTTTTTGGAATCAATTTTTCCTTTTAGGGTTTTCAATAATTTTTTTATTTTGTTTTTTATTTTTTGATTTCATTGGTTAAAGATCAATGAATTGAGCAACCACTTTGAGAAGTGCCAGCAGCTGTTAAACTCAATCTCTGACTCCATTAGCACCAAGGCTATGGTAATCTGCAATTCTCTAACTTTACTCTTTTCTTGTTTTATATGCTAATTGTTAAGTAGGATAGGCAAGTTAGTTTAACACTTGAACTTCAAACTTCTGTGTTTCTAAAGTCTGCTGAAACTGAATGGCTCTAGTTTTGCCTTTAATCCAATGCACATCATGCACTTTCTTTCTCTCTCATTCTCTCTTTATACTCCTACTATCTTTTGTTATGTGGGCCATGCATGATAAACAAAAATCCACATTATTGATGTTGAAAATCTGCTGACATACATCTCAATATGCTTGTGCTCAAAACATCTTTAACTCGTAGTTTGAAGCTAGGGTTATACTTGGCCTTACGTGAGTGCTTTCTGGATGCTGAATGCAGACAGTTGAGGGACAGAAGAAGAAGCTAGAGGAAAGTGAGCAATTGTTAAATCAGCGAAGGTCTGTAGTTCTGTACAAATTGTATCAGATATTTCTGTCTAGTTAAGTTTCTAGATGCTAAATAAATGTGTTTGTTTTCGCTTGTTCCAGAGACTTGATTGCCAATTACAGAAAATCTGTGGAGGATCTTGTTAGGTCTGAGCCATAAGAGAATGTCGTGCATTCAGATAGGTAGGGAACCTTAAATGGGGTTTTGTTTCTTGATAGACTGTAGCCTTCATGTTTTACTAGGAATGGATCACCAGTTCTCTACCTTAAAATTAAGATTAGCAGAAATGCTAATAATTATTTATACTTATAACTGATAATTACTTAAAGATGTATGTTTCAATACATGTTCTATTATCTGCGGATATTAAAGGATGAATGTGTATAGCTATAGGAGAATTTTAATATCTTTTACTGCTTTTGGTTCTTTTTTAATTTGGAACGTGGAAGTCCCAGTTACAGAATCCAATTTTGAGTCATACTTGCATGTATTTTGGGTTGGATTAGTCACAGGAAATCTCGAGCCAAAGATTTAAAAGCTGTAGATGAGATTAATCAATAAACTTTGAATTCTCTTTGAGGAACCCCATTTCATTGAGCACTTGAGTCTCAATTGTAATTTGTGCTTTGGTTTGAGAAAAGATCACGATTTCTTTGTTTTTATATCTTTTAATTGATTGAACAATTCCAGTCTTGTTTTTTTAAGTTTTTCACTCCTAGCACAATGAAGGGAACAAATTTGTTAGGCACTCGGGCCGTATATTGTCCTTTTCATCAATTCCAAACCACAAAAGATGCAGACTACAGACATTCTCAACTAAATTCTGTGCCACCATCTCAATTGCTTTCTAGTCTTGTTTATTTATGTGAATATTATACTGTTGCTGGACTACTGGCTGAAGCTAATCACCTGCTTAGTTGTAAAGAGAAAAATAATTTTAGTATCTCAAACAGATAGTCTGTAAAGAATGTAGTTGTATTTCCTTTTTAGATAACAAGGAACGTAGGTGTGATTAAGAACATTTAATCAATTCCAAAATACACTTGATGCAAGAGAGAAATTGAGTGTACTAAATATCACTTTCTTCCATTTATGATCCTGATGCTTGTCCTTGGTTTTGATTCAATGTTCATTATGTATGTCAGTTAAACTTGAAGCTTTTTGTGACCTTAAGTTTTGATCTTGTATTACAGATGTCACTGTAGACGTTTGCTTTGAATTTCTCCCTTCTCAGAACATCTTGGGATTATTATGTACAACTACCTTGGAGATTTATGCCTTGAATTTATAATTCAATAGATATTCTGCAAGTTACTGGCCATGTTTTGGATCCTATCAAGGTTTCTATATCCATGAAACAAGGTCAAAGTAGGTTTAAATCAACTGCACATCTTTACTGTTCTCTTCTGTTGCCTCTATTAGAGAGGCAGGACATTAAATTAAAGAGCATACTCTGGCAATTTGGAGGAATGTAATGTTTCCGATAGCTTATGTAAAAATGGTTGGCCCCCTGCATTGTATTTGGGTCATTTATCTATCATGTGTTGAGTCAAAGTTTGGTGATATTGAATGCATAATGTCATTCATTACACTAACCCAAATTGTTTAAAGAACATTGAATGTTGTCTCTAGACCCAAAAGAATGAAAAGTGAAAGATAAAATGCTGCTGTCGCAACT

>GmMED9-2

AATACTATAATTTTGTAAGAACTAGGCATGGTCTCAGTCATTCTCGGCCTACCGCCGCCACAAAATGATCTCAATTTATGGGTGGATAAAATTCTACGGCAATTAATTGCTTCCTTCTTTGGTTTCGATTCTGAGCCTAAGACAGAAGCCAATCTATTCTCTAAGAAAAAAATTGAGGAAAATTTAGAGTTCACTAGTACACATCAAGTTGAAGTTCCAAAGAAAGAAGGAATCAAACCTGGAATTAGAAAAAAAAAAGGAGATGCAAGCGATGAATGAAAACTGAAACCTTAAAATGATATGAGAAGAGGGAAAGAGAATAGAATGGAAGCAGTGAATCTATAGTCTCTGGGATGTTTCCCTTAACAAATAAGGGGGCACTGTATGTTTGATAACCGGGGCAAAAACGTCCAATTTGCCAAATCCGCACCGGCCCAATCCAGTTTAATCTGATCCACATTTATTACCCGTTTAAGGATCCAAAATCCATTTTAACATACGGATTTTGGAAAACTAGGGAAAAATGTATCTGCTCCCGTGGCATTGTAGGGACTAGGGATTGTTTCACAGAACACCTCTGAAATTCAAATCCCTTTTTTTTGTTTGATATTAATCTATGGTAGGATATAGAATCTGAAGCTGAAAAAAGGGGTTAATTGTTTTGCAGAATCAGTATAATTGTAGAGAGCGAGATGGATCACTATGGTTCTTCTGGAGGAAGCTGGACAATGATCCCAACCCACAATTCCCAATCCCAATCGAACCAGGACCCTAACCTCTTTCTGCAACAACAACAACAGTTTCTCCAGTCACAACCTTTTCAGCAAGCACTAGCGCCTCAATCTCCATTTCAACAGCAGCAGCAGCAGCGTCTTCTACAGCAACAGCAACAACAACAACAGCCGCAGAACCTTCACCAGTCACTGGCCTCTCACTACCATCTCTTACACGTAATGTAACCCTTTTCAATACTTTAGTCCCATCTACTTATTCTTTTATTTTTCTTTTCAATAAAGGAATTGCTTGTTATGGGCACAGTTGGTGGAGAATTTGGCTGAGGTTATCGAACATGGAACCCCGGATCAGCCGTCTGATGCATTGGTTTGAATTTTCCTCTTTTCACTCTTTTGATTAATCCATTTTTTTTATTCAATTTTTCTTTTTAGGGTTTTCAATATTTTTTTGTTTTTATTGTTTGATTTCATTGGTTGAAGATCAATGAATCGAGCAACCACTTTGAGAAGTGCCTGCAGCTGCTAAACTCAATCTCTGGCTCCATTAGCACCAAGGCTATGGTAATCTGCAATTCTGTCTGACTTTACTCTTCTCCTGTTTATATATATATATATATATTAATTGTTAAATAGGATAGGCAAGATAGTTGAACACTTGAACATCAAACTTCTGTGTTTCTATAGTCTGCTGAAACTGAATGGCTCTACTTTGGTTTTGCCTTTAATCAAATGAACATTCTGCACTCTCTCTCTATAGTCTATACTCCGACTATCTTTTGCTACGTGGGCCATGCATGATAAACTAAAATCCACATTAATGATGTTGAAAATCTGCTGTCATACATCTCTATATGCTTGTGGTAAAAAACATCTTTAACTCGTAGTTTGAAGCTAGGGTTATTCTTGGCCTTACGTGAGTGCTTTCTGGATGCTGAATGCAGACAGTTGAGGGACAGAAGAAGAAGCTAGAGGAAAGTGAGCAATTGCTAAATCAGCGAAGGTCTGTATTTCTGTACAAATTCTATCAGATATTTCTGTCTAGTTAAGTTTCTAGATGCTAAATAAATGTGTTTGTTTTCCCATGTTCCAGAGACTTGATTGGCAATTACAGAAAATCTGTGGAGGATCTTGTTAGGTCTGAGCCAGTAGAGAATGTTGTGCTTTAGATAGGTAGGCACCCTTAAATGGGGTTTTATTTCTTAATAGACTGTAGCCTTCATGTTTTACTAGGAGTGGATCACCAGTTTTCTATCTTAAAATTAAGATTAGCAGAAATGCTAATAATTATTTATACTTATACCTGATAATTAGTTAAAGATGTATGTTTCAATACATGTTCTATTATCTGCCG

>GmMED10-1

TTTGCTGCTGCGGTTTAGTAGTGCAGCTAAACGTCGACGAACAAGGAAGGAATAACGAAAGAGCCTTCAACGCTGATTTTTGTAGAATATTGGACCGACAAATAGTTACCAGCTTCCACCCTGTCTCTCTGCGATTGAGAAAGGGACTCACGTTCTCTGTTCATCACACCCTCTCTCTCTCTCTCTTCGTCTCTCTCTCTCATCCATGTGCATTTCTTGTCTCATATTAGGGTTTGTTGATACAATTTTGATTTTATTAATTTGTGCTTCGATTTCTATTTCATTGATTAGTATTTCGATTTTGGTTTTAATTTCAATTTGTGCTTCCATTTGGATCTCTAAATTATCTGGACTGAATCATACACGCGCTGTTACGCAGCTTCGAGTTCAGAGGTCGCCCCCTTCATAGCTTAGAAGCAACTTCCGCAACGTTTTCTCCTTGATTTCTTCAGGTACCATATGCTTTTTTTAATAATAAAAAAACATTTTTGCTTCTCAATCCTAAGAGGTTCCATTTTCCTTACTCATCAATATAACATGGTTACGAAGTAGGATCTTAGGCATGTCAGTGTTGGAAACCCCAATAGAACGGAAGAACTTGAGTTTAGGCAAAAGGGTTCTTGAGAAGATCAAGAACAACATTTGGGCGACCTGGGTTTTTCAAATTGTCCCTGTTGAAGTGTATTGAGGACACCCCACATGAGTTGATGAGGCAAGACGCTGTAAAGGTGTCACTTTTAAGATGGTTTCTATCAGAATGATGAATGAGTTGAAGAGAAAAAGCGAGGCATTGTGTTTGCATTGCAGCAAAGAACCCAATACGATAAATGGGTAGTATTAGTGTAGTAACTTGAGAATGATGATCTTCCGATGAGAAAAGTAGGCACCATTTAGTCTCTTACATTGAGATTATTGCGTTGCACACTGTTTTTGGTCGGCTAGTTGTTGAAACCGCTGTGGGGTTTGGACAAAAGGATCTGTCTCGTGAAGTCTGGCCAAAGGGTTTGTTCAATTTCCATCTCATGCAATTTTTAACTTGGTTTATGAGCCTTCTGCAAACTGACTGATTCTGATATATTAGCATTTATTCTATGACGTTGGGAATTCCATTCTTTTATACTTCACTTGTTAACATGTACAGGCTGGGAAATAGGGTAAAAATATTCGCTGGTAATCAGGGTTTCAAATAGCGTGCTATAGTGCTGTAGCGGAGTAGCCCTTGCCTGCTACAGGGGCTGCTGTAGTGGAACAGTTTAGTGAAGCGGCTGTAGCAGACAAAAATAGCGGGCTGAATACCGGCCGTAGCAGATGCTATAGCAGCGCCATGTGTTGATTAATTCCAAAGAAAACCCTCTAAAAAACCGAGGTTAGGGAAAATGAAACCTACTGCTATATTTGTAGGGAAAATGATTAAGACTACTTGATTTTTAGAAGATGATGATTGACGACTCTTATTTAGGAAACTTGTATTTGACTTTTTTTAGTATTTACATGTATGTTTTATTTAAGACACCTATGATTTTTTATTGTTAATTATGAATGTCATGAATTTTGAGCAATTTTTATCATTTTTGAGAATTTATACGTATATAATTATATATTGTACATTATATTAAGTATAAGCTACATAAAAAAAAATTCAGAATAGCGGCTATCCTCTATCCCACTATATTGGTTTTTTGACCAGCTGCTACATGCCGCTACTCAAGATTGAAAACATTGCTGGTAATTCTAGAACATGTATAGTTTTTTTTCCAGGTTTTAGAATGCCATTGTATTATTATTATTATTATTATTGAGCATGTTTGGTATATAGATTACTTTTTGAGAAATAATCGCCAAAAGTTCTCTTGTGAATGATTGTTCCTCCCGAATAAATTGAACCACCACGCACTAAGATCATATTGTTAAACAATCATTCTTTTGGATAGAATATAGTTATTGCAGATGCAGTAATGCTCTGCAAGCCGCAATAATTGATAGTAAACTCTAAAGGGCTAAGAGGGCAATTAAGTTTTTTATTTAGTTGTTAACAACTAACAAGCATCTTCATTACTTACAAGTTTTTTCCAATCCTTCATCTCTTTTTCACTTTCTCTCACAGCTCTAGTTCTCTCTCCCAAGCTTTCTCCAGCTTTGCAACTAGCTGTGTCTGGCTCTGGCCATCATCAAGTCTTCTTCAGGTAAGCGAGAATCCATAGGTCTCACTTATATTTTTGTTCAGTTTAATGCAACTTACTGCTTTTAGTTTTGCCATTTTAATTTAATTTTGAATTTCAGGGACTAATTCCTAATATTCTATAATATCTATGAAAATTGGTTTCTGAATCTAAAATGGAGATTTACTCGATTCTTTCCTTTATTTTGAAATTGTTTGTACCTAATTCTCTTTACTAATGTAAGGGACTTGTCTTTTGTTTGTGTTGCGAATGTGATTGCCACACTAACTGAAAGAAATGAAAATCCTAAGGGGTGTTAGTGTAACTCCATGTTACCTTTGAACTGTTTATAACATGTCTTGCCTGATTATGAGGGCTTTGATTTTTTTGGAAAGTTTTGTGCCTGTTAATTTGAGATTATATTCAATATTAGTGCATCATGTCCAACATTACTACAACATTTTCCTTCATTTCATGTAGTTGATGACAGTACCAATTTGCCAATGGTCTTATACATGTATTAGAACTTAGAAGAACCTCGTGCATGCTTTTAATTTTTTAAATATCTACAGTAGTTCCTTATAATTTACTTTCAAGAAAATTGCTATATAGTCTCCAGGCTACCTCACATTGCTCTTTAGCCCCTTACTTTTATTGTTAGCCTTTTGCATTTTGAATTGAAGTTTTTTCATAATTTTTTGTTAATAGAAAATTACAAATGGATTCATCACAAAGTGCAGCTCTTGGGGGAAATGGTGGGAGTGGTGGAAATGGGACGTTGATTTCTCAAAATAATGACATGGCAGCCTCTGCAACAGGAGCTGATGATTCTATGCAGAAGCTGAACCAGGTCAGCAATTCCATTCAGAAAACCTTAGGCCTTATCCATCAGCTTTACCTCACAGTTTCTACCTTCAATGCTGCCTTTCAAATGCCTCTCCTCCAACGCATGTATTTCTCTCTAATCCTTTTTCTGCAATGCATTTTTTCTGTTTTACTTTTGCTTCATGTTTAAAATTGATCATATTTTACTTTAAATGTCCCAGCAATGGCCTTGTTGCGGAGCTTGACAACATGGTTAAATTGGCAGAGAAGTGCAACATTCAGGTTCCTATGGAGGTTGTCAAGTGAGTTGTTTGCAATTGCCTTTGTGTGCTCCTATAGTTTCTTATTACCTAATTTAAGTTTTACTTTTTAATTTTTCTCATTTGAATTTAATTGTTGGCTTTAGTTTAATTGATGATGGAAAGAATCCAGACGAGTTTACCAAAGATGTTATAAATAGCTGTATTGTAAAGAATCAGATCACCAAAGGAAAAACTGATGCTTTAAAGGTATATTCTTATTCTTGATTTTCGTTACATATGAATTTCTTTGTTTCCAGTTTTGCCTTCCCTTATATTTAACTTAGTGTTTCTGTGTGGGCAGAGTTTGCGCAAACATCTTTTGGAGGAATTGGAGCATAACTTCCCTGATGAGGTTGAAACTTTTAGAGAGAGTCGTGCTGCTGCAGCTGCTGTAAGTACTATTACTTATTTGGTCTTTTAATTCAATGTGGAACAGAGCTTTAAAAAGACCCTGCTATGTCATTGCTTTGCTCCCAGTTCATTGTAGGATCATACGATCTCATGATTTCTGTATTTTAACTATAAAACTCCTCTTTCCTTAACTGATAACAGAGACAAACAAGCAATAACAATCCAGAGAGGAAAAATACAGATATCACTAGCGAAACTGAAATTGAAAGAAACTACAGAAAAAAGGCCAATGCAGCTCTTAGAGGGTTTACTAGCATCCCACACTTTCCCTCTCTACTTCTAATAATTTTCTCTATAGCAGATCACACCTTCCTCAATCCTCCTTTCCTATGATAATTCTGTTATTGGTGAATCCATACTATCCACGCTGGCATTTTATATAACAATTGTTCTACATGCCTGACAAGTTCAGTTTTGATGCTCACCTTCAATTCAAGTACTACCAAGGAGATACCAAATGATACAACCAAGAGAGAACATAATACACATTATTTCTGAATGCTTTCTACCAATTATTATTTATTAGGTTGTTTGATAATCCTCATCTAATTCTTTTCCATTCATATTAGGATTCTTAAAAACAACCAATCCTACTCAATTTAAATACCTTGGGTTGGTTTCTGCTGCACTATTTCCACCGTATAACACATGGTTAATTTTGTAGTACTTTTGTAAATGGCAGCTTGTCAAGGGACTATTTATATTTCTAAATATTTTGAAAGGGCTTAGGGGAAAAAAACCGAATACTAAACTGAATCCTTGAGTGAATTACAAGAATTATATAATTTTGTTAGTTTACATAAATCTTGGCCTTATTTCATCCATGCAGGAGTTGAAACGTCAGGCACAGGCACAAAGCGCACTGCCAAATGGAGATGTAAGGGTTAAATCAGAGCATTGAGTGATAGGCATATTTCTTGTGCCTTCTAACGTTTATGTATACGTTGTATAGTGCAGTTCAGCACTGTTCCTTGTTTCTGCATCTTGGTTAAATGTTGTAAATTTGATGACTATTTTTTACTTATTAATCACTTAATTTGGCTTGTTGCTAATTTTTGAGTGCATATTTCAATGAGCTTGAGGTGCAGGGGAAGTGGAAATTAATTGGTGTTATTAGTTTTCTTTAACACTGGTAAATTTCAAAGAAAGAAAATCAATCGTTCCAAAATAAATGTAATTTTCTGCATTTTGAATTAGATTTCCC

>GmMED10-2

GTGTTCCGCAGGTGAGGAAGGATTTCCCCGACTTCACCTTAGCTATCGCCGAAAACCCGTACCTTCATATAAACATATATATATCTGATACTCTCACGTTTCACGCGTCCTTCTCCCACCGCACCCCACACTTCCGGCACCGTCGGAACTCAGCACCGCACATCTGCACCCCACACTCGTCTTCTCACAACCGTCAATCGCAGTCACAGCGCACAGGTCCATCTTAGTTTTATCCCTTTTAGGGTTCTAAACTAATACCGCTTTGGCTATTTTTATGAGTGATCTCAACTGTAATTCGTTGTTCCTATATTCTAAGCCAAAGATAACGTTTCTGAGTTTCGCAATTTGTATTTCGCTTTTTTATTTCATTGGTTAGTTTTTCAATTTTGGTTTATTTAATTTGCGCTTCGATATCTATTTCATTGATTAGTGTTCCAATTTTCATTTTAATTTGTGCTTCCATTTGGATCTCTAAATTATCTGAACTGAATCATACACGCGTTGTTACTCAGCTTCGAGTTCAGAGGTCCCCCCCTTCACTGCAACGTTTTCACCTTGATTTCTTCAGGTACCATATGCTTTTTTAATAAATAAAAAAAAAACATTTTTGTTTCTCAATCGTAAGAGGTTCCATTTTCCCTACTCATCAATATACGTAGGATCTTAGGCATGCCGGTGTTGGAAACCCTAATAGAACGGAAGGTGTTCTTGATAAGATCAAGAACAACATTTGGGCGACCTGGGTTTTTCAAATTGACCCTGTTGGAGAGTATTAACTATTGAGGACACCCCACATGAGTTGATGACGTAAGACACAGTAAAGGTGTCACCTTTGTGATAGTTTCTATCAGAATGATGATTGAGTTGAAGAGAAAAAATGAGGCATTGTGTTGTGTTGCAGCCAAGAACCCAATAGGATATACGGGTAGTGTAGTAACATGAGAATGATGATCTTGCAATGAGAAAAGTAGGAACCATTTACTCCCTTACATTGAGATTATTGCATTGTACACTGTTTTTGTTGGTTAGTTGTTGAAATTGCTATGGGGTTTGAACAAAAAGATCTGTCTCGTGAGGTCTGGCCAAAGGGTTTGTTCAATTTCCACCTCATGCAATTTTTAACACGGTTTATGAGCCTTCTGCAAACTGACTGGTTCTGATATATTAGCATTTAATGTTGGGACTTGCATTCTTTTATACTTCACTTTGTTAACATGTGCAGGCCGGGAAATAGTGTAAAAACACTTGTTGGTAATCAGGGTTTTAAATAGCGTGCTATAGCCCTTGCTTGCTATAGGGGCTGCTGTAGCGGAACAGTTTACTGTAGTGGACTAAATAGCGGGCTGAATAGCAGCCGTAGCGGATGCTATAACAGTGCTATATGTTGATTCCAAAACCCCCCCTCTAAAAAAACAGAGGTTAGGGAAAATGATTAATAAGACTTGAATTTAGAAGATGATGATTGATGATTCTTATTTAGGAAACTTGTATTTGACTTTTGTCAGTATTTACCTGTATGTTTTATTTAAGACACCTCTGTTTTTTTTTATTGTTAATTATGAATGTCATGAATTTTGAGGGCGAGCCCTGGTGCAGCGGTAAAGTTGTGCCTTGGTGACTTGTTGGTCATGGGTTCGAATCCGGAAACAGCCTCTTTGCATATGCAAGGGTAAGGCTGCGTACAACATCCCTCCCCCATACCTTCGCATAGCGAAGAGCCTCTGGGCAATGGGGTACGAAAAAGAAAAAGAAAGATGAATGTCATGAATTTTGAGTAATTTTTATCATTTTTGAGAATTAATATGTATATAATTATATAGTATACATTATATTAACTATAAGCTACATAAAAAAATTCAGAATAGCGGCGGTTATCCTCTATCCTGCTATATTGGTTTTTCGGTCGGCTGCTATGTGCCGCTACTCAAGATTCAAAACATTCTGGTAATTCTAGAACATGTAAAGTTTTTTTTTCCAGGTTTTAGAATGCCATTGTATTATTATTATTATTGTTGAGCATGTTTGGTGTATAGATTACTTTTTGAGAAATAATCATAAAAAATTCTCTTGTGAAGTTATAAACAAATGATTGTTTCTCCCGAATAAATTGAACCACTGTGCACTAAGATCATATTGTTAAACAATCATTCTTTTGGATAGAATATAGATATTGCAGATACAGTCATGCCCTCAGATTTTAGGTAATTTTATATGAAAATGCATAAAAAATTAAAGTTCTGGAGTGTGGGTTGGGAAATGAGAGGAATGATACATTGTTCTTAATAATTGCAGCCTTGTTAGCTACCTGCCCTCTTTAAGCTGCAATAGTAAACTCTAAAGAGGGAAAATAAGTTTTTAATTAGTTGTAACAAGCATCTTCATTACTTACAAATTTTTTCCAATCCTTCATCTCTCTTTCACTTTGTCTCACAGCTCTAGTTCTCTCTCACAAGCTTTCTCCAGCTTTGCAACTAGCTTTGTCTGACTCTGGCATATATTTTTGTTCTTCAGGTAAGCGAGAATCCATTGGTCTCACATATATTTTTGTTCAGTTTAATGCAACTTACTGCTTTGTGTTTCACCATTTTAATTTAATTTGAATTTCAGGGACTAATTCCTAATATCCCATATCTATGAAAATTGGTTTTTGAATCTAAAATGGAGATTTACTCGATTCTTTCCTTTATTTTGAAATTGTTTGTGCCTAATTATCTTTACTAATGCATGGGACTTGTCTCTTGATTGTTTTGTGATTGCCACACTAACTGAAAGAAATGAAAACCCGAGGGGGGTGTCAGTGTAACTCCATGTTACCTTTGAACTGTTTATAACATCATTAACATGTCTTGCCTGATGATGAGGGCTTTGATTTTTTTGAGAAGTTTTATGCCTGTTAATTTGAGATTATATTCAATATTAGTGCATCATGTCCAACATTACTACAACATTTTCCTTCATTTTGTGTAGTTGATGACAGTACGATTTGCCAATGGACTTATACTTTCAAGAAAATTGCTATATAGTCTCCAGACTAGCTCACATTGCTCTTAGCCCCTTATTTTTTATTGTTAGCTTTTTGCATTTTGAATTGAAGTTTTTCATAATTTTGTGTTCATAGAAAAATACTAATGGATTCATCACAAAGTGCGGCTCTTGGGGGAAATGGTGGGAGTGGTGGAAATGGGACGTTGATTTCTCAAATTAATGACATGGCAGCCTCTGCAACAGGAGCTGATGATTCTATGCAGAAGCTGAACCAGGTCAGCAACTCCATTCAGAAAACCTTGGGCCTTATCCATCAGCTTTACCTCACAGTTTCTACCTTCAATGCTGCCTTTCAAATGCCTCTCCTCCAACGCATGTATTTCTCTCTAATCCTTTTCCTGTAATGCATTTTTTCTTTGTTTTACTTTTGCTTCATGTTCAAAATTGATCATATTTTACTTTAAATGTCCCAGCAATGGCCTTGTTGCGGAGCTTGACAACATGGTTAAATTGGCAGAGAAGTGCAACATTCAGGTTCCTATGGAGGTTGTCAAGTGAGTTGTTTGGCTTCGTGTGCTCCTATAGTTTTCTTTTGACCTAATGTAAGTTTTTCTTTTTAATTTTTCTCATTTGAATTTAATTGTTGGCTTTAGTTTAATTGATGATGGAAAGAATCCAGACGAATTTACCAAAGATGTTATAAACAGCTGTATTGCAAAGAATCAGATCACCAAAGGAAAAACTGATGCTTTAAAGGTCTAATCTTATTCTTGATTTTCATTAGATATGAATTTCTTTGTTTCCAGTTTTGTCTTTCCCATATATTTAATAAGAAGCTTTAGTCCAACTTAGCATTTCTGTTTGGGCAGAGTTTGCGCAAACATCTTTTGGAGGAATTGGAGCAGAACTTCCCTGATGAGGTTGAAACTTTTAGAGAGAGTCGTACTGCTGCAGCTGCTGTAAGTACCATTGCTTATTTGGTCTTTTATTTCAATGTGGAACAGAGCTTTAAAAAGACCCTGCTATTTTAGGAGGATCGTATGATCTCTTGATTTCTTTATTTTAACTATACAACTCTTCTTTCCTTAAATGATAATGGAGACTAGCAAGCAATATCAATCCAGAGAGAAGAAAAATACAGATATCACTAGAGAAACTGAAATTGAAAGAAACTACTGAAAAAGGACAATGCAGCTCTTAAAGGGTTTACTAGCATCCCACACTTTCTCCTCACCTTCCCTGTTTCCACTTCTAATAATTTCCTCTAGCAGGTCACGCCTTCCTCAATTCCCTTTCTTGTGATAATTCTGTTATTGGTGGATCCGAACTATCCACGTTGGCATTTTATGACCATTGGTTGTTCTACATGCCTGACAAGTTCACTTTTGATGCTACCAAATTATACAACCAAGAGAGAACTTAATACACATTATTTCTGAATGCTTTCTACCAATTGTCACTGTATATCACATGGTTAATTTAATAGTACCATTGTAAATGGCAGCTTGTCAAGGGATCAAGGGACTACTTATATTGTTAAACTTGGGCTTGGGGGAAACTCAAAGACAACTGAATCCATGAGTAAATAACAAGAATTATATGTTTTTGTTAGTTAACATAAATCTTGGCCTTATTTCATCCATGCAGGAGTTGAAACGTCAGGCACAAAGCGCACTGCCAAATGGAGATGTGAGAGTTAAATCAGAGCATTGAGTGATATATTTCTTGTGTCTTCTAGCGTTTATGTATGCACTGTATAGTGGAGTTCAGCACTGTTCCTTGTTTCTGCATGTTGGTTAAATGCTGTAAATTTGATGACTAATTTTTGCTTACTATTAATTACTTAATTTGGCTTGTTGCTATTTTTTGAGTGTGCATGTTTCAATGAGAGGTTGAGGTGCCGGGGAAGTGG

>GmMED11-1

GCAGCTTGTGTAAAGAAGAATGGAGGAGGAAGGTGACATATACGACGGTGCTCCAGCTCAGTTCCCTATCTCATTCGGGAAGCAATCGAAGCCTCAAACCCCTCTTGAAGCCATCCACACTGCGACACGTCGTTCCAATTCCAATCCCAAAACCGCCAACGACCTTCCTTCTATCTCCTCCTCTTCCAGGGAATGCCTCTTGGTTGATCATGACTTCAAATTCTTGGTTAATGTCTTCCAATTGAAACGCTACACTCTCATTCTTCCTAATGAACTTGATGCAAAGTTCACATAAAGATTTTTCATGCTCGATCTGATCTCTTTATCCATATTATCATTAGTTGAAGTCATCTAACAAATTTATCAAGAAAGTATGATGAATGAATTGAAGTAAAAAACATAAAATAAAACTGAATAAAACTGAAATAATGTTAATGAGAATGATTTATTTTATTTTTTTAGGGCTGAATTTGGTCTTTCTTGATATATACAAGTGTAGTATGTTTGGGTGGTTGGACATGAGATGAGAGTGAATGTAGAGACAATAGCCTAAGATTTTTTTTGAGATGTTAGTTATTTTTCTTATGAATTGTACGGTGTCATGTTACTTTTAAAATAACTGTTTATTATTTTAATAAACTATATTTTGAGATTTTCAATTATTTTTTTTATTTTTCTTCCTTCACCCCCACCCTCTCTCCTCTCTCCTCTTGTTTTTTTTTTTTTTTGCTTTCTTTCTCCGGCCCAACCCTCTTCTCCCTCCCTTTTTTGGTTCCAGGTCCTCACCCTCCCCCCACCCTTCCTTCTTCCGCCACCCACCTCCCCTCTCCTCGCGCTGCTCCCCTCTCCGCGTGCTGCTCTCCTCTCTGCGCGCTCCTCCGTTCGTTCGAAGACGGTGACGTTCCTCTCCAGATCTGTTTATTTTTTTCGTTTTTAATCGTTCATGATTTGAGTCTGTGTTAGTGTTGTTGTGTATCTGGTTGTTCTTCTCCTCCTCAAATCTCCATTTTATTTTCTTCCTGTTGCTAGCAGTTGCTTCCCGCTGGAATGTTGTGGAGAGAGAGAGAGAGGAAAATATCAATTTTAAAAAAAAAATTGTTGCAGATGTTCTTGTAGAAGAAGTGGATCTCCACGTGGAGATCCAACAAAATGCTCCAATAATGGATCTTAGCAAGATCAGATCTTGAGGCTAAGATCTACTTGCAAGAACCCTCATTGGAGATTGCCTAGTAACCAATTACAAGCCAGAAACCAGTTTTGCTTTTTGACTTCGATTTTGTTCTTATGTAACCACTAACCAACCTTTAGAATATTGGCAGTTTCTTAGACAAATAGCTTCCACTGAAAGAGAGAAACTGAGTGACGTATGTATATTAGTGATGTTTGCATCTTCTCTGATTACATCAAAATGAATTGTGGATGTTTTCTATCTTACAATTTTTTCTACAAAATGAACTTGGCTTGGTATTTGTTATCTGTTTGCAGATGTGAATCTCTGCTTTTAGTTCATATAATTTGTGCTCAATTATATAGATTGAATTATTTACAATAAAACTAATAATTTGATGAATTGTTAGATTTTTATTATTAGTCTAAAAAGCCCAAAGACATACCGGGTTGGCCCTGTCCCTAGGCCTAATATCCAGCTTTAGTTTTTTTTAATTGACAACTTGGCCCATGAGTTGGCATTGGCCCTTTCCCTACGTGGCTTTACGGGCTTTTGCTCACAGTTTTTTTATCTAGTGGGCTTTTTGGCCCTGACCCATATGCACTAAGGCTGGGCTTGTCTTGATGGGCCATATTGAGCGCTCTATTTACTAATAAATTCTTACCATCTTATGCATCTCAACTCTATTGTTGCTACTATGTGATTATGAAATTGTTAGTTGTGCATTTGATGGTTATCCAGTCTTAGATAATTTTTTTGTCTTCAGAATGATTTATAGCATAGACTTGAATTAGAGAAGGAACACAAGAAATCAACATCCATGGATTCACAGGGCCAGACAACTTCATTGCAGAGACTTCAGAATGTAGAAAAGGTAATATATTGAAACCATGTTAAAAAAAATGTCCTGAAAGTGACAGAATATCTAGTTTGTTCGATTGATTTTTTTTCTCCTTTCTGCTGGTCATGTGTAGAGAATTGTGAAGGTTTTGGAGCTTGCAGGAGGAGTCATGGATGAGCTTGCAAGCCCTGTTGGTCCTAGGAAGGATGTGGTCCAAAACCACTGCCTTGAGTTCATGCAATTAATCAAGGTCCTTGTCTCCCAGAAATGAATTTATTCTTTTGTCTATTTGTGTATATTTAGAGGGTGGCATTATGGGTGCAAGTCCCAAAAAGAGCGCACACTACCAAAACTGCATTAGTCTAATTTAAGTGGTCTCATTTAAATAATACCTAATGTTATATAAGATTAACAACAGCCATTGTGTAACCATATTTTATTTACTTTACATTAATCTTTTGTTAGGTACTGGCCCAGAAATTAGCAAAATCATGAAAGAAAAGATAGGATTTTATTGACGAAATATCCATTAGCCTTGAATCAGTATTAAGTACCCTGGTTAGGAAGAGAAAAATAAAAGTTGTTCTCTGATTTCAAGAACACGAGATCACTCCTTCATAATCAATCTCTAACTTAACAACATACCTCTAATCTTTCTCTCCCTATCTCATAATAGCCTATATTTGCCAAATTTCATTAGAATTTTAACTAAGCAATAACCCTAACTCCCTTAATTAGCAGAAATCCAGTCTCATTGACTTTTCTAACCTCTTGTTCTAACACCACGTACTTGCAGCCAGCATTTCTCTGAGCTGGAATTTGAACTCTGAATCTGTGTTATTGCCATACATAAATGTCTTAAATAATGATGATCGATCTAAACATGTGTGAACAACAAGTAGATCAGCCTATTACTTAAAGTTCATCATCATGTTAATGCTCAAACTTGTGCTGTAGTTTTGTAAATCTCTTTAATTTCATTTGTTTTCTTTTCAAATCTTTCAGGACATTCAGGTTGCATTGCGTGATGAAATCAAAAGTGCTTGTGAATATCGTCCATTTGAGAAATGTGATTATGGTTCAAGAATAGCCAATGAGATTTGTCACAAGAAAGTGGAATTTATTATGTCCCAGTTGGATGCTATAAAACAAACTGTAGATGAGTATCATGTAGCAGTTTGAAAATGTAGCTTAGTTCCCTGTAGAAGCCTGGGTTACCTCCCGGTAATTGACTTATTTGGGATTGCTTATTACTAGTTAGCATGTTGTTTTGGATGGGGGTAACAATTACCTTTGTAGAGATAGGAAATCAGTGCATTTATTGGATGATTATATAGAAGAATATCCCAGTTTCCTGCAAGAAGAAAATGGTTATCTTAGAAACATGTGAAAGTTAAGGGCATTTTTAATTTTAGATTTTTTTTCATTTTTTATTTTTACCTTCACTTT

>GmMED11-2

AAAATTTTAGAATTTGAAAAAGTAATTTACAACTACCTAAAAACACATAATTCTAAATCCCCTCAAAATAGTTCTGGATCGAAAGGCGAGGCGGACCTTCGGGCGTTGCTCGCTCTCGACACCAGCACTCTCTCTGTGGTTTGTCTTTCTTGCTCTCTCTCGTTTGCTAGGGTTTCTCCATTCAAATTTGGTTCTAAGGTTTCATGATCTTGATTTTATTATACTTTGATGACTATTTTAGTTCTCATTGGTTCTGGCTCTCAAGCTCTCTCTATCTCTCTCTCTCTCTGGTTTGTTTCTCTTGCTCTCATCTCCCCATTTCTTGTTAGGGTTTGTTCATTCAGATTTGGTCTTAAGGTTTCATGGTTTTGATTTTTTATTCTACTATATATTTTATGAGTTAACAGTTTTTAATTTAGGTTTCTGTTTGTTAGATATCTGAATTCCAGATAGATACATTTTGTGCATGCATGAGAGGGTTTTTCTTGAATTCTTTCTTAATTTGAGAAAGTTAAATGTTCAATCGAGATGGAAGAGTTCAAATTTCGAAGCTGGGTCATTTGGTTTGGGGACTAGTTACTTCATGAAGCAGTGTTGTAACTATGTTTGACTGTTTGTGGTAAGGCATTGGTCAAAATTGAATTGGAAATTGAAATTAGGTGTTTTTTGTCAATTTATTTCATAGAGACACGGGAATTATCTGTGGAACTGGATTGGTTAAAGTAGTGATTCACTATGGAGCTATTTTACTTGTTCATCGTGTTGTTTTTCAGTTCCATGTTTAGATTCTATTTGATAAGTTTGAGTCTCTTGCTGCATAACCTCATTTTTTTCATTGCTAATTTGTAGTCTAGTTAATGTATCTCACTGATGGTTCTGGTTGTCTAGTTAATGTATCTCACTGATGGTTCTGGTTGTTGTGTTACCCGTGGTAGTTTTGATCATTTGCTCGACCTTGAATTGAATTTATTGTGAAATGCTGATTGTATTTTACACTTGTAGTTTGACCTCCTTTTTTCACAGTATCCAATTTGGTTGTGCATCTATCTTTTTCAGTTGTTATTCTACTATGGTAAGAAATATCATAGTACTCACAATCGTAACTTCTAAGACAGCATTCAACTAAAGAGCCTTAACTCAAGTATACTATTTTTCTCTAATTAACAAACTATATTGAAATTTAAATGTTGTGTCTAATTAGAAAATGCGAATGAAGATTAGAAGATTCATTTGATTTTGTTAAACCATGTTTAAAATATGCATCTGAGATTTGAACAGTCAGATTTTCATCCAACGCCAATGACTCATGATTGTGTGAATGCATGAGTGTAGGAAATCCAGGTCAGGGGCCCTAGCATATAGCTTATAATTAGTTATTAAGTCAAATACCATTATTTTGGTGAAAACAGTTAACAAAATGCTCAGAATTTGTGGTTAATTTTTCTCTAAGCGTGACAAACAGACCATTAAGATTCTAGTATTTTACTAATTAAACATTAGGGATCCGTTAGGCTAATTATTATTATTATTATTATTATTATTATTTTTTTTTAATTCAAACTTGTCAGAAAATACTTTACTCTCTGATTGGTAGATATGGCCGTTTTTAAATTAATAAAGTATATTTTAGATAAAGTAAAAGGCTGCTCTCCCTGCAGCACTTCTCTCATTTCCTCTGCTCAGGTTAAGAGCTTAAAATATAGAGAACATGATGCATCATGCATGCATGGCCAAATTTCCTCTGCTACACGTCTCTCATTTCCTCTGCTCATTGTTTTTAATTCTTGATCAGTTTTTCTGCTGTGACTTGCACGCTATGATTGAAAATTAACTGCTGGCTATGGCTATTTTTTTCTTGAATATGCTGTAGTTTTGCTATGAGTTTGACTGAAAAATTAACCGCAGTTTAGAGTGAAGCCTGCTGGTGTATCAGATTATAACAATTTATAATCTGTATATCTTCAAATGGCAAACCTTATGTATCTGAAATTAATCCTAGTTTTTGGTGTGTGATCACAGAGAAGGAACACAAGGAATCAACAACCATGGATTCACAGGGCCAGACAACTTCATTGCAGAGACTTCAGAATGTAGAAAAGGTAATATTTTGAAAACATGTTCAAAAATGTCATGAAAGTGACAGAATATCTAGTTTGTTCGATTAATTTTTTTATCCTTTCTGCTGGTCATGTGTAGAGAATTGTGAAGGTTTTGGAGCTTGCAGGAGGAGTCATGGATGAGCTTGCAAGCCCTGTTGGTCCTAGGAAGGATGTGGTCCAAAACCACTGCCTTGAGTTCATGCAATTAATCAAGGTCCTTGTCTCCCAGAAATCAATTTATTCTTTTGTCTATTCATGTATATAAAAGAGGGTGGCATTATGGGTGCAAGTCCCAAAAAGAGCACACACTACCAAAACTGCACTAGTCTTATTTAAGTGGTCTCATTTAAATAATTAACGTTATATAAAATTAACAGCAGGAATTATGTAAGAATATTTTATTTACTTTACATTAATCGTTTGTTAGGTACTGGTTCAGAAATTAGCAAAATAATGAAAGAAAAGATAGGATTTTATTGACGAAATCTCCATTAGCCTAGAATCAGCGAGCCTTGGTTAGGAAGAGAAAAATAAAATTTGTTCTCGGATTTCAAGAACCTGAGATCTCTCCCCCATAACCAATCTCTAACTTAACAATATATCTCTAATCTTTCTCTCCCTATCTCTTAATAGCCTATATTTGCCAAATTTCATTAGAATTTTAACTGAGTCAATAACCCTAACCACCTTCATTAACAGAAATTCAGTCTCATAGGCTTGTTCTAACACCACCTACTTGCAGCCAGCTGGAATTTGAACTCTGAATCTGTGGGCCATACATAAATGTCTTAAATACTGACGATCTAACATGTGTCAACAACAACTAGATCAGCCTATTAGTACTTACAGTTACTTAAAGTTCATCATCATGTTAATGCTCAAACTTGTGCTGTAATTTTGTAACTCTTTAATTTCATTTTTTTTTTCAAATCTTTCAGGACATTCAGGTTGCATTGCGGGATGAAATCAAAAGTGCTTGTGAATATCGTCCATTTGAGAAATGTGACTATGGTTCAAGAATCGCCAATGAGATTTGTCACAAGAAAGTGGAATTTATTATGTCACAGTTAGATGCAATGAAACAAACTATAGATGAGTATCATGCAGCAGTTTGAAAATGTAGCTTAGTTCCCTGTAGAAGCCTGGGTTACCACCCGGTAATTGACTTATTTGGGATTGCTTATTACTAGTTAGCGTGTTGTTTTGGATGGGGTAACAATTACCCTTGTAGCGATAGGAAATCAGTGTATTTATTTGGATGATTATATAGTAGAATATCCAATGAGTGTGGATTATTTTGTTCCCCCTATCTTATTACGGGTAACACATTTGGATGATTATATAGAAGAATAACTCAGTTTCTTGCTA

>GmMED12-1

CTTCACCCTTTATTCCTATCAGAGGAAGCAACCAACAGAAGCATCGTCGTCGTCATCACCACCCAAAAACTCAGTCAAAGTTTCACACCCTTTTTCTTCTTCATCTCTGCAATCTCTCTCTCTCTCTCTCTTCGACCCTTCTGCTCAAAGTTCCAAAATTTGTCTTCCCAGGCACGCCCCCAGTTTTCCCTTTTAACAATCTTCAACCCCGTTTCACCTTATTTTTTTATTATTATAAAAGAAACCCCTCTTTTTCTTAATTACGAATCTGAAACCCTAGCTCCCATGACCCCAATTTTTTCCTCCGCAATTGGCTTCTGAAGTTGAGGGAAAGGCAAAAAAAATTAAAAATTGGGGATTTCGCCCCCACAAACCCTAGTTTTGGATCTTGCTTCGGAGTTCAAATTTTGGCGCTTTTTCTTGCATTAGGTTCGAATTTTAGGTTTTTGCAATTTCGCTTTCAATCCCACGTTTTTTAGACGATTAAGCAATTGGGTTGTCTTCAAGGTTGTGTTTTGCATCCGAAGAAGGTTTCATTTGGTGCTCTAGTTAGTTCAGTAGGTGTTTTGAGTTGGGAGGAAAAAAATTTACAAGTGAAAGGGTATTTATTTTTTTTATTGCCTATGGGTTTTGAACTATACTTAAGGATCGGGAATGATTTGATCAAGGTTGCTACTTTCTGTGTTCTCTTGTAATTTATTGCTTCAGATATGGGCTGGGGCTACAATTATAGCTACCATAATGATTGATCACAACAAATATATATTCTAGTTCGAAGAGTTGGCACTTAATCATATATAGGTACAATTTCCATATGTATTTTCATTGGAATTTGTCTCTAGGAAGCCTTTAATGGAGATTCCAATTTGAAAGTTGTGCTCTACATTGATAACTAATATTTTATCTGCTTATTTATGACTTTGGTTTTAGCGGTAGTATAGATGAGAAGATGCTGTTGAGGGTGTATGTTTAAAATTTTCTAATTTTCCAACTGCTTGGCCATGTTCTTCTGTTTGCGTTTTAGAAGGAGCTGTGGTTTTATATGGGGGTATCAATGTTCACTTCAGTTTCAGAATATAACGGTCAAGTTCCTCAAAATCTTTAGCAGCTGGGTTGGGTTATATCTTGTGAATACCAGACTCAGTGTGAACTAGACTTTAGTGTTTTGTATTTTGTCGTTTTCCAGATTGTGTTTTATGTTTAGCATTTTAAAAGCAGTCTGCAGAATGGAGTTTGATATTATGATTTATTTGTGGCATTGTTGTTTTTTTCAGTTGTTATCCATTTTGTGTTTGTATGCTTACAAATAAGAATAATAACACTCTGCTTGATGGTGATTAAAATTGGGATATATAATAGTGCATTAGGGATTTCAGTACATATCCTTTAGCAGCTGAAGGAAGTCCAATAATGCTTAGCCGTATGCATCCTTTTTTCTGTTTTATTTTAATGTACTTCTTGTTTGCTTAATTGGCAATGATTGGAAATTAATTGGGTGGCATTGTTGATCATGCAGTTTAGCTAATACTACTTGTGGTATGATGCCGAGGGCAGTATGCTGGTTATAGTTGGGGGAAATGCAAAGGTATCATGCTGGCAGCTGCACTAGTGCAGTTAACAACAGTACAATAGGTGGACCATCTGCTAGGGACCCAGGAAGATCTGATTCACCTTCTTTGCCAGCTAACTTTGCTGTAAGTTCAAGGTAAGGGAGCTTCCTGTCACTTTCCACTTTTGCCACTTTCTTCTTTTCAATGATTGTGACTATAGCATATGTGATAAAGGAACTGCAAATTCCAAATATTTGTGGTTGAGAGAGACAGTGGTCTGCTTTAATATATTTTCTTTTTCTGTTATCTAATAATTTGGGTTAATGATTTTTTCTTTTTTCACTTGATGAACATCATTTTGAAAATTGTGAAACTACACCGGAGTGGGGTTCATTTATTTCTTGAGTGATCCGGATATTCAAGCATTTATTATATCTTTTCATTTTTCATTTCTTATGCATTTAATTGCTTAGAGTGTCCTCTGATTACAACCAATTAAATATTCTTTTTCCTTTTATTTTCTTTACTATTAATCTGTCTACTTTTGATAATCTGCTTTGATGGTAAAACGGCAAGCAATATTAGGTTAGATATTCATGCTCATAACCATAGTGGTGTGAAATAGCTGACTCCAAAACTACTTTAGTAGGATAGTGGGTAGTGGGATGACTGCTAATATCATGCTTTGAGATGCACATTAGTTAATTTACAATACACATGTATAAATACTGAAAAATAAAAATGACCCAAAATTAAAGACATTAATATTCATAGTAAACAATCAAAAGTCAGAATTTGAAGCTAAACACTCTACATATAAAGATGAAACACCTGTTTGACAGTGGAGTCTGCTATCAAACCAAAAGAAAGGAAGGAACAAAGCAGCGGTGCTGAGACAGAACAGAGAACAGAGGATAAAACACCAGGCAAGGGCCATCGAGGTCTACAGGCTTTCTGGAAACTGTCACAACCTGGCCAGAAAAAGCCGGAGATACTGCTGGAGAAGGGAGGTGGTCGTGGTTCATCCAGATTCAGGGTGACTGTCCCATTATGATGCTAAACCAACAACAACCTTGCATACTAAAACTAGGATTTTTTTTTATTAACAGATTAACCACTGAAAAAGTTGAAATTGAAGATTACCCTCCACAAAACCCCCTGATCGCCTTTGTTACAGGGGTTAAAATGAGTTTTTGAACTAACACTTCCAGGAAAACATCAGCCTTAAATGTTCTTGAATCCTGTTGGAGTTGTGCTGCTGTTGTGCACAAATTTATGACTTCTGCTGCAAGTGTGACTGTGAACCAGAAACTGTGATGTGAGCCTGCTTCTGTAGCTTCCCAGGTCCGCACTGCAATGCTCCACTTCTATAGCACATCCAATGTTGTGAAAATTGAGATTTCACGGGACAGGAAAGGGGATGAAAGATTGTAAATTGAAGGATCTCATGGATTGTAAGATCCTCCCAAAAGATGCAAAATTATATATAGTAAATATACATGTGATAAAAATGACATAGATAAGGAAAATATCTTGTAAATGATAACATTGAGCTAAACCTAAACTGGAAATTAATATTTATAGATTTATAAGTCATAGCTAATAGGTCCGAAAAAACTCATAATAAGTCAAAATAGTAAACATAAGAAATTCATAATCAGTTAGCCTAAATCAAAGTAATAAATAAGTAAGAAATTTTGACTCAACCACAAAAACAGTCCAATAAAAAAAGAAATTTCTTACATAGAACAAGAGAGGCACCACATCACACCAACAAAACAGAGCAAGAACAGAACAGAACCCTGACAGAGACACATGTTATGAACAATATGCAACAACCCAGTGGACGCAAGGCAGAGACAGAGCAGAAAATGTGGTTGCAGTGAAACGGAGAGCAGAGGGAGGAGTAGTGAGGATTGAAGTCATCGTCGCGATAGATGGTTTTGCAAAATGGGTGAAATCAAGAATTTTAGGGCACTTGCAAACGAATCGGCTGGGACAGCAAAGGGGTTTTGTATCAAAACCTGACCTGCGGGAGATTGGCATGGAGTGGATCATGCGATCTTGGATGAGGAAGATCGCAATCCTCTGCAATCTTCACGAAACCTGATCCTTATGTCGATCTTGCGTGGGATTTGGGCCGAAAATTGTTAAAGCGTGTAATGTTGCAATCTTACCCGAGATTTTCACAACCTTGAGCATGTCATTAATTGGTCTGCTCATAAATAGCAAGGACCAAAAATGTTAGTTGTAAGAAAATTAGTAAATTGGTAAATACAGGGTTGAAAAGTTTTCACTGATTTAATTATTTTCTTTCTGTATAGTGGATATGTTTGCAATTTTGCATCCTATTTTGATTTTTTTATTCTTCTAGTAATATCTTCTCGTTTTCATGATATTGTTAAGTAAAATTTATGGTAGCAATTTAAATAACTATTTGCCTGGATGTGTTGACTTAATAAATGTAGAAAATGTTAAAAGTGTTAATTCAAGTTTTTTTTTTGTCCTTTTTAATTTGAATTCTGTTTATATTCTGCAAAGTTAGCAAGTATTTATTGTTGGATTGGTTATATAAAATTAGGAATATATTGAGTTGGACAAAATAATTACAAAGTGAAAGGAGATAAAGAATTCTCCTAAAGGACAAAAAATACAGAAAACATTCAAGAAAAAAGAGAAAATTGATGTAACAAAGCTTCCTTGATCAATAATTAACATATCTGTTAGCAAAACCCCATTAAAAAGGCCATGTGCTCTATGCCAAATAAATGCCAAACGTTTTAATGTGATGAACCAAATAAACCTCTTTAAACATTTGAGAGTTTTTCTCCTGCCAAATGCACCAAATGATAGCAGAAATTTCACATTGGCACAAAATTTAGCCTGTCTCTTTCTACCAAAACCATGAAAGTTAATCGTCAAGAATTGTTCCATGGACCTAGCACACACCCAGCATTCACTAAAAGTGCCAAATAAAGGAGTGCCTCACCAGGATTGCAGCTTAACAATGGAAGAACGAGTGAGGAACAGATTCTGAGATGGCATCACATGTCATAAAGGGGGTGTTTGGTAGGGGAGAAATTTTCACTTTCCTAGGAATCCTTGGGAAACTTTAGTTTCCCTCTTTGGTACGCATTTTTAAGAAACTTATTCCCAGGAATTCCTGGGAATCGTTTTTAACTAACATTCCCACAAAAACATTCCTGTGGAGGCGGGTGGGAATCCTACTTTCCCAAGTTTAATTTTTTTTATTACGGTAGAAATATCATGTTACTCTTATTAAATTATTTAATGTGGTAAATAATTAAAGTTATTGGTAAAAATATTATGTAACTCTTTGATTCCTAGGAAACCAGGATTCATGATTCTTGGGCATGAAAAAATTTCTCTCCTACCAAACACACCCTAGGGGAAAGAACCTTATGTAGTGCAACATGTCATTAGTATTAATTCTATTAATTAAGTACCACAATCCAAATATAAAACCTGACCTTCCCTTGTCCACTAAAACCATATAAAGTAGTAAACATAATTTTCATATAAAATTAGAGAATGGCGGCACTAGTTCGGCAGAAATTGGTATAATCTTTCACTACTTCCCCTTTCTGTTTTTATGTTTATCAAGATAAGATTTCTCTTTTTCTTATCATTTTAATTTTTTTTTTTAGTGGGAGTGATTCTACCATACCATAGCTAGTTTCAAAATATTCTTAATCATTTGAATGCACAGAGTACCATATGATGGATTGCATTTTTATTTTTTATTTTTCTGAAAAGATGGATGCAGTGTAACTAGGGTTGTGTATATTTTAAATGAAGAACTAAATTGGAAAGCATGGGTTGGGAGTTCTGTTTTTGTTCCATTCATTTGGTTAATAATGACATTTTAAATTCATATTTTCTTGCTTCTGGAATATTGTTTATATTTGTTTGACCTAGTATTGCTAAAGTTTCTTTTCTTATAAAGTTCATATTCAATAATAAAGTGGCATCTTATTTTCTTACTATGCTTAATCTGATGCTGTTAATCAAGTTGAATACAATGGTTTTGTGTTTGCAGGCGACAACTACCATTAAACCCATACAAGTTGAAGTGTGATAAAGAACCCTTGAACTCTAGGTAGTTCCTGTTCATTATAGCATTTTTTTAAAATTGTTGCCTACTTTTACTTCATGATTAACTTACTAGTACCAGGCTCTATTGGGGGGAATATTTTCATATTGCGCTGTTAAAATACAAAAAAAATGCATTTATGATGTTTTCCCCTTCCCCACCTTGTTATCAGCTTATGTTTTTTCTTGGAGGAGAATTCTCCTTTTTTCTGAGATTCAATAAACCTTTATAGAATTGTTCTCTTTCTCTCCAGATTTCTCAGCACTCTTGTGGTTGTGGGTTTGTAATTCTACTCTAAGGCGTTAAGCTTCAAGGTTTTATTATCTTGTATATTAACATGTAAAATGAAATTATTTTTTCTCAGGCTTGGGGCACCAGATTTTCATCCCCAAACGCCAAATTGTCCTGAAGAGACTCTGACCAGAGAATATTTGCAATCTGGATACAGGGATACAGTTGAGGGGCTCGAGGTATGCTGTTGAGTACTTTTTTGGGAAAGCTCAAATTTTGAGAAGCAAATGTTTTTTTTTTAAATCTCTCAGGAAGCTGACCAAAAAGCCAAAGCTAAGAAAAAGATGCTCTAATTTTACTTTTGTATTTATACCAAATCAGATATTTTAGAGTAGCAACTTTGCTTATGATTGATTGGGTTATACTTCTCCAGTTATCCATTTGTTGTTTTTTCAAGGTTTGAATATTTTATGCTGAACTCTCATATTTCTTTTGTAAATATTTTTGATGGAATATGTCCAGGGTAATTGAAAGTTTTGGAACTGTAATCTGGCATTTTCTTGAACTTGGTGTTTTTTTTATAATCAAATTCTTTCATTCTTTCAAAATGGAAATAGTGTTATGGACATGCATATATTCTTTAGAATCATAGATCTCCTTGATGACTCACTGAATTATAGAAATCAAATTGTGCTCTTATTAGAATAGATTATTTTCAACTGTTCAAACCTGCTCTTTTGTCTGGTATCTGCTATTTTTTGACCTTGTCTTGCATCCTGTGTCTGTTAGGAAGCAAGAGAAATTTCACTAACTCAGGTTCCGCATTTTAACAAGAACATTGTTCTTAAATGCAAAGAGGTGACTCTCTTGTCTCTTGTCTCTTTGGAGATATTTATGTCCTCTTTATTTTTGTTTATATGTCTGAGCTCTGAATTTACTGCACATTTTTTTTGGGTATTCATTGGAAGCCTATGTGGATATATAACTTGTAGTTTTTCATCTAATTTCCAGGCTATTAGAAAACGTCTGAGGGCCATCAATGAATCTCGTGCTCAGAAGAGGAAGGTGATGATCTTTAATCTTGTGATCTATTTATCCTTGCTGAACATTCTACTTCTATAGTGTATCCATATTTGTGTGCTTTCTTCCAGTTTATTTTCGATTTAGTGCAGTCTACCTATCTCAGAAGGTGTTTGATCTATATGAGCGTTTGATTTGACAGGCTGGTCAAGTATATGGTGTGGCTCTTTCAGGATCACAACTTGGCAGGTCTGGCATTTTCCCTGAACTAAGACCCTGTGGTGAGGACTTCCAGAAGAAATGGATTGAGGTATACTTGGTGATAGCCTTGCTCCTACTGTTTTTTTCTTTGTGCAACTTCCTGTATACCAGCTTTGACCCCTCACTTTTGTTTCTAATACATTTTTGTTAAATTTATATGTAGAAAGCACAAGGTTTTAAATGGTCACTAACTTAGTAGTAGATGGTTGTAGTCTTGATGTGACTGGCTGCAGGATCTGAATATTGGAAGGATCTGGAATGTTTTTATGCTGAATTGTTCTGGACAGGCTTAGCTATTCACGGATTTACTCAATTAGGAGAACTGTTATATACTTATATAAGTTACTATGACTAATTCCTTAGGTTTTTAGTTGTTGATGACTTGATGCTGACAGGTTGTTATGGCCGTAAAATCCTTAAATACTACTTTTTTGGATATTACTTGGTGGTTCAATGTATCTTTAGTAACTTGGTTAAAATTATATTATAGGGTTTATCTCAGCAGCACAAGCGATTACGCTCTTTGGCTGATCATGTTCCTCATGGTTATAAAAGGGCATCACTTTTAGAGGTTCTTATCAAGAATAATGTTCCATTGCTTAGGGCCACCTGGTTTATCAAGGTTACTTACCTCAATCAGGTAGGGATGCTTCAGCGTTGCTATATTTACCTAATATGATGAATTGCTTAATATCTTTTAATATTGTAGATTATGATTGACCTTTTCAGGTTCGACCTGGTTCTGTTGGTATTTCTTCTGGGGCAGCTGACAAGATTCAGCTGTCTCGCTCTGATGTTTGGACCAAAGATGTTATCAATTACTTACAAACACTTGTGGATGAATTTTTGTCAAAGAATGCTTTGCATTCTGCTTCTCATGGTCGAGAGCGATCACCACAAATCCCTTATACTGGCTCACTGCAGAATAAAAATGATCCATTATTATCTGTTTCTGATGGTGAAGGACCATCCTTACATTTTAGATGGTGGTATATTGTGAGGCTTCTGCAATGGCATCATGCTGAAGGGTTGCTTCATTCTTCTCTTGTCATTGATTGGGTGTTTAATCAACTACAGGTCTTTACCTTCTACATCAATTTGCATTTAACAAGTACAACGTGCTCATGAAACTAAAAGTGTGATGTATATATCTTTCACTGATTTCAACCTTCATGTTTTGATTTCTCACTTCTGTAATTTTCAGGAAAAAGAACTGCTTGAGGTTTGGCAGCTGTTATTGCCTATTATATACGGTTTTTTAGAAACTATTGTCCTGTCTCAATCTTATGTACGCACTCTTGCTGGATTAGCTCTTCGTGTCATTCGTGATCCTGCTCCAGGTGGTTCTGACTTAGTAGATAATTCCAGGAGGGCATATACAGCTTATGCTGTGGTTGAGATGCTCCGGTATTTAATACTTGTGGTGCCAGATACTTTTGCTGCTCTGGATTGCTTTCCTTTACCATCCTCTGTAATTTCACATACAATGAATGATGGGAGTTTTGTACTAAAATCAACTGAAGCTGCAGGGAAGATAAAAAATAGTTCAGATGATTTTGGTCACATTATTTCATGTATTCAGAAACATACAGAAGATTTGGCGAAGTCTGCAAGCCCAGGCTATCCAGGTCATTGTCTAGCTAAAGTTGCAAAAGCTTTGGATAAATCTCTTGTGCTGGGTGATTTGCGTGTAGCATATAAATTTCTTTTTGAAGAACTTTGTGGTGGAACTGTATCTGAAGGCTGGGTTTCCAAAGTCAGCCCTTGCTTAAGGTTATCTCTGAAATGGTTTGGGACTGTAAATACAGCCCTTATATATTCTGTGTTTTTCCTCTGTGAGTGGGCAACTTGTGATTTTAGGGATTTTCGCAGTACTCCTCCTCGTGACATAAAGTTTACAGGTAGGAAAGATCTTTCCCAAGTGCATATAGCAGTTAGACTTTTACTGATGAAGATTAGGGATGTGAAGATTTCACAAAAACAAACAAATGAAAATCACAGAGCCAGTCATCTTGCAAAGAATTCGAGTCAGTGCCAGAATTGGAATTATGTGGGTAATGTATCCAGATCGAAATCTAGTTCAAAGAGTATGGGTTCTTCTGTATTTGAAAGCCCAGGTCCTCTACATGATATTATAGTTTGTTGGATTGATCAGCATGTGGTGCATAAAGGGGAAGGTCCCAAGCGCCTACATCTATTTATGGTTGAACTCATACGTGCAGGCATCTTTTACCCCTTGGCATATGTACGCCAGCTAATAGTGAGCGGGATCATGGATGTGTATGTAAATGTGGTTGACCTGGAGCGATGGAGGAGACACTATCGAATCTTAAAGCAGCTACCTGGATGCTTTATCCATGATGTTCTGGAAGAATCAGGGATTGTTGAAGGGCCGCAGCTCAAAGAAGCCTTGCAAATTTACTTGAATGAACGCCGCCTCATACTTCGGGGTCCTCTGAGCATGTCCCATGATGATGCCAATGGTTCCAATTTATCTGCTCTGAAGAAAAAAAAATATCCAGCTTCTACAAAGGATGAAGTTTCTGCAGTGCCAATTGATCAGAGAAATGTTATTTCTACCACAATATCTTCTAAAAGTGCAAAGGATAATGCTAACATTGAAGAACTAAGAACAGCAATCTCGGTACTGTTACAGCTACCTAATTGTTCATCTAATTTGAGCACTACAGGTGATGAATCTGAGGGCAGTGTTAGAAGACCTATTGGGTCTCCCTACAGCAAGATTGATCCAGTGGAGGGTACACCTGGGTGTGAAGAATGTAGCAGAGCAAAGAGACAAAAGTTAAGTGAGGAAAGAAGCTCATTTGTTCAAGGGCATTCTCCAGTTCAATCTGATGATGACGATGCATGGTGGGTGAAAAAGGGAATGAAATCCCCAGAGCCTCTCAAAGTTGATCAATCTCAGAAGTCAACCAAGCAGGTCACCAAGATTCGGCAAAAGAATGTGCGTAAAACTCAGAGTCTAGCTCAACTGGCAGCTTCTAGAATTGAGAGTAGCCAAGGGGCATCAACTAGTCATGTGTGTGGTAACAAGGTAAGCTGCCCTCACCATAAAACTGCTATGGATGGAGAGGGGCAGAGGTCTGTCGATTGCATCCAAACAAGTCATTTTGGGGATATAGTTTCCATTGGAAAAGCATTAAAGCAGCTACGTTTTGTTGAGAAAAGGGCACTAGCAGTTTGGCTGTTGACTGTTGTTAGGCAGGTAATTGAAGAGGTGGAGAAAAATATTGGTAAAGTTGGTCAGTTCGGCAGGCCTTTTCCTGTTGCGGATGATAGAGGCTCAATACGGTGGAAACTTGGTGAAGACGAACTTTCTGTGATTCTTTATTTGATGGATATCTCTGATGATTTGGTATCAGCTGTCAAGTTCCTCCTTTGGTTGCTGCCAAAGGTTCTTAATAGCCCCAATTCTACAATTCATTCTGGGAGGAATGTGCTGATGCTACCAAGGAATGTGGAAAACCAAGTTTGTGATGTGGGAGAGGCTTTTCTGCTATCATCACTCAGAAGGTTTGCAGCTCTGCAATCTTATATATATATATAGTTTTTGTTTGCTGTCTCTTGGAAAATATTTTTATTTTGACTTTCCCCCATCCAGCATTTCCTTCTAATTATTGTGATGTTTGCTTCTTTTTTATTAGAGTAGTCTACTACTCTAATACTTTTCAATGGCTTAGCACCTTTTACATATGTGATATAAATACTATGCTTTATAGAGTACCTGGTAGTAAGCTGTTATATCTGTTTCTTCTTATGCTCCATTTCTTGTCATCTTTAATCAGGTCAGTATTGATTAAATCTGCATTGCTTGTCTACTCAGGATCCGGATTGCCTAAATAAAATCCTATACTTACAAATTAACAACATAAATACTTGTTCCAGTTTCAAGTTTTAACATAATTATAGTACAAGTTTGCTGAACCTTCAGTGAGGTTTCTACATCTATTATGGGCCATCCTCCCCCTGAAGTATTTATCATTTTTGTTATTCAACCAGTGCTTTACGAGTGGTGTTTTCCTTGTGTTGGACAGTTTCTTTATGGATGTTATGAGATTTTGTCAAACTTGACTTCTGCAATGTATTTGATGTAGGTATGAGAACATTCTTGTTGCAGCAGACCTTATTCCCGAAGCTTTGTCATCTGCAATGCATCGTGCTGCCACTGTTATTGCATCTATTGGAAGGGTTTCAGGGTCAGGGGCCCTAGCTTTTGCTCGGTATTTGTTGAGAAAATACAGCAATGTGGCTAGTGTCATCGAGTGGGAGAAAACTTTTAAGACTACATCTGATGCTAGACTTTCCTCTGAACTTGAGTCTGGTGGGTCAGTGGATGGAGAGTTAGGTTTACCCCTAGGAGTTCCAGCTGGAGTCAAGGACCATGATGATTTTTTCCGTCAGAAGATAAGTGGTGGTCGGTTACCGTCCAGAGTAGGTGCAGGCATGAGGGATATAGTGCAGCGTAATGTTGAAGAAGCGTTCCACTATCTTTTTGGAAAAGATAGAAAGCTATTTGCTGCTGGTACACCAAAAGGTCCTGCTTTAGAAAAATGGGATAATGGATATCAAATTGCTCACCAAATAGTTATGGGTTTGATTGATTGCATAAGGCAGACTGGTGGTGCTGCTCAAGAAGGAGATCCCTCTTTGGTCTCTTCTGCTGTTTCTGCGATTGTTGGCAGTGTTGGTCCAACTTTAGCAAAAATGCCTGATTTTTCATCTGGCAATAATCATTCAAATATAATGTCAGCTACAAATTCATTGAACTATGCCAGATGCATTCTGCGAATGCATATAACCTGTCTATGCCTGCTTAAGGAAGCTCTGGGAGAACGCCAAAGCCGTGTATTTGATATTGCTCTAGCTACTGAAGCTTCTAATGCTCTTGCAGGTGTTTTTACTCCTAGTAAAGCATCTCGTTCTCAGTTTCAAATGTCTCCTGAAGCCCATGATTCTAGTAACACTATTTCAAATGATATGGGAAGCAACTCTATTAAGGTTGTGGCAAAAACAACAAAAATTGCTGCTGCTGTTTCTGCACTTCTTGTTGGTGCAATTGTATATGGTGTTACCAGCCTGGAAAGGATGGTGGCAGTTCTCAGATTAAAGGAGGGGCTGGATGTGGCACAATTTGTAAGAAACGCTAGATCCAATTCAAATGGAAATGCTCGTTCAGTTATGGCTTTTAAGGTGGATAGTTCAATTGAAGGTCATGTCCATTGGTTTAGATTGCTTGTTGGAAACTGCAGAACAATCTGTGAAGGGTTAGTGGTGGAACTCTTGGGTGAACCATCTATTATGGCTCTTTCAAGGATGCAACTTATGCTTCCTCTAAATTTGGTCTTTCCACCTGCCTATTCAATATTTGCCTTTGTTAGGTGGCGACCTTTCATGCTGAATGCTACAGTTCGGGAAGACATGAATCAAATTTATCAGTCTCTTTCAATGGCCATAACTGATGCAATAAAACATTTGCCATTTCGAGATGTATGTTTTAGAGACTGTCAGGGTCTTTATGATCTTATGGCTGCAGATGCAAGTGATTCTGAACTTGCAACCTTGCTAGAGTTTAATGGGTCTGATATGCATTTAAAATCCACAGCATTTGTTCCCCTACGCTCTAGGCTTTTCCTGAACGCCATGATTGATTGTAAGATGCCACCATCTATTTATACAAAGGATGATGGGAGCCGAATGTCTGGACTTGGTGAGTCTAAAATTAAATTCACAGATAGTGAGTCTAAGCTTCAGGATTTGCTTGTGCATGTTTTGGATACCTTGCAACCTGCCAAATTTCACTGGCAGTGGGTTGTACTCAGGTTGCTCTTAAATGAACAAGCCCTCGTTGAAAGACTAGAGAATCGTGATGTGTCCTTGGTTGATGCTATAAAGTTGTCCTCACCTAGTACAGAGAAGGCTTCTGCTGCTTCTGAGAATGAGAACAATTTTATTCAAATACTTCTCACAAGGTTACTGGTTAGACCTGATGCTGCACCCCTTTTTTCGGAGTTGATTCATCTCTTTGGCAGGTCACTAGAGGATTCAATGTTGTTGCAAGGTAAATGGTTCCTTGCAGGCCAGGATGTCCTCTTTGGTCGGAAGACCATTAGGCAAAGGCTACATAACATTGCTATGAAAAAAAATCTTTCTGTTAAGACCCAATTTTGGGAGCCATGGGGTTGGTGTAGCCCGTCTACTGATCCATTAACTATCAAAGGGGATAACAAGAAGTTTGATAGCACATCCCTTGAAGAAGGAGAAGTTGTTGAAGAGGGAATGGATTTGAAAAGGTGCCAGCAGCAAGTGACTGAGAGGGCTCTTATTGAGTTGCTTCTTCCTTGCATAGATCAAAGTTCTGATGAATCCCGCAATTCCTTTGCAAGTGATATGATGAAACAGTTAAGTTATATTGAGCAACAAATAACTGCTGTTACAGGTGGAAGTAAACCAGTAGGAAGTGCTCCTCCTGGAGTTGAAGGTCAGCCGAATAAAGTAAATAACCGCAAAAATATGAGAGGTGGAGGCCCTGCATTAGCTAGACGACAAACAGTTGCAGCAGATTCTTCTCCACCATCTCCTGCAGCTCTACGAGCTTCTATGTCATTACGGTTGCAGTTGCTCCTGAGATTTCTTCCTATTCTTTGCACTGACAGGTAATAAGACTGGATATTGTAATGTGTAATTAGCTTTCTCATTATAGGATTCTTGATGCACTGGTCAAGTGAAAAAAAAATAAAAATCTGCGGTCCTTTTCTCCTTTCTTGAAATGTAGAAAAGATTGGAGCTAACTGCTATATGATTATTTTCAGTTTCGGAAATACAATTCTCTATTATGTATGAATTATCTGGGTCCCAGCTGCTGCTACTGAAAGTGATAGTTTTTCCTTGTAACAAAATGGATTTGAGCTTTAAATTTTGAGAGTTGGAAATCAATTAAATCAGTTAGGGCCTCTCTTTTAGTAGTGGAAATATGGATATTTTTTCCATGAAGGCATTGCCCTTTTTTTATTTTTTGTGTGTGCTTGTGATGGAACTATTACATCCTAGAATGTTAGAAACTATTCTTGAGTTCCATTTATTTCAATTTTGCAGGGAGCCATCTGTGCGGAGCATGAGACAATTTCTTGCCACTGTAATTTTTCGTCTCCTTGGTAGCCGGGTTGTGCATGAGGATGCAGACATTTCGGTGAATGCTGTGCCATTTCTGCCTATAAGGGAGGCAGAGTCATCTTCTGAAGTTGCTTCTGCTGCTTTTGTGGATTCTTCTTCTGGGAGTTTGTTTGATCGTTTGTTGTTGGTTTTGCATGGATTATTAAGTAGTTATCCACCAAGTTGGCTTAGGGCAAAGCCTGTTTCAAAGACAATCAGTGAACCTACGAGGGAAATTTCTGGAATTGACCGAGAATTGTTGGAGGCTTTGCAGGTTTGTTCTTTTAGCCCGTATGTGTATAATTTTTGGTTCTAGTCACTTAGCCCTTAGGTGGCTTTTCAGCATATTTAACACTAATGTAATTTTCCTTGTGAAACTGGTTGTCAAAGATGAAATAGCCAACAAACATAATATTCTTTGAAGTATTGGGAAGGTGTTAATTTAAAATACAATGTCTTAACAATTATTTGTTTGTGCATGTATGATCTGCATGCTTAGAAGTTCTGTAGCTGATTGGAGTCTTAATTCAATTAGTTTTTCTTTTTAAGGATTTATAGATGCTGTATGCAAGTTTTTTTTTTTTTTTTTGTTAATTGGATATGATGGCATCATGGTAATACCAGACATTTCTTCAAGACATCTTGTATTATTAGATTTTCTGTTTTGCTACTTACTAGGAAGGACTGATACTTGAAATTTTTTATACCTTTCCGGTGTGAGATATAAACCGCCATATGGTACGTATGCTATTTCAACATCATTGGAAATTGTTATCTATTGTAATTTTTTTCTTTTTCGTTTTCCATTGAGGTTTTTTGTCAACAAATGTGGGATATAATCAGAATTTGATAAATTCTAGACTTCGGTTGTCTGCAATGAATAATATTGGAACTAAAAACTCTTTATTTTTGTTTACTTTCACCCTTCAATCATGGAATGACCTGACCATTATTTTCTTATGATGGCTTTATGTGTTTCCCAAGGTTTTTCATAAATGATCTCTATAATATGTATGTATATGTTATATGGTGCATCATGCCATGCCCATACCTTTATTTTTCAAGAAATTATGTATCACATGCTGTACTGGTGTCATATCAGATATTGCTAGCCATATCCGTGCTTCATAAATCTCTACTGAAGCTCTACAATTTGGCAATGTTTATGTTACATATTTGGCAATCTAGAAATGTTAATTCAATCTTCCTAGTCACGCTTGTCTAATGATCTGTTATCCGATGCAGAATGACTTGGATCGTATGCAACTGCCAGACACTATTCGGTGGCGTATCCAAGCTGCAATGCCCATGCTCATTCCCTCTATGCGGTGCTCTTTATCCTGCCAGCCACCATCTGTTTCAAATTCTGCTCTTGTTTGCCTTCAACCCAGCATTACAAATCCTGGGTCTAACTCCAGTAGTTCAACTATCCCTCAGAGGAATTCAGTTCTATCAAGGGTTGCATCTAATGCATCAGGGAAGTCAAAATTACAGGACAATGATTTGGAAATAGATCCTTGGACGCTCTTAGAAGATGGCGCTGGATCTTACCCTTCAGCAGGTAATACTGCAAGTATAGTAAGTGGTGACCACGCTAATATTCGTGCCACCAGCTGGCTTAAAGGGGCTGTAAGGGTGAGACGGACGGACCTCACATATGTTGGTGCTGTGGATGACGATAGTTGATTAACAATATTTTTTGTTGGTTTGGACTTTTGGAGTATACAAACCCCTCAGCTGACAGCCGGCGATTGCAGTTTTGTTATTTATATTTGATATTTGATGCATGAATGGCATGAAGATGTCATTTTGTGCTTGCCCTTCTTGTGTTGAAGTTTAAACGAAGCTGAAGGGGCATTGTTTTGAAAAAAAATGGGTGAAGGGAGGGTTCAAGACAGCTCATCCAATGGTTCTGGCCTGTTTGATCACCCCCATCTGGATTGTGTGCACTTGCTCAGTTCAGAGGTTTGTTGTATATATGGGGATATGATTGTTTTACTTTACCAATTGATCTGAGTAATATAGTCTAAAGTGATATTCGTAGCTGTTTCATGCTCATTTTTCTGTCTAGTGAACGCTTGTGTGAGCACTGTCTATAGCTTTTAGGGGCACAAGCCCACTCAGGCGCAATCAACCATTGTTTCTTTTTCTCATATTCATATTATCAAAAGTTATGTGGTTGATCTGGAGCATCACCATGAGCTGCAGCATTCAATTATTTGTTGGCAATTCTAGATAGTTTCATTTCTCCCTAAATATTGTTTTCCTATCTTGATAGGAAAAACTACATTATGTTAGCTTTGTGATAAATGCACACTTGTATATTAGGTAAATGTTATCAAAGCAAAACCAGAATGCCCCTAAATGGAATTGAACGTTTGTTTCGTATGGCATACATTGCAAAATTTGCAGTGTTT

>GmMED12-2

TATTCCTGAAGCAACCAACAGAAGCATCGTCGTCGTCATCACCACCCAAAAACTCAGTCAAAGCTTCACACCCTTTTTCTTCTTCATCTTCGCAATCTCTCTCTCTCTCTCCGACCCTTTTCATCAAAGTTCCAAGATTTCTCTTCCCAGGCACGCCCCCAGTTTTCCCTTTTAACAATCTTCAACCCCGTTTCACGTTATTTAATAAAATCCTTTTCCTTCTTAATCCCAAATCTGAAACCCTAGCTCCCGTGACCCAATTTCCGCCTCCTCAGTTGGCTTCTGAAGTAGGGGGAAAAGCAAAAAAAAAAAAGTTAAAAATTGGGGATTTCGCGCCACAAACCCTAGTTTTGGATCCTGCTTCGGAGTTCAAATTTTGGCGCTTTGTCTAGCATTAGGTTCGGATTTTGGGTTTTTGCAATTTCGCGTTCAACCCCACGTTTTTTTAGACGATTAAGCAGGTTGTTTGTCTTAAAGGTTGTGTTTTGCAATTTGAACTCGAAGAAGTTTCATTTGGGGCCCTAGTTAGTTCATTAGGTGTTTTGAGTTGGGAGGAAAATAAGTTACAAGTGAAAGGGTATTTATTTGTTTATTGCCTATGAGTTTTGAACTATACTTATGGATGGGGAATGATGTGATTAAGTTTGCTACTTTCTGTGTTCTCTTGTAATTTATTGCTTCAGATATGAGCTGGGGCTACAATTATGGCTACCATAATGATTGATCACAACAATTATGGCTACTTTCTATATGTATTTTCATTGGAATTTGTCTCTAGGAAGCCTTTAATGGTGATTCCATTTTAAATGTTGTGCTCTACATTGATATCGCTTATTTATGACTTTGGTTTTAGCAGTAGTATAGAAGAGAAGATGCTGTTGAGGTTGTATGTTTAAATTTTTCTAACTGCTTGGTTATGTTCTTATGTTTGCATTTTAGAAGGAGCTGTTGTTTTATACGGGGGTATATGTTAACTTCAGTTTCAGAATAGAATGGTCAAGTTGCTCAAAATCTTTAGCATCTGGGTTGGGTTTTATCTTGTGAAAACCGGAGCCAGTGTGGACTAGACTTTAGTGTTTTGTATTTTGTCGTTTTCCAGAATATGTTTTATGTTTAGCATTTTAAAAGCAGTCTGCAGAATGGAGTTTGAAATTATGATTTATTTGGGGCACTGTGTTTTTTTCCAGTTATTATCCATTTTGTATTTTGTATTTGTATACTTACAAATCAGAAAAATAATACTCGGTCTGATGGTGAATAAAATTGGGATATATAATAGTGCATTAGGGATTTCAGTAGATATCCTTTAGAAGCTGAAGGAAGTCCAATAATGCTTAGCCATATGCATCCCATTTATTTGTTCTTTTTTTAATGTACCTTTTGTTTGCTTAATTGGCAATGGTTCGAAATGACTTGGGTGGCTTTGTTGATCATGCAGTTTAGCTAATACTACTTGTGGTATGATGCCGAGGGCAGTATGCTGGTTATAGTTGGGGGAAATGCAAAGGTATCATGCTGGCAGCTGCACTAGTGCAGTTAACAACAGTACAATAGGTGGACCATCTACTAGGGATGCAGGAAGATCTGATTCATCTTCTTTGCCAGCTAACTTCTCCGTAAGTTCAAGGTAAAGTAGCTTCCTGTCACTTTCCACTTATTGCCACTTTCTTCTTATCAATGATTGTGACTATAGCATATGTGATAAAGGAACTACAAATTCCAAATATTTTGGTTGAGATACACAGTGGTTTGCTTTAATATATTTTCTTTTTCTGTTATCTAATAATTTGTGTTAAATGATTTTTTCTTTTTTTACTTGATTAACATCCTTTTGAAATTTGTGACACTATGCCGGAGTGGGGTTTCATTTATTTCTTGAGTGATCTGGATTTTCAGTTCTTATGCATTTAATTGCTTAGAGTGTCCTCTGATTACAACCAATCAAATATTCTTTTCCTTTTATTTTCTTTACTATTAATCTGTCTACTTTTGATAATCTGCTTCGATGGTAAAATAGGCAATCAATATTAGGCTAGATATCCATGCTCATAACCATAGTGGTGTGAAATGGCTGACTCCAAAACTATTGTAGTAGGATAGTGGGTAGTGGGATGCCCGCTTATATGATGCTTTCAGATGCACATTAGTTAACTTATAATACACATGTATAAATTCTGAAAAATAAAAATGTCCCAAAATTAAAGACATACTCATACTCATAGTAAACAATCAAAAGCCAGAATTTGAAGCTAAACACTCCACAAATAAAGATGAAACACCTGTTTGACAGTGGAGTCTGTTATCAAACCAAAAGAAAGGAAGGAACAAAGCAGCGGTACTGTCACAACCTGGCCAGAAAAAGCAGGAGATACTGCCGGAGAAGGGAGGTGGTCGTGGTTCATCCAGATTCAGGAAGACTGTCCCATCATAATGCTAAACCAACAACAACCTTGCATGCTAAAAGCCTAAAACTAGGATTTTTTATTTATTAACAGATTAACCACTGAAAAAGTTGAAATTGAAGATTACCCTCCGCAAAATCCCCCTGAACATCTCTGTTACAGGGGTTAAAATGAGTTTTTGAGTTAAAACTACGAATCATATTTTGGAGAACTCAACTTGTCTCACACGGCTGACTGAGACTATTGTATATATATTGAAGAAGAAACAAGTACATGACAAGATAGGGTGCAGACTGCAGCCCCTTGTACAGCAGAGAAAGAGTGCAAAATAAGAAAGATAATAAGACATACAAAAAACACATATGAATGAACACTGCCACCAGGAAAACATCAGCCTTATATGTTCTTGAATCCTGTTTGAGTTGGGCTGCTGTTGTGCACTAATGCCATGACTTCTGCTGCAATTGTAACTGTGATCCTGAAACTGCGATGTGAGCCTGCTTCTGTAGCTTCCCAGGGCTGCACTGCAATGCTCCACTTCTATAGCACACCCAATGTTGTGAAAATTGAGATTTTACACGGGACTGGGAGGGGGATGAAAGATTGTAAATTGAAGGATCATAGCATGGATTGTAAGATCCTGATCCTCCCAATAGATGCAAAATTATATATAGTAAATATACATTTGATAAAATGAGATAAGGAAAGTATCGTGTAAATGATAACATTGAGCTAAACCTAAACTGAAAATTTAGTATTTATAGATTTATAAGTCATAACTAATAGGTCTGAAAAAACTCATAATCAGTCACATAAATACTAAACATAAGAAATTCATAATCAGTTAGCCTAAATCAAAGTAAAAAATTCTGACTCAACCGCAAAAATTGTCCAATAAAAAAAGAAGAATTTCTTACATAGAACAAGAGGCACCACATTGCACCAACAAAACAGAGCAAGAACAACAGAACAGAACCTGAGAGACACATGTTATGAACAGTATGCAACAACCCAGTGGATGCAAGGCAGAGACAGAGCAGAAATGTGGTTGCAGTGAAATTGAGAGTAGAGGGAGGAGAAGTGAGGATCAAAGTCATCGTTTTTCAAAATGGGTGAAACCTAGAATTTTAGGGCACTCGCAAACGATTCAGCTGGGATAGCAAAGGGGTTTTGTATCAAAACTTGACCTGCAGGAAGATTGGCATGGGTGGATCGTGCGGTCTTGGATGAGGAAGATCGCAATCCTCCGCAATCTTCATGAAACATGATACTTGGGTTGATCTTGGGTGGAGTTGGGGGAAATTGTTAAAGCATGTAATGTTGTGATCTTACACAAGATTTTCACAAGCTTGAGCACGCCATTAATTGCTCTGTCATAAATAGCAAGGACCAAAAATGTTAGTTGTAAGAAAATTAGTAAATACAGGTTGAAAAGTTTTCACTAATTTAATTATTTAATTATGTTTGCAATTTTGCGTCCTATTTTGATTTTTTATTCTTCTAATAGTATCTTCTCATTTTCATGATGTTGTTAAATAAAACTTATGGGAGCGCTTTAAATAACTATTTGCCGGGATGTGTTGACTTAATAAATGTAGAAAATGTTAAAAGTGTTTATTCAACTTTTTTTTTGTCCTTTTTAATTTGAATTATGTTTATATTCTGCAAAGTTAGCAAGTATTTATTTTTGGCTTGGTTATATAAAATTAGGAATAAATTAAGTTAGACAAAATAATAACAAAGTGAAAGGAGATAAAGAATTCTCCTTAAGGACAAAAAATACAGAAAACAATAAAGAAAAAAGAGAAAATTGATTTAACAAATCTTCCTTGATCAATAATTAACATATCTGTTAGCAAAGCCCCTTTAAAAAGGCCATGTGCTGTATGCCAAATAAATGCCTAACATTTTAATATGATTAACCAAATAAACCTCCTTAAAAATTTGAGAGTTCCTCTCCTGCCAAATGAACCAAATGATAGCAGAAATTTCACATTGGCACAAAATTTAGCCTGTTTCTTTCTATACCAAAACCATGAAAGTTAATCATCAAGAATTGTTCCATGGATGTAGCACATACCCAACATTCACAAAAAGTGCCAAATAAGAAGTGCCTCACCAAGACTGCAGCTTAACATTGGAAGAACGAGTGAGGAGCAGATTCCGAGATGGCATCACATGTTACATTTGGGGGAAGAGGCTTATGTGGGGCAGCATGTCATTAGTATTAATTCTATTAATTAAGTACCACAATCCAAATGCAAAACCTGACCTTAGATAGAGCTTTGGATTTACATAGGGGTTTATCGAGAAGTTATACCTATTCAAAGGGTTATCTTATATTCTATTAGAGATAGAAAACCTTTCCTGGGTCCACTAAAACTACGTAAAGTAGTGTTAGAACTTCCAAGTATGGAGTGATCATATATAATTTTCATTTAAAATTAGAGAACGGCAGCACTAGCTCTGCAGAAATTGTTATACTTGAAATGTTTGAGTATGAAAATATGCACACAAAAAGGATTCTCAGCCTTAGTTTGCTAATATATAGTTCATGATGAAGGCTACAGCCTAGCCTATGGTGACAGATTACAGGATACCTTCTACAAGCTTTTATAACAAGCTAGAAATATAAAACATTATTAAACAATAACTTTAAGACTCCCCCTCAAGATGGAGCATATAAATCGTGTGCTCCAAGCTTGGAACATATAAAGTGGATCCAAGGACCTCTCAAAGACTTGGTCACGATGTCTGCAAGCTGGTTGTTGGAGTTAATAAAATTCAGTACTGATTTCTTTGGACTGCAGTTTTTCCCAAACAAAATGGCAATCGATCTCTATATGTTTAGTTCTCTCATGAAATACAAGATTAGAAGCGATGTGAAGAGCTGTCTGATTATCACAATACAACTCCATGTCTGCTGAGCATCACAAAATTTTAATTCTTGAAGAAGTTTTTTAATCCACACCAATTCACAACAAGAGCCATAGCTCTATAATTTGCTTTTGCACTTGATCGGGCAACAACACTTTGTTTCTTGCTTTTCCAAGAGACAATATTTCCTCCAAAGGATACATCGTATCCAGTGGTGCATTGCCTGTGTATGGGACAACCTGCCCAATCGACATCATAGTACCCACAAATTTGGGTATTTCCATTATCTTTATACAACAATCCCTGTCCTGGAGTCTTTTTGATGTATCTAAGAATGCAAATCACAACATTCCAGTGGTCAATGTGAGGTTCCTGCATAAATTGACTGACAACTTTGACCACAAATGACAGATCAGATCTTGTAATTGTGAGATAAATAAGTTTCCCAACTAGCCTTCTATACCTCTCTGGATCTGGAAAAGATTCACCTTGATCTGACATTAACTTTTGATTTGGATCCATAGGACTATCAATAGGCCTATAATTAGTAAGAATTGTCTCTTCCAAAATGTCCAAAGCAAATTTTCTCTGTGAAAAGACAACACCTTCCTTTGATTGAGCCACTTCAATACCAGAAGTACTTTAGGCAGTTGAGGTCTTTGGTTTGAAAATTACTGAATAAGTGTTGCTTTTGTTGGGAAATTTTGGCAACATCATTCCCTGTAATAACAATGTCATCAACATAGACAATTAGATAAACACACTTCCCAGGAGAAGATGACAATAAAAAACAGAGTGATCTGCTTCACTTCACTTCATTCCAAAATTCTGAACAATATGGCTAAATTTCCCAAACCAAGCATGTGGTGACTAGTTGAGCCCATAGAGAGATCGACACAATTTACACACCAACTAGACTCCCCTTGAGCAACAAAACTAGGAGGTTGCTCCATTTAGATTTCCTTCTCTAAATAACCATGAAGAAATGCATTTTTTTATGTCTAATTGATAAAAAGGCCAATGATGAATAGCTTCCATAGCTAGGAAGAGGTGAACAGTTGTGACCTTGGCCATAGGATAGAAGGTGTCACAGTAATCAAGGCCATAAATCTGAGTGTAGCCTTTGGCAACCAACCGAGCCTTAAGTTGATCAACCTCACCATTAGGCTTGACTTTAATAGCATAAACCCATTTGCAACCAACTATCTTCTTCCTAGGAGGAAGATGAACTAATTCCCATGTGCCACTGTTCTCAAGAGCCTACATTTCAACAATCATGGATTGTTGCCAACCAGGATGATCAAGTGCCTTGAACATTTTTAGGAATAGTAATAGAAGACAAGGAAGAGACAAAGGAGAAATATGAAGGTGATATGCGATGATAACTCAAAAAATTGTAGATAGGGTGAGAATTTTGAGTAGATTGAATACCTTTCCTAATAGCAATGGCTAATCGGAACTTGGTTCACTAGGATGTGATGTGGAGGAAGGATCCGTTGTATGGGAAGCTGATGGCGAAGGACATGAATCAGGAGAACCTGCAGGCATTGAACAACTAGCTCCCTGTGTCCTGTGTGGAGAAGGAGAGGCACTATCAGCTGAACTAGGAATAGGAGACTGAGGCTGATGTCCTTCTTGGTCAGAAGGGTCTTGGGTAGGAAGTATCATAGGACCAAAAGATGGAATGGGTAGAACTTGCTGTATGGAACTAGTATCCTGCATGGAGGATGAGAAAATAGGAGTTTCTTCAAAGAAAGTAACATTAGCAGACATGTAATACTTTTTAGTTTGTGGAGAGTAACATTTGTAACCTTTCTATAGACGAGAATATCCTAAAAAGACACACTTAATAGCCTTTGTAGAAAGTTTGTCTAATCCGGGTGAGACATTATGAACAAAACATGTACAACTGAAAACTTTAGGGGTGACATGATACAATGGTTCATGTGGAAAACATTGGAATAAAGAATTTTGTTGTTAAGAGAAGAAGACATCCTATTTATTAAAAAACAGGCAGTCAAAACTGCATCTCCCCAATGATGTACTAGAACTTTGTTATTAAGCATCAGGGAGCGTGCAGTCTCAACTAAATGTTGATTCTCTCTTTTTGCTATTCCATTTTGTTGTGGTGTGTGTGGACATGTGGATTGATGCAGAATACCTTGTGAGGATAAAAACAAAAGCAAGAAAATATTCTTTGGCATTGTCACTTCTAAAATTTTTAATTGTTTTGCCAAATTAGTTCTTAATCTCATTAAAGAAGGACTTGAAGATGGACACAAGTTTAGATCTCTCTTTCATTAGGTAAACCCAATTACATCAAGAGTATTCATCGATAAAGGTTACAAAATATCTGAAACCAAAGATGTTACTCAGCTTGGTCCCCAAATATCTGAATGAATGGTAGAGAAAATCGAGTTACATCTTTTTTCAGTTTGTCTAGGGTAAGAGATCTAACATGTTTGCCTAATTGGCTCGACTCTCATTCTAACTTCTGAAGTTGCTCGAGACTAGGAACCATGATCTTCAATTTTGCCAAACTAGGGTGACCTAAATGGTCATGCAAAAGTTTTGGAGAAGAGGTTGCAAAACAGGACACAAATGAGTTGGCTTCGAGATAGCAAAGGCCTTGTGATTCAGGCCCAACGCCAATCAGATGGCCCGTACCACGTTCCTGTGTGACAAAGGAATCAACATCAAATGTTATTGAACAATTTAAGGAGCAAGTCAATTGGCTCTGAGAAATTAAGTTATAAGGACAATTTGGAATGTACAAAACATAATTTAATTTTAAGGAAGAAGAAAGGGGAACTTGACCAATACCTAGAGAGGCAACTTTGGACCCATTTGCTAAAGTAACAAGATGAGGAAATTTAGGTGGAAAGATAGATGAGAACAAAGAGTTATTACCAAAAATATGATCAGAGACACCTGAGTCAATTATCCATGGATTTTGACCTTCCACAAATTGAGAAATACAAGTTGTTGAGAGACTTAGCACTGAAGAAGACTGGACTTGACTGATGGATTTCAGCCTCAGATAGTCTTGATACTCCTCAGTCCTCATCAGAAAATTTGGATTTTGATTTTTCAGATTTGGTGACATGTGCTACGTTGTCAGGATAACCGTGCAAGGCATAACACTTCTCTTGAGTATGACCAGGCTTTTTGCAATGTGTACATTGAGGACATCCACCTCTACTACCGCATCCTCCTCTATTTCCACGACCTCCTCTTCCACGAGATGCTACCATGGCTGATGTTTCAGTAGGTGCTGCTGAAGTTTCAACGGCTACAACTGAGTTTTCACCTTTCACCGAGGTAGGAACACGAAGAAGCCTAGTTACTAGGCTGTACATTGAAGGAACTTGCTCACCCGCTAGAAATTGGTCACACACATGATCAAAATCTGAGTGTAGAGTCCACAAAATCACCACCATGAACAGCTTATCGAGTCTTCTTTTCATTTCTTCCAGAGTCATGTATTAGGAACTTCTTTAATTCTTCAACAACAGTTTTGGCTTTCGCTATGTGTGAGGTCAGATCGTGGTTAGTATGTTTCAGAGAAAATGCACGGTTTTCAGAATCAAATAAACTCTGGATGTCATTAACAAAAATATCTTGAGCATTTTTCCAGAAGGAGCAACAAATCTTGAAGGATCTGAGTATTTCCAACACATCAGGTTCTACTGATTGCCATAAAACAGCGCACAGTTGGAAGTCAAGTGTCTTTCACTGATTTCTGCTCTCTGCTGGAATTTCACTAGCTTCTTTCTCCAAATGATCATAGTGCCCTTGAACAAGAAACCATAACTCAACCGAGGCAAACCAAGATAGATAACTTTTCCAGTTGAGTTTTTGAGTAGTAATATTAGGAGTTCCAGAGAGAGGATAAGGAGTAGAAGTGAGAGTGAAATTCGTAGATGTCATTTTGAGAAATTAAGGGCAAGTATCTAGTAATAGACGCGTAGCACAAAATCTAAGGAAACAAATATAGGTCAATGGGCAGAGGTGTTTAGACTCACTGTCCTAACAACACCAAAATGACGATCGGAGGGCTGAAACAGATCTGGGTATCATTGGAGATAGTGCGGCAGCGGACGGCGGTGCTGGTGGACAACAATAGATAGAGACGGAAGCGCAAAGCGGGATCGTCCCACTTTGATACCAACTTGAAATGTTTGAGTATGAAAATATGCACACAAAAAGGATCCTCAACCTTAGTTTGCTGAGATGTGTGTGTGTGTGTGTGTTTGTGTATGAGAATTGACAGATAGTACAAAAAATAGTTCATGATGAAGGCCACAACCTAGCCTATGGTGTTAGATTACAGGACAGCTTCTATAACAAGCTAGAAATATAAAACATTATTAAACAACTTTGACAGGTATAATCTTTCACTGCTTCCCCTTTCTTTTTTATTTTTTATTATCAAGATAAGATTTCTCTTTTTCTTATCATTTTAAAATTGTTTTTTTTAGTGGGAGTGATTCTACCATACCATAGCTAGTTTCAAAATATTCTTAATCATATGAGTGTGAAGAGTACCATGTGGCAGATTGTATTTTATTTTTATTTTTCTTAAAAGATGGATTCAGTGGATTTATATTAAAATTCAGTCTAAATCTTGCTACTCTAACTACGGTTGTGTAGTTTTTAAATAAAGAACTATATTGGAAAGCATGGGTTGGGAGTTATGTTTTGTTCCATTCATTTGGTTAATAATGATATTTTAAATTCATATTTTCTTGCCTTTGGAATATTGTTTATATTTCTTTGACCTAGTATTGTTAAAGTTTCGTTTTTTATAGAGTTCATATTTTTTATATTCAGTAATAAAGTGGCATCTTATTTTCTTACTATGCTAAATTTGATGCTGTCAATCACGTGGAACACAATGGTTTTGTGTTTGCAGGCGACAACCACCGTTAAACCCATACAAGTTGAAGTGTGATAAAGAACCCTTGAACTCTAGGTAGTTCCTGTTCATAATTTCATTTTTTAAAAATTATTGCCTACTTTTACTTCATGGTTAACCATTAGTACCAGGCTCTATTGGGGGAATATTTTCATATTGCACTGTTAAAGTACAAAGAAAATGTGTGTATGACGTTCTCCCCTTCCTCACCTTGTTATCGGCTTATGTTTTTGTTTGGAGGAAGAATTCTCTAATTTTTTCCGAGATTCTATAAACCTTCATAGATTTGTTCTCTTTCTCTCCAGATTTCTCAGCACTCTTTCTTGTGGTTGTGGGTTTGTAATTCTACTCTAAGGTGTTAAGCCTCAAGGTTTTATTATCTTGTATATTAACATGTAAAATGCAATTATTATTTCTCAGGCTTGGGGCACCAGACTTTCATCCCCAAACACCAAATTGTCCTGAGGAGACTCTGACCAGAGAATATTTGCAATCTGGATACAGGGATACAGTTGAGGGACTTGAGGTATGCCGTAAAGTACTTGTTTGGGAAGCTCAAATTTTGAGAAGCAAATGTTTTTTTAAATCTCTCAGGAAGCTGATAAAAAAACAATTATTTCTTCAAAAGCTAAGAAAAGATGCACTAAATTTTACTTTTGTATTTATACCAAATCAGATATTTTAGAGTAGCAACTTTGCTTATGAGTGATTGGATTATACTTCTCCAGTTATCCATTTATTGTGTTTTCAAGATTTGAATATTTTATGCTGAACTCTCATATTTCTTTTGTAAATATTTTTGATAGAATATGTCCAGAGTAACTGAAAGTTTGGAACTGTAATCTGGCATTTTCTTGAACTTGGTATTTTTTTATGATCAAATTCTTTGAAAACGGAAATAGTGATACGGGCATGAATATATTTTTCAGAATCATAGATCTCCTTGATGAGTCACTGAAATATAGAAATCAAGTTGTGCTCTTGTTAGAATAGATTATTTTTAACTGTTCAAAACTGCCTTTTGTCCGGTATCTCTGCTATTTTTTGACCTTGTCTTGCTTTCTGTGTCTGTTAGGAAGCTAGAGAAATTTCACTAACTCAGGTTCCGCATTTTAACAAGAAAGTTGTTCTTAGTTGCAAAGAGGTGACTCTCTTGTCTGTTGTCTATTTGGAGATATTTATGTCCACTTTATTTTAGTTTGTATGTCTGAGCTCTGAATTTACATCACATCTTTTTTTTGGGTATTCATTGAAAGCCTATGTGGATATAGGTAACTTGTAGTTTTTCATCTAATTTTCAGGCTATTAGAAAACGTCTGAGGGCCATCAATGAATCTCGTGCTCAGAAGAGGAAGGTGATGATCTTTAATCTGTCTTGTGATCTATTTATCCTTGCCGAACATTCTACTTCTATAGTGTATCCATATTTGTGTGCTTTCTTCTAGCTTATTTTGGATTTAGTGCAGTCTACCTATCTCAGAAGGTGTTTGATCTACATGATTGTTTGATTTGACAGGCTGGTCAAGTATATGGTGTGGCTCTTTCAGGATCACAACTAGGCAGGTCTGGCATTTTCCCTGAACTAAGACCCTATGGTGAGGACTTCCAGAAGAAATGGATTGAGGTATACCTGGTGATAGTCTTGCTCCTACTGTTTTTTTCTTTGTGCAACTTCCTGTATACCAGCTTTTACCCCTCACTTTTGTTTCTTATACATTTTTAGTAAATTTATATGTAGAAAGCACAAGGTTTTAAATGGTTAGTAACTTAGTAGTAGATGGTTGTAGTCTTGATGCGACCGGCTGCAGGATGTGAATATGGAAGGATCTGGAATGTTTATATGCTGAATTATCCTGGACTGGCTTAGCTATTCATGGATTTCTCAATTATGGAAAACTAATATATATTTACATAAGTTACTGTGGCCAATTCCTTAGGCCTGTTCGGGGTGCTTTCCTGTTTTTGGTTTCATTTTTTTTTTTAAGACTACAACCAGTTTAGGGTTTGAGTTTCTAGTTTCAGTTTATTTTGGTTTTTCGTTTTTATAAAATTGTTTCAAATTGAAATATGCTATTAAGTCAAATATTTTAGTAGAGACAAAATTCCATGACTGCCACGGTTTCTTGAATCTCCTATCATCAAACCATGTTGAGCTCGGGTCCTTACACTTCATAAGAGATCAAGTTATCTATGATTCTCCTTACACTTATTGTTTTTTTTTTTTGGAATGGCCAAAAGAAAATATAATCAAAACACAGTTTCTGTACAAGGTGTACAAATAAAATGCATGTAGCTACTAAGACCAGTCAAGGAATTCCTAGTCCTAGATCATGACAAGGCTACAGACTTTGAATTCCACATATAACCTTAACTTGAAGACATACAAACATTGTAAAATGCCCACATTTGCTCCATGTAAATTGCACCCCCAAATCCAACCACGTAAAACCATTCTCCTGCACCTTGGACTGCATAATCTAGCTTTCTTCCAAACACCAACTGAGTTGACTCAAATTACCTCTCCTATGCACATGACCTGTTGCTTTTACCATTTTTGCAGCTGCAGGCATGCTGTAGTATCCAACCACCACACAACACATCTGATAACATCTACAAAGAAATTCCACCAATCAAGAATTCACAACCAAGCTTAGAAATGTAACTAACATCTGCTTCCATCATACCCGCTGCATAAGAATGTCCTTGCCTATGAATATTGTCCCTTTTCATTAGCTGTTATAAGATTCTTGGCAATAAGCTTGCCTACTCATATCATCTTAAAATCATGAGACAACTCCACTTCCGCTTCATACACTTCTTAGTGCTTTTGATTAAAGAAACCTGTTCCAGTCAATGTGTGGGCTGTAGCATAAGCAACTCCTAAAAATAAATTAGACACAAAATGTTATTATATTTTATCAAAAAGGAGAAACAAAATTCAAAATGAGCCGTGATGGATATCCCAAAAGAAACATTATGGAATTCAACAATAAGACAAGGCATTCTTTACCTTTCTTGACCTTTCTCATTAACAAAATTCAATGAAAAATTGTAGATCCAGCTTATAACATGCAAGCCAGTAATAACAAACACATAAAACATTGCTTTAAACAGAGAGTTTCTTGCCCACAAGAAAACCCAACAATGGACAACAAATCATAAACAAAAATAGCAAGATAGTGAAAGAAAATCCCCCAATTTGGTACAAAACCAAAACCTCCAATTTACTTCCCCATTATGCACACCACACACGGTGTGAGAAAGAGAGACAGATCAGTGTACAAGAGGGTTAAGAGTACTCACGGATTGAGAGAGGGAGGCATTGACAGGTGATTGGCGATTGGCGGCGAGCGACGAGTGAAGTAGATCAAACGACACAGAGAGAGAGAGAGAGAGTCTGAAGTACGAGAGGGTGGGAGACCAGTGTGAACAGAGCAGTGTCGAGCAGGAAAGAGAGAGCAGTGGCGAGCACAGTACCATTGGGAGACAGAAAGCGGCGGGGAGGGAGGCTGGCGCAGATCGTCAAGGTAGGGACGGGAGAGCAGCCTAGATCGTTGAGGTAGGGATAGAAGTAAAACTAAAATAATAAAACATAAAACCTATTTTTATTGTTTATAAAATGGGGTGACAACTGTTTTAAAAACAGATAAAAATCCAACTCGCAACCATTTTAGCCGAACACCTTCCATTTTGAAATAGCATCTTAAAACGGAAAACAGAAAACTCAGCCACCCCGAATGGGCCTTAGGTTTTTTGTTGTTGATGCTGACAGGTTGTTGTGGCTGTAAAATCCTTAAATACTACTTTTTGAACATTACTTGGTGTTTCAATGTATCTTTAGTAACTCAGTTAAAATTTTATTACAGGGTTTATCTCAGCAGCACAAGCGATTACGCTCTTTGGCTGATCATGTTCCTCATGGTTATAAAAGGACATCACTTTTAGAGGTTCTTATCAGGAATAATGTTCCATTGCTTAGGGCCACCTGGTTTATCAAGGTTACTTACCTCAATCAGGTAGGGATGCTTCAGTGTTGGTATATTTACCAATTATGATGAATTGCTTTATATTTTTTAATATTTGTAGATTATGATTGGCTTTTTCAGGTTCGACCTGGTTCTGTTGGTATTTCTTCTGGGGCTGCTGACAAGATTCAGCTGTCTCGCTCTGATGTTTGGACCAAAGATGTTATCAATTACTTACAAACACTTGTGGATGAATTTTTGTCAAAGAATGCTTTGCATTCTGCTTCTCATGGTCGAGAGCGATCACCACAAATGTCTTATACTGGCTCACTGCAGAATAAAAACGATCCATTATTATCTGTTTCTGATGGTGAAGGGCCATCCTTACATTTTAGATGGTGGTATATTGTGCGGCTTCTGCAATGGAATCATGCTGAAGGGCTGCTTCATCCTTCTCTTGTTATTGATTGGGTGTTTAATCAACTACAGGTATTTACCTTCTACATCAATTTGCATTTAATAAGTACAACGTGCTCATGAAACTCAAAGTGTGATGTATATATCTTTCACTGATTTCAACCTTCATGTTTTGATTTCTCACATTTCTGTAATTTTCAGGAAAAAGATCTGCTTGAGGTTTGGCAGCTGTTATTGCCTATAATATATGGTTTTTTAGAAACTATTGTCCTATCTCAAACTTATGTACGCACTCTTGCTGGATTAGCTCTTCATGTCATTCGTGATCCTGCTCCAGGTGGTTCTGATTTAGTAGATAATTCCAGGAGGGCATATACAGCTTATGCTGTGATTGAGATGCTCCGGTATTTAATACTTGTGGTGCCAGATACTTTTGTTGCTTTGGATTGCTTTCCTTTACCATCCTCTGTAATTTCACATACAATGAATGATGGGAATTTTGTACTAAAATCAACTGAAGCTGCAGGGAAGATAAAAAATAGTTCAGATGATTTTGGTCACATTATTTCATGTATTCAGAAACATACAGAAGATCTTGTGAAGGCTGCAAGCCCAGGCTATCCAGGTCATTGTCTAGCTAAAGTTGCCAAAGCTTTGGATAAAGCTCTTGTGCTGGGTGATTTGCGTGTAGCATATAAATTTCTTTTTGAAGATCTTTGTGGTGGAACTGTATCTGAAGGTTGGATTTCCAAAGTCAGCCCTTGCTTAAGGTTATCTCTGAAATGGTTTGGGACTGTAAATACACCCCTTATATATTCTGTGTTTTTCCTTTGTGAGTGGGCAACTTGTGATTTTAGGGATTTTTGCAGTACTCCTCCTCGTGACATAAAGTTTACAGGTAGGAAAGATCTTTCCCAAGTGCATATAGCAGTTAGACTTTTAAAGATGAAGATTAGGGATGTGAAGATTTCGCAAAAACAAACAAATGAAAATCACAGAGCCAGTCATCTTGCGAAGCATTCAAGTCAGCGGCATAATTGGAATTATGTGGGTAATGTATCCAGATTGAGATCTAGTTCAAAGAGTACTGGTTCTTCAGTATTTGAAAGCCCAGGTCCTCTGCATGATATTGTAGTTTGTTGGATTGATCAGCATGTGGTGCAAAAAGGGGAAGGTCCCAAGCGCCTAAATCTATTTATGGTCGAACTCATACGTGCGGGCATCTTTTACCCCTTGGCATATGTACGCCAGCTAATAGTGAGCGGGATCATGGATGTGAATGTAAATGTGGTTGACCTGGAGCGACAGAGGAGACACTATCGCATCTTAAAGCAGCTACCTGGATGCTTTATCCATGATGTTCTGGAAGAATCTGGGATTGTTGAAGGGTCGCAGCTCAAAGAAGCCTTGCAAATTTACTTGAATGAACGCCGCCTCATACTTCGGGGTCATCTGAGCGTGTCCTGTGGTTCCAATTTATCTGCTCTGAAGAAGAAAAAATATCCAGCTTCTACAAAGGATGAAGTTTTTGCAGTGCCAATTGATCAGAGAAATGTTATTTCTACCACAATATCTTCTAAAAATGCGAAGGATACTAACATCGAAGAACTAAGAACAGCAATCTCAGTACTGTTACAGCTACCTAATTGTTCATCTAATTTGAGCACTACAGGTGATGAATCTGAGGGCAGTGATAGAAGAGCCATTGGGTCTCCGTACGGCAAGATTGATCCAGTGGAGGGTACACCTGGGTGTGAAGAATGTAGCAGAGCAAAGAGACAAAGGTTAAGTGAGGAAAGAAGCACATTTGTGCAAGGGCATTCTCCAGTTCAATCTGATGATGACGATACATGGTGGGTGAAAAAGGGAATGAAATCCCCAGAGCCTCTCAAAGTTGATCAACCACAAAAGTCAACCAAGCAGGTCACCAAGAGTCGGCTGAAGAATGTGCGTAAAACTCAGAGTCTAGCTCAACTGGCAGCTTCTAGAATTGAGGGTAGCCAAGGGGCATCAACTAGTCATGTGTGTGGTAACAGGGTAAGCTGTCCTCACCATAAAACAGCTATGGATGGAGATGGGCAGAGGTCTGTCGATAGCATCCGAACAAGTCATTTTGGGGATATAGTTTCCATTGGAAAAGCACTAAAGCAGCTACGTTTTGTTGAGAAAAGGGCAATAGCAGCTTGGCTGTTGACTGTTGTTAGGCAGGTAATTGAAGACGTGGAGAAAAATATTGGTAAAGTTGGTCAGTTCAGCAAGCCTTTTCCTGTTGTGGATGATAGAGGCTCAATACAGTGGAAACTTGGTGAGGATGAACTTTCTGTGATTCTTTATTTGATGGATATCTCTGATGATTTGGTATCAGTTGTCAAATTCCTCCTTTGGTTGCTGCCAAAGGTTCTTAATAGCCCCAATTCTACAATTCATTCTGGGAGGAATGTGGTGATGCTACCAAGGAATGTGGAAAACCAAGTTTGTGATGTGGGAGAGGCTTTTCTGCTATCATCACTCAGAAGGTTTGCTGCTCTGCAATCAATTACATATATATAGTTTTTCTTTGATGTCTCTTGGAAAATATTTTTATTCTGCTTTTCCCCCTTCCAGCATTTCCTTCTAATTATTGTGATGTTTGCTTCCTTTTTATTAGAGTTGTCTACTCTTAATACTTTTCAATGGCTTAGCACCTTTTACATGTGATATAATACTATGCTTTATAGAGTACCTGGTAGTAAGCTGTTATATCTGTTTCTTCTTATGCTCCCTTTTTCTCGTCATCTTTAATCAGGTCAGTATTGATTAAATCTGCATTGCTCTCATCTACTCAGGAGTCTGGATTGCCTAAATAAAATCCTATATTTACAAATTAACAACATAAATACTTGTTCTTGTTATAAGGGGCCAGTTTTTAACATAATTATAGTACAAGTTTGCTGAACATTCAGTGAGGTTTCTACAACTATTATGGGCCATCCTCCCCCTGAAATATTTATCATTTTTGTTATTCAACCAGTGCTTTATGAGTGGTGTTTTCCTTTTGTTCTTTATGCTTGTGTTGGACAGTTTCTTTATGGATGTTATGAGATTTTATCAAACTTGACTTCTGCAATGTATTTGATGTAGGTATGAGAACATTCTTGTTGCAGCAGACCTTATTCCTGAAGCTTTGTCATCTGCAATGCATCGTGTTGCCACTGTTATTGCATCTAATGGAAGAGTTTCAGGGTCAGGGGCCCTAGCTTTTGCTCGGTATTTGTTGAGAAAGTACAGCAATGTGGCTAGTGTCATTGAGTGGGAGAAAACTTTTAAGACTACATCTGATGCTAGACTTTCCTCTGAACTTGAGTCTGGTAGATCAGTGGATGGAGAGTTAGGTTTACCCCTAGGAGTTCCAGCTGGAGTAGAGGACCATGATGATTTTTTCCGTCAGAAGATAAGTGGTGGTCGGTTACCATCCAGAGTAGGTGCAGGCATGAGGGACATAGTGCAGCGTAATGTTGAAGAAGCGTTCCACTATCTTTTTGGAAAAGATAGAAAACTTTTTGCTGCTGGTACACCAAAAGGTCCTACTTTAGAAAAATGGGATAATGGATATCAAATTGCTCAACAAATAGTTATGGGCTTGATTGATTGCATAAGGCAGACTGGTGGTGCTGCTCAAGAAGGAGATCCCTCTTTGGTCTCTTCTGCTGTTTCTGCGATTGTTGGCAGTGTTGGTCCAACTTTAGCAAAAATGCCTGATTTTTCATCTGGCAATAGTCATTCAAATACAATGCCCGCTACAAATGCATTGAACTATGCCAGATGCATTCTGCAAATGCATATAGCCTGTCTATGCCTGCTTAAGGAAGCCCTGGGAGAACGCCAAAGCCGTGTATTTGATATTGCTCTAGCTACTGAAGCTTCTAATGCTCTTGCAGGTGTTTTTAGTCCAAGTAAAGCATCTCGTTCTCAGTTTCCAATGTCTCCTGAAGCCCATGATTCTAGTAACACCATTTCAAATGATATGGGAAGTAACTCTAGTAAGGTTGTGGCAAAAACAACAAAAATTGCTGCTGCTGTTTCTGCACTTCTTGTTGGTGCAATTATATATGGTGTTACCAGCCTTGAAAGAATGGTGACAGTTCTCAGATTAAAAGAGGGGCTGGATGTGGTACAATTTGTAAGAAGCACTAGATCCAATTCAAATGGAAATGCTCGTTCACTTATGGCTTTTAAGGTGGATAATTCAATTGAAGTTCATGTCCATTGGTTTAGATTGCTTGTTGGAAACTGCAGGACAATCTGTGAAGGGTTAGTGGTGGAACTCTTGGGTGAACCATCTATTATGGCTCTTTCAAGGATGCAACATATGCTTCCTCTAAATTTGGTCTTTCCGCCTGCCTATTCAATATTTGCCTTTGTTAGGTGGCGACCTTTCATTCTGAATGCTACAGTTCGTGAAGACATGAATCAAATTTATCAGTCTCTTACAATGGCCATAACTGATGCAATAAAACATTTGCCATTTCGAGATGTATGTTTTAGAGACTGTCAGGGTCTTTATGATTTTATGGCTGCAGATGCAAGTGATTCTGAATTTGCAACCTTGCTAGAGTTTAATGGGTCTGATATGCATTTAAGATCCACAGCATTTGTTCCCCTACGCTCTAGGCTTTTTCTGAATGCCATGATTGATTGTAAGATGCCACAATCTATTTATACGAAGGATGATGGGAGCCGAATGTCTGGACCTGGTGAGTCTAAAATTAAATTCACAGATAGTGAGTCTAAGCTTCAGGATATGCTTGTGCATGTTTTGGATACCTTGCAACCTGCCAAATTTCACTGGCAGTGGGTTGTACTGAGGTTGCTTTTAAATGAACAAGCCCTCATTGAAAAACTGGAGAATCGTGATGTGTCCTTGGCTGATGCTATAAAGTTGTCCTCACCTAGTACAGAGAAGGCTGCTGCTGCTTCTGAGAATGAGAAAAATTTTATTCAAATACTTCTCACAAGGTTACTGGTTAGACCTGATGCTGCACCCCTTTTTTCAGAGTTGATTCATCTCTTTGGTAGGTCACTAGAGGATTCAATGTTGTCGCAAGCTAAATGGTTCCTTGCAGGCCAGGATGTCCTCTTTGGTCGGAAGACCATTAGACAAAGGCTACATAACATTGCTGTGAAAAAAAATCTTTCTGTCAAGACCCAATTTTGGGAGCCATGGGGTTGGTGTAGTCTGTCTACTGATCCATTAACTGTCAAAGGGGATAACAAGAAGTTTGATAGCACATCCCTTGAAGAAGGAGAAGTTGTTGAAGAGGGAATGGATTTGAAAAGGTGCCAGCTGCAAGTGACTGAGAGGGCTCTTATCGAGATGCTTCTTCCTTGCATAGATCAAAGTTCTGACGAATCCCGCAATTCCTTTGCAAGTGATATGGTGAAACAGTTAAGTTATATTGAGCAACAAATAACTGCTGTTACAGGTGGAAGTAAATCAGTAGGAAGTGCTCCTCCTGGAGTTGAAGGTCAGCCGAATAAAGTAAATAACCGCAAAAATATGAGAGGTGGAAGCCCTGCATTAACTAGACGACAAACAGTTGCAACAGATTCTTCTCCACCGTCTCCTGCAGCCCTACGAGCTTCTATGTCATTACGTTTGCAGTTGCTCCTGAGATTTCTTCCTATTCTTTGCACTGACAGGTAATAAGACTGGATATTGCAATGTGTAATTAGCTTTCTCGTTATAGGATTCTTGATGCACTTGTCAAGTGAAAAAAAAATCCACGATCCTTCTCTGTTTTCTTGAAATGGAGAAAAGATTGGAGCTAATTGCAATATGATTATTTTCAGTTTCAGAAATACAATTCTCTATCATGTATTAATTGTCGTGGGTCCCAGCTGCTGCTACTGAAAGTGGTAGTTTTTCCTTGTAACAAAATAGATTTGAGCTTTAAATTTTGAGAGTTGGCAATCAATTAAATGAGTCATGACCTCTCTTTAGAAGTGAAAATACGGATATTTTTGCCATGAAGGCATTGACCTTGTTTTATTTTTTATGCGTGCTTGTGATGGAACTATTAAATCCTAGGATGTTAGAAGCTATTCTTGAGTTCTATTTATTTCAATTTTGCAGGGAGCCATCTGTGCGGAGCATGAGACAATTTCTTGCCACTGTAATTTTTCGTCTCCTTGGTAGCCGGGTTGTGCATGAGGATGTGGACATTTCGGTGAATGCTGTGCCATCTCTGTCGATAAGGGAGGCGGAGTCATCTTCTGAAGTTGCTTCTGCTGCTTTTGTGGATTCTTCTTCTGGGAGTCTGTTTGATCGTTTGTTGTTGGTTTTGCATGGATTATTAAGTAGTTATCCTCCAAGTTGGCTTAGGGCAAAGCCTGTTTCAAAGACAATCAGTGAACCTACGAGGGAAATTTCTGGAATTGACCGAGAATTGTTGGAGACTTTGCAGGTTTGTTCTTTTAAGCCTGTATGTGTATAATTTTTTGGTTCTAGTCACTTAGCCCTTAGGTGGCTTTTCAGTGTATTACTACTGTAATTCTTTGAAGTTTTAATTCAATTAGTTTTTCTTTTTAAGGATTTATAGATGCTGTATGCAAGTTTTTTTTGTTAATTGGATAAGGTGGCATCATGGTAAATACCAGAGATGTTTCTTCTAGGCATCTTGTATTATAAGATCTTCTGTTTTGCTACTCACCAGGAAGGACTGATACTTTAAATTTTTTATACCTTCCTGGTGTTAGATATAAACCACCATATGGTATGCTATTTCAACATCATTGAAAATTGTTTTCTAGTGTAATATATTTTTTTTCTTTTTCCATTGAGGTTTTTTGTCAACAAATGTGGGATATAATCAGAATTTGATAAATTCTAGACTTCAATTGTCTCCAATGAATAATATTGGAACTAAAAACTATTTTTGTTCACTTTCATCCCTCAATCGTAGAATGGCTTGACCATTATTTTGTTATGATGGCTTTTATGTTTCCCAATGGTTTTTTATAAGAAATCTATAATATGTATGTATATGTTATGGTGCATCATGCCATGCCCATGTCTTTATTTTTCAAGAAATTATGTATTCCATGTTGTACTGGTGTCATATCAGATATTGCTAGCCATATCCGTGCTCCATAAATCTCTACTAAAGCTCTACAATTTGGCAATGTTTATGTACATATTTGGCAATCTAGAAATATTAATTCAATCTTGTCGCGCTTGTCTAATGATCTGTTGTCTGGTGCAGAATGACTTGGATCGTATGCAACTGCCAGACACTATTCGGTGGCATATCCAGGCTGCAATGCCCATACTCATCCCCTCTATGCGGTGCTCTTTATCCTGCCAGCCACCATCTATTTCCAATTCCGCTCTTGTTTGCCTTCAACCCAGCATTACAAATCCTGGGTCTAACTCCAGTAGTTCAACTATTCCTCAGAGGAACCCAGTTCTATCAAGGGTTGCTTCTAATGCATCAGGGAAATCAAAACAACAGGACAATGATTTGGAAATAGATCCTTGGACACTCTTAGAAGATGGTACTGGATCTTACTCTTCGGCAGGTAATACTGCTAGCATAGGAAGTGGTGACCATGCTAATATTCGAGCCACCAGCTGGCTTAAAGGGGCTGTAAGGGTGAGACGGACGGACCTCACATATGTTGGTGCTGTGGATGACGATGGTTGATTAACAAAATTTTGTTGGTTTGGCCTTTGGAGTATACAAAGCCTTCAGCTGACAGCCAGTGCAATTGCGGTTTTGTTATTTATATTTGATGCATGAATGGCATGAAGATACCATTTTGTGCTTGTCCTTCTTGTGTTGAAGTTTAAAGGAAGCTGGAGGGGCATTGTTTTGAAAAAAAAATGGGTGAAGGGATGGGTTCAAAACAGCTCATGCAATGGTTCTGGCCTGGTTGACTACTAACCTGCTTGATCTCCCCCATCTGGATTGTGCACTTGCTCAGTTTAGAGGTTTGTTGTATATATAGGGATGATTGTTTTACTTTACCAATTGGTCTGAGTAATATAGTCTCAAGTCATATTTGTAGCTGTTTCATGCTCATTTTTCTGTCTAGTGAACGCTTGTGTGAGCACTGTCTATAGCTTTTAGGGGCACAAGCCCACACAGGCGCAATCAACCATTGTTTCTTTTCTCATATTCATATTATCAAAAGTTATGTGGTTGATCTGGAGCATCACCATGAGCTGCAGCATTCAATTATTTTGTTGGCAATTCTGGATATAGTTTCATTTCTACCGAAATATTTTATCATGAAAGTAAAACTATGTATGAA

>GmMED12-3

CTTTAACTAAAAATTATTATAACTAGCTCCTCCACCTTCACTGTTCTTCCATTGTGATTCACAGAAAACACCACCAACTCAACACAATCATCATCGTCACTACCAATTTTTTTTAATAAAAAAACCAAAAAAAAAATACAAAGAGTCAAACCCTCGACCACACTACAGAGACTCTTCTTCTTCTTTGTTGCTCTGTTTCTCTCTCTTACATTCTCACTCTTTCCGTCAAAGTTTGCAATTTTTCTTCCCAGGTAATGTCCTTATCATTAATCTTCAATATATTGCAATTCCCCCATTATTATATTTGTTGTTAATTTTGATTTTTCGGAAACCCCTTTTGTTCCGTTTTGCCCAATTGTGAATCATAAACCCTAGTTTTGCTTGTGCTGGAATATCCACAATTTTCAGTTCCGCAATTAGCTTGCGCCGGCATTTCCAAAGTTGGGTAAGAACGAAACAGTAGCCGGAATTTCGTTCTCAAAAACCCTAGTTTTGGATCGTGCTTCGGAGTTCTAATTGTGGAGTTTTTTTTTTTTTTTTCTCACATTAGATTTGATTTTGTTGGATTGGGTGGTTGCTGTTCAGGTTTTTGTAGAGTATAGGTTGGATTTGACGTTAAAGTTTAGATTTTTGGACGATTCAAGTGGTGGGTTTTTTCTCTCAGTTTTTGCCTCTTGCATCTGTAGAAGTTTCATTTGGTGCCCTTGTTAGTTGGAGCAGGGAAAGTGAGGGTAATTTTTATTTCAATGAGTTTTGCAGTTATGCTTGTGGATTGGAAATGATTTGGTTAAATTCTGCAACTTTGTATTGTATCTCGTGTTATAAGTTATTACTGTTGGTTACGGATTCGAGACTTGAAGTTATGATTCTGGCAACGGTCGACCACGTGTTTATATGCGATAGGACTTGATATAGGTGTGTTTTGGTATATGTAGATACATTTTAATGGAGAGTTTCCAATTTCAATGGGAATTGAATTCTCTTGTGTAATTGTTGTGATTGTGTTTGTGTTAGTCTGACTAGGAGATTCCTTAGGGTAGAAGTAAAGCTTCTAAGTTCATTGGTGTGCTTACAGTTACACTATTTTTGAAAACAAGACTTTTATATGGTTGGGAATGGGGTTTTGGCGTCAACGACGGTTTCAGAACATAAATGTGAAAAAAGTTGTTCAAATTTTCTTTACCCCTGCTGTTCCACATCTATGCATTGGAAACTGGACTTAGTGTGGACAAGAGTTTAGGACTCTGTTTTCTTTTATTTTCCAGAATATTTTATGTTCTTTGCATCTTAAATGAAACATGCATAGCGTGTTTGGATCATGCCATGTTCGATTCAAAATCGAGTCCTTTCCATGTTTGATGCAAAAGCTTAAAATGACAGCTTCTCTTTTTCCTGTCTGATACTCACGTGTTGGTTGTTGGACACGAAGCCAAACACCCAATTCAGACTATTGGATTTTTTTTGGAGCTTTCATATTGCCCTTGAGTTGCACTCATCTGATGCATTCTGGAATCTGGATGTTAGTTAATGATTCTTCTTATGGTGCATGTTATGGATTTGTTTCTTAATTCTTTATCCATTTGTGTTTTACAGGCCAGAATAGTACCACTCTGCCTCATACATCTGGGGTCCACAAATTGTGGGTCTCATGTTTTGGGGTATTAGGATTTCAGTACATATGTTTTATAGTGGTTGAAGACAGTTAACACATAGATTTGGTGCTTAAGGTCATTTTCTCTTATTCTCTCTTCAATTTATGTGTCTTTTTTTTCCTTTGTCACTTTTTTAGGATTTTTGTTGTAGTTTGCTTAAATGCTCTGGTTGGAAATGACACAGCACATTTTCAATGATGCAGTTTAGCTATTACAGCTACTTGTAGTGGTTTGATGCTGAGGGCAGTATGCTGGTTATGATTGGGGGAAATGCAAAGGTATCATGCTGGCAGCTGCACTAGTGCAGTTAATAACAGTGCAATTGGTGGGCCATCCACTAGGGACATTGGAAGAACTGATTCATCTTCTTTGCCGGCTAACTTTCCTGTGAGTTCAAGGTAAGATACTATGTATGCCAACATTTCTTTGCTGAAAATCTTATTATTATTCTTTCTTTTCAGATGTTGTTATTGTAGTACATGTGATTATGTTACTGCATATTCCAAATAATTGTTGTTGTGATGCAGCTTCTGGTGCTTCAGTCCCTGTTATCTAATAGCTTTCTTTATTGCAACAGTGATTATTTATTTACTTGACGTCCTTTTGAAACCTGAAAAACTCCACTTCAGAGCTGGTTCATTTATCTTTTGACTCTTGAGTAATCACAATATTCTAGTTTTTTTGGTCCTTTTGGGTCATCATTTCTGTTCTTATGCATTTAAATGATTGAGATATCTCAGCCTAGATACATTAACATGGATTCTTTTCTAAAAATACTTTATGTACATGAGCAATGTTTATAAATTTCTCATTGGTAAATGACTAAATTAGTGAAGGATGATCATAGTTTGAAAGAAAAATACTTTAATCATAATAAAAAACCAGTTACATATCTCTCTCGTATCCATATCAGAATTTATTCTCTCCCCAATGAACATGTCATAGAATTACATATTTTTCCCCAACTCAAGTTTGAATGTAAAAGAAAAAATAATCAGTTGTCCTATTCAAAAGAAATATTGGGTCTACTTTATTGTCTACTATTTACAACAGCTTTGAAGATACAGAGGAACATAGAGCTGTTTTAATATTAGTCCTTTGTTCTGTTTGATAGTAGATACTCCATAATAAAATGGTATATTATTTTATTTGTTCTGATAAATCATTTATTTGATGCTGTTAATCACTTTAAAAATAATGGTTTTGCTTTTGCAGGCGACAACCACCATTAGCCCCATACAAGTTGAAGTGCGATAAAGAACCTCTGAACTCTAGGTAGTTCATGCCTGTAGCAGAACCTGTTTCCAAATCGTTCAATTTTGTTAGTTCATGAGTAACAAACATCTTAAGTTTTTCTGGAGTAAAATTTGCATGTACTCGGTTAATATTCAAAATATTGTTTGATGCCCTTGATCGTCGGTATCTGTTTTTGTTAGTTGTTTTTTTTTTTTTTTTTTTGGAAGGCAAAAAATATATATATATATATTATTAATAGGCAAACCAGTACCAGTGGTACCAGAAATAGAGATTACAAGGCACCTAAGTGCTGCCCTCCAAAATAACAACCCACCCTAATATAACAAGAGTATGAAAGCCCAGTCAAACTATCCAAAGGAATTCGATATATTGGAAGACCAATGGTTGAAGCTAATATTGAAATCTTTTTCTTTAGCCTTTAGCCAGGACCAAACTAATGTTTTTGTTAGTTGTTATTCAATGGTATTAAGATTGCTATGTTTTAATCCTGTTAGTACTCCTAGTTTTTGCTTTAAATTTCCTTTTTGTCTTGTTGTATAAATTTTAAATGTCACTGTTAAATCTCAGGCTTGGGCCTCCTGACTATCACCCCCAAACACCAAATTGTCCTGAAGAGACTCTGACCAGAGAATATTTGCAATCTGGATATAGGGACACAGTTGAGGGGCTTGAGGTATATTTGTTAACTTCTGTATTTATATCAAATGGATAATGTAACGTAGCAATTTAGCTATGAGTGTTTGAGTTATAGCCATTGTTAGAATCTGACAATTCTTGATTTGAATCCTATGATTCAATATGATTCAGCTACCTAATGATTCATGTCATAAAATGAATCTCTAATGACTTTGTATCTTATGATAATCAATGAATATGTGCAATTTCCTATCATCGATATGAATCACTCGATTCAATTAATTTTTTCTTACTTTTCTTTCACCAATTCTCATGAAAATAATTGAAAATTCATAAACTTAAATTTTTAAATGAAACGGAATTGAAGGAGCGCTTCTGAACTTATCCAATTTGATCATTCTACTGCTTAGTTTGTCACCCTCTCATTCAATGCATGCACTAACTAGTAACTTTGTTTAATAAAACTTATGATTAAGTTGTTTTTATCTATTTCTAGTTTAAAATTATTACGTTGAACACTGTATATTTGGATGTGTTTATCATTATATTTGTATGTGCTATTTTCAGTCAATTTGAAAACAGCTTGGTTTTTTGTATCAAATTTCTTATTTGGATCTCCTACTGTTCAGTATGATTGAAACTATGCTGTAGGGAATTTGATATTCTAGTGGATTGGCTTCCCTTGATGAATTGTTGAAAATCAACTTGTACTTTCTAAAACTGATTGTTGTTTTTATAAAATGGTATTACCTTTTTTTTGTCTTCAGGAATCTAGAGAAATTTCTTTGACCCAGGTTCAAAATTTTGGCAAGACAGTTGTCCTTAGTTGCAAAGAGGTGAGTTCCTAGTCTTTGAGAGAGAATTATGCCCTTTTTTTTTGTATAACTATGAGTGTGTGTGGCATGTCAATTAAGCTAGCTTATTAGCTTGTCTGGCTAAATTATGTTGTTTGATAAACAAGCTTATTTTGCTACCTTATAGCTTATAAGCTATAAACTACTTCAACTAGCTTATAACTTATGAGCTTGCAGCTTGTTTTTTTTCATCCATTTTTTTCCTTACCATTTTAATGTACTTAATTTTAATAAAATATTTGATCTTTTAAAAAATTAAGAAGAATGTAAAATTAATAAATGAATCTAAAACTATAATTACCTAAGAATATATTTTTTGTCTACGAAACAAAATATCATTTATATTAAAAATTATATAGATGTCCTTTTATGTCATTTTGCATTTTTAGCTACTTCAATAACTAATTTATCAAACACTCGTAATACAATAAGATAGTTTATACGTCTTAAGCTGGCTTATTTGCTTTAAGCTAACTGATTAGTTAGTTTTGTCAACCATAGCCTATGCCATGAATATACTGGACATTTTCCATATTTGGTTTTTATTTCATCGGAAGTGTTTGTAGGTTAATTCTTTAATCTTTCATCTTCTCTCTCCAGGCTATTAGAAAACGTCTACGGGCAATCAATGAATCTCGTGTGCGGAAACGGAAGGTGATGACCGATGATCTTTATTCTGTTATTTTATTATGCTTGCTGAACAGTATGTAGGCCTCTAAAGTATTCCATTTTTTTTGTATTGTTTTATTTTGGGTCTAGTGGGCCCTACGTGCTATGAAGGTATTTGATCTACATGATAGTGTTTATTTTGACAGGCTGGTCAAGTATATGGAGTGGCTCTTTCTGGGTCACAACTTGCTAAGCCTGGTGTTTTCCCTGAACAAAGGCCTTGTCCTGAAGACTTTCGGAAGAAATGGATTGAGGTATTAATGAAAGTCTTGTTCCTGTTGCTTTACTCTTTGTACAACTTTCCTGTATAAATATTGTTTTGCTTGTTATTTGTTCCTGTTAGAATGTTAGTGTTATTGTTTTGTTGTTAGTGAACTTGGTTGGGCTTGTAGCCTTGTACTAGACCTAGTTATTGGTATAAATTATTAAGAGCATTAGGGATTGTAAAACACATCATTATTCAGCTTACTTTAGTCATTTTTCTCTCTTTTCACCCAAACTACACAATTTTAGCCTATTAAAATTGTAGGGTTTAGGTAAAAAGGATGGAAAAGAGAGAAAGACCGCTAATACAATGGAGATTGATATTAGATGTTACACAATACCTAGCCTAATGCTTATATTTATACTTCTATAAAAAATGCTTATATTTAGACTACTAAGTCAAGTAGTAGTGCTGCCAGTACAAGCTCAGGCCCTCTGACTAAAACTAGTAACCTTGGTATTCTAACAGTTTTTATCAAGTTTTTGCTGAATTTATATTTACAAAAGCATGAGGTTATGAATCATTATATTTGGATGGATATTTGTAGTTTAATCTGGCTGGAAGAAGTGTGCAAGTATGGATGGAAATAGAACATGTTCACATGCTAAATACACCAGTACAGGCTATAGCTATTCATGATTTCAGGAGAGCCATTGTTAAATACGTTCCTATAAGAGATCCCTTGGCTTTTTGTCATCTAGGCTGATATTCCTGTTAAATCCTTCAATACTTTATTAAAACTTACGCATTAGTGCTTATTATCTGGGATGTTTTCTGCCACTAGTTTTTTTTTTTATTGTTTTGTTTTGTTTTTTTAACTCAATTATTTTTTTATAGGGCTTATCTCAACAGCACAAGCGATTACGATCTTTGGCAGATCTTGTTCCTCATGTTAGAAGGAAATCACTTTTAGAGGTTCTTATTAGGAATAATGTTCCATTGTTGAGGGCTACCTGGTTTATAAAGGTTTCTTACCTGAATCTGGTATGGATGCCTCAATGTTGTGTAATTTACCTGTTTATTTTACCCTGAAATCACCCTCCCCCTTTTCTCAATATTTAAGATAGATTATGGTTGAATTTTGCAGGTTCGGCTGGGTTCTGCTAGTATTCCTTCTGGGACTGCTGACAAAACTCAGCTGTCTTGTTCTGAGCTTTGGACAAAAGATGTTATTGAGTACTTGCAAACTCTTCTTGATGAATTCTTCTCAAAAAATACTTCTCATTTTACTCCTCATAATCGGGACCAATCGCCCCAAGTACCTTATACTGCGTCGCTTCAGCACAGAAGTGACCAGTTATTATCTGTTGCTGATGGTGAAGAACCATCTTTACATTTTAGATGGTGGTATATTGTTCGGCTTTTGCAATGGCATCATGCTGAAGGGCTTCTTCTTCCTTCTCTTATCATTGACTGGGTTTTGCGTCAATTACAGGTGATTGCATATAAGAAGTATTGTGTGTTCATAGAAAATAAACTGAGTATTTTATATTTTTTGCTGATTTCAATCCTTTAGACTTAAGGTTTTGATTTTTCCCACTTCTGCTCCTTTCAGGAAAAACAATTGCTTGAGATTTGGCAGCTGCTATTGCCTATTGTATATGGCTTTTTAGAAATTGTTGTTCTATCTCAAACATATGTTCATACTCTTGCTGGTGTAGCTCTTCGTATTATTCGTGATCCTGCTCCTGGTGGATCAGACCTTGTAGATAATTCCCGGAGGGCATATACTACTTCTGCTCTGATTGAGATGCTCCGATATTTAATATTTGCGGCTTCAGAGACTTTTGTTGCTTTGGATTGCTTTCCTTTGCCATCCTCTGTAGTTTCACATACAATAAATGATGGGAATTTTGTACTGAAAGCAACTGAAGCTGCAGGAAAGATAATAAATAGTTCAGAAGATGTTGTGTGTTTATTTAGAAGTAAAGGATTTGATGCACAATTCCAGTCATTGGCATTTGATCATGTCATTTCGTGCATTCAAGAACGTGTAGAAGATCTCACAAAGGCTGTAAGCCCAGGTTATCCAGGTCAATGTCTGGCTAAAGCTTCCCAAGCCTTGGATAAATCCCTTGTACTTGGTGATATACATGGAGCATACAAATTTCTTTTTGAAGATCTTTGTGATGAAACTGTATCTGAAGGTTGGGTTGCAAAAGTCAGCCATTGTTTAAGGTTATCGCTGAAGTGGTTTGTGACTGTAAATAAATCACTTGTTTATTCAGTGTTTTTCCTGTGTGAGTGGGCAACATGTGATTTCAGGGATTTTCGAAATGCTCCTCCTTGTGACGTAAAGTTCACTGGCAGGAAAGATCTTTCCCAAGTCCATATAGCAATTAGACTTTTAAAGGTGAAGCTAAGGGATATGCAGATTTCACCAAAGCAAAAGAGTGGAAGCACTCGTGGTCATGGAGTCAGTTATTTAGCAAAATGTTCAAGTCTGCAGAGTAATCAGAATTTTGTGAACAATGCATTCAAAATAAAATCTAGTTCAAGAAATTTGGATCAGAATATCTGTTCTTCAGCTATATTTGAAAGCCCAGGTCCTCTACATGATATTATTGTTTGTTGGATTGATCAGCATATGGTGCATAAAGGGGAAGGTTTCAAACGCCTACATCTATATATAGTGGAACTCATACGGGCAGGCATCTTTTACCCACTGGCATATGTACGTCAGCTGATAGTGAGTGGGATCATGGACATGAATGTAAATGTGGTTGACTTGGAGAGACAGAAGAGACATTGCCGAATCTTGAAGCAGCTTCCTGGGAAGTTTGTGCGTGGTGCTTTGGTAGAATCAGGGATTAGTGAAGGGCCATGGCTCACTGAAGCATTGCGGGTTTACTTGAATGAACGCCGCCTCATACTTCGTGGTTCCTTATGGGAGAACCACGACAATGCCAATAATGTGAATATATCTTCTCTCAAGCGAAAACACTGTACAACTTCAACAAAAGACAGAGCTTCTACGGTGTCAATTGATCCATGGAAGAGTATTTTTTCTAATAAAATATCCTCTAAAAATGCAAAGGATGACAATTGTGTTGAAGAACTGAAGACATTCATCTCAACCCTGTTACAGCTACCAAAAAGTTTAACTAATTTGAGCACTACAGGATTGGATGAATCTCAAGGCAGTGTCAGAAAACCTATTGGGTCTCACAACAAGATTGATCTAGTGGAGGCTACACCTGGGTGTGAAGAATGTAGAAAATCAAAGAGGCAAAAATTGAGTGAGGAAAGAAGTTCATTTGTTCAAGCTCCGTCTCTAGTACTGTCTGATGATGAAGACACATGGTGGGTTAAGAAGGGGCTAAAATCCTCCGAGCCTCTCAAAGTTGATCAACCACTTAAGTCAACTAAACAGGTTACTAAGACTCGGCAGAAGACTGTCCGTAAAACCCAGAGTCTTGCTCAACTGGCAGCTTCTAGAATTGAGGGTAGCCAAGGGGCATCAACAAGTCATGTGTGTGGCAATAAGGTTAGCTGCCCTCACCATAGAACTGCTATGGATGGAGATACAACAAGGTCTGTTGATGGAATTCGATCAGGTCACTGTGAAGATATTGTTTCAATTGGAAGGGCACTAAAACAGCTGCGCTTTGTTGAGAGAAAGGAAGTAACACTTTGGCTGATGACTGTATTTAGGGAGCTTATTGAAGAGAGTGAAAAAGATGTTGGTAAAGTTAGCCAGTTTGGCAGGCCTTTTGCCACTGTGGATGATAAAAGTTCAATACGGTGGAAACTTGGTGAGGATGAACTTTCTGCTTTACTTTATTTGATGGATGTCTCAGACGATTTAGTATCAGCTGTCAAATTCCTCGTATGGTTGCTGCCAAAGGTTTATATTAGCCCAAATTCTACAATTCATAGTGGAAGGAATGTGGAGAACCAAGCTTGTGATGTTGGCGAGGCTTTTCTGTTATCATCGCTGAGAAGGTTTGTACTCAACTTAGTTTATGTATTTTTTGGGTCTATTCTGCCACCATTTATTTTGTTCCTTTTATCAGTTTATGTGCTATTCCTAGTTCCTCGTCTGGCTTTCCCTTCTAATCCTTGTGGTGTTCGCTGTGTTTTTAATAACTATGAGAATTGTTTATGTGATATACCACTTGTCTTTCTTCATTTTTTTATTTATTATTCTAACAAGTGCTTGATAAACTTTTGCATGTTGGCGCTTCCTTTGCTCTTCTTTTCCTTATATTTTTAATTAAGTAATTTTTCTGTGACTTCGTATGTGCTTCCAATTTTCCCCCTCATTATTTTTTAATCTAATGAGTATTATTTACTTTTCATCTTTATGTTAACCTAAGGCCCCTTTTTTTTTTTTTTACAATGGATGTTTACATATAGTGATTCTGTTTAGTATGTCTGTCTTGTTGTATTTGATGCAGGTATGAGAATATTCTTACTGCAGCAGATCTTCTTCCTGAAGCTCTGTCATCTATAATGCATCGTGCTGCGGCTATTATAGCAGCATCTAATGGGAGGGTTTCTGGTTCAGGTGCCCTAACTTTTGCTCGTTATTTATTAAAAAAATATGGCAATGTGGTCAGTGTCATTGAGTGGGAGAAAAGTTTTAAGAGTACATGTGATAAGAGACTTGCTTCTGAACTAGAGTCTGGACGGTCAGTTGATGGAGAGTTAGGGCTTCCACTTGGTGTTCCTGCTGGAGTTGAGGACCCTGATGACTTTTTCCGTCAAAAGATAACTGGTGGCCGGTTCCCGTCCAGAGTAGGTTCAGGCATGAGAGATGTTGTACAGCGTAATGTGGAAGAAGCATTTCTTGATCTTTTTGGAAAAGACAGAAAGCTCTTTGCTGCTGGTACACCAAAAGGTCCTGCTTTTGAAAAATGGGATAATGGATATCAAATTGCAAAACAAATAGTTATGGGTTTGATAGATTGCATAAGGCAGACTGGTGGTGCTGCTCAAGAAGGGGACCCCTCTTTGGTTACTTCTGCTGTTTCTGCCATAGTTGGCAGTGTTGGTCCAACATTAGCAAAATTGCCTGATTTTTCAGCTGGCAGTAATCATTCAAATATGTCATTGGCTACAAGTTCATTGAACTATGCTAAATGCATTCTGCGAATGCATATAACTTGTTTGTGTTTGCTTAAGGAAGCCTTGGGAGAACGTCAAAGCCGTGTATTTGAGATTGCACTTGCAATGGAAGCTTCTACTGCTCTTGCTGGAGTTTTTGCTCCAAGTAAAGCATCTCGAGCTCAGTTTCAAATGTCACCTGAAACCCATGATACTGGTACTATTTCAAATGATGCTGCAAACAACAGTAGTAAAATTGTGGTTGCAAGAACAACGAAAATTAGTGCTGCTGTTTCTGCACTTGTTGTTGGTGCAATTATATGTGGTGTTACCAGCCTGGAAAGAATAGTAACCATTCTCAGACTAAAGGAGGGCCTGGATGTTGTACAATTTGTAAGAAGCACAAGATCCAATTCAAATGGGAATGCACGTTCAGTTGGGGCTTTTAAGGTGGATAGTTCAGTTGAAGTTCATGTCCATTGGTTTAGATTGCTTGTTGGGAACTGCAGAACCATCTGTGAAGGCTTAGTGGTGGATCTCTTGGGTGAACCGTCCATTGTTGCTCTTTCAAGGATGCAGCACATACTTCCTCTAACTTTGGTCTTTCCACCTGCCTATTCAATATTTGCTTTTGTTATTTGGCGGCCCTTTGTTATGAATGCCAATGTAGCAGTTCGTGAAGACATGAATCAACTTTACCAGTCTCTAACAATGGCCATAAGTGATGCAATAAAACATTTGCCGTTCCGAGATGTATGCTTAAGAGAGTGTCAGGGCCTTTATGATCTTATGGCTGCAGATACAAGTGATGCAGAGTTTGCAACCTTGCTAGAGTTGAATGGGTCTGATATGCATTCAAAATCTCTGGCATTTGTTCCGCTCCGTGCTAGACATTTTCTAAATGCCATGATTGATTGTAAGATGCCTCACTCTATTTATACAAAGGATGAAGGAAGCCGGAATTCTGGACATGGTGAATCCAAAATTGATTTTACTGATAGTGAATCTACACTTCGGGATAAACTTGTAGATGTGTTAGATGCTCTGCAGCCTGCCAAGTTTCACTGGCAATGGGTTGAACTAAGGCTGCTTTTAAATGAACAAGCCCTCATTGAAAAACTGAAGACACATGATATGTCTTTAGCTGATGCTATACAGTTGTCCTCACCTAGTTCAGAGAAGGGCACTGCTTCTGAGAACGAGAACAATTTTATTGAAATAATTCTCACTAGGTTACTGGTTAGACCTGATGCTGCACCCCTTTTCTCGGAGTTGGTTCATCTTTTTGGGAAGTCACTAGAGGATTCAATGTTGTTACAGGCTAAATGGTTCCTTGCAGGTCAAGATGTCCTCTTTGGTCGGAAGACCATTAAGCAACGACTGATTAACATTGCAGAGACTAAAAGATTTTCTGTTAAGACACAGTTTTCAGAACCCTGGGGTTGGTGCACCCCATGTAAAGATCCAGTTGCTGTCAAGGGGGATAAAATGAAAGTTGATTCTATGCCTCTTGAAGAAGGAGAAGTTGCTGAAGAGGGAATGGATGTGAAAAGGTCCATAAAAGGGTTCTCCCAAGTGGTTGACTCTGAAAGCTCAACCAGTAAGCAGCAGCATGGGACTGAGAGGGCTCTTCTTGAGTTAATTCTTCCTTGCATAGATCAAAGCTCTGATGAATCTCGTAATTCCTTTGCAAGTGATTTGATCAAACAATTAAATTATATTGAGCAACAAATAGCTTTAGTTACTCGAGGACCAAGTAAGCCAATGGCAAGTACTCCTGTTACTGAAGGTCAGACAAATAAAGTAAATAACCGCAAAGCTATAAGAGGTGGCAGCCCTGGGTTAGCCAGACGACCAACACCTGCACCAGATTCTTCTCCACTTTCTCCTGCTGCTTTGCGAGCATCAATATCTTTACGTGTGCAGTTACTCATGAGATTTCTTCCTATTCTTTGCACAGACGGGTAATTAAGACTACATTTCTTCTAATCTTTAAATTATTTGCTATGCTGTCCTGAAATACTTTCTCATTCCTTCCTGGGTTGTTGCTGCACTGAAGTTGTCTTTTTTCACTGGATTAGTATGAGATTTTCTTGAAACATGTTTATATAGTTAACTATTGTGGGGGTAAAAGTCTATGCCTTTTTCAGTTTTCTTGAAATGCATAAAAATGGAGATAATTGCAATATGAACATTTTCAACTGTACAGTTATCTACTGTCTGCTGTTTTATCATGGTTTCCAACTACTTGCATTGAAGATAGTTCATTATGAGTGCTGGAAATCAATTTTGCTTCTGTCCTTTTCAGAAGTGAAATATGGATATTGGTGCTATGAAGGAATATGCTTGTTTTAATGTATGCCACATTAGTGTATCAAATGTAGGCTGTTGTAGAGGTAGTTTAGCTAGATGTTCAATTTTGCAGGGACTCTTCTGTATGGAGCGTGAGATACACACTTGCCTCTGTACTTCTTCGTCTCCTTGGCAGCCGGGTTGTGCATGAGGATGTAACTGTGAAGGCAATGTACTATACTCCATTGAGAAGGGAGGCAGAGTCACATGCTGAAGCTGCTTTTGTAGATTCTTCTGTTGAGGGCCTGTTTGATCATCTGCTGTTGATTTTGCATGGATTGTTGAGTAGTTCTCCTCCAAGTTGGCTCAGGTCAAAATCTGTTTCAAAGACCACTAATGAACCTACAAGGGAGTTTTCTGGATTTGAACGCGAGCCATTGGAGGCATTGCAGGTATGAATCTTTTAGCCTATAGCTTTTAGTTTGTGACCTGATCCCATGTTATTAAATATAGGAAGGCATTTTATTCTTGATATTATCATCTGGCTGATGCTGATTAGAATGAATGAATGCGGGCTTCTTGCTTTTCTTCTGGTCTATTGTATAATACAGAATCATCTTGATAATATGCAACTGCCAGACACTATCCGGTGGCGTATCCAAGCTGCCATGCCTGTGCTCCCTCCCTCTATACGGTGCTCTTTCTCATGCCAGCTGCCAACTGTTCCAGCTTCTGCTCTTGCATCTCTTCAACCCAGCACTACAAATTCTGGGTTTAACTCCAGCTGTTCAACTGTCCCTCAGAGGAACCTGGTTTCATCGTCAAGGACTACAGCATCAGGGAGGTCGAAACTGCAGGACAATGATTCGGATGTTGACCCTTGGACACTTTTAGAAGATGGCGCTGGATCTTGTTCATCTGCAAGTAATACTGCTATCATAGGAAGCGGTGACCGTGTTAATATTCGAGCTGCTAGCTGGCTTAAAGGGGCTGTAAGGGTGCGACGAACTGACCTTTCATATGTTGGTGCCGTGGACGAGGATAGTTGATTTACAAGATTTTTTATTGGTTGGGAGTATGCAAACCCCTTATCAGGTGTATTTGTTGTTTCATTGTCCGTTATTGATGGCATGACTGGCATGAAGATGTCATTTTGTGCTTGCCCTTCCTGTGTTGAAGGGAAGTTGAGGGGTCATCAATTTGAGAAACAGGTGAAGGGATGTTTTCTTTGTTGAAGAGAAGTGAAGGTTCTAGCCTGGTTGACTAACTAACCTGTTGATCACCCCACCCTGGACCTGTGCACTTGCAGACACAGTTCAGTGGTTTGTTGTATATATAGGATCATTGTTTCAACTTTCCCAATTTATTTGATGATATATCTCAAATGGTCTGTTGTTCCGTTCTAATTTATGTCTCAAGGCGTATGCTCAGTCTGTTATCTTTTGGGGTCACAGGCACACACCAGTGCACATGCTCAGTTTTCTTTTTCTATATATTCAGAATATCAAAATTATCTGTGTTTTTCATCTGGAGAAATGCAGCATTCAATTATTAACTTG

>GmMED12-4

TTCTATTGCGATTCACAGAAAACACCACCAACTCAACACAGTCATCATCGTCACTACAAAAAAAAAATAATAAAAAAAACACAGAGTCAAACCCTCGACCACACTACAGAGACCCTTCTTCTTCTACTTTGTTCCTCTGTTTCTCTCTCTTACTTTCTCACTCTTTCCGTCAAAGTTTGCAATTTTTCTTCCCAGGTATGCCCCCTATCATTAATCTTCAATATATTGCAATTCCCCCATTATTAATTTTGTTGCTAATATTGATTTTTCTGAAACCCCTTTTGTTCCGTTTCGCCGAATTGTGAATCAAAAACCCTAGTTTTGCTTGTACTGGAATATCCCCAATTTTCAGCTCCGCAATTAGCTTGCGCCGGCATTTCCGAAGTTGGGGAAGAACGAAATAGTAACCGGAATTTCGTTCTCAAAAACCCTAGCTTTGGATCGTGCTTTGGAGTTCTAATTGTGGAGTTTTTCTTTTCTCTCGCATTGGATTTGATTTTGTAGGTTTTGGTAATTGCTATTCAGGTTTGCGTAGAGTATAGCTTGGATTTGACGTTAAAGCTGAGATTTTTGGACGATTCAAGTGGTGGGTTTTTAATTCAGTTTTTGCCTCTTGCGTAAAACTTTCATTTGGTGCCCTTGTTAGTTAGAGCAGGGAAAGTGAGATTGGAAAGTGAGGGTATTTTTATTTCTCTGAGTTTTGCAGTTATGCTTGTGGATTGGGAATGATTTGGTTAAATTTTGCAACTTTGCATTGTATCTCGTGTTATAATTTATTACTGTTGGTTGCGGATTCAAGACTTGAAGTTATGATTCTGGCAACGGTCGGCCATGAGTTTATAAGCGATAGGACTTGCTATAGGCATGTTTTGGTACATGTAGAAACATTTTAATGGAGAGTTTCCAATTTCAATGGGAATTGAATTCACTCGTGTAATTGTCGTGATCGTGTTTGTTTTAGGTTGACAGGAGATTCCTGAGGGTAGAATTAAAGCTTCTAAGTTCATTGGTATACTTACAGTTACACTATTTTTGAAAACAAGACTTTTATATGGTTGGGCATGGGGTTTTCGTGTCAATGAAGGTTTCAGAACATAAATGTGGAAAGTTCTTCAAATTTTCTTTACCCCAGCTTTCACACACCTATGTATTGGAAACTGGACTTAGCGTGAACAAGAGTGTAGGATTATGGTTTCTTTTATTTTTCGGAATATTTTATGTTCTTTGCATCTTAAATGAAACATTCATAGCTTGTTTGGATGTATGCCATGTTCGTTTCAAAATCGAGTCCTTTCCACGTTTGATGCAAAAGCTTAAAATGATAGCTTCTCTTTTTTTCGACTGATACACGTGTGGGTTTTTGGACACGAAGCCAAACATCCACTTAGACTATTGGATTTTTTTAAATTTATTTTTTGGAGCTTTCATACTGCCTTTGAGTTGCCCTCTGTAATCTGGATGTTAGTTAATGATTCTTCTTATGGTGCCTGATATGGATTTGTTTCTTAATTCTTTACCCATTTGTGTTTTACAGGCCAGAATAGTACCACTCTTCCTCATACAGCTGGGGTTCACAAATTGTGGGTTTCATGTTTTTGGGTATTTGGATTTCATTACATATTTTATAGTGGTTGAAGACGGTTCAATAACACATAGATTTGGTGCTTAAAGGTCATTTTCTCTTATTCTTTCTTCAATTTGTCTTTTTTTTTTCCTTTGTCACTTTTTTAGGATTTTCTTTTTAGTTTGCTTAAATGCTCTGGTTGGAAATAACACAGCACATTTTCAATGATGCAGTTTAGCTATTACAGCTACTTGTAGTGGTTTGATGCTTAGGGCAGTATGCTGGTTATAATTGGAGGAAATGCAAAGGTATCATGCTGGCAGCTGCACTAGTGCAGTTAATAACAGTGCAATTGGTGGGCCATCCACTAGGGACATTGGAAGAACTGATTCATCTTCTTTGCCAGCTAACTTTCCTGTGAGTTCAAGGTAAGATTCTATGTCTGCCAACATTTCTTTACTGAAAATATTATTACCTATTCTTTTGTTTCAGATTTTGTTACTGTAGTACATGTGATTAGGGTACTACATATTCCAAATAAATGTCGTTGCGATACAGCTTCTGGTGCTTCAGTCCCTGTTAGCTAATAGCTTTTTTTATTGCAACAGTGATCGATTTTATTTACTTTATGTCATTTTGAAACCTGAAAAACTCCACTTCAGAGCTGGTTCATTTATCTTTTGACTCTTGAGTAATCACAATATTCTAGTTTTGGGTTGTCACTTCTGTTCTTATGCATTTAAATGATTGAGATATTACAAGGAGTTTTATCTGTTATAACCAACTAAACATTTCCCCCCTTGTATGCTCTTTTCTATTAATATATAAACTTCTGATACTTTGCTTGGATGATCAGATGAGCAAGAAATATTAGGTTTCATATCCACAGTAGGCATGACTGCATGAGCCACTAGCGAGGGACATAGTATTTGTTATTAGAGAATTGGTAAATACAGGGTTGAAAAATATCTATTTCTTTTTTTTTTTTGATCGGAAAAGATATATATATATATATATATATATTAAAAGAAGTACCAGAGGTACTATTACAAAGTTGGGCTCCATATAAATGGTTCCTGAAAGCAGACAGCAAAAGTCTGATTTGGAACCAAATTATGGAAAAAATACATGTGTTTTAATATAAACTCTAAACCCTACTACTGCAAAAAACCCTGTCGTAAGTTACTCGACCACTGATTAAAATGCAATGAAAAGTCCTTCTCCAAATTATGAAGCCAAGTCCATATTAGAAAAGTCGCGTCTTCAAACAGTTTGTTTCCATCAAAAGTTGCATTGGAGAATAAAATTCTGTTTCTAAGCTGCCAAGTGGACCATGTAACCGCCAACCACCAAATCTGCCACCTCCTAGGTCTGATGCCATCTACCTGCAGAGAAGAATACTGCATGAAATGATCTTTTATGCTCCATGGATGGGCACCCTTCAAATTTATCCAAGACATTGTTTCCCACCATATAGGTTGGATCTTAATGCAATGGATAAAAATATGGGATGCATTCTCCTCCACCCTTCTGCAAAAGGGGCATGTCATATCCAAAATCTGCACTTGTCTAGCCCTTAAATTAGATTTTGTAGGTAGTCTGTCCCACAACATCCTCCATGCGAAAACCGCAATTTTACTAGGAATCTTGATCTTCCATAGGTCCTCAAAACATTCATGAAGGTTCTCAACAGCTGCCTCCTCCCAAAACGTTGAGTAAGCACTACGAGTAGAGTAGATACCCGACGTGTCTGCTGCCCAAACCCAACTATCAGAACCTTGAGTATGTATAGATTTACCTTCAACTTCAGAAAGAAAAGAGATGGCTATTTCAATTTCACTGTCAAACAGCTGTCTTCTCCAACTAAAGTTCCATTCCCACCCATAGTTACTATACTTCCCCAAGAGACCAATCAGCTGGTGTTGTTGAGCAGAATTCATGTAAAGCCGAGGGTACTTTTCAGCTAATGATTGGTCATGACTAATCCACCTATCCTCCCAAAATCTTACCTTATCCCCAGCGTGTACCTTCCAATTAATTTGCTGATGTAGAGTCTTGCCATGGTGTGACTGATGGATAACTTTTTTTAAATCCCTCCACCAAATTGACTGTAAACTTGAGCTTCCCTCTTCAGTTAGACCTCTCCAGCCCCCATATTTAGCCTCCAAAACTGCAACCCAAAGAGCCCCCATATTTAGCCCCCATATCTATTTCTTTGATGATGTTGGTTCTGTAGAATGGTTTATGTTTCAAATCTATTTTGATATTCTATCCTAATATACTTCAAAACAGTGGCATACTTATTCACTAATCACTAGAGATTGTTATACTATAATTTGTAAGAACTGTTCAAAACTTTAAATATTTATCCTCCTGGTTGTGTTGACTAAATAAGTGAAGTAAATGGTATTGTGCTTATGCTGGATGTTTTTCAACCCTGAAATTTAGTTTATTTTCTTTATACCGAAAATTTAGCAAAGATTTCTTGAACTGATTATCTAATATTATAGGGACCTCTCTGAATTGCCCCTATCTTTTAAAAAGTAGTGAGGTTGGGAACATAAAGTTACTGTCACCACTTAAGCTAGATAGCTGCTCACTGATTTATAAGAAAAAACTCAGAAATAGCCACTAGCTAGCTAAGTTACGTAACCAGAATATCTTTTTGGTTTTTTTCTGTTTTGAGGTTGAAAGTACACAAACTAGTGTCTGAAATTCCAAATTAGGAGTAATGAATTATCATATAAAATTAGAGAATGACGCTATGATAGCAATAAATCTGCAGAAGTTGGTTGGTCTTTCTGTGTAACCTTTTACTCATGTTTTGTGTTGAGGTTTCTCTGTTTTCTTTTCATTTAAATAATCAGAACCTTATATCATTTATTATTTTGTTACTGATGCTGCTATGTGGAGGAGCTGTTTTTTAGATGATAAAAGATATGGAGTTTTTTTTTTTATTTTTTTTTAAATTTTATAACTCATTTCATTTGAAGCCCAGATAACTTAACATGACACTGGTATGATGTGACACAGGTATGAGGATATGAAAAATTCTTTAAAAATTCAAGATATGGTAATGATACAGCCTAGATACATTAACGTGAATTCTTTTCTACAAATTCTTTATGTACATTAGTGAAGGATGGTCATTATTTGAAAGAAAAATACTTTTAATCATAATAAAAAACCATAGTTACATATCTCTCTCTTATCCATATCAGAATTTATTCTCTCCCCAATTAACATGCCAAAGAATTACATATTTTCCCCCAACTCCAGTTAGAATGTAAAAAAATAAGAAATCACTTGTCCTATTCAAAAGAAATATTGGGTCTACTTTATTGTCTACTATTTACAACAGCTTTGAAGATACCGAGAGAGATAGAGCTGTTTGTAATTTTAGTCCTTTGTTCTGTTTGATACTCCATAATAAAATGGTATATTATTATTTGTTCTCATAAATCATTTATTTGATGCTGTTAATCACTTTAAAAACAATGGTTTTACTTTTCAGGCGACAACCACCATTAACCCCATACAAGTTGAAGTGTGATAAAGAACCTCTGAACTCTAGGTAGTTCATGCTTGGAGCAGAACCTGTTTTCAATTTGTTGCTCAATTTTGTTAGTTCATGAGTAACAAACATCATAAGTTTTTCTGGAGTAAAATTTGCATGGGCACGGTTAATATCGAAAATATTGTTTGATGCCCTTGATCATCAGTATCTGTTTTTGTTAGTTGTTATTCAATGATATTAAAATTGCAATGTTTTAATCCTGGTAGTACTCACTTCTAGTTTTTGCTTTAAATTTCCTTATTGTCTAGTTGTATAAATATTAAATGTCACTGTTTAATCTCAGGCTTGGGCCTCCAGACTATCACCCCCAAACACCAAATTGTCCTGAAGAGATTCTGACCAGAGAATATTTGCAATCTGGATATAGGGACACAGTTGAAGGGCTTGAGGTACATTTTTTTTAAACTTCTGTATATATATCAAATGGATAATATAAAGTAACTATTTAGCTATGAATGTTTGAGTTATAGCCATAGTTGTTAGAATCTTACAATTCTTGATTTGAATCCTATGATTCGATACGATTCAGCTATACCTATTGATTCATATCATAAGTTGAATCTCTAATGACTTTGTATCTTATGATAATGAATGAATATGTGCAATTTCATATCATTGATATGAATTGCATGATTCAACTGTTTATTTTTTCTTTTTCTTTCACCAATTCTCATGAAAATAATTGTAAAGTCATAAGTTTTAATTGTTAAATGAAATGGAACTGAAGGACCATTTATGAACTTATCCAATTTGATTATTCCACTACTTAATTTGTCACCCACTCATTTAATGCATGCACTCTATTTGGCTATTTAATTGTTTTGTTTCCATTCTTCTAAAACAAGGCTTCACAAACTCAGATATAACTAGTAACTTTGCTTAACAAAACTTATGATTAAGTTGTTTTTATCTATTTCTAGTTTAAAATTATTACATTGAACACCGTATATCTTATGTTGGAATGAAGATCCTGTTGTGTTTGGATTTGTTTTATCATTATGTTTCTATCTGTGCTATTATCAGTCAATTTGAAAACAGTTGGTTTTTTCTTTCAAATTTCTTATTTGGATCTCCTACTGTTCTATATGATTGAAACTATGCTGTAGGGAATTTTATATTCTAGAGGATTGGCTTCCTTTGATGAATTGTTGAAAATCAACTTGTGCTTTCTAAAACTGCTTGTTGTTTTTATAACATGGTATTACCTTTTTTGTCTTAAGGAATCTAGAGAAATTTCTCTGACCCAGGTTCAAAATTTTAGCAAGAAAGTTGTCCTTAATTGCAAAGAGGTGAGTCCCTAGTCTCAGAGAGAATTATGCCCTGTTGTTTCTATAACTATGAGTGTGTGGCATGTCAAATAAGCTAGCTTATTAGCTTGTCTGCCCAAATTAAGGTGTTTGATAAACAAAATTATTTCACTACCTTATAGCATATAAGCTATAAACTAATAACTTATGAGCTTGAAGCTTTTTTTTCCTTCCATTTTTTTCCTTACCATTTTAATGTACTTAATTTTAATTTTAATGAAATATTTGATCTTTTAAAAAATTAATACGAATGTAACATTAATAAAAGAATCTAAAACTATAATTGCCAAAAAATATATTTTTTTGTCTACAAAACAAAAATTCTATTTATATTATATTTTTTATGAATGCTTTTTATGTCATTTTACATTTTTAGCTACTTCAATAACTAATTTTTACCAAACACTCGTAATACTATAAGCTAGTTTATAAGCTTTCAGCCTTTAAGCTTGCTTATAAGTCTTAAGCTGGCTTATATGCTTTAAGCTACTGATTATTTAGTTATGTCAAACATAGTCTATGCCATGAATATACTGGACATTTTCCATATTTGGTTTTTATATTCATTGGAAGTGTTTGCAGGTTAATTCTTTAATCTTTCATCTTCTCTCTCCAGGCTATTAGAAAACGGCTACGGGCCATCAATGAATCTCGCGTGCAGAAGCGGAAGGTGATGACTGATGATCTTTATTCTGTTATTTTATTATGCTTGATGAACCATATGTATGCCTCTAAAATATTCCATTTTTGTGTTTTTGGTTTTGTTTTATTTTGGGTTTAGTGGAGCCCCTACTTATTATGAAGGTATTTGATCTACATGGTAGTGCTTATTTTGACAGGCTGGTCAAGTATATGGAGTGGCTCTTTCAGGGTCACAACTTGCTAAGCCTGGTGTTTTCCCTGAACAAAGGCCCTGTCCTGAAGACTTCCGAAAGAAATGGATTGAGGTACTAATGGAAGTCTTGCTCCTGTTGCTTTACTCTTTGTACAACTTTCCTGTATAAATATTGTTTCGCCTGTTATTTGTTTCTGTTAGAATGTTAGTGTTATTGTTTTGTTGTTAGTGAACTTGGTTGGGCTTGTAGCCTTGTACTAGACTTAGTTATTGGTATAAATTATTAATAGCGTTAGGGATTGCACAACACATCATTATTCAGCTTACTTTAGTCATTTTTCTCTCTTTTCACCCAAACTACACAATTTTAGCACATTGAAATTGCAGGGTTTAGGTAAAAAGGATGGAAAAAAAGGGAAAGACTGCTAATACAATGGAGATTGATATTAGATGTTACACAATACCTAGCCTAATGCTTATATTTATACTTCTATAAAAAAATGTTTATATTTATAATACTACGTCAAGTAGTAGTGCTGCTAGTATAAGCTCAGGCCCTCTGACTAAAGCTAATAACCTTGGTATTGTAACAGTTTTTATCATGCTTTTGCTGAATTTATATTTAGAAAAGCATGAGGTTATGAATCATTTGTATATTTGGGTGGATATTTGTAGTTTAATCTGGTTGGACGAAGTGTGCAAATATGGATGGATACAGGACATGTTCATATGCTAAATACACCAGTACAGGCTATAGCTATTCTTAATTTCAGGAGAGCCATTGTTAAATACGTTCCTATAAGAGATCACTTGGGCTTTTTGTCATCTATGCTGATATTCCTGTTAAATCCTTTGATACTTTATTAAAACTTACTTATTAGTGCTTATTATCTGAGATGTTTTCTGCCACTAGTTTTTTTTTTTTTTTTATTGTTTTGTTTTAGTTTTTTTAACTCAATTTTTTTATAGGGCTTATCTCAACCGCACAAGCGATTACGATCTTTGACGGATCTTGTTCCTCATGTTAGAAGGAAATCACTTTCAGAGGTTCTTATTAGGAATAATGTGCCATTGTTGAGGGCTACCTGGTTTATAAAGGTTTCTTACCTTAATGTGGTATGGATGCCTCAATGTTGTGTAATTTACCTGTTTATTTTACCCTGAAGTGACCCTCCCCCTTTTCTCAATATTTAAGATAGATTATGGTTGATTGCAGGTTCGGCCAGGCTCTGCTAGTATTCCTTCTGGGACTGCTGACAAAACTCAGCTGTCTTGTTCTGAGCTTTGGACAAAAGATGTTATTGAATACTTGCAAACTCTTCTTGATGAATTCTTCTCAAAAAATTCTTCTCATTTTACTCCTCATAATCGAGACCGATCACCCCAAGTACCTTATACTGCGTCATTTCAGCACAGAAGTGACCAGTTATTATCTGTTGCTGATGGTGAAGAACCATCTTTACATTTTAGATGGTGGTATATTGTTCGGCTTTTGCAATGGCATCACGCTGAAGGGCTTCTTCTTCCTTCTCTTATCATTGACTGGGTTTTGCGTCAATTGCAGGTGATTGCATATAAGAAGTCTTGTGTGTTCATGGAAATTAAACTTAGTATTTTATATCTTTTGCTGATTTCAATCCTTTAGACTTCAGGTTTTGATTTTTCCCACTTCTGCTCCTTTCAGGAAAAACAATTGCTTGAGATTTGGCAGCTGCTATTGCCTATTGTATATGGATTTTTAGAAATTGTTGTTCTATCTCAAACATATGTTCGCACTCTTGCCGGTGTAGCTCTTCGTATTATTCGTGATCCTGCACCTGGTGGATCAGATCTAGTAGACAATTCCCGGAGGGCATATACGACTTCTGCTCTGATTGAGATGCTCCGATATTTAATATTTGCGGCTCCAGAGACTTTTGTTGCTTTGGATTGCTTTCCTTTGCCATCCTCTGTAGTTTCACATACAATAAATGATGGGAATTTTGTACTGAAAGCAACTGAAGCAGCTGGAAAGATAAAAAGTAGTTCAGAAGATGTTGTGTGTTTATTTAGAAGTAAAGGATTTGATGCACAATTCCAGTCATTGGCATTTGATCATGTTATTTCGTGCATTCAAGAATGTGTAGAAGATCTCACAAAGGCTGTAAGCCCAGGCTATCCAGGTCAATGTCTGGCTAAAGCTGCACAAGCCTTGGATAAATCCCTTGTACTTGGTGATATACATGGAGCATACAAATTTCTTTTTGAAGATCATTGTGATGAAACTGTATCTGAAGGTTGGGTTGCAAAAGTCAGCCATTGTTTAAGGTTATCGCTGAAGTGGTTTGTGACTGTAAATAAATCACTTGTTTATTCAGTGTTTTTCCTGTGTGAGTGGGCAACATGTGATTTCAGGGATTTTCGGAATGCTCCTCCTTGTGATGTAAAGTTCACTGGCAGGAAAGATCTTTCCCATGTCCATATAGCAATTAGACTTTTAAAGATGAAACTGAGGGATATGCAGATTTCACCAAAGCATAAGAGTGGAAGCACTCGTGGTTGTGGAGTCAGTTATTTAGCAAAATGTTCAAGTCAGCAGAGAAATCAGAATTTTGTGAACAATGCATTCAAAATAAAATCTAGTTCAAGAAATTTGGATCAGAATATCTGTTCTTCAGCTGTATTTGAAAGCCCAGGTCCTCTACATGATATTATTGTTTGTTGGATTGATCAGCATATGGTGCATAAAGGGGAAGGTCTCAAACGCCTACATCTATTTATAGTGGAACTCATACGGGCAGGCATCTTTTACCCACTGGCATATGTACGTCAGCTGATAGTGAGTGGGATCATGGATATGAATGTAAATGTAGTTGACTTGGAGAGACAGAAGAGACATTGCCGAATCTTGAAGCAGCTTCCTGGGAAGTTTGTGCGTCGGGCTTTGGTAGAATCAGGGATTAGTGAAGGGCCACGGCTCACTGAAGCATTGCAAGTTTACTTGAATGAGCGCCGCTTCATACTTCGTGGTTCCTTATGGGAGAACCATGGCAATGCCAATAATGTGAATATATCTTCTCTCAAGCAAAACCACTGTACATCTTCAACAAAAGACAAAACTTCTACAGTGTCAATTGATCCATGGAAAAGTGTTTTTTCTAATAAAACATCCTCTAAAAATGCAAAGGATGACAATGGTGTTGAAGAACTGAAGACATTCATCTCAACCCTGCTACAGCTCCCAAAAAGTTTATCTAATTTGAGCACTACAGGATTGGACGAATCTCAAGGCAGTGTCAGAAAACCTATTGGGTCTCACAACAAGAGTGATCTAGTGGAGGCTACCCCTGGGTGTGAAGAATGTAGAAAAGCAAAGAGGCAAAAATTGAGTGAGGAAAGAAGCTCATTTGTTCAAGCTCCGTCTCCAATACTATCTGATGATGAAGATACTTGGTGGGTGAAGAAGGGGCTAAAATCTTCAGAGCATCTCAAAGTTGATCAACCACTTAAGCCAACTAAACAGGTTACTAAGACTCGGCAGAAGACTGTCCGTAAAACCCAGAGTCTTGCTCAACTGGCAGCTTCTAGAATTGAGGGTAGCCAAGGGGCATCAACAAGTCATGTGTGTGGCAATAAGGTTAGCTGTCCTCACCATAGAACTGCTATGGATGGAGATACAACAAGGTCTGTTGATGGAATTCGATCAGGTCACTGTGAAGATATTGTTTCAATTGGAAGGGCACTTAAACAGCTACGCTTTGTTGAGAGAAAGGAAGTAACACTTTGGCTGATGACTGTAGTTAGGCAGCTTATTGAAGAGAGTGAAAAGAATGTTGGTAAAGTTAGCCAGTTTGGTAGGCCTTTTGCCACTGTGGATGATAAAAGTTCAATACGGTGGAAACTTGGTGAGGATGAACTTTCTGCTTTACTTTATTTGATGGATGTCTCAGACGATTTAGTATCAGCTGTCAAATTCCTCTTATGGTTGCTGCCAAAGGTTTATAGTAGCCCTAATTCTACAATTCATAGTGGAAGGAATGCTTTAATGCTGCCAAGGAATGTGGAGAACCAAGCTTGTGATGTTGGCGAGGCTTTTCTGTTATCATCACTGAGAAGGTTTGTACTCAACTTAGTTTTTGTATTTTTTTGGGTCTATTCTGCCACCATTTGAGGGCGAGCCCTGGTGCAACGGTAAAGTTGTGCCTTGGTGACTTGTTGGTCATGGGTTCGAATCCGGAAACAGCCTCTTTGCATATGCAAGGGTAAGGCTGCGTACAATATTCCCTCCCCCATACCTTCGCATAGCGAAGAGCCTCTGGGCAATGGGGTACGAAGTTTTTTTTTATTCTGCCACCATTTGTTATTTTGTTCCTTTTATCAGTTTATGTGCTATTCCCAGTTCCTCGTTTGTCTTTCCCTTCTAATCCTTGTGGTGCTTGCTGTGTTTTTAATAACTACGTGAATTGTTAATGTGATATACCACTTGTTTTACTTCATTTTTTTAATTATTCTAACAAGTGATTGTTGAATGTTTGCATGCTGGCGCTTCTTATGCTCTTGTTTTTCTTATATTTTTAATTAAGTAATTTTCCTGTGACTTCGTATGAGCTCCCAATTTTCCCCCTCATAATTTTTTAATCTAATGAGTATTATTTATTTTTCATCTTTATGTTAATCTGAGACCTTTTTTTTTATGTTTTTTATAATGTATGTTTACATATGATATTGATTTTGTTTAGTATGTCTGTCTTGTTGTATTTGATGCAGATATGAGAACATTCTTGCTGCAGCAGATCTTCTTCCTGAAGCTCTGTCATCTATAATGCATCGTGCTGCGGCTATTATAGCATCTAATGGGAGGGTTTCAGGTTCAGGTGCCCTAACTTTTGCTTGTTATTTATTAAAAAAATATGGCAATGTGGTCAGTGTCATTGAGTGGGAGAAAAGTTTTAAGAGTACATGTGATAAGAGACTTGCTTCTGAAATAGAGTCTGGACGGTCAGTTGATGGAGAGTTAGGGCTTCCACTTGGTGTGCCTGCTGGAGTTGAGGACCCTGATGACTTTTTCCGTCAAAAGATAAGTGGTGGCCGGTTACCGTCCAGAGTGGGTTCAGGCATGAGAGATGTTGTACAGCGTAATGTGGAAGAAGCATTTCGTGATCTTTTTGGAAAAGACAGAAAGCTCTTTGCTGCTGGTACACCAAAAGGTCCTGCTTTTGAAAAATGGGATAATGGTTATCAAATTGCTCAACAAATAGTTATGAGTTTGATAGATTGCATAAGGCAGACTGGTGGTGCTGCTCAAGAAGGGGACCCCTCTTTGGTTACTTCTGCTGTTTCTGCCATAGTTGGCAGTGTTGGTCCAACATTAGCAAAATTGCCTGATTTTTCAGCTGGCAGTAATCATTCAAATATGTCATTGGCTACAAGTTCATTGAACTATGCTAAATGCATTCTGCGAATGCATATAACTTGTTTGTGCTTGCTTAAGGAAGCCTTGGGAGAACGCCAAAGCCGTGTATTTGAGATAGCTCTTGCAATGGAAGCTTCTACCGCTCTTGCTGGAGTTTTTGCTCCAAGTAAAGCATCTCGAGCTCAGTTTCAAATGTCACCTGAAACCCATGATACTGGTACTATTTCGAACGACGTTGCTGCAAATAACACTAGTAAAATTGTGGTTGCTAGAACAACGAAAATTAGTGCTGCTGTTTCTGCACTTGTTGTTGGTGCAATTATATGTGGTGTTACCAGCCTGGAAAGAATAGTAACCATTCTCAGATTAAAGGAGGGCCTGGATGTTGTACACTTTGTAAGAAGCACAAGATCCAATTCAAATGGGAATGTACGTTCAGTTGGGGCTTTTAAGTTGGATAGTTCAGTTGAAGTTCATGTCCATTGGTTTAGATTGCTTGTTGGGAACTGCAGAACCATCTGTGAAGGCTTAGTGGTGGATCTCTTGGGTGAACCATCCATTGTTGCTCTTTCAAGGATGCAGCGCATGCTTCCTCTAACTTTGGTTTTTCCACCGGCCTATTCAATATTTGCTTTTGTTATGTGGCGGCCCTTTGTTATGAATGCCAATGTAGCAGTTCGTGAAGACATGAATCAACTTTATCAGTCTCTAACAATAGCCATAAGTGATGCAATAAAACATTGGCCGTTCCGAGATGTATGCTTAAGAGAGTGTCAGGGTCTTTATGATCTTATGGCTGCAGATACAAGTGATGCAGAGTTTGCAACCTTGCTAGAGTTGAATGGCTCTGATATGCATTCAAAATCTCTGGCATTTGTTCCGCTCCGTGCCAGACATATTCTAAATGCCATGATTGATTGTAAGATGCCTCAATCTATTTATACAAAGGATGAAGGAAGTCGGAATTATGGACATGGTGAATCCAAAATTGATTTTACCGATAGTGAATCTACACTTCAGGATAAACTTGTAGATGTGTTAGATGCTCTGCAGCCTGCCAAATTTCACTGGCAATGGGTTGAACTCAGGCTGCTTTTAAATGAACAAGCCCTCATTGAAAAACTGAAGACACATGATATGTCTTTAGCTGATGCTATACAGTTGTCCTCACCTAGTTCAGAGAAGGGTGCTGCTTCTGAGAATGAGAACAATTTTATTGAAATAATTCTCACTAGGTTACTGGTTAGACCTGATGCTGCACCCCTTTTCTCGGAGTTGGTTCATCTTTTTGGGAAGTCTTTAGAGGATTCAATGTTGTTACAGGCTAAATGGTTCCTTGCAGGTCAAGATGTTCTCTTTGGTCGGAAGACCATTAAGCAACGACTGATTAACATTGCAGAGACTAAAAGATTTTCTGTTAAGACCCAGTTTTCTGAACCCTGGGGTTGGTGCTCCCCATGTAAAAATCCAGTTGCTCTCAAGGGGGATAAAATGAAAGTTGATCCTATGCCTCTTGAAGAAGGAGAAGTTGCTGAAGAGGGAATGGATGCGAAAAGGTCCATAAAAGGGTTCTCCCAAGTGTTTGACTCTGAAAGCTCAACCAGTAAGCAGCAGCATGGGACCGAGAGGGCTCTTCTTGAGTTAATTCTTCCTTGCATAGATCAAAGCTCTGATGAATCTCGTAATTCCTTTGCAAGCGATTTGATCAAACAATTAAATTATATTGAGCAACAAATAACTTTAGTTACTCGAGGACCAAGTAAGCCAACAGCAAGTACTCCTGTGACTGAAGGTCAGACAAATAAAGTAAATAGCCGCAAAACTATAAGAGGTGGCAGCCCTGGGTTAGCCAGACGACCAACACCTGCACCAGATTCTTCTCCACTTTCTCCTGCTGCTTTGCGAGCATCAATATCTTTACGTGTGCAGTTACTCATGAGATTTCTTCCTATTCTTTGCTCAGACGGGTAATTAAGAACTATATTTCTTCTAACCTTCTAATTATTCACTATGCCATTCTGAGATACTTTCTCCTTCCTTCCTGGGTTGTTGCTGCACTGAAGTTGTCTTTTTTCATTGGATAAGTTGTATGAGATTTTCTTGAAACATGTTTATATAGTTAACAATTGTGGGGGTAAAAGACTACACCATTTTCAGTTTTCTTGAAATGCATAAAAATGGATATAGTTGCAATATGAACATTTTCAACAATACAGTTATCTACCATCTGCTGTTTTATCATGGTTTCCAACTACTTGCATTGAACATAGTTCATTGAGTGCTGGAAATCAATTTTGCTTGTCCTTTTCAGAAGTGAAATATGGATATTGGTGCTATGAAGGAATATGCTTGTTTTAATGTATGCCACATTAGTGTATCAAATGTAGGCTGTTGTAGAGGTAGTTTAGCTAGATGTTCAATTTTGCAGGGAGTCTTCTGCAAGGAGCATGAGATACATGCTTGCCTCTGTACTTCTTCGTCTCCTTGGCAGCCGGGTTGTGCATGAGGATGCAACGGTGAATCCCATGCACTATACTCCATTGAGAAGGGAGGCAGAGTCACATGCTGAAGCTTCTTTTGTAGATTCTTCTGCTGAGGGCCTGTTTGATCATCTGCTGTTGATTTTGCATGGATTGTTGAGTAGTTCTCCTCCAAGTTGGCTCAGGTCAAAACCTGTTTCAAAGACAACTAATGAACCTACAAGGGAATTTTCTGGATTTGAACGTGAGCCATTGGAGGCATTGCAGGTATGATCTTTTAGCCTATGAATGTACAGCTTTTAGTTTGTGACTGATCCCATGTTATTAAATATAGGAAGGCATTTTATTCTTGATATTATCATCTTGGTGATGCTGATTAGAATGAAAGAATGCAGGCTTCTTACTTTTCTTTTGGTCTATTGTATAATACAGAATCATCTTGATAATATGCAACTGCCAGACACTATCCGGAGGCGTATCCAAGCAGCAATGCCTCTGCTCCCTCCCTCTATACGGTGCTCTTTCTCATGCCAGCTGCCAACTGTTCCAGCTTCTGCTCTTGTATCTCTTCAACCCAACACTACAAATTCTGGGTTTAACTCCGGCAGTTCAACTGTCCCTCAGAGGAACCTGGTTCCATCATCAAGGACTACAACCTCAGGGAAGTCAAAACAGCATGACAATGATTTGGATGTTGACCCTTGGACGCTTTTAGAAGATGGTGCTGGATCTTGTCCGTCTGCAAGTAATACTGATATCATAGGAAGTGGTGACCGTGTTAATATTCGAGCTGCCAGCTGGCTTAAAGGGGCTGTAAGGGTGCGACGAACTGACCTTACATATGTTGGTGCCGTGGATGAGGATAATTGATTAACAAGATTTTTTATTGGTTTGGAGTATGCTAACCCCTCATCAGGTGTATTTGTTGTTTCATTGTCCATTATTGATGGCATGACTGGCATGAAGATGTCATTTGGTATTTGCCCTTCCTGTGTTGAAGGGAAGTTGAGGGGTCGTCAATTTGAGAAACAGGTGAAAGGGATGTTTTCTTTGTTGAAGAAAAGTGAAGTTGATGTCCTGGCAAGCTCATCCTGTTGGTTCTAGCCTGGTTGACTAACTAACCTGTTGATCACCCTACCCTGGACTTGTGCACTTCCAGACACAGTTCAGTGGTTTGTTGTAAATATAGGATCATTGTTTCAACTTTCCCAATTTATTTGATAATATATCTCAAATGATCTGTAGTTGTTCCGTCTAAGCGATAATATATCCCAATTTATTTGATGTCCTGGTAAAGTTTGTTTTTAGTCTTTAAAAAACAATTTGTTTCATTTGTGAAAACGTGTTAGATGTGATATTTATTCATATAATTAAATTGTCTCCCTATGGCATCTCATTTATG

>GmMED13-1

TTTTTCACTCCTGATCGAAACAAATACTCGGTGGGATCTGTGGTGCAATCTGAATCAGATTCGGAATTTTTTAATGGAGTGGGGTGCATTTGTGGTTGGTTTGTGCAGAAGCAGAAGCAAGTAAACGTATCATAAACCCTCGTCCCATTCTTAATCTTAGGTCGGAGTCTCAGTCTGACACAACACAAACAGATCGACCGCCCGGCATCATCGCATTTGCAACCAAACCCTAGTTCAATTTACTGAAACCTTCATACCATATTCCAGATATGTGGACAAATGTTTTCAAAATCGTAGGTTTTTCTACTTCTCACTTTTTTTTGGGTGATTACTTTTATGCTGCGACTTAATATGATTAATTTCGCTTTACCCCTTTTCTTATCTCGTAAGCTGACTTTGTAGTTGGTGCTGTACTCATCTCATGAATAAGTTAGTGCAAACATGTTTAACGACTAAAAGTATTGTAAATTATCGTATAATTAGTCGATACAAGGTGTTTATTTTAGAAGTAAGTTTGGTTTGTGACGGCATATAATTTTATTACACACACACACACAGGGAGGCTTTAACAAGCTTTGAGCACGTTCTTTCTTCCCTATTTGATCGAATTCTGGAATTGTGGTTTTTCCGCTAAGCATCACAAGGGTTGACTGGAGAATTTTTTAACTATACCTGCACTGTTGCTCTGTGCTGAAACACAAATGGTTACATGTTTCAGTCCCCCTTCCTTAGTGGATGACTAGTTTCATTATCTGCACATGCTTATAATTATGTTTGGTGTTGCAGTATGGTACTCTAGCCAAGGGTTACCTGTATCTTTGTTGCTTGGACTAGTGAATGGCCTGATCCGAAACTCAACTTATCTATAAGGCTTTCTTTTCCTTTTGATCTCCATTAGTAGAACAAGATGAGAAGAGTCTTTTGAAAGCTAGGCTAACTAATTTTTTTTTTTGTTTAGTTGCTTGATCATATCAATCAGTGGCATGGATGGATATCAACCGAGCTTTAGCACCTTTTTTCATAAAAGAGTTTAAAATAACCAAGACTTATAACTTGCTGCTCTTGTGGGAAAGATATCAAATAAAAAGAAAATGACCTGCTGGTCTAATTAGAGATCTCAAAGTCAAATCTCCCTTCTCCAATCATTAAAATGGGAACAAAAAGATAAAGCTCAAAGATGCGATTTGGTTAACTTTTACCACAAATTAATATATCCCTAGCTGCTTCACTTTTGTTATGAATTGCTTGTATGACTTCATATATGTGTTTATATTGTTGGAAAATTGTACAGGGAAGTATGCATCAGATATCTTGGTTTCAGTTTCTTCCTCATGAACCTGACCTGAACCCTCTACCTGACAAAAGGTTAGATTGAATACATTTTCTTTATTTTTGGTTTGTTGGTTTACTAATACTGAGGCTTCCTTCTGTTCAAACAGTGTTAAAGTGGACCAAAAGGATGCTGCAATGTTACTGGTGCTTTCATCACATCTGCAACTTCAGAAGGAGGGGTTTCTCAGCACATGGACCAATTCCTTTGTTGGACCTTGGGATCCATCCCAGGGATTGCATAATCCAGGTAGAGATTGTGTTGACTTAGTGGTTTTGTGATCATCTGCAATTATGCGTCTTTTTACAACGAAAGGGTGGAAATGGTTACCCATTTTCTAGTTGCCTAATATTTTGTTTGGGGTGGGAGAGAAAGTAAGAAGAAAGAAAATAAACAGGAAAACTAATTTTCTTTCATTGTTTTGATATAATTTATTGTACCGCTATATGTCTGACTATACTCTATGGTAGTGAATGTTGGGTTTTAAAGGGACAAGAGAAAAAGGTGATCGTAGCAAAAATGAGAATAATGTTAAGATATGTGTAGCCATACAAGGAAGGATGGAATATGAAATGATTGTAATTGTATACAGAGAGATGTTGGTGTAGTACCGATTGAGGAAAAGGTGACAAAATCAGTTAAGGTGATTTGGACATGTACCAGGAAGATTACTAGAAGCACTAGTGAGGATGATTGCATGGTTTTTATTCCAGTAAAAAGGAGAGAACAAGAAGGACATTAGAGGAAGTTGTTAAGAGGGATCTTATGGTAAATAATATCTTTGAAGATTTGGTCTTTAATTTCGTGTGATCCATGTAGCTAACCTAACTTAGTGGAACAAGGCATTGTTGTTGTTGTTTTTATTGTTTTCATATAAAGAAAAAGGAGAGAAAAGTGACAAGAAAGAATGTAATCATGTTAAATACTTAAATGACATTTAAACCTTTACAATGTAACAAAATATAAATATAAATGAAATGAGTATATACCTGTCATTTTGCTACTTTTCTTCTGGCTCAATGTGTTTTCTTTTCCTATCATGAGGTGAAAATGTTTAACATAGGATTCGCCCATATTTTTTCTTTGTTTCATGCTTTCTAGCATTCCAAGCAGAAGAAAATGCATTAAAAACCGATTTTCTCATTATGTTTCATTTCTTCAACTTTTCAATTGTTCCAAATGATTGCTAAATTTCTGCCTTCTATATTGAACATGAAGATGAAAAGATTAAGCTGTGGCTTTTTCTTCGGGGGCGTCATTCATCAGTTGTTGAGACTGCTCAGACAGCAGTATCTGGATTAAGAGGTACCGTATATATAACTAAACCTTTGTAATGGTTATATACATTCCCTTCTTCAAAGGTTATGGGATTGGAGTTTTCCCACTTTTGTTTTTCCTGATTATATCTTTACGTTGGATTTTTTTTTATTCATTGAAGTTGTTGCTTCTGGGCTTTGGTTGGCTCCTGGGGATTCAGAAGAGGTTGCAGCTGCTCTCTCTCAGGCTTTAAGGAATTGTATAGAAAGGTATTTTCTCTCTCTCTAAACTCTTGTAAGTGAAATTAATATTGTTCATATCTTGCCATTTTATCTTATTGAATTTGGCAGAGCTTTGCTTGGGCTTTATTACATGAGATTTGGAGATGTATTTTCAAAGTTTCATCAATTTCAAAGAGAAGAAATTTTCAGGTATGTTTTTGCTATATTTAGATTTGTTAGAGTATATAGTAATGCTAGATGGAGTGCCTGTGCGATGCATGGGACAATATTATTATAAAAATTATACGGTAATATTTTTTTAATATAATATAATTTATAAAATATGAATGAAATAATTTCATAATTACTTGAATATGAATATTAATAATAAAATTTGTTTTAAAAAAGTTGTGTTTTATATAATCTTAGAAATTATATAATTGTAATCATAATAAAACAAAATCTAAATATTGATCTCACAAAACATAAAATGCTTCAAATTCCTAAAAAATAGAATTAATGCTTTAAAAATTGTACCAATAATTGAAACAGTGAAAGTAACTGTTTCATAGTTTATTGGTCTGATTTTTAAAACAATAATATAAAATACATGCATCTCTCTCTCAGCCACAAATTCAGCTTGAGGGGGAGTGTCAAATATACGTTTTGATACGATTTGATTGTGTAGCTAAAATTAGGGATTTCTCTCATTATTGTTCATTATTTGATTTCCTTAATTATGTATAATTACAGATTATAAATAAAGGTTTATGTCTGTGGTACAACCACAGAAACAGTAAAATATTTTACAGTCATGTTTGCTTTGACCTTGAAATTGCTAAATCCAAAATCCCAAAGCTGTTTTGACTGTTCAATGCAATTTGTATGTATTATGTGTGATCTAGTTTGTTCACTGCTTTCTGACAGTGGCCCTGCTATCTTCTATTTATAATATTTCTTAATTTGAATTTGCTATTGCATATTTCTGATATTTCCAGAAGAGGACAGCCTGCAGTTGAATTTGTCTTTGCTGCAACTGAGGAGGCAATCTTCATACATGTCATAGTATCCTCAAAGTATGACAGTGCTATTTGTTTTAGTTTGTCAATAGTACATCTTGCATTGAAATATTTACCTATTATTTTCCCTCCCTTTTATCAGGCATATCCGAATGCTTTCCACAGCTGATCTGGAAAAAGTATTAAAACATTCTATGGAGTCGACATATAGACTTCCAGGTTGGCATGCTCTGTGTTCATAAAATTCTGTTGGTTCAAAGGATTACCATTCTTTGATATGCTATCTATCTGTTTATCTATCTATCTTTGCTTGTGCTAAAATATGTGTTACACTGGTATTAGAGTATTCTTACTCCACTGAGTAAAGTGTATTGGTTTTTACTTGGTGTCAGTTAGAGTATTCTTACTCCACTAAGTAAAGTGTATTGGTTTTTACTTAGTGTTAGACACTAAGTAAAAACCAATACACTTAGTGGAGTAAGAATACTCTAATATATAGCTGGCTTTTGTTTTGTGTGTAATGATGGAGAGCAATGATAGGATTTTCAAAGATTGGTTGATGAGGATTCCTGTCCTTTGGGATAGCGTTTTGAATTTGCATACCTTTGCTAATGTGAACATGGTCAATTTTAGTCTATTATATAATTTAAGAGGGTTAGGGGAGTTTCGTTGTTCTGAGAACTAAGTTTTGCTTAGAGATTTTGTTACAGTTGATTGCAGATATTTTGATAATTAGAAGGAATGTGCATCCCAATTTAACTATGCTGGAGGAGACTATTTGTTCTTTTTCTATCTATGTGACACGTCCATATGTCATGTTATTATCACTTTCTAAATGTTGTCCCCTGTTTTAGGTGTCATAATGTTAAGACTGGGGGGGTGTTAATTAGGATAGTTAGAATGAGGGAAGGAGTCAGATGAAGTTTGTGACGTAAATTAATAGAACTGAAGGGAGGGGAATTCATTCAGATCTGTTATCATTTGTAATTTGTGACAGAAATCCTTTGTAAAGGGGAATCTCCTGGAGGAGATCTTTCTCTCCTTTGTTCTGTTCTATTTCAATCTAGATATTCAATCTTTTTCTTTGCTCTATTTTCTAAGCTGGGTTTCTAACAAATATCATATGCTCATGCATGTTCATTACCAATCAGTAGTTAGCAATATCCCAAAGGGTTACTAATTTCATCTTAGTTCTCCAGGTGACTTTTCTTGGCACCATTTGATTTTATTTTACATTTTTTGTGGCAGTAATTGTTTCTCCGCATGGAATTCGTGGCAGCTTGACTGGTTGTTCTCCTAGTGATCTTGTCAAGCAAAGCTATTTCAGGTCCCTTTTTTAACCTTTTATATAGACATTTGATATATGAAGATATCACCCATGACGGAATTCCTTTTTTTTTTCCTTTCTATTTTTAATAGCAGTTCTACCAAATTTAGGGTGTCCAATGGAATTATAGGTCTCCCATATCATGTTTCCCAGGGTGTTGGTTGCCAGCTGAGGGGGCAGAATTGTTATGTTGAAGTAAGTCTTGGCTTCCCTAGATCTGGAACTGACAACACGTTGCAGCCAAACAAAAATAGTGTCAGGAATTTACCCAAACTTCATGTTGCTGAATCTCCTGTTGTGGGACGAAGTGATCACAAGGGACCACCAGATCATTTGTTGGATTACGATAAAACGTTTCTCTATCCAGCTGAGGCAGTGCTTGTGCCAGTCTTACAAACATCATTAGCAAGGTCTTCCTTGAGAAGGTATTTTTTTTTCCATGGACATATGCTTCTATGTTATTTTCTTCCTTTCTTTGATTACGAGCTTTGTGTGTAACTGTTAAGACTCTCTAGTCAAGCTAGTTTTCCTGCTTGATTTTTGTTGATATATGATTTTTATTTTAAATAATAAAAGTTCATTTAGATAGAAGATAGTTACAAGGAAATTTAAAAATAAGGGATCATCATTATGAAATTAAAAGGAAAATTAAAACAAGAAAAAAATGTCAGTGGGGAAAAATTGCCCAATCCCTCTGCATATTTATTTGGAAAACCTCCTTGAAATGACTGCATGCTTGGCTCCCAATAGAAGCTAATCTCTGAATCATATCCAAATCAGCTACATAGAAAACAAATCTCATCAACAATACTGGTGCTATGCTCCAACAATATGCAGCAAATAGTAGCGTGAATTGTGCAAGAGTAGTTAATGATGGGTGGAGCAAAGTAGAGCACAGCCTCCCTGCTCCCAAATGCTCATGCTGGTATGATTCACCATCATGGTGTTTTGCAATAGTGCCCTGTTGCCACTTGAATACCAGTCATACTGTCATAGTGAGCATATCTTCTGTATAACCCCTAGATTAAATAACTCATCACCCATCTGACCAGAATCTGAACTCTAATAGTTGGCCTTTAATCCACAAGCTACCATGCCTTGTCTTCTCTGTTTTGTGTGCACTACACCCATCTTTACTTGTTTTCATTTTACTCTTGTTTATTTTTTTGACACAATTTCCTACACTAGTAATTGGTTCCACCTAAATTACAATTCTTGGTGCCACTTTAGCTGTAACAAATTTATTTTTGTTAGATGAATATGCTGTATGTGCATTGTGATTGTCCTGTCATATGAACCATTATACTTCAATTCCCAGAAGAGCCCTGTGATTCATATACTCTATTAATGTGAAACATACAGATTTTGGCTGCAGAATTGGATGGGACCATCCCTGCCTGGTTCCTCTTCCTTTATTCACTGGTATGTAGTCTTTGCATCGCTCTCTGCATTCATGCATGTTTTTGTCTGAGATGTTTCTTGATACATCTTTGATTATTTTTAAGGTTAATTGTGAACCCTGTTTGAGTGCATTTGTTCTGGTTTTAGCTAAAGCCTCAATTTTCTTCTGCTTTCTGCAGTGCTGGTAATGTGGATTGTACTGAAGATCCTTGGACTGAAATCAATGGAACCCGCACACAAAGTAGTTATGATAGTAGTAGCAATAGCAATAATAGCAGTATTAGTAGCTTAAGTGCCAGCTCTAGTGATAGTGATTACAAGACTACAGGACCAAGTGAACTAGAGGCAGATGCTGATTCTTTGACGTGTAGACAGTCTATGGTATCTTCTGCTGATCAGTTGGACAGTGATGGCCCCAAATTGGTATATCATTATTTACTTTTGAAAAAATAATATTGAACTTTTTTTACTTTAAAAGAAAGTTTACCATGATGAGTAAAAGACAATTACAACGAACTTGGAGGATAATTGATCATCCAAAAGGGAAAATCTAAGCAACAAAGCTACCAATCACTCTGCATATCTGCTAGGGACCTCTTAAAAAACCATGTGCTTTACACACAATATGAACACTACCCTTCAACCAAATGAATCAAATAATAGTATAAACTGCACATTGCCATAAAATTTTTTAGTCTCTTTACTTCTGCCAAAACTACTAAAGCTAATTACCATAAATTTCCCACCTTTTTATATTCTTTGGGATAAAGGCGTTATAATATTTTATTGGATTATGAAAAAGCAAGGTGGTGGTGTAGCATTTTGCTAAATGTAATGTATTGCATGCATAATGCACCGATGCTGATCAGAGTAGTTACCATACCCTTAGGTACCTAATGAGTACATGACAGACACACTCCCACAGTTTAAATTTGAATGTAAAACTGAGACCATCGGTTCTGGATACAGGTACTTGGCCAATTTAGGTTTAGCAGTACCCTTACAACATAGTGATACACATACTTCTCACTTCATAACCTAGCCACTTGCTAGATTGTATGCACATGAAGAAGGCTAACTTACTGCAATTTAAAAGTAAATTGAGAAAAAATATTATTATGCAGTATTTTTCTTGAAAATATGAAAGATTCTTTAACAATATTCCATGTGCTATTATTGTACTGTCTTTTTTTTTTGCTGTAAGAATGTTTTTAATTTTCCTGTTTGCCGATCCTAGTTTTTGTGCAATTGAGCATTATAGCCGCTTTTATTTATCTGCAGGGCTCTAAGCGGTCTCGAACAGGGGTGACAGAGTTGTTAAGTACAGCTACAAACATTCCTGTGCAAGATGCTTACATGTCTGATTTTGGTTCCATGGAAGTGAACAATTCAGCCATTACAGGAGTAGGAAATGAGCCAATTGGGTCTTACTGGGACTGGGATGATGATGATAGAGGCATGGAAATGGATATTCAAGCCCTTCTATCAGAGTTTGGTGATTTTGGTGACTTCTTTGAAAATGATGTTTTACCTTTTGGAGAGGTATCTGAATATTTATACTACGATTTAAAGAGGATTTCTTCTTTTGATTTCCACTTTTTTCTCCTCTATAATCCTGAGTTTTAGTTAGAGTCATTGAGTTGTGACTTTCATTTCTAAACAGGTGCCCAACACTTTTAAACTCTTAAATTGTAAATTAGACATCCAAAGAGTTGTGTTACTCCTTTACAATTATGCATTTTGTTCGTTCTAGCGGTTTATAAATTGGAACAGTATAAGTTTGGTTTTCCTATGGATAAAAATACATACACTACCCTCTACACTTTTTGGTTTGTTTGCTATACCTTAATATAGACCTCCTCGAGTATAAAGAGGGTTTGAAGATCCATGATTCTTATTGCACTAATGAATTTGTTTAGATTGTTCTATCTTCTCTTTGTTTGGGAGTGTAAGGTGTTTTCCTAATTTCTTATGCCTTGTCTCTTCTTTCTTATGTGTGTGTGTAAAATAATGCATTTTATTTCATTTATTATTTTATTTATTGATGATTCTTCAAATTAAAATTTGGATATAAGATATGGTTGACTTCATAGATTGTATAATTTCCATAAATTCACTCGGGATCTTTTTGTTTGACTCATGAATACTTCAAATTTATAAGCTCCTCCTTTTTATTATTCTAATGTTATTGTTCTTTCTCTCTGCTTCTTGGTCTCTTATATATCAGTTTCTTATTGTGTGATCATTTTCTGACAGCCCCCAGGAACTGCAGAATCTCAGGCACTCATGCTTTCTGCCCCGGATTGTGGAGATGTCAACAGCAGTCCAGGTGGTGTGATAGATGTTCCAGATCAAATACTTTTGCCTGTTGGCTTTGCTTCCTTTGAGAGCTTTAACCCCCCTCCTTCAACATCCATAGAAGAATGTCTTAACAAAAGTCAAGATAACTTGAATAACTCCATGTCTTTGGGTCCAACCAATCAAAATCAACTGTTGTATACAAGGGAGTTTGATCATATAATGAAAGCTGAAGCAATGATGACATTCGCTCCTGAATTTGGAGCTGTTGATACCCCTACTTGTGAGTTCTCCACAACATTATTCAGAAGCCCATATTTTCCGAAATCTCGAAAAGCAAAGAGTTCAACTTCAAGTTCAAGCAATTACTTATATGGTGCAGCACCGCCCACTTCTACTTGCACTGAAGGATCAGAGGGGAAGAATGGAAAGTCCGTCAACACAAAAACAGGTTCTGGAAAACATGATGCTAGTACTATGAGTCTCCACTCAAAATATTACTACACTTTTGTGGAAAGTAGGAAAGAAAAAAATGATAAAAATCCTGCCACTTGCAATGATAACAGCATAACTAAATCTGAGGGGATGCCACCACTCTCAAACATTGGTTCTAATGCTATTGTCAAGTCTGCCATAAGGAAGACAACTGATTGCACACATGAAGCAGAGCAGTTCCTTCTATCTGCAAAAACTTTGTTGGCAACTGATATCACATGTATTATGTTGCAAGCTTCCATGTGCAGGTTACGCCATATTCTCTTGTCTTCAGGTAACCTAATGCCTGCTGGTTTGAGTAGGTCAACTGGGGTTTCATTTTTGAATCAGCTTCCTAGTGATCCAAGTATGACAACAGACAACATATCTGGCAAGTATGATGTTAAGAAGAAAGAAAATATACCAATTAGAATTGCAGGTGATATTGATGGAGGAATGCTTGATGGGCACCTTAATGCCCCTGTTGGTGTTTGGCGCACATTAGGAGCTTCCAAAGTTGTAAAACCTTCAAATTCACCTAACATGGAAGTTGTTCCTTCCTTTCCTCATAATTCTTTCAATGAAGAAGGTATCCTTTCTTATGGTCAAAGGAAACCACTTCAGGAGCTTCTTGATGGGATAGCATTACTTGTCCAACAAGCTATTTCCTTTGTTGATCTGGCCTTGGATGTGGATTGTGGTGATGGTCCTTATGGTTTGCTTGCAATGCAAGAACAGTGGAGGCGTGGATTTTGTTGTGGGCCTTCCATGGTCCATGCTGGCTGTGGGGGAAGTCTTGCCTCTTCTCATTCATTGGACATTGCTGGCTTGGAGTTAGTGGATCCACTTTCTGCTGATGTAAGCTTTTATTGTAATGTTTTTAAATGCTCCTTCTATTGCATGTTGTAAATCTCTGAACCGTTTATGTCTCTTTCCTTAGGGGTATGGATTTAGGATTTGGTCTTTGATTATAGAAGTAAAATGAATTTCTACAATTCTAACATAGGATGGATGTTTTTGAAGCTCCCAAAAAACATATATAAACCCATCACAAATCCCCACCAACAGACAAAAACACCCCAGATTAACCAAAGGCCTCCACAAGGTTGGAAGACCAATGATTAAAAGGGATATTAAATTCTTTATCTCTAGCTTTATACCAGGACCAAGCTAGGAATAAGGCGTCATCCATCACTTTGGAAGATACAAAAGGAATACCCTGAAAAATCAGTAAGTTCCTATGCTTCCAAATAGCAAAAGTTAATGCAACCCACCACCCACACCATCTAGAATGGTGTCTCCCTATATCAAAACCATCAGAAAATAAGCTGAAATGACTTGCTGGAGAAGCAGGGAATGCTCCACTAGCCTGAATCCACCTCAAGGATTCCCACCACAGGCCATAGTTTGAGTGCAATGGAAAAAGAGATGTCCTGCCTCTTCCTGTTCCTCATGGCAAAGAGGGCAGGTAGCTTCCTGCAGGCCCACATTTCTTCTTTGGAGATTAGCTCTAGTAGGGAGCATATCCTTTATAAGCCTCCAAGTGAAAATGGCAGCCTTTGGGGGGATTTTTAATTTCCAAATAAGCTGAAAGTTCCTCCCATCTAAGGCAAAACTATTTAGATTTGACAGAAGTCTATAAGCTGATCTAGTAGAGTACCTCCCAGTAGGATCAGCTTTCCACAGTAAGTTATCTTTCAGATGACACTGAATAGGATGAGCACTGATCTCATCCATGAAAGCCACAGCTATTCCCTCCTCATGCTCGAATAAGTGTCTCCTCCATTTCAGATCCCATCTCCAATTGTTCTGGTAGAAGCTACCCATGTTTGAAATTAGATCATTCTGCTGCTTACTAATAATGAAGAGCTGAGGATATTTCTATTCCAAATTGCAGTCCTCCCCAAGCCAGTTGTCTTTCCAAAATCGAATTTTGTCCCCACAACCTGCCTTCCAAGCCATGTTTTGATGAATAATCCTGAAATCATTGTGCTGATATATCCTCCTGAGGTCCCTCCACCAATGAGAATCCCCACAATGAACTCTGCCATTCTGCAAGGCTGGCCATCCTCCATACTTGGAATTTAGAACCCTGACCCAGAAATGATTCTGTTTAGAGTATAAATCCCAAATCCATCTTCCCACGAGAGCTGCATTAAACTTAGACAGATTCTTGATTCCCAAACCCCCTTCCTTCTTGTGAAGACATATTTCATCCCATTTGACCCAAGGGATCTTCTTGTGATCATGCTCCCCTCCCCACATGAAATTCCTTTGTAGAGAAACCAATCTATCAGCTATTCTTTGAGGTAGTTTAAAAAAGGATAAGAGATAGATTGGTAAAGCATTCAAAACAGAATTTATCAAAGTGACCCTGCCAGCCATAGATAAGCTTTTCTGATTCCATTTAGTGAGTTTAGCTTCAAATTTGCTGAGCAAAGGCTGCCATACCACCTGACTTGAGGATTTGACCCCAATTGGGATACCCAAGTAGTGAAAGGGGATACCCATCTGCCTACAATTCAAAAACTGGGTAGCTTCATGATCCCAATTAACAGAATCTCCAAAGATCCCAAACTAGCTTTTAGCATAGTTAATTTTCAGACCAGAGGCCATCTCAAAGCCTCTAAGCATTGCCTTCAAAACAAGAACATTTTCCAAAGAAGCTTGACCAATAAAGACAGTATCATCAGCATATTGGAGGATATTAATAGGCTCTTTCTGTTTTCCCACCAAGTAGCTTCTATAAAGATTTTTCAAAATGGCCTCTCTCATCAATCCAGTGAGACCTTCACCCACTATATTAAAGAGCAAAGGGGCTAGAGGGTCCCCTTGTCTCAGACCTCGAGTAGGGGCAAATTCTTTAGTAGGGCTACCGTTCACTAAAATGGAAATAGTTGCTGATTGAAGGCAGGCAGCAATCCATTGCCTCCATTTAGTACAGAAGCCTAATCTTGACAGCATATAATCCAAGAAAGACCAAGATACAGAATCATAGGCCTTTTCAAAATCCACTTTGAAGACCATGACTGGCTGCTTTCTCTTCATAGCTTCCTCCACCACCTCATTAAGTATCAGAATTCCATGAAGGATATGTCTATCCTTTATGAAGGCTGATTGCCTTTCATCAACAATACCAGAAATGACACTCCTCAGCCTGTTTGCCAAAAGCTTGGCAATGACTTTGTACATACACCCAATCAGAGAAATAGGCCTGTAATCGTTTAAAGATTGAGGATGATTTGATTTAGGAATAAGAGCCATGAAGGAAGCATTGCTGCCTCTAGGGAAGCTTCCATGATAGTGGAATTCATCCACAAATCTCCTGAATTCAGGTTTCAAAATCTTCCAAAAAGCCTTAATAAAATTGAAATTTAAACCATCGGGACCCGGGCATTTATCTCCACTACAACTCCAAACAGCGTCTTTAATCTCTTGGTCAGAAAAAGGGGCAATGAGACCCTCTCTTTGGTTCTGATCAATGGACTGGAAGAATACCCCATCAAGAGTGGGTCTGAACATCTTGTCTTCAGTAAATCTATGAAGAAAGAAATTAAGAACTTCATCTTTGACCCGGTCAGGCTGTTGGACCCAATTACCATCAATGAAGAGACCCTGAAGAGCATTGTAATTTCTTCTGCAATTTAAAACTTTGTGAAAATAAGTTGTATTGCTATCGCCTTCCTTAATCCATTTCTCTCTAGATTTCTGTCTCAGCATAGATTCATAAGCAATTGAAACCTCCCACAGCTCTTGTTGAATAGATTTCTTGACTTTGGCTTCATCTTGAGATAAAGGTCTATCAACAGCTGTAATTTCCAAGTCATTAAGCTGCTGCCTCAATCTCTGGATTTTACTAACATTAATATCAGCATTCACTTTACTCCACTGTTTGATGGTATTTTTAAAGTTTCTCAGCTTGTTTTTAAGAACAATGCCCCCCCATCCACCTTGCTGATCCTTAGACCAAGTCTCCTTCACCATCCTTTGATACCCTTTTTGATTGAGCCACGAATCCAGCACCCTAAAGGGCTTGGGACCCCAATCAACCAGCTTAGTTTTCAAAATTACAGGGCAATGATCCGAGTAATCTCTGTGAAGAACATGCTGGGATGTGTCAGGCCATCGGGCTAACCATTGGTCTGAAACTAGGAATCTGTCAAGTCTGCTCTTGGCAGATCCATTGGGCCTGAACCAAGTGAAATTACCACCAAAACCTCTGATTTCATGGAAAAGTGTTATTTACCAACTTTCACTCTTGAAAGGATTTGCATGTCATTTAAGGTTATGAAATAGACACTAAATAGATACACTGAATTTACTAATAAATTTTATATGTGTTGAAACCTTGTAAATGTAAATTTTGTGTTCTTGCAGAAATATTGCTTGGATAATAATAACAATAGTAACCCTTACATACATTGAACTCAGCTAAACACCGTAACTAAGCTACTTGGAAAATAATAATCATTAAATGCTCCTATTTCCAACAAGGTAAGATGCTGATCCCCTTATGTAGGATAACACCTACAATTTTCTGTTCTATGCTTATATTGACTCCTCCATGTAATATTCTCAATATTCTGGTTTTATTTAGTTGTTCAGCCAATAATCAACTTATCTGTCTATGTCATAAGGATTTGAGGATGTTTTACATTTGTTGTTGATTGCCTAACACCAGCCTCTTAGATTGATTATGTAAATTGACAACTGGTGAAAGGCTTTGTTGTAGTTTATGAGTTTATCAAATTTGAAATCTGTCTAATGATTTATGATGGGATGTTATGAATTCTTTGTCACCTCCCCCTCCCACTTACTAATATTGGTTATTTTATGGGGTTCCTTATTTTAGGGCTTTATGTTATTAGATAATGGTCAATGGTGTTGTTGGTTGGTTTCCGTTCCATTTTTTATACTTATATTCCAGTATTCTGAATTCCCAGGTTCATGCATCCACTGTCATCAGTTTGCTGCAGTCTGACATAAAAACAGCTTTGAAATCTGCTTTTCCCAATTTGGAAGGGCCATTATCTGTAACTGATTGGTGCAAAGGACGCAATCAATTGATTGATACTGGAAGCGTAGTTGATGGAGTTTCTGCTGAATCCAACATTAATGAATGTAGGGATTCTTCAGAACCAATGAGTCCATCTCAATCATCTGTTGGTGGATCTTCCAGTATTAAGGGTAGAAGTTTGCATTGACGTTAGTGATGGAGATTTGTTGTTCATTATTATACTAATTATACTAATACTGTAATACAATTTGATTGCTAGTGCAGTTTCCAACATGATGGATAAGGTGGATGAGACTTCTCAAAGGAGATCTGGCCAAGATTTATGCAGCACAGAGTCAGAGCAGCAAACATGTTCCCGGCTAAAACCTACTCTCATTGCTCTTCCGTTTCCTTCTATACTTGTAGGGTATGTACACTTGTTGTCATTTATGTTTATAGAAAAGTAAGTTTGAATACATAATTGCCTATGGTGCAAGGCTTTTCATAGTATTTGAGTGTATTGGGTGCTTTAGATGATTATGATGCTTCATAGGGTTGTTCATATGACAAGATGAATATATGGTGCTTGGGCTTAAGCTTAATTTGAACATGCATTGCATAAGCATGGCCCATACAAATAAACTAAGGGAGAATGACCAAGTGAAAAGAGTTAGGCATGGATAACAATGGGTAGGGTTTGGGTATGATACTATAGTATCTATTCCCATACACTTGAAGCTTTGATTTTGTGAGGAGAAAACTGTTCACTCACTAGCTTAGCTTTTATTTCATTCAAATAACATATATATATACACAATGGGGAGAGAGACAGCTTGACAAGCTTTGACAAGACAAGACACACTGTTGTCTTCTACAACAAGATAAAACAGAGAAATCTATTCTAAAGATATATCTAATTATAACCCATAATTATAACACATACCAATGTTTAAAAGAACTTTTTCCTGCACTCGTATTTGAATGAATTGCAACTTCAGTGTACATCCCCATGTCTTATATCTACCTATATACCCATACTCGCTTCTATGACATGCATTTTAGTTAAATGAAATATGTTTTCTTTTTAATGTACCTTTTTTTTATAATCTAGTAAAACAAAACTACTGCTTAATATTAAATTAACTATGAAAAATCATCAAAACAGAGAGCATTAATTAAAATTCTATTCCTATGATTGGTTGGATTGGGGAACAGCTGACTGATCTTAACTAGATGAAGATGAAGCCACAAAGACATTAGATGACAAGAGTTAGGAGAAAGTTTTGAAATGAGAATACAATGTGTGGAGACTGATAAGTCACCAACTAATTTGTGTTAATTGTCTAAAAAATTTTAATTTCAAATTTATATATCAAATAACATATGTGCTGAGGCAAGGTGGGTATTAAAGTAATTATATTTGCATCCATCTTCAATAGCTTTATCAGGTAAATACTAAATACCTGGATCCAAATCCTTTTAGTGGGTAATTTTGTCCCAAATTACGGGATATCAGATTGTGTTGCTGTCTTGTGACCTAGAGTTCAATACATGAACACTCACATAAGGCAAATCAGGTGAGATGGTAGGGTTGCTGATTTATGCAATGTTATCAAAACTGGACCGGATCATTTGGTTCAACCGGAAATCCTGCCTGGTGTCCGGTCCAATACACCCATAGAACCAGATCTACTAAAATTATATGACTTGAGTTGATTATGGTACAACATGAAAAATTAGTATAATCATATAAGCACATGAGAGAGAGAGGGAGATGAGAGAGAGAGGAAGAGAGAGTAGACAGAGAGAGAGAGGGAGAGACAGGGGGGGAGAGAACGAGAGAAAGTGAGAGAACCAAAGGGCGACCCAGAGGGAGACACCTGGAGAGAGAGCGGCGAGGACGTGTACAGAGCTGGAGCACTGATCGTGGAGGACAATATACAAAGAAGAAACGCAGAGAAAGGGGATGTGGACTACGAGGAGGACACCAAATCCTTAACCAATGATGGAGAATAGGTTGAAGTAACACGTAGGAGGAACAAGAATACGACAGCTAGTAGAGTGCAATCAAGACCAACGAACCAGCATCGAGCTAATAGAACAGTAACATGGAGGAACAAGGCTGACGTTACAACTTTTTACTTCTCTAGATTCCCATCATGGGTTAACGAGAAGGACTTGTGGTAGACATTCCAAAGATGGGGTAAGGTCTGGGAGGTTTTCATCCCCAAATATAAAAACAGAGAAGGGCAGAGATTCGACTTTGTCAGATATAAGGATGTTAAAGATGCAGTGCGGCTGGAAAGAAAACTAGATAACAGCATTTTCTTTGGAGGGAGGAAAATGTTTGTTAACCAGCCCAAATTCGAAAGGAGCAAGGAGGCCATAAGGAAGCAAAATAGTAACAGTATGCCTGATAATGTCAACTTGCAAGGCGAGGTCAGACGTAGCCAAGGAGGCTCGAAAATGAATGATAATGGCGGAAGATTTCGGTCGTACGTCGAGGTAGCTAAGGTGGCTTCACCGGGAACGAGTATCCTCTGCAACCAGAAGGAAGAATTTCCCCAACTCGCTGAGGTTGCGAAGAGACCAGTGGTCCTCGATATATCAACGGCGCAGAAGGAGTGGATACAGAATGCTTGGGTGGGGCGTTTAAAAAATATAGGCATGTTTGAAAGGTTAGACGAGGAATTCAAATGGGTGATAGATAATGATGTTACCCCGTGTTATTGGGGTGATGATTGGGTCATCTTCCATAACCTGCAAGAGTCTAAGGCCACGCAGATTATACATGAGGAGAGGACGAACGGGTCCACTCCGATCATGGACCTTCAGAAATGGTCCCAGGACATCCGTCCGACACATAGGCTGGCATGGGTTCTCCTGTGGGGTTTGCCGCCGACGGTGTGGGAGCCGGAATCTATGGGAAAGGTGGTGGCGGAAATAGGTGACCTAGTGGAAGTTGATGAAATGGTGGAGAACAGAACGCGGATAGACGTGGCAAGGATCCTTATCAGGACGAAGCGAAGGTTGGGGATCCAATCGGAGGTGATGGCGACCATAGACGGAGCTTCAAACGTCATTCATGTGGTTGAAGACATGTCTTGTTTGGGTGCGCGGCGGAACCTGAAGAGAATGGCCAGTGGGTTTCCGCCATCTCCGTTTTCGACAGAGCCAAACACCCCTGCCACTGTCAGAGGAGATATCCACGGGACCGATAGTACTTTCGAGATCTCCGACAGCACTCCCGGCGACGCTGAAGGATTTTCCACCTACGGGAGGCAGCCCTACTCCCTTCAATCGCGTCGTGACCATTGGGTAAAAGCCATTGGTCAACGATGCTTGGACCGGGTCTTTGACGACCATGGGGTCGTGGACCAACCACCCAACGACAATAATTTTTTGAATTGCCCCCCACCCGTTGATCCTGCTGTGTTACCCGAAGAAAGCTCTGGCCAAAAGCTGATAATGAGGAGTGCATTTAATGATATGCCTCAAGGGGATGGGAGAGATAATACCCACAACTAGGAGGCCCAACACGGAAAGCTTTTTGCGGAAATTCCGACAGGTACAGTCCGAGGTTCAGGTAGGGAGCAGCATAAGTCAACTCCAAACGAAGCAAGTCTGCCAGCTTTTGTTGACCAGCCTTATAATAAGGTCCATTCTACCCCAGATCAAATTTGCGTGGATGAGGGGGGGATATGGGCCCCAGACCTAAAGGCCCAACCCCAACTCCTGCCACTAGATATTATGTTAGAAGAAAGGAGCTTGTGGGGCACAGAGGACAGGCCCTATCTCACGAGGAATCAGCTGTGGACCTTCTAACCTCTCCTAAATTGGCTCCTGAAGTGGTTCCAAATAGCAAATCTCAAGATAATAGTCTCGGGCAACTCAGTTCTCATACCCTGGAAAACCAATATGCCTTAGTAAAACAAATGGGCCTGACCCATGGAGAGGATAGCTTGCAGGTTCGAAGGATGATGTTAGACATGGAAAACAGAGATAACAAGATGGCAGTTGAGATGGGAATTAAATTGGCCTCATTATGATTATTCTCTCCTATAATTCCAGAGGTTTGGGTAGGGGGATTAAGTGGGCTGCCATCAGGAGGATTAATTTGAAGCATAAGGTGGACATTGTGTGTATTCAAGAAACCAAAAAGGAGTCTTTTGATAAGCGCATCTGCCAATCTATGTGGGGAGATTCTTTTGTGTCCTAGGATTTTGTTCCTTCAATTCAAGCATCAGGTGGCTTGTTGTGCTTGTGGAATAACTCAGACTTTCAGGTGGAGAGGAGGGTTAAAGGCAGGAATTTTCTAATGCTGGAAGGGAAATGGGTAAAAGATAATCAGTGGATTCGAATTGTTAATGTCTATGCACCTTGTGACTTAGCTGGGAAAAGGGCATTGTGGGATGATCTGAGGCACCTCAAGGATTTTGATCCTAGTGGTCTATGGTGCTTTCTAGGGGACTTTAACACTATCAGGAATCAGGCTGAGAGAATTAGTATATCTCAGAGGTCTGATGTCACCTCAGATTTCAATGATTGGATATCTGAGACGGAGCTACAGGATATTAGATGCTTTGGTAGCAATTTTACTTGATTTAGGCCCAATGGCAGTGCCAAGAGTAGGCTAGACAGATTCTTGGTGTCTGATCAGTGGGTATCCTTGTGGCCTGACACCTCCCAACATGTCCTTCAAAGAGATTATTCGGATCATTGTCCAATCATTTTGAAAACAAAGATGGTGGATTGGGGCCCTAAGCCTTTTCGGGTGGTGGACTGGTGGCTTAACCATAAAGGGTACCATTCTATGATTAAAGAGGCATGGAGTACAGATCTGCAGGGTGGTTGGGGGGGAATTGCCCTTAAAAACAAGTTGAGGAACCTGAGATTGTCTATAAAACAATGGTGTAAAGATAAGGGGGACATTAAAGCTTCCAAGATTCAGAACCTGAAGCAGAAATTATGTGATTTGGAGAACCTAGCTTCTCATAGAACTTTATCTGATACTGAAGTTATAACCAAGAGAGCTTTGCAACAACAGCTGTGGGATATTTCAACTGCTTATGAATCATTGTTGAGGCAGAAATCTAGGGCTAAGTGGATCAAGGAGGGTGACAGAAATACAGCCTATTTTCACAAAGTGATAAATTTCTGAAGAAGCTCAAGTGCTGTTCATGGTATCCTCATTGATGGTGTATGGGTCCAGCAGCCTGACCTTGTTAAGAAGGCAGTGGTTAACTTCTTTGTTGAGAGTTTCACTGAGCAGAATCATCATAGACCTACTTTGGATGGGGTTTACTTTCCCTCCATAGATCAATATCAAAGAGAGGGGTTGATTGCTCCCTTTTCTGACATAGAGCTCAAGGATGCTGTTTGGAGTTGTGCTGGAGATAAATGCCCGGGCCCAGATGGCTTCAATTTTAATTTTATCAAGGAGTTTTGGGGGCTGCTGCAACCTGATTTTAGAAGATTTGTGGATGAATTCCATGCTCATGGCACTTTTCCTAGAGGAAGCAATGCATCTTTTGTGGCTCTTATTCCTAAGATAACCCAACCACAGTCTTTAAATGACTATAGGCCCATATCTCTCATTGGTTGCATGTATAAAATCATGGCTAAGCTCCTAGCAAACAGGTTGAGGGTAGTGCTATCAGGATTAATAGATGAAAGGCAGTCAGCCTTCATAAAGAATAGGCATATCCTTCATGGAATTATGATTCTCAATGAAGTGGTGGATGAAGCTATTCGGAGAAAGAAGCCTGCTATGATATTTAAAGTGGACTTTGAAAAAGCCTATGATTCAGTATCTTGGTCTTTTCTGGACTATATGTTGATGAGATTAGGTTTCTGCCTTAAATGGAGAAAGTGGATTTCTGCTTGTTTCCAATCAGCTACCATTTCAGTCTTAGTTAATGGGAGCCCCACAAAGGAATTTGTTCCTTCTAGAGGATTAAGGCAAGGGGATCCTCTAGCTCCTTTGCTCTTTAACATAGTGGCTGAGGGTCTTATTGGTATGATGAGAGAGGCCCTTAACAGAGACCTTTATAGAAGTTTCTTGGTTGGGAAGCAAAATGTGCCTACAAATATTTTACAATATGCAGATGACACAGTTTTTATTGGTGAGGCCTCTTGGGAGAATGTTGTAGTCTTGAAGTCAATGCTGAGAGGCTTTGAAATGGTCTCGGGTCTGAAGATCAATTTTGCCAAAAGCCAAATTGGGGTTGTTGGGGTTCAGGCCAATTGGATTCAGGAAGTAACCCAATTTCTGAATTGTAGACAGATGGACATTCCTTTTCATTATCTTGGTATGCCTATTGGAATTAAATCTTCAAGCAGGGTTGTTTGGGAGCCTATGATATCTAAGTTTGAAGCTAAGCTTTCCAAGTGGAACCAGAAGAATCTATCAATGGGTGACAGGGTTACCTTGATAAAGTCTGTTCTGAATGCTCTCCCTATTTATCTCCTATCTTTCTTCAAGATCCCCCAAAGAGTAGTTGATAAGCTGGTGTCTCTTCGAAGGAATTTCATGTGGGGGGGAAACCAACAACACAAGAGAATTTCTTGGGTAAAATGGGAAGTGGTCTGCCTTCCAAAGAGTGAGGGGGGCTTGGGGATCAAGGACCTGGCCAAATTCAATGCAGCATTGAGGGGGAGATGGATATGGGAACTAGCTGCTAACCACAATCAGTTTTGGGCCAGAGTTTTAATCTCCAAATATGGAGGCTGGTCAGACCTGCAGAGTGGCAGAGATAAAGCTTGGCATTCCCAATGGTGGAAGGACCTTAGAAGACTTTATAATCAGCCTGATTTTCACAGTATTCACTAGAATATGGTATGGGTTGTAATCTTCAGCAGAAGTATAATCAACTCTTCATGATCAGGAGACACCAGAATCTTTCCATTTCTAAGATGGGAAAATTATCTCAGAATGTATGGAGTTGGGAGTTCAAGTGGAGAAGGAGATTATTTGACCATGAGTACGAGTTGGTTGTTGCTTTCATGGATGAAATTTCTGATATCTCCATCCAGCATCAGGTTCAGGATACCATGCTTTGGAAAGCTGATTCTAGTGGTGTCTATTCCACTAAGTCAGCTTATAGGCTCTTGATGCCCTCCAACAGTCCTGTTCCTAGCAGGAGGAATTTCCAGATCCTTTGGCATCTGAAAATCCCCCCAAGAGCTGCGGTTTTCTCTTGGAGGTTGTTTTTGGATAGACTCCCCATTAGGGCTAATTTGTCCAGAAGAAATATCCCTATTCAGGATATTATGTGCCCTCTTTGTGGTTGTCAGCATGAGGAGGCTGGGCACCTTCTCTTCCATTGCAAAATGACTAGGGGGCTGTGGTGGGAATCCATGGGATGGATCCAGGCTATAGGAGCTCTTCCAGCTGATCCTGCTAGCCACTTTATCCAATTCTGTGATGGATTTGATGCAAGGAGTAATCATAGTAGGAGGGGCGGGTGGTGGATTGCTTTATCTAATACAATTTGGCAGCATAGGAATTCCTTGCTGTTTCACGGCATTCCTTTTAAACCGCAAAAAGTATTGGATGATGCCTTATTTCTAGCGTGGTCTTGGCTAAAAATTGGAGAGAAAGGTTTCAATACATCATTTAACCACTGGTCTACCAATCTTGTGGAGGCCTTTGGTTAAATTTTGGGTATTTGTATTGGTGTCCTTTTGGTGGGCTTTGTTGGGGTTGTTTTTGTTTTTTGGAAGGTGGTACCTTGGTGCCTTGTATCTGTTATCTTCAGTACCTCTGGTACTGTTTAGTTTATTAATAATATTCATATTTTTGCCTTCCAAAAACAAAAAAGAGCTACTAAAAACCAGGGGTGTAAATTCAGCGACAACCAATGATTCGACAAGCTGAACCAGTTTCTGGAAACGAACCAAAAAAACGAGAAAATCGAAAACGAAGCTGAAAGAGATGAGATGATCTATAGAGAAGGGAGGAATAGTGTAAAAATGAGAGGCATGCAGAGTAGTGCAACTATGGAAGAACTCTGGTGGGCTTGCAGCGGCGCAAAATGAAATGGTGGCTGCAAATGGCACCTCTTGGCATCATCTGCTGGTGGTGGGTTAGAGTTGTGGCTTTGATAGAGGAAGAAGAAAAGGCACCGTACATGTATCTTGAATTTCTAATAAAAGCTCAATACTCTACTGAATAAAAGACAAAAGGTGAAAGACTCAATGAGCATTGCAACTCACAAGGCACATTGATACTTACCAACACGCACTTGTTGCCTATCATCTGCTTTTTACTGTTGCATTTCACTTCATATAATTAATTTTTATAAATTATTATTTTATTTTAAATTATCCTATATTAATCCTAAATATATACTAGTACTTTTCCCTCTAATTTGGTACAGTAAAAAGTAAATTGACGATTAATTATTATCGCAAATTACTCTTATGAAAAAAAATTAATGTATTTAATGTTTGGCTAGGAAAAATTAAATAATAATCCTATGAATAATTTTAATGTTATTAAGTGTATATTATTAGTTAATATATTTTCTTATAAATTTAAAATATTATTTAATAACATAACCGGTTTAACTACTATTGAACCATTGAACCTTGAATCAGTGTCTTGACCGGTCCGTTTTAAAAACATTGGTTTTATGACCTAAAGCTCACAGATTGAAGTCCTGGAAACAGCCTCTCTGTTTGTGGGGCATAAGGCTGCTTACATCTACCTTCCTTAGACCCCAGTTGGTGCGAACCTAATGCACTGATTTTATTTATCTATTATATATTATATATAATATATGAAATTCAGAACTGATTCAAGTTATTTGTCCTTCTTAAAAGCTTATGCTTGTTTAGTAAAAGAGCAAGCTTAGCTCAGCTTTATCTAAGCCATAGCATATGTTTAATACAGTTGAATGCCATTATTTTTCAGAAATTGAACACTTACAATACTTTTTTTTTATCAGCAAAAATAGATATTTATATATATATATATATATATATATATATATATATATATATATATAAAAATAATTGAAGTACCAGAGGTACTTAATACATGGGTAGTGAAGCCATACAAATGGTTCCATAGTCAGACAAAACAGTCTGAATAGGAACCAGACTATGACTACATATGAAATGTATATCTGCTGATTATCCTATTATACTACCCTCTGCTGATACAAAAAGCCCTGTAGGAGGTTACTGGACCAATGATTAAAGTGGATTGAGAAATCTTTCTCCTGGCTTCTAAGCCAAGTCCATAGTAGAAACACTGCAGCCTCAAATAGTTTGTTGCCATTGAAAGTTTCATTGGAGAATAGTATTTTGTTTCGAAGCTGCCAAGTAGACCATGTTACAGCCAACCACCAATACTGCCACCTCTTGACCCTTATGCCGTCAGCCTGAACAAATGAATGTTGAAGGAAATGATGTTTTGGATTTAATGGGAGAGCACCTTTAGTATTCAACCAAGACATGGTATCCCACCAAATAGGTTGGATTTTGTTGCAGTGAATAAACAAGTGGGATGCGTCCTCCTCCACCCTTTTGCAGAAAGGGCATGACATGTCCGAGATCTGCACTTGTCTCCTCTACAAGTTTTTCTTTGTTGGCAACCTATCCCTAAATAATCTCCACGCGAAAACCACAATTTTGCTTGGGATCTTTATTTTCCATAGCTCCTGAAAACATTCCTCCTGACTTCAATCTGCTATCTCCTCCCATAACATATTATAAGCACTGTGTAGAATATGTACCTGATGGGTCTCCTAACCACTCCCATCCATCGGATTGTTGTTGTTGTATAGGCTTGCCTTCAGCCTCTCTAAGGAAACTAACAGTCGAATCAATTTCAATATCAAACAGTGGCCTTCTCCATATAAAATTCCATTCCTAACCATTGTCCTTGTGAGTGCCCATTTGTCTAATAAACTGGTTCTGCTGTGATGAAACCTAGTCTGGGATATTTTTCTGCTAATGATTCCTCCTGGCATATCCACCTATCCTCCCAAAATTTGATTTTATCCCCCCCTCCTACCTTCCATTTTAATCCATTTTGGATAACCGTACCCTGATGTGAATGATGAAGAGCCCTTTTTAAGTCCCTCCACCAAATTGATTCAGGGCCTACCCTACCTGTTTCATCCATACTCCTCCATCCACCATATTTGGACTCCAATACTCTAGTCCATAATTTCCCTTGATGCTGCAAAAGATGCCACTTCCATTTGTCGAGAAGAGCAATATTAAAAGTGTTGATGTCCTTGATGCCTAGTCCTCCCTTTTCTTTTGATAAACACACTGTCTCCTACCTAATCCATGCAATCTTATTTTGGTCAGGTCCTCCTCCCCACAAAAATCTACGTTGTAATTTCACTAGCTTATCCACCACTGATTGAGGCACCCTGAAAAAAGAAAAGAAATAAATAGGAATTGATGTCAGCCCTGACTGAATTAGTGTCACTCTCCCCCCAAAAGATATGTGTCTCTGTTTTCATTTTGCCAATTTTCTCTCAAATTTGTGGATAATGGGATCTCACATCTGACATCGTCTCGAGTTGGCCCCGATGGGTATGCCAAGGTATACAAAAGGAAAAGCCAACATGCTACAATTCAGGTAATTGGCTGCAGATTGCTTCCACTGTTCTGTTACCCCAAATGCTCCAAAACAACTCTTTGCAAAGTTGATTTTTAGACCTGATACAAGTTCAAATGTCCTTAGGATAGCTTTTATTGCTTTGACATTCTCCATAGATGTTTTCCCAAAAAAGGTTGTATCATCTGCATACTGTAAGATGTTTATGCTCACATTATTGGAGCCAACTGAGAAACCTTTGTATAGCTCTCCTCCATAGCTCTCCTCATCAGCCCATTTAGAGCTTCAACTACATCATTAAAAAGGAAGGGTGCTAATGGATCCCCTTGCCTAAGTCCCCTTTGAGGGAGGAACTCAGCTGTGGGGCTATCATTCACCAAAACTGAAATAGATGCAGATTTCATACACCCCTCAATCCATTTGACCCATTTGGAACTGAAACATGTTCTCCTTAGCATGTACAATAGAAAATCCCATGAAACCGAGTCATATGCTTTCTCATAATCTACTTTGAATACAAGGCATGATTTGCTACTCCTTTTGGCTTCTTCAATCACCTCATTGGCAATGAGTGCACTATGTAGTAAATGTCTACCTTCAATGAAGGCAGATTGGGCTTCATCTATGATAGATGGCATCACCTTCTTCATTCTTGTAGCTAACAACTTCGCCACGATCTTATACATGCAACCTATTAGTGAGATAGGTCTATAGTCATTCAATAGATGTGGGTCAGCTACCTTGGGAATTAATGCTATGAAGGATGCATTACAGCCTCTAGAAAAAACACCGTTAGCATGAAATTCATCTAGAAAACGAAGAACATCAGGTTTAAACAGCTGCCAAAATTTCTTAATAAACTTAAAGTTTAACCCATCAGGACCTCGGCTTTTCTCACTCCCGCAATCATAGACAGCCTGTTTTACTTCCTCCTCCTGGAAAGGAGCCACCAACATGTCATTCTGATGCTGATTGATAGTTTGAAAGCTGATGCCATCAAGCCTAGGCCTGTAATAATCTGGTTCTTGGAGTCTTTGTGAGAAAAAAGATCTAACTTCCTCCTTCACCTTATGTGGCTCATCAGTCCATGTTCCATCAATCATGACCCCCTTTAACGAGTTATTCCTACGGTTGACATTCATCAATAAATGGAAGTAACGTGAATTGCAATCTCCCTCCTTGATCCATCTCGCTCTAACCTTTTGCCGTAGCAAAGATTCATGAGCTTGGGCATCTACCCATAATGCTTCCTGTAATTGTATCCTTTTCATGACTTCTTGGGAAGATAGCTCTCTTTGGATTGTGTCTTCCTCCAATTTGTTTAATTCAGCCTCAACTTTCTTGACCTTTTTGAGGGTATCTCCAAACTGTTCCTGGTTCCAAAACTTCAACCTTTGCTTTAATCTCTTTATTTTTTCTTTGAGAATATACCCTCCCCAACCTAATTGCTGGTGAGAAGTCCAGCATTGGTGTACAGTTGCCTTGAAGGAGTTGTTTGCCAGCAATCTAAAATCCTAAAAGGTTTAGGACCCCAATCTACGACCTTGGACCGAAGCAAGATTGGACAGTGATCTGAAAAGTTCCTTGCAGGAGTAACTTGAAAACTGCCAGGCCATCTGCCAAGCCACTCATGGGATACACCAAGAACCTGTCAAGCCTGCTCCTAGATGCTCCATTTGATCTGTACCAGGTGAACTTTCTTCCCAGCCATGGTACCTCTATAACCTCCAGATCATCTATCCAGTCATTGAATTCTTTGATGCTGCTTTCCCCAGATAACCTTTGACAAATGCCAAATCTTTCAGCTGGGTCCCTGATGTTGTTGAAGTCACCCAGGATACACCATAGCCCTCCAGACAATGAATCTTTCAGTTGCTTTACTTTGTCCCATAGTATTCTCTTATTGTGAATGTCACATGGTGAATATATGGTAACTATATTGATTTGTTGTGCCTCTTTGATCCATTCCCCCGTCAAGAGAATAAATCCATTGCTGATGACTTTCCTCTGTAACCTGAATGTTTTTTCGCTCCATAGACATAATATGTCTCCTGCTGTAGCAGGCTGCATCTCCCAACACACCTCAACATCCTCCCATAATGCCTGACACATAGCTTTGTCAATCACTTCTTTCTTTGTCTCTTGGAGACATATCATATTCACACCCTCCTTCTTGATCAATCTTCTAATTGCTGCCCACTTTACCCCCCTCCCTAAGCCTCTGACATTGTATGAAACTATATTCATTGATCCCTTCTGCTGTCTCCCCACCTCTCAAATTCTGTCTTGTCCCTTTCTTCCACAGATTTAATTTTTTCAATGATTATATTCTGCTCATCTCCTCTTGTAGCCCCCATTTGTTTAGCCATGCTCCATATGTTAGCTGCCTCATGAATGTGTTGTGGCTCTGTATTTGCTTCTAGACCTCCATTGTGTGTGGTTGTTTCAGTGATCGGGGCATTAATGGCAGTACATGCAGTCCCAATTTGTGCCAAGTTCTGTGGGTATTGAGCCTGTATTCTGCTCCTATCTATTTCTGCCTCTATAGTATAAAAAGAATGGCCCAATTGGGATTTCTTTTGACACCTCCTTTGTCTTAAATATACCTGCCAGGTATTTTCAATTCCTGCTTTGTTGTTTTTTGGGCTAAGGCCCAATTTGTATGTTTTCGCGGTGGAGGTATAAGTCAAGCTATCCATCCTTTTGTTTGACCCACTTGTATCCATAACACAACTCCCCCCCTGCTCACGTGCTGCTTCTTCAACTACAGCTTCATCATGCGCCTGTTTTTTAAATTTGTCACGTGCACAAAAGTAGTCTTGTGCCTCTGCCTCGTCGCGTGTTGCAGCTACTTATGTCTTCTGGGCCTCTGCCACGCCACATGTCTCCCCCTTTTCTTTGTTACCATTTCCGTGAAAAGTCTGCTCCTCTGCTGCCTAGTAGGTAAACCCATCATTGAGTGGTTGCCCCTGATTGGCTAATTCGTCATGGTGGTAGGTGGCCTATGCCTGCCGCTCGTGACAGTCCTTATGTGCAGCCGTAGGTGTTCGCTGTTTTTCGCCAAAACCCCGCGGACTATTGCTAGTATCCAGTGGGTAATTGTAGCCACTTTGTCCGCTGGTTAATAGAGTCATGCCGAGATCTGCGACGCCGGCACTGGGCCTAGTAGCGAAGTGGCATGGTGCGGGTCACGTTTCTGGCGCTGACAGGTGTTGCATGTCCTCTTCACACACCACGTTGTCCGTTAGTGTCGGAGTAAGGTCGCCGAAGTGGCTTTCGTCTGAGTCGATCTCCTTCGACGAACCCCCGAAGCTTCTTCCCCTCCTGAGGCAGTCGTGAATAACGCTGCCACACTCCTCGACAACATACACCTTGAAAGTTTCGCCACCAATATGGACATTGATGGTGTGTTGGATTGTCGACCACCATGGGGTCTTAATGAGCACCCTTGCTCTATCTAGTCTCCGTTTTTCCTCAACCTCGTCATCCATATCCACCATATCTCCTATAATAGCCAAAATCTGGTGGATATGTTTCTTATCCTAGGCTTGAAGTGGGATACCCCAGCAGTGAACCCAGGCCAGTCTGCTCCTTGTGCGTAAGTTCAAACTCCATCTTTCCATTGAGTAGAACATAGATTTGCCTCCGTCTTGTCCACCATTCATGATTTGTTCTGCACCAACGTCATTGAGGCCAAGTAACAAGAGTAAGTCATCTCCTATGTATTTGGGAGTTATATCCAGTCCTATCTCCCACAATAAATCGTCCTCCAGTCTTTCAAACATAGCTGGGTTTTTTAGGCACCCTACCCACACATACTTCAGCCATTTGATGTCTTCTGGTCCCAATGTTTATGTGGATCGAGGAAGTTGAAGTTTCACGGCTGTGGGTTTGGCTGTTGAGGGCCAGTCGTTGTCCCGGGGCCCTGTTATTTCTTGTTAGCGTGTCCATGTATGAAACTGGGTTTGTTTGGCGTGCTTGTCTGGCTCGATCAGCTTTTATTTGAGTGCTCTCCTCATGCCCCTGTGGTTTGGTCCCTGTAACTGCCTTTCTTGGCATCTCCCTTCCATGCTTGGGGATGTTGACATATAATTTCAGCCCACCAATAACAATCCTGTCTAGCTGCCTTGCCAAGTAGGGAATGTCGCGTACCCCTTTAAATCTGACGAATCCATACCTCCTTCCAATGTAGTTTCTACGATTGGGTATGAAGATTTCCCTGACATCACCCCACTTCTTGAAGTGATGCCAGAGTTCATTCTCTGTAATGTCTTCACCAAAACGAGTGAAGTAAAAGGATGTGATATCCTTGTGGTCCATCCAGTTTGACACAGTATGAAATTGCTGGACTCTTTCCTTGTCTCCTGCATAGTCCCGTCGGGGTGCCTCCCTGATGCTCGCTCTGTCTCTCTTGGCTACCTCACCTCTAAACCTATCCCTCCCCCACCCAGTGTTTCTCTCTCTAAACCTATTCATTCTCTCTAGACTCGAACACTTACAATACTATATGTAGAATAGTTTTAGTTAGTTGATTGAGTAGTAATCCCTATAATTATGCGATATGTTTGTATATGTATAAAAATATACACCACGAGTTGATTCTTATAGAATTCAGCCATAAACAATATCAGTTCTTTATTTTAAGTGCTAAAAATAACCCAATAGATTAATCACTTGCTTATTTGCTGGTATATTGTCCATCATATATTGTATATGATAAGATAGCTAGCACAGCCGGCATCTAGTACTATAAAGTTTGGTATTTAGTTAACCTCTCATGCAAAACATGTCCAATAGGTATCAGGATGATTGGCTCAAGACATCAGCAAACTCTTTGCAGCACTGGGAGAAGGCTCCTCTAGAGCCCTATGCTCTACAGAAACCTGTAAGTTCATTCACTAATGGCTGTGGTTACTAATGGAACCTTGTCTATCATATAGCCTGACTTAATTACCTATAGGAGCATACTTACTCCTATATTACCTGTGATGGGTAATGAGCTTAAGAACTTGCTTTCATTTAAATCATTATATGATATTGTTGGTTGCAGATTACGTATCATGTTGTATGTCCCGACATTGATCCCCTTACTTCTGCTGCTGCTGATTTTTTTCAACAACTAGGAACAGGTAAATAGTGGTAATTATACTTCAATTAACTTATAAGAACTATTTGATGATGATTACAAATTGCATATTTGCAACGTATTATGCTTTATGATGGCTTTGAGAAATTTTGTATTTAAGTCAAATTATTTATCATGTATTATTGACAGTAAAAAGGATGGAAGAAAATGTGGAGAAACCATAGCTAAATCCTGTCTCTTAGGAAAGAAAAGTCATATCTTTTTTATGGATGTAGATGCTATTTGAGATGCTTAGACCATTTTGTGTTTCTGAACTTTCTTGTGTGAATGCAACAATAGTTGATTGTTGTTGCAGAGCACAATGCTTCCAGATTTCTATGCATTGTGACTGCGGATTTAAGTTTCACCTTTTTTTTCCCTTCACTCAGATTTTACGTATTGATTTGTTAGTTTAGTCTTATTTTGCTTTTATTCTTTTCAGTGTATGAGACATGCAAGCTAGGTACTCATTCACCTCAAGGCTTGGGGAATCAGATAGAGATAGAGTCTGCAAAGTTGTCATCTTGTGGTTTTGTTTTACTTGATTGCCCCCAATCAATGAAGATTGAGAGCAGCAATGCATCTCTTGTTGGTTCAGTAAGTGATTATTTTTTATCTTTATCTAATGGTTGGGACCTGACTAGTTATCTCAAGTCTCTTTCAAAGGCACTTAGAGGTTTGAAAATTGGCTCTTGCTTTTCTACAAACCCCAGCGAAGGAAGTAACAGCTCATGCTTGGTAAGTGACTTGTTTAGTGGCTCAATTTTGTAGATTGAATGGTTATCTTGTTCCCTAAGTGCTAATCAGACCAAATTATGATGAGGCTTTAACAGCCTAGTTGGTTGTTTGAAGCATTAATGGCACGACACTGCTCATATAAGAAAACTGTTTTCTCAAAGCATATTTCTCTCTTTTTATAATCGTAGATCTACATGCATTGATGCTTTTGATCTCATTTTTTCCTTCTTACATTTAGGTAATCTATGTGGTGTGCCCATTTCCTGATCCCACTGCAATTTTGCAAACTGTAATTGAGTCTTCTGTTGCCATTGGATCGGTTGTCCAACAGTCGGATAGAGAGAGGAGATCTAGCTTGCATAGTCAGGTTGTGAAGGCATTAAGTGGCTTGGCCACTGTCGATGAAGCTTCAGCGTCTAATATCCTTGTGCTTTCTGGGTTTAGTATTCCAAAATTGGTCTTGCAGATAGTTACAGTGGATGCCATTTTCAGAGTCACAAGCCCATCTGTTAGTGAGCTTGTCATTTTAAAAGAGACTTCCTTTACTGTATACAGCAAGGCTCGTCGCATTTCACGTGGAATCTCTAGTGATTTTGCTCAATCTGCATTTTCTAGTAGATCTCATTCTGGTTTGACACAAATGCCTTCTCCCATCTCTGGGATGTGGAAAGACTGTGTTGGTCCTCGAATGGCAGGACATTCTCTTCCAAGAGAGGGTGACATTGATGCTAGCTTGAGGCCTGGTACTTGGGATAATTCTTGGCAACCAACAAGGACTGGGGGGTTAAGCTGTGATCCAAGTAGAACTGGGGATAATTTTCTTCATGATGAAATTCGTTACATGTTTGAACCCCTTTTTATTCTCGCAGAACCAGGTTCTCTTGAAAATGGTATTTCAGTTATTGGTAGTCCCACTTCAGAATCTTCCAAGGCATTGGCAGATGACAGTAGTGGTAACTATGTACAGAGTACAAGTACAGCAGGAAGTGTGGAGTCTGCTTCAAGCACTGATGCATCTGGGTCCGATCCAAAGACTCCTCCAAGCCTACATTGTTGCTATGGATGGACTGAGGACTGGCGTTGGCTCGTGTGCATCTGGACAGACTCAAGGGGAGAATTGCTGGACTGCAATATTTTCCCCTTTGGTGGAATAAGCAGCAGGCAGGATACAAAGGGTCTGCAGTGCCTATTTGTTCAAATTCTTCAACAAGGTTGTCTGATACTTCAATCTTGTGATCCTGGCCTTGCAAAGCCTCGAGATTTTGTGATAGCACGCATTGGAGGTTTTTATGAGCTCGAGTACCTTGGTAAGTCTGTCTCTGCCTCTTGTGAGATATATTATATGTAAATCAGTTTTGGGGCATGGAACATCCCCTCATGTAAAAAGACATGGCTGATGACATTGACTATGACATACGGTGTAATTTGCTGGTTGTTGAAAAACACTTATACAGTCCTAAAGATAATGATCGATACAAACGTTGTTACATTCATGTGTGCATAAATTCCCTGATACTGGCCATACCTATACTGATACCAGTGTCAGTTGATACTGTTGAGTGAGAATTATTGCAAAGTACCCAGTGGTGATGTTATTTGCATGGTTATGAAGTGGTTGGTGGGTCATTTTTCAAATTCTAATATATTATATACCACAGGACTTCTTAATAAAAATCATAAAGTTTAAAACCAGTTCCATTGAGATTTGGGTAGGTCCAGTGTAGGTTTTGATAAGTTCAGGTACCTGCCAATATGTGATGTGTAAACGTAACAGTGGTGTGCAAAAGCTTCCTTCTGAATTGTGTGAAGCAATATGTCAGAACAAGTGAGCATCTCACTTGTAATAATGCAATTTAGTTGGCTCCATTTATTTTAGTACATTCACACAAAATATTTTGGATTAAAAGGTTAGTCACTTTTAATTATCTAATTTATTTCATCAAATGTATATAAAAAATTATCAAGTGATTTCATGTCAGAAAAGTTAAGAAAATAAATTAGAAAATACATTATCTCCTCTGTCATATGTTATAACTTATTATTATTAGAGCAAATTACACTAAAACCTCCTGTGGCTTCTTCAAATTGCACACAATACCTCTGCCTTTTAACGTTCACAGTGACCCCCCTATAGGGGTACACATGTAATGTATTTATTGAGAAACAGGGGGTGTCACTATAATGTATTTTAAACAATTAAGGGGGTTATTGTAGGGGCTAAAAAGGTAAGGTATCAGTTTCAACTTAAAGAAACCATTGGGGGTGTCAATGTAATTTTCTCTTATTATTATTATTATTATTTGTTTTAACCTGTGACATGTAAGTTAGAACAGAGTCTTGCATTTTGTATATTTTAATTTTGTTTTGTACATATGATTCCATTCACTGTTTAGTTTATCAAAATGAACTCTATTTCTTTTTGATAATGATGAATTTTTCTAAAAGTTTAAGGTATAGTCCCCGATGTACTTGAACATGTGGGTTTAGACCATGTTCAAACTGTTCCAACCTTAAAGTTATACATAAACACACACAAAGGTGCATGGTTGGTTGTTGGCATATTTACATCCATCAAAAGTAAATTGGCTTTATATTTACACCACTTTAAGTTGAGAAGCTAATTGGAGTACATTTGCTCCTTATGTCTGCATCTATACATGCATTCTTACTTTGGCATTTTATTCAAACAGAGTGGCAGAAAGCCATTTATTCAGTTGGTGTATCTGAGATGAAGAGATGGCCTCTGCAATTGCGGAAATCTATGTCTGATGGGATGTCTGCAACTAGTAATGGTTCTTCTTTGCAACAATCAGATATGAGTTTGATCCCAGAAAGAACTCTTCCTTCCTCACCGAGTCCTTTGTACAGCCCTCATACCAAATCCCCTGGTTTTATGAAAGGCAGTTTAGGGCAACCTACTGCAAGAAAGCAACTTATCGGTGGACATTCTATGGTTGACAATTCTAGGGGTTTGCTTCATTGGGCGCAAAGCATTAGTTTTGTTGCAGTTTCCATGGACCATACTTTACAACTAGTTCTCCCAGCAGATTCATCAACTCCTGGTGAGTTATCTTTACTAGAATAATAATAATTTTATTTTCTATGTTCTTTTCTTATTTCATTAAATTGCATTCATGAGATGAGGGTGTTCTGATGGTTGAGTAGGCGCACAAGAATAGATAAGATCAGTATTGAAAATCATGATGCGAGAAAATGAAAGAAAAGATAAAGAGAGAAATGTTACAATAACTGAAGGAAATACTGAGGTATAAGCTTCTGTCAAAAGCAAACTGATAGTTAATTGATAGCTGTTAACACCCCATTTCAACTACAATGGGTAGGGTAGGGTACTATGGCATTTGTCCCCCCATCCTCACATTTTATAAAAATACTTGTCCCCAATCCTGCGTGGAGAGCAAATTTAGTGCCTATCTCTGTATCCTCTTAATACTTGTCCTCTTTTCTTCAATTGTAAAATGACAAAAGGCCTGTGGTGGGAATCCATGAGATGGATTCAGGCAGTAGGTCCTCTTCCACTTTCCCCAGCTTGCCATTTTACTCAATTCTGTGAAGGTTTTGGTGCAGCTGTGAACCATAGCAGAAGGTGTGGGTGGTGGATTGCCCTAACTAGTTCTATATGGCAGCACAGGAATCTACTGATTTTCCAAGGAAAATCTTTTGATTCCTCTAAAGTCATGGATGATGCTCTCTTTCTAGCTTGGTCATGGTTAAAGGCTAAAGAGAAAGATTTCAACATTAGTTTTAACCAATGGTCTTCCAATATCTCGAATTCCTTTGGTTAGTTTGGTGGGGTTTTCATGTTCTTGTTGGATTAGTTTGGGTTTTTCTTTTGGAGGGCAGCACATTGGTGCACTGTAATTTCTATCTATGGTACCACTGGTACTGTTTTTCTATTAATAATATATATCCTCTGCCTTCCAAAAAAAAAATACGTAGATACACTTACCTAGACCGAGCAGTCTAGTACCCACGCTCAGTTAAATAAAAGTGCTATGTCATATCCTAATTATAAAATATGACAATCAAAATATTTAGTTAGATTCATATCTATGAAGTGATTAACTATAAAACATAAGTCCAATTATGTCTAATAATTTTATATTCTTTGACTTTTCAAATTCATAGAGATTGGTATATGTAATTTTAGGAAAAGAAAACGATCTTTATTGATCAAAATTACATGAGAATTATATTGGCAGAGGAGGCTAGGAGAGATTCTACCACCCTGTTTAGAAAAAAAAATATAATGATCAGATAGTGAGAAAAAAAAAAGGAGAATAAAATCAAATCAGATTTGAATAATCTAATCTTGATTGTTTTCAACTAACTAAATCTTCTAGAATAAAGGAGAATAAAATCAAATCAGAACTGAAAAATTTTATCTGGATCTTATAACTACTTAAATCTTCTAGAATTCTTATTTGAAATTTAAATTTAAAGTAAAACACATACAACTGTCAATACCCCCTCTTGTTGGTGCACAGATGTCCAACATGCACAACTTGCTTATAAAGAAATCAAAATTAGGTCTAAATCCCTTTTTAAGAATGTTTGCCACTTGCTGTGAAGTAGGAACAAATGGCATGCAAGTTAGGCCTGCATCCACCTTCTCTTTTATAAAATGTCTATCAATTTCTATACGCTTGGTCGGGTCATGTTGAACTGGGTTCTGGGAAATATTAATTGTAGCCTTGTTATCATAGTACAACTTCATGGGGAGTCATTGGAAGTTGTAACTCTTGTATAATTTTTTTTAGCCATAAACTTTCACAAACTCCTTGACCCATTGCCTTACATTTAACCTCTGCACTACTAGCCGCATCATTTTGTTTTTTGCTTCTCCAAGTAACCAAATTTCCCAAAACAAAGGTACAATAGCCAAATGTTGACCTTTGGTCAGTAATGGATTCAGCCCAGTCCACATTTATAAACACTTAAATGCCTTGCTGCCCAGTTTTCTTAAAAGATAGTCATCTGCTCAGAGCATTTTTTAATTACCTCAATCCTGTAATCTACCTTAAGGTGTTCTCCATGTGGTGAGTGCATGAATTGACTCACTAAACTTACTGCAAAAGCTGTCTGGCTGGGTGGCCGGATATGAGATAAATAAATTAATCTTCCCTATAATCTTAGGTATCGAATTGTTTGTACTGGATTATAGGAGTTTTAAGCTTGCCTGCAACCCACATCTCAATTCTTTGAGAAGGTCTAGAACATACTTTCTCCTTGACTCAATAACTCCTTTCTTTGATTGTGCAACCTATCCCCCAAAAAACATTTTAAGGACCCAGGGTCTTCAATCTCAAAATCAGCTGAAAAGGGAAGCTTTCAATTTATTTATTTCATTGAAATCATCCCCAATAATGACAATGTTGTCCATATAAACAATTAGCACTGCCCAGAAAAATGGTTTACAAACTTACTGTCACTTTGACATTGAACATACCTTAGCTACAGCTACACATTTGTGACGATTATGTTTTTTAAAGTTGCGTTGCACCAATATACCAAACTCCTGTTTTGGCATATATGACATAAACAAAGGAAATTCGGACAAATATCCTTCAAGGACTTGAATCTATTATGGATTTGCAGCATGGTAATGATAATGATAGACTTCTTCATCATTATTCTCAACTTTGGATTTCTGGATAAAAAACTTATAATTGTACAATGGCATTGGAGAATGAAGTTTCACAAGCAACCATGAAACAAAATTTTGAACTTGTGATGCTACCTCATATTTGAATATTCTTAATTAAGTAAATCATGTAAGTATTGATAGCAAAAACTTTTGGAAGAGGAACTGAAAGTGGTGGTGGTGGTGGTTTGTCTATTTCAGGTTACATAGAAGGTTTCACTCCAGTTAAGTCTCTTGGTTCTACGTCTTCTGCATATATTCTAATTCCTTCTCCTAGCATGCGGTTCCTTCCTACAACAGTTCTTCAACTCCCAACATGCCTCACAGCAGAGTCGCCACCACTAGCTCATCTGCTTCACAGCAAGGGTTCTGCTCTTCCTCTCTCTACTGGATTTGTGGTTTCAAAAGCTGTACCTTCCATGAGGAAGGACTATAGAGCCAACCAAAAAGAAGAATGGCCCTCTGTACTTTCAGTGAGCCTCATTGATTATTATGGAGGTACTAACATCCCCCAAGAGAAGATTGTCCGAGGAATTAACAAGCAAGGTGGTAGAAGTTTAAGCTGGGAAGCTAAAGATTTTGAAATTGAGACCCACTTGGTTCTGGAGTCTCTTGCAGCAGAGCTTCATGCCTTGTCATGGATGACAGTGAGCCCCACATACTTAGAGCGGCGAACAGCACTTCCTTTCCACTGTGACATGGTTCTAAGACTAAGAAGACTTCTTCACTTTGCTGATAAAGAGCTCTCCAAGCAGTCCGAGAAATCTTGACTATGATTTATATACAATGATGGCGACAAAACTCTGCTCATTGGTTAGAGAGTAAAGACAGCCAAACTGAGCAGAGTAGACTGTATCATACTAGATACACTGTGATTACATGTAAATTAAGTGTAGAAGCTGATGTGTTCCTGGATGAAATAGATTTAGCAATTGTTGGGAATGTACATGAGCTTTTCTAGAACAGCTGATTTTAACCCAGATTAGCATGATTGATAGAAAATGTTGATTGTAGCATAGAATACCATATCCTCTATCTTTGTAGTATGTTGGTCTCCGAAATTTCATTTCCTTTGTAATTAGATATTGTAAGACAATAATGTGCCAGTAACTAATTTTATGCTTAATATTGGTTTCGTGGCTATAATTATTGTGTTGAACAATTTTTGGGGTGCTGTAAATTATTTACAGGTTCTGGACTGCCACCATAGTTGGG

>GmMED13-2

AGCACTCGGTGGGATCAGTGGTGCAATCTGAATCAGATTCGGAATTTTCGAATGGAATGGGGCGCATTTGTGGTTGGTTTGTGCAGGAGCAAAAGCAAAAGCAAGTAAACGTATCATAAACCCGCGTCCCATTCTTAATCCTCCTAGGTCGGAGTCTTAGTCTGACACAACACAAACAGATCGACCGTACGGCATCATTTACAGCCAAACCCTAATTCAATTTCACTGAAGCCTTCATACCATATTCCAGATATGTGGACAAATGTTTTCAAAATCGTAGGGTTTTCTACTTCTCACTTTTTTTTTGGGTGATTACTTTTATGCTGCGACTTAATATGATTAATTTCGTTTTACCCCTTTTCTTATCTCGTAAGCTGACTTTGTACTGTACTCATCTCATGAATAAGTTAGTGCAAACATGTTTAAGGACTAAAAGTATTGTAAATTATCGCATACTTAGTCGATACAAGGTGTTTATTTTAGAAGTAAGTTTGGTTTGTGAAAGCATATAATTTTAACAAGACATCTTTGAGCACGTTCTATCTTCCCTATTTGATCGAATACTGGAATTGTGGTTTTTCCAATAAGCATCATAAGGGTTGACTGGATAATTTTTTAACTATACTATACCTGCACTGTTGCTCTGTGCTGAAACACAAATGTTTACATGTTTCAATCCCCCTTCCTTAGTGGATGACTAGTTTCAATATCTGCCTGCACATGCTTATAATTATGTTTGGTGTTGCAGTAGGGTACTCTAGCCAAGGGTTACCTGTATCACCCTAATTGCCTGGACTAGTGAATCACCTGATCTGAAACTCAACTTATCTATAAGGCTTTCTTTTTCCTTTTTATCCCCATTAGTAGAACAAGATGATAAGGCTCTTTTGAAAGCTAGGCTAACTAATTTTTTTTGTTAGTTGCTTGATCATATCAATCAGTGGCATGGATGGACATCAACCTGAGCTTTAGCACCTTTTTTCATAAAAGAGCGCAAAATAACCAAGACTTAAAACTTGCTGCTCTTGTGAGAGAGATATCAAATAAAAAGAAAATGACCTGCTAGTCTATTTAGAGATCTCAAGGTCAAATCTCCCTTTTCCAAGGGCAACATCATTAAAATGGGAACACCAAGTTAAAGCTCAAAGATGCGTTTTGGTTACTTTTACCAATTACCACAAATTAATATATCCCTAGCTGCTTCACTTCTGTTATGAATTGCTTGTATGTCTTCATATATGTGTCTATATTGTTGGAAAATTGTACAGGGAAGTCTGCATCAGATATCTTGGTTTCAGTTTCTTCCTCATGAACCTGACCTGAACCCTCTACCTGACAAAAGGTTAGATTGAATACTTTTTCTTTATATTTTGTTTGCTGGTTTAATAATACTGAGGCTTCCTCCTGTTCAAACAGTGTAAAAGTGGACCAAAAGGATGCCGCAATGTTACTGGTGCTTTCATCACATCTGCAACTTCAGAAGGAGGGGTTTCTCAGCACATGGACCAATTCCTTTGTTGGACCTTGGGATCCATCCCAGGGATTGCATAATCCAGGTAGACTGTTGACTTAGTGGTTTTGTTATCATTTGCAATTATGTGTCTTCTTACAAGGAAAGGGTGGAAATGGTTACCCATTTTCTAGTTGCCTAAGACTTTGTTTGGGGTGGGAGAGAAAGTAAGAAGAAAGAAAATAAAAGGAAAGAAAATGAACAGGAAAAATATTGTTCTTTTTATTGTCTTGATACAATTTATTGTACTGTTATATGTCTGACTATATTCTATAGTAGTGAATGTTGGGCTTTAAAGGGACAACAAGAGAAAAAGGTGAGAGTCACAAAAATGAGAATAATGTTAAGATGGATGTGTAGCCATAAGGAAGGATGGGATATGAAATGATTGTAATTGTATACAAAGAGATGTTGATGTAGCACCGATTGAGGAAAAGGTGACAAAATCAGTTAAGGTGATTTGGACATGTACCAAGAAGATTACTAGAAGCACTAGTGAGGATGATTGCATGGTTTTTATTTCAGTAAAAAGGAGAGAGAGACCAAGAAGGACATTAGAGGAAGTTGTTAAGAGGGATCTTATGATAAATGATATCTTTGAGGATTTGGTCTTTAATTGAGCTGGATGGTGTCATGTGATCCATGTAGCTGACCTAGCTTAGTGGGACAAGGCATTGTTGTTGTTGTTTTTATTGTTCTGATGTAAACAAAAAGGAGAGAAATGTGACAAGAAATCAATGTTGTTAATGGCAGATGGCGGTTCATGGCGGAAAGCCAAAAATTCGCCATAAAATATGGCTAATGGCGAAACCGAAAATGGCGGACGTCATGGCGGACAAAAAAAATCTATGTATATATATATATATATTTATTTATTTATATATACATATAAATGAGGGCGAGCCCTGATGCAGCGGTAAAGTTGTGCCTTGGTGACTTGTTGGTCATGGGTTCGAATCCGGAAACAGTCTCTTTGCATATGCAAGGGTAAGGCTGCGTACAATATCCCTCCCCCATACCTTCGCATAGCGAAGAGCCTCTGGGCAATGGGGTACGAAGTTTTATATATACATATAAATATTAACAAAACAATAGTTAAAAAATAAATATAAATAAAAAATTAAAAAAACAATGGATTGCATTCAAATAAACTATAAAAGTTTCATGAGTTCACACAATAACCAAGTACCAACACCAAATAAACTCATTCAAGAGTTCAAAATAACAAAATGGTAAAAGCATAATGCAAAGTGCAAAGTAAAGGTTGAGGCTCTGATCATCTTCTCCAATCCCAACATAATCATCTTCGTTGTTATCATAAGGTAGTGGAGCATCTCCCTCTCCATCTTCTCCATCGACGCCTCAAAAATTAACCATGATTTCTTCTTCTTCAGATTCAAGTTCAATATCCTCAAACTCTAGATCCTCATCATTTTCCTGGACTGTTTTTTGCTTCCTTGATGAAGATCCAACAACCATTGCCCGTTTTTTAGAAGTGCGGGCAGCAGCAGTAGTAGGACTAATAGCAGCACTTGTTGGTTGCTTTCTTTCCCTTTTTTTCCGCCTAGTATACATCCTACACTCTCCAACCCCCGAAGTCTCATACACAGTTGCCCAATTTAGTGCATCATCATCTTCAAATACCAAATCATTTTCAGCATCATTATCATCATCTTCATCCATCTCTCCCATGAGCCATTCATTGCACACATCAATATCATTAAGAGAAATTGGATCAATTTCATCTCTTGCATTATATCTTTGCTTCAATTGTTGGTTGTATTTGACAAACACCAAATCATGCAACCTCTTGTGCTCAAGCCTATTTCTTTTTTTTGGAATGAATCTACAACATAACAAATAAATGTTGATCTCAATCTCAAATACACTCCAAATAAAACTTGACCATCAAAATTAAATAAAAATTAAAAAGCATAGTTAGAGTTTTGTCACAATTACTTGCTCAAATACACTCCAATTTCATTCACATCTTGAAGCACTGCAAGTCAAACTCAAAATTTTAATAGCTAGCTTCTACAAATTTGGAGTTTATGACCCAAACATTCGTCACCAATATGTTGCAACACCAACCAAGAGCATAACTTAGAATTCTGAATTATAACATTAATACGTAAAAAATAGTAATATAGTATTTGTATTTGTTATTTGGTAAAAGTTTCTTACTAGGAGAATGGGTTTTCCTTTGAGCCATTGCAAAGTCAGAACCAAAGTGTTTAGCACCAATCTTGTAAAGATGCAACTCAGTTAGAATTTTCTGTTGCACATCACATCGTGGAACCAACTTCTTAATACAATCAAACAATCCATTGGTGACCTCAAAATCAAACTTCAAATCAGTGTTGTCATAAAAGAACTCTAGATTTAAGAAGTGGGCATCTGCATGCAATGACCTATGAAGTTGACAATTCCATCTTTTATCAATGATTGCAAACACATCTTTGTACTTGCTTTCATTGTTGTTGAAAGACTTGATAATTGTTTCTTTTGCCTTGTCCATTGCTTCATAAATATAGCCCATGGCTGGTTTCCTTTCACCATTCATAAGACGAAACACTTTGACAAGTGGAGTCATGACTTTAAGAGTGTAAACCACACTATTCCAAAAAGAAGGCACGAGCACTACCTTTGCTGCTTCTTTCCCCTTAGGCTCCTTAGATAACTTGTTTAAGATCCATTCATCAGAAATAAACATCTTTCTAATATTAGCTTTTTCTTTGTGGAGCCTTTCCAAGGTTAGATAAGAAGTGACAAATCTAGTAATAGCATGTCTCACCAATTCCCTGTTGTTTGTACAATTTCTCAACAAGCTTAAGGTACTAGAATGGGCATAGATAAACCCAACTAGATTAATTGCCCTTCTAATTGTCTTCCTTATCAAGGGAAGCTTCCCAATGTCTTCAAGCATTAAATCAATACAATGAGCTGCACAAGGAGTCCAATAAATGTTTCCTTTTGTCCTCTAACAACTTACCTGCTAAAACATAGTTGCTCCCATTATCAGTTACAACTTGAACAACGTTCTCTTCCCCAACTTCCTCCACAATAGCATCAAGCAACTCAAAAAGCTTTTCACCTGTCTTTACAAAATCAGAGTCATCAACAGACTTCAAAAACATTGTACCAACTTGAGAGTTAATAAAAAACTAATGATGCATCTTTGTTTCCAATCAGTCCATGCATCGGACATAATAGTACAACCATACTTGACCCATTGCTCCTTGTGGCCTTTCATCAAATTTTCAGTGTATTCAACTTCCTTCTTCAGGAGTGGAACTCTGATGTCATGATAGCTTGGAATAGGAAAATGTGGCCCATATTGACCAATGGCTGCAACCATATTCTCAAAGCTTTTCAATTTAATGAGGTTGAATGACAAACCAGCTTGGTACCAAAAGCGAGCAATATATTGATGCACCTTCAATACTTCATTCTTATCCACTGACTCTCTTATGTTCATTTGCCTCAACATTTCTATTTTTCTCCGATTGATTGCATTTTCTAGATTCTTACAAAATCTGTCCATTGGTCCTTTTTTAGTCCCACACTTTGTCTTTGCAGCTAAAGCAGTAGCATTGCAAGAGTCCGCAAACTCATCTTCACTTTCATCACATCCAATTGGTTCACCAAATTCAAAATCTCTTATATTTGCCACATTACCATTGCCAGAACTACTGTAAGTGGTCCCACTTTTTTCCACTTTTTTTGTTAGCCATATATTCCTTTAGCTCTTAAACTACATTTGGTGAAGTTTTCTTGCAAGATGCAATGTTACCCGACTTCCCAATCAAGTGTTCTTTGGCTCTGGTTATTCCTCCCTTTGTGATTTTTCCACAGTAATTATAAACAATGGTGTTTGTATCCCCTTCCATAAGTGGATGACAATACTTCCAACCAGGGTTTGTCTTATTTTTCCTTGTTGCAGTTGCAACATTAGATGATGGTTGTTGAAGTTAAGGACTACATTGTTGAAGGGTTTGAGACACACATTACATTAGAGAAGTGCTGAGTTACTTTTCTCAGCCACACATTGTATATATTTATATAAATCAGAAAGAGTACAATAAGAAAGAAGAAAAGACAGGTTCGGACTAGGGAACCTGCTCCTACTTAGGCTTTTCAGAATCCACACTAAAAACAGCCTTTTCTAAACCTGTCAAGACTATTCTTACAAAGATACAACTAGTACACAATACAACTGTCTACATACAGCTGTCTACAAAATATAATACTTTAACAATTTTCAGCCATTTAGAAGAGGGCTGAACAGAAAAAAAAAAGCAGAACCAAAAATAAAAAAGGGTGTCAAACAGAAAAAAAACCAAAAAAAAAAACAGAACCACGTTGCTGTCGCAGACTGTAGCCGTTGCCGTCGCCGTCCGTTCGTCTTGTGCGTGGTGCTCGCTTGCGTGGCGCGCTGGTGCGGGGCGCCGTCGTCGCTGGTGCGTGGTGCTAGTGCTGTCGTCGCACTGGAGTTTGCCGTCGCCGTCATCACTCATGTCGTCGTCGCGCTGGAGTTCGCGTGGTGCCGCTGGTCGCACTGGAGTTCGCATGTTGCTGTTCGTGCTTGCGTTGCTCATCCCATTTTCATGCGAAAATCGGGTTAGGGTGTTGGGTCGGGTCACCCCAACACGACCCAATCTGTGTTGCAAAAATTTCAAAAAAAAAAAGGTGTCTTCTGCCATAACCGCCATTCCGCCACCACTGTTAAGGTTATGGCGGCCGCCATATGAAACCCGCAACGCCACCGCTATTTGGTGGCATTTTCCCAAAAACCCGTGATGCCATACCGCCATGGCGCCGTCATAACCGCAATTTAACAACACTGCAAGAAATAATGTATTCATGTTAAATACTTAAATGACATTTAAACCTTACAATGTAACAAAATATAAATATAAATGAAATAAGGATGTACCTGTGATTTTGCTACTTTTCTTCTGGTTCAATGTGTTTTCTTTTCCTATTATGAGGTGAAAATGTTTAACATGGGATTCACCCATATTTTTTCTTTGTTTCATGCTTTCTAGCATTCTAAGCAGAAGAAAATGCATTAAAAATCATTTTTCTCATTATGTTTCCTTTCTTCAACTTTTCAATTGTTCCAAATGATATTTCTTCCTTCTATACTGAACATGAAGACGAAAAGATTAAGCTGTGGCTTTTTCTTCCGGGCCGTCATTCATCAGTTGTTGAGACTGCTCAGACAGCAGTATCTGGATTAAGAGGTATTGTAGATATAACTAAACCTTTGTAATGGTTATATACAGTCCCTTCATCAAAGGTTATGGGATTGGAGTTTTCCCACTTTTGTGTTTCCTGATTATATCTTCACGTTGGTTTTTTTTTTTTATTTCATTGAAGTTGTTGCTTCTGGGCTTTGGTTGGCTCCTGGGGATTCAGAAGAGGTTGCAGCTGCTCTCTCTCAGGCTTTAAGGAATTGTGTAGAAAGGTTTTTTTTCTCTCTCTAAACTCTTGTAAGTGAAATTAATATTGTTCATATCTTGCCATTTTATCTTATTGAATTTGGCAGAGCTTTGTTTGGGCTTTATTACATGAGATTTGGAGATGTATTTTCAAAGTTTCATCAATTTCAAAGAGAAGAACTTTTCAGGTATGATTTTGCTATATTTAGATTTGTTAGAGTGTATAGTAATACTTGATGGAGTGCCTGTGCGATGCATGGGACAATATTATTATAAAAAATATTCGATAATATTTTTTAATATAATATAATTTATAAAATATGAATGAAATCATTTCATAATTACTTGAATATGAAAATTAATAATAAAATTTGTTTTAGAAAAGTTGTGTTTTATATAATCTTAGAAATTATATACTTGTAATCATAATAAAACAAAATCTAAATATTGATCTCACAAAAAGTAAAATGCTTGAAATTCCTAAAAAATAGAATTAATGCTTTACAAAACAGACCAATAATTGAAACTAAATCATTTAGAGAGAGAAGAGAAAGAGTAAGAGAATTCTGTTATTGGGGAAATTCAACCCAATTGAAACTGATTACAAACCTTGCATGGTCTTCAAAGTGGATTATGAGAAGACATATGACTCTGTTAGTTGGAATTTCCTGATTTATATGATGAGGCGTATGGGCTTTTGCACCAAATGGATTAGGTGGATGGAGGGATGTCTAAAGTCAGCAACAGTTTCGGTGCTGGTCAATGGTAGCCCTTTAGCAGAATTCTTGCCTCAGAGAGGGCTAAGGCAAGGAGACCCACTATCACCTCTACTTTTTAACATAGTTGCGGAGGCTTTGAATGGTATTATGAATCAGGCGCTAGAGAAGGGGCTGTTTAAGGGATTTTTGTGTGGCAAGAACAAGGTGGAAATTTGTCTCCTTCAATATGTGGATGATACTATTTTCTTTGGAGAAGCAACAATGGCAAATGTCAGAGCTATCAAAGCAATGTTGAGGGTTTTTGAGCTAGCATCGGGGCTCAAAATCAATTTTGCTAAAAGTGGATTTGGTGCTTTCGGGGCAATGCAGTCATGGCAGAGCGAGGCAGCTGATTACCTCAATTGTAGCTTGTTGACACTCCCTTTCATATACCTTGGTGTTCCTATTGGAGCAAATCCGAGAAGATATCAGACATGGGATCCTATCATCTCTAAGTGTGAGAGAAAACTAGTGAAGTGGAAACAGAGAAACTTGTCATTCGGGGGGAGAGTGACTATTTTAAAATCAGTGCTAACATCTATTCCTATTTACTTTCTCTCTTTTTACAGGGTTCCTTAGAAGGTGGTGGATGAGTTAGTAAGACTTCAGAGGAATTTTCTATGGGGTGGTGATCAGCAGCAGAGGAAAATAGCTTGGGTCAAGTGGGAGACGGTATGCATGCCAAAGGAAGCTGGGGGTTTGGGAGTCAAAGATATTAATTCCTTTAACTTATCTTTACTGGGTAAGTGGAATTGGAACCTATTTCAGCGACCAGGGGAGTTGTGGGCTAGGGTGCTGGACTCGAAATATGGTGGTTGGAGGGGCCTAAGTGAAGGAACTAGAGCACAGGCATAATCTATATGGTGGAGGGACCTAAAGCTTGTGGTTCAACATCCACAACATGGTGTGATAATCAACAACTCAACACTATGGAAGGTGGGCTGTGGGGACAGGTTTAAATTTTGGGAGGATGCATGGATGCGTGGGGAGGAATCGCTGCTAGCAAGATACCCCAGGTTGTATAGTATATCTTCACAGCAAAATCAATACATACAGCAAATGGGGGCCTTTAGAGATAATGGATGGGAATGGGATTTCAGATGGAGGAGACCTATGTTCGATAATGAAGTAGATATGGCGGCCTCTTTCCTAAGGGAGGTCGAGTGTCTCAGGATTCAGGCTCAGCAAGGTGACTAGTGGGTGTGGAAGGCAGATCCGAGTGGCCAATACATGGCCAAATCTGCCTATTGCGTTCTACGGGAGGAGGAGGCTGATGGGAACCTGGATGGGGCTTTTGAGGAACTATGGAAGCTTAAGATTCCCTCAAAGTGGGTTAGAGAATGAGGGTTAGAGAGAGAAACAGAGACAGAGACAAAGATGGAGGAGAAACAGAGACAGATACAGAGATGGAGAGAGAGACAGAGAACGAGAGGGAGACAGAGATCGAGAGAGACACAGAGTCAGAGAGAGAGAGACAAAGAGAAAGGGAGTGAAGGAAGAAGAGAGGGAGCTTGTGCGAGAGAGAGGCCGTTCGAGTGCCAGAGACCTGACAAGGAACCTATATGGAAGGGCTCGATACAGTTTGAGTAAGGAGTGTAGGGGGAGAAGATGTCAAGTCATGGCTGAAAGAAATTCTACCGGAGAAGATGACAATCACAGAGTCACTGATAATAAACACCAGGAGGATCCTTGGATCGAAGTAGGAGGTAGGAGGAAGCCTTGGAGACAGACCAAAACTGAACAAGGAGCTGATTCATGTAGACAGAGACAAACTTACGTATACCAGACAAGGGGAAAACACAGACCCCAATCATGGAGGAGTAAAAGGACATAACCTCCTTTTATTTCTCCCATTTCCCAGATTCCGTTAGTGAGTCATTTCTCTGGAAGTTATTCCAGGAATGGGGAAATGTATGGGAAGTTTTTGTACCTAGGACTAAGAACAAACAAGGCCACAGATACGGCTTTGTTCGTTTTAAAGGTGTAGAGGAGGAAGGTAGATTGGAGAGACAACTTGACAATAACATATTCATAGAAGGTATGAAGTTGTTTGTTAATAAACCAAAGTTCTAGAGAGGAGGAAAAAGAGACATCCAGAACCTGGTGGGAGAAGGTAAAGGAGATAGGAAGCAACTCACGATCCCCATGGAACCCAACCAACGGAGACCCAACAGTCTGCCATCCACCAACATGAAATCATATGCGGAAGCTGTTAAGCGCAATCCGGCGATGAAAGAGAAGGAGTCTATGATGCCTTTGTCGGGGAAGTATAGCTCAGAACCTCCTGTTACATCTGTAACTTTTCATACTTCAACGGAGGACAACAAATGGTTGGACCAAGCTTGGGTGGGTCGTATAAAAAACAGAGGTATGTTTGAGAGGGTGGTGGAAGAGGTTCAGGAGGTGGTAGGAGAGGAGGTGAAGGTATCCTACTGGGGGGACGACATGATTATCCTTTTTGACATGGATGAAGCCAAAGCAGGAAAGATCAATCTCAGAGAACAATCTAATGGAGACACACCCTTATACTCAATCCAGAAATGGACACCGGAGATGAAGACCGAATTCCGTTTAATATGGATTCACATTTGGGGTGTTCCCTTGGAGATATGGGGTGCGGAACATTTTGCGACCTTGCTATCGACATATGGAGAACTTATCGAGCTCGACGAGGAGACGGAGGATCGCTCGAGACTCGACGTCGCGCGGGTTCTGTTACGTACGGAGGTGAAGCCATTTTTCTTCAAATCCATGGTGGTTACGGTGAATGGTACAAAGCATCAGTTGGTTTTAAGGGAGGACATGCCTTGTTTTAAAGGAAAACGGCAATGGCGACCGGAATCGGAGATTTTTCCACCATAGCCGTTCACAACAGCGGTGGAGGATTCGGATGACGGCATGACAGTTTTCACTCCCGACGGAGCTTCGTCAAACTGCCTCAGGGACAGACGCCGCCGACGGTGGACCAGGGCCATCAACCTGTGGCGCACGGAGTCCGAAGCCCATTGTGACGACGACGCGTCATTGCATCGTTCGGAGGCGCCATCTGCTGTGGGCCTATCGCCTCTCCCCGCGCGACACGTGGACTACAACGGTCCCCTCCATAGCCACTTGCATGGACATGGTATTCTAGAACGTAGGGATTCCTCCCTTGGCCAACAGCAGATGCTTCTTAAGGAAAAAGCAGGTGAAGGTCCCAATGAAGTTGACTCTGGTAAAGGGGCTGACCTTTGGGTCGTACAGCATGATCCAGAGGATATTAACCTAGTGTCATACCCCCTGGTCAAACCAAATTCAAATCTCTCATACAAAGAGACTTTTGAAGTAAACAACACCGTTCAGAAAAAGGCATGTAATGCGGAGTTACAGGATTTGGGCCTCATTACTAATGGCCCAACATCAAAGGGAGAGGAATCTGTCCTCAGCTTAAAGGTCTACTCTAGAAGAAAGGGGGGTGTAGGAAGAAATTTCTTGGCCCACTTAAACAGCAACTCTGCTGCAAAGGCCAAAAGGGACGTATATGACACCTTTTCCCTGGATCTTACAGAAAATGACACGCTCAATAAAAAAGATGACATGGCAGGGGATGTTCAGGTAGCGGAGGTGGCTTCATTTCAGCACCAGCAGTGTCTCAGCCCTATTCTTTCCAGTAGTTTCAACAAAGCTCCTCTAGAAAACGAGGAAGACTTCCATCTTCAATTAGCCAGAGATTTGGGCCTTTCTTTTGAAGAGCATAATCACAGTTCTGTAAACATGAAGACTGCTAAGGTTGCTAGTGAACATACAGCAGAGGTAACAGCTGTGATGGGAAAGGATAAGATTGACTCATGAATATTCTATCCTTTAATTCTAGAGGGCTGGGTAGTGGTATCAAGTGGTCAGCTATCAGAAAACTGACCTTGACCAACAATCTTGATATTCTATGTATCCAAGAAACAAAAATGGAAGCCATTGATAGGAGGACTTGTCAGTATTTATGGGCTGATTGTAATGTTGCTTCGGAATGCGCCCCTTCAATTAATGCTGGTGGTGGCCTCCTCTGCATCTGGAATAATGATTCATTTTCAGTTGATAGAAAGGTAGTGGGGGCAGGATTTATTTTGTTGGAAGGAATGTGGATCAAAGACAATAAGAGGGTCTCCATTATCAATGTGTATGCTCCGTGTGACATCCATAGGAAGAGACAGCAATGGGATGAAATTCTGCAGCTGAAGACATCCTCTCAAGAAGGTCTATGGTGTGTAGTAGGTGATTTCAACTCTATAAGACACCAAGATGAGAGAGTGAGTGCAGCCCAGCTTGTGGGTCCTGACCCCAGCATTTCTGAATTCAATTCTTGGATTTCAGAGATGGCATTAGAGGAGGTTAGGTCCATTGGAAGGAAATTCACTTGGTTCAGACCCAATGGCAGTGCCATGGGCAGATTGGACAGGTTCCTCTTATCTGATGAGTGGTTTCTGCAATGGCCAGATTCAACTCAGTTTGTGCTTGATAGGGATTTTTCTGATCACTGCCCTATTCTCTTGAAGTCAAAGAACATTGATTGGGGGCCAAAACCTTTTAAGGTTATGGATTGGTGGTTGAAGGATAAGGGGTTCCAGCAGCTAGTGGAGCAGAAATGGGGCAATTACCACCCTCCTGGTTGGGGAGGCTTTGTCCTGAATCACAAAATAAAGCACCTTAAGCAGAGTATTAAGAGCTGGAGTTTGACAAACAGGGAAGCTAATGCTAGAACAGTCCAGAATATTAAAAAGGAGCTGAATGATTTGGAGACTGGCCTAATTGATAGAGCTCCATCTCAGGAGGAATTGATTCTTAAGAAATCCCTTCAAGGTCAATTGTGGGATGCAGCTTATGCTTACGAATCTATGCTAAGACAAAAGTCTAGAGTAAAGTGGTTAAAAGAAGGGGACAACAACTCTACTTATTTTCACAGATTGATCAACCATAGAAGGAGAAAAAATGCCATCCAAGGTATCTTCATAGATGGTGTGTGGGTTCACGAACCCTGCACTGTTAAAAATGCAGCTATCCTTTATTTCAAGGACAGATTTTCAGAGGAAGCTCCTAGAAGACCAACCCTTGATGGTGTCCAGTTCTCTACTCTTGATTCAAGAGATAAAGAAAGCCTTGTGACTAGATTTTCTGAAGTGGAGATCAAATCAGCAGTTTGGGACTGTGGTGGGGACAAAAGCCCTGGTCCGGATGGGCTAAATTTCAACTTCATTAAGCATTTTTGGGAGATCTTGAAACCTGACTTCATCAGGTTTATGGATGAATTCTACATCAATGGTTCCTTCCCCAAAGGAACTAATGCTTCATTCATAGCCCTCATCCCAAAGATTAATGATCCTCAATCTTTTAATGATTATAGACCCATCTCCCTCATAGGGTGCGTCTATAAAATCGTGGCTAAAGTTCTGGCCAAGAGGCTGGCCCTTGTTCTACCTCACCTTATAGATGATAGACAGACAGCTTTCATGAGAGGAAGACACATTTTGCATGGTGTCTTGATTGCTAATGAGGTTATAGCTGAGGCTAAGGCTAGAAATAAACCTTGCTTGGTTTTCAAAGCGGATTTTGAAAAGGCGTATGACTCGGTTTCATGGGGCTTTCTCGACTACATGCTCATGAGGATGGGCTTTTGTGATAGGTGGAGGAAATGGATTAATGGTTGTCTGTCTACAGCAACCATATCCATTTTAATTAATGGAAGTCCTTCTAAGGAATTTGCTCCTAAGAGAGGTCTAAGGCAAGGTGATCCCCTTGCACCCCTTCTCTTCAATATTGTAGCGGAAGGCCTCACAGGTTTGATGAGATCAGCTGTGTCCAAGAATCTATTTAGCAGCTATAGAGTTGGAATTTTAAAAGAAGAAGTGAACATCCTCCAATATGCTGATGATACCCTGTTTTTTGGAGATCCCACTCAGCAAAATGTTAGATCTTTAAAATGCATTCTGAGATGCTTTGAAAACGTTTATGGCCTCAAGATTAATTATTCTAAAAGCCATCTTGGCTGTTTGGGCAAATCTGGAAGTTGGTGCAGGGCTGCAGCTCAATTCCTTAACTGCAGTCACATGGATTTTCCTTTTTCTTACCTCGGAATGCCTCTAGGGGTGTCCTCGAAGAGCTGGAGTGTGTGGCAGCCTATTATAAGGAAGTTTGAAGATAAACTTGCAAAGTGGAAGCAAAGAAGCCTATCTATGGGGGGCAGAATCACTCTCATTAACTCAGTTTTAGCTGCCTTACCTATCTATCTTCTTTCTTTTTTCAAGATTCCCAAAAAAGTGGTGCACAAGATTGTCTCCATCCAGAGAAAATTTCTGTGGGGAGGTCAGCAAGAGGCTAGCAAGATTTCTTGGGTAAAATGGGGTTCAGTTTGCCTCCCCAAGAACAAAGGGGGCCTTGGGATCAAAGATTTATCCATCTTTAATGAGGCTTTATTTGGCAAATGGGGGTGGGAGCTGGCCAATAACCAGAACCAGCCCTGGGCTAGAATTTTAATCTCCAAATATGGTGGGTGGAAAGAGATGATCTCTGGTGGTAAGAGTAAATTCCACTCTCAATGGTGGCAAGACTTAAAGGCTATCTTCCAGCAGCAGCACAACAACTGTTTTGTTGATAACCTCAAGTGGAGAGTGGGTTGTGGCACCAAAATCAGTTTCTGGAAGGACAAATGGCTGGGGGATAATTATAATCTTCAAACAAAATATCCTACTCTTTTTTTAATAAGCAATCAGTAGACCTCTTCAATCAATTCTATGGGGAATTTGGTGGAAGAGAGATGGGAATGGAAGCTAACATGGAGAAGGAACTTTTTTGACTATGAAATTGACATGGTAGCTGACTTCCTAGCTGACATTGAGTCAGGCAACATCAATCATTCCAGCAGGGATTTCCTTTGCTGGAAGCCTGATCCTAATGACCTATATTCCACAAAGTCTGCTTATAAAATGCTGCAGGAGGCTCATGATAATGCTAATGAGGACAGGGTTCTTAAGATCATGTGGAGCCTGAAAATTCCCCCAAGAGCTAGTGCTTTTTCATGGAGATTATTCAAGAACAGGCTCCCTACTAGGGATAATCTCAGAAGGAGGCAAGTGACTCTGCATTCTTATAGCTGTCCTTTATGTGACCTTGAAGAAGAATCTGTCAATCATCTTTTTTTCAACTGTCCCAAGACTAGGAGTCTCTGGTGGGAGCCTATGAGATGGGTTAATAGAGTGGGTCCTCTCTCCACTGACCCAAACAATCATTTTCTGCAATTCTCTCAATGGAATAGACCAAGCAGCACAGTCAAGAGATGGGAATTTCTCTGGATAGCTCTGTCATTGTCCATTTGGCACCACAGAAATGGCATGATTTTCAATAATCAGCCTTTCGAGGGCTTCTAATAGAAAGTGGAAGATTTGGTGGATAGCAGCAACAGTATCTATCTGGAACGCTAGAAATGATATGATATTTAAAAATCAACCGTTTGTTATCTCTAAGTTGGTGGATAATGCAATCTTTCTCACCTGGTCTTGGTTGAGGGGGTGGGAGAAGGACTTTGCTGTTCCTTTTCAGCAATGGTCCTCATCCATGTCTCTTGCCTTTATCTAGTGTGGGATGGTTGACTGGGTTCTGTAGTTTTACTTGTTGGGGTGGTACTTTGTTGTCTATGTTTTCTAGTCTTAGGAAATGGCATCCTATAGGTATCTTTGTAGTACCTCTGGTACTATTAATATCTAATATATAAATACTTTTGGCCGTTCAAAAAAAAAACATAATAAATGCAGAAAGGAAAGTAAATAAAAAGGGATCAGATCAAATCAGAGAGATTTGATTCTGACCCAAAATAATAATCAAATCAGATCAAATCGGAATAATCTGATCTTGATCCTAATTATTCAATTTTCTAGAAAATTAATTCCAAAAATAAACATCCAGCTGTCTAATTAATTCTTAATATAGACATACAGCTGTCAACACTTTTTTATATGTTTCTGCATTGCAATTTTATTTCTATGTATATGTTATTTTTATTCTGTCCACCACATGTTGCAATGCCATCTCAGATTTTGGGTTGACCACCATAATCTCCCATTAATAACTCTGAGTATGGGTAAGTATAAGTTATAACTATTTATGATTAATGCACCCCAATCTCACTCAACATATTCTGTGGTGGAGTAATTATTTTGAACTGCAAACTGTATCATAACAGGCTTCTTTCGTTGTAGACCTCAAGTTTCCTATAGATTTAGTCATGTTTGCTTTGACCTTGAAATTGCTAAATCCAAAATCCCACCAGAGCTGTTTGACTGTTCAATGCAATTTGTATGCATTGTGTGAGATCTAGCTTGTTTGCTGTTTTCTGACTTGTGACCCTGCTACCTTCAATGCAAATAAAATGATGGAGGATGCAGTGTTTTTATTATGGACTTGGCTGAGACATTTAGAGAAGGGTTTTCAATCTCACTTTAATCACTGGTCTTCAAATATTAGAGAAGGCTTTCGATGTAATCAAGGATAGGAATCTAGACCATATTAGCTACTAGTTTTGTTTTCTTCAGTTTCTATAGAGTGGTTCCTTCTCAGACTATTTTGTCTGATTACGGAACCATTCCCATGGCAACTTCCTTTCTGTATTTCAGTACCTCTGGTACTATATATTTATTTAATCTATTATCTTTGCTGACCAAAAAAAATTGCATATTTCTGATATTTCCAGAAGAGGACAGCCTGCAGTTGAGTTTGTCTTTGCTGCAACTGAGGAGGCAATCTTCATACATGTCATAGTATCCTCAAAGTATGACAGTGCTATTTTTTTATAGTTTGTCAATAGTGTATCTTGCATTGAAATATTTACCTATTATTTTCCCTCCTTTTTATCAGGCATATCCGAATGCTTTCCACAGCTGATCTGGAAAAAGTATTACAACATTCTATGGAGTTTACATATAGACTTCCAGGTTGGCATGCTCTGTGCGCATAAAATTCTGTTGGTTCAAAGTCATTTGTCAGGATTATTCTTTGATATGCTATCTATCTATCTTTTCTTGTGCTAAAATATTTGTATTACGCTGGCTTTTGTTTGGTGTGTAATGATGGAGAGCAATGCTAGGATTTTCAAAGAATCCTTGATAAAGATTCCTGTCCTTTGGGATAGCGTTTTGAATTTGCATATCTTTGCTAATGTGAACACGGTCATTTTTGGCCTATTATATAATTTACGAGGGTTTGGGGAGTTTCGTTGCACTGAGAACTGAATTTTGCTTAGAGATTTTGTTACAGTTAATTGCTGATATTTGTTCTTTCTCTATCTATGTGACATGTCCATGTCATGTTATTGTCACTATCTTTGTCAATGTTAATCCCTGTTTTAGGTGTCATAATGTCATAATGTTAAGACGGGGGGCGGGGTGGTTAATTAGGATTGTTAGAATGTTAGTTAGAATGAGGGAAGGAGTTAGAGGAAGTTTGTGACAGAAATTAATAGAACTAAAGGGAGGGGAATTCATTCAGATCAGTTATCATTTATAATTTTAGCAATTGCTCTTTGATAAGGAGAAATCCTTTGTAAAGAGGAATCTCCTTTGTTCTGTTCAATTCAATCTTTTTCTTTGCTCTATTTTCTAAGCTGGGTTTCTAACAAATATCATATGTTCATGCATGTTCATTACTAATCAGTAGTTAGCAATATCCAAAAGGGTTACTAAGTTCATCTTAGTCCTCCAGGTGGCTTTTCTTGGCACCGTTTGATTTTATTTTACATTTTTTTGCGTCAGTGATTGTTTCTCCACATGGAATTTGTGGCAGCTTGACTGGTTGTTCTCCTAGTGATCTTGTCAAGCAAAGCTACTTCAGGTCCCTTTTATAACCTTTTATGTAGACATTTGATATATCAAGATATCACCCATGACGAAATTCCTTTTTTTTTTCTCTTTTCTATTTTTAATAGCAGTTCTACCAAATTTAGGGTGTCCAATGGAATTATAGGTCTCCCATATCATGTTTCCCAGGGTGTTGGTTGCCAGCTGAGGGGGCAGAATTGTTATGTTGAAGTAAGTCTTGGCTTCCCTAGATCTGGAACTGACAACACGTTGCAGCCAAACAAAAATAGTGTTAGGAATTTACCCAAACTTCATGTTGCCGAATCTCCTATTGTGGGACGAAGTGATCACAAGGGATCACCAGATCATTTGTTGGATTATGATAAAACATTTCTCTATCCAGCTGAGGCAGTGCTTGTGCCAGTTTTACAAACATCATTAGCAAGGTCTTCCTTGAGAAGGTATTTTTTTTCCATGGACATATGCTATGCTATTTTCTTCCTTTCTTTGGTTAAGAGCTTTGTGTATAACTGTTAAGACTCACTAGTCTAGCTGGTTTTCCTGCTTGATTTTTGTTGATATATACTTTTTATTTTAAATAATAAAAGTTCATTTAGACAGAAGATAGTTACAAGGAAGTTTAAAAATAAGGGATTATCCATATGAAAATAAAAGGAAAATTAGAACAAGAAAAAAAAAATGTCACTGGGGAAAAATTGCCCAATCCCTCTGCATATTTATTTGGAAAACCTCCTTGAAAAGACCACATGCTTGATTCCCAATAGAAGCTAATCTCTGAATCATATCCAAATAAGCTACATAGAAAACAAATCTCATCAACATTACTGGTTTATGCACTATTGGGAAAAACCCAAATCCCACATCGGTTAGATATAGTGCCGAGATATAGTATATAAATGGGGGGCAACCCTCACCCTATGAGCTGGCTTTTGGAGTTAAGTTAGGTCCAAACCCACATTCAAAGATGGTATCAGAGCCTATCCTAGATTCATTAACGGGTCACCCACCATATTATCCACGTACCAAGCCCAAAAGTGCTTGAGCGTGAGGGGGTATATTGGGAAAAACCCAAATCCCACATCGGGTAGAGATAGTGGCAAGATATAGTATATAAGTGGGGGGAAACCCTTACCCTATGAGCTAGCTTTAGGGGTTGGGTTAAGCCCAAACCCACTTTATAAGATGCACCAACAATATGCAGCAAATAGTGGCTTGAATTGTGCAAGAGTAGTTAATGATGGGTAGAGCAAATTAGAGTACAGCGTCCCTGCCTCCCAAATGCTCATGTTGGTTTGATTCACCATCATGGTGTTTCGCAATAGTGTCCTGTTGCCACTTGAATAGCAGCAGTCATACTGTCATAGTGAGCATATGTTCTGCATGACCCCTAGATAAAAGAACTTCTGAAATATATGTTTTTAATATGATTGATTGTATAGCTGAAATTAGGGATTTCCTTCTTAGTCATTATTAGGTTTCTGTAGTTATGTATCAATTCTGCTTATAAATAAAGGTATTGTGTGTGGTGAAGAACATACAACACAGTAAATTCCTTTCTTACATGGTATCAGAGCCTGGTTCGATCCTGGGACCAGTGGGTTATGGGTCCACCATGCTTCCGCTGTGTCTGAACTCTAATAGCTGGCCTTTAATCCACAAGCTACCGTGCCTTGTCTTCTCTGTTTTCAATGGCCTCCTCTGTTTTGTGTGCGCTACACCCATCTTTACTTGTTTTCATTTTACTCTTATTTATTTTTGGACACAATTTCTTACACTATTAATTGGTTTCACCTAAATTACAATTCTTGGTGTCACTTTAGCTGTAACAAATTTATTTTTGTCAGATGAATATGCTGTATGTGCATTGTGATTGTCCTGTCATATGAACCATTATACTTCAATTCCCAGAAGAGCCCTGTGATTCATATACTCTATTAATGTGAAACATACAGATTTTGGCTGCAGAATTGGATGGGACCATCCCTGCCTGGTTCCTCTTCCTTTATTCACTGGTTTGTGCTCTCTTTATCGCTCTCTGCATTCATGCATGTTTTTGTCTGAGGTGTTTCTTGATTCTTACATCTTGATTATTATTAAGATTAATTGTGAACCCCTAGCTGTACTTTAGCACCTCTGGTACTCAATTTATCAATACATATTATATGTTTGCCGATCCAAAAAAAAATTGTGAACCCTGTACTACTTCTGTCTTCTGCTGATGGCTGTCTGGCATACAGAGACTATGCAGTCTGTTGCATTTTTTTCCCATGTTTCTTGAGTTCTTGTTGTGTCTAGAATTTTCGGTATTAGTATCTATCATATTTTCTCCATAGTCTATAGTCGTACTTTTCTTCTTTTCTGGAGAAAAAAAAATTGTTGTCAATAGGTTTTTTGATAAACCTTGTCTCACTTCTATTCCTCTTTCAAAACCTTATCCCTGTAATTTATTGGTTAATGTTTTGAGCACATAAATTTGTTTGAGTACATTTGTTCTGGTTTTAGCTAAAGCCTCAATTTTCTTCGGCTTTCTGCAGTGCTGGTAATGTGGATTGTACTGAAGATCCTTGGACCGAAATCAATGGAACCCGCACACAAAATAGTTATGATAGTAGTAGCAATAGCAATAGTAGCAGTATTAGTAGCTTAAGTGCCAGCTCTAGTGATAGTGATTACAAGACTACAAGACCAAGTGAACTAGAGGCAGATGCTGATTCTTTGACGTGTAGACAGTCTATGGTATCTTCTGCTGATCAGTTGGACAGTGATGGCCCCAAATTGGTATATCATTATTTACTTTTGAAAAATAATATTGAACTTTTTTTACTTTAAAAGAAAGTTTACCATGATGAATAAAAGAAAATTACAAGGAACTTGGAGGATAATCAATCATCCAAAAGGAAAATCTAGGCAACAAAGATGAGAAAACCAATGGAACAAAGCTGCCAATCACTCTGCACATCTGCTAGGGACCTTTTAAAAAACCACGTGCTGTACACAAAATAAGAGTCAATTGCTGAATCTTATCTTATATCATTAGACAAGCCTATCGACCTCCTTAAAATATGAACACTATCCTTCAACCAAATGAATCAAATAATAGCATAAACTGCACATCGCCATAAAATTTTTAGTCTTTTTACTTCTACCCAAACTACTAAAGCTAACTACCATAAATTTCCCACCTTTTTATATTCTTTGGGATAAAGGCTTATAATATTTTATTGGATTATGAAAAAGCAAGGTGGTGGTGTAGCATTTTGCTAAATGTAATGTATTGCATGCATAATGCACCAATTCTGGTCAGAGTAGTTACCATACCCATAGGTACCTAATGAGTACATGATACACACACACACACACACACTCCCACAGTTTAAATTTGAATGTAAAACTGAGACCATCGATTCTGGATACGGGTACTTGGCCAGTTTAGCTTTAGCTGTACCCTTACAACATAGTGATACACATACTTCTCACTTCATAACCTAGCCACGTTCTGGATTGTATGCACATGAAGAAGGCTAACTTACTGCAATTTAAAAGTAAATTGAGAAAAAATGAAAGATTCTTTAACAATATGTTATGCAGTATTTTTCTTGAAAATATGAAAGATTCTTTAACAATATTCTGTGTGCTCTTATTGTATTGTATTTTTTTTGCTGGAAGGAGTGTTTTTAATTTTTGTGTTTGCCGATCTAGTTTTTGTGCAATTGAGTATTATAGCTGCTCTTATTTCTCTGCAGGGCTCTAAGCGGTCTCGAACAGGGGTGACAGAGTCATTAAGTACAGCTACAAACATTCCTGTGCAAGATGCTTACATGTCTGATTTTGGTTCCATGGAAGTGAACAATTCAGCCATTACAGGAGTAGGAAATGATCCAATTGGGTCTTACTGGGACTGGGATGATGATGATAGAGGCATGGAAATGGATATTCAAGCCCTTCTATCAGAGTTTGGTGATTTTGGTGACTTCTTTGAAAATGATGTTTTGCCTTTTGGAGAGGTATCTGAATATTTATACTACAATTTAAAGAGGATTTCTTCTTTTGATTTCCACTTTTTTCTCCTCTATAATCCTGAGTTTTAGTTAGAGCCATTGAGTTGTGACTTCCATTTCTAAACAGGTGCACAACACAATTAAACTCTTAAATTGTAAATTAGACATCCAAAGAGTTATGTGTTACTCTAAAATTATGCATTATGTTCGTTCTAGCAGTTTATAAATTGGAACAGTATAAGTTTGGTTTTCCTATGGATAAAAATCCATATCCTAACTATCTAATGTAACTAACTAATGGGTAGGTCGAGAATGATTTGAGAATGAGAGAGAGAAACTTCCATCCGAGATCGTGGGAGAGACAGTGGGAGAGAGAGAGATGGTAGACTGAGACATAGAGTGTGGGAGAGAGACAGGGGGAGAGAGAGAGAGGGTAGACCGAGAGAGAGAGTGAACACAAAGAGAGCAACAAACGGCTACAGGAACGACTTCCACCAACGGGGATTTCGGAGCAACACAAGGAGGTTCTGGAGGATTGATGACGCTTACTTGCGAACTCAACGAGACTCCCCCTCACGGACCGCATACGAAGAACAGGAGCAACAGAAACTTGATGTTAACAATCATGGAGCCTGGGTGGAAGTAACCCGCAAGAAGAAAAGGAACGAGAAGGATAATAGAGGCAGACCGGATGGAGGCAGTCATCAACGGGAAGAAAAGAAAGTGACATGGAGAGACAAGGCTGATGTTACGACATATTACTTCTCTAGATTCCCTGAAGGGATGATGGAGAAAGACATGTGGAGGATCTTCCAGAAGTGGGGAAAGGTGTGGGAAGTCTTTATTTCCAAAACCAAAAACAAGTTGGGCCATAGGTTTGGTTTTGTCCGATTTAAAGAAGTCATGGATGAGAAGAGATTAGAGAGGCAGCTAGATACTAATATCTTCATTGGTGGGGTGAAAATGTTTGTCAATAGTCCGAAAGTCGACAGGGGGAAGATGATAGGCAATCGTGAAAGAAGGATCTTGAGTCACGGTGAGGGAGCTACTCAACAGAAGCACAAAGCTGGGTATGTGGACGAAGGTGTAAAGGAGGAAAATAAAGTCAATACTAAAACTCGATCATATGCTGTGGTGGTGAAGGAGTCTGTATATGGTGAAACGAATCATATGAACCCGCTGGTAACTATGCCCGGGGCTGAAAAGGTGAACCATGGCCCAGTCACTCTGCATTCAAGTAAGACGAAACTTGAGTGGCTCTCTAAGGCATGGGTAGCTAGACTGAAAAACAGAGGCATGTTTGAGAGGCTGGACGAAGAATTGAAATGGGTGGCTGAAGATGATATCAATCCATGCTACTGGGTTGATGATTGGGTCATCTTCCCTGATATGGATGAGTCCAAAGTTGTTCGACTCATACACCAAGAGAACGAGAACGGGTCTACCCCCATATCGGAACTCCAGAAATGGTCCCCGGAGATACGGCCCACACACAGGCTCACCTGGGTTCTCCTTTGGGGTCTCCCTCTGTCAGTTTGGGAAGAGGAGTGTATGGCGAAGGTGTTGGCTGAAGCTGGGGAGGTGATTGAAGTAGATGAGAACGTGGAGGTCAGACGGCGTTTGGATGTGGCGCGTATCCTGGTTTGGATGCAGCTTAAACCTTCGTTTCAGGTGACGATTCCGGCCACCGTTGACGGAGTTGAGTGCGTCCTCCAAGTTGTTGAGGACATGACGAACCTGGGCGGGTCGAAGCAGATGCCATGCAACCCATCGTGGCTCCCCCCTTCCCCTTTCTCGACCCAGCCAAACACCCCCGTAACAGGGGGTGTTCTCCACCCAAAGGTCTTTTCCGGCGACGGGTTTCCCGACGGGGCGTCCGACGACACTTTCGGCGGGGACTCCGAAGATCCCGATGGGTTCCTTCCGCATAATCCTCGCAGAGACCAGTGGGTAAAGACCCTGGCTCGGAGTTGCAGGGACTGGTCCGTTAATCCTGATGACGTGGAAGCATTTCATAGGGACACTGTTCAAAATTCACAGGACATAGGGACAGAGATGGCGTTGACAGCCTCCAACGGCCATCTTCCCCGAAATGGGTCAGCATTAAATGGAGATGAAGAAAGTGATAAGCAGGATATCATGTTTAAGGAAAAGTTTTCTGTTATAGACAGCCTTGTAGACCAGGCCCATTTGACTTACGGGCTTGAGGAAATTCAATATAGGTCAGAAAAAGGAGTTGCAGGGGATAAAGTGGATGTTATGCCCCGTGAGGAAATTACACCTCACGTTTCAAATGTCAGGGACAAGGATAATGGTACAGCTAGTCAACTTTTTTCAGAGAAGGTAGCTGAGGAAATGGGCCTTCCAAATGGCCCAACTTCTTCAGTCAATAAGGGGTATGTGAGAAGAAAAACAGTGTTGAAAAGTAAAACCAAGGCCCAGTGGGGGGAGGACTCAATCGTGGACTGCATTTCAGACCATAATGCTCTTCCTTTGCCTCTTTTACAAGAGAAGACAAATTCAATACCAGGCCCTAAAGATGATCCTTCGAATTCACAATCAGAGGACCCTATACATATGCAATATGCCCTTCTAAAGGAAATGGGTCTATCATGTGGGGAAGATGACAGCAAGGTTAAGGGGATGTTATTAGACATGGAGAACAAGGATGTTATGTTGACAGCAGAGAGGGGAGCTAAAAATCACAATTATGATAATTCTGTCTTATAATTCCAGAGGTTTAGGGAGGGGGGTTAAGTGGACTGCTATAAGAAGGCTGATTTCAAAACACAAGGTGGACCTTGTGTGCATTCAAGAGACTAAAAGGGAGAATTTTAACAAGATTATTTGTAATGCCATTTGGGGGGATTCCACTGCTCAGTGGGATTATGTTCCTTCTGTGCAAGCTGCTGGTGGGCTGCTTTGTATGTGGAATAACTCCATTTTTGAGGTTGAAAGGAGGGTGAAAGGAAGAAGTTTTTTAATGCTTGAAGGGAGATGCACTTACAATAACCAGAGGTTGATAATTGTCAATGTTTATGCTCCATGTGATTTTGCTGAGAAAAAAGCTTTATGGGATGATATATGGCAGCTAAAAGTCTCTAACTCAAGGGGTTTATGGTGTGTTCTTGGGGACTTTAACAGTATTAGAAGTTTAGAGGAAAGGGTTAGTTTATCTCAAAGAAGGGTAGAATCACAAGACATCTCAGCTTTTAATCAATGGATCTCTGATATGGAGCTCCAGGAAATTAAATCTGTGGGTTCTAACTATACTTGGATTAGGCCTAATGGTTATGTAAAGAGTAGGCTTGACAGATTCTTGGTCTCGGATCAATGGTTATCTGTGTGGCCTGAGAGCTGCCAACATGTTTTGCAAAGGGATTTATCGGACCATTGTCCGACCATTCTGCAAACTAATATGGTGGATTGGGGTCCTAAGCCCTTTAGGGTTTTTGACTGGTGGTTGCAGCAGAATCAATATCAGAAAATGGTGAGGGATACATGGTGTAATGATCAGCAGGGAGGTTGGGGGAGTATTGCTTTAAAAAATAAGCTGAAAAACTTGAAAGCTGCTATAAAACAGTGGAGTAAGGATGAGGGGAAAATTGATGCTAACAAAATATCCAGCATCCAGCAGAAACTAAATGAGATGGAGGACTTAGCTTCTAATCGTATTTTATCCGATCAAGAGCTCAAAATCAGAAACTCCCTTCAGCAGGATTTATGGAATGCTTCAATTGCTGTGGAATCACTTTTGAGACAAAAGTCTAGGATATCATGGTTGAAGGAGGGGGACTATAATTCTGGGTATTTCCATAGAATAATAAATCACAGGAGAGCATATAATGCTATTCCAGGTATTACTATTGATGGTGTGTGGGTGCAGCAACCAATCTTAGTGAAGAATGCAGCTGTTAATTATTTCCAGGCCAGGTTTACTGAACATGATTATTCTAGGCCTTATTTGGATGGGGTCCCCTTTAAATCTATTTCTCAAAGACAAAGAGAGCAGTTGATTGTCCCATTTTCTGATCTAGAGATCAAGGAGGCTGTGTGGAGTTGTGGAGGTGATAAATGCCCTGGGCCAGATGGCTTTAACTTCAACTTTATAAAAAAATTTTGGGATATAATGAGTCCAGAATTTAGAAGATTTGTGGATGAGTTCTATGCTCATGGCAGCTTTCCTAGAGGAAGCAATGCTTCCTTTGTGGCTTTAATTCCTAAGTTGTCTCATCCTCAGTCTTTCAATGACTATAGGCCTATATCCTTGATAGGTTGTATGTATAAAGTTATAGCCAAGTTGCTGTCTAACAGACTGAGGTCTGTAATGAATGGGTTAATTGATGAAAGGCAGTCAGCCTTTATTAAAGGAAGGCATATTCTTCATGGTATTGTGATTCTCAATGAGGTGGTTGAGGAAGCCAGGAGGAAGAGGAAACCTGTGATGATCTTCAAGGTGGATTTTGAAAAGGCCTACGACTCAGTTTCTTGGTCTTTTTTGGACTACATGCTGTTCAGGTTGGGATTCTGCTCAAAATGGAGATCATGGATATCAGCTTGTCTCCACTCAGCATCTATATCTATTCTGATTAATGGCAGCCCTTCTAAGGAATTCTCCCCTACAAGGGGTTTGAGACAAGGGGATCCCCTAGCTCCCTTACTTTTCAATATTGTAGGTGAAGGTATTACTGGTATGATGAGACAAGCAGTCCACAAAAACCTTTATAGAAGCTTCTTGGTTGGACAGAAAAAGGAGCCTATCAACATTTTGCAGTATGCTGATGACACTGTCTTTGTAGGAGAGGCTGTGTGGGAGAATATTCTTGTTATAAAGGCCTTATTAAGAGGCTATGAACTAGCTTCTGGTTTAAAGATTAACTTTGCTAAAAGTCAGTTTGGGATAATAGGGGGTGGAGTCAATTGGTCTTTGGAAGCAGCCAATTATTTGCACTGTCGACAGCTGGATTATCCTTTCCTTTATTTAGGCTTACCTATTGGGGCTAATCCTACTAGCCAGCTGGTGTGGGAGCCTCTTATTTCTAGATTCAAGTCTAAATTAGCCAAATGGGCTCAAAGAGATATATTCATGGCTGGGAAGATCACTCTTATCAATTCTGTCCTTAATGCCCTTCCAATTTATCTCCTTTCCTTTTTTAAAATTCCTCAGAAGGTAGTCAAAAAGCCGGTATCCCTCCAAAGAAATTTTTTGTGGGGTGGGGACAATGATCACAAAAAAATTCCTTGGGTCAAATGGGATGATATCTGTTTGCCTAAGACAAAGGGCGGCCTGGGGATAAAGGATATCTCTAAATTCAATTTAGCTCTGATGGGTAGATGGATTTGGGCTTTTGCATCTGATCAACAGCAGCTCTGGGTCAGAATTTTAATTTCAAAATATGGTGGTTGGTCAGAATTCCAAAATGCTAGTGACAAAAGGGGCTTTTCCCACTGGTGGAGAGACATTAGAAATCTGTATCACCAGTCGGACTGCAGTATTTTTAAAGACAACTTGGCTTGGAAGGTTGGGAGTGGGGAGAATATTAAATTCTGGACTGATAAATGGCTAGGGGACCAGCACACTCTGCAGCAGAAATATAATCAGCTTTTCCTCATAAGTAGACAGCAAAAGGACCATATTTCTCAAATGGGATATTTTAATAATAATAGCTGGAGGTGGGACATGAGGTGGAGGAGGAATCTCTTTGATCATGAAAGCCAGTTGGCTGTGCAGTTTATGGAGGAAATTAGTTCTGTTCCTATTCAACGGCAGGTGAAAGATAATATGCTATGGTTGGCTGAATCTAATGGTCAATATACTACTAGATCAGCTTATAGCTTATGCTTGAACACCACTTCAGTAGTTTCTGATGGCAATATTTTTAAGACTATATGGCAGCTGAATATTCCCCCTAGGGCTGTGATCTTCTGTTGGAGATTACTTAAAAATAGACTACCTACCAAGGTCAACCTTCTAAGAAGAAATGCTATTACTCAGGAAGACACCTGTTCCTTATGTGGCTGTGTGCAGGAGGATGTTTATCATTTGTTTTTCAACTGTAAGCTGACAAATGGCCTGTGGTGGGAATCTATGCGCTGGGTTCGAGTAGTAGGTCCCCTTTCCATTAACCCGGTTCACCATTTTTATCAGTTTTGTGAGGGGTTTGGGGCAAATAAGAACTACAGTACAAGATGTGGGTGGTGGGTTGCGTTAACTACTTCTATATGGCAGCATAGGAATCTTTTTATTTTCCAAGGAAAACCTTTTGATCCCTGTAAAGTTATGGATCATGCTATTTTCTTAGCTTGGTCATGGTTAAAGGCTAAGGATAAAAAGTTTAGTACTAGCTTCCACTACTGGTCCTCTAATATTCCCAATTTTGTTGCCTAGTTGGGGGTTGGATCTGGCTGTTTTCTGGCTTGTGTTGTTGGTCTTTTTGGAGGGTAGCACCTTGGTGCTTTGTATTTATACTTTCTGGTACCACTGGTACTGGTTGTTTTATTAATAATATATATATTTTCAGCCTTCCAAAAAAAATAAAATGCATACACTACCCTCTACACTTTTTGGTTTGTTTACTATACCATAATATGGGCCTCCTTGAATATAAAGAGGGTTTGAAGATCCTTATTGCACTAATGAAGTTGTTTAGATTGTTCTATCTTTTCTTTGTTTGGGAGTGTAAGGTGTTTTGTAATTTCTTATGCCTTGTCTCTTCTTTCTTATGTGTGTGTGTAAAATAATGAATTTTATTTCATTTATTATTTTATTTCTTGATGATTCTTCAAATTAAAATTTGGATATAAGATATGGTTGACTGTATAGATTGTATAATTTCTATAAATTTACTCAGAATCTTTTTGTTTAACTCATGAATACTTCAAATTTATAAGCTCTTCCTCTTTATTATTCTAATATTGTTTTTTTCTCTCTGCTTCTTGGTCTCTTGTATATTGGTTTCTTATTGTGTGGTCATTTTCTGACAAAGCCCCCAGGAACTGCAGAATCTCAGGCACTTATGCTTTCTGCCCCGGATTGTGGAGATGTCAACAGCAGTCCAGGTGGTGTGATAGATGTTCCAGGTCAAATACTTTTGCCTGTTGGGTTTCCTTCCTTTGAGAGCTTTAACCCCCCTCCTTCAACATCCATAGAGGAATGTCTTAACAAAAGTCAAGATAACTTGAATAACTCCATGTCTTTGTGTCCAACCAATCAAACTCAACTGTTGTATACGAGGGAGTTTGATCATATTATGAAAGCTGAAGCAATGATGACATTTGCTCCTGAATTTGGAGCTGTTGATACCCCTACTTGTGAGTTGTCCACAACATTATTCAGAAGCCCATATTTTCCAAAATCTCGAAAAGCAAAGAGTTCAAATTCAAGTTCAAACAATTATTTATATGGTGCAGCACCACCCACTTCTCCTTGCACTGAAGGATCAGAGGGGAAGAATGGAATGTCTGCCAACACAAAAACAGGTTCTGGAAAATATGATGCTAGTACTACGAGTCTCCACTCGAAATATTACTATACTTTTGTGGAAAGTAGGAAAGAAAAAAATGATAAAAATCCTGCCACATGTAATGATAACAGCATAACTAAATCTGAGGGGATACCACCACTCTCAAACATTGGTTCTAATGCTATTGTCAAATCTGCCATAAGGAAGACAACTGAAGGCACACATGAAGCAGAGCATTTCCTTCTATCTGCAAAAACTTTATTAGCAACTGATATCACATGTGTTACGTTGCAAGCTTCCATGTGCAGGTTACGCCATATTCTCTTATCTTCAGGTAACCTAATGCCTGTTGGTTTGAGTAGGTCAACTGGGGTTTCATTTTTGAATCAGCTTCCTAGTGATCCAAGTATGACAACAGACAACATATCTGGCAAGTATGATGTGAAGAAGAAAGAAAATATACCAATTAGAATTGCAGGTGATATTGATGGTGGAATGCTTGATGGCCACCTTAATGCCCCTGTTGGTGTTTGGCGCACATTAGGAGCTTCAAAAGTTGTAAAACCTTCAAATTCACCTAACATGGAAGTTGTTCCTTCCTTTCCTCATAATTCTTTCAATGAAGAGGGTATCCTTTCTTATGGTCTAAGGAAACCACTTCAGGAGCTTCTTGATGGGATAGCATTACTCGTCCAACAAGCTATTTCCTTTGTTGATCTGGCCTTGGATGCGGATTGTGGTGATGGTCCTTATGGTTTGCTTGCAATGCAAGAACAGTGGAGGCGTGGATTTTGTTGTGGGCCTTCCATGGTCCATGCTGGCTGTGGGGGAAGTCTTGCCTCTTCTCATTCATTGGACATTGCTGGCTTGGAGTTAGTGGATCCACTTTCTGCTGATGTAAGCTTTTGCTGTAATGTTTTTAAATGCTCCTTCTACTGCATGTTGTAAATCTATGAATCATTTGTCTCTTTCCTTATGGGTATGGATTTAGGATTTGGTCTTTGATGATAGAAGTAAAATGAATTTCTACAATTCTAACATAGCATGGATGTTTTGAAGTTATCCTTGTTGAGGTGCGTAGAAATATTCAGGCGAAAGAGGGAAGATACAATTAATTTGGAATCCTTGATTTGGTGTGGATATACCACCTTTCACTCTTGGAAGGATTTGCATGTCATTTAAGGTTATGAAATAGACAATAAATAGATACACTGAATTTACTAATAAATTTTGTGTGTTGAAAACATGTAAATGTAAATTTTGTGTTCTTGCAGAATTATTGCATGGATAATAATAATAATAATAAATAAATAAATAAATAATCCTTGCATACATTGAACTCATCTAAACACAGTAACTAAGCTAGGAAAATAATATATTTTTTTAATCAGCAAAAGTAGTATTGTATTATATATCTAACAGTACAAGTTGTACTGAATGATTACAGAGTGGCCAGTTTAGGTAGGACTATGGTGTCCTAATACATAGTTTACAACATGAATTAAGAAACCATAGTACCGGCGATATGCTAATCTCACCATTACCCCTACTAATATTGTTATCTCCCCTTCATCCCATGAAAGCCTCCTTCATATTGCTGGACCAGTGATAAAAAGGCATGTCAAAACCTTTAACCAGATTTTTAGCTAGGTCCAGCAGAAAAATATGGCATCCTCCAACAGCTTGCTGACATTAAACCTTTCCTCTTCAAAAACAATCTTATTCCTATGCTGCCAGATACTGCATGTCAAGGAAATCCACCAACATTGCCATTTTTGAAAGCTATTGCCATTTCCATTCCCAACCATATGCTGGAGGAAGTGCATACGCGGGTTCTCTAGAAATGGTGTTGAGGTATTTGTCCATGATAATGATTCCCACTAAAGCGGCATGATTTTGCTGCAACTAAAAAAGAGATGTGTTGCATCCTCTTCTTTATTTTTGCAAAATGGGCATGTAGGATCATGTATTTCCACATTCCTCTTGCGCAAATTGATTTTAGTTGGTAATCTATCCCTGATTAGTCTCCACGCAAAAAAGGAAACTTTACTAGGGACCTTGACCTTCCACAGCTCTGTAAAAGCACCATCCTGATTTTCATTAATTTGATCTGACATGAGCAACCTATACTCGTTCCTGGCTGTTATAAGCCTGAAACATCCTCTTTCCAAATCCACTTATCTTGTTGTTCCGGATGAATGCTGGTACCTTCTAGGTCTTCTAGAAACCTCACAGCCATATCCAACTTGTTGTCAAAGTTAGCGTCTCCATTTGAAATCCCACTCCCAACCTTCATCTAAGAAGTTGCCCATTCGCTGAATGACCTGATTTTGCTGCTTCGAAATAGAGAAAAGTGTAGGGTATTTCTGCTTAAGTGATACTCCATCCTCCTTCCATCCATCCTCCCAGAATTTGACCTTTGCTCCGCACCCCACTTTCCATTGTATGCCTTTAGCTAAACATCTTCCATCCTCACCAGCGAAAACTATGTTGTTTATATCCTTCCACCAAACTGATACTCTACTGCCTCTACATACCCCCTCCAAGTTACACCAATCACCATATTTTGAAGTTAGTATTTTAGTCCACAATTCTCCTTGGTTATGAAAAAGGCTCCGTTTCCACTTTTCGAGAAGAGCATATTTAAACTTCCTAAGATCCCTAATTCCAATTCCTCCTTTCTCCTTTGGTATACATACTGACTCCCATTTCATCACGCTATCTTCTTATGCTTTGCCCCTCCACCCCACAGAAACCGCCGCTGCAACCACACCAATTTATCCAGAATGCTTTTTGGAACCCTGAAAAATGAAAAGAAATATATAGGAATAGAGTTTAGGACAGCCTTTATGAGTGTTACCCTTCCCCCAAAAGATAGTAGTTTTTGTTTCCATTTCGCCAATTTCCTCTCACATTTGTTGACAATAGGATCCCACAATTCAGAGCCCCTTGGGTTTGCCCCAATCAGAATACCCAGGTATTGAAAAGGTATGTCCAGTAGCTTGCAGTTTAGGTAGCTGGTAGCAGACGCCTTCCAACTCTCCGACATCCCAATTACCCCAAATCTACTCTTTGAGAAGTTAATTTTGAGGCCTAAGGCTAATTCAAAGCTTCTCAAGATCACCTTGGTGGCTTTCACTTTTTCCATGGATGTTTTCCCAAGGAAAAGTGTGTCATCTGCATACTGCAGTATGCTGACTTTGACTTCGTTTCTGTCCACACTGAAACCTAGAAACAGGTTTTTATCCATTGCTTCTTTCACTAGTCCATTTAGTCCTTCCACAACAATGTTAAACAAGAACGGAGCTAGAGGGTCTCCTTGTCTTAGGCCTCTTTGGGGAATGAACTTAGATGTAGGACTACTGTTAACCAATATGGATATAGAAGCAGACTTTAGACAGCCTTTGATCCAAGATACCCATTTTCCACAAAACCCCATCCTCTTCAACATATACAATAAAAACTCCCAAGATACTGAATCATAAGCTTTTTCGTAATCTACTTTGAAGACCAGACACGACTTGTTCTTTGTCTTTGCTTCATGGATCACTTCATTCGCAGTGATAGTGTTGTGCAGCAGGTGCCTACCCTCTATGAATGCTGATTACGTCTCATCTATTAATAGTGGCATCACCCCCTTCAACCTCCTTGCTAACACCTTGGCCACCGTTTTGTATATGCAACCTATAAAAGAAATAGGCCTGTATTCATGTAGGTTCTGTGGGTCCTTGATCTTAGGGACAAGGGTTATGAAGGATGTGTTACACCCTTTCGAGAAAGATCTATGAGCATAGAACTCGTCCATGAATCGAAGCACGTCAACCTTGATAACTTCCTAGAATTCCTTAATGAATATGAAATTTAGGCCATCTGGGCCAGGAGTCTTATCACTCCCAAACCGTGTCCTTAATTTCTTTTTCAGTGAATGGCCCCGACAATCTGACGTTATGCTGCTGTCCAATTTCTTTAAATCTGACCCGTCTAGTTTGGGTCTCACCCATTCAACCTCCTGAAATCTGTTCTTGAAAAACATGTATGTCTCCTCCTTCACCCTTCCAGGTTCATCAACCCATACATCATTTACAACCACTCCTCTCAAGGTGCTGGATCTGCATTTTGAGTTTGCAAATCTGTGAAAAAACTTGGAATTAAAGTCCCCCTCTTTGATCCATCGTGACCTTGCTTTCTGCCTCAACAAGGACTCGTGAGATTGGGCTGCTGACCACAACTCCTCCTGAAGTTTTTTCCTACACGTTAATTCTTGGGTAGATAGCTGTCTTACATCACCTTCAGCTTATAATTTATTTAGCTCCTTCTCAATAGTTACTAACTTCTGCCGGGGGTCTCCGAAATGATCCTTATTCTATGCTCTCAGGGTAGCCTTAATTCCTTTTATTTTTTCTCTGAGAACAAACCCCCCCCCCCCCCCCCATCCCCTTACCTGATTGTTCGACCAAGATTCTTGAACCACCCTTCGAAAAGATTTATCATGGAACCAGCAATTTAAAATCTGAAAAGGGCGTGGTCCCCAATCAGCAATTTCTGATTTCAGCATAACTGGGCAATGATCCGAGAAATTTCTTTCCAGGATAAACTGTGTCGAGCCTTGCCATTTCTGCAACCAATCAGCAGTGACAAGAAATCTGTCCAGTCTGCTTTTTGATGTTCCGTTTGGCTTAATACCAGGTGAATTTTCTCCCTACACAAGGCACATCTTCAACCCGAATGTCCTCTATCCATTCATTAAAATCATCCATGGTGGTATCATTTACCCTTCGTTGGCTCATCCCTACTCTCTCCTCTGCTGTTCGAATGCTGTTAAAATCACCAAGTATACACCACAACACGCCTGGGTTTAAAGCTTTCAGTTGAGTGATTTGGTCCCACAAATTCCCCTTCTGAATCATGTCACATGGCACATATATGTTGACTATAACCATCCTTTGCCCGTCCGGGACCCATATTCCCTCTAGGTAGATGAATCCTTGCCCACACACCTTCCTGTCCAATCTGAAAGAGCTTTCATTCCACAAGCATAGCAGTCCTCCTGCTCTACTGATGACAGGTTGCATTTTCCATTTGACATTTGTATCTCCCCATAAGGCATGGCAAATATAATTATCTAAGTGTTCCTTCTTAGTTTCTTGTAAACACAGCATGTCTGCTCTCTCCTTTCTAATCATTCTTCTGATTGCAGCCCATTTCACTCCCCTCCCTAGCCCCCTTATGTTGTATGTAATAATCTTCATTATCCAGGCCTCCTGTTACCCAACTTGTTGGCCTCCTCCTTATCCGTCACCTCCATCTTCATAAATTTTGTGACCACTGTTGCATCTCCTTCCTCGCATGTAACTCCTAGGTTTTGTGCCATCTTCCATTGTGCTGAGGCTTCCCTTATTTGAGCACATGTCTGGTGGCTATCTTTTTGCAAAGGAGTCTGAGTTTTAAGCTGTGCTTCGACTGGTGCTGTTGTCTCTGCCACTTCTTCCGACCCACTGCCTCTGGTTCCTGGTGAGAAGTTCTCCTGGTTCTCAATAACACTACTGACTTTGTTTTGGGCGTTATTATCTGTTTTTCTTAACAACCCAGCAGTGTGCTCTTGGTCCAGTTTCTTTCTACACCCTCAACTTCTTGAGTACACCTTTCCCATGTGCCTTGGGCCTATTTGTTCACAATGGGTATTTCTTTCTGCACCCCCTGGTTTGTCACGTAGGTAATCTTTTGAGAGGCCCATTGAGGGCCCACCATCCTCCACATTTTGTAAGACAACGCATGGAGTTGTGGCCTCTAATTCAAAATTTGCTAACATCACTAGGTCCGCCTCATTCCCACTAACCTTGCAATTTTCTGATTCCATTAAAAGTTCCTTTTCCGAAATTACAGCTACTACCTTTCCTACCTCCTTAAGCAATTCCTTCTCCATCTTGTCTCCATTTGTTTTATCCTCTTTCCGTAAATGGCTGCTAGGGGTGCGATTTGACTCATCAGCAGTCCCTTGTGCCTGTAATATTTTACTTTTGTCAGGCCTAAGGTTGCTTCGGTCAGTTTCTCTTTGAACATTCCAGGTTTCTGGTATGGCCAACTCACGTGTTGTCGACAGTGACCTCTGGTTACTGACCTGTGGGTTGACAGGCTGGTATTGTCCTTCGGTCCGTTGTGTCACCGTGACTACTTGATCTTTCTCGCCTCCCGTTGCACTTGGCCGAGCATCCTCCGGTGGCAGTTAAGTATCAGACATCATGTCTCCATTCGTCATTTGTGTTCCTGCTTCATTGTCACTGTCGACGGAGCGTATTTCCTTCGATGATCCTATAAATCTTCTCCCCCAACATTTGCATCTATCCTGGTTATAATAAGTTTCTTCAACCATGTGCACGGTGTACTCTACTCTGTTAATCCATACTGAGACCTTATGTTGGATTAATGGTGGTTGTGGTGTCTTAACTAGTATCCGCCTCCTATCCAGCCTGTGGAGTTCGTCCATGTCATCATCCACCTCCACCACTTCTCTGACCGCTGTTGCAATCTGTTTAATGTGCTCCATGTCCCAGGCATGCAATGGTATACCCCAACATAAGAGCCAGACCAGACGGTGCCCAGTCCTCAATTGAGGGGTCCACTTTTCCAACGAATAGAATAATGCCGAATGTTGTTCATTTACTTCCTCCGTCATTTGTGCAGCTTTGGTCTCTGTAAGGCCCATTAGCAATACCATATCATCTCCGATATATTTCGGTGTGATGTCGTCTTACCCAGTCCACATAAGGCCGTCTTCTATCCTGTCAAACAGTGTGAGGTTCCTCAGTCTTCCCACCCATGTCCTATTTAGCCATGTCTTTTCGTCCGCAGGTATGTTCATCTGCACCGATGAGTGAGATACAGGTCCGGCTGAACTCAAAACTGTTTGATTCCTCCCTGGGGCTAGTTTCTTGTTGTGGTGGTTTTGACTTTTCCTGTTGTCGTTCTCTACTGTCTTCTCCGCGTACCATCCTCTCTCTGCCATGCATCTTTAATCCTCTTACTACCAGGTTGTCCAGCTTTCTCTCCAACGTCGTGACATCCTTCACCCCCTTAAATCTAACAAAGCCGTATCTCTTTCCCACTTATTACGATTTCTTGCGATGAATACCTCTCGAACGTCCCCCCATTTTTTAAACATGTACCACAACTCGGTTTCATTCATATCTTCAGGAAATCTTGTGAAATAGAATGAGTTTAGTCAGATGCATCCCTCCAGTTTCTCCTAATGATGCGCTGCTGGCCATACCCATCTCTGTTGGGGTACCGTTGGGATCCTACAGTCACCCACTCTCTGTTTCTCTCACCCCCCTCCCCCCACTCTCTCTCTCTGTTTCTCGCTCTACCTGTCTCTCATCTCAGTTTATGAATATAACATCAAAAAGAAAGTGCTAAGGTGAAATATAAGCTAGGAAAATAATAATCATTAAATGCTCCTATTTCCAACAAGATAAGATGCTGATCCCCTTGTGTAAGATATCACCTTCGATTTTCTGTTCTATGCTTATATTGACTCCATGTAATATTCTCAATATTCTGATTTATTGAGTTGTTTAGTCATTAATCAGCTCGTCTGTCTATGTCATAAGGATTTGACGATATTTTACAATTTTTGTTGACTGCCTAACACCAGCCTCTTAGATTGACTATGTTAATTGACAACTGGTGAGAGGCTTTGTTGTAGTTTATCAAATTAGAAATCTGTCTAATGATTTGTGATGGGATGTTATGAATTCTTTGTCACAGTCCCCTCCCACTTACTAGTTACTACTTACTATTATTGGTTATCTCATGGGGTTGCTTATTTTAGGGCTTTACGATATTAGATAATAGTCAATGGTATTGTTGCTTGGTTTCTGTTCCATTTTTTATACTTATATTCCAGTATTTTGAATTCTCAGGTTGATGCATCCACTGTCATTAGTTTGCTGCAGTCTGACATAAAAACGGCTTTGAAATCTGCTTTTTCCAATTTGGAAGGGCCATTATCTGTAACTGATTGGTGCAAAGGACGCAATCAATTGGTTGATACTGGAAGCATAGTTGATGGAGTTTCTGCTGAATCCAGCATTAATGAATGTAGGGATTCTTCAGAACCAATGAGTCCATCCCAATCATCTGTTGGTGGATCTTCCAGTATTAAGGGTAGAAGTTTGTGTCGACATTAGTGGAGGTTTCTTGTTCATTATTATACTACTACTAATTATACTAATACTGTAATACAATTTGATTGCTATTGCAGTTTCCAACTTGATGGATAAGGTGGATGAGACTTCTCAAAGGAGATCTGGCCAGGATTTATGCAGTACAGAGTTAGAGCAGCTATCATGTTCCCGGCTAAAACCTACTCTCATTGCTCTTCCATTTCCTTCTATACTTGTAGGGTATGTACACTTGTTGTCATTTATGTTTATAGAAAAGTAAGTTTGAATACATAATTGCCTTATGGTGCAAGGCTTTTCATAGTATTTGAGTGCATTGGGTTGGGTGGTTTAGATGATTATGATGCTGCATAGGGTTGTTCTTATTACAAGATAAATAAATGGTGCTCGGGATTAAGCTTAATTTGAACATGCATTGCATAAGCATGGCTCATACAAATAAACTAACGGAGAATGACCAAGTGAAAAGAGTTAGGCATCGATAACGATGGGTAGGGTTTGGATATGATACTATAGTATCTATTCCCAACCAACTTTTAAAAGAACTTTTTCCTGCACTCATATTTGATCGGATAGCAACTTCAGTGTCCATCCCCATGTCTTATATCTACCTATATACTTGCTTCTATGACATGCATTTTAGTTAAATGAAATATGTTTTCTTTTTAATGTACCTTATTTTTTATAATCAACTAAATCTAGTAAAACAAAACTGCTGTTTAATATTAAACTAAAATTCTATTTCTATGATTGGTTGGATTGGGGAACAGCTGACTAATCTTAACTAGATGAAGATGAAGCCACAAAGACATTAGATGACAAGAGTTAGGACAAAGTTTTGAAATGAGAATACAGTGTGTGGAGACTGATAAGTCACCAACTAATTTGTGTTAATTGTATAATATTTTTTAATTTCAAATTTATATATCAAATAACATGTGGCTGAGGCAAGATGGGTATTAAAGTAATTAGATTTGCATCTGTTGAATAAACTGAGAGAGAAGGAAGAGAAAACTTGTTGAACATAAAGGCTAATTTTATTGATTATCAAAAGTTAATTACAGAAGTTTATAAAAGAGGTATATATAGACCTCTGTTCTGTTATTGTAACAAATTGTAACTAACTAACTGAACCTAACCAACTCTAACTGATTCTAACCAACTCTAAGATAGGTGCTGTCAGCAAAAGCTAGCAGCCCTTACAATTAGTTTACAGAACTGTTTTCACAGTTGACTAACAACATACTCTAATACCCTCTCTCAAACTCAAGGGGAAGAGTTTTCAGGAGAAACACTCTTCACATTGAGTTTTCCTTGAAGAACTTCAAATCGTGATGCAGAAAGAGGCTGGGTGAGAATATTAGTCCACTGATCTAGAGCTGGAACATGGACAATAGTAAGCTGCTTGGCGAAAATCTTCTCTCCTACAAAAAAGACATCAATTTCCATGTGTTTGGTACGGCTGTGAAAAACTAGATTGTGTGCAATAGAAACTGCACTCTGATTATCACAAAAAATAACAGGAGTAGTGAATGAAATTTGCAATTCTGTTAGCATAGTATGAATCCAAGTTAATTCAGTGGAAGTCTGAGTTATACTGCGATACGATACTCAGCTTCTGTACTAGACCTGGCAGTAACTTTCTGCTTTCGAGACCACCAAGAGATTAAGTTTGTACCAACAAAAATAGCAGTCCCTGAGGTAGAGCGCTCGTCATCCACATCTGATGCCCTGTCAGCATCACAAAAAGCTCGAATAACTAAGGGTTTTGTAACAGATGTAGGTTGAAGAAGCAAACCATGAAACAAAGTGCCCTTCAGATACCTTAATATCCTTTTTACCACTGTCCAGTGAGAATCCAAAGGAGCAACCATAAATTGGAAAACTTTGTTGACAGCAAAACTTATCTCAGGTCTAGTAAGGGTAGCATACTGTAATGCACCAAACCAACTATAGACCTGTATAAGGTTGGATCATGAAAAGCATTAGCACTATGTCGAGAGAACTTGTAGTTGGATACCATTAGAGAAGAGAGAGTGAGCTTCATCCATGTTAGTCTTGTGAAGTAAATCTCTAATGTATTTGGTCTGAGTCATAAGGATAGAACTGTTGGGTAGATACTTGATTTCCAGACCCAGGAAATAATCTAGATGATCAAGCTGCTTGAGCGAAAAAGAAGGGTTTAACTTGCATGTAATCTGCTGTATTTGTGAAGGTGAACTACCTGTTAGAATGATATCATCCACATAGACCAGGAGATAAACAACATGCATCTGTTGGCAAAAAATGAATAATGATGAATCACTTTTGCTTCCAATAAAACCAAATTGCAGTAAAGTAGATTTTATCCTGTCAAACCATTGCCTTGGAGCCTGTATTAAGCCATAAATGGCTTTGTTGAGTTTGCATACCAATAACTTATCTGTAACTTCAAAACCAGGAGGCTGAGTCATGAAAACAGTTTCTTCAAGTGACCCATTCAGAAAAACATTATTTATATCAAGTTGAAATAATTTCCACCCATAGACAAAGCCAAAGTAAGGATGATCTGAATAGTAACAGGTTTGATTGTAGCGCATGAGTTGAGTTTCATGTCCATAGAGCAACGCTTCAATCTCTGCGATGGACAGAGTTCGCTTTTTGCTTTCGATAACAGAAACCACCGACGTGTAATCTGACGATAGTCCTTCAAGCAGCGTGTCAACGTATTCCTCATGACGAACAGGGACACCAACACCAGCAAGTTCATCCACAAACCCTTTGATTTTTCGCAAGTACTCAGTTGTTTTGCCATCAAGAGTAACTACTCTCATGGCAGTGCGAAGTTGTCGTGCTCAAGTTTTCGTGTGAAGGCTAAAATGCTCATGAATTTTATCCCAAACCTAATAGGAATGATCTGAACCAAGAACACGCAACACTGATTTAGGAAGAGTTGACTGCAACCATACCAAGAGTGTTTGATCTTGGACTTCCCAGGCTTCATAATCAGGGTTGATATGATTAGTAACACGATCATCTTCTGTGAGATAACGAGGAGGAATAATCGGATTAACCACGAAACGTTGCAATCTGTGTGACTTAATAACTGGTTCAACATGTTGACGCCTGTGAAGATAGTTGGAATTATCAAGTTTTTCAGCCACAGAGTTGGGAAATGATTGGGGAATTGAACTGGAAGGAGCAGAAGCCATGAACACAGAACATTGGACCTTGTAAAGGAACTAGGAATTCAAAAAAATGGCTCTAGATACCATGTTGAATAAACTGAGAGAGAGAAGGAAGAGAAAACTTGTTGAACATAAAGGATAATTTTATTGATTATCAAAAGTTAATTAATAAAAGAGGTATATATATACCTCTGTTCTGTTATTGTAACTAACTAACAGAACCTAACCAACTCTAAGATGGGTGCTGTTAGCAAAAGCTAACAGCCCTTAAAATTAGTTTATAGAACTGTTTTCACAGTTGACTAACAGCATACTCTAATAGCTTCAAAGCTTTTTCAGGTAAATACCTGGATCCAAATCCTTTTAGTGGGTAATTTTGTCCCAAATTACAGGATATCAGATTGCGTTGTTGTATTGTTACCTAGAGTTCAATGCGTGAACACTCACATAAGGCAAATCAGGTAAGATGGTAGGGTTGCTGCTTTATGCAATGTTATCAAAGCCAGACCGGATTGGCTGGTTCAACCAGAAAACCTGCCTGGTGTCTGGTCCAATACACCCATATAACCAGAGCTACTAAAAACTGGCCAAACCAGTGAAAACTGACGGTTCTATGGGCTGAACGGGTTTCTGGAAACAAACCAAAAAAACGAGAAGATAAAAAATGAAGCGGAAAAAGATGATGTATAGAGAAGGAAGGACTAGTGTAAAAACGAGAGGCAAGCAGAGTAGTGCGACTATGGAAGAACTCTGGTGGGCTTGCAATGGCGCAAAACGAAATGGTGGCTGCAAATGGCACCTCTTGGCATCATCTGCTGGTGGTGGGTTAGAATTGTGGCTTTGATAGAGGAAGAAGAAAAGACACCATTCATGTATCTTGAATTTCTAATAAAAGCTCAATACTCTACTGAATAAAAGACAAAGGGTGAAAGACTCAATGAGCATTGCAACTCATGAGGCACATTGAAACTTACCAACACACACTTGTTGCCTATCATCTGCTTTTTACTATTGCATTTCACTTCATATAATTAATTTTTATAAATTATTATTTTTTATTTTAAATTATCCTATATTAATCCTAAATATATACTAGTACTTTTCCCTTAAAGTAATTATTATCGTAGATTATTATTTTGAAAAAAAATTAATGTATTTGATGTTTGGCTAGGAAAAATTAAATAATAATCCTATGAATAATTTTAATGTTATCAAGTCTATATTATCAGTTAATATATTTTCTTGTAAATTTAAAAATATTATTTAATAACATAACTGGTTCAACCACGATTCAACTATGGCTAAACCATTGAAGCTTTAATCAGTGTCTTGACTGGTTCAATGACCGGTCCGGGTTTAAAAACATTAGTTTTATGACCTAGAGTTCATGCATTGAAGTCCTGGAAACAGCCTCTCTGCTTGTGGGGCATAAGTCTGCTTACATGTACCTTCTATATACTGTAATATGGTATTAGAGCTAGGTTATTCTTAACAGAGGAATAATGAAAGTGGTTCTCCACCGAGGACCTATTGCCCCCAATGAACCTTTCTGCTCGCTGCATATCTTCCAGCGACCCTTTCTCCTTTTCCAATGCTTCTCCAGCAACCGCTATCACATGCCAAACAATTTTCTTTTCTTTCTTTCAGCATGCGTTTTTCTGTTTGCATGAACAGTAACATGAAGCGCTGTGAGCTTCCGTTCACCATTGCTGAGTCTTCTTTCTCCAGCGCAACCACACTCATGGAATCGCCCCCCTCTGGCAAGCAAAACTCTGCTGCACACGAACCCATGCAGGGTCCTGGTCCGTGAACTTCACGCACATAACACGTGACGCATCTCTGGCTAGTGTCACACTGATCTTCTCTCCCTCCACCTCAAGGCATCCTAGGGGGATCTTTTTGCTTTTTTCACCAGTGAACAGTACCTGTTGCCATCACGGAAGATTGCCACGCCGTCCACGTGCGCAGCCAACTCAAAACACCACTGCATGCAGCCCCACGCAGGGCCCTGGCATGTGAAGTTCACGCACATAACGCGTGACGCATCTCCAGGGAGCATCACACTGGTCTCCATCGTCACAGCAGGCCTTCTTTTCTATTGGGTTTACCACATTTGTAACAAGCTTGGTTCATATCACACATATGCTCCAACTTGAGGGGGAGTGTGAGATTATGTTTTGATAGTATTCCTGATATCTATTTCCTATTAATCAGAAATTGATTGATCTTGTAATCAATACTGATTTTCTTTTCTATATTATAAATAGCATGAGGTGTGGTCAACATTGACACACAACATTACACACTAAAACACTATTATAATTATATATGAAATTCAGAACTTATTCAAGTTATCCGTCCTTCTTAAAAGCTTATGCTTGTTTAGTAAAAGTGCAAGCTCAGCTCGGCATTATAGCATATGTTGAATATAGTTGAATGTCATTAATTTTCAGGAATTGAACACTTAAAATACTATATATAGAATAGTCTTAGTTAGTAGATTGAGCAGTAATATAAAAATGGGATATGTTTGTATTTATTTATGTATAAAAATACACACTACGATTTGATTCTCATGGAATTCAGCCATAAACAATATCAGGGTGCTAAAAATAACCCAATAGATTAATCACTTGCTTATTTGCTGGTATATTGTCCATCATATATTGAATATGATAAGAATGCTAGCACAGCAGGCATCTGGTACTGTAAAGTTTGGTATTTAGTTAACCACTCATGCAAAACATGCCCAATAGGTATCAGGATGATTGGCTCAAGACATCAGCAAACTCTTTGCAGCACTGGGAGAAGGCTCCTCTAGAGCCCTATGCTCTACAGAAACCTGTAAGTTCATTATCACTTATGGCTGCTGTTACTAATGGAACCTTGTCTATCATATAGCCTGACTTAATTACCCGATGGGTAATGAACTTAAGAACTTGCTTTCATTTAAATCATTATATGCATTGTTGGTTGCAGATTACCTATCATGTTGTATGTCCCGACATTGATCCCCTTACTTCTGCTGCTGCTGATTTTTTTCAACAACTAGGAACAGGTAAATAGTGGTAATTATGCTTCAATTAATTTATAAGGACTATTTGATGATGATTACAAAATGCATTTTGCAACGTATTATGCTTTATGATGGATTTGATAATTTTTGTATTTATGTCTACTTATTTATCATGTATTATTGACAGTAAAAAGGATGGAAGAAAATGTGGAGAAACCATAGCTAAATCCTGTCTCTTAGGAAAGAAAAGTCATATCTTTTTTATGGATGTAGATGCTATTTGAGGTGCTTAGAGCATTTTGTGTTTCTGGACTTTCTTGTGTGTTGTACTAGTTGAACTTGAATGCAACAATAGCTGATTGCTGTTGCAGAGCAGAATGCATCCAGATTGCTATGCATTGTGACTGCTGATTTAAGTTTCACCTAATTTTTTTCCCTTCACTCGGATTATGTATTGATTTGTTCTTTTAGTCTGATTTTGCTTTTATTCTTTTCAGTGTATGAGACATGCAAGCTAGGTACTCATTCACCTCAAGGCTTGGGGAATCAGATGGAGATAGAGTCCGCAAAGTTGTCATCTTGTGGTTTTGTTTTACTTGATTGCCCCCAATCAATAAAGATTGAGAGCAGCAATGCATCTCTTGTTGGTTCAGTAAGTGATTATTTTTTATCTTTATCTAATGGTTGGGACCTGACTAGTTATCTCAAGTCTCTTTCAAAGGCACTTAGAGGTTTGAAAATTGGCTCTTGCTTTTCTACAAACCCCGGCGAAGGAAGTAACAGTTCATGCTTGGTAAGTGACTTGTTTAGTGGCTCAATTTTGTAGATTGAATGGTTATTTTGTTCCCTAAGTGCTAATCAGACCAAATTATGATGAGGCTTTAACAGCCTAGTTGGTTGTTTGAAGCATTAATGACATGACACTGTTCATAGAAGAAAATAGTTTTCTCAAAGCATATTTCTCTTTTTTTTTTTAAATCGTAGATCTACGTGCATTGATGCTTTTGATCTCATTTTTTCCTTCTTACATTTAGGTAATCTATGTGGTGTGCCCATTTCCTGATCCCACTGCAATTTTGCAAACTGTAATCGAATCTTCTGTTGCCATTGGATCGGTTGCCCAACAGTCAGATAGAGAGAGGAGATCTAGCTTGCACAGCCAGGTTGTGAAGGCATTAAGTGGCTTGACCACTGTCGATGAAGCTTCAGCGTCTAATATCCTTGTGCTTTCTGGGTTTAGTATTCCAAAATTGGTCTTGCAGATAGTTACAGTGGATGCCATTTTCAGAGTCACAAGCCCATCTGTTAGTGAGCTTGTCATTTTAAAAGAGACTGCCTTTACTGTATACAGCAAGGCTCGTCGCATTTCACGTGGAATCTCTAGTGATTTTGCTCAATCTGCATTTCCTAGATCTCATTCTGTTTTGACACAAATGCCTTCTCCCATCTCTGGGATGTGGAAAGACTGTGTTGGTCCTCGAATGGCAGGACATTCTCTTCCAAGAGAGGGTGACATTGATGCTAGCTTGAGGCCTGGTACTTGGGATAATTCTTGGCAACCAACAAGGACTGGGGGGTTAAGCTGTGATCCAAGTAGAACTGGAGATAATTTTCTTTATGATGAAATTCGTTACATGTTTGAACCCCTTTTTATTCTCGCAGAACCAGGTTCTCTTGAAAATGGTATTTCAGTTATTGGTAGTCCCACTTCAGAATCTTCCAAGGCATTGGCAGATGACAGTAGTGGTAACTATGCACAGAGTACAAGTACAGCTGGAAATGCGGAGTCTGCTTCAAGCACTGATGGATCTGGGTCCGATCCGGAGACTCCTCCAAGCCTACATTGTTGCTATGGATGGACTGAAGACTGGCGTTGGCTCGTATGCATCTGGACAGACTCAAGGGGAGAATTACTGGACTGCAATATTTTCCCCTTTGGTGGAATAAGCAGCAGGCAGGATACAAAGGGTCTGCAGTGCCTATTTGTTCAAATTCTTCAGCAAGGTTGTCTGATACTTCAATCTTGTGATCCTGGCCTTGCAAAGCCTCGAGATTTTGTGATAGCACGCATTGGAGGTTTTTATGAGCTCGAGTACCTTGGTAAGTCTGTCTCTGCCTCTCTCTTGTGAGATATATGATATATAAATCAGTTTTGGGGCATGGTACATCCCCTCATGTAAAATGGCATGGCTGATGACATTGGCTAGGACATACGGTGTAATTTTCTGGAAGTTAATTGAAAAACACTTCTACAGTCCTAAAGATAATGACCAATTCACACATTGTTGCATACATGTGTGCATAAATTCCCTCTGGTACTATTTCATCTATCTAATACATATTTATTTTTGCTGAGCAAAAAAAAAAAAGTGTTCATAAATTCCCTGATATTGGCCATACCTATACTGATACCGGTGTCAGTTGAGACTGTTGAGTGAGAATTATTGCATAGTACCCAATGGTGATGTTATTTGCATGGTTATGAAGTGGTTGGTGGGTCATTTTTTCAAATTCTAATATATTATACACCACAGGACTTCTTAAAAAAATCGTGAAGTTTAAAACCAGTTCCATTGAAATTTGGGTGGGTCCAGTGTAGGTTTCGATAAGTTCAGTTATTGCCAATCTGTAATGTGTAAATGTAGCAGTGGTGTGCAAAAGCTTCCTTCTGAATTGTGTGAAGCAATATCTCACAGAACAAGTGAGCATCTCACTTGTAATAATGCAATTTAGCTGGCTCCATTTGTTTTAGTACATTCACACAAAATATTTTGGATTAAAAGCACAGTCACTTTTAATTATCTAATTTATTTCATCAAATGTATATAACAAATTATCAAGTGATTTCATGTCAGAAAATTCAAGAATAAAAGAAATTAGAAAATTCATTATCTCCTCTGTCATGTTATAACTAATTATTATTATTAGAGAAAATTACATTGACACCCCATGTGGCTTCTTCAAATTGCACACAATACCTCTGCCTTTTTAACGTTCACAGTGACCCCCCTTAGAGGGGTACACATGCAATGCATTTATTGAGAAACAGGGCATGTCACTACAATGTATTTCAAACAATTAAGGGGGTTATTGTAGGGGTTAAAAAGGTAAGGTATCATTTTCATCTTGAAGAAACCATAGGGGGTGTCAATGTAATTTTCTCTTATTATTACTATTATTGTTGTTGTTGTTTTAGCCTGTGACATGTAAGTTAGAACTGATTCTTGCATTTTATTATCTATATATGATTCCATTCACCGTTTAGTTTATCAAAATGAACTTTATTTCTTGTTGATAATGATGAATTTTGCCAGAAGTTTGAGGTGTAGTCCCAGATTTATTTGAATTTTGAACATGTGGGTTTAGACCATATTCAAACTGTTCCAACCTTAAAGTTATACACACAAAGGTTCATGGTCGGTTGTTGGCATATTTACATACATCAAAACATACATCAAAAGTAAATTGGCTTTATATTTACAACACTTTAAGTTGAGAAGCTAGTTGGTGTACATTTGCTCCTTATGTCTGCATCTATACATGCTTTCTTACTTTGGCATTTTATTCAAACAGAGTGGCAGAAAGCCATTTATTCAGTTGGTGTATCTGAGATGAAGAGATGGCCTCTGCAATTGCGGAAATCTATGTCTGATGGGATGTCTGCAACTAGTAATGGTTCTTCTTTGCAACAATCAGATATTAGTTTGATCCCCGAAAGAACTCTTCCTTCCTCACCTAGTCCTTTGTACAGCCCTCATACTAAATCCACTAGTTTTATGAAAGGCAGTTTAGGGCAACCTACTGCAAGAAAGCAGCTTATGGGTGGACATTCTATGGTTGACAATTCTAGGGGTTTGCTTCATTGGGCACAAAGCATTAGTTTTGTTGCAGTTTCCATGGACCATACGTTACAACTAGTTCTGCCAGCAGATTCATCAACTCCTGGTGAGTTATCTTTACTAGAATAATTTTAACTTCTATGTTCTTTTCTTCTTTCATTAAAGAACACTAATTTAGTACCCATCCTCAACTACAATGGTTAGGGTTTGGGTAGGGTACTATAGTATTTGTCCCCATCCTCACATTTTATAAAAAAAACTAGTCCCCAATCCTGCGTGGATAGAAAATTTAGTGCCCGTCTCTGTATCTTGTTAATACGTAGATACACTTACCTAGACCCAGTAGTCTAGTACCCATGCTTAGTTAAATAAAAGTGTTATGTCATATCCTGATTATAAAACATGACAATCAAAATATGTAGTTAGATTCATATCTATAAAGTAATTAACTATAAAACATAAGTCCAATTATGTCTAATAATTTTATATTCTTTGACTTTTCAAATTCATAGAGATTGGTATCTGTAATTTTAGGAGAAGAAAATTATCTTTATTTTTGTACATTAATCAAAATTACAAGAGAATTATATAGGCAGAGGAGGCTAGGAGAGACTCTACCACCCTGTTTACAAAAAAAATATAATGAGAAGATAGGGAGAAATAAAGGAGAATCAAATCAAATCAAATCAAATTTGAATAATCTAATCTTGATTGATTTTAACAAACTAAATATTCTAGAATAAAGGAGAATAAAATCAAATCAGAACTGAATAATTTGATCTTAATCTTACCTACTTAAATCTTCTAGAATTCTTATTTGAACTTTAAATTTAAAGTAAAACACATACAACTGTCAATACCCCCTCAAGTTGGTGCATAGATGTCCTACATGCACAACTTGCTTATAAAGAAATCAAAATTAGGTCTGAATCCTTTTTTAAGAATGTTTGTCACTTGCTGTGAAGTAGGAACAAATGGCATGCAAATTAGGCCTACATTCACCTTCTCTTTTATAAAATGTCTATCAATTTCTACATGCTTGGTCTGGTCATGTTTAACTGGGTTCTGGGAAATATTAATTTTAGCCTTGTTATCATAGTACAACTTCATGTGGAGTCATAGGAAGTTGCAACTCTTGTATTATTTTTTTTAGCCATAAACTTTCACAGACTCCTTGTGCCATTGCCCTACATTTAACCTCTGCACTACTTGCCACATCATTTTTTTTTCTTTTTTTGCTTCTCCAAGTAACCAAATTTACCAAACCAAAAGTACAATAGCCAAATGTTGACCTTTGGTCAGTAATGGATTCAGCCCAGTCCACATTTATAAACTATTGGAGTATGTAATTGTACAATTGTGAAAACAGTTTTGTAATCTAATTGTAAGAGCTGTTAGCTTTTGCTAACAGCACCCATCTAGTGTTAAACAGCTGTAGTATGATGATAAGGGCAGTGAGCTTTGACTAACTGCACCACATTAGGATACAAGGCTATATTCTGTTAGTTTTTGTTAGAGTTAGTTAGAATATGTTTTCTGTTATGTTAACAGGACACAGGTCTATGTACCTCTGTATGTAACTAACTGCAACTAACTTTTCTGATCAATAAAAATCAGTTTCACTTTTGTTCATTTTTCTCTCTTTTCTCTCTCTTAGTTTGTTCAACCTGGTATCTAGAGCCATTTTTACGTAATCATGGTTCCTTCGCAAGGTCCAGAGTCCTGCGTTCTGGCTTCCGCTCCTTCGAATTCAGTTTCAGTGGTTTCCCAACTCCATTGCCGAAAAACTTGATGATTCCAGTTATCTTCACTGGCATACATGTTGAACCAGTTATTAAATCACATAGGTTGCAACGTTTTGTCGTCAATCCAGTTGTTCCTCCTTGTTACCTCAGAGGATGATCAAATTGCTGATCGCGTCAACCCTGGCTATGAAGCCTAGGAGGTTTGGCTGATCGCGTCAACCCTGGCTATGATCGAATTGCTGCTCCTGGTTTGGCTGCAATCCACTCTTTCAAAATCGGTATTATCGCGCATTCTTGGCTCCTGTCATTCCTATCAAGTTTAGGATAAAATTCACATGTACTTTAGCCTTCATACGAAATCTTGTGTGCGATAGCTTCGCACTGCGATGAGAGCAGTTACTTTTGACAACGAGACGATTGAGGAGTACTTGTGCAAAATCAAAGGGTATGTTGATGAACTTGCCAGCATCGACGTTCCTGTTCGTCACGAGGAATGCGTTGATGCGCTCCTCGAAGGTCTACCGTCTGATTACGCGCCAGTGGTCTCTGTTATTGAAAGTAAAAAGCACACTCCATCCATCGTAGAAATTGAAGCTTTGCTCTATGGTCATGAAACTCGACTCGCGCTACAACAGAGATGCCCATGTGATAAGTTCTGCTTTGTTAAACTACACTCAAGGATATTTGCATCCAAACGCTTATAAAACTGGTGATTCTGGTAGTTCTTGAGGTTCGTATGGTCGCGGTGCCTTTTCGGACTGAGGTGTTGGCCGTGGTGGTGGTGTCTTCGGCAGAGGATGCAGCAGTGGACAATTTGCAAACTTCCAATGTCAAATCTGTCTTAAATATGGGCACACTATCAATGTTTGTCATTTTAGGTCTGATGTGAACTTTCAACCTCACGAATCTCTAACTTTCATTGATCCTACCATACTTCGACCCATCCCCTACTCTACTGGTTCAGTCAGAACCTCCAACACCTGGGTTAATCCGAATTCTAAGAATCCTGCTCCAGGTCACAGCCAACCCAGTGCAATGCTCACTAATTCATCCTCTCATGGGAATGGTCAAGTTGGTACCACCTGGATTCCAGATTCTAGAGCGAGTTTTCATGTGACTGGTGACACAGAATATTAAACAATTCACCCACTTCGATGGACCAGATCAGATATTCATAGGCAATGGTGAAGGTTTAAGTATTTCTAACATTGGTTCCTCATCATTTGTGTCTCCTAATGATGTTGGCATAACCTTTAAACTTCACAAATTATTACATGTTCCTTCAATTTTGAAAAATCTGTTATGTGTTAGTCAATTTGCCAAAGTTAATTCTATTTTCTTTGAATTTCATCCTCATTTGTGTCTTGTTAAATCTCACGAGACCAATAAGATCCTTCTTAAAGGAGTTTTTGGTGTTGATGGACTTTACTCCTTTCATAATCTCAAGCTTCAAGAGTCCTTCACTGCTGATGTCTGCATCTACTATTTCAAATGCTGACTTCTCTAGTTCAGCTCTCACTGTTAATAATAATAGCTCTACTGTAATGTCTAATTCTGTTGTTTCTTCTTTCAATGTTGCTAATCTCGGCATGCTAGGTTAGGGCATCCTAATGATCATGTAATGAAAGTTGTTCTCACTCATTGTAACATTTCTTCATTGAATAAAAGTTCCACAGGATTTTGTTCTTCTTGCTGTATGGGCAAATATCATAAATTACCCTCTCATAGCTCTACTTCTGTTTATTCTCCTTTTTAGAGCTTATTTTCACAGATTTGTGGGGACCTTCTCATATTACCTCATATTCTGGTTATAAATATTATGTCTCCTTCATTGATGCTTTCTCTAGGTATACCTGGATATTTCCTATAAAGTGCAAGGTTGAAACCATTTCTGTTTTCCAAGCTTTCAAGTCCATGGTTGAATTGCAGCTTAACACCAAAATTAAGAGTATTCAGTCTGATTGGGAAGGTGAATACAGACCTTTCTCTACTCTTGTAGCCTCCTTTGGCATCTCTAACAGACTGGCTACCCACACTCATCACCAAAATGGTGTGGTTGAAAGAAAACATAGACATATAGTCGACTTGGGTCTTACATTGTTGCATCATGCATCTTTACCCTTGCAGTTTTGGGATTATGCCTTTACTACAGCTGTATACCTCATTAACAAGCTTCCCACCACTTCCCTCAAATTTGCTATTCCTTTTGTTACCTTGTTTAATAAGAATCCTGATTATCATTTTCTTAAAACATTTGGATGTGCCTATTTTCTTTTATTGAGACCATACCAGACACAAAAACTGAATTTTCGATCTCAAGAATGTTTATTTCTGAGGTCTTCTTCCCACAAGGGATACAAATGCTTATCCTCATCTGGTAGAGTGTACATTTCTAAAGTTGTTTTGTACAATGAATTAAGGTTTCTCTAATCTGATCTGTTTCCCCTTCCACTAATTCCATCACCAGCTTTTCCTACTTTAGTCTCAATCCTAATCTTTTTCCACCTTTTGTCACATCGATACCTCAATCATCTCAAATGTCACCTGCTGATACAGCTTCTATTCCCCTTGTTCTCCCTGGGTTTTCTCCTTTACCAGCTCATTCTCCCATCACCATTGTTTCTGTACCTCAAAATTCTACTTCCTCTGCACCTGTCTCTGTTCAGCCTCTAACTTCAGAGTCAACTACACTTGCTACATCCTCTTCAGAGTCTGTTTCTGTACCAAATTTTGTACCAGTAAATACTCACCCTATGCAAACTAGGTCTAAATTTGGAATTCACAATCCTAGGTTACATCCTTCTCTGTTTCTTACCCATTCTGAGCCTAAAACTGTAAAACAAGCTTTGGCAAATACAGACTGGCTTACTGCTATGCAGTAAGAGTATGATGCCTTGCTGAAGAATAAGACTTTGGGACTTATTATCATTGCCTCCTAAGAGACAAGCTATTGATTGTAAATGGGTTTTCAGGGTGAAGGAAAATGCTGATGGTTCCATTAACAGGTTCAAAGCACGGTTGGTAGCCAAAGGATTTCATCAAGTTCATGGTTTTGATTTTCATGAAACCTTTTCTCCTATGATCAAGCCTGTTACTATTTGGATTATTCTTACTCTTGCTTTGTCTAATGGGTGGAAATTATTTCAACTTGATGTAAACAATGCCTTTCTGAATGGGTCACTTGAAGAAACTGTTTTTATGGCACAACCTCCTGGTTTTGAAGTTGCAGATAAGTCACTGGTCTGCAAACTCAACAAATCTATCTATGGCTTAAAACAGGCCTAAGACAATGGTTTGACAGGCTAAAGTCTACTCTGCTGCAATTTGGTTTTGTTGGGAGCAAAAGTGATTCATCCCTATTCATCTATCGGCAATAGATGCATGTTGTTTTCCTTTTAGTCTATGTAGATGATATAATTCTAATAGGTAGCTCAACTTCTCTGATTCAGCAGCTTACATACAAACTAAACACTGCCTTTTCACTCAAGCAGCTTGGTCATTTAGATTATTTCTTGGGTTTGGAAATCAAGTATCTATCCAACAATTCTATATTGATGACTCAGAGCAAATACATTAGAGATTTACTTCACAAAACTAACATGACATGGCTGAAGCTGACTCTATTTCTTCCCCAATGGTGTCTCCAACTGCAAGTTGTCTAGACATGGTGCTGATGCTTTTCATGATCCAACTCTATACAGATCTGTAGTTGGTGCATTACAGTATGCTACCTTTACTAGACCTGAAATCAGTTTTGCTGTCAACAAAGTTTGTCAAGTTATGGCTGTCCCTTTGGATTCTCACTGGACATTGGTGAAAAAAATATTAAGGCATCTGAAGGGCACCTTGTTTCATGGTCTGCTTCTTCAAACCACTTATGTTACAAAGCCCTTGGTTATTTGAGCATTCTGTGATGCTGACTGGGCATCTGATGTGGATGACAGGCGCTTACCTCAGGGACTGCTATTTTTCTTGGTCCAAATTTAATATCTTAGTGGTATCGCAAGCAAAAAGTTACTGCCAGGTCTAGTACTGAAGCTTAGTATCGCAGCATAGTTCAGACTTTCACTGAATTGACTTGGATTCATACTTTGTTGACATAATTACAAGTTCCATTCACCACTCCTGTAATCTTTTGTGACAGTCAGAGTGCTGTCTCTATTCCACACAATCCAGTATTTCACAGCCGTACAAAACACATGGAAATGAATGTTTTTTTTGTAAGGAAGAAGATTCTGGCCAAGCAACTTTCTATTGTCCATATTTCAGTTCTGGATCAGTGGGCTAGTGTACTCACTAAGCCTCTTTCTGCATCAAGATTTGAAGTTCTCATAGGCAAACTCAATGTGAAGAGTGTTTCTTCTGAAAACTCTTCCCCTTGAGTTTGAGGTGGGGAGGGGGGGACCTCATTAGGATACAAGGCTATATTCTTAGTTTCTGTTAGTTAGAATCTGTTAGTTAGTTAGAATCTGTTTTTTGTTAGGTTAACAGGACAGAGGTCTATATATACCTCTGTATGTAACTAACTGCAACTAACTTCTCAGATCAATAAAAATCAGTTTAACTTTTCTTCAGTTTTCTCTCTTTTCTCTCTCTTAGTTTGTTCAACATAAACACTTCAATGCCTTGCTGCTCAGTTTTCTTAAAAGATAATCATCTGCTCATAGCACTTTTTAATTACCTCAATCCTGTAATCTACCTCAAGGTATTCTCCATGTGGTGAGTGCATGAATTGACTCACTAAACTTACTGCAAAGGCTGTCTGGCCACCATCGGATATGAGATAAATAAATTAATCTTCCCTATTATCTAAGGTATCGAGTTGTATCTACTGAATTTTCTTTGTCTTCACTTCCATGTTTTTGATTAGGATCTATAGGAGTTTTAAGCTTTCCTGCAACCCACTTACCCACATCTCAATTTCTTTGAGAAGGTCTAGAACATACTTTCTTTGTGACTCAACAATTCCTTTCTTTGATCGTCCAAGCTGTCCCCCAAAAAATATTTTAAGGACCCAGGGTCTTCAATCTGAAAATCAGCTGAAAGGGAAGCTTTCAACTTATTTATTTCGTTGAAACCATCCCCAATAATGGCAATGCTGTCCATATAAAAACAGTTGGCACTGCCCAGAAATTTTTTGACAAACTTACTGTCACTTTGACATTGAACATACCTTTGCTACAGCTACAAATTTGTGACGATTACATTTTTCAAAGTTGCACTGCACTAATATACTAAAATCCTGTTTTGGCATATGACATAAATAAAGGAAATTCAGACAAATATCCTTCAAGTACTTGAATCTATTATGGATTTTCAGTATGGATAGACTTCATCATTATTCTTGTTGAGAATCAGTATTGATATTCTGCATTCATTGTAATAAATATAGTTTATAGGCACATAATCTCTTATTTAGAGGAAATCAAATATCCTTCATTTATTTGGAACAAGTGCAATAAGCCTATATAAACTCATTCGTGCTGTATTCAGACACATGGTTCCAGTCTCAATATGCCTCTGTCCTTTCTCTTTAACAATTTTCAACTTAGGATTTCTGGATAAAAAAACTTATAGTTGTACAAAGGAATTGGAGAATGGACTCTCTAGAATTTCACAAGCAACCAAGAAACAAAATTTTGAATTTGCAATGCTGCCTCATATTTTAATATTGTTAATGAAGTAAATCATGTAAGTATTGATAGCAAAAACTTTTTGGAGGAGGAACTGAAAGTGGTGGTGGTGGTGGTTTGTCTATTTCAGGTTACATAGAAGGTTTCACTCCAGTGAAGTCTCTTGGTTCTACGTCTTCTGCATATATTCTAATTCCTTCTCCTAGCATGCGGTTCCTTCCTCCAACAGTTCTTCAACTCCCCACATGCCTCACAGCAGAGTCACCACCACTAGCTCATCTGCTTCACAGCAAGGGTTCTGCTCTTCCGCTCTCTACTGGATTTGTGGTTTCAAAAGCAGTACCTTCCATGAGGAAGGACTATAGAAGCAACCAAAAAGAAGAATGGCCCTCTATACTGTCAGTGAGCCTCATTGATTATTATGGAGGTACTAACATCCCCCAAGAGAAGATTGTCCGAGGAATTAACAAGCAAGGTGGTAGAAGTTTAAGCTGGGAAGCTAAAGATTTTGAAATTGAGACCCACTTGGTTCTGGAGTCTCTTGCAGCAGAGCTTCATGCCTTGTCATGGATGACGGTAAGTCCCACGTACTTAGAGCGGCGAACAGCACTTCCTTTCCACTGTGACATGGTTCTAAGACTAAGAAGACTTCTTCATTTTGCTGATAAAGAGCTCTCCAAGCAGTCAGAGAAATCTTGACTATGATTTATATATATACAATGGTTGCGACAAAACTCTTATCATTGATTAGAGAGGAAAGACAGCCAAACTGAGCTGAGTAGACTGTATCATACTAGATACACTGTGATTACATGTAAATTAAAGGTAGAAGCTGATGTGTTCCTGGATGAAATAGATTTAGCAATTGTTGGGAATGTACATGAGCTTTTCTAGAACAGCTGATTTTAACACAGATTAGCATGATTGATAGAAACTGTTGATTTTAGCATAGAATACCGTATTTTCGCTCTTTGTAGCATGTGGGTCTCCGAAATTTCATTTCCTTTGTAATTAGATATTGTAAGACAATAATGTGCTAGTAACTAATTTTATGCTTAATATTGGTTTCCTAGCTATAATTA

>GmMED14-1

CAAATTGGATGAAACAAATCCCAAATTGGTGGCTGCTGTGAGAGTCAAAAAGGCTTTTCAAGAAAGAATAAAGCATTTTTGTGAGTAAGAGCATTTCACAACCAACATTATACATACTGAGGAGAGAGAATCAACTAACCTGAGTGAGACTAGTTAAAAGTGTGTTAGAGAGAGAAAGAGGAATCGGGAGAGGGAGAGGGCGTGGTCGTTTTCATTTCATTTCATTTTTGGTCTTCTTGTTCTTCTCCAAATAAAAATTCTCCCTATTTCGTTTCCTTAAAACCCTAATTTTAATGTTGCTTCTCTGACACAACACAATTCATCGGTAAAAACCTAACCAAACAAGAAACCAATGACTGCCGAGTTAGGGCAACAGACGGTGGAGCTCTCCACCCTCGTTACACGCGCAGCTAATGATTCCTATGCCTCTCTCAAAGAACTCGTCGATAAATGCAAGTCCTCTGAATTATCCGACACGGACAAGAAGATTAGCATCCTCAAATTCCTCAGCAAGACTCAGCAGCGCATGATCCGCCTCAATGTCCTCTCCAAGTGGTGCCAACAGGCAATTCAATTCGCTCCCCCTTCTATTCTTCAATTCCAATTTCATGCTATTCCATTATTATTTTTGATCGAATTTCCTTATAATTGATTCGTTTTTTTTTTTTTTTTTGCTTGCAGGTTCCTTTAATACACCACTGTCAGCAACTGGCTTCGACTGTTTCAAATCACGACATGTGTTTCACGCAAGCTGCTGATTCGTTATTCTTTATGCACGAGGGGCTTCAACAGGCGCGTGCCCCCGTTTACGATGTACCTTCCGCCATTGATATTCTTCTCACCGGAAGTTACCAGCGTTTGCCCAAATGTATAGAGGATGTGGGGACTCAGTATGCTTTGACCGAGGAACAGCAGAAACCTGCTTTGAAGAAGCTGGACACGCTTGTTCGGTCCAAATTGCTTCAGGTTTCTATTCCCAAAGAGTTTTCCAATATTATGGTTTCTGATGGTACCGCAATGCTTAGGTTAGATGGGGAGTTTAAGGTTTTGATAACGCTTGGGTATAGAGGGCACTTGTCTTTGTGGAGGATTTTGCATTTGGAGCTGCTTGTTGGTGAGAAGGATAAGCCTGTGAAATTGGAGGCAACACGACGTCACTTGCTTGGGGATGATTTGGAGAGGCGAATGGCAGCGGCAGAAAATCCATTTTCGGTGTTGTACTCTGTTCTCCATGAGCTGTGTGTTGCACTTGTCATGGACACGGTCATAAGGCAAGTGCAGGTTCTTCGACAGGGAAGGTGGAAGGATGCAATTCGGTTTGAGCTCATATCTGAGGGGCATGGAGCGAGTTCTAGCTCTGCGCTGAACCCTGATGGGGAATCTGATTCGTCTGCCATGCGAACTCCAGGGTTGAAAATTGTGTACTGGTTGGATTTTGATAAGAATGCTGGTGCGTCTGAATCGGGCACATGCCCATTTCTCAAAATTGAACCAGGGTCAGATCTTCAGATAAAGTGTCTCCACAGCAGCTTTGTCATAGATCCCTTGATGGGCAAGGAGGCAGAGTTTGTTTTGGACCAGAGTTGTATCGATGTTGAGAGGTTGCTGTTGAGAGCTATTTGTTGCAACAAATATACCCGTCTATTGGAAATTAAAAGAGAGCTGGTAAAAAATGTTCAGGTCTGTAGAACCGCAGATGATGTTGTGCTCCAGTCTCAAATGGGTGAACTTGATATTGAATATAAACAGGTTGGTTTACATTTATTGGGAAAAGTTTTCTCTTATTGGGAGTTTGGAGTGTCAAATTGGCGTGTTGCATTGCATTGGACTTTAGTAGGTTTGTAAGGATTAAATTTGGATGATTATTTTGGGGTTTTATTTTATGTGCTTGTTTGGTGTCTGTCCTTTTGGGGTTTGACTTTGCTTACATTCTTTTAGTTTCAGAAAATAGATTCTCAGATCCTGGATATTCAACTTTTTTTTTTAAATCCTGGATTATCGAAGTACTAGGCCCGATGTACATTTGTTGCTGAACAATCAATTTTGGCTAATAATGCTTTATGTAGGTTAAGATAGATAATTTTGAAGTTCTGGTCAAAATTATAGTCATCTTATTTCTGCGTTATCTGCCAAAGATTTGGGGTTTACTATTGTTGAGATACCACATCGACCAAAGACATGACAAATCTAGTCCTTACAAGGCTTCAACAGTCCTCAACCCCACAAAATCGGTTTGTAAGATGAGGATTGCACAAGACTATAAGGACTACATTAGCCATATCTCTAGTCAATGTGAGATCTCAACATCCCTCTATGTTGATATGCACGTGTTTTCTATTGAAAGACTCTTCATCCTTACATCATACAAACTGTTGCAATTTTATAGATTCATTCTTGAATCTTGATACCTTCCCATCAATATTATGCGTGTAATCTGATGCCCTACCTTCTTCCATTCAATCTTGCTTGGGGTTTTGAATTTGAGATCTGCCTCTGTCTGTAGAAGGATGATAAGTGCTGCAGCAAGGACTCTGAGGGCCATGAGGTTTTGTGTGTGCGTGCCTATGGCTCTTCTTTTTTCACTCTTGGAATTAATATAAGGTACCTAACTTTACTTGCATTTTTCTAATCTATTGCATTTTAATTTGGGGGATTTTTTCAAACCCATTCCTTTGTTTTGCTATTCAGGAATGGCCGTTTTCTTCTTCAATCTTCTCAAAACATAGTGGTTTCTTCCGCTTTATTAGAATGTGAAGAAGCTTTGAATCAGGGATCTATGACTGCAGCTGAGGTCTTCATAAGCCTGAGAAGCAAAAGTCTATTGCACTTATTTGCATCTATTGGAAGGGTTTTAGGCCTTGAGGTATATTTACCTGTTTTCTCTCCTTTGGACTGTCTTCGAGCTTATTATTTGGATTACTGGCTTACCTGACAGTCTAAGACCTATGGATTATGGGGCATTCTTGCAGGTATATGAGCATGAATTCAATACAGTTAAAATACCCAAGAATGTTTCAAATGGTTCAGCTATGCTATTGATGGGATTTCCCGACTGTGGAAGTTCTTACTTCCTGCTGATGCAACTTGATAAGGATTTCAAACCCTTGTTTAAGTTGTTAGAGACTCAGCCCAACCCATCTGTAAAAGATAATTTATCTGGTGAGCTTAATCAGGTGCTGCGGATCAAGGAAATTGATATAGGACAAATGCAAGTTCATGAAGATGAAATGAATTTAAGCCTGGTTGACTGGGGAAAATTACGCTCTGTTTTGCCCAATGCTGTTTGTCCAAATCAAACATCTGGACATGAGTTTTTTTCTGATATTCGCCTTGAGAATTCCATACAGATTGCAAGAGGTCATCCTTCAGGTTTTTCATCACTTGTTGATGAAGTGTTTGGACTTGAGAAAGGGTCTTCAACGCCTCCATTCTCTGTTAAAAATCTTTCTTCATCTGTGAATACATCCCTTCCTTCCCAATATGGCTCTGTCCCTATGACTCTCCATAGTTTAAAGGCTGGAAGCCCTTCGCCAAAGTGGGAAGTAGGAATGCAGATGCCACTGGTTAGTAATGTAACAAAAGCTTCTAGTGCTACTAACCACTATAGTGGTTCCTTGTTCTCATCAGGCAGTGTGAAGGGCCCAGTTCAGTCCAGTTCTGTTGGCTCAATACCAACTGGACAAGGAAGGAACTCTGCTGGAACAAAGCTATCTGCTTCCAAGTCTGAACAGGATTTGGCTTCTCTTAAATCTCTGCATTCAGTTGACTCTAGTTCCTCTGCTGCAATGGATGAAGAACAGCTAAGAGTGTTCAGTGATAATTCCAACGATGCTTTAGCTGGAAGTCGGTCATCTCGACTTTTATCTCCTCCACGGCCAACTGGCTCTCGAATGTCCATACCTAATTCCAGACCTAATGGACCTCAAGTTGAATCGTTTAAGGCTGCTGGATCTGGTTCATGTGCTACAACTCCCGTATGTAAGATACTTTCTTTGCATCTTCATGTTTATCATGTTGAGTTTATTCATGCAGTTATTTATTTTATCTTTCTTTAAGTAATATATGACTGGAAAAATGGATCTTTGCAGCCCAAACACTGGAATCAACGGTTAGCTATAACACAGGTGAAGATGTTACCTCCAAAAATGATAGGAAATCTGGAAAACGAACAGCTTCGGATATGTTGACCTTGATTCCATCTCTTCAAGGTGTTGAAAGCAACTCGGGAATCTGCAAGAAAAGAAAAATTTCTGATTCAGCTGGCTGTCAACTGTCTTTGCCACAAGGTGTCATGTCCGCTGAAATAATACCCAGAACAGAAGGATACAGTTATGGAAGTTTAATCGCAGAAGCAAATAAAGGGAATGTTCCATCTAGCATTTATGTTGCGGCTCTTCTTCATGTGGTCAGGCATTGTTCACTTTGCATTAAGCATGCTAGACTAACCAGCCAGATGGATGCATTAGACATCTCATATGTTGAAGAAGTGGGTTTGAGAAGTGGATCATCTAACATTTGGTTTCGACTTCCACTTGCTAGAGGTGACTCATGGCAACATATATGTTTGCGACTGGGCAGACCTGGTTGCATGTACTGGGATGTTAAAATAAATGATCAGCACTTCAGGGATTTATGGGAGCTTCAGAAAGGGATCAATAATACACCATGGGGTTCAGGTGTACGAATTGCTAATACATCTGACATAGATTCACATATACATTATGATCCAGATGGTGTTGTTTTGAGTTATCAATCTGTTGAAGTAGATAGTATAAAGAAATTGGTGGCTGATATCCAAAGGCTTGCCAATGCAAGAACATTTGCCCTTGGCATGAGGAAATTGCTTGGCGTAAGAGCAGAGGAGAAGTCAGAAGAGCTAGTTACAAGTTCTGACACCAAAACACCCAGTACCAAGGTTGCTCTGGATACTGCAGACAAACTAACTGAACAGATGAGAAGGGCGTTTAGAATTGAGGCAGTTGGACTGATGAGCTTGTGGTTTAGCTTTGGTTCAGGTGTCCTTGCTCGTTTTGTTGTTGAATGGGAGTCTGGTAAAGAGGGTTGCACTATGCATGTATCTCCTGATCAACTTTGGCCTCATACCAAGGTTGTTAATTCCCCTCTTCAACTGATTTTTATTTGCTTTTCTTTTTCCATTGTGTTCGTTTTGGCATCAGTGATAGATGCATATTTTTCTGTTGTGCTTCAAAATTTTGATAATTATTGGAAACTAAAACAATATATGCATGCCATATTATCCAGTCATGATTGTTGGCTTTGATCTGTTTTCTTGGTTCATCATGTTCGTTATTTCCTCCATTGTAATGTTTTTGGACTTCCCCCCTTTTCATGAGAAATAATACACATGATCCTGCCAAGGTATTGTTCTCCTTTGACTTTAGCTGGTACATTGCATATTTACCCTGATTATTTATGCTATCTTTTGGGTGAAATTACTATGGAGCATCTTTCTTTTGCTTGTTCAGCCGACTTTGTAAAAACATAGACCTGAAAATGCATTGCATTAATTTAATTGTTATGGTTATTTCTGCTGATGTATGATGGTCCGTAATGCTTGATACCATTTTTTTCCTTTGATATTTCAGTGTTGATAAGAAACCAATTCCTTAATTTTAAGTTGTCTTTTGTATAGATGTATCTAATATTTTGTCCTGGATCTGTTCCCAAGATAAATTCCTCCCTATGTATTTACTAAATTAGATGCTTCTATTCTGTACAGTTTCTGGAAGATTTCATAAATGGAGGTGAAGTTTCACCGCTGTTGGATTGCATTCGGCTGACTGCAGGGCCCTTGCATGCTCTGGCAGCTGCAACCCGGCCAGCAAGAGCTGGTCCTGTTCCAGGGGTTGCAGCTGCCTTATCTTCTATTCCTAAACAGAATGGTAGTTATATATCCTCACACGGTCTTCTGCTTAGTAATTCAACTACTAATGTTGGTCTGCCAACATCTGGACCTGGGGCAAACACTGTCATGCCTACTGCTAGTGGTCTCACTAGCCAAACACTTTCAATGTTAGCTGCTTCTGGACGTGGTGGCCCAGGCATTGTTCCAAGTTCACTTTTGCCCATTGATGTTTCTGTTGTGCTCCGCGGTCCATATTGGATACGGATCATGTACAGGAAACAATTTGCTGTTGACATGCGTTGCTTTGCAGGAGATCAGGTGTGGTTGCAGCCTGCAACACCGCCTAAAGAGGGCCGTCTTTCTGGAGGTTCACTGCCTTGCCCCCAGTTTCGGCCTTTTATCATGGAACATGTTGCCCAGGAATTGAATGGATTGGATCCTAGTTTTACTGGACAACAGGCAGGTGGGATGGCAAATTCAAATAATCCAAACCCTGGTTCAGGATCCCAGATGATGGCTGCAAATGGAAATAGAATAAATTTGCCAATTTCTGCTGCAATGCCTAGGACAGGAAACCAAGTAGCTAGTTTAAACCGTGTTGGAAATGCTTTAGCAGGCTCTTCAAACTTAGCTTTGATGACATCAGCTGTCTCTCTACGGAGGCCACCTGGAACGGTTGTCCCTGCACATGTCAGAGGCGAGCTGAACACAGCCATTATTGGTCTTGGTGATGATGGAGGATATGGAGGTGGTTGGGTTCCTCTTGTTGCTCTTAAGAAGGTTTTGAGAGGTATTCTCAAGTATCTTGGAGTGTTATGGCTTTTTGCCCAGTTACCTGAACTTTTGAAAGAGATCCTAGGATCTATTTTGAAGGAAAATGAAGGCGCACTTTTGAATTTGGACCCTGAGCAGCCTGCCTTACGTTTTTTTGTTGGGTAAGTATCTTATTTCCCCCCCTTCTGTGGTACTTCACTTCTTATGTTTCTAGAATTCTTTTATTTAAGCACCACTCTGCTGTAATTTTTACATTCCTTCTTGATACATGGACTTTTGCTATATCTTTACCCTTCCTGCTTAAAGGGGAATAATTGCAGTTGTGATATTGGCAATTTTTACATTCCTTTTCTTATTCATCTACTAACTTTTTCAGCATGTTTCTTACTTCATTGTTTGTTTCATTTCTTTGTTCCCTTGGGTGATTTAGCCTGGAGAGTTCTTGCAACAATAATTTTCAATTATTGAATTTTGGATTAGGATGTTAAACAAAATGTCTTTAATCAATTTTTTTTTTAATTTCTTTATGAGCATGATACATCAAATTGATTTTTATATGTGAATGAATGTTATGCATATTGATACTTTATGACTTATTAGCTATAGTTGGCAAAAAGTAAGAAAAAAGTTTATTGCATTTTGATCTCCAGTGTTAGGAGCGTTCAAATTTAGCCCCCTTAATTTAATTTGGAATGTTATCTCCTGGGTTTATAAGTTGATGCAAAATTATGCTTCCCTGCCCATAATCTGTTCGAAGTGGCTATTTTTGCTAATGGGTCATTTTTGGCACATGGACTCTGCATCATCAAAACTAGAAACTGATTGGTACTTCCCTAGGGTTTCACTTTCTTTGTACCATCAACTGTACCATATCAGGTGTAGCTTTAATGTATTTAGAGTGGGAGACAGTGATGACTCACATAACCAAGGTGCTACACATTTAATATTACAAGAGTAAGAAATCAAATTTAAAGCCATGTGAGCAAAAGCAGTGTGACGAGTCTTGATTGCTTACATACAGAAACTACAGGGTAATGTAGCAATTGGAACTAGAGGAACCAAAGTCATTTTTGGTGAAACTAGGACTAAAATTGTAACTAAGCTTAAAAAAGGAAACAGCTGGTGTCTTCTCAGTCTCTTCTTAAATATTTGCCATTCTTATTCCAGTTATTAGACACACTAAACTGAAATGCTGTCTTTTCATCCTTCTGCAGAGGATATGTATTTGCTGTTAGTGTCCACAGAGTTCAGCTTCTTCTTCAGGTATTAAGTGTAAAACGCTTTCACCAACAGCAACAGCAACAGCAGCAAAATTCAAATCCCGCACCAGAGGAATTAAGTCAATCTGAAATAAGTGAAATATGCGACTACTTCAGCCGTCGAGTGGCATCAGAACCCTATGATGCTTCTCGTGTTGCATCATTCATAACTATGCTCACTTTACCTGTGGCAGTACTGAGAGAATTCTTGAAATTAATTGCTTGGAAAAAGGGATTATCGCAGGCACAAGTTGGAGATGTTGTTTCAGCTCAAAAGCCACGGATTGAGTTATGTCTTGAAAATCATTCTGGGTTGAATATGGATGAGAACTCTGAGAGCTCATCTGCATTCAGAAGCAACATCCATTATGATCGCCTGCATAATTCTGTTGATTTTGCGCTTACTGTTGTTCTTGATTCTGCTCACATCCCTCATGTCAATGCTGCTGGTGGTGCTGCATGGTTGCCATACTGTGTTTCGGTAAGATTGAGATATTCATTTGGTGAAAGTCCCAATGTATCATTTATTGGCATGAATGGTAGCCATGGTGGTAGGGCATGTTGGTTACGTGTTGATGATTGGGAAAAATGCAAACAGAGGGTGGCACGAACAGTGGAGGTTAATGGGAACTCAGCAGCAGATGTAAGTCAAGGTAGGCTGAAATTAATTGCAGATAGTGTGCAAAGAAATCTGCATATGTGCATTCAAGGGCTAAGAGATGGTAGTGGGGTCACAACCAGCTCTGGAGCTGCGTGATTTTGTTAATAGCAGCAATCATATCTATCTATCTATCTAGATATTTTGGGTTAGTGTTGTCAATATTGCGTTTTAGTTTACCATGATATGAAATGGTGCCATACTGAGTTTAAGCATGCCCCCACCATCCTAGCATGTCTCTTCTGCTCATAGGTTATGCTTATGCAGTTCTATTGGAGATGAGCTTTGCTGTAATGGAGAGAGTGCACAGGTGATTACAGACAATTTTGATTAAATTACTTTTGATAGCCTAGCTTAGATTTGCGGGCACCAATTTTAGATTACTAACCAAATATATAAAACGAGTATTCGACGTTTCTCTCAGATCATTAACTAGGTTGTACAATATCTTTGCTGATAGTGTAGTATTATGGTATAACTACTTGGCTGGTGGCTATTGTTATTGTACAGATGACATCAAATGAATAGATTAGTAAAGGAAATTTGTTACGCTCGATCTCTATCGACAATAGATAAAGACCTTGCTGCGTATTTAATTCTGAATTCATCTTGTTCTTGGCATGCTCTGTCTCAACCCCGGTTGCTTTTATGTTATCTGTAATAACCTCATTTTGGCAGCAGCTTCTTAATTGTGTAACGTGTATCGTTTTCTGATCATTGAAACAGCTGCAGGATTTATTGGT

>GmMED14-2

GATGAAACAAATCCCAGATTGGTGGGCGCCGTGAGAGTCAAAAGGCTTTAGAAGAAAGAATAAGGCATTTTTGTGTGTGTGAGAGAGAGAAAACCAAACGAAACTTGAGTGAGACTGTGTCAGAGAGAGAGATTCATTTCATTCTTGGTTGTCTTCTTCTTCAAATAACAATTCCTCTCTTCTGTTTCGCCCAAAACCCTAGTTTTCGATGCTGCTGCTGCTCTGACAGATCGGGAAGAACCCTAGCCAAACAAGCAACCAATGGCTTCGGAGTTAGGGCAACAGACGGTGGAGCTCTCCACTCTCGTCACACGCGCCGCCCACGATTCCTACGCCTCCCTCAAAGAACTCGTCGATAAATGCAAGTCCTCCGAATTATCCGACACCGACAAGAAGATTAGCATCCTCAAATTCCTCAGCAAGACTCAGCAGCGCATGATCCGCCTCAATGTTCTCTCCAAGTGGTGCCAACAGGCAATTCAATTCGCTCCCCGTTCCCTTCTTCAATTGCAATTCCATTATTTTTATTTAATTTCCTTTTGATTTATTCTTTTTTATTTTTTTGCAGGTTCCTTTAATACAACACTGTCAGCTACTGGCTTCAACTGTTTCGAATCACGACATGTGTTTCACACAAGCTGCGGATTCGTTGTTCTTTATGCACGAGGGGCTTCAACAGGCGCGTGCACCCGTTTACGATGTACCTTCCGCCATTGACATTCTTCTCACCGGAAGTTACCAGCGTTTGCCCAAATGTATAGAGGATGTGGGGACTCAGTATGCTTTGACCGAGGAACAGCAGAAACCTGCTTTGAAGAAGCTGGACACGCTTGTTCGGTCCAAGTTGCTTCAGGTTTCTATTCCCAAAGAGTTTTCTGATATTAAGGTTTCTGATGGTACCGCAATGCTTAGGGTAGATGGGGAGTTTAAGGTTTTGATAACACTTGGGTATAGAGGGCACTTGTCTTTGTGGAGGATTTTGCATTTGGAGCTGCTTGTTGGTGAGAAGAACAAGCCTGTGAAGTTGGAGGCAACGCGGCGTCACTTGCTTGGGGATGATTTGGAGAGGCGGATGGCAGCGGCGGAAAATCCATTTTCGGTGTTATACTCGGTTCTCCATGAGCTGTGTGTTGCACTTGTCATGGACACGGTCATAAGGCAAGTGCAGGTTCTTCGACAGGGAAGGTGGAAGGATGCAATTCGGTTTGAGCTCATATCCGAGGGGCATGGAGCGAGTTCTAGCTCTGCGCAGAACCCTGATGGGGAATCTGATTCGTCTGCCATGCGAACTCCAGGGTTGAAGATTGTGTACTGGTTGGATTTTGATAAGAATGCTGGTGCGTCCGAATCGGGCACATGCCCGTTTATCAAAATCGAACCTGGGTCAGATCTTCAGATAAAGTGTCTCCACAGCATCTTTGTCATAGATCCCTTGACGGCCAAGGATGCAGAGTTTGTTTTGGACCAGAGTTGTATCGATGTTGAGAGGTTGCTGTTGAGAGCTATTTGTTGCAACAGATATACCCGTCTATTGGAAATTAAAAGAGAGCTGGTGAAAAATGTTCAGGTCTGTCGAACCACGGATGATGTTGTGCTCCAGTCTCAAATGGGTGAACCTGATATTGAATATAAGCAGGTTGGTTTACATTTACTGGGAAAAGTTCTCTCTTATTGGGAGTTTGGAGTGTCAAATCGGTGTGTTGCTTTGGACTTTAGTAGGTTTGTAAGGGTTAAATTTGGATGATTATTTTGGGGTTTTATTTTATGTGATTGTTTGGTATCTGTCCTTTTGGGATTTGACTTTGCTTACCACATTCTTTTAGTTTCAGAAAATAGATTCTCAGATCCTGGATATTCTACTCTTTTTTTTTTTTTTTAAATCCTGGATTATCAAAGTACTACGCCCAATGTATACATTTGTTGCTGAGCAGTCAATTTTTGGCTAATTATGCTTTATGTAGGTTAAGATAGATAATTTTGAAGTTCCGGTCAAAATTATAGTCATCTTATTTCTGCATTATCTGCCAAAGATTTGGGGTTTACTATTGTTGAGATCCCACATCGACCAAAGATATGAGAAATGTAGTCCTTATAAGGCATCAACAGTCCTCAACCCCACATTGGTTTGTAAGATGAGGATTGCGCAAGACTAGTAAGGGTTACACTATCTCTAGTCAATATGAGATCTCAATATCCCTCTATGTTGATATGCACGTGTTTTCCATTGAAAGACTCTTCACCTTTACATCATACAAACTGTTGCAATTTTATATGTTCATCTTTGAATCTCAATACCTTCCCATGGATATTATGCATGTAATCTTATGCCTTACCTTCTTTCATTCAATCTTGCTTGGGGTTTTGAATTTGAGATCTTCTTCTGTCTGTAGAAAGATGAGAAGTGCCACAGCAAGGACTTTGAGGGCCATGAGGTGTTGCGTGTGCGTGCCTATGGCTCTTCTTTTTTCACACTTGGAATTAATATCAGGTACCTAACTTTACTTGCATTTTCCTTATCTATTGCATTTTAATTTGGCGGATTTTTTAAAAATCCATTCCTCTGTTTTGCTATTCAGGAATGGCCGTTTTCTTCTTCAATCTTCTCAAGACATAGTGGTTTCTTCCGCTTTATTAGAATGTGAAGAAGCTTTGAATCAGGGGTCTATGACTGCAGCGGAGGTCTTTATAAGCCTGAGAAGCAAAAGTATATTGCACTTATTTGCATCTGTTGGAAGGGTTTTAGGCCTTGAGGTATATTTACCTGTTTTCTCTCCTTTGGACTGTCTTCGAGCTTATTATTTGGATTACTGGCTTACCTAAGCGTCTAAGACCTATGGAATATGGGGCATTCTTGCAGGTATATGAGCATGGATTCAATACAGTTAAAATACCCAAGAATGTTTCGAATGGTTCAGCGATGCTATTGATGGGATTTCCAGACTGTGGAAGCTCTTACTTCCTGCTGATGCAACTTGATAAGGATTTCAAACCCTTGTTTAAGTTGTTAGAGACTCAGCCTGACCCATCTGGAAAAGATAATTTATCCGGTGACCTGAATCAGGTGCTGCGGATCAAGGAAATCAATATAGGACAGATGCAGGTTCAGGAAGATGAAATGAATTTAAGCCTGGTTGACTGGGGAAAATTACGTTCTGTTTTGCCCAGTGCTGTTGGTCCAAATCAAACATCTGGAGATGAGTTTTTTTCTGATGTTCACCTTGAGAATTCCATACAGATTGCAAAAGGTCATCCATCAGGTTTTTCATCACTTGTTGATGAAGTGTTTGGACTTGAGAAAGGGTCTTCAATGCCTCCATTCTCTGTTAAAAGTCTTCCATCATCTGTGAATACATCCCTCCCTTCTCAATATGGCTCTGTCCCTATGAATTTCCATAGTTTAAAGGCTGGAAGCCCTTCGCCGAAGTGGGAAGTAGGAATGCAGATGTCACAGGTTAGTAATGTAACAAAAGCTTCTGGTGCTACTAACCACTATAGTGTGAAGGGCCCACTTCAGTCCAGTTCTGTTGGCTCGATAACAACTGGACAAGGAAGGAACTCTGCTGGGAAAAAACTATCTGCTTCCAAGTCTGAACAGGATTTGGCTTCTCTTAAATCTCCGCATTCAGTTGACATTAGTTCCTCTTCTGCAATGGATGAAGAACAGCTAAGATTGTTGAGTGATACTTCCAACGATGCTTTATCTGGAAGTCGGTCATCTCGACTATTATCTCCTCCACGACCGACTGGCTCTCGAATGTCCATACCAAATTCCAGACCTAATGGACTTGAAGTTGAATCATTTAAGGCTGCTGGATCCAGTTCATGTGCTACAACTCCCGTATGTAAGATACTTTCTTTGCATCTTCATCTTTATCATGTGGAGTTATTCATGCTGTTATTTATTTTTTCTTTCTTTAAGTAATATATGACTGGAAAAATGAATGTTTGCCAGCCCAAACACTGGAATCAACAGTTAGTTATAACACGGGTGAAGATGTTACCTCCAAAAATGATAAGAAATCTAGAAAACGAACAGCTTCGGATATGTTGACTTTGATTCCATCTCTTCAAGGTGTTGAAAGCAACCCAGGAATCTGCAAGAGAAGAAAAATTTCTGATTCATCTGGCTGTCAACTATCTTTGCCACAAGGTGTCATGTCCGCTGAAATGATACCCAAAAAAGAAGGATACAGTTATGGAAGTTTAATCGCAGAAGTAAATAAAGGGAATGTTCCATCTAGCATTTACATTGCGGCTCTTCTTCATGTGGTCAGGCATTGTTCACTTTGCATTAAGCATGCTAGACTAACCAGCCAGATGGATGCATTAGACATCTCATATGTTGAAGAAGTGGGTTTGAGAAGCGGATCCTCTAACATTTGGTTTCGACTTCCACTTGCTAGAGGTGACTCATGGCAGCATATATGCTTGCGACTTGGAAGACCTGGTTGCATGTACTGGGATGTTAAAATAAATGATCAGCACTTCAGGGATTTATGGGAGCTTCAGAAAGGGAGCAATAATACGCCATGGGGTTCAGGTGTACGAATTGCTAATACATCTGACTTAGATTCACATATACATTATGATCCAGATGGTGTTGTTTTGAGTTATCAATCTGTTGAAGTAGATAGTATAAAGAAATTGGTGGCTGATATCCAAAGGCTTGCCAATGCAAGAACATTTGCCCTTGGCATGAGGAAGTTGCTTGGTGTAAGAGCAGAGGAGAAGTCAGAAGAGCTAGTTACAAGTTCTGACACCAAAACATCCAGTACCAAGGTTGCTCCGGATACTGCAGACAAACTAACTGAACAGATGAGAAGGGCATTTAGAATTGAGGCAGTTGGACTGATGAGCTTGTGGTTTAGCTTTGGTTCAAGTGTCCTTGCTCGTTTTGTTGTTGAATGGGAATCTGGTAAAGAGGGTTGCACTATGCATGTATCTCCTGATCAACTTTGGCCTCATACCAAGGTTTGTTAATTCCTCTTTGACTGATTTTTTTTCCCCATTGTGTTCATTTTGGCATCAGTAATAGATGCATATTTTTCTGTTGTGTTCATTTTGGCCTTGATCTGTTTTCTTGGTTCACCATGTTCGTTATTTCCTCCATTGTATTGTTCTTGGACTTAATACACATGATCCTGCTAAGGTATTGCTCTCCTTTGACTTTAGCTGGTACATTGCATATTTACCCTGATTATTTATGCTATCTTTTGGGTGAAATTACTGTGGAACATCTTTCTTTTGCTTGCTCAGCTGACTTTGTAAAACTTAGACTTGAAAAAGCATTGTGTTAATTTAATTGTTACGGGTATTTCTGCTGATGTATGACAGGTAATGCTTGATACCATTTTTTTCCTTTGATATTTCAGTGGTGATGAGAAACCAATTCCTTAATTTTAAGTTGTCATTTGTATAGATATATCTAATATTTTGTCCTGGATCTGTTCCCAAGATAAATTCCTGCCTATGTATTTACTAAATTTGATGTTTTTATTCTGCACAGTTTCTGGAAGATTTCATAAATGGAGGTGAAGTTTCACCGCTTTTGGATTGCATTCGACTGACTGCAGGACCCTTGCATGCTCTGGCAGCTGCAACCAGGCCAGCAAGAGCTGGTCCTGTTCCAGGGGTTGCAGCTGCCTTATCATCTATTCCTAAACAGACTGGTAGTTATATATCTTCACAGGGACTTCTGCTTGGTAATTCAACTACTAATGTTGGTCAGCCAACATCTGGACCTGGGGCAAACACTGTCATGCCTACTGCTAGTGGTCTCACTAGTCAAACCCTTTCAATGTTAGCTGCTTCTGGGCGTGGCGGCCCGGGCATTGTTCCTAGTTCACTTTTGCCCATTGATGTCTCTGTTGTGCTCCGTGGTCCATATTGGATACGAATCATGTACAGGAAACAGTTTGCTGTTGACATGCGTTGCTTTGCAGGAGATCAGGTGTGGTTGCAGCCTGCTACACCTCCTAAAGAGGGTCGACTTTCTGGAGGGTCACTGCCTTGCCCCCAGTTTCGGCCTTTTATCATGGAACATGTTGCCCAGGAACTGAATGGATTGGATCCTAGTTTCACTGGACAACAAGCAGGTGGGCTGGCAAATTCAAATAATCCAAACCCTGGTTCAGGATCCCAGATGATGGCTGCAAATGGAAATAGAATAAACCTGCCAATTTCTGCTGCAATGCCTAGGACAGGAAACCAAGTAGCTAGTTTAAACCGTGTGGGAAATGCTTTAGCAGGCTCTTCAAACTTAGCTTTGATGACATCAGCTGTCTCTCTTCGGAGGCCACCTGGAACAGTTGTCCCTGCACATGTCAGAGGTGAGTTGAATACAGCCATTATTGGTCTTGGTGATGATGGAGGATATGGGGGTGGTTGGGTTCCTCTTGTTGCTCTTAAGAAGGTTTTGAGAGGTATTCTAAAGTATCTTGGAGTGTTATGGCTTTTTGCCCAGTTACCTGAACTTTTGAAAGAGATCCTAGGATCTATTTTGAAGGAAAATGAAGGCACACTTTTGAATTTGGACCCTGAGCAGCCTGCCTTGCGTTTTTTTGTTGGGTAAGTACCTTGTCCCGCTCCCCCTTCTGTGTTACTTCACTTCTTTTGTTTCTAGAAATTTTTTATTTAAACACCACTGCTATAATTTTTGCATTCCTTCTTGATACATGGATTTTTGCTATATCTTTACCCTGCCACTTAAAGGGGAATAATTGCAGTTTTACTTCCTTGTTTGTTTCATTTCTTTGTTCCCTTGGGTGATTTAGCCTGGTGAGTTCATGTCTTTAATCAATTTCTTTTTTATTTTTTATTTCTTAATATGAGCATGATACATCAAATTGATTTTTATGTGAATGAATATTATGCATATTGATACCTTATGACTTTATTACCTATAGTTGGCAAAAAGGAAAAATAAAGTTGATTTCATTTTGATCTCTCCAGTGTTAGGAGAGTTCAAAATTAGCCCCCTTAATTTCAGTTGGAACATTATCTCCTAGGTTTAGAAGTTGATGCAAATTTATGTTCCCCTGCCCATAATCTGTTTCAAGTGGCTATTTTTGCTAATGGGTCATTTTGGCACATGGACTCGGCATTATCGAAACTAGAAACTGATTAGTGCTTCCCTAGGGTTTCCCTTTCTTTGTACCATCAACTGTACTGGCTCAGCCCTCAGGTGTAGCTTTATAAATGTAGTTAGAGTGGGAGACAGTGATGACCCACATAACTAAGGTGCTACACATTTTATATTACAAGAGTAAGAAGTCAAATTTAAAGCCATGTGAGCAAAAACTGTGTGATGGGGTCTTTGATTGCTTAGATACGAACTACAGGGTAATGTAGCAATAAGAGGAACCAAAGTCATATTTGGTGAAACTAGGATAAAAATTGTAACTAAGCTTAAAAAAGGAAACAGCTAGTGTCTTCTCAGGCTCTTCTTAAATATTTGCTATTCTTATTCCAGTTATTAGACACACTAAGCTGAAATGCTGTCTTTTCATTCTTCTGCAGAGGATATGTATTTGCTGTTAGTGTCCACAGAGTTCAGCTTCTTCTTCAGGTATTAAGTGTAAAACGCTTTCACCATCAGCAACAGCAACAACAGCAAAATTCAAATCCTGCACCAGAGGAATTAAGTCAATCTGAAATAAGTGAAATATGTGACTACTTCAGCCGTCGGGTGGCTTCAGAACCCTATGATGCTTCTCGTGTTGCATCATTCATAACTATGCTTACTTTACCTGTATCAGTACTGAGAGAATTCTTGAAATTAATTGCTTGGAAAAAGGGATTATCACAGGCCCAAGTTGGAGATGTTGTTTCAGCTCAAAAGCCACGGATTGAATTATGTCTTGAAAATCATTCTGGGTTGAATGTGGATGAGAACTCTGAGAACTCATCTGCATTCAGGAGCAACATCCATTATGACCGCCTGCATAATTCTGTTGATTTTGCGCTTACTGTTGTTCTTGATTCTGCTCACATCCCTCATGTCAATGCTGCTGGTGGTGCCGCATGGTTGCCATACTGTGTTTCAGTAAGATTGAGATATTCATTTGGTGAAAGTACCAATGTATCATTTATTGGCATGAATGGTAGCCATGGTGGTAGGGCATGTTGGTTGCGTGTTGATGATTGGGAGAAATGCAAACAGAGGGTGGCACGAACGGTGGAGGTTAATGGGAACTCAGCCGCAGATGTAAGTCAAGGTAGGTTGAAATTAGTTGCAGATAGTGTGCAAAGAAATCTGCATATGTGCATTCAAGGGCTAAGAGATGGTAGTGGGGTCACGGCCAGCTCTGGAGCAACGTGATTTTGTTAATAGCACAAATCATATCCATCTATCTCTTTATTTTGGGTTAGTGCTGTCAATATTGCGTTTTAGTTTACCATGATATGAAATGGTGCCTCACTCAGTTTAAGCACGTCCCCAACATCCCAGCATGTCTCTTCTGCTCGTAGGTTATGCTTATGCAGTTCTATTGGAGATGAGCTTTGCTGTAATGGAAGAAGTGCACAGGTGATTACAGATAATTTTGATGAAAAAAAATACTTTTCATAGCCAAGCTTAGATTTGCGGGCACCAATTTTAGATTACTAACCAAATATATAAAACGAGTATTCAACGTTTCTATCTTCAGATCATTAACTAGGTTGTACAATATCTTTGCTGATAGTGTAGTATCATGGTATAACTTCTTGGGTGGTGGCTATTGTTATTGTACAGATGACATCAAATGAATAGATTAGTAAAGGAAATTTGTTACGCTCGATCTCCATCGACAATGAACACTTGCAAGATAAAGACCTTGCCGTGTATTTAATTTTGAATTCATCTTGTCTTTGGCATGCTGTCTCAACCCCGGTTGCTTTAATGTAATCTGTAATAACCCCATTTGCCAGCTGCTCCTTAATTGT

>GmMED14-3

AAACGGTGTCGTAGATGGGCATTTTCTTCCGTGTCTGGCAATCCTCGTTCGTTTGCGTTGGCGGAAAATAGAAAAGAGAGATCCTGAATTTTTCCCGCGCCATTTTCCTTCGATCCAAACAATGGCTCCAAAATCCGATAGCGCCGAAGGTACCCTCACCACTCTCTCTCTCTCTCTCTCTCTCTCTCTCATTTCGCTGTTTCTCTTTCAGAACCTAACTAACCTAACATGTCTCTCTTTCATTTCGCAGCGATCGTGTTGAACTTCGTCAACGAGGTAACTAGCAACTAACCCTTTTCCCAATCACGTGCCTCACGACTCACCTCCCGTGACACACCTTTGCTCCCTTTGCCATCAATTATCATGCATGCTAACTTCAATTCTGTACCACACCAGCAACAAAATTGAAATTAAACTTATAAATTCAAAACGCCCCAATTCTGAGTTGAATGGTTGTTTGTTTCAGTTTTGATTTTGAATTTTTTTTTTTCCAGCAAAATAGGCCGGTGAATGCGCAGAACGTGGCGGATGCATTGCAAAAGTTCAATCTTAAGAAAGCGGCGATTCAGAAAGCGTTGGACATACTCGCCGATGGAAAGCGGATTTCGTTCAAGGAGTTTGGGAAGCAGAAGATATACCTTGCGCGGCAAGACCAGTTTCAAATTCCCAACACTGAAGAGCTGAATCACATGAAGAAACAGAATGCTGACCTTCAAAAGCAGCTAGAGGATCAGAAAAAGGCTACTAGTGAGGTTGAAGCAGGTATATTATCAAACTTAAACAGAATTTGAGATTTGAGAAGTCTGTTGAAACGATTCCGATTAACTGACTGTGTGAGTGCGTGAAATTGCTTATAATAGCAAATTCAGATGCAATCTGGACGCTTAGGAAGTAATATTATGAGGTAGAATGATAAGGGTTTGTTTGACTAAAAAGAACATTTTCAGTTATAATTTTTGGTTTATACACGTAAAATGATGTAATTCCATTTTGATTGAGTGGCTGATCGTTGTCTAATGGTTTCAATTTGCTAATTGTACCGGCTATACGAATTCTTGACTATACCTAATCCAGGTCATTTGATGTGGTTATTGTGGATATTAGCTTTCGTGCATTAGGAACCGTTGAGAATTTTTATTGTGGACAAACCTCTTGATCTTGCTATTTATGGATGCAACTTGTTGGTGCAGAAATTAAGTCATTGCAATCAAATTTGACACTGGAACAGATCTTTGAAAGAGAAGTAAATCTTAGATTGGAGGTAACTGATGTGGATGCTTGTGTGCTTGTTTTAATTTCATGAAAGGGAATTGGTGGTTGTGCTGAGATAATTTGCGTCTTTATGTTCTGCTTCAGGTTCAAGAAATGGAGGATAAATTGACCAAGTTACGTGGAGGTGTTACTCTGGTGAAGCCAGAGGAACGCAAGGCTGTTGAGAATATGTTGTCAGAGATGATAGGTCAGTGGAGAAAGCGGAAAAGAATGTTCAAGGATTTATGGGATACCCTCACTGAGAACTCACCTAAGGATCCCAAAGAATTTCAGGTTTCTTTCTTGGCTTGTCACAACCATAGATCTTATTTAGTTATTTTTAATGTTTCTGTTATCATATTGACACTAGTTTGCACTTTGCAGGAGGAGCTTGGAATAGAATATGATGAAGAAGATGTTGGGGTGAGTTTGCAGTCATACAATGACCTGATTCAACAGGGTCAGAAGAGGCCTAGGGGGCAGTGATGTATTTATTGGAATCAACAAATTAGGAGTACAGATTGTTTGGTGTTCTGACCTCAGTGAAAATTACTGCAAATTACATATAGCCTTATGTATTAAGTTTTTACAATTCTGACTTAGTTTGGGTTTATTCCCTAAATTTACGAGCCTGGCTGGAGCATAGTCTCAATGCAATATTATTGTTAATATCTCTTTGGGCACAGGCATTGGTCCTCTTTGCATTGCATACTATTATAGTGCTCATGTTTCAAGGACACAAAACATTATTGTAATTCTTGAACTTCGTATCTCCTGAAGAATGATTATTGACATAAACAGATTCTAG

>GmMED15-1

TATTCTTCTTCACGGTAACGGCCGGGCAAATGAATTGAAATTACTTTAGAATTAATTTAATTATTTCCTTAACATTATAAGAATCAGACTCATCGATAATATCTATCCAGAGATAATTTTGGACAATTGCCTAGGTTTGATTTCGCTAAGAGCAGTGTTAATCTCTGAACCCTGAAGGTTAGCATTGGACAAGCCCTCTCTAATAAATATTTAGATTCTGCTATTTGAGTGTATTTAGTGTGAAGATTGATTTCACAATTAACTTTGGGACGTGAATTTGGCGGAGAAAGCCTCACTACTCTGAAAGCTCCGATTCGGAGCAGGCTTCGCCGGACGAGTCCTCGCTCTCGCCTTCGCCCGTCGTCGATCATCGCTTATGACGGTACCGTCTCTCTGTTTTCGTGTTTTTAATTTCTCTCGTTTCTGTCTCTGGTTTATTCGATTCGATTCTGTGAATTAATAGAACTCTCTCTCTTTGATTATCGCTGATTTTGATTGAAGGCCTCGCTCTGTGTAAGGTTTTTGTATCTGAAACTCGATATTTTTCGACGGAAATTATAGTGTTTGTGAATGATGATGGAATCGAAATTTCTGACCAGTGTTGAATTGCCTGATGCATGTGTTTTTGAAAATAAACGGAGAATCTGCATGGAAATGGCATTGAGTGTGATAGATTATGAGGAAGAAGCCGAGGTTTGATGAATTGATTAAGGAAAAAAATAAAGAAAATTGGAGTAGAGGAGAACAGATAGAAGTTAGGCCTTTTGTTGTTTGTTCTAGAGTTTAGTTACTTGAGATGATAACAACTTTATTATTATAATATAACATGAAGAATCAACAAATTTTGACAGTTAGTTTTTAACTGAGTCAATTTGATCTTTGAAGGGATTTTTTTTCCTGATGAAAATCAATATTTGCCATGAGAAATGAGATACCGTTGTGCTATTCTTGGTTTTTGAGTTGTCTTTGTATCAATTTTAAGCTCTAGGTTTTCAGCCTAGTTTGTAAGTTGTAACTGGTGATGCAATTGTTTCCACACCGTACATGAATCATCCGTCAACTCTACACATGGTCACTTCAACAACTTTAATCAGTTCTGTTAGGAATAAAATTTATTACAGCTTTAATGTTTGGTGCGGTATAGATAATTTATAGATATTCCTGTTTTGAGGGGAAACCATAGCGCAGCAGTAATGTTGCCCTGTGTGACTGGTCACAGGTTCGAATCCAGAAACTACTTCTTTGTATATGTCAGTGAAAAGCTGTGTACAATGACCCTCCCCATACCTTCTCAAAGTAAGGAGCCTTTAAGGGTACTGGGGTACATTAGTTAGAAGTATTCCTGTTATATATTATAGGGTCCTATGACTTGCTGTCTCTTTGGCATTTCCAATATGGATAAGAAGGTCTCGTCTCTTATTTCAACTGATTTGTATTTTAGTTAAGTTTTTCTTTCTTAGTCTTCATTTTGTCTCTTATTTCTACCGATTTGTATTTTAATTAAGTTTTTCTTTCGTAGTCTTCATATGAAGTGTCTTTCTTGCTAAGCTTCCTTGGAAAATTCAGATCCCTTGAATAGTCAAGTCTTTTGTTTGGACAGTGATTCTTAATAAAAAAATAGTACTGGTGATAACTACAGCTTTATAGACCAAAGAGTTGTGTGTCTGTTTTGTGTGGTTCTAGTTCAGAAAGAGCTGCCCACTTATTTTTATATCATACTGATGCCAGGTTAGTTTGCTGTGTTCAGGAAATATTGAGGTTGGGCACATTTGGACAAACAATTTAATTAAGCGCTTATTCAATAACTTTATTTTTATAAGCCTAATTGATAAGTTTTACCGTGAGTGAAACGTATTGAAATAAGGTAAAGAATTAATAGGAAGATTATAAGCCTCTTTTGTACACTTGACCTAATGATAAAATAAGTGCTAATAAGTTCCATGGAAACTCTTCCAAATGTGCCCTATGTCTTTTGAATCTATAGCAGTTCTTATTGATTAACTTTAGAGGTTTTGGAAATTGGAAGGAGCAAGGATGCCAAAACTTTATTCTTGTAGTTCATTTGTTTGCTCATAGTTGTTACTAGCAAGCCATAGCCACAATGGAGATATGATGTCTCTTGTATGTATACGTGCCCCCTTTATATCCGATTTGATTTGAACATTCTTGTATAGGTGCTTTAGGTAATTTGAATTTGATGGATTCCAATAATTGGAGACCCAATCAAGGTACTAACCCCACTATGGATACAAGTGATTGGAGAGCTCAACTACCAACTGATTCACGCCAAAGAATTGTCAACAAAATGTAAGCTTCAATTTCAATACTGTATCCTTGAGGTGATTTGAATAGTTCTTTTACCAGTTGTTGCTTATCAAAAATTAAGTTAATAAATGAATATTTAATCCTAGTTGTTCGATTTCATCAACTTTTTACAGTTAATCATGTTGTGTCTTTTAAGGATTGGCCGATATTATTAACATCTCCATCCTTGCATTATCTAATTATGAAACTGTTGTTTTTCATTTTGTAGAATGGATACATTAAAAAAACATCTTCCTGCTTCTGGTCCTGATGGATTGCATGAACTTCGGAAGATTGCTCAAAGGTTTGAAGACAAGATTTATACTGCTGCAACAAGCCAGGTACTTTCATTGTTAAAATTGAGGAGCCTCCATTTCATTTATTGTTACCTTTGCAAATATATACAAGATTGATGGTTTGACACAGCATTGGTGTTCTCTCTTTTCAGCCTGATTACCTACGGAAAATATCTTTGAAGATGCTTACAATGGAGACTAAATCCCAAAACACCTTGGCTAACAATATGCCACCAAATCAAGTTGGCCCTAGCAATAAACCTCCTGATCAAGGTTAGCATTTAATAGCCTTTGGTTTGGTGATTATATTCTGATGTGGTACCAAAGTGTCAAATCCTAATTATTTATGTATAAGTATTGGGAACAGTGCATTCAGCTCTCCTAAAGTTTGGGGCATTTGCAAAGTACTACTACAATCTTTAACGATGTCTCAATCAATTATCCCCTAAACTTCAGTCATTGGTCTAATAGATTCTTTTGTTTATCCTTTATCAAATCCTTAACAGAGGTGACATGTGGCAAATTTGAAGCCCCCCATGTCTATGTAATTTGTGCCCTTTATTAATGTATTTAAATTCTATTTTTCTAACTTGCAAAAATTACAACATACAAAGTTTGATAGCAAGATCTAGACCAATATGGTCTGGTCCTCAATTTGTTTGACTTGCTGGAGGATCTCCATCTTCTTGTCAATGCTTCTCCACTTCACAAAGAGAGTAAAATCCTCTTTCTCTTTCTATAACCATACACCACCTAGCAGAGCGTTCCAACTCAAGCATACCAGAAAGTGACTACAGTTTGCATGCAATGCCACCTTGTTTCGCCGAAGAATGTACCAGCAAATACCAATGATCACACAAGAAGCTAAAAATGCAAGTAATTATAACAAAATTTTGCTACAACATGATGTTTTAGGTTTGACAAATTGTAGGGATTCAAGGCATGTAGATAAAACTTGACAAAAAAAAGTAAAAATTAACTTTAATTTGACAATTTGGATCGATCAGTGTGGAAGATGTTCTCATTATAGTTATTAATTTATTATTTATTTGTTTTCAAATTTTGTACTACTTTTAGTTATGGAAATTATTGAAATTAGATCAAATTTTTTTATTTTTTTTTTATTAAATGGAACTTACAAGTAGTTATCTATTGAAGTAGTAGTTAAAGTTTAAGAACTAATTGGGATGACTTTAAATTTCAAGGTGTCACTTTAGAAATGCCCCAAAGTTCAAAGGAGCCAATTACTCTTTAGCCTTATATATTTGATTAAGTTTTGTCATTCTGTTCTTTTAGTAATTATTCTGATTCTCTGAACTGTTCGTGTTTTTAAGCTGATGCTATTGAGAGTATATAATATCCTTTTGCTTGAAATATATTAAGTGAATCAAGTTTTGACTTTATTGGTCATTCATATTATCCTTTCCATTTGTCTCCTGATCCTTTGTCTTTTGAATTGTTGTGTTGACCTTTTCCGTATATAAAATCCTAACATATTGATGGTATACTGTAATCAATATTCTGGTTACTTCTCAGTTCTCACCCAGGCAAATAAATTTTTTCATAGGGCTTGTTTTGCAGTCTCAAGTTCATAATCTTGGGCAACAGCATTCTATTCCTTTGTCTAGTCAGCTCCAGCCACATCAGCAGCTTCTATCTCAAAATGTTCAAAACAATGTTGCATCTCAACCTAACCTTCCACCTGTATCTAGTCTAGCCCAGACTACCAGCCAAAATATTGTCCAGAACTCCAACATGCAGAATATAACTGGGCCAAATTCAGTTGGGAGCACTATTAGCCAAAATTCCAACTTACAGAATATGTTTCCTGGTTCTCAGAGACAAATGCCAGGAAGGCAGCAGATTGTTCCCCCACAGAATTCACCGCAATATCTTTACCAACAGCAGCGTTTGAAGCAGAAGCAGCTTCAATCTCAGATGCAGCAACAACAACAAAACCTTCTGCAACCAAATCAGTTGCAATCTTCTCAGCAATCCATTATGCAAACTTCATCTGTTATGCAGCCATCCATGATGCAAACATCTTCTCTATCTAGCATTCCACAGAATCAACAGTCTACTAATGTTCAACAGTCAATGCAGTCCATGCCACAGCAGCACTCACAAGTTATCAGGCATCAGCAACAACAGACTTCTATTGTACATCAGCAACAAATACCAGTCACTCAACAGCCAAGCTTACCTGCACAACAGCAACAACAACTTATGGGGCATCAGCCAAATACAACAAACATGCAACATGCACAGATGCTTGGGCAACAGAACAATGTTGGTGATATACAGCAACCACAGAGGATGCTCTCACAACAGAGTAATCTTACAAACCTGCAACAGAGACAACAGTTAATAAATCAGCAAAACAACCCAGCAAATATACATCAACAATTGGGAAATAATGGCCCTGGGTTACAGCAGCAACATTTGCTTGGACATGAATCTGGCAATGCTGACATGCAAACAAGTCATCACTCTGCACACATGTTGCAACAACCAAAGGTTCCAATGCAGCAACAATCACAACAAAGTACATCAAATTTGTTGTTACCTCATTCACAGCAGTCACAACCACTGGGTTCACAACAGCAATTAATGCCACAGATTCACACTCAGTCTGCACAGTTGCAACAGCAATTGGGTTTGCCGCAGCAGCTAAATCCCTCACAAAGAGATATGCAGCAAAGAATTCAAGCGTCAGGGTCCTTACTTCAACAACAAAATGTTCTTGATCAACAGAAGCAGTTGTATCAGACTCAAAGAACTCTTCCAGAAACATCAGCAAGTATGTGCTTGCATTTATGTGTTTCTCTGTCATTATATTGTTTGGGGAGTCAATAGTAGCTTTATTAGCAAATGCAATTACTCTTCCACATTTTTTTAGCATTGTATGTTCATGCATGTTTCATATCGGACCTATTGTAGCTTCTCTGGATTCTACAACACAGACTGCACAACTAAGTGGGGCTGATTGGCAAGAAGAAGTATACCAGAAGGTACAATGCCATTTTTATTATCACTTTTATTTCCTCACTGAAGCTTTGCTAGAACAACAGAAATTAGTCAAGCAAACCATAATGTAAAATAAAAAGTTATATGATGGTTTTTATTGTGAGAATTTTTAATTCATTGTAAAATCATGAACCCTTCTCTAGTCTTGGTGGAAAAATCACTTTTTTTAAAAAAAATATCCATATTTCTGAAATCTTCTATGCATTTTGTGCAATGTTTAAATGAGTTATATGGAAAGATGTGAACTACAAGAATACATACTCTTGCTGTTTGACAACAACAAATTCAATGTTGTAGGTTTGCATAATTAGCATAGGGTTTTGATCCCATTATTTCTTTTTATTTGCAGAGATGTTTCCCTAAATTCTTTAATTAGGATAGATTAGGACAGATTTGTTTCCCTTTAAATCCTATCTATGTTAAATATTCTATATATACTATAATTAGTAGTTGTACTGTAAGTCTATAATCAATTAATATTCAGATATTTTCTCTTTCAAGTTTGTATAAGAGCTTTGAATCTTGGAACTGGGAGACACTATTTCCTCATCCCAGAACCCTAGAATGCATTCATTGAAGCTGCCTTCTTAACTGCCACATCTTCAAGATTTTTTTTGCTCTCGCTGCCTTTTTTTTCTCCCAATGATTCCATTGAGCATAACTGCCACTTTTTGCTGCCAAAGTTGCTCTGATCAAATCGAAATCGCCATTAGAAGATCTACTAAGCATTTTTGGGTCAGATCTAGAAGCACCACTGCCCAACATACTCCCATGTGCCCTCACACACTTTCTCTGCACAGGATTATTTTCTGCCTGCCATTAGTCCTATTCAACTTGCCTGGGCTCTCTACATGGATCTGACCTTACCTTGTCTAACCGATTCTCATGGGTCCACCCTTCTTCCAAATAGCTGTAACTTTTCAACTTGGAGTTAGTGTTTAACCTTTTTTGTATGTAGAGCTATCAAACTAATGATATCATGTCTGACAAATCTCTCAAAAACTCTCAGTTACCCACTTACCCTTATGGTAACCTTTTTTTCTTTGTCCATCACTGGTAATGGCTTTTTTCTCAATCCTTTAATGCTTTCAATACTATTTCAAACAATTCTTAACTTCGGTGCCAGTTTGCCACCATGTATGACCTAAAACTTGTCCCATTTTTTATTAACCTTTTTTTAGTAATCATACCACAATTGTGGCTAAAAGCACCACTCCTCAATCACTGGTTGTGCACACATTAAAATCCAAACTTCCCTATTTCTAGATAATGTCCTACATGTTTCTCAACTCTCCAATAATCATATTTCCATTCACAAACTTACTAGATATTTAAAATGTGAAGTAACCCATCATTTCCACTGAAGGTGGTACCAATTATAAGTTTTCTACTTCCTATCATCAAATGAGCTAAAAGACTGATGCTTTTCAAATTAGGCTCCAACATAAGCCTCGTGGGCATCCTCCATTTCTTAAGTCGTTGTTTCTATCTTTGTTCTCAAAAGAATTTGTCAAGTCCTTTTAATGTGATGATGTTTGTTAGTTTGCTAAACATCATTGATCATTTTTTCTCCCAATATTGATAAAGTACTAAATCTTTTGAAATGATTCGTTCTAATGTGCGGAAAAGCAGAATGCAAAATGTTGTTGCAAGGTTGAATGCTGAGGCAAAGTTTAAGCTATGGCACAAAGGATTTGTGTGCTATCATGGATAAAGATCATAGTTGATGACCTTAAAATATGAAACTCCTATAAATTTATTTGGCAATAACAGGCCAGCCATTAGTAATTTAGTATTGCACACAACTTTATGATAGGATAAAGCACACAAAGATAGACTATTCATCAAGGAGTTATAGACCAAGAAACTTCTTACAAAGCAATTCCAATAACTCACTAGCAAATTAGGAATGATAGATATCCCATTCACTAGCTTGAGGGGAAGTGTTGTGTTGCATAATTAGCTCATTTATTGTTTCCTTGTAAACCAGATTAGCTTTATTAGAATTAGTCTATACATATTGTAAATTGCAGCAGTACCATAATTCAGAAATAATACTTAGATATTTTCTCTTTCAAGTTTTAGTTTTATGTCGAGTGAAATTTCTTTTGTCACTACCCTTTTTATCCTCTTTTAGTTTCTGCCAGCCTGCCATTAAGAAAGACTCTTTGAAATTATTCATCTGATGCTATATTGATTTGAACTAGTGCAAGTTGGTGTTATTTTTTAGAGATCTTGATTTAAGCACAAATGTGAAACTTGTGATTTATGAGGCCCTGCCAAAGGGGGGAAAAGAAACTTCTAGTTATCATCTTCGTTGGTGTCAATGCAATAGTGATATAGATGTTTTTACATGAGAGATCTCCAAGTTACTGATATTTATTTGATTTCATTCTGCTGATTTCAACCTAGACATCCAGACCATGCAAAATCTCAAGGTTACTAATGTTTTGATTATTTGATTTGCTGATTTCACTCTACAGCTCCAAACCATGAAAGAAAGTTACTTACCAGAGATGAATGAAATGTATCAAAAAATTGCTAATAAACTTCATCAGGTATATTTACAATTATTTTTTTACTTGTCAGTTTTCTGTCTGTTATGTGCAAAAAATGTTTTCCCAGCTAAAAGTATTGAGTTCAATAGTAGGCTTTGTTGATAGTTTTTTTCATGTTGTATAAAAGGAAGTTTGCTGTAGAGATAATTGATGTTGATGATTCTATATATGGGATCCATAAAATATATTTCTATTTACATTTCAAAAGTTATTCTACTTTACCATCATCCAAATAAAATCAGTTCTTTTAAACTCATCAATATTTTTTTTAATCCTGATTTTTCCGAAATCAATTTTACCAAATCTTAAAGAAATATACCCTTTAACAGATGCTACTGATAAAGCATCTTTTTTCCATAGTAAGTGTCATGGAAAATACTAGAATTTGCAGCCTGTGTGAAGCGCAGTAAGTACGATATGTATTGCTTATAATTTATTTATTAGTGATGCATGAATTAGAATGAAAAATATCAACTACTATGCAAAAGTATTTAATATCATGGGAATCCTAAAAGCATTTAATATTTATTTTCCGTAATCAGCTGGATCTATTCATAGATAAATATTACCAATGGGTACTGAATTCTTCTAAAATCTTAATCAGTGGCTTCTAGGGCACTTTAGCAAATGTCATAATTTATTTGTTTTTTCTTTTCCTCTATTTAATCCTACATCTGCTTTTCTCAATCAGCATGATTCTCTTCCCCAACAACCAAAGTTAGATCAGATTGATAAGCTGAGGGCATATAAATCGATGTTGGAGCGTATGATGGCACTCCTACAGATTCCCAAGAACAACATTTTGCCTAATTTCAAGGAAAAACTGGGATCATACGAGAAGCAGATTATAAATCTTCTCATAAATTCAAATAGACCCAGGAAAGGCATGAATTCAGTACAGGCAGGACATCATCCCCCAACTCACATGTCTTCCATGCAACAGCCACAATCCCAAGTTACTCAAGTACATTCTCATGAAAATCAAATGAACTCTCAGTTGCAGTCAACAAATTTACAGGGTTCTGTACCAACCATGCAGCAGAATAATATTGCAAGCATGCAGCACAATTCTTTGTCTGGTGTATCAACAGGACAACAGAATATGATGAATTCAATGCAACCTGGAACTAATTTAGATTCTGTTCATGGAAATTCTGTGAACTCCCTTCAACAGATTCCAATGAACTCCCTCCAACAAAACCCTGTTAGTACAGCTCAACAGACTAACATTAATTCCTTACCATCGCAAGGTGGGGCAAATGTGATTCAACCAAATGCCCTTCAGTCAGGTTCCAGCGCACTTCAACACCAACTGAAACATCAGCAGGAACAGCAGATGCTGCAGAGTCAACAACTCAAACAACAATATCAGCGACAACAACTGATGCAGAGGCAGCTCCTGCAGCAGCAGCAGCAACTACACCATCCAGGAAAGCCACAGCTATCTTCGCAATTGCAGACACATCAAATGCCTCAACTTCACCAAATGAATGATATAAATGATATAAAAATGCGACAAGGAATGGGTGTTAAGTCAGGTGTCTTTCAGCAGCATCTTACATCAGGTCAACACTCAACATATTCCCATCAGCAGCTGAAACAAGGGAGTGCATTTCCTGTTTCTTCACCCCAACTCCTTCAGGCTGCCTCTCCTCAGATTCAGCAACACTCATCTCCCCAGGTTGACCAACAAAATCATCTCCCATCTAAAACAAAAGTCACAACTCCTCTGCAATCTTCTAATTCACCCTTTGTGGGACCTACCCCTTCACCTCCCCTGGCTCCATCTCCTATGCCAGGAGAGTCTGAGAAGTCTATTCCTTGTGTTTCATCAATATCAAATGCTGCAAATATTGGACTTCAACAAACAGGGGGTGCAGTAGCACCAGCTCAATCCCTTGCCATTGGGACTCCTGGGATATCAGCCTCTCCTTTATTGGCAGAATTCAGTTGTCCTGATGGTGCGCATGGTAATGCTTTAGCTGCCACTTCTGGGAAGTCAACTGTTACAGAGCAGCCTCTTGAACGCCTAATTAATGCGGTCAGTATAGAAGTTTATCTGATGCTAGCTTGATCAGTTGTTTACCTGCATTTTATCTGGATTTGACAAATTAAAGAGTGAAAAGGTCCCCTTAATTATTCTATTGCTATATGCTTGGAATTGTAGTTGACAAATTTATGCCTAAATTATGATTCTAGGTGAAATCAATTTCGCGTAAAGCATTAAGTGCTGCGGTCATGGATATTGGTTCAGTTGTCAGCATGAATGATAGAATAGCAGGATCAGCTCCAGGTAATGGATCCAGAGCAGCTGTTGGTGAGGATTTGGTTTCCATGACTAATTGTCGTCTACAGGCTAGAAATTTCATTGCACAAGATGGTTCCAATGGAATTAAGAGGATGAAACGTTACACCAGTGCTATTCCCTTGAATGTTGTGTCATCTCCTGGTAGCATGAATGATAGTATCAAGCAGTTAACTGCTTCAGAGACTTCTGATCTGGAGTCAACTGCAACATCCAGTGTCAAGAAACCAAAGATTGAGGTAATAAATTATATGTTTTTAACCGTTATATTTTATTTTGTAATTGACATCTTTTACTGTCAATTATCTTTGTTCCTTGACTAGACAGTGTGACTTAGAAATAGGTCTGTACACATGGATAATAGATGCGTTGACATGTTATCTACATTACTTCTGCACATTTTTTATTTAATATACTACCTTGCCCAACTGAAAGTTCCAAGTAAGTGATTTTACATTTTCTCACTACAAAAACCTGAAGTAAAAAAAAAAGTCAATTGAATTTAGTAGAGTGTACAAAGTTGCAATTTCATTTTTCTCATTTAGACAGTGGACACAGAACTTGGAATTCATTTAGAAGTTGGAATTTGTTATTTCTAATGGTTTCACTCGAATTTTTGTCTTTCACTCTACCCATTCATTTGATCCATTTTTCTGCTAGAGCCTCTATCAGTCTTTTACCCCATTACAGAACCATCTTAGGCTAGTTTGTCATTTTCTCCTCAGTAGATGTTACTCCAACTTTCTCCCAAAACATGCACTCCTAGTCCTAATCTTACCAAATGTTACTAAATGATGTAGAAATTACTAATTAGTAAAATAACAATAATAACGTCCAACTAAATGATGTAGAAATTACTAATTAGTCAAATAACAAGTATAACATCCAATCAAGATAAACAAATATAGTAATTGTATGACACAAATAAGTTTAGTGGTTAGCCCAATGCTTGTACAGCAATAGTTTGTCTCATGTTTGCTTAGGCCATTATCCACCTCATTTACCTATTGTTGGTGTGAGTGACTCATGTATATTATTGAAGTTGTTGATGTAGCACATGGGCATTCCATTACATTCATCATATGATGATGATTTGGATACCTATTAAAATCTGTTTGTGTTTCATTAGAGCATGATGGTTAATCAGATAATAAGTTTTAAAAGACTTACAAAGTTATTATTGTTAGGAAACTACATATAATTTCTGGATTTTCTCTAGTTAATGTGTAACTTATCTGCTGTATTGGTCTTATGATTTCTGATATGGAAGAGTTATATTTTGGTAGGTTAATCATGCCCTTTTGGAAGAAATAAGGGAGATTAATCACCGACTTATTGACACAGTAGTAGACATAAGCAATGAAGATGTTGATCCAACAGCTGCTGTTGCTGCTGCTGAAGGGGCAGAAGGGATTATTGTCAAATGCTCTTTCATTGCTGTGGCTCTCAGCCCTAGCTTAAAATCCCAATATGCTTCATCGCAGATGGTGGATTCCCACTTCTGAGATCTAGCAATTACTACAATTTTAAGTCTTCTTATTTTATTTTCTTTATTTATTTGCTTTGTTAAGCCTTATTTGTGTTTTCTTACTGTACAGTCACCTATTCAGCCCCTGCATTTGCTGGTTCCTGCAAATTATCCTAATTGTTCTCCCATACTCCTAGACAAATTTCCAGTTGAATCCAGGTGAGATTATGAGCAACATTTTGGTACTTTGTTTTACAACTTTTTGAAGGCAATCATTTCAATGGTCACAAAAACTTAGGGACTATATGCTATCCATAGATCAAATCAGAGTTGTTCATTTCACATTATTTAATGAAATAAATATTTTATAATATAAAATGGATAAGTATGGACCGTCCTTGGTCCCTAAATTTTGGGAGGACAGTAAAAGCCCTCAAACAAATTATGAATTCTTCTCAAAGCAACAATGATGATGGTACATAGGTGTTTTGCTTTATCAACATAAAGGAGAAATATTCATATCCTGTAAAAAACTTCTGAAATCACCATGACTCTAGTTATAGATATTTTATGATGAAACAATTGTATGATTATATCAGACTGAGTGAAGTATTTAAAGCCGACCAAATGTTTGGTATGATGAGGGTTTAAAATGGAGGTGAAAATTCATGGATTTTTTTTTTATAAAATTATATTTAAGACTCGTCTATGAATATTTTTACTCCCAATATTACCCCATTGCCAAATTGAGCCTGAATATACCTCTTATTTACCTCTTACTTAGTATTGTTGAGTATCTATATGTTTTTATTGAGAAATCTGTTTGGTCTATTCTGCTTTTAAGTTACTCAAATCACCAACTTTTTATGCATTAATATTTATTGAACGAGCATTTTCTGCATATATATATATAGATATAGAAATCATGCTCTTACGGAATCCAGAAATGTTATGGTTAAGACATTGTTGAAATGATTTGCTGGGTTCCTTTCCAATTATTTAAGCCAGTGGTTTTGAATGTTATCAATTCAATTTTGTTAATGTAGTTTTCGTTCCTTTACCCTCCACCCTGAATTTATTTTGCTCTTATGGAATAAAATTCTTAAATGGGTCATTGTAATAATGAGAGTAAAACATATATAAGGACTAAAAGCCTGTTTATGTTTTATTTATTATGGTTTGATATGTATCGGAAAGAAAAATTGATTAACTTAGAAGTAAGAAAAGAACTCTGCCTCTAATATCATCTACTGCTGAAAACTTTGATTTTCCTCTTTAAAAACCATGTGTAATTGTCGAGCATGGAAGATGGCAAGAGAAATTTAGCCTTTAGTAAATATAGAACTGATGAATGAAAGTCTAATGTGCTAGTGAGACTTGGTATTTTTAATCTTAAGTGCTCTCTGGAATGTTTGCTTTCCCTTCTAAATAGTTTATCTTATGTTTGATGAGAAATCTGTTCTTAATGCATCTGTCCTTCTATTCTTACAGTAAGGAGAATGAAGATCTTTCAGTGAAAGCAAGGTCAAAGTTTAGCACATCTTTACGCAGTTTATCACAACCTATGTCACTTGGGGAGATAGCAAGGACTTGGGATGTTTGTGCTCGCAGTGTTATTTCTGAGCATGCACAGCAGAGTGGGGGAGGAAGCTTCAGCTCTAAGTATGGAACTTGGGAGAATTGTTTGACCACCAACTAATGTTCCATGCAGGCCTTTTTGTTTCGACCCAAACATTCTGCTTCTATTAATATAAGTAATTTATTGGATGTAGATAAAAGCCTAGTTATGAAGTAGCATACCATGGAGTTTGTCAATCTGTATATACTGTTTTTGCTATTGGCAAGGGAATTATCATGATTAGGAAGTAATACTTAAATTATATAAAGCATAATCGCATATTAAGTGGAATATTGTTTCAATAATTGTTTACCTTTTCTATTTCATTTTAGCTTTATAATGTATAAAATTTTGATTTTGATGAGATGAATTTGATTTTTCTATTGGC

>GmMED15-2

TTACACAAGTTGGAAGGCTTCACTGATAAATAGGCCTTGTCTCCCACGCCTAAGTTCCCTCCCAAGTTCACGAGTCAGATACGCAACCGGTAACACTGCTCACGCCACCGCCGGAAAATTCGCAACCACCATTTGCCAGCCACGATCAGGTTCGCTTTCTCTCTCACACTCTATCTCTCGTCGTTGATCTTGCTCAGAAAAAAAAAAAGCTTATTGTTGCATTCACCGTGATTGCTAACGTCTTAATTGAAAGGCCGAGTCGGAAAGAGGCATATTTCGTTCTAAATTCGAATCATTTGGATTCAATAGGACTCGATTTCTTTGAAGGAAACTAGAACGGTACATCGTTCATTGAAGCCAATTTTGGAGAACGGATTTCTAGATAGGAACAAAAATGAGGGAATGCAGAATCTAATATTGGAATTTTGTCCTTTGCTTCCAAAATTAATTACTAGACAAGCCATTTTGTTCTTCGGTAGGAATTATTACTTTTGCATGCATCCTTAGGTTTTCGTCAAGGGAGACCTTAGACTCAATAATATCTCTGAAAGTTTGGTTTTTAACTGTGAGTGACGTCATGTAATCCATGTAGCCGACCTCACATAATGGGATAAGGCTTTGTTGTTGTTGTTGTTGTTGTTGTTGCATGCATCCTAGGTTTAAGCATGGTAAATTGACATTCTAAGTCGTATAAACCCTTGTCTGTCGAGGGAAAAATTATTTGGTTTGCTTAGCTGTTGCTGTGACTGATATTTATGGATGGGCGGTTTGGCTCTGTGAAAACCAATGATTTCTATTGATTATCTTCTTAGTTTTTCATACATTTATATATTCATCCTTTATACGTTCCTTTCGAAATCCGATTTGACAAGAATATTCTGTTTTTGATGTTTCAGTTTATTAAATTTGATGGATAACAATAATTGGAGACCTAATCAAGGCACTGAAGCCAATATGGATACTAGTGATTGGAGAGGTGGACTGCATCATGAGTCACGCCAAAGAATTGTTAACAAAATGTAAGATTTAGTTTTAATATTGTATCACGGATATAATTTTGTAATTTTTTTTATTAACGTTTCACATCAGATATAAATCACTGATTTCATACATTGGCAACATTTCCGTGAGGATTCTGGTTGTCTTTTCTACAAGAAATGATAGATGAGTGTATGTGAAAGAACTTAAACAATGCAAATAAATTATGAAACTTGTGCTCTAATTTTTCTCAAAACAAATGTTTATTTGAATCACTGCTCAACATTGATATTTTCAATATCATAAATTCATAATCATAATCAAAGTGTATGCATGTCAGTGTGGCACGTGCTTATTAGATGGGTTAGTGGGACCGTGTAGTTAAAATCATGATCTGCCTCGTAAGGCCGTATGATGACACGTCAAAGGACCCCTGCTAGCCAAATCGCGAGAGGGTGAGATCACTCTAGGACTGTGATTTACCAAGTTCAATCATGGGATCGTCGATCCCAGCAAAGTGGAGCACAACTATAAATTACCCTTTTTTTCTCCTTTGATGAAATGTTTCCTGCCATTCACGACCTAAATCCCATTCGAAAATCACTCAATCATGCACCATTGGATCTCTCCTTCTTCACAACTGCCAAAGCTCCACCTCATGCCATTAGTGCTTCCACGTTGTCACTGCTGTTGCCGGTTATTGCACCTGTTACCCAACGTCCTGGATTCCCAGCATATTTCTTCTCATTTTCTCAAGGTCAGCCTTCGTTACACAGCATTTGGTAGCATCTCTGCTACCCACCATTTGGTTGTGCTTCTGCTAGCCAGCATTCAGTTGTGCCTCTGCTACCCAGCTTGGGTCTCTCTCACTCAGTGACTCAGTCACTCTCATCTTATCTCACTTTTTTCCATCTTTTAATTCTATTTTTTGGTTTCTCTTACAGAGGCTGGAGCGCTGAATATTTATTTAATTAGCCATTTATTTTCATATTTTTCTAACATTATCTTTGACTTATTATGAATTTTGATTCTATCAATCTATTTTGTTATAAACTTTCATGAATTATGTATTAATCTATTATGTTATGAATTTTCAGAATTATGAATTTTAATTCTCTCTGTCTATCAATCTATTATAATGAATTTCCTATTTAAGTTTGCATATGTATTATTTATAAGTATTTTAGGTAGAAACATAGGATCTTCCGATGTGAGTTACAATCCCTGAGTTATGAGTTTTCACCCCCTCCTAATCCCAGGTAGGATCTTGAGTTTAACACCTTGGGGTGGGACATTATCATACTTGAGTGTTAAACACCATGTCACCAACGTGTACAACTTTAGTGTTTTTCGATCATTTGGAGTTGTATTGCACAATGTGTCGGAACGAACTTTTGCTATCACGGGTGCCTTAGAATATGGGGCACTTGCTTATTCATTTTACTATTTTAGAGGTCTCAAAGGAGGAGTATGAACAAGGTTTATTTTTTCCATGCCCTATATTTGCTAGCCTTTGGTGTGTTTGGTTGGAGGTTACTGCCATTATTTTCAAAGAGGAGTGTGTTTTTTCTGAATACTGATTAGGATTGTGTATTTTGCTCCCTTATGACGGAGGTTATACTTATTTTCTCTTCATGGAATGTGTGGCAAATGGATGTTTAGTAGAAAACTATAGTACTATATTATTTTTCTGTAGGATTTCTTTTTCTTATGTTTTGTGTTTTAGAAGTTTCTGACACCCATTCTACTATATTATTTTTCTGAATAGTTTGATTTCAATGAGGTCTTCTTACATGCTACAATCCTGTAAAATTTGAGGATTGTAATTACCATAAAATTTCAATAATTTGTATTATTTAGTCACCAAACAGCTGTTTTTCATTTTTTATAGAATGGACACATTAAAAAGACATCTTCCTGTTACTGGTCAAGAGGGATTGCATGAACTTCAGAAGATTGCTCAAAGGTTTGAAGAGAAGATTTTTACTGCTGCAACAAGCCAGGTACATTTCTAATTTCTAATTTCAGATTTATCAATTTATGGTTATGTATACAGACATATACAATATGAAAGATTGAAGATTTAATGTGGTATTCTCTAATTTCAGTCTGATTATCTACGGAAAATATCTTTGAAGATGCTTACGATGGAGACTAAATCCCAGGGCTCCATGGCCCCCAACTTACCTACAAATCAAGGTGGCCCTAGTAATAAACCTCCTGATCCAGGTTAGCATTTAGGAACCCTGGCATAGTGGTAGTATTCTCATTCTGACAGTATCTAACTCCGAGTTATTTCAAGTTTTTCAGCTTAAGTTGTTGGGACTGTATTGTTTTCTATTGATTTGAAATATCAAATATCATTAACATTTTTGTTTTTCCTCTGCTATATGATCCTTGTTGTTTGTCTTGAATCTTTCTTCCTTTAAATTTTTTACATTGATATCATTCTTGTGTTGTTAACAGTTCATTTGGATCCTGATGTTATATTGTACTTTTTTTGGGGGGTTTCTCTTACGTCAATCATTATTTATTTAATAGGACTTGGTATTCCACCTCAAGTTCATAATCCAGGGCAGCAACATCCTATTCCTATGCCCAATCAACCTCCGAACCGTCAACAGCTTCTACCTCAGAACATTCAAAATAGTATTGCGTCTCAACCTTCGAATATAGCCCAGGCTCCCATCCAAAATGTTGGGCAGAATAATCCCAACATGCAGAATATTCCTGGTCAGAATTCGGTGGGGAGCACCATTAGCCAAAATTCCAACATGCAAAATATGTTTCCTGGTTCCCAGAGACAAATTCAAGGAAGGCAACAGGTTGTTCCCCAACAACAACAGCAGCAATCACAAAATTCGCAGCAGTATATCTACCAGCAACAAATGCAACATCAACTTTTGAGGCAGAAGCTCCAACAACAACAACAGCAGCAGCAGCAGCAGCAACAGCAGCAGCAGCAACAACAACAACAACAGCAGCAGAATCTTCTACAATCAAATCAGTTGCAATCTTCTCAGCAACCTGCCATCCAAACATCAACTGTTATGCAGCAACCTTCCATGATGCAAGCATCTCTACCTAGCATTCAGCACAATCAACAGTCTAATAATCAACAATCAACACAGTCTGTGCTTCAGCAACACTCCCAAGTTATCAGGCAACAGCAACACCAACAGACTTCGATCATTCATCAACAACAAACACCGATGACTCAACAGTCAATATTACCTACACAACAGCAGCAGCAGCAGCAGCTTATGGGGGCACAGGCAAATGCACCAAACATGCACCATACCCAAATACTTGGGTCACAAAATAATGTTGGTGATTTGCAGCAGCCACAGAGGTTACTTACTCAGCAGAATAATCTTTCAAACCTGCAGCAGCAACAGTTAATAAATCAGCAAAACAACCTATCAAATATGCATCAACAGCTGGGTAATAATGTCCCTGGGTTACAGCCACAGCAGGTCCTTGGACCTCAATCTGGCAACTCAGGCATGCAAACAAGCCAGCACTCTGCACACGTGCTACAACAATCACAGCAAAATGCATCAAATTTGTTACCATCTCAAGTGCAGCAGTCACAGCCTCAGGCTCCACAGCAGCAATTGATGCCACAGATTCAATCTCAACCTGCACAATTGCAACAACAGTTGGGTTTGCAACAGCAACCAAATCCCTTGCAACGAGATGTGCAGCAAAGACTTCAGGCCTCAGGTCCCTTACTTCAACAATCAAATGTTCTTGATCAACAAAAGCAGTTGTACCAATCACAAAGACCCCTTCCAGAAACTTCATCCAGTATGTAAAATTGCTACTATGCATTTTTCTGTGTCTTTTATCTTTGGTCAATTGATGGTTCTAAGGTGATCAAATTTGTGATCCTACCTACTACCTTGGATCATACAGAAGGTAGACACAAATTGTGTGATCAAATCATGGAATATAAGATCCCCTCTTATTTTTTAATTTATTTTTTGGTAATTGATCCCCCTCTTATTTAAAAACTATATTTCAAACATATATAGGTAGCATAACTTTGAAATTGTTACTGACGTTGGAATAACTCTTATTCAAAATACATTATAGATGTAAGTCTAAGCCCTGTCTGCCCAACAAAAGAATCAGATGGTCTAAGCCCTCTAATAAAAACCAAGGTCACAAAGTTAAAGATACAAATCACAACATTAAAACACTAAAAGTTGAGGTGATTGGCCTTGAATATTTATTTTGTGCAAGTATCTTTGTGAAAAGTTACCATCTTTTTTGGCCTTGAATGCTCATGTATGTATGAGATATCTTTTGTAGCTTCTCTGGATTCAACAGCACAGACTGGACAGTCAAGTGGAGGTGATTGGCAAGAAGAAGTGTATCAAAAGGTAATATTCTATTTTCTCATCATCTTTTTTTTTAGTAAATTGGGTTGAACAGATATTTGTCAAATGAACCCTAAAATAAAATAATGAAGATTGATGACTTTTATTGTGATAAATTTTCAGCAAGAACATGGAACTCTTCACTAGTCTTGGAAAAATAATGAGAACTTTGCTTAAATCCATACTGTTCAATGTTTATGCGGATGACTCTCATCCTTCACATAATATTTCTGAAAAATGTGTACTGCAGCAGTAGAAACTACTGGTGCTTTACCCAAAAAGGTTTTTTATTTTTTGGTTTTCTGTCTGCAGTTAATTTCTTTTATTACTATCTTTTTGTCCTCTATTAGACATTAGTTGCTTGCATTGAGAAAGACTCATAGTAGTTGTAAATATGGTTGTGTTGATATTTTGTTTTTAATCAGCAAAAGTAATTGTATTATATATGATTCAGTACAAGTTGTACTGAGAAAATTACACTTGGGTGTCAAATTGAGCAGAAATATGGTGCCCTAATACAAGTTATTCAGCAGAAATATGGTACATATCTTCTCGCACAAGATATTACTAGGTTTCTAATGCATTATTTTTTTTATTATGTTGATTTTAACTGGCAGATCAAATCTATGAAGGAAAGTTACTTGCCAGAGTTGAATGAAATGTATCAAAAAATAGTTAGTAAACTTCAGCAGGTATGTTTAGTCATGCAAATTTTTAATATCTTCTATGGATAAAGAAGCCTTTTCCATTATTATTATTATTATGGCAAACTCTGGAGATTGCACTGTGTGCTGATCAAGTCATCACCCTTCCTGAAACCCTTTATTGTTTTTGTTTTTACTCATTATTTTTACTGTGTTTAACCTTGTATCTTTTCATTATCAGCATGATTCCCTTCCACAACAGCCCAAGTCAGATCAACTTGAAAAGCTGAAGGTATTTAAAATGATGTTGGAACGAATTATAACATTCCTTCAGGTTTCCAAGAGCAACATCTCACCTAATTTCAAGGAGAAACTGAATTCATATGAGAAGCAGATTATAAATTTTATAAATACAAATAGACCTAGGAAAAACATGCCTGGACATCTTCTCCCACCTCATATGCATTCCATGCCACAATCACAACCCCAAGTTACTCAAGTGCAGTCTCATGAAAATCAAATGAACCCTCAGTTGCAAACAACAAATATGCAAGGCTCTGTAGCAACAATGCAGCAGAATAATATGGCAGGCATGCAGCATAATTCTCTGTCTGGTGTATCTACAGTACAACAGAGTAAGATGAATTCAATGCAACCAAGTACCAATTTAGATTCAGGACCAGGAAATGCTGTGAACTCCCTGCAACAGGTTCCAGTGAGCTCACTTCAACAAAACCCTGTTAGTGCTCCTCAACAAACTAATGTTAACTCCTTATCTTCACAAGCTGGGGTTAATGTAGTCCAACCAAATCTTAATCCCCTTCAGCCAGGTTCTAGTATGCTTCAACACCAGCAACTAAAACAACAGCAGGAACAACAGATGTTACAGAATCAACAGTTAAAACAACAATACCAGCGGCAATTGTTGCAGAGAAAACAACAGCAGATGTTACAGCAGCAACAGCAACAGCAACAGCAACAGCAGCAGCAACAACAACAGTTACACCAGACATCAAAGCAGCAGCTGCCTGCACAGTTGCCAACACATCAAATACAACAACTTCACCAAATGAATGATGCAAATGACATAAAGATGAGGCAAGGAATCGGTGTTAAGCCAGGGGTCTTTCAGCAGCATCTTACTTCCAGTCAACGCTCAGCTTATCCCCATCAACAGATGAAAGGGAGTCCTTTTCCTGTTTCTTCTCCTCAACTCCTTCAGGCCACATCTCCTCAGATTCCACAACACTCATCTCCTCAGGTTGACCAACAAAACCACCTTCCATCTCTCACAAAAGTTGCTACCCCTCTGCAATCTGCTAACTCACCTTTTGTGGTACCCACTCCTTCACCTCCCTTGGCTCCATCTCCCATGCCAGGGGATTCTGAAAAGCTGATTTCTGGTGTTTCATCGATCTCAAATGCTGCAAATATTGGATATCAACAAACAGGGGGTGCAGCAGCACCTGGTCAATCCCTTGCCATTGGAACGCCTGGAATATCAGCCTCTCCTTTACTTGCAGAGTTCACTGGTCCTGATGGTGCTCATGGTAATTCTTTAGCACCCACTTCTGGAAAGTCAACTGTTACTGAGCAGCCTATTGAGCGCCTAATAAAAGCGGTTAGTCTAGTGGATCTTAGGCTGGCCTGATCAGTGGCTGTGGCCTTTAAGTTTTTTATGAATTTGACAAATAAACGACATAAAGGTTTCCATAATTGTTCAACTGACAAATTTATGTCCAATCATGATTCCAGGTGAAATCAATGTCACCAAAAGCATTAAGTTCTGCTGTCAGTGATATTGGTTCAGTTGTCAGCATGAACGATAGAATAGCAGGATCAGCTCCAGGTAATGGATCCAGAGCTGCTGTTGGCGAAGATTTGGTTGCTATGACTAATTGTCGTCTTCAGGCTAGAAATTTTATCACCCAAGATGGTGCCAATGGAACTAGGAGGATGAAGCGCTACACCAATGCAACACCCTTGAATGTTGTAACTTCTGCTGGTAGCATGAATGACAGTATCAAGCAGTTAGCTGCTGAGGCTTCTGATTTGGATTCAACTGCAACATCTAGATTCAAGATGCCGAGGATTGAGGTACTCATTTTTATTTATAATTGATATCTTTTACCATCGATGCCTTTTTGGGCTTGCTATTGCAATTTATTAATGGGCTTCTACACATGATGAATGACATATGCACCAACACATTATGTAATTACTTTTTTACCCTTTCTGTCTACAAGGATTCAGACTCAAAAAACAATGCTACTAGTTCAATATAGAAGTATGTAGAAAGGTGTAATTTCGTGTTCTCATTTAGATGGTGAAATATATCTAGAGGCTATATATCAAAATGCATTGTAGATTTCATACTAATGATATCATCAACCAACCAAATATTTGATGCCTGAAAAAAACACACTTAACACAATAAACAAGGATCCAGGTGGGGCAAGTGGAAAGAAATAAACATGTGGTGATATATTTGAACTGATGCACAGTTTTATTTCATGAAAGTGTAAGTTATTTGCAACTACCAGTATTTTGTACATGTATCAAAGGATAAAATGTAGTTTATACCTTTGTTTATGATTTTTCAGGACATTTTTGCTGGATCTAGTCCAAATTTTGAGATAGGAATTGCTGAATTAGACACTCATCTGTGATATTAATTTGATGAATTTAGTTGTTAAATGTTAATTTTAAAGATTCAAGAGATACTGAATGATTTAGTAAAATAATAGCAATCACAACCAATCAAAAGAAAATTTGTAGTAGTAGTGGTGGTAGGCAGTAGCATAAAAAAAGTGCTAGTGGTTAGGTTGATTCATGTTGTTGAGTTGTTAGTCTTTAATTCTTTTGAATTTTGATTAATAATTTCTCCAGTTTTTACAAATAACCATTATCCTCCCCATTTATGAATAATAATCAAGGGTGGCATGAGGTTAGAGCATAGAACCTATTGTTACGTGTGACTCTTGTAGCAGGTCTTAAATTGTTGATCTGGACAGCAACTGGATTCATTATATAATGTCTTGGATTTTGCATGATCTGGATTAGCTATTGACATTTGTTTTATCATAATGATTTGTCAGATATTAGGCTTTGAAATTATTCTGAAGTTTGCACTGAGGATTTGGAACACTTTATAATTTATGGATTTGCTCTACACAGCCTTTGATTGCTGATTAGAAATTTATGTGACTTATATGGCAGGCTAATCATTCCCTTTTGGAAGAAATTAGGGAGGTTAATCAGCGACTTATTGACACAGTAGTAGACATAAGTAACGAAGAAGTTGATCCAACGGCAGCTGCTGCTGCTGCTGAAGGGACAGAAGGGACCATTGTCAAATGTTCTTACAATGCTGTGGCTCTCAGTCCGAGCTTAAAATCCCAATATGCTTCAGCACAGATGGTGGGCTTTAAAACTCTTAAACCATATGCATATCAAGTATTTATTAATTCTTTTTGTCATAAAACTTTGTCTTTATTTGTGTTTTACTGCAATGCAGTCACCAATTCAACCCTTGCGATTGCTTGTTCCTACAAATTATCCTAATTGTTCCCCCATACTCCTAGACAAGTTTCCAGTTGAATCCAGGTGAGAGAATGAGTATTTTGGCATCTCGTATGTGTTATACTTTTATTTGCTATGTTTGCATTTATCAATAAAAAGGACAAATGTAATGCTATCTCGCTAAACAAAACTTCTTTTGGTGATTGTATTTTGTTTTTTCATTGATGAAACATGTGTTTGCTCTATTATATCAGACTGAGTAAAGAAGATAATGCAGAAGGGTGCCTCTTATAATTTCTTCTGCTTGTATTGATTAAAATTCTGAATCATGAGGCATTAGAAAACATATGTAAAGATCAAAAGTTTTTTTATGACAACCTAAGTTTTTTTTTTCCACTAGGTGGAGTTGGGGTTATATTGATCAATTCAGTTCATTTTGTTACGTCACAAACCACGTGTTCACAAATCATTCAGGTCTAAAAAAAAGTCTTTTGTTATGGTTTTACCCAGTTTTTCTTCATCTTTCTTGCCTTCACCTCTGACTGTCTGGGTATCTTCTTAGGGCTTTCTATTTGTCTACTTTTCATATGCTGAACCACCTTAAGTGTGTTTTTATCTTATTTTACTCAACAGATGCTACCCCTATTAAATTTTCTTCTTAGAAATGTATAGCAAAGACCATGGCCATTTTACCTGTCAGTAATGTTTTAATATGTATCCGTCTTCCGAATGAACATGGGTGATTTTAGAAAAACATGTTTAATTTATGCCTCAAATTAATCCTCTATTGGTTTAAACTATAATTTCTCTTTTTAAATAGCAGATGTGAATTTGAGTTTAAATCTAACGCAAAAAAATGTGGAGATGTTTTGTCTGAAAGATACATAAGACTCTGATGTGCTAACGAGACTGACCATTATTAATTTTTATTATTCTCTGAAATATTCGTTTATGCCCCTTCCAAACAGATATGATAAACAGAATGCACCACTTTACCTTTTCTCCGTAGTTTATTTTAGATTTGCTGAGAATTCTTCTGTTTCTAATAAAAGTAAGTGGTATTGAATATTTCCCAGTAAGGAGAATGAAGATCTTTCTGTGAAAGCGAAGTCAAGGTTTAGCATATCCTTGCGAAGTTTATCACAACCTATGTCCCTTGGGGAGATAGCAAGGACTTGGGATGTTTGTGCTCGCACTGTTATTTCTGAGCATGCACAGCAGAGCGGTGGTGGGAGTTTCAGCTCTAAGTATGGGACTTGGGAGAATTGCTTGACCACATGATGATCCTTGCCGTTCCATGCAAACAAACATCTTTTTTCTTCTGAAATTTGCCAAAACTTGTGTCAATCATTGGAATCGGAGTTTTTGGGTGCAGGTGAAAAGCAATTTTGCATCAGGTCAAATTGGTTCCGTTTCAGTGAATTGTGTATTCCGTTAGTATCCATTTTCTTGTTTATTTATATCTCAAATTGCGATATTTACCGCTGCCTAAGATATGTGGTAGATTTGTGTGACTTGAATCAAATTGCCTTCACCAAGGCTTTGTTCATAACTAATATAGTAGCTCAACAGTTTAGGCCCGTAATTTCTGCATAAGAACAATTCTTCCAGATGCTCATAATTAAATTGTTAGTTATGATTAGTTATGTATCTTAAAAAACTTGTTCATGTGAAGTGAGTTATGAATA

>GmMED15-3

TAAATAGGCATTGTCTCCACGCGTAAGTTTCCTTCAAGTTCACGAGTCAGATACGCAACCGGTAACACTGCTCACGCCACCGCCGGAAAATCGCAACCACCGTCTGCCACCATCAGGTTCTCTTTCTCTCTCTCCCTCTCCCTCTCGTCGTTTATCTTGCTCAGAAAAAAAAGCTTATTATTGCATTCACCGTGATTGCTAACTTCTTAATTGAAAGGCCAAGTAGGAAAGAGGCGTATTTCGTTCGAAATTCGAATCATTAGGATGCAATAGGACTCGATATCTTTCAAGGAAATTAGAACAGAACCCGTCCATTGAAGCCAATTTTGTAGAACGGATTTCTGGATAGGAACAAAAATGAGGGAATGCATTATCTAATATTGGAATTTTGTTCTTTGCCTCCAAAATTAATTACTAGACAGGGCATTTTGTTATTCGGTAGAGAATTATTTCTTTTGCATGCATCCTAGGTTTAAGCATGATAAATTGGCATTCTAAGTCGTATAAACCCTTGTCTGTGTCGAGGGAAAAATTATTTGGTATGTTTAGCTGTTGGTGTGACTAATATTTATGGATGGGCGGTTTGGCTCTGTTAAAACCAATGATTTCTATGGATTATCTTCTTAATTTTTCATACATTTATTATATTCATCCTTTATACGTTCCTTTTGGAATCTGATTTGACAAGAATATTCTGTTTTTGTTGTTTCAGTTGATTAAAATTTGATGGATAACAATAATTGGAGACCTAATCAAGGCACTGAAGCCAATATGGATACTAGTGATTGGAGAGGTGGACTGCCGCACGAGTCACGCCAAAGAATTGTCAACAAAATGTAAGATTCAGTTTTAATATTGTATCACTAATATCATTTGTAATTTTTTATTTACGTTTCACATCAGATATAAATCACTGATTTCATAGATTGGCAACATTTCCATGAGGATTCTGGTTGTCTTTTTTACAAGAAAATGATAAATGAATGTATGTGAAAGAACTTAAACAATGCAAATAAATTATGAAACTTGTGCTCAAATTTTTCTCAAAAATGTTTATTTGAATCCCAGCTCAACATTGATTTTTTAAATATCATAATCATATTCAAAGTGTATGCATGAACTTTAGCATGTCAATGTGGCACATGCTTATTAGATGGGTTAGTGGGACCGTGTTGTTAAAATCATGATCTGCCTTGTAAGGTTGTATGATGACAGGTCAAAGGACCCCTGGCGAGCTGAATCGCTGGAGGGTGAGATCACTCTAGGATTGCGATTTACCAAGTTCAATCATGGGATTGTCGATCCCAGCATCCCAGCAAAGCGGAGTATGGCTAAAATTACCCTTTATTTCTCCTTTGATGAAATGTTCCCTGCCATTCGAGACCTAAATCCCATTCAAAAATCACTCAATCGTGTGCCATTGGATCTCTCCTTCTTCACAACTGCCAAAGCTTCACCTCGTGCCACTAGTGCTGCTGTGCGTTGTCACTGCTGTTGCCAGTTATTGCACCTGTTACCCAACGTCCCAGACTCCCAGCATATTTCTTCTCATTTTCTCTAGTTCAGCCTTTGTTACACAGCATTTGGTAGCATCTCTGCCACCGACTATTTGTTTGTGCCTCTACTACCCAGCTCAGGTCTCTCTGACTCAGTGACTCAGTCACTTTCATCTTATCTCACTTTGTTCCATCTTTTAATTCTATTTTTTGGCTTCTCTCACAGAGACTGGACTGCCAAATTTTATTTTTTTTAATCGGCCAATTATTTTCAGATTTTTCTAACATTATCTTTGACTTATTATGAATTTTATTTCTATCAATCATTTTGTTATGAACATCCATTAATCTGTTATGTTATGAATTTTCAGAATTATGAATTTTAATTCTCTCACTCTCTCTGTCTATCAATCTATTATAATGAATTTCCTATTTAAGTTTGCTTAATGCTTTGATTTAGAGGTTATGTTCTTTATCTGTTTTTTTTATGCATATGTATTATTTATAAGTATTTTAGGTTGAAACATAGGATATTCCAATCCGAGTTATGAGTTTTCACCCCCTCCTAATCCTAGGTAGGATCTTGAGTTTAACACTTGGGGTGGGACATTATCATACTTGGCTGTTAAACACCATGTCACCAACATGTACAATTTTAGTGTTTTTCGATCATTTGGTGTTGTATTGCACAATGTGTTAGAACAAACTTTTGCTATCATGGGTGCCTTAGAATATGAGGCACTTGCTTATTCATTTTACTATTTTAGAGGTCTCAAAGGAGGACTATGAAAAAGGTTCATTTTTTCCATGCCCTATCTTTGCTAGCATTTGGTGTGTTTGGTTGGAGCTTACTGCCATTATTTTCAAAGAGGAAAATGTGTATTTTCTGCATACTGATTAGGATTGTGTATTTTGCTCCCTTATGACAGAGGTTATACTTATTTTCTCTTCGTGGAATGTGTGGCAGATGGATGCTTAGTAGAAAACTATAGTACTATAAGTAGGATTTCTTTTTCTTATGTTTTGTATTTAAGAAGTTATCAAAAATAATCTCTGCCGCCTGCAATCCATTTCTGACACACATTCTACTATATTATTTTTCTGAATATGTAGATTTCAATGAGGTCTTCTTACATGCTACAATCTTGTAACATTTGAGTATTGTATAATTACCATAAAATTTCTATAGTTTGTATTATTTAGTCACTAAACTGCTGTTTTTCATTTTTTATAGAATGGACACGTTAAAAAGACATCTTCCTGTTTCTGTTCAAGAGGGATTGCATGAACTTCAGAAGATTGCTCAAAGGTTTGAAGAGAAAATTTTTACTGCTGCAACAAGCCAGGTACATTTCTAATTTCAGATTTATCAATTTATGGTTATGTATACAGACATATGCAATGTGAAAGATTGACGGTTTAATGTGATATTCCCTAATTTCAGTCTGATTATCTACGGAAAATATCTTTGAAGATGCTTACGATGGAGACTAAATCCCAGGGCTCCATGGCCCCCAACTTACCTCCAAATCAAGGTGGCCCCAGTAATAAACCTCCTGATCCAGGTTAACATTTAAGAACCCTGGCATAGTGGTAGTATTCTCATTCTGATAGGATCTAACTCTGAGTTATTTCAAGTCTTTCAGCTTAAGTTGTTGGGAGTGTATTGTGTCCTATTGATGTGAAATATCATTAACATTTTGTTTTTCCTCTGCTATATTATCCTTGTTGCTTGTCTTAAATCTTTCTTCCTTTTAAATTTTTACATTGATATCATCTTGTGTTGTTAATAGTTCATTTGGATCCTGATGTTATATTGTACTTGTTTTGGGGGGTTTCTCTTACGCCAATCATTATTTATTTAATAGGACTTGGTATTCCACCTCAAGTTCACAATCCAGGGCAGCAACATCCTATTCCTATGTCCAATCAAACTCCGAACCGTCAACAGCTTCTACCTCAGAACATTCAAAATAGTATTGCGTCTCAACCTTCGAATATAGCCCAGGCTCCCATCCAAAATGTTGGGCAGAATAATCCCAATGTGCAGAATATACCTGGTCAGAATTCAGTGGGGAGCACCATTGGCCAAAATGCCAACATGCAAAATATGTTTCCTGGTTCCCAGAGACAAATTCAAGGAAGGCAGCAGGTTGTTCCCCAACAACAACAGCAGCAATCACAAAATTCACAGCAGTATATCTACCAGCAACAAATGCAACAGCAGCTTATCAGGCAGAAACTCCAACAACAGCAGCAGCAGCAGCAACAACAGCAGAATCTTCTACAATCAAATCAGTTGCAATCTTCTCAGCAACCTTCCATCCAAACATCAACTGTTATGCAGCAACCTTCCATGATGCAAACATCTCTACCTAGCATTCAGCACAATCAACAGTCCAATAATCAACAACAATCAACACAGTCCGTGCTTCAGCAACACTCCCAAGTTATCAGGCAACAGCAACCACAACAGACTTCGATCATTCATCAACAACAAACACCGATGACTCAACAGTCAATATTACCTCCACAACAGCAACAGCAACAACAGCTTATGGGGGCACAGGCAAATGCACCAAACATGCACCATACCCAAATACTTGGGTCACAAAATAATGTTGGTGATTTGCAGCAGCCACAGAGGTTACTTACACAGCAGAATAATCTTTCAAACCTGCAGCAGCAACAGTTAATAAATCAGCAAAACAACCTATCAAATATGCATCAACAGCTGGGTAATAATGTCCCTGGGTTACAGCCACAGCAGGTCCTTGGACCTCAACCTGGCAACTCAGGCATGCAAACAAGCCAGCACTCTGCACATGTGCTACAACAGTCGCAGGTTCCAATTCAACAACAATCACAGCAAAATGCATCAAATTTGTTACCATCTCAAGTGCAGCAGTCACAGCCTCAGGCTCCACAGCAGCAATTGATGCCACAGATTCAATCTCAACCTGCACAGTTGCAACAACAGTTGGGTTTGCAACAGCAACCAAATCCCTTGCAACGAGATATGCAACAAAGACTTCAGGCCTCAGGTCCCTTACTTCAACAATCAAATGTTCTTGATCAACAAAAGCAGTTATACCAATCACAAAGACCCCTTCCTGAAACTTCATCCAGTATGTAAAATTGCTACTATGCATTTTTCAGTGTCTTTTATCTTTTGTCAATTGATGGCTCCAAGGGGATCAAATTTGTGATCCTTCCTACTACCTTGGATCATATAGGAGGTAGACAAATTGTGTGATCACATCATGGAATATAAGATCCCCCTCTTATTTAAATACTATATTTCAAACATATATAGGTAGGATAACTTTGAAATTGTTACTGGCATTGGAATAACTCTTATTCAAAATACATTATAGATGGAAGTCTAAGCCTTGTCTGCCCAACAAAAGAATCAGATGATCTAAGCCTTCTAATAAAAACCAAGGTCACAAATTAAAGATACAAATCACAACATTAAAACACTAAAAGATTGACCAGCTAAATGGAATTTTGTGAGGGTTGGCCTGCAAAATGTAATATTACAAAAAAGAGAAAGGACTGGACACAACAGGCATATTAATTATTAATATAGTATGCTGGGCTTTCTGTTATGACAGCTGTCATCTTTAACTAACTCTTAGTTAGTTAGACACTGTTAGAGCTTAGAGTCTCTAGCCAAACTCATATAAATACTTAGTGTTTTTTTAAAAGGCATATAAGTACTGAGTGTAACTCAGTCTTTGTAGCTTTTGATTCATATTTCAGAATACAAAAATATTTCACTGAAATTCTCTCTGAAGTCTGAGCTTTATTATGGTATCAGAGCAGGGTTCGAACCCTGGATTTATTCCCTCCCTTCTGTGCACAACAACTCTTCTCCTCCTTCCCGATTGACACCTATTCATGCAGAGCCTGTATCGGCTTTTCCTCTGGCAGCATCTGTTATCTTCTCGCACACCATATCTGAAAAATGGATGTAATCAATAGCATGCTCTAGCAGAGTGCGGACCGAAGCACAGCCATGGGCAGCTATGGGATTCAAATAGTAGTCAGTAGAGTGGTTTTCAATAGTGCTCATTGCATCTGCAGTAGCTGCCAAAATAAGGGGGCACTACGAAGATATGGCCTGCAAAAACCCATTTTTACCCTTGAAAAAATATGATTATTATTATTTATTTATTTACTCTTTTTTTTTTTTTGGGGGGGGGGGGGGGGGTGCAAAGTTGTGATTTCTCCCCTATTTTATAATTCTTTTATTTTGCTTTAGAAACATAATAAGACTTGAAACCCTTCTTCCTTGACCGTTGTACAAAAGGTTACCTCTATTTGACCTTTGTTTCTTCACTTTTTACAAGACTTTTTTCCCCTTTGTGTTTGTTCTATCTTCCTTTAAACTTCCCCATCCTCATTCAGCTACTTTTTTTCTTCTAATTTGTTATGTTCTTTTTCTGTTAATTTCACTATCACTTGTTCTTATTGTGCTTAGATTGGTGGTAATTGTGTGTGTAAATTGTGACTTATATTTATTACCTTTTTTTTTATTGGCAAATGTTAGTAGTTATTATTCATTACTATGTTCGTATTGCGCCTGGATTGGTGGTAGTTGTGTGTGTAAATTTTGACTTATTATTATCATGAATATCTTTAATTATATGTGTAGTATAAATCAGTGGTCCATAGGTTAAGCCACTTTGTGAAAGTATCTTTGTGAAAAATGACAACATAGGTTATCACAATGTCTCATGAATGTATCTTTTGTATTTGATAGATAATGTTCTCTAAGTTAGTTGGCAAATATCTCATTTTTTACTTCCAAAAAAGCAAATATCTGCATTTTTTGGCCTTGAATGCTCATGTACGTATGAAATATCTTTTGTAGCTTCTCTGGATTCAACAGCACAGACTGGACAGTCAAGTGGGGGTGATTGGCAAGAAGAAGTGTATCAAAAGGTAATATACTATTTTCTTATCATCTCTTTTCCTTTAGTAAATTGGGTTGAACAACAGATATTTGTCAAATGAACCCTAAAATAAAATAATGAAGATTCATGGCTTTTATTGTGATGAATTTTCAGCAAGAACATGGAACTCTTCACAAGTCTTGGAAAAATAATGAGAACTCTTCTTAAATCTATACTGTTCAATGTTTATGTGGATGCCATTCATCCTTCACATAATATTTGAAAAAATGTGTACTGCAGCAGTAGAAACTACTGGTGCTTTACCCAAAAAGGTTTTTTATTTTTTGGTTTTCTGTCTGCAGTTAATTTCTTTTATTACTGTGTTTTTGTCCTCTTTTAGTTGCTTGCATTGAGAAAGATTCATTGTAATTGTAAATATGATTGTGTTGATATGAGTGAGATTTTAAAGTTTTCAATTCAATATTCAAAATCGTGGTGCCAGGTGCACGTGTGCATGGTTAACATCTCAATTTTAAGCATGAAAGTGAAACTTGGAATTTATGATGCATTACAAAATTGGAAAATAATTCTTATCCTTTTTTAAGGCATCAGTGGTACATATCTTCTTGCACAAGAGATTACAAGATTTTTAATCCATTGATTATTTGATTCTGTTTATTTTAACTTGCAGATTAAATCTATGAAGGAAAGTTACTTGCCCGAGTTGAATGAAATGTACCAAAAAATAGCTAGTAAACTTCAGCAGGTATGTTTAGTCATGCAAATTTTTAATATCTGCTATGGATAAAGAAGCCTTTTCCATTATTATTATTATGGCAAACTCTGGAGATTGCACTGTGTGCTAATCAAGTCATCACACTTCCTGAAAGGGAGTCCTTTATTGTTTTTGTTTTTACTCAAATTATTTTTACTGTGTTTAACCTTGTATCTTTTCATTATCAGCATGATTCCCTTCCACAACAACCAAAGTCAGATCAGCTTGAAAAGCTGAAGGTATTTAAAATGATGCTGGAACGAATTATAACATTCCTTCAGGTTTCCAAGAGCAACATCTCACCTAATTTCAAGGAGAAATTGAATTCATATGAGAAGCAGATTATAAATTTTATAAATACAAATAGACCTAGGAAAAACATGCCTGGACATCTTCCCTCACCTCATATGCATTCCATGTCACAATCACAACCCCAAGTTACTCAAGTGCAGTCTCATGAAAATCAAATGAACTCTCAGTTGCAAACAACAAATATGCAAGGCTCTGTAGCAACAATGCAGCAGAATAATATGGCAGCCATGCAGCATAATTCTCTGTCTGGTGTATCCACAGCACAGCAGAGTAAGATGAATTCAATGCAACCAAGTACCAATTTAGATTCAGGACCAGGAAATGCTGTGAACTCCCTGCAACAGGTTCCAGTGAGCTCACTTCAACAAAACCCTGTTAGTGCTCCTCAACTAACTAATGTTAACTCCTTATCTTCACAAGCCGGGGTTAATGTAGTCCAACCAAATCATAATCCCCTTCAGCCAGGTTCTAGTATGCTTCAACACCAGCAACTAAAACAACAGCAGGAACAACAGATGTTACAGAATCAACAGTTAAAACAACAATATCAGCGGCAATTGATACAGAGGAAACAACAACAGATCTTACAGCAGCAGCAGCAGCAACAACAGCTACATCAGACAGCAAAGCAACAGCTGCCTGCACAATTGCCAACACATCAAATACAACAACTTCACCAAATGAATGATGCAAATGACATAAAGATGAGGCAAGGAATCAGTGTTAAGCCAGGGGTCTTTCAGCAACCTCTTACTTCCAGTCAACGCTCAACTTATCCCCATCAACAGATGAAAGGGAGTCCTTTTCCTGTTTCTTCTCCCCAACTCCTTCAGGCCACATCTCCTCAGATTCCACAACACTCATCTCCTCAGGTTGACCAACAAAACCACCTTCCATCTCTCACAAAAGTTGCTACCCCTCTGCAATCTGCTAACTCACCTTTTGTGGTACCCACTCCTTCACCTCCCTTGGCTCCATCTCCCATGCCAGGGGATTCTGAAAAGCCCATTTCTGGTGTTTCATCAATCTCAAATGCTGCAAATATTGGATATCAACTAACAGGGGGTGCAGCAGCACCTGCTCAATCCCTTGCCATTGGAACACCTGGAATATCAGCCTCTCCTTTACTTGCAGAGTTCACTGGCCCTGATGGTGCTCATGGTAATGCTTTAGCACCCACTTCTGGAAAGTCAACTGTTACTGAGCAGCCTATTGAGCGCCTAATTAAAGCGGTTAGTTTAGTGGATCTTAAGCTGGCCTGATCAGTGGCTGTGGGTTTTTTATAAATTTGACAAATAAACTACATAAAGGTTTCCATAATTGTTCAACTGACAAATTTATATCCAATCATGATTCCAGGTCAAATCAATGTCACCAAAAGCATTAAGTTCTGCTGTCAGTGATATTGGTTCAGTTGTCAGCATGAATGATAGAATAGCAGGATCAGCTCCAGGTAATGGATCCAGAGCTGCTGTTGGCGAAGATTTGGTTGCTATGACTAATTGTCGTCTTCAGGCTAGAAATTTTATCACCCAAGATGGTGCCAATGGAACAAGGAGGATGAAGCGCTACACTAATGCAACACCCTTGAATGTTGTATCTTCTCCTGGTAGCATGAATGACAGTATCAAGCAGTTAACTGCTGAGGCTTCTGATTTGGATTCAACTGCAACATCTAGATTCAAGATGCCAAGGATTGAGGTACTTATTTTTTTAATTAATTTTTATTTATAATTGATATCTTTTACCATCAATGTCTTATTTTTTGGGCTTGCCATTGCAATTTATTAATGGGCATCAATACATGATGAATGACATGCACCAACACATTATGCAATTACTTTTTCACCCTTTCTGTCTACAAGGATTCAGACTCAAAAATCAATGCTACTAGTTCAATAGAAGTATGTAGAAAGGTGTAATTTCATGTTCTCATTTAGATGGTGAAATATATCTAGAGGCTATATATGAAAATGCATTGTAGGTGGGGCAAGGGGAAAGAAATAACGCAACAAACAAGGATCCAGGTGGGGCAAGGGGAAAGAAATAAACATGTGGTGAGATATTTGAACTGATGTACACTTTTATTTCATGAAAGTGCAAGTTATTTGCAACTACCAGTGTTTTGTACATGTATCAAAACATAAAATGTAGTTTATACCTTTGATTATGATTTTTCAGGACATTTTTGCTGGATCTAGTCAAAATTTTGAGATAGGAATTACTGTATTAGATATTCATCTATGATATTAATTTGATCAATTTAATAGATAAATGTTATTTTTAAAGATTCAAGTGATACCGAATGATTTAGTAAAATAATAGCTATCACAACCAATCAAAAGAAAAATTGTAGTAATAGTGGTGGTAGTAGTAGGCAGTAGCATGCAACAATAAAAAAAATATATTAGTGGTTAGGTTGATTCATGTTGTTGATTTGTTAGTCTTTAATTCTTTTGAATTTTGATTAATAATTTCTCCAATTTTTACAAATAACCATTATCCTTCCCATTTATGGATAATAATCAAGGGTGGGAGGAGGTTAGAGCATAGAACCTATCGTTACGTGTCACTCTTGTAACAGGTCTTGAATTGTTGACCTGGACAGCAACTGGATTCATTATATAATGTCTTGGATTTTGCATGATCTGGATTAGCTATTGACATTTCTTTCATCAGAGCATAATGATTTGTCATATATTAGGTTTTAAAATTATTCTGAAGTTTGCATTGTGAGGATTAGGAAATCACTTTATAATATATGGATTTGCTCTACACAACCTTTGATTGCTGATAAGGAATTTATGTGACTTATATGGCAGGCTAATCATTCCCTTTTGGAAGAAATTAGGGAGGTTAATCAGCGACTTATTGACACGGTAGTAGACATAAGTAATGAAGAAGTTGATCCAACAGCAGCTGCTGCTGCTGCTGAAGGGACAGAAGGGACCATTGTCAAATGCTCTTACAATGCTGTGGCTCTCGGTCCGAGCTTAAAATCCCAATATGCTTCAGCACAGATGGTGGGCTTTAAAACTCTTAAACCATGTGCATATTAAGTATTTATTAATTCTTTTTGTTATAGAACTTTGTCTTTATTTGTGTTTTACTGCCATGCAGTCACCAATTCAACCCTTGCACTTGCTTGTTCCTACAAATTATCCTAATTGTTCCCCCATACTCCTAGACAAGTTTCCAGTTGAATCCAGGTGAGAGAATTAGCATTTTGGCTTCTCTTATGTGTCTTATACTTTTATTTTCTCTGTTTGCATTTATCAATAAAAAGGACAAATGTAATGCTCAAATCTTGCAAAACAAAACTTCTTTTGGTGATTGTGACGTTTTTTTCATTGATGAAACATGTGTTTGCTCTATTGTATCAGACTGAGTAAAGAAGATAACGCTGAAGGGTGCCTCTTATTATTTATAGCAAAGACCATGGCCGTTTTACCTGTCAGATTTGCTTTAATATGTATCCGTCATCCAAATGAACACGTGTGATTTTAGAAAACATATGTTTAATTTATGCCTCAAATTAATCCTCTATTGCTTTAAGCTATAATTTCTCTTTTTAAATAGCAGATGTGAATTTTTGAGTTTAAATCTAACAAGGAAAAATGTGAGATGTTTTTCCTGAAAGATACATATGACTCTGATGTGCTGACGAGACTGACTGACCATTATTAATTTTTATTATCCTCTGAAATGTTGGTTTATGCCCCTTCCAAACAGAATGTATCACTTTACCTTTTATTTTAGATTTGCTTAGAATTCTTCTGTTTCTAATAAAAATAAGTGGTATTGAGTATTTCTTTTTTCAGTAAGGAGAATGAAGATCTTTCTGTCAAAGCGAAGTCAAGGTTTAGCATATCCTTGCGAAGTTTATCACAACCTATGTCCCTTGGGGAGATAGCAAGGACTTGGGATGTTTGTGCTCGCATTGTTATTTCTGAGCATGCACAACAGAGCGGTGGTGGGAGTTTCAGCTCTAAGTATGGGACTTGGGAGAATTGCTTGACCACATGATGATCCTTGCCGTTCCATGCAAACATCTTTTTTCCTCTTAAATTTGCCAAAACTTGTGTCAATCATTGGATACGGAGTTTTTGGGTGCAGGTGAAAAGCGATCATGCATCAGGTCAAATTGGTCTCGTTTCAGTGAATTGTGTATTCAGTTAGTATCCCATTTTCTTGCTTATTTTTACGCTCAAATTGCGATGTTTGCCGCTGCCTAAGACGCTCAATGTAGTAAGATTTGTGTGACTTGAATCAAATTGCCTTCACCAAGGCTTTGTTAATAACTAATATAGTAGCTCAACAGTAAGGCCCGTAATTTCTGCAGAAGAATAACACTTCCAGATGCTCATAATTAAATTGTAGTTATGATTAGTTATATATCTTAAAAAAACTTTTTCTTTC

>GmMED16-1

AAAAGTTGAAATCCCAAAAGCCTTTAAAAACAGCTTAACTCTTAAGAAAACAACAAAAGAAAAACAAAAAGTATTCTGGAGAATCACCGGTGTCTTTGTTTGGTTTGTCCACCACATGCCCAGCACGAAAAACCCTAACCCCAGAAATCACAATCCCCCTTTATTAAACAATTGAAAACCCCTTCCCTCTGTTCCTCCCCTTTTTCCATTCACACACACAACACTAACCCCACTCTCTCTCTACATTTCCAACAGTGGTATCTGTCGCCTTCAATTGCGACTTTCCACCACTAACCCCATTTTTTCTCTCACTTTCTCTTTCTTATATAAAATTTAGGGCAATTGGGAATGAATCAAATTGGTGGGACAAAGGACCCGGAGGCCGAGCCTGTCTCTCCGTCTCTGCCCCAACCCCAAGACACTCCCAAGAGCTCCGACAAAACCGATTCCGCCACCGCTGTCGTTGTCGTCGCCGCTGCCTCCGAAGAGCAGGAGGTCGGAGAGGAGGAAAAGCCGCCGCCTCCGCCTGTCCCGATGGAGGAAGATCCTGTCAACCCCGCCACCGTTTTCTCCATCAGGCTCAAGCAGCCTAGGTCCAATTTGCTGCACAAGATGAGTGTGCCTGAACTTTGTCGCAATTTTAGGTCAGTGCTTTGAAAATTGAACTGTTTCTATAGCTCAAATTAATGATTTTGTTGATGCATTTTTTTTTGGGGGTGGGGGAGAGGGGAGTGTTATGAATTGGAAGCTTGTTTTCTTAGTGTGATAGTTAGTGCTTGACTTTGATCGGATAAACTTCTCCATGAGGTAGAAATGAATGAATTTAGTTCCTCTTATAAGCTAAAATCAACTTATTGCACTTCAATTTTCTGAGAAGTTAGACGAGAGGATTTATAAAAGTTACGTGCATAAGTTGATTTTAGTTCACCGTAGAAATTTTCCTCTTATATGTGATTATGGAGAAGTTTTTCCAAACATAGTTCATGAAATGAAGTGCTTTCTTTTTGTGTATTCAAATTTGGGAGAACAAAAAAAATGCTCGGTGCTGATGTAGCTTGTAGTCATCCCTTATGATGATTATTTTCATCCTTATGCTGATCATGGGCATAATATGTGGCAACAATTCCTAAAAGAACGTAGACTGACTTTGCATGGTTCAGAGTGTACGAGTTTTTCAGTGTAAAATTTAGATTCTGACATGCTTTTTTTTGGTCTTGCAATTCATGTTTGTGTACTATTGTAGGTCTTTTATGGCACAATAATTTTGGTACTATGGATTTCTCACATTTTTTTTAATCTTGTTTGTTTTTTCTGCAGTGCTGTTTCGTGGTGCGGAAAACTGAATGCTATAGCTTGTGCAGCAGAAACGTGTGCCAGAATTCCAAGGTTTTTTTTAATACATTTGGACATGTTTTCAAATCTTTGACTGTTCTATATGTTTAATTAAGAACAAAACTTTCTGTTTGTATATATGATAATTGTAGTAAAACACAACCAGAAGAATTGTTGGAGTTTATAAGATTGTGAAGATCCATTGCATGATACGTTATTGATAAAATCAACTAGACTTTGGAGTGTGTTTGGGTAAATAGCTTAGTTATGTACTTATTAATAAGTACTTATCATAAATTGTTTTTATAATCAAATAGTAGATATGATTAAACTGCTTTCATACAATTTGTAAGCTGTTTTCATAAGCTTATTATGGAGAGCTTATTGAACTAAGCTGAAACAACATGTAAACAGTAAACATATCATAAACTATTTTCATAAGCTCTCCCAAACACTTACACAAGTACTTCTGTCAAGAGTAAGATAAGCCCAAATAAGCTGATAAAAAAAAAAAACTCTTTCAAATGCACATAGTTTAGTTGATGAACATCTCAAACTTTCTAATCTACATTTTTTCTATGTTGATTGTGAAGACCTAAATGAAGTACTGACCAGAGATATTGTGGATATATCTTTTAACATATTTAACAGTGTCTTTGAGGTTTGAACATGTTTCATTAATTGGCTAACATAATATCTTTTCTGTGCTTAGCCACTTTTCTTATTTGTTGCCTGACAAATTTGGCAATGGATGACTAGAACAAATCCTGCTCACTAACACTCTTTCATGAAGACTCCAAATGCAATAAAAATACTAAAGTTCTAACACTATGTTACCAAAATAAAATGACAATAAATAATGGGCTTCATAAACCATAGCTTTTAATGAGGTTAAATATGTAGTAGAAGAAAACAAGCCCTAAAGAGACATTTTTGTTTAAATGACCTGAATTGAATGTACTGTGAAATGCAAACTGGCTTATTCAAGTTCTAATTTAGCTTAACTTAATTTTGGTTGAAGCCCACATCATTTATTAAATAATCCTTTACATTTGTTTGAAGTTCAAACACAACTTTATTTTTACAAACAGGAACGAATGCTGTCTTGTCTTGCTCATTCATGTATCTATTGCTCATTTCACTGGTTTGCTGGCACTAGCGTGTTTGGTTTTATTGCCAATTTCTAATGCTTATTAGTGTTTTTCTTTTCTCCATAGTTCAACTGCTAATCCACCATTTTGGATCCCTATACATATTGTGATCCCAGAGAGGCCGACTGAATGTGCAGTGTTCAATGTCATAGCAGGTACTTTGATGTCATTGCTTCATTTTGTGACCTCAAATGCTTCATTTTTGCTGCATCTAATTCCAAAGCTGCCTGAATAATTGTGAAGTTTTAGATTGGTATTTTTTAGCTCTCTTGCATTCCTTGCATAAAGCTTATACTATGCTAGGCTATTCAACAGTATAATCTGGCATCAACTAATAGTAACAATATTTTGATTTTGGGTTATGCACTTACGCAGTTGGAGTTTGGACTCCACTTTTACCTAAATAAGAAATCTTTTAATTATCAAATCTTGCAGCACATAAATAAATTAAATTAAACCAGCGGAAATCCTTGATCTGTATAAACTTTATGAACATCATGATCATATAATATATCCAGGACGGTGTCTAGCGTGTGGAACTAAAACTTGTTTCGCACTGTGTGAGAGTCTATTTTTTCCTTTTTTTATTTTTTATTTTTTTTATATTTTCTTTCCCAGTTTGCCCCTTACCCTAAGCCTAAACCTGCAGTGCGTTGCTGATGTCACCAAGAAGGGTCTCTTCCACTGTTTATCTTCACTATTTTACTTCTTTTTTCGTTTTTCCTCTTTTTTCCCCAAATTTTGTTCTTCCCTCCTCTCCGACGATTAACATCGACCACAAACAAAAATTGCATGAGTATGTTGAAACCCAGATAAAAAAATCGCAAGTGAGTATGTTTAAACCCAGATAAAAGAATTGCATGGGGGTATGTTGAAACTTAAATACAAAGAATCATGAGCTTGTTTCATTCACAACGCACATTTTCTCAATAATCAAGTACCAACAATTTCGGATATAGAATCAAAATGGAGAACCGTCACCATGGTCGCCATTGATGTTAGAGAAAATTTAGATCTGCGGATTTCTAGATCTCTTTGATTTTTGTGGTCGGATTCAAATTTGTGATTTTTTTGTTACAATCTCTGTTTTATGTTGTGGGGTATGGTTGTGTTGGTGAGGGAACAGGGATTTGGTGTTTGGGAGCAACGAGGATGTGGGGGTTGAATAAGGAGGAGTGCCGTCGTAGGCGGCGGCAGCGAAGATAAAGGAGATAAGAAAAAGAGATAAGGGGTGAGGGTAAATCGGTAAAGATGAATGAAGAAAGAAAAAAAGAAATCTCAAGCATCTAATTTAAAATTAATATATTTGACATTCTGTGTGACTTTCGCACCGTGCAAAATGAGTTTTCCTTTTGGAAGCTAGACACCGTCTATATTCAGACATGTATTAGTACGATAAAATTGTCTTTGAGTTTTGTAGGTACTTTGCAATGACTAATGAATAACCTTCAATGCCTTGTATCTTGTGTGTGACATTCTCATTAATTTCCCCATTGCGATGTAGTGTTGATCCCATATATTTAAACTTAGATTCCTATGGTATAACATGTTCTCCTATTTTTATTTCCAAGTCATATTCCTCTTGTGTCTTGCTAAAATTGCAATGCATATATTCTCTCTTATTCCTAATAAGCAAAAATCCTTTGTTTTCAATGTCTCTCTCCAAAGCTCAAGTTTAAAGTTGACTGCTTCTCTTGATTCTTCAGTCAAAACTATATGATTTGCAAAAATCATGCAATTAGGGATAGTCTTTTGTGTGTCCCTGGTAAGCACATCCTTGACTAGATTAAACAAGTAAGGACTTAGGGCTGGCTGAGTCTTGATGGTATCAAGTAAGGGAAGTTCTTAGTTTTGCTTCATAGAGTTCTCACATTAGTAGTTACCCTATCATGCATGTTTTGTATAGTCCAGGGAAAGAGTGATTTTCATATAGGTGAATTCAATTATTACAAGCGAGCAGGTGCAGCTATTTATAGCTGCCCCAAATACAAGTCTAGGTTAGCTGACACATCAGCTAGCTGTGGCTATCAGCTCGCTTTATCTAACTTTACTAACCAACAGCTGTTAGGAGATAAGCTACTCTGTTTTACGCTAATACCCCCTTGCAAGCTCAAGAGGGTTGTGATGCAGAGGAATCACCCTCAATGAGCTTGGATATAAAGGCATGGAAACATTAACAACTAAGCTGCTTGCTTAGGACCTTCTCTTGAATAAAAGTAGATCAAGTTCCATGTGTTTGGCCTGAGCATTCAAAACAGGAGTCTAAGCCTGGGCCATGATGCTCAGATTGTCCTAGAAGACTTGAGGAGCTGAGTGACTGACATCTAATTCAGATAGCAGAGTTTGGACTTTGGATCAAACTCCAAATGATTTTAGGAGCAGCATATCCTCAAATATGACAACATATTCTTTGACATTATTTGGCGGACTGTCCATTGATTGAGTGCCATTTGGATGTTATTTCTCATTTCTTTTTTCATTCTGAGCTTTCTTTTTTACCATCTGAGTTTCTTTTTACTTTAGGAAGCTTAATTAATTGTATTCATATCTTCTGCAGATTCTCCTCGTGATTCTGTCCAGTTTATTGAATGGTCCCCGTCCTCTTGTCCCCGTGCATTACTGATTGCAAATTTCCATGGGAGGGTAACTATTTGGACTCAACCTCCTCAAGTAAGTGTGATTTATCAATCTAGTTCCTAGAGCTTCTAAATGTGTGACTATCATATATTCTTGGGGATTTGACTCCTCTAAAGTGAGAAAGCGTGTAAAGCGAGAAAGTTAGGGCGCATCCACCCTTGATTTATAGTGGGTCAAGTTTATGTGATACTTTAATTAAAATCAATGGTGGATACACTTTTACTTTCTTACTTTATGCATTTTCTCACTTTGGAGGTGTTAAAAATCCTATTTTAGCATATATTTTAGTTTAGTACATTTGATCCATCATTTGCCCACCTCTACCCTTTCATGTTTTCTCTATTGCACATATTTGCTTGTGTTGTGTATTTGTTCCTCACATATTGGTTTGTCCATTCAGGGGCCAGCTAATCTTGTCCATGATACTAGCTGTTGGCAGCGTGAGCATGAGTGGCGGCAAGATATTGCAGTTGTTACAAAGTGGCTATCAGGGGTGTCTCCGGTATAATGTTTGCTAGTCTTCTACTCATATGTTCAAATTCTTTGTACATTTCTTTTGATAAAGTCTAGTAAACATTTTATTCGTCTAATTTTGTCCTCAAATTTATGTGATTATGTAAGGGATAGGGATTCTCTCTCTCTCTCTCTCTCTCTCTCTCTCTCCTGGTTTGGTTATTTTCAAACATGGCCTTTCAATGATTGAACCTCTGAGCAAATAGTTCAGGATTTGTATCTTATTATTTTTCAGTAAGATAGACCATAGTTTCAAATTATAACATGGATCCATTTATCTGCAAAAAGAACTCAAGCATTGGTTTACAGACTGTTAGAACCAAGTCTATTCCATGTACTATTGGTTACTTCATTTTTAATCGAAGTCAATTGCTTTCTGAATTAATAGTATAGGTGGCTTTCATCCAAATCAAGTGCTCCTGCCAATTCAAAGTTGACATTTGAGGAAAAGTATCTTTCGCAGCAATCTCAAACTTCAGGTCAATTATTCTATTTTCATACTTTTCTGCCCCTTCCCCCCCCCTCCCCTTTTTTATCTTGGATACACTGAGTGCAAGCACATGAGTAGGGGGATAGTTGTCCTATTGAGCTACAAGCTCATTGTGTCTAATTGATTGTGTTTTTTTTCTTTTTCTTTTTGTTCCACAACAATTTTGCTAGTTGCTAGTGGTAATATGTATGTCAAGAAGTACCACTTTTATATTGTTTTTGCTCTTATGCCAATAATTTTTCATTGTTTTAGTGACAAACCATGAGAACCCATCATCTAAATTTTTTTACTTGCCGTTTCCCTCACTTTTTGTGTTGGTGTTGTTTTTAATATTGCTTGTGATTTTGCCATTTTGGGAATAAAATTTCTTTATGGAGGCAAACCATGCTAGATGAAATGATTATCTGAAATTCATATAAACTTTGTTGGGCAATATTTTCATTTTCTCGTAGGGTAGGGGTCTGGGTGTCTTTTGGATGACCAAACCTCTGGCATCTTCACAACACTCGAGTCTTTCCTAAGTCCTGAATAATTGAGTTGGTGTCATTTGGAGTTACAAGCTCATTGGCACTTTTTGTTTTAATTATGATAGTCAACCATCTTAGTGATTGGTTGATACTTGGGCAAGTTTTATACTGTGATAAATGATGGTCAATGCTATGATGGCACCTGACATTACCTCTATTTGATCGCATATTTGTTCATTCCTTATATTTAATTTTTAAATTATCGTCAAATATATGTATTTAATAGTACTGTGCTTGTGCAGCTGATGCATGCAATTGTTTATTTTTTCAGCTAGATGGCCCAATTTTCTTTGTGTCTGTTCTGTGTTCTCATCGGGTTCAGTTCAACTTCACTGGTCCCAGTGGCCTCCTACTCAGAATGGCACAACACCGAAGTGGTTTTGCACTAGTAAAGGACTTTTGGGTTGTGGGCCAAGTGGCATTATGGCCGGTGATGCTATCATAACAGACAGTGGTGCCATGCATGTAGCAGGTGTACCAATTGTTAATCCGTCCACCATTGTCGTTTGGGAGGTTGCACCTGGGCCTGGAAATGGTTTCCAGGTAACTCCAAAGACAAGTACCACTTGTGGTGTCCCACCTCTTAGCCCTCCCAACTGGGATGGTTTTGCGGCTTTAGCTGCATATTTATTTAGTTGGCAAGATTATCTATTATCTGAAGCAAAGCAAGGGAGAAAACAGACAGACCAAAACCTTGTTGATGCTGTACCTCTATATTGCTCACCAGTTTCGAATTTTTCAGCATATGTGAGTCCTGAAGCTGCAGCTCAATCTGCAGCAACCACTACATGGGGTTCTGGTGTAACAGCAGTGGCCTTTGATCCAACTCGTGCGGGTTCAGTGATAGCTGTTGTGATAGTTGAGGGTATGAAGCTGATTCTGACTATTTTTTTCCCCTCAAATAAGTTTTCAAATTGCTCAGTGCTAAGGTGTGGCTTCATTGATAATGAATTTATTCGTCCATTTTTATTCGTTTTCCATCTCATTTTGTCTCCTTCCCTTCAGCCCCCCCTCTTATCTTTATCATTGTAATTTTAATCCAACATGATTGTACATGTTTTTATTACTGAGTCCTGATGAAGTTCATTTAAAATTTGTGAAGCTCTATTGTGTTTATTTTTGTTTATATAGATTAAAGGTAAAACAATTGTGAGAAACATGGGACTTAATATTTTCTCCACAGAAACTTCTGATATTCTTTCATTTCTTTTACTATTTATGGTTCTGCTCCATTATACTATTCAATTTAAATCATTATTTGTTATTAATGTGTTGAATGTCTTACACTTCTACTGTATATCTTTTGGAATACGGTTGCCCTTCATCAAAATGTCTTTCTCAATTAGTCTTCAGCAGTCTGTCTAGCTGCTGTCTTTGTTCCCACATTCCCCCATCGCCACAACACAAGTGCAAATCTTTACTCTATATGTGTTTTAAAGGTTACAGTTGAGTTGACCAAATCTGCTAAGAATCGGGGATTTGTAAGTGAAACCCATTTAAATATCCTTATACTGTGGTTAAACTTTAAAGCCATATGTTGCCATTTAAAGAATAATTAATCTGGTGTGAAACAAAATAAGAATCCACAATTTACAAGAAAAATATCTTATATCAAATGACTGATAATGGACTGGATAAAGCATTATCAAGTTCCCTCCTCCTTATAGTCATGCTTCCAATGTTTCACAATTCATCCTTGGGCATACTTTAAGATCCACTATAATAGAAGCACCCTGTACCTATCTTGCCTTCTTAGTCCTCTAGGTTGTGACAATACTGGCTGTGATGCTAGTTTATCAGGATTTTACACCAATAGGTTTCATGTGCCCAGATAATTGAACTAAAAAATTGATGTATAGCTGATTTTACATGTACAGGACAGTATATGTCCCCTTATGATCCAGATGAGGGCCCATCAATTACAGGGTGGAGAGTGCAACGCTGGGAATCATCTTTACAGCATGTGGTTCTCCATCCTATATTTGGAAACCCTACTTCTAGTATGGGTGGACAACCGCCTATGCAAACTGTTTGGCAGTCCAAAGTAGACCTGAGCATACCTCCAACAAATGATTTCAAGAACCATCAGTCACCTGCAATTGGAATGTCTTCTGATGTGCAAAAGGTATCTGAGTCTGTTTCTGATAAATCAAAAAGGGTCAATTTTGATCCATTTGATCTTCCAAGTGATGTCAGGGCACTTGCTCGAGTTGTTTACTCTGCTCATGGTGGTGAAATTGCCATTGCTTTTCTTCGAGGTGGTGTCCATGTATTTTCAGGTCCAAATTTTACACCTGTAGACAACTATCAGATTAATGTTGGATCTGCAATTGCTGCTCCCGCTTTTTCTTCAACAAGCTGCTGTTCGGCTTCTGTTTGGCATGACTCTAGCAAAAATTGTACAATATTGAGAATAATTCGGGTTCTTCCTCCTTCTATTCCCATTAGTCAAGCAAAGGCCAATTCATCAACTTGGGAGCGTGCCATTGCTGAAAGGTGATTTCTTTAATCCTTTCTAGTAAGTCATATATTAACTGTTTCTTGATATCCTTATAAATTTGCAGCTGCTTCAATTATTTGTCTACTTTGTCTGGTAATGGAAGTTGTGATTTGTTTCTTTGTCAATGATTTACATTACATGGCTTGCCTGGTGCTAACATCTGTTAGGAAAACAGGTTTTGGTGGAGCCTTTTAGTTAGTGTTGATTGGTGGGATGCTGTGGGCTGCACACAGAGTGCTGCTGAGGATGGTATTGGTAAGGCAGTTCTTATTGTCAATTCCTTTGGCTATTTTTGTTTATGTTCAAGTTTGTGCCAGAGAATGGAGAATATGATTTCCTATTTCATATTTGTACTGTTAGAAAGCATCTGTGCAATATACTGTGGTGTTGGTTCTGTGACTTCTATCTATTAACTAGTGACATTTTTTTTCCTGGTTTCAGTTTCACTCAATAGTGTTATCGCAGTTTTGGATGCGGATTTCCATTCTCTCCCTTCTGTTCAGCACAGGCAACAGTATGGTCCTGTATGTTACTGTTGAACCATCACCTTTTCAGAATTTTGTCATTGGCTTTTATAGTTTACTTCAATTTCCTCTTCAACTTTTTCGGTTAACTGAGAGTAAATTGTTCCATATTAACTGTGGGTGGAATGTTTCATGGATATATCATAAAATGGAATTTGGACCCAGCAGCAAATGCTTGCTATTGACAATTTTGTATTTCATGTTGAAACTTAGACTTTTACTATTTGGCATAGCCTCTACATCATTGATTATACTCAATAACTTGTGGTCTTGTGGATGAAAAAATGCTCAGTTTGGTGTTGAAATTTTCGTTTTCATAGTTAATATTTGATGTGAGGTTGCTTCACTCCTATAAATTTGAAATTTTGATTGTTCATGTGGATTTCAGTCTGAATATGATTTTTAAGCTTCCTTAATATCTGCTAAGGATTACTTTTCAACCAAATTTGCACCAGCCACTCTTTTACCGTGTCTTTCAGATAGGATAATTAATACTATTTTTTATAATTAAACAATATGGACTTCTATTTGTTTCTTGACTTTTTCTGTTTATTTGTATTTTCTGATTGGGATGTGATCGTGTTTGTGTTGCTAATTTGTTGGCTAAATGGACTTGGCTCTAAATGATTGCAGAGTCTAGACAGGATAAAGTGTAGGTTACTGGAAGGATCAAATGCCCAAGAGGTGAGGGCGATGGTTCTGGATATGCAAGCTAGGTTGTTATTGGATATGCTTGGGAAAGGAATTGAGTCTGCATTGATAAATTCTTCAGCTTTAGTTCCTGAGCTATGGCAAGCATCAGGTGAAACGTTATCCAGCATTGACCCTGAATCAATGGCTGTTGAACCTGCACTAATTCCCAGTATTCAGGTATGCTTTGGTTACATTTTGAAACTTGATTACCTAATTTTTCTTAATCAGAATATATAATAATTTTGTTGTGGTTATCTGCTTGTCTTTCTGCATACGACTCATCTTTGTTATGTTTGGTCTTTTGTCATAGCACAGTTGTCATTTGTGGATAGTGGTACTGAATCTCTGACCCTAAAATTGCTGTAGGGGATGGCTGCTATACCAAGATAATGAAATATGCAGCATGCACCATACACACACACACACACATGACCGGTTTTGTTTTTTTAAAAGCACAAAATTACTGGCATGTCATCATTGATAATAAAAATATTTTCTAGACATACAATCACCATCAATATGTGGCATAATTAACAAGTCATTAACCACGGAAAGTGACTGAAGCTGAGGCTGAGACCAAGGAGGTGCTCTGTTGGGTTAATTGTGACTGATATGATTATGATGCCATGGTAGCACCAAAGAGGGGTTGAAATTAAATTATCACCAGCAAAAAGGATCAGAACAGAGGATGTGTCTCACAGCAAACAAAAAGAATGGTGATAGCAACTGAAATTTACTATAGAATTGAAAGGGAAATTCAAAGAAGAAAACTCTGCCCTGTGGTAACGGCCACCCAACAATTCCCAAGAGATTGCCTACCCTCCCTCGTACCCCCCTCCCCTCTCATATTCTTGCCCATCTCCTCTGATTCCCGTATGTCTCTTTTCTCTCTCCTGCTGCTAGGTGTCATATTTGTTATGATTCTGCATAACAGAATCCTCCCCGTAGGTGACATTCTCCTCTTCTCTGTTCTACAATACACTTTTATGGGAGGCCCATTACGGGGCCCATCAGATTGAAACATGGAAAGAGCTGCAGCAAAAAGGGGCAGGCCTTTCTGGGAGTATGAAATATTAAACTCTATCTAGAAGAGGGAGCATATTCAACATAGGTTATGTGATGCTAGAAAATGTTATGCTGTTGGAAAAGCAAGGGCTTTGCCAGAAAATGCATTGCCTGGTGGAGATGGTTGGTACAAAGGAAGAAAGCAGTAACTATGTGCATCACTCTTTGAAGAATGAAGGGTGGTGAATTTTATTTTCATTTTGATGTGGGAAAACTTGTGAAATTTCAGACAGGGTTAAACATCTTTTTGGTCCTTACAAAAATGAAAAAGTTTGATTTTAGTCCCTCCTTTTAAAAATGTCGACTTTTCGTCCTCCATTTTTCAATGGAAGAAATCTTCCCGAACCCAACTTCGCTGCCGACAGCCTCACTCGCATCAAGAAATTGTCTTGATCCATCTCTACATGTAGCTTCCAGATCCACTCCACTACATAGCAGTCCTCAGCTGCCTTCTCAAATCTTCGTCTGCCAATTTTGTCTGCGGTCCTCTATAGAAGCTTCTGTGATGAGTGGATCCATGACTCTGTGATCCAGTGGGCATTTTGATGGGGTGGCTTGTTGGTTGCTGGTGTGGTGGGAGGTGAAATTGATGGAGTTGAGGACAAGGAAGAGCAAAATGGTGTGGTGTTATGATTTTCGTGGCAATAAAAAACCACCACATTTTATAATCCACACTCCACACTTTTTAGTCCATATTTCACAGTATGTGACAGCTTTTGATCACCAAAAATTGAATCACCGTCAACTCCAACTGACAGAGTTAGTGATGGGTTTATTGTGACATTTTTTAAAAGTTGTGGGACGAAAAATAATGTTTTTAAAAGGAAGGACTAAAAATGAACTTTTCCATATTTATGGGGGCCAAAAATATGTTTAACCCTTTTAAAATTTACCCCATAAATCTGATGAGTAATTAAAAAGAGTGGCTAAACATGGATTTAATTGTCTGTTAACCATAGCAGCTGTGATGTCTGCAATAGTGCAAATTGTCTGATATCTGCTCACGAACCAAGCTGCTATTGCTTCCCCACAAAATGTTGACCCAGTGCCACTTCTGTTGCAGCACAAGCCGCCATAGCATGCTATTAGTTACTCGTGCAATCGTTATTTGTACCATTATGTTCTTACAGTTACACACAAGCAGAGATACTTGCATATAATTTTTGTAGATAAAGATGCTTGTCAACCTATTTGTACTTGTCTATATATTATTAAACCCCTTAGAGCATTTACAATGCATAGTTGCTTATCATAATTTTGTGGGACTTATAACTTCCTATAGATTTAAGCATTGTAAACAAAAATTTACTCCAATGCTAAGATTGCTAAAGTGAGTGTTTAAATTAATTTTATGGGTCCTACGATGCTTGAAAATGTTATTTATGGTATAATGTTGTTAAGCAACTCCACGCCATATGCTCCAGTGGGAAAAAAAACTTAAGCAAGGTTGCTTAAAGTTAAGCAACTCATGTAAGCACCCCCAGTGGAGATGCTCTTAGAGGTTTTTCTTTGGCTTGCTTCTTTCATGTGTTATTCTTTGGTGTTTTTATACATTTGATTGTATTTATTTCTATAAATTCCTGTTTCTTAATTGTTTCAATGTTTGCAGGCTTATGTGGATTCAGTTCTTGATTTGGCTTCACATTTTATTACTCGATTGCGCCGTTATGCTAGTTTCTGTCGCACATTGGCAAGTCATGCTGTGACTGCAGGCTCTGGGAGCAACCGCAATATGGTTGCTAGTCCTACCCAAAGTTCAGCAACACCCGCAACAAGTCAAGGTCAATGTGCACTGATCAAATGTCAATTTTTGGTGTTTAAATTGATCTGCATATCCCTTAGTCTATATGAATTTTTAAATTGGTGCCTATAATTTAAAAGCTATGAATGGGTTCCTGTAGGTTCTGTTGAAAGGGTTTACTGTAATGTGTTGTATGTCTTGGATGACCACACTGAGTCTTTATTTATAATAAGAACATAGAATCTAATAATGAGTAATAAGGACTAAATCCCTAATTGCTATCTAATAATAAATAGGAGAATAGAAGATAATCTCACAGGTTCATTTTTTTTTCTTGATGTTCAGTCAATCCTCTTGTTGAAATGTTTTTAAACTATAGGGACCAATAATCTTCTAACTTGTAAAAAGATAGAAACAAAGATTTTATTTTCTGAATTGGCTTCAGTGAGTATACCACTCAGCAAGGTGATCTATTTATTTATAACAATAATGATAACAAGGATAATAAATAATTACTATTTTCAAGAGAACAATTAATATATCTCTATTTCCTAAGATACATTAATCTTAGGAAAACACATATTTGAAAGAGTAATAGTTATTAAATGCTCCTATTTCCGACAAAATGACCTGTTTAAAAATTCACAAGCGGTATAATGATCAACATATTAATATAACCTTTTTTATGATAATTGTTTAAAGAGAGAAAAACAAATGCTAGAGATGTTGAAACCGTGTGTCTGAACTACAAAACAAAGTCCATTTTATATAGACTTAATGTAATAAATACAAATAAATATAGGAGATACTATTCTTATTTACATGATACGATATGCCCTTAATTACAGAGATCCTAATTAAATAAGGAAATATATAAAAAAATATAAAAGAAGAATAAATCCTAAGAGAACATCTAAACCTTAAAATATTCTTAGATATAAAAAAGGATATCATCGTATATTTTTCACATTTTCTACCAATTATTTTTTCTTATATATAGTTTACTTTATCAAATAATATCTCCATATGGATATCCTGTGGCATTCATATGCTTGTTGAGTAACTTTTTATGGAGAGTGGAAAAAAGAACTTCTAGGTAACCTCGGGGGTTGGTTTTTGCTTGACCCTTTGGAATCTTGGAATGCCCAAAGTGCTCTGACCGAAAAAATATGTATAGATCCATATATATATATATATATATATATATATATATATATATTTTAAGTTATTTCAATCTGGTGTAATTATGGATTACTTTAGAATTGTTGAAATATGGGGATTTTGGTATTGAGGGAGGAAAATAAGAGAATATAAACTATTTGTAGTGTATTAATGTGATCTGAGGTGATAAAGAAGACAAAAACACTAATTATGAAATATAAGGAAGTGTGGAAACTATTATGAAATAATCTAATGCACTCTAAATTATTCTAAAGAAACAATCTGGATAGCATAGGATATTTTTATATATTCTAACAGGAAGCATAGGTTTGGTGCTTCTCTTTAAATGGTTATAGAAAATGATCAACTATCCTACCATGAAAAGCTTTAATCATTTTTACCACTAGCTTGATGTTTATTATTTGTTTTACAATTTACATATCAAACTGAAATAAAAGCAATATTTTCAGGAGGTCAAAATGGGACCAGCAGTTCTATGGGAAGCGCCCAGTTGCAAACCTGGGTGCAAGGGGCTATTGCCAAAATTAGCAACACAACTGATGGCGGGTCCAATCCAACTCCTAATCCCATCAGTGGCCCTTCAACATTTATGCCTATTAGCATTAATACAGGAACATTTCCTGGAACACCAGCAGTTAGACTTATTGGGGATTGTCATTTCCTTCACAGACTTTGCCAACTATTGCTCTTCTGCTTTTTCTTTCGACGGACACAACTTCCTCGCTATATTAATAGAACATCTGATACAAATATACAAAAGCCTCAATCTAATACTCCTGCTCCTGGCAAGGTGGAGGAGATTGCAAAACCAGTTTCAGCTGTGGTTAAGTCAGATGATGGTCAGACAGGTCGAACTGGGGCAAAAGGAGCTGAAGAAGTACCCTCTGGACGTTCAAGATTGGGTAGTGGGAATGCTGGCCAGGGATATACGTTTGAAGAGGTATACATTAATAAATGTTGCAACTTGTTTAAGGCTCCTTTTTTCCCCCTGTAATTTGTGTCCTACATAATGTATTTTGAAAGTTTTGCTGATTCCTTTTTATCTTATGGAGCTTACAAGTTACAAAGTAGGCTCTGTTATTTTAATTTTCTCATTGCACGTACAATGCAGGTCAAGGTGCTTTTTATGATGCTTATGGATCTATGTCGCCGAACAGCTGGGCTGCAACACCCTTTGCCAGTTTCTCAAGTGGGGAGCAACAACATTCAGGTTCGGCTGCATTATATTGATGGGAACTATAGTGTACAGCCAGAGGTTGTGGAAGCATCCCTTGGTCCACATATGCAGGTAACTTTACGGTTTTCTGTTTCTTTTAAATTCCTTATTTTCTGTTACATAGGATTTGGGTAGATTATAGATAACAATCTGGTTAGATTTATGGTTTTCTAATTCTAGTATTAAAACCTGTATTAGGAAACCATAATTCTGCTAATCTTGGACATCAATTGTACTCCTTCAGTACCCCTGGTACTGTCTTATATATAATATTTTATCTTTGCTGATAAAAAAAAGGTTAATTATTAAGTAGGGCTCGACAGTGGCTCCGTTACCATTTTTTTCATGAGGGTTCCTTTATACAGCTACAGTTTATAACTGAGTTAGTGATACAGCCCAGTTTGTTAGATTATGTAGGTACTGTTGATCATTGGAAGTTAAACTTTGTTATCTTGATCTGTTTTTCTTTCTTTTGATGTGTAACCTTTCGGTGAATGCTCCCTGTGAAAAAAAAACTAATTTGCAGAATATGCCCCGTCCCAGAGGTGCAGATGCTGCTGGTCTTCTTTTACGCGAACTAGAACTCCACCCTCCAGCAGAAGAGTGGCACAGGCGGAATATGTTTGGTGCACCTTGGTCTGATCCAGAGGATGTGGATTGTGCTAATGATACACCTAAACTTGTTAATTCTGACCCACTTGATTTTAGCTCATTGGAGCATTGTGATGTTTACTATGGAACTCATCGCTTATGGCCAAGGAAGCGCAGGATGTCTGAAAGAGATGCAGCTTTTGGGTTGAATACTTCTGTGGGCTTGGGAGGTTATCTTGGTATAATGGGTTCTCGAAGAGATGTTGTTACTGCAACGTGGAAAACTGGCCTTGAAGGGGTCTGGTACAAGGTTGGAAAAGAAATCTTGTTCATTTGTTTATATTGTTTTCTGTCATTCTGTACATCCTTGTATGCAATTAAGGAATTCAAAATGATTATTGATTTTTATGTGAATTTGAAGATCTGGCATTCATTATTGTAAGAAGTGCATGTGGTTCATATATCCCACGCTAAAATGCATTTAATGTTCATGTGTATTAATGCCTAGGAAGTTGCAACGCTTATTATAATCCTAGGGAATGGTCTCCACCAAGTCTATCTAGAGTCTATAAATAAGGATGATAATGATATGCAGAAGAATAGATCGAATAACATTTGTGATTTGGCAACTGATGGAAGTATTGATGGTGACTGAGACATTAGAATAGGAGCAGTGTCATCTAGGTCAATCATAATTTTGTAAATGAATTAATGAAATCATTCAAATGCTCATGCTTTTAAATTAGTAAAACACACTTGTGTGTATTCTTCTACTTGATCCCTTAGTGTTTTTTACTGCATGATTCATCTTCTATTGGTGATTTTGTTTACTTCTATGGTGCAGTGCGTAAGATGTCAGCGGCAGACTTCAGCATTTACTTCACCAGATGCTACTGCTTCTCCTAGTCAGAATGACCGGGAAATGTGGTGGATCAGCCGCTGGGTACATAGCTGCCCAATGTGTGGTGGAACATGGGCTCGGGTTGTATAAATAGGTGCATTATGTTATTATAATGTCAGTTTCACTCTATGGAAAGAGAGACATGGATATAGTTATGGCTAACTGTGAACTGAATCAGATGGAATGCTGACTGGAGTTGTACAGGAAGAAAGGTGTGGATTTGCTTGACAAGGTGGGTTGTCAGCATGTTGTTATAATTGGTGACCATACCATACTATCTTGGACTTGAGTTGTTTTCTTTCTGATCACTCTGCTGGTTTCAATTTTTTGAAAATCAGTTTCAGATCTCAATTTTTTTGGGAGTAAATTATTGTCAAGTCACTCCACAAACAGTAGGTGTTAATTTATCCATGGAGTGGTTTATAATTAACTCCTTGCAGGTATGTAATATCCCATTTAGACTTCTCTACTTTTAAAATGACTGAATTGCATCCCATCACAAATGCATTGGAAATTTTCTTTTGCTTGAAGAAAAATAAATGACTTATGGAAAATTGTTCAACAACAATAATAGCCTTATCTCACTGGGTGAAATTGGCCACAAGGAATACAATGCCATTGGGTTGGGTTAAAAACCAAATTCTTAGGGATACTATTTATTATGAGATCTCTTTTAGTAATCTTAGTCTCCTTATACCTTTTTTCATATGGCTAAAATTCATGCGGTCTATTCTCCTCACCGGTGCCTCCAATGGTTTTATTTGCACGTGTCCTAATTAACTTAATTGATTTTTTGCTATCTTTTACTTGATAAGTGTAACACCAATATCTCCTTGTATACAATCATTCGATATGCTGTCTTTTCTTGTGTGGTGTTACATCCATCATATCATACCCCTCTTAGTTACTTTAACTTTTTTCTCTTGGTAAGTACTTTGTAGTCACAAATAACCCTTGGTGCCATTTTTTTAAAAATTTATTTCTATCATCCAATGTGTGACATCATTAATTTTCCTATCATTTTGTACTATTGATCCCAGATACTTAATCATAGAAACTTTTCGTATGATATCTGTAATTTTTACCTCCAAGTCACTTTTCTCTTATTTCTTGTTAAAATTGCAATTTATATACTCAATCATATTCCTACTCAAGTGAGAACTCAGCCGATGCTTATTCCCCGGATACCATCATTGCTTCTTCTCCGGGAAAAGAAGTTCGATTCCAATGTCTGTGTCTAACACTCAAATTTATTGTTTACCTCTCTTGATTCCTCAAACAAAACTATATCATTTGTGCAAAGCATGCATTTAGGGTTTATCTTTGTGTCTTCCTTTTAAGTACATTCAAGTGTGTTTGCAAAATTGTTCAACCTCTGTTGAATGTGGATCCAACAAATTTTCCCTGTTGCGTTTAGTTTCTTGGAAAGTGGGAAATGAATGCTGAAATGTGAAAACAAACACTTGTAAGGCCATGTTTTGATGTAAACCTATCCCTATAGGAAAATCTTTAGTTTCACTTTCTAGAGACACTGTTAGTTACCCAATTAAAAGAATTGGTTGTGAGCCTAAATTTGCTTAATTTTAAAAGTAGAGACTTATTGGTTAAAAAGCTTGATTGGCTAGATTATGCAATTGCAACTAAAGGGACTAAAATTGTAACAAAGCCTAATTGTTGTTTTAATTTTACTGGTTGGCAGAGTGTTATTTATACACCTTAAGGCATGATGGTCAAACTAGTATTGGATTTACATCAGCAATCTCCAGCAAAGCTTCTAAATAATTTTGTGAACTGTTAATGCTAATAAGATGGAAGGCCAAAATTACTGGTGTTTCTGGGATGAATGTTTCATCTGTTTCAGGATATATTCTTATTTCTTAGTATTTTGATCCTTTGCAAGATAGCTTGCTTGAAATTAGATGCACAGTTGCCAAGTTACAACTTAGAACTCTACTTGACATATCTGTTTTGAAAAATCAGTTTTTGATGATTTTTGTGGCATTAACTTTTTGTCTTGCGCTTTACAAACTATTCATTTGTTAATGCAGATTGTCAATCCTCTGTAAAAACACTACTGTATCCTGCATGCCGTTTCATGCATGCTGATGTGGGTGGGTGCCTTGGAAGTTGCTGCTGGTGAATCAGAGATTAGGAGCTTATATTAGTTATCACTCATTAATCCCTGAGAAATCTCTGCATAAACTTCTTAGACTTTCTTCACTTGTTGGTTGTATTATGTAAATGATGCTCACCATATTGTTAGATTAGCTTAGCATCTTATTATCTTCTCATGATACCTGTAAAATTGATGAAAATTTTTGTTTTATTTCATTGGGGTACATCAAAAAAAGATATAATTCAATGTACTGGACCTACATAAAGCCTGGATTTTTGTATTTGTCCTCACTGCTTAGGGGTCTCCTTAACATTATGCAGTTTTTAGTAAATAGTTTAGACGAATGGCTGCTTTCCTTGTCTTGTACAGAGCCTAGAAAGGTAAACGCAGTGTTTGACACCCACCTGCACATACAGAACCAAACGTGATTATTGCCTCCCATCCTTGACTTTCCCCCCTCTGCAAGCCAAACATTATTATTATTACTATTCTCTTTATTTTCGTGGTTC

>GmMED16-2

TTCTTTCTCTTGCATCTGTCGCCTTCAGTTGCGACTTTCCGCCACTTGCACCCTCTCTTTCTCTTGTTTCGTGTATAAATAAACATTATATCATAGCCTCTTCTTTTGGGTTATGAATCAAATTGCTGCTATAAAGGACCCAGAGGAGGGTTCCGAGAAGAAGAAGAAGAGCAATGAAGCAATAAGTGGCGAAGAAAAAAAAGAGGATCCAATGGAGCAGGAAATGGAATCACTCACCCCTGCCACCGTCTTTTGCATTAGGCTCAAGCAACCCAAGTCCAATTTGCTCTACAAAATGAGTGTCCCTGAAATTTGTCGCAATTTCAGGTATCTCTACACTTGCATGACTTGAATTTTGCGCTATCAGCCTTGAACTTTGTGTTTGAGCAGTAATAAGTAAACTACTAATTCTTGTTATATCGGAAATAGCTTCCTTTAATCTTTTGTGTCACTGAACTTGGGATTACCATGTCTATAGTAGTAAGATTTTTCAGTGTTTTTATCATAATTTAGTTTATCTGTTGACTTCAAGAGTTGGCTATGTAAGTTTGTATCCACTAACAAAGCAGGGGAGCTAGATCAAACTAAAGATGATCGTGAATCGTGATGCTGTTTCAATAAGGATAGACACCATAGTCAAGAAATATGTACCTATTGTTAAACCAACCTCAGACCTAACTCATTAACATTTGGTGGTATAGAATAAAAAAACATTGATTTGTGGTCTGCAAATCTGTAGTGTCTTATTAGTATTAATTCTGTTAAGTAGTGTTCCAGTCCAAACAGAGAATGTGACCTACAAAAAGCCTTTGACTACCAGATAGGTTTGTCAAGAAGGAGATGCTCCTGATTAGAACAAGAAAAACTGTAAATTGGATTTACACAATGCATACTAGAAGTGTTAGTACTTAGTAGTTCAAAATATTGCATTTAGATAGGATGACTGGCAAATAGAATCAAGTGCAGAAGAGGTTCTTAATAATGCTTCATGTCCGAAGGATTTCCATTTCATTTGACCACTTTGGTGAAAATATCCTTTTATGCCTTCTGGATTTTGTATAACCTTTTTAGTTTAGGATACTTATTTTGGAGGATACTGGCTCAGTGCATTGCTTTCCTTTGAATAAAATCATATTTTAGAAAATAAAGAGAGGATATCAATGCCTTGGGCAGAGAATCATGGAAAAATATAAAATTTGCAATATAGTTTTCCTTGAAAATGTGGATTGAGAAAAATTCCGGTCTGTCATCTGCTAAATTTTTTCAAACAAATATTATTCCTGCATATTTGTTTGATTGTTCCAAATTGTGAGAGGAAGATTTGTGGAAATTGTACAATCAGTCAAGTCATTACCTTTCTGTGTTGCTTGAAAATCAACATAGTAAACAACTTGTCTTTAGTACCCGCATTGTTTTCATTTCAGTGGTACTAGTGCCATTATTCTTACATGAGGGAACCACATTAGTTCATGCTTATGCTTTTGCTATCATTTTAACAGGAGTTTTGTTTTAACATCTTTTATTGTCTGAATTATAATTAAACTGTTTTATTTTTCTCCATATCAATATAAGCTTGAACAAACTTCATCTCTCACTTAAATATTTATATAAGCATCTTTGGGAAATGGGAAGTTGATTAATATGTAGATCAGTAGATGATCAACTTCCTTGGATGTACTTCATAATGCTGTGGAGTGTGGACGCTGGTTTAGCCAGTGCTCCGTACTTCGATGGGCACACACATATTATCTCACAAGTCACAACAATTCCTAAGACAATGTAGGCTGGTTTTTCATGGTTGATAGTTTATGAATCTGTTGGTGTAAAATTGGGATTATGTATTTGATCTTCTGAGCATATTATTTTATGTCCTTTATGGTATTCTAATTCTGGCGCTGTCCCTTTTTAAAAGAATTTATTGATTTTTTTCCTAGTGCTGTTTCCTGGTGTGGAAAACTGAATGCTATAGCTTGTGCTTCTGAAACGTGTGCAAGGATTCCAAGGTTAGTTTTATCCAGTAGTCATGTTTCCTGGGTGAAAATATTTGATTATGTGCTAAAATGTTTGTTTAAAATATGACTATTGGTTTGGATAACTGAAAGTTGATGGAAAAATTTAAGAGCCAAAAAACAGATTAGATTATTTTGAGGGTCCATTGAATTTTAGCATTTATGGATGAATTCAAGAACATTTGATTAATGTTTCAAAATTCAAACTTTCCCAATTGCTTTTATTCTATTGTGATACCCAGGCCAGAAGAAGCACTAGGAAAAGATATTATGGGATATATCTTTTTGTATATTTATCTAGGTGACTGTCTCTTGGATCTTCTTTTTCTGACTGGTTTACATAATAATTTTCTTGCTCTTAACCAGTTTCCTCATTTGTTGGCAAATGAATTTGTCAATCGACTTTAAGTTTTTGTCCTGCTTGGTTTCTACTTTCTAGTAATATTGTAAAAGGTTTATATTATATGCATTTTATAGGCATATTCATTTGTTAGACAGACCCACAAGCAAATTTGAATGAATAGGGTTTATGGCAAAAAGACTTGTTCACATGCTAAATAGGCTTTGCATAATGATGTCCAATGCTTATATCATTTATGTAATATGCTTCCAATATTTGTTCAAGTATTAAATTTATATAGACAAACAGTCACATATGATGTCTAACCCTGCTCAGTTGTGTGTACCCAGTAGTTACTTTAGCTTGCTGACACCCCTGTATGTCTGCTTTTACTGGCAATTTTTGTAACGGTTATTAATGTTTCTTTTTTCTAAAGCTCAACCGGAAATCCACCATTTTGGATCCCTATACATATTGTGATCCCAGAGAGGCCGACTGAATGTGCAGTATTCAATGTCATTGCAGGTATTTTGATGTCACTGCTTCCTTCTATAATCATGGATGTTGCAGTTTTACTGTTCTGTTGGTGTTGTTTGATCTTATTTTTCACTCTGGCAGGAATGCCAAAATTGGCTTAACAATGGTAAAATTAGGTGTTTGTCTTTTTCAATTTCATTATGATAGTAATGCAGTCAAACACTGATTTTAATAAACCCTTGGTATTATTCTTCTGCGGATACTGATATCAGTGTACTATAAACACCTGTGGGTGGATTTACAATGAAAGGTAAATACTGAAACAAAAACAGAAACAGATGAAGTGGGGTATACGAGAAACCCTACCCTCTCCTTTGATTGCCTTCTAGCAGTCTGTTTACTGATATCCCAACCCCTTCTTTGATTGCCTTATTGGCAATCTGTTACACCAATTTTTCCTTCCTGAGATTTCAATACCCCCTGATTGTCTTTTTTGCATCTGATATATTCCAAGGTCCAGATCCTCTGTGCCCCTTCCTAATATAACCCTTAGTAATTGGTGATTTCCTAGCACATTACCAGCATAAAGGTTCTGCCAAATCCATGAGGCTGTTCGACATTGTTTTCTTGCATAAACTAATTATGGATTTGGGAATATAAAATTCAGGTGTAAGGCCACAAGTTGAAAACACAGAAGAAATCTTTTAATTTTCAGAACTTTTGGTCTTGGCACTTGTATATGTTGAATTAAGCCAACTCTTGGAGGATCTTGATCTGGATAGCCTTTAAGCATCATTTCAGATGTTATTGACACAAGTGTTCACACTTTTTGAAGTTAAATGATTTAATGTTTGGGGTATCAAGTATCAACGGAAAGGTCTGAAGAATTTCCTTGATGACAATATTCAAAAAATTGCCACCATTATCGTTATTATCATTGTAACCAATTTACATGGAATATTTTTCATAAATTACATCTCATTTGTAATATTGGTAAAACGAAAAAGTAGTCAAATTGAATCTGAACAATTAACAATTAGCTCATCTATTCTTTTCTTCTTGCCCTCAAATTGGTGCTATTTCTTGTCTTTTTTTTTTTTTTTTTTTCTGGGCTTTTAGTTTCTGAACTGAACTGCTTCACTATCTATGTATGTGTGTCTATATAAATTTTGAAATTTGATCAGTTGAACTGTATGCCTTCACAGATTCTCCTCGTGATTCTGTCCAGTTCATCGAATGGTCCCCCACCTGCTGTCCTCGTGCATTATTGGTAGCAAATTTCCATGGGAGAGTAACTATTTGGACTCAACCGTCTCAAGTAAGTCAGAGTTACTTAAGTAGATACTTCTACAATCACAATTTCCTGCATAACATGGTTGTGTAGTATAATTGCAAGTGCTCTGTCATTGACTTTTATGTGGCCATGGATTTAATTATTAAGTTTGTTGTTCGTCTGTAAAATAATACATGTTACTATTGCTAAAACTTACCCCAAACTCTATAGATGTCTATTATTGAGATGGTTTTTCTAAATCACAGAATGGAATCTTTAGCTATGTATTATACCCTAGCTCTACAAGGAGGTTAATGAAATTGACTTAGCGCTTGTGTGTGCCTTTGAATCGATCTTTTGTCCAACTTTAACCTTCTCTTGCTTCTCCATTATTCATATATGCTTTTCTTGTTTATTTGTTACTGATTTCTTGTCCATTCAGGGGCAAGCTAATCTTGCACTTGATGCTAGCTGCTGGTTGCGAGAGCATGAGTGGCGGCAAGATATTGCAGTTGTTACGAAGTGGCTATCAGGGGTCTCCCTGGTATGCAAACAATGTCTCTTCTATGTGATGTTTAAATTTTCTGCATATCATTTTTGAGGAATTGTTATAATATTTTTTATTTATCCAATTTTGCCCTTAAACAATTGTTCCTAAAAAACATACTCTTTTTAAACAATTGTCTCTTAAAAAATTCTATCTCTAAACATTATTCTCTAAAAACTGTTTATGAGAGAGCTTTTTTAGAGATAAATTCTTAGAGGGGGTGAATTTTTATTTTTTTTAGATAAGGAAAACTTTGTTTAGAGAGACAATTTTTTTAAGCTAAATGGGTAATTCATATTTTCCCTAGAAAATCCCACAATTTGGAGGAACTTTTAATTGTTAACAAAACGTGAAGAGAAAGATTTTGTAGGATTAGTGAAAAATCCCTAATTCCCCTTTAGTCACAATGTCATTGCATTGTGCTGACTTACAACTTATACTGTAGCAAGCTTGCACACACACACATGGATCCTGTGTAATTCAATATGGTGATTCTGATATTTTTTAATCCAATTTAATTGTGTATTTGAAAAAATCAGTATAGGTGGCTTTCATCTAAACCAAGTGGTCCTGCCAATTCAAGGTCAACATTTGAAGAAAAGTTTCTTTCACAACAATGTCAAACTTCAGGTATTTTTTTCCATTTGCCATTATGATTGCCTTTTTTTCCAGTAATTTTTTTCACTGTAGCAGTTATGAGATATCAAAAGAAATACTACTTTTGACATTGTATTTTGTCATTTTTGGTAATATTTGTTTTTTTTCCTTTTCCAATGAACCATCTGAAAAAGCTTAAGCTAAAATTATATCTTCCACGGTGCAATCCAACAAGGATTTTTCATGCACAGATTAGATGTTGGAAGAAGAGGGACTTGTGTGTGTGTTGGGATTAGGGTTTGAGGGTCATATGGTCCATATGTATACTCTTTGTAATTTAGTTGATCATAGCGTATGATTCTGTTTCACCAGAGGACACAAGTCTATCGATTTCTCTTATTATTTTCCACTTTACATCGGGTTATAGCAATTTTCCATTTTGCTTTTCTGATTTTGACAATAGAAGTATCTTCGGGAAGACAAGTAATTTGAAGTGAAGTACTATACTTAATCTTCCTTCCACTTTTGATTGAGTTTGGCTTTTAATTATGATACTGAACCATCTTAGTGTCTGGCTGATGCTTATCAAGCCCTGATTCTTCTAGCGAGATGTGCTATATCGGACACTCATGTCATGATGACACTTGTAATGTGTCTATGTCTATGTCATGTTCTGTTTCCGTGCTTGTTAGTCTTAATATTAACACAATTACACAATTTGTATGTTGACGGTGTAGTGTTGACAACCATGGAAATGCCTGAAATTAGAACTTTTTTCCTCTCTTTATTTGATTGCTTATTTCAGTTATTTTTTTAATAATTCGTTTTACATGTTCATTTTAATATGTATATTAAAATTGCTGTATGCTAGTGTGTCTGACACATGGGTTTGTTTTGATTTCAGCTAGATGGCCCAATTTTCTTTGTGTCTGTTCTGTGCTTTCGTCAGGCTCTGTTCAACTTCATTGGTCTCAGTGGCCTCCTCAGAATGCAACACCACCCAAATGGTTTTGCACTAGTAAAGGACCATTGGGTTGTGGCCCCAGTGGCATTATGGCTGGTGATGCTATCATTACAGACAGTGGTGCCATGCATGTGGCAGGTGTGCCAATTGTTAATCCATCCACCATTGTTGTTTGGGAGGTTATGCCTGGGCCTGGTAATGGTTTCCAAGTAATTCCTAGAACAAGTACCAATAATGGTGTTCCACCTCCTCTTAGCTCACCCAACTGGACTGGTTTTGCACCTTTAGCTGCATATTTATTTAGTTGGCAAGATCATCTATTATCTGAAGAAAAACAAGGGAAAAACCAGACAAACCAAAACCTTGGTGATTCTATACCACTTAACTGTTCACCGGTTTCAAATTTTTCAGCATATGTGAGTCCTGAAACTGCAGCTCAAACTGCTGCAACCACTACATGGGGTTCTGGTGTAACAGCAGTAGCTTTTGATCCAACTTGTGGTGGTTCCGTGATAGCTGTTGTGATAGCTGAGGGTATGGACCTGATTCTTGATCTTTTTTCACACAATTTATATTTGTTCTGTGCTATGGCATACCTTCATTAATAATGATTTTGTTCCTTCAGTTTCAATATATTCTTCTGACATATAATTTACATCATGGTTATTGAACTCGAGTTAACTCATTAACTCTTACGAGTTTACGAGTCCACTTACCCTCTGAGTTAACTCGTGTGTAAACTTTTTTTTTAGTAGACTCTGAGTCTTCTATAAACTCTAAGTAAACTTGGTAGACTTTCGAGTTTACCATTGAGTCAACAAGTTAAAAAAATTAGATCAAAATATAAGTCATTTTGGATTGTTTTCTGTTTGTCTAATGTTCAACATACCTTCATTTAGTGTGTTGTTCCCGAATAATAAAATTCTCACCTTTAATAACACCAAACTTTTGTTCTCTAATAACATCAAATCCTCGATGGGAGTGATCTACCACTACTATAACATCTGCAAGTTATTATCTAGTAGTGATGCATTACTAGACTTGATTATTTAAATATTGTGAAATTTATGATTTACTATTTTGCTTTATTATATTGTTAATTGGTATGTTATTTATAGATATTTTGTTATTATTTTTATATGAAGTGGACTCTTACGAGTCTACGAGTTAAATCCACGAGTCGAGTCTAGGGAACTCTCACTAGTCTACGTAAACTCTTGAGTTTGATAACCTTGATTTACATTATCATTATTTTACCCATTTTTATGATTGAGTCCTAGTGAATATCACTTAGATCTGAAAACTTTATTGTGTTTAATTGTTTATCTGTTCAAGTTATTAATCCATCGTATTTGTGAACATTTCACTTAGATATGAATAACAAGTTAGGGAGTAATTATACAATGTTGAAGACACACTAGGGATCAGATTTCACTTGGTTTTCTAACCATTAATTAGAATTCTGACCTAAATCTAAACAATCAATTGATGGGAAACCATTAAAGTCATGGAGAAGAGTTAAAATACTAAAGCTCCAAATAAAGTCATTCTGAATCAAATTAAATCACGTATAAATCTTAATAATTAAAGCCCAAGACCTGTTTAATTTTGGAATACATGGGTTTGTAATTGAAACCAATAAATTGATTAAATAACCTTTCATTGTGGTCAAACTACTTGTCTTTTTAATAACAGCTATGCGTTATGAAACAAAAGGAATTTAAATATCCACAATTGAGAAGAAAAAATTGAATGTCAAATGATAGTATGATACAAATGGAGCATATGAAGGTTATGCATGTTCCCTGCAAATAATCGTGTTTCCAGTCTTGGACTACTCATCCTTGAACAAGTTGGACATTTTAGCCATGACCAACTAAAGTAACATACTGGAAGGACCCATGTGTTTTATCTAGCCATCTCTACATCTTTAGATTTCGTGACAATGCTATCTGTGATTTATGTTTGGCAGAATTTATGCCATAGTTTCTTGTGCATTGATCATTGAGCTAGGAAATTGATATTATATTTATAAGTATATAATTTATCTTACCTTTGTAGGACAGTACATGTCCCCATATGATCCAGATGAGGGCCCATCAATCACAGGGTGGAGAGTGCAACTCTGGGAATCATCTTTACAGCATGTTGTCCTTCATCCAATATTTGGGAATCCCACTTCCAGTATGGGTGGACAGCCTCCTATGCAAACTGTTTGGCAGACCAAAGTGGACCTGAGCATTCCACCAACAAATGATTTCAAGAATCATCAAGCTCCAGCAGTTGGAATGAACACTGACATTCAAAAGGTATCCGAGTTTGGTTTTGATAAGTCAAAAAGGGTCAATTTTGATCCTTTTGATCTTCCAAGTGATGTTAGGGCACTTGCACGAATTGTATACTCTCCTCATGGTGGTGAAATTGCCATTGCTTTTCTTCGGGGTGGAGTGCACATCTTTTCTGGTCCAAATTTTGCACCTGTAGACAACTATCAGATTAGTGTTGGATCTGCAATTGCTGCTCCTGCTTTTTCTTCAACAAGCTGCTGTTCAGCTTCTGTTTGGCATGACCCAAGCAAAGATCAGACAATTTTGAAAATAATCCGGGTTCTTCCTCCTGCTATTCCAACTAGTCAAGTAAAGACCAACTCATCAAACTGGGAGCGTGCAATTGCTGAAAGGTGATTTCTTAAAACCCTTCTCAGTATATGACATGTTAATGCTTTCTTATTTCTTTTACATAACTCAGTGGAGGTTGTGATTTATTTCCCTTTTCAATTGTTTATTTTGTTGCGAACTGACATCTGTTAGGAAAAGCAGGTTTTGGTGGAGCCTTTTGGTTGGAGTTAATTGGTGGGATGCTGTGGGCTGTACACAGAGTGCTGCTGAGGATGGTATTGGTAAGGAGGTGCAAATTATTTAGTCATGTCGAAGTTAGTGCCAGGGGAATTAGAGAAAAGGGATAAAGCAATTTGCTTTGTTTCATATATGCATTCTACTATGAAGCAACTGCACAATGTGCCATTGATGCTCTGTATTGATGAGTTTCTTTGTTTTTTCCTTTTTCTGTTTCAGTTTCACTCAACAGTGTTATCGCAGTCTTGGATGCAGACTTCCATTCTCTTCCTTCTGCTCAGCACAGACAACAGTATTGTCCTGTATACTTCTCTGAACTTTCACCATTAGCTTTCGTAGTTATCTTCTTCATTGTTAGGTCCTGGGGTGTCTTATTATTTTTCTGTTGTTTCATAGTGTTTCTGGGATATAATATTACCTGGATAATGGACCTTGCAGTAGATACTTGTTATTAGCAATTTTATCTCTATCTCATAGTTTTAACTATTGGCTTCCTTATAGGACTTTATGCTGACACAACTTGATGTTCATTTCCACCTGATGATCCTGTCATCCTGCTTTGTTTCCCTTCTTGTTTTACTATGTTTCCTGTTGGCACAGATCTCCCCCCTTCCTCAAAGTTCTAATATTGGCTTTGACATTCTTAATTATTTTTTTAGCCATTTCATTGGATTGTTTCAATTTATGTTAGTCTAGTATGAATCTCATTTTAAATCTTTTCAGTACCTTCTGAGATCCACTTGAACTAAACTTTCAGTATCCCTGTCCTGTGTCTTTCAATTATTGTTATAAAGTTAAACAATATTTTACTTTCAGTTTCTCCTGTTTATTGGTGTTTTTATGAAGACGTGTGGAAATGGGCGTGTTGATGCTTTTTGGGCTAAAAATAGCTTGGTTTTGTATGATTGCAGAGTCTAGACAGGATAAAGTGTAGGCTGCTTGAAGGGGCAAATGCTCAAGAGGTCAGGGCAATGGTTCTGGATATGCAAGCTAGGTTGTTGTTGGATATGCTTGGGAAGGGAATTGAGTCTGCTTTGATAAATCCTTCAGCTTTAGTGCCCGATCCATGGCAAGTATCTAGTGAGACATTAACCAGCATTGACCCTGAAGCAGTGGCTGTTGAACCTGCACTAGTTCCATGTGTTCAGGTTTGCTTTGGTCTCTGTTGTAATCATGTGCTCCTTATTTTGTTTAATGAGAAGATCTACACTCAGCTTTCTGGTGTAGCAATGGTTAACTGCATGGTTCAGTCCGCATACTTTGTTTTGTATGCACTTGGCATACATTTTATTTATATTCATTTGTTAAGAAAACTGATTTTTTTTTTACATATAGTAAACAATACCCGCCAACCTATTGAACATGTAATATTATTAAAACCTTTGATTCTCTCTCTTTGTTCAATAAGCTGACATTTTTTTTTAATTTGTTTAACATTTGACTGTATATACATCTCAGAATTGTTTCAATATTACTCAGGCTTATGTTGATTCAGTTCTTGATCTAGCTTCACACTTTATCACCCGGTTGCGGCGTTATGCAAGTTTCTGCCGTACATTGGCAAGTCATGCTGTAACTGCAGGGACGGGAAATAACCGAAATGTGGTAGCTAGTCCTGCCCAATGTTCTGCAACTCCTGCAACAAGTCAGGGTAAATGTGCAATGATATGATATTGTATTGTTTTTTTTAATAGTTTGTTTTATTCTATTAAAATTTGTTTCATTTTATTTATAACAAATCAGATAGATCCTTACTTCTAAAGTCTTGTCATTATGGATCTACTATAGAAGCAAGGATTTGGTGCTCTGTTTTTTATTCTCACAAAAAACTCAGTTGTCCAACCTTGAATACCTTTATTTGTCTTTCTATCATCATTACCTTTATTCTTTATATTACATTTTGATATGGTTATAATTTTTCCCTTCATGTTTGAATAGTTTTCTAGAAGGTTGGATAGGATAAAATCAGATCTCATTTGATCTTTGATTTTCTTCTATAATCAAATTTGATCTTGTTTTTATTTTGATTTATCCTTGATTTTCTCCTATCTTTAATTTAATTTACTTTGCAATCAGATTGGCTGGAGAATCTCTCTAACCAATTTTACCTATTCTTATCCTCATTGTAACTCTCTCTTAATCAATGAATAAGAAAATCATTCTCTTCTATACTACTTTACATACCAAATTAAAGCAAAAACAACGTTGTCCAGGAGGTCAAAATGGGACAACCAGTTCTAGTGGAAGCACACAGATGCAAGCTTGGGTACAAGGGGCCATTGCCAAGATTAGTAGCACTACTGACGGAGTGTCCAACCCGGTTCCTAACCCCCCCATCAGTGGTCCTTCGTCCTTTATGCCCATAAGCATTAATACAGGAACTTTCCCTGGAACACCTGCAGTTCGACTTATTGGGGACTGTCATTTCCTCCATAGATTATGCCAACTGTTGCTCTTCTGTTTTTTCTTTCGACGAGCACAACAACCCCGCTATGCGGGAGGTGTACAGAGAACTGCTGACACAAATCTACAAAAACCTCAACCTAATGCTTCTGTCCCTGGCAAGGTGGAGGAAATTGCAAAACCAGTGTCAACTGTGGTTAGGCCAGATGATGGTCAGGCTGGTCGAGTTAGTCAACTTGTGCCTGCATCAAAAGGAGGTGAAGAACCATCTCCAGGGCGTTCAAGAATTGGTACTGGAAATGCTGGCCTAGGATATACATATGAAGAGGTATAACTATATTATGACTTGTCTGAAAGCGCTCCTATTTCAGTGATTTATGACTGACATAAATGTGTTTCAAAAGTCAACATAATCCTCTCTTGTACTTACGTAGGTTAACTGTTAACTTATGGTCTTACTACACTGGTAATGCAGGTCAAGGTTCTTTTTCTAGTACTTATGGATCTATGTCGCCGAACTGCTGGCTTGCAACACCCGTTGCCAGTTTCTCAGGTGGGGAGTAATAACATACAGGTTCGGCTGCATTATATTGATGGGAACTACACTGTACTGCCTGAGGTTGTGGAAGCAGCCCTTGGCCCCCATATGCAGGTTACTTTCTGTTTTCCCATTTCAAAGTCAGTATTTTTGGCAGACAAACAAATTCATGATTTTGAAAACAAGATGTAATAGCGATACCTGCTAGTCAAGTAGGACTAGAAAGGGCTCTATTGTCTTTCTTCAGTAACGATGCTTTTTTGACGAAGTTTCTGATTTAGTGATGGAGCACACTGTTAGATTATGTAGGAGCATTTGATGATTGAATGTTGTGCTTTTTAAAAAATATATGTTTTTGTTCTTTTTGGGTGCATTAAAATGTGTTTGTTTCCTTGGAATAAGTGACTATCTTTCCTGTGAATTAATCCTGCCCTCAAAACTAAATAAAAACTGGCTATGCAGAACATGCCCCGTCCTAGAGGTGCTGATGCTGCTGGTCTTCTACTACGTGAGCTAGAACTCCATCCTCCAGCTGAAGAGTGGCATAGACGGAATATGTTTGGTGGACCTTGGTCTGATCCAGATGATTTGGATTCTGCAAATGATGCACCAAAACTTATTAGTTTAAATCCACTTGATTCCAGCTCATTGGAAAATTGTGATGTCTACTATGGAGCCAATGGCTTATGGCCAAGGAAGCGCAGGATGTCTGAACGAGATGCAGCTTTTGGGTTGAACACTTCTGTGGGCCTGGGAGCATACCTTGGTATAATGGGATCTCGAAGAGATGTTGTTACTGCATTGTGGAAAACTGGCCTTGAAGGGATCTGGTACAAGGTATGGAAAGAAACTTTGTTTGGTTTTTTGTGTTGTTTTATATCCTCCATATGTGCATGTAAGGTTTTATAACCTATTAAATGTTTGGTATTTAGTACGGTCCAAGAGTTATTCTTTTTAATCTTTGATAAAGTCATGGCGGTTTTCTCTTGATAGATGCAAGACAAATTGCTAATTGACAACCGATGTAAATGAAATGTTGAATGATTGAAAATTTTAGCAGTGTCAGACTTGTTAATCTGGCTAACATGGACCAAATTTGTAAACAAAGCCATTCCTTCAATGATGCCTAGAAATTAGTAATAGACACTTCTCTTTCTTCTTCCTGACCTTTTAATTTCATTATCTGCTTGCATGTAAATATTATTTTACTTTCTCATTCTCCATTTTACTTCTGTTTGATACAGTGTATAAGATGTCTGCGGCAGACTTGTGCTTTTGCCTCACCAGCTTCCACCAATCTTCCCAGTCAAAATGACCGGGAGATTTGGTGGATCAGCCGCTGGGCTTATGGATGTCCAATGTGTGGTGGAACATGGGTTCGAGTTGTATAGCTGGCTTCAATACAAAATTGTCATTTGAGTTTCTCTTTTGGTGAGGGAAGAATTGCTTGTATTTTACCTGCAGAAACTGAAGGTGGATGGAATTGTACAGGAGAGGAGACATGTAGATTACCTGACAGGTTCTTTATCAGTATGTTGTCATTAAAAGTGACCAAACGAACTTGGATTTGAGTATTATTCTACTGATCACTATGTAAAGTTCCAAGAGTTATCAACTAATTCACCTAGATCTCAAATTTCTTTGGAATTATGATCATGTGATTCGTGTTACACCGCCAAAATTAGGTATTATCCTTGTGATGTTTGTAGTTGCTCCTTCAACATCTATGGTTTAGGATTTTTAACCCTTAAAATTGACTTGAGTGCACCATATCAAGCACAACATATGGTGATATCATGCAAGGCATATTAATTTTCCTTTTACTCATCACAAGTTAGTTGGATGATCATTTGGTCATAATTTTTTTCTTTCACCGTGCGTCTTTTCTGTCAAATGCTTTATGTTTATTTTTGGAATTAGTAATCTAGAAGATTGAATAATTAGGAATTTGGATCAGATTA

>GmMED16-3

ATGAAATCAAAATCAACTATTGTAGTCTGTAGTACCATGAGAGAGACACATGTCTCTGCCTATGACTCTTGTACTGTTGTGAGTGTGTCCTATTTTCCTTCCTTGCAATGAAATAAATTATTATTGCCAGACAAGCAAAAAGCAATGCACAGCTTCCACACTCCATGTCCATGCAATTCCATTGCACACCCCTCTTTCTTTCTTTCTTCATCTCAATCTCATTCTCAAGCAACAAAGTTTGTAAAATACACTGAACTGAATCCAACCACCAACTTCACCTTAGATTTTGTAATTTCCTTTGCTTTGTCTTATCAGCTAGTGGTTCTATAACACTAAATATGTAGTATGATTTTCATCAATTTTTATTATTATTATGTAATACTCCATGTATTTAAATTCTTTTAATTTATACTAGTAATGTTTAACTATGAAGATTAAATAAAACTATAATACTAACAATACCAAACCACTTAATAATTTCTCAAACATCACAATCGTCACCCTTTTAATAAGCAAACAAAAATGTTATCAATATGTCTTCTAATATACTCGATTAAATATATTCTTTATTATTAGTCAAATTTAAAAAAAAATCAACTTTTTTGTTGTAAAATTGCTTCTTATTGAATTTCTATCATAATTATGTAATTTTTAACAAAATTAAATCAGTCATAAATAAATATATTTTAAGAAAACTTATGTTAGAAAATGTGTTTATATAAAAAAAATATAAAATAGTCTTACACTATCATATAATAAATGTTATTTTCATAATAACTATGTTAAAAATCTTACAAACTGGATTTTCTAATTGATTTAGAGGATAAACAAATTTATTTGTTCGTTACTATTAAAATTGATAGATAAAAATATATAAATTAATAATTAACAATTAATATTTGTCAACTAAACTCATTTTAGTCTATTATATTTTAGTGTTGTTTTACTTTAGTACATCAAATTTTAAAATTTTCATTTTAATTCTTTATATTTTCATGAGTTTTATTTAATTCTTTATACTTTTAAAAGTTTTATTTTAATTCTTTTTATTTTCAAAAGTTTCATTTTAATTATTTATATTTTGAAAGTTTTATTTTAATCATTTTTTTTATTTTCATTAATTAGCAAACATTAATGTTATGTTAATTTTTAAATTTTAAAATAATTAAATTAAAATAGTGACAGCTAAAAACTAAAACTAAGACCCAAAATTATAAGAAACTTCTCCTAATTGGCAGCAATAGATTCCATAGTCAAGAAATAGGTACCTATTGTTAAACCAACCTCAGACCCAACTCATTAGCATTTGGTGGTATAGTCGTCCAGATGCATTGATTTGTGGTCTGCAAATCTGTAGTGTCTTATTAGTATTAATTATATTGAGTATTCCAGTCCAAATAGAGAATTTGACCTACAAGAAACCTTTGACTACCAGATAGGTTTATCATTAAGGAGGTGCTCCTGATTAGAACAAGTAAAACTGTCAATATGATTTACAACAATGCATACTAGAAATGTTAGTGGTCCAAAAAATTGTATCTTGATAGGATGACAGGCTAACAGAATCAAGTGCAGAAGGGGTTCTAAATAATGCTTCATGTCCGAAGGATTCCCATTTCATTTGACCACTTTGGTGAAAATATTTTTTTATGCCTTCTTATTTTGTTCACCTCATGGATAAGCTTTTAGTTATTGAGGAAAAAATTTCTCAGCAAGTGATGCTCAAAAGTCAAAGAATCTTTATTTGGCTGCAGGCACCTATCAGTATTTTTCAAACGTTGATTATGCTACTTTTTTTAAGGAAGATTATGTTACTGGTTGATTTAAATATATATATATACACCCATAAAAAAAAACTATATATCAACCTAATTGATTAGAGAAAAACCTTAAAATGAAATAATTATTTTATTCAGGAATTCAAATCCCACACAATATTCCCTGTAAACAAAACAAATGCTGCGTCTGTATGGAAACTAAACATGCACATATAAAATATGAAAATATTTCGACTTTCTAGAACTAAAAAAAAGTTATGATTAAAACCGTATTATTTTCATGGTTGATAGTTTACGAATCTGTTGGTGTAAAATTGAGATTATGTATTTGATCTTCTGAGCATTTTTTGTAAAATTAATTTGTTTCGTATTATTTTATGCTCTTTATGCTATTCTAATTCTGGTGCTGCCCCTTTTTAAAAGAACTTATTGATTCTTTTTCCTGGTGCTGTTTCCTGGTGTGGAAAACTGAATGCTATAGCTTGTCCTTCTGAAACGTGTGCAAGAATTCCAAGGTTAGTTTTATCCATTGATCATGTTTCCTGGCTGAAAATTTTTTATTATGTGCTAAATGTTTTGTTTAAAATATGACTATTGGTTTGGGTAACTGATAGTTGATGGAAACATTTAAGAGCCAAAAAACAGATTAGGTTATTTTGAGGGTCCATTGCATTTTAGAATTTGTGGATAAATTCAAGAACATTTGATTAATGTTTCAAAGTTCAAACTTTTCCAATTGCGTTTATTCTATTGTGATACCCAGACCAGAAGAAGCACGAGGAAAAGATATTATGGGATATATCTTTTTGTATATTCATCTAGGTGACTGTCTCTTGGACCTTTGACTGGTTTACATAATATTTTTCTTGCTCTTAATCAGTTTCCTCATTTGTTGGCAAATGAATTTGTCAATCAACTTTAAGTTTTGTCCTGCTTGGTTTCTACTTTCTAGTAATATTGTAAAAGGTTTAAAAAAAAAGTGTTAAAAGAAGGGCTGCAACCAAACCTTCCAATAAATATCAGCATATATATCATATTCATTTTATAGGCATATTCATTTGTTAGACAGGCCCACAAACAAATTTGAATGAATAGAGCTTATGGCAAAAAGACTTTGTTCTCATGCTAAATAGGCTTTGCATAATGATGTCCAATGCTTATATCATTTTTGTAATATGCTTCTAATATTTGTTCAAGTATTAAGTTTATATAGACAAACATTCACATATGATGTCTAACCCTGCTCCATTGTGAGTACCCAGTAGTTACTTTGGCTTGCAACACCCTTGCATGGCTTCTTTTACTGGCAATTTTATAATGGTTATTAGTGTTTCTTTATTCTAAAGCTCAACCGGAAATCCACCATTTTGGATCCCTATACATATTGTGATCCCAGAGAGGCCGACTGAATGTGCAGTATTCAATGTCATTGCAGGTATTTTGATGTCACTGTTTCCTTCTATAATCATGGATGTTACAGTTTTACTGTTCTATTGGTGTTGTTTGATCTTATTTTTCACCCTGGCAGAAATGCCAAAAGGTGTTTGTCTTTTTCAATTTCATTATGATAGTAATGAAGTCAAACACAGATTTTAATAAACCCTCGGTATTATTCTTATGCAGATACTGATATCAGTGTACTATCAACACCTGTGGGTGGATTTACAATGAAAGGTAAATACTGAAACAAAAACAGAAACAGCTGAAGTGGGGTATACGAGAAAACCTGCCCTCTCCTTTGCTTGCCTCCTAGCAGTCTGTTTACTGATATCCCAATCCCTTTGATTGCCTTATTGGCAATCTGTTACATCAATTTTTCCTACCTGCCCCTTCCTAATATAACCCTTAGTAATTGGTGATTTCTAGCACATTACCAGCATAAAGGTTCTGCCAAATCCACGAGGCTGTTCGACATTATAATTTTGCATAAACTAATTATGGATTTGGGAATATAAAATTCAGGTATAAGGCCGCAAGTTGAAAGCACAGAAGAAATCTTTTAATTTTCAGAACTTTGGTTTTGGAACTTGTGCATGTTGAATTAAGCCAACCCTTGGAGGATCTTGATCTGGATAGCCTTTAAGCATCATTTCAGATGTTATTGACACATCTGTTCACTCTTTTTAAAGTTAAATATTTGGGGTATCAACTATCAACAGAAAAGTCTGAAGAATTTCCATGATGACAACATTCGAAAAATTAGGAGATATAAGGTGTGTTGGATGGTTAGAGGAGAAGAAAAGAGGGAAAAGGTTGTGGGTTCCATTTCTTCTATAAACAAAACTAATATTCTAACAAACTAATATTTGTCGATAAAAAAATATTCGAAAAATTATTATCATTATCATTATTATCATTGTAACTAATTTACATGGAATATTTTTGATAAATTATATCTCATTTTTAATATTGGTAAAACAAAAAAGTAGTCAAATTGAATCTGAACAATTAACAATTAGCTCCTCTGTAATTTTCTTGTTGCCATCAAATTGGTACTATTTCTTGTCTTTTTTTTCCTGGGCTTTTAGTTTCTGAACTGAACTGTTTCACTATATATGTATGAGTGCATATGTAAATTTTGAAATTTGATCAGTTGCACTGTATGTCTTCACAGATTCTCCTCGTGATTCTGTCCAGTTCATTGAATGGTCCCCCACCTGCTGTCCCCATGCATTATTGATAGCAAATTTCCATGGGAGAGTAACTATTTGGGCTCAACCTTCTCAAGTAAGTGAGACTTACTTAAGTGGATACTGCTACCATCACAATTTTTTGCATAACATGGATGTGTAGTATAATTGCAAGTGCTCTGTCATTGACTTTTACGTGGCCATATTTTTTGGTGAATCGTGGTCATAGATTTAAGTAGTAAGTTTGTTGTTCGTCTGTAAAAGAAAACATGTTACTATTACTGAAACTTACCCCAAACTCTATAACTGTCGATTATTGAGATGGTTTTTCTAAATCACACAATGGGAGCTTTAGCTATGTATTATACCCTAGCTCTGCAAGGAGGTTAATGTAATTGACTTAGTGCTTGTGAATGCCTATGGATTGATCTTTTGTCCAACTTTAACCTTCTCTGCCTTCTCCATTATTCAGATATGTTTTTCTTGTTTATTTGTTTCTGATTTCTTGTCCATTCCGGGGCAAGCTAATCTTGCACTTGATGCTAGCTGCTGGCTGCGAGAGCATGAGTGGCAGCAAGATATTGCAGTTGTTACAAAGTGGCTATCAGGGGTATCCCTGATATGCAGATAATGTCTCTTCTATGTGATGTTTAAATTTTCTGCATATCATTTTTGAGGAGTTGTTATAATGATTTTTATTTATCCAATTTCGCCCTTAAACAATTGTCTCTAAATCGATTCTATCTCTAAACATTATTCTCTAAAAATTATATGAGAGAGCTTTTTTTATAGAGAGAGAAATTCTTAGAGGGGGTGAGTTTTTTTTTTTTTTAGAGAAGGAAAACTTTGTTTAGATAGACAATTTTTTTAAGTTAAATGGGTAATTCATTTTTTCCCTCGAAAATCCCACAATTTGGAGGAACTTTTAATTGTTAACAAAATATGAAGAGAAAGGTTTTGTAGGATTAGTGAAAAATCCCTAATTCTCCTTTAGTCACAATGTCATAGCATTGTGCTGACTTACAACTTATACTGTAGCTAGCTTGTGCACACACACATGAATCTTGTGTACTTCAATCTGCTGATTGTGATATTTTTTAATCCAAATTAATTATGTATTTGAAAAAAACAGTATAGGTGGCTTTCATCTAAACCAAGTGCTCCTGCCAATTCAAGGTCAACATTTGAAGAAAAGTTTCTTTCACGACAATGTCAAACTTCAGGTATTTTTTTTCCATTTGCTATTATGATTGCCTTTTTTCTTCCAGTAAATTTTTTCACTGTAGCAGTAATGAGATATCAAAAGAAATACGACTTTCGACATTGTATTTTGTCATTTTGTGGTAATCTATTTGTTTATTCTTTTTGTTCTTTTCCAATGAACCATCTGAAAGGCATAAGCTAAAATTGTATCTTCCATGGTGCAATCCAACAAGGATTTTTCATGCAGATTAGATGTTGGAAGAAGAGGGACTTGTGTGTGTGTTGGGATTAGGGTTTGAGGGTCATATGGTCCATATGTATACTCTTTGTAACTTAGTAGATCATAGCGAATTATTCTGTTTCACCAGAGGACATAAGCCTATCGATTTCTGTTCTTATTTTTCCACTTTATGTTGGGTTATAGCAATTTTCTTTTTTGCTTTTCTGATTTTGACAATAAAAGTATCTTCATGAAGACAAGTAATTTGAAGTGAAGTACCATAATCTTCCTATGATACTCAACCATCTTAGTGTCTGGCTGATGCTTATCAAGCCCAGATTCTTTAAACGAGATGTGCTATATCAGACACTCATGTCATGATGACACTTGTAATAGTAGGGAGACAATTGCCAAATTTGTGTCTATGTCTGTGTCATGTCCTGTGTCTGTGCTTGTTAGTCTTAATATTAACACAATTTGTATGTTGATGGTGGTAATGGTGACAACCATGGCAATGCCTGAAATTAAAACTGTTTTCCCTCTATTTAATTGCTTACTTCCGTTATTTTTTTAATAATTCATGTTACATGTTCATTTTTTTCTAATTATAGTGCAATTACTGCAATATGTATATTAAAATTGCTGTATGCTAGTGTGTCTTACACATGGAGTTGTTTTGGTTTCAGCTAGATAGCCCAATTTTCTTTGTGTCTGTTCTGTGTTCTCATCAGGATCGGTTCAACTTCATTGGTCCCAGTGGCCTCCTCAGAATGCAACAACACCCAAATGGTTTTGCACTAGTAAAGGACCATTGGGTTGTGGCCCCTGTGGCATTATGGCCGGTGATGCTATCATTACAGACAGTGGTACCATGCTTGTGACAGGTGTGCCAATTGTTAATCCATCCACCATTGTTGTTTGGGAGGTTATGCCTGGGCCTGGTAATGGTTTCCAAGTAATTCCAGACAAACCAAAACCTCGGTGATTCTATACCACTTAACTGTTCACCGGTTTCAAATTTTTCGGCATATGTGAGTCCTGAAACTGCAGCTCAATCTGCTGCAACCACTACATGGGGTTCTGGTGTAACAGCAGTAGCCTTTGATCCAACTTGTGGTGGTTCCGTGATAGCTGTTGTGATAGTTGAGGGTATGAACCTGTTTATGACTTCTTTTTTTCCACACAATTTATATTTGTTCTGTGCTATGGCATACCTTCATTAATAATGATTTTGTTCCTTAAGTTCCAATATATTCTTCTGACATATTATAATTTACATCATCATTATTTTACCCATTTTTATGGCCGAGTCCCATTGAATGGCATTTAGATATGAAAACTTTATTGTATTTATCTGTTCAAGTTATTAAAACCACGTATTTTTCAACATATTTTTTTCCTCCATTGACAACATCATATATTCCTTTGTTACTATGACCATATATAGTTCCACTCCATTACCTTCATCATTATTGCCATCTTTCTTGGTATAAATTTCTGCAGTCTATCTAGCTCTCTCTCTCTCTGTTTTCCCCCTCCCCTTCTTTATAGCCACAACAATTGCAAGCAAGTCTTCATTCTTTATCTACTCAAAACAAAACGATTTTTTTGGCCTACAGCTCTTAGACTGGACAGTCCAGAATTTCTCCATTTGACAGCTGAAAAAGGGGGGGTAAAAGCATATGAAATCAATCAATCAAGTGGACGAGTCCAAAATTGTTCCCATGAGGATTATAATTAAGAACCAATTACCAACTACTCTTAATTGGATGAAACTGGAATACAAGCAAAAATTAAATACTGAAAATCTGAAAAGCAAATGCAAAAAACATCATGAATAACAAGTTAGAGAATAATTATACAATGCTGAAGACACTAAGGATCAGATTTCACTAGGTTTTTCTAACCATTAATTAGAATTCTGACCTAAATTTAAACAATCAATTGATGGGATTATGCATGTTCCCTCCAAATAGTGGTGTTTCCAGTCTTTGACTACCCATCCTTGAACAAGTTGGACATTTTAGCCCTGACCAACTAAAGTAACATACTTGAAGGACCCATATATTTATCCAGCCATCTCCACATCTCTAGATTTCGTGACAATGCTATCTGTGATTTATGTTTGGCAGAATTTATGCCATAGTTTCTTGTGCATTGATCATTGAGCTAGGAAATTGATCATATGTTTATAAGTATATAATTTATCTTACCTTTGTAGGACAGTACATGTCCCCTTATGATCCAGATGAGGGCCCATCAATCACGGGGTGGAGAGTGCAACGCTGGGAATCATCTTTACAGCATGATGTCCTCCATCCAATATTTGGGAATCCTACTTCCAGTATGGGTGGACAGCCTCCTATGCAAACTGTTTGGCAGACCAAAGTGGAGCTGAGCATTCCACCAACAAATGATTTCAAAAATCATCAAGCTCCTGCAGTTGGAATGAACACAGATATGCAAAAGGTATCCGATTTTGGTTTTGATAAGTCAAAAAGGTTTGATTTTTATCCTTTTGATCTTCCAAGTGATGTTAGGACACTTGCAAGAATTTTATAATCTGCTCATGGTGGGGAAATTGCCATTGCTTTTCTTCGGGGAGAAGTCCACATCTTTTCTGGTCCAAATTTTGCACCTGTAGACAACTATCAGATTAGTGTTGGATCTGCAATTGCTGCTCCTGCTTTTTCTTCAACAAGCTGCTGTTCAGCATCTGTTTGGCATGACACAAGCAAGGATCAGACAATTTTGAAAATTATCCGGGTTCTTCCTCATGCTATTCCGACTAGTCAAGTAAAGGCCAACTCATCAAACTGGGAACGTGCAATTGCTGAAAGGTGATTTCTTAAAACCCTTCTCAGTATATGACATGTTAATGCTTTCTCAGTTTTTACACAGTGGAGGTTGTGATTTGTGTCCCTTTTAAATGGTTTATTTTGTTGCGAACTGACATCTATTTAGGAAAAGTAGGTTTTGGTGGAGCCTTTTGGTTGGAGTTAATTGGTGGGATGCTGTGGGCTGTACACAGAGTGCTGCTGAGGATGGTATTGGTAAGGAGGTGCAAATTATTTTGTCATGTTGAAGTTAGTGCCAGGGGAATTAGAGAAAAGGGATAATTCAATTTGCTTTGTTTCATATATGCATTCTACTATGAAGCATCTGCACAATGTGCCATTGATGCTCTATATTGATGAGTTTCTTTGTTTTTCCTTTTTCTGTTTCAGTTTCACTCAACAGTGTTATTGCAGTCTTGGATGCAGATTTCCATTCTCTTCCTTCTGCTCAGCACAGACAACATTATTGTCCTGTATATTTCTGTGAATTATCACCAGTAGCTGTCATAGTTATCTTCTTCATTGTTAGGTCCTGGGGTGTCTTATTATTTTTCTGTTGTTTCATAGTGTTTCAGGGATATAATATTACCTGTATAATGGACCTTGCAGTAGATCCTTAGTCAACTAGTGCCTGCGTCAAAAGGAGGTGAAGAACCATCCCTAGGGCGTTCGAGAATTGGTACTGGAAATGCTGGCCAAGGATATACATATGGAGAGGTATAACTATATTATGACTTGTCTGAAAGCTCCTATTTCAGTGATTTATGACTAACATAAATGTGTTTCAGAAGTCAACATAATTCTCTCTTGTACTTTCAGAGATTAACTGTTAACTTATGGTCTTACTACACTGGTAATGCTGGCTTGCAACACCCATTGCCAGTTTCTCAGGTGGGGAGTAATAACATTCAGGTTCGGCTGCATTATATTGATGGGAACTACACTGTACTGCCCGAGGTTGTGGAAGCAGCCCTTGGCCCCCATATGCAGGTAACTTTCTGTTTTCCCATTTCAAAAGTCAGTATTTTTGGCGGAGAAAAAAATTCATGATTTGAAAACGAGATGTAATAGTGATACATGTTAGTGAAGTAGGACGAGAAGGGGATCTATTGTCTTTCTTCAGTAACGATGCCTTTTTGATGACGTATTTAGTGATGGAGCACACTGTTAAATTATGTAGGAGCATTTAATGATTGAATGTTGTGCTTTTTTAAAAATACATGTTTTTGTTCTTTTTGGGTTCATTAATATGTGTTTGTTTCCTTGGATTAAGTGACTAGCTTTCCTGTGAATGAATCCTGCCCTCAAAAATAAATAAAAAGATGGCTATGCAGAACATGCCCCGTCCTAGAGGTGCTGATGCTGCTGGTCTTCTACTATGTGAGCTAGAACTCCATCCTCCAGCCGAAGAGTGGCATAGACGGAATATGTTTGGGGGACCTTGGTCTGATCCAGATGTTTTGGATTCTGCAAATGATGCACTAAAACTTGTTAGTTTAAATCCTCTTGATTCCAGCTCATTGGAAAATTGTGATGTCTACTATGGAGCCAATGGCTTATGGCCAAGGAAGCGCAGGATGTCTGAACGAGATGCAGCTTTTGGGTTGAACACTTATGTGGGCCTGGGAGCATACCTTGGTATAATGGGATCTCGAAGAGATGCTGTTACTGCATTGTGGAAAACTGGCATTGAAGGGATCTGGTACAAGGTATGAAAAGAAACTTTCTTTGTTCTTTTGTGTTGTTTTCTGACCATATGTGCACGTAAGGTTTTATAACTATTAAATGCTTGGTATTGAGTGCGGTCCATGAGTTTTTCTTTTTAATCTTTGATAAAGGCAATATGAGGTTTTCTCTTGATAGATGTAAGACAAATTGCTACTTGACAATCAATGTAAATGAAATGTTGAATGATTGAAAATTTTAGCAGTGTCAGACTTGTTAATCTGGCTAAACATGGACCAAATTTGTAAACAAAGCCATTCCTTCGATGATGCCTAGAAATTAGTAATAGACACTTCTCTTTCTTCTTCTTCCTGACCTTTAACTTCATTATCTACTTGCATGTAAATATTATTTTACTTTCTCATTCTCCATTTTACTTCTGTTTGGTACAGTGTATGAGATGTCTACGGCAGACTTGTGCTTTTGCCTCACCAGCTTCCACTAATCTTCCTAGTCAAAATGACCGGGAGATTTGGTGGATCAGCAGCTGGGCTTATGGCTGTCCAATGTGTGGTGGAACATGGGTTCGAGTTGTACAGCTGGCTTCAATACAAAATTGTCATTTTAGTTTCTCATTTGGTGAGGGAAGAATTGATTGTATTTTATCTACAGAAACTGAAGGTAGATAAAATACTGGATGGAATGGAATTGTACAGGAGAAGAGACATGTAGATTACCTGACAGGCTCTTTATCAGTATGTTGTCATTAACAGTGACTAAACAACCTTGGATTCGAGTATTATTCTACTGGTCACTTTGTAAAGTTCCAAGAATTATCAAGTAATTTACCTAGATCTCAATTTTTTTGGGAATTATGATCATGTGATTCGTGTCACACCGCCAAAATTAGGTATTATCCTTGTAATGTATGTAGTTGCTCCTTCCAAATATATGGTTCAGGATTTTTAACCCTTAAAATTGACTAGAGTGCACCATATCATGCACAGCATATGATGATATCATGCAAGGCATATTAATTTTCCTTTTACCCATCACAAGTCAGTTGGATGATCATTTGGTGATATCATGCAAAGCATATGGTGATATATGGTAGGTATCATTAAAAGCTTATGGGTTGATAGTTAGGCTTCATATATGCATAACTTATGCTAAAGATCAGTTTTATCACATAACGAAGTTATTTTGCTTATTAAAAAAAAAATTACATGGCTGGTGTAAAAAATACCTTGTGCATCATTGTTGAGCGTTTTGGAACCTAGTATATGGGTAAGACTAGATAATGTCTAGAAGCAGATCATTATTTCCTTCCACAATAGAGCGAAACTTACCAATGACATTTGAAATATATCATCATTATTTTTCTTCAGTGGCTGGTTGTTGAAAACAGATCAAAATCGTGTATGTATCAGAGAAGGCAGAGCTAGTTGAAGGCATGGATGAAGAATTGGTCACACTGAGAATGAGAAGAAATGATTGAAGCCTTCACACATAGGTGAGTGATGAAAATCTCAAAACTATCCCAAAAACAAAGATCAATGACAGGAGTAGGACCAAACAGTCAGAGGATACTCTCCAACAAATGGTATCAGCCATACCTGACAAGGTCCAAGTGAAAAAGGATGAAGGTCCAGATATACTACCAAAAATCTTGATTATTGCTAAAGGTCTATGTCAATTTTTTTTTCTATTTTTCGTTGTAATTTTAATTAAATAAAGTTTGTGCCTTTTCCACCTTTGGACTATATGTATTGAACTCGTTTTCAATGGAGGGACTTTTGACATTCTGAGAAAATAAGTGTGAAGGCAACCGTGACTTATCTTCCATTGCTGGAAGTGGCATCCTCCAAATTTTGTGACCTGTTCCGCTCCTAGTATGGATCACATCAGAAGACAAGGAGAGAATGCATGTGGTGGGGATTACTTTCTTGTGGGGCCCAAAAGGTTGAAGTTGACTGAGCATGACAAGCTGTTGAATAAGTTTTTAGGCACAGGGGAGCTCTGGTGTCTCTGTTGGGAAGGGATTATCCTGGGGAATGTGTTTGCTGTGATGGAGGAATTAGTGTCTAGGAGGAAATTGCTCAAGAAGTGTGTCTCCAACTTGGATTTGGAGAAGCTGGTACTTTTGTAA

>GmMED17-1
[truncated: 666,364 more chars]
