# Supplementary material for: Genome-Wide Analysis Reveals the Role of Mediator Complex in the Soybean—Phytophthora sojae Interaction
Source: Int J Mol Sci. 2019 Sep 15;20(18):4570. doi: 10.3390/ijms20184570 (PMC6770253; doi:10.3390/ijms20184570)
Supplement: Supplementary file 1 [file ijms-20-04570-s001.zip › Supplementary Materials/Data sheet S2.docx]

**Data sheet S2: The protein sequences of the mediator subunits in Arabidopsis thaliana, Phaseolus vulgaris and Medicago sativa used in this study.**

>AtMED2

MDNIVDSLNKAYEKFVLASAGVLESKESAGGQKALLTDTALENFKEKWELFRVACDQAEEFVESVKQRIGSECLVDEATGLTTTAAGGQAPAAVTGAATSLPPISAVRLEQMSRAVRWLVLELQRGSGVAPGSVHSSSTGFDSRFSEDSTQ

>AtMED5-1

MAPSEFQPSLWESVTSLIRSAQEKNVDPLHWALQLRLTLASAGISLPSPDLAQFLVTHIFWENHSPLSWKLLEKAISVNIVPPLLVLALLSPRVIPNRKLHPAAYRLYMELLKRHAFSFMPLIRAPGYHKTMNSIDDILHLSETFGVQDQEPGSILLAFVFSIVWELLDASLDEEGLLELTSNKRSKWPSSPHDMDLDGLENSVKRNENHDALEKANTEMAIELIQEFLQNKVTSRILHLASQNMESKTIPRGEFHAIVSSGSKLALTSDSALWLPIDLFFEDIMDGTQAAAASAVENLTGLVKALQAANSTSWHDAFLALWLAALRLVQRENLCLRYCFFMHMLEILSEERDPIEGPVPRTDTFLCVLLSVTPLAVANIIEEEESQWIDQTSSSPSNQWKEKKGKCRQGLINSLQQLGDYESLLTPPRSVQSVANQAAAKAIMFISGITNSNGSYENTSMSESASGCCKVRFSLFTLKMFVVMGVYLLCNISCWSLVMKGSPLTPSLTNSLITTPASSLAEIEKMYEVATTGSEDEKIAVASILCGASLFRGWSIQEHVIIFIVTLLSPPAPADLSGSYSHLINSAPFLNVLLVGISPIDCVHIFSLHGVVPLLAGALMPICEAFGSGVPNITWTLPTGELISSHAVFSTAFTLLLRLWRFDHPPLDYVLGDVPPVGPQPSPEYLLLVRNCRLECFGKSPKDRMARRRFSKVIDISVDPIFMDSFPRLKQWYRQHQECMASILSELKTGSPVHHIVDSLLSMMFKKANKGGSQSLTPSSGSSSLSTSGGDDSSDQLKLPAWDILEAAPFVLDAALTACAHGSLSPRELATGLKILADFLPATLGTMVSYFSSEVTRGLWKPVSMNGTDWPSPAANLASVEQQIEKILAATGVDVPRLPADGISAATLPLPLAALVSLTITYKLDKATERFLVLVGPALDSLAAACPWPCMPIVTSLWTQKVKRWSDFLIFSASRTVFHHNRDAVIQLLRSCFTCTLGLTPTSQLCSYGGVGALLGHGFGSRYSGGISTAAPGILYIKVHRSIRDVMFLTEEILSLLMFSVKSIATRELPAGQAEKLKKTKDGSRYGIGQVSLSLAMRRVKLAASLGASLVWISGGLNLVQALIKETLPSWFISVHGEEDELGGMVPMLRGYALAYFAILSSAFAWGVDSSYPASKRRPRVLWLHLEFMVSALEGKISLGCDWATWQAYVTGFVSLMVQCTPAWVLEVDVEVIKRLSKSLRQWNEQDLALALLCAGGLGTMGAATELIVETCHQH

>AtMED5-2

MVVPGRRTVWDCVIELTKMAQENCVDPRLWASQLSSNLKFFAVELPSTELAEVIVSYICWDNNVPIVWKFLERAMALKLVSPLVVLALLADRVVPTRSTQQAAYRIYLELLKRNMFTIKDHISGPHYQKVMISVSNILRLSELFDLDTSKPGVLLVEFVFKMVSQLLDAALSDEGLLELSQDSSSQWLVKSQDMEIDAPERYNEKTGSLEKLQSLNTIMAIELIAEFLRNTVIARLLYLVSSNRASKWHEFVQKVQLLGENSSALKHSKVLNSGDLLQLISNRRFGYSYDSKVTSARKSNAIVDFGSLSSYAGLCHGASLSSLWLPLDLVFEDAMDGYQVNPTSAIEIITGLAKTLKEINGSTWHDTFLGLWIAALRLVQRERDPIEGPIPRLDTRLCMSLCIVPLVVANLIEEGKYESVMEKLRDDLVTSLQVLGDFPGLLAPPKCVVSAANKAATKAILFLSGGNVGKSCFDVINMKDMPVNCSGNMRHLIVEACIARNILDMSAYSWPGYVNGRINQIPQSLPNEVPCWSSFVKGAPLNAAMVNTLVSVPASSLAELEKLFEVAVKGSDDEKISAATVLCGASLTRGWNIQEHTVEYLTRLLSPPVPADYSRAENHLIGYACMLNVVIVGIGSVDSIQIFSLHGMVPQLACSLMPICEEFGSYTPSVSWTLPSGEAISAYSVFSNAFTLLLKLWRFNHPPIEHGVGDVPTVGSQLTPEHLLSVRNSYLVSSEILDRDRNRKRLSEVARAASCQPVFVDSFPKLKVWYRQHQRCIAATLSGLTHGSPVHQTVEALLNMTFGKVRGSQTLNPVNSGTSSSSGAASEDSNIRPEFPAWDILKAVPYVVDAALTACTHGRLSPRQLATGLKDLADFLPASLATIVSYFSAEVSRGVWKPVFMNGVDWPSPATNLSTVEEYITKILATTGVDIPSLAPGGSSPATLPLPLAAFVSLTITYKIDKASERFLNLAGPALECLAAGCPWPCMPIVASLWTQKAKRWFDFLVFSASRTVFLHNQDAVIQLLRNCFSATLGLNAAPMSNDGGVGALLGHGFGSHFYGGISPVAPGILYLRMYRALRDTVSVSEEILSLLIHSVEDIAQNRLSKEKLEKLKTVKNGSRYGQSSLATAMTQVKLAASLSASLVWLTGGLGVVHVLIKETIPSWFLSTDKSDREQGPSDLVAELRGHALAYFVVLCGALTWGVDSRSSASKRRRQAILGSHLEFIASALDGKISVGCETATWRTYISGLVSLMVSCLPLWVTEIDTEVLKSLSNGLRKWGKDELAIVLLSLGGLKTMDYAADFIIHLRS

>AtMED8

METQPQQPPPPPVAEKLNPKLEKELNLESLKTRAVSLAKAIARILEDFDAYGRTNTTPKWQDILGQYSMVNLELFNIVEEVKRVSNAFVVLPKNVNAMNAAILPVMLSSKLLPEMETDDNAKREQLLQGVQSLPIPMQIERLKARMDMIAAACENAERVLADTRKAYGFGTRQGPSMLPTMDKGQAAKIQEQEKMLRDAVNDGKGTQLPPDQRQITTALPPHLADVLIINDAGKIALPGQSNNINNQGMMQVSGTQFVGRSAASPSGPNFDNTTSPLPYSNSPRATGMVNVPSPQHQIQQQQFQQQQQRSKLMQLPQHQQQQLLAQQQQQLRQSSMQGLGQSQIPALHDMHGQAQQKFQTSHGQHQMPYSQPMGAHQQFQARQLSGGHIQHSMSQGQLNPAMNRHLNQFSGGANSALFTSAQGSPSSQMIPNMSSMQSQTLVPRMQQFGVSGTNPQRSHSSQMLGDQMFNTSGMMQTQQTQIQQSQQQQQQQQQGGYGNMQTNQQSLQPNNMMQNAQQRHQNPQ

>AtMED12

MQRYHAANCTSAVNNSAIGGASARDSGRADSSSIGNYSLNSRRPPPLTPYKLKCEKDGLNSRLGPPDFHPPTSNSPEENLTKEYVQFGYKETVDGLKESEEIILSQVHTFSKPVVHKCKEAVRKCLRAINESRALKRKAGQVYGVPLSGSLLCKPGFPEQRSCGEETKKRWIESLSQQHKRLRSLADNIPGYRRKTLFEVLIRNNVPLLRATWFIKVTYLNQVHCWAINWCDLVLLLFLQEHLTRHKLLGVSNGQKMLLNICNTSWMNFCHGIAHFLLSKLEIGHHRCFIQDQCKRIVQHQQAFTARKHLYILNGGIWCVFYSGTMLKGFFFLISLLIGFSSSYSFVNFHFALLQEKEIFEILQLLLPIVYGVLESIVLSQTYVQSLVAIAVRFIQEPAPGGSDLVDNSRRAYTLSALIEMVRYLVLAAPDTFVASDFFPLPPSVAACGPNDVSYTSKAYENLEKLRSNSAEISAQFQGRGVLSRFEFLSFDYTISTIQRSADDLAKIASAGYPQHNVAKAVQALDKALSDGDIRAAYSYLFEDLCNGAVDEAWITDVSPCLRSSLRWIGAISTSFVCSVFFLIEWATCDFRDFRAGVPKDIKFSGRKDCSQVYLVIQLLKQKILGGEFTARKGKNCRNNFLGVSKPSGSMDAFESPGPLHDIIVCWIDQHEVHKGGAKRLQLLVFELIRSGIFNPIAYVRQLIVSGMIDVIQPAVDPERRMRHHRILKQLPGCFVHETLEEAQLFGGDKLSEAVRTYSNERRLLLRELLVEKGKYWNNLVLSDQKSKKISTSLSSVIFPRACNAKSNSKGPRKHTKSSVDIRELKERISALLQFPGMSCGVETPVRDEFQNSVKRSSGSVYSKMDQPEATPGCEDCRRAKRPKMNDEKSSCYQGNSPIASDEEDNWWIKKGSKTVESSLKVDPQIEITKQVPRGRQKMARKTQSLAQLQAARIEGSQGASTSHVCDNKVSCPHHGPGVEGENQKVVDVFRTSTPVDMVSVGNSLKQLQFVDKRSIAVWLTTAVRQLVEEPQKSSVRVGQFNRGAPVEEKNTIRWKLGADELYSILFLLDISLDLVSAVKFLLWLLPKANSTPSFAVQGGRNLVTVPRNVENNMCEIGEAILVSSLRRYENILLSADLVPEAMTALMNRAASLMSSNGKISGSAALVYTRYILKRYGSLPSVVEWHNNFKATSEKKLLSELDHTRSGNGEYGNPLGVPAGVDNPDDYLRKKISIGGARPSRVGLSMRDVLQRHVEEATHYLKKLIGTGTMKASLAEKNDDGYQVAQQIVVGLMDCIRQTGGAAQEGDPSLVSSAVSAIINSVGLSVARITDFSLGNIYQNHPSGVDSSNIARYILRIHITCLCLLKEALGERQSRVFEIALATESSTALTGVFAPVKGSRGQHQLSPESYDSNANNSTIDMSNGTGKMALSRATKITAAVSALVIGSITHGVITLERIVGLLRLKDYLDFVQFVRRTKSSSNGSARSMGASKVESPIEVYVHWFRLLVGNCKTVSEGLVLELVGESSVVAISRMQRMLPLKLVFPPAYSIIAFVLWRPFVSNSNSNSSVHEDTHRLYQSLTMAFHDVIKHLPFRDVCFRDTQGLYELIVADSTDAEFASVFESHGLDMHLKSVAFAPLRARLFLNSLIDCKVPSSGYSHEGVSEAKNRHQGNGTKLVDKLVSVLDCLQPAKFHWQWVELRLLLNEQALAEKLENHDMPLTDAIRSSCPTSEKPDASENEKNFIQILLTRLLVRPDAVPLFSEVVHLFGRSVEDSMLKQAEWFLAGQDVLFGRKTIRQKLIIVGESKGLPTKPQFWKPWGWCNSSSSDHITANKAGKKRKFEITSIEEGEVIEEGSGSRKVLLPRVLDENSPSVGYGITTERAFVQLVLPCIDQSSDESRSTFVNELVRQFSNIEQQLSSVTNRSTTSNKQMGTASSGSEISSNKGSTRKGLRGGSPSLARRSSANTTDTSPPPSPAALRASMSLRLQFLLRLLPVICGEPSFKNTRHALASTIVRLLGSRVVYEDYAVCSPRSELSKAETESTIDPSSMADLSSEVLFDRLLFVLHGLLSNHQPKWLKPRPSSNESSKDFTLFDRDAAESLQNELSRMQLPDTIRWRIQAAMPILLPSLRCSLSCQPHSVPPTALTLVQPSGSTAAAGTNQRNSPAISKSGTAAAQGKLKPTMLAPHQQQEADNTDVVDPWTLLEDGTSSGLSSSNASNSSDMANLRATCWLKGAVRVRRTDLTYVGSVDDDS

>AtMED13

MWTNVFRIGGLHNVSWFQFLPSETELNPGFDRSSRAEQNEVATYLVLSSHLRLQKEGFLTTWTNSFVGPWDPSQGLYNPDEKIKLWLFLPGRHSSISDKAQAAVSKLRVVASGIWVAPGDSEEISVAFSQSLRNCIERALSGISYMRFGDVFSKFSPQSEEYLRRGQPTVEFIFAATEEAVFVHVIISAKNVRTLSSGDAERMLRSSLKNSSYRLPVIVSPHGMRGSLTGFCPNDLVKQVYFSSGNLKTSTGYVGLPSHIGRGSRLINGNHCYVEVTLGCCQNRNDNTSQANSTFAVNLPHNQCPEPSVGSKDHRKGQSDLSSVCEKKFIYPAEAVLVPILQSAFAKFSLKRFWLQNWIGPSLAGSSLFMHWAGDFDCLGASENKSDGFYEKNGYNSSGSSRNSSISSTSSASSGSGWRMTSRTGDLDADADSLTCRQSGLTCNDDRLKMGSKRPRTGMAESFGQVGIENDQIGWDWDADDDDDDREVGMDIKALLSEFGDFGDFFENDALPFGEPPGTAESHVLVFPPDSADVGSSPVDMMDVSDQIVLPVGFSSFESFNPVPPIIDECLIKSQEVLHSSITSVPSNQMSISSTGEFDHLLKAEAMMTFAPEYGAVEAPMSEISSTFFKSPYLPKSHKVESSNSRTSNYVYGATPPTTDSDGAGDMILFGSKSCIGNNAGRTLYHSREHYTQVEGRKGRHDKLPTVISDNSSTKEGVSQSIHSKHSAANAVKVVQGKKTDGISAVVSTLLSSKTLLATDVGSVMFQAFMCRMRHIITSSKHSSPVSLTRLSGNFFLNQLSNEPSTLTDNISARNEIYKKDIPTRIAGDFDGGMLDSHMSAPVGVWRTVSVPKTAKPASSPNIEAGSSLPHSSFSEDSLLSYGQRQPLQDLLDGIALLVQQATSFVDLALDSDCGDGPYGWLALEELWRRELSCGPSAGHAGCGGTLASCHSLDIAGVKLVDPLSAEVFPSSVITLLQSDIKTALKSAFGQSDGPLSVTDWCKGRNQSGDGGSISEGFTAESALSEVSNAIDGGKGDETAQSQDIYSSELLRPTLFVLPSPAILVGYQDDWLKISTNSLTHWEKAPFEPYALPKSINYAVVCPDIDPLTCAATDFFQQLGTVYETCRLGTHLPQSLGNQMEKDVGRLSSSGFVLLDCPQSMKIESNNTSLLGSLSDYFLSLSNGWNVNSYLKSLSKALKGLKLGSGLYTNQKEGSGSPCVVVYIVCPFPDPSAVLRTIVESSIALGSVIQSDRDRRSILNSQVARAFSSSTAVDEASISHIPVLSGFSVPKLVLQVVSVDSIFRITSPSFNELVILKDTAFSVYNKARRISRGMPNDAFFSSSLPSRSSSALTPMNSISGIWKDCGGSRMTGSTHPRDGEIDVSLRTSGWDTSTSWQIPRSGGLSCDPNRNGDFYLNDEIFYLFEPLFILSEPGSVERGVSPTFTSLGSESSKPIPEDGGRGSGPGMNSMEGITSGSSSQGDVSQLEGKAIPSLHCCYGWTEDWRWLVSIWTDARGELLDTHIFPFGGISSRQDTKGLQCLFVQVLQQGCQILQACSSPDNGSFKPRDFVITRIGNFFELEYQEWQKAIYSAGGPEIKKWPIQLRRSAPSGIATNSNGSSLQQQDLSLIQERASSTSTLYSSHSKQSTFVKGSMGQSAGRKQIMGGQTISGTPRGLFQWVHSISFASISLDHSLHFVLPAELVSAGGGQSSTGMSSVNYIEGFTPVKSLGSTAFSYMMIPSPNMRFLHPSPLQLPTCLTAESPPLAHLLHSKGYAIPLSTGFVVSKAVPSMRKDSRINVKEEWPSVLSVSLIDYYGGYDNAHDKILQGIVKQGGGTKETRDFEVESHLILESIAAELHALSWMTVSPAYLDRRTALPFHCDMVLRLRRLLHFADKEVSRIPDKTGLKVLTTDSGSQSLSMSLPRDHLDGICFQHTDCNFLRCCSPFLSGHIRRDGEQVNGQIKTAAIYYGVSLFPGKIDNLNAMVWSHSCCRPLSSMHHMA

>AtMED14

MAELGQQTVDFSALVGRAAEESFLSFKELVDKSKSTELSDTEKKVSLLKYVAKTQQRMLRLNALAKWCKQVPLINYFQDLGSTLSAHDICFTQAADSLFFMHEGLQQARAPVYDVPSAVEILLTGSYQRLPKCLDDVGMQSSLDEHQQKPALRKLEVLVRSKLLEITLPKEITEVKISKGTVTLSVDGEFKVLVTLGYRGHLSMWRILHLDLLVGERSGPIKLEVTRRHILGDDLERRMSVAENPFTILYAVLHELCVAIVMDTVIRQVRALLQGRWKDAIRFDLISDTGTTPANQEGEADSVSLRTPGMKLFYWSDSDKNSGPFIKIEPGSDLQIKCSHSTFVIDPLTGKEAEFSLDQSCIDVEKLLLKAICCNRYTRLLEIQKELLRNTRICRTPSDVILQALLDEPGIEGDNMVDSKERVEPEVLRVRAYGSSFFTLGINIRTGRFLLQSSKSILTSSILEEFEDALNQGSISAVDAFINLRSKSILHFFAAIGKFLGLEVYEHGFGINKVPKSLLDGSSILTLGFPDCESSHLLLMELEKDFTPLFKLLETQMDGSGKPQSFNDPSNILRAKKIDIGQIRILEDDLNLITSDVVKFVSSFSDAEGINQASGHRQPGLVDEALTEMSGSQLSFSSVVDGVFGLQKVTSALMSIDGHGLVPKNLSAVTGHGKAPMLTSYHSDSLYNRQGPLQSSSYNMLSSPPGKGSAMKKIAISNSDQELSLILSPSLSTGNGVSESGSRLVTESSLSPLPLSQTADLATSSAGPLLRKDQKPRKRSASDLLRLIPSLQVVEGVASPNKRRKTSELVQSELVKSWSPASQTLSTAVSTSTKTIGCSYGNLIAEANKGNAPSSVFVYALLHVVRHSSLSIKHAKLTSQMEALDIQYVEEMGLRDAFSDIWFRLPFAQNDSWQHICLQLGRPGSMCWDVKINDQHFRDLWELQKGSKTTPWGSGVHIANSSDVDSHIRYDPEGVVLSYQSVEADSIKKLVADIQRLSNARMFSLGMRKLLGIKPDEKTEECSANSTMKGSTGGKGSGEPVDRWRAFKIEAVGLTSLWFSFGSGVLARFVVEWESGKDGCTMHVSPDQLWPHTKFLEDFINGAEVESLLDCIRLTAGPLHALAAATRPARASTATGMPVVPATASSRQSNQIQQTQGIIAPSTLAAPNATGQSASATSGNTVASSAPSPLGGGFHGVAMLAAAGRSGPGIVPSSLLPIDVSVVLRGPYWIRIIYRKRFAVDMRCFAGDQVWLQPATPPKGGASIGGSLPCPQFRPFIMEHVAQELNGLEPNLTGSQGATNPNSGNPTVNGVNRVNFSPSSARAAMNRVASVASGSLVVSSGLPVRRTPGTAVPAHVRGELNTAIIGLGDDGGYGGGWVPLVALKKVLRGILKYLGVLWLFAQLPDLLREILGSILKDNEGALLNLDQEQPALRFFVGGYVFAVSVHRVQLLLQVLSVRRFHHQAQQNGSSAAAQEELTQSEIGEICDYFSRRVASEPYDASRVASFITLLTLPISVLREFLKLIAWKKGLSQSQQAGEIAPAQRPRIELCLENHSGTDLDNNCAAKSNIHYDRPHNTVDFALTVVLDPVHIPHINAAGGAAWLPYCVSVRLRYTFGENPSVTFLGMEGSHGGRACWQRVDDWEKCKQRVSRTVEVNGSAAGDLTQGKLKLVADSVQRTLHLCLQGLREGGNNNNNTHQKEFTI

>AtMED15-1

MDNNNWRPSLPNGEPAMDTGDWRTQLPPDSRQKIVNKIMETLKKHLPFSGPEGINELRRIAARFEEKIFSGALNQTDYLRKISMKMLTMETKSQNAAGSSAAIPAANNGTSIDSIPTNQGQLLPGSLSTNQSQAPQPLLSQTMQNNTASGMTGSTALPSSMPPVSSITNNNTTSVVNQNANMQNVAGMLQDSSGQHGLSSNMFSGPQRQMLGRPHAMSSQQQQQPYLYQQQLQQQLLKQNFQSGNVPNPNSLLPSHIQQQQQNVLQPNQLHSSQQPGVPTSATQPSTVNSAPLQGLHTNQQSSPQLSSQQTTQSMLRQHQSSMLRQHPQSQQASGIHQQQSSLPQQSISPLQQQPTQLMRQQAANSSGIQQKQMMGQHVVGDMQQQHQQRLLNQQNNVMNIQQQQSQQQPLQQPQQQQKQQPPAQQQLMSQQNSLQATHQNPLGTQSNVAGLQQPQQQMLNSQVGNSSLQNNQHSVHMLSQPTVGLQRTHQAGHGLYSSQGQQSQNQPSQQQMMPQLQSHHQQLGLQQQPNLLQQDVQQRLQASGQVTGSLLPPQNVVDQQRQLYQSQRTLPEMPSSSLDSTAQTESANGGDWQEEVYQKIKSMKETYLPDLNEIYQRVAAKLQQDSMPQQQRSDQLEKLRQFKTMLERMIQFLSVSKSNIMPALKDKVAYYEKQIIGFLNMHRPRKPVQQGQLPQSQMQPMQQPQSQTVQDQSHDNQTNPQMQSMSMQGAGPRAQQSSMTNMQSNVLSSRPGVSAPQQNIPSSIPASSLESGQGNTLNNGQQVAMGSMQQNTSQLVNNSSASAQSGLSTLQSNVNQPQLSSSLLQHQHLKQQQDQQMQLKQQFQQRQMQQQQLQARQQQQQQQLQARQQAAQLQQMNDMNDLTSRQGMNVSRGMFQQHSMQGQRANYPLQQLKPGAVSSPQLLQGASPQMSQHLSPQVDQKNTVNKMGTPLQPANSPFVVPSPSSTPLAPSPMQVDSEKPGSSSLSMGNIARQQATGMQGVVQSLAIGTPGISASPLLQEFTSPDGNILNSSTITSGKPSATELPIERLIRAVKSISPQALSSAVSDIGSVVSMVDRIAGSAPGNGSRASVGEDLVAMTKCRLQARNFMTQEGMMATKKMKRHTTAMPLSVASLGGSVGDNYKQFAGSETSDLESTATSDGKKARTETEHALLEEIKEINQRLIDTVVEISDDEDAADPSEVAISSIGCEGTTVRFSFIAVSLSPALKAHLSSTQMSPIQPLRLLVPCSYPNGSPSLLDKLPVETSKENEDLSSKAMARFNILLRSLSQPMSLKDIAKTWDACARAVICEYAQQFGGGTFSSKYGTWEKYVAAS

>AtMED16

MNQQNPEEEVSLVNNSGGGGIIEAPAIVEEKEEEGLQQKQEETIESTDPILVVVEEKLLEKSVDGEKEDDNSSSSNMEIDPVSPATVFCVKLKQPNSNLLHKMSVPELCRNFSAVAWCGKLNAIACASETCARIPSSKANTPFWIPIHILIPERPTECAVFNVVADSPRDSVQFIEWSPTSCPRALLIANFHGRITIWTQPTQGSANLVHDATSWQCEHEWRQDIAVVTKWLTGASPYRWLSSKPSSGTNAKSTFEEKFLSQSSESSARWPNFLCVCSVFSSGSVQIHWSQWPSNQGSTAPKWFSTKKGLLGAGPSGIMAADAIITDSGAMHVAGVPIVNPSTIVVWEVTPGPGNGLQATPKISTGSRVPPSLSSSSWTGFAPLAAYLFSWQEYLISEIKQGKKPSDQDSSDAISLSCSPVSNFSAYVSPEAAAQSAATTTWGSGVTAVAFDPTRGGSVIAVVIVEGQYMSPYDPDEGPSITGWRVQRWESSVQPVVLHQIFGNPTSNFGGQVPTQTVWVSRVDMSIPPTKDFKNHQVAAAGPSVDAPKEPDSGDEKANKVVFDPFDLPSDIRTLARIVYSAHGGEIAIAFLRGGVHIFSGPTFSPVENYQINVGSAIAAPAFSPTSCCSASVWHDAAKDCAMLKIIRVLPPALPRNQSKVDQSTWERAIAERFWWSLLVGVDWWDAVGCTQSAAEDGIVSLNSVIAVMDADFHSLPSTQHRQQYGPNLDRIKCRLLEGTNAQEVRAMVLDMQARLLLDMLGKGIESALVNPSALVFEPWRVDGETITGINPEAMAVDPALVSSIQAYVDAVLDLASHFITRLRRYASFCRTLASHAASAGTGSNRNNVTSPTQNASSPATPQVFPDKSLYLAVGQPTTTTTTTATTNSSGSSHVQAWMQGAIAKISSSNDGSNSTASPISGSPTFMPISINTGTFPGTPAVRLIGDCHFLHRLCQLLLFCFLQRSSRFPQRNADVSSQKLQTGATSKLEEVNSAKPTPALNRIEDAQGFRGAQLGTGVKGIDENSARTTKMGSGNAGQGYTYEEVRVLFHILMDLCKRTSGLAHPLPGSQVGSGNIQVRLHYIDGNYTVLPEVVEAALGPHMQNMPRPRGADAAGLLLRELELHPPSEEWHRRNLFGGPGSEPEDMILTDDVSKLSNSLDLPDTNFSGICDGYNRVHSLWPRKRRMSERDAAFGSNTSVGLGAYLGIMGSRRDVVTATWKTGLEGVWYKCIRCLRQTSAFASPGATKQPNPNERETWWTSRWVYCCPMCGGTWVRVV

>AtMED17

MDSDMEISLDRLPIKRLESIEENGAERFPSDVDYDDKRVSLIRRIDFAWALEEEDELKKKKQKKSSKDSVEQWKWKGMVENLQLAHQELTVIIDLIDTVQANDAVTVAGMTRPKPMPNEILSDLAVSTATKLQGYRNLGNYFKQSAKALEQKINREARFYGALIRLQRNWKVKRQRMLASNASNEGFTIDLSDSSLYDPTSGFRPSTLSTIRVDHDSAGMLAINVPQDSWYSLRFGYVGLNPIGNSNESDEHIDSTTGHDIPGTSEKLSASDDKYVKETHSLLREVHKSIFAEQLFDMLNREAFNEGVGFNISGLRENFMEMSIGQGASLFVSLHPSGKNPSIKKSESATLLIESSGRVEPAEGGDYRLKKLGFPNRTSYEIYLQQIFHEHAFGKAKDQLKSKSIRASNQTEKDSNSGLLDHFCLSLTHRIFSNRVLVHLESVVCKVPYLHLISHPTWNSRTSSWTVFMTVPPSIIPQGRSETQSPDGKRNLKTQFRTKVVVKDDCISVEAECTPNVVGLLKSSSCNLFSINKYECDVADLPVMILQQVASQIVCWLLEEARTVGTKASREFLSLSLEIVEGERVSLVAHVNPEDAKGCISWWLVMENGCTEEREGVSESRKLLGHLSLDVLYSVLMDLINLCGTGRNALERL

>AtMED18

MSMECVVQGIIETQHVEALEILLQGLCGVQRERLRVHELCLRSGPNLGVVSSEVRLLCDLDQPEPTWTVKHVGGAMRGAGADQISVLVRNMIESKVSKNALRMFYALGYKLDHELLKVGFAFHFQRTAHISVSVSSVNKMPKVHAIDEAVPVTPGMQIVDVTAPATSENYSEVAAAVSSFCEFLAPLVHLSKPSISTGVVPTAAAAAASLMSDGGGTTL

>AtMED19-1

MEPERLKFGGPRELCGAADLISQFKLVQHHEFFCKKSLPVSLSDSHYLHNVVGDTEIRKGEGMQLDQLIESISQSRETNIRIQPFDIDELQESFQLNDMTPVELPPAEKGAPTIPSKSKSESKDRDRKHKKHKDRDKDKDREHKKHKHKHKDRSKDKDKDKDRDRKKDKNGHHDSGDHSKKHHDKKRKHDGDEDLNDVQRHKKNKHKSSKLDEVGAIRVAG

>AtMED20-1

MPVKWVLHWQPNQGSTVSSQILNEATQCVESINGVKEGRWKATLNYYKPMLKDQANQLEFPRDFLGISLADQPNKYYFIIRTQRIVLEADSSIQLIMEKLQSYKSKVALYFDGFQYQLGDFRLRVGKVVPTHSENVRGIVMEVEYLPISSMEKAQKVMEEFLEIWNEALAKRSLPGKFVNIDLNFGEFGLGDIYTPQHTAVRYALVMAHMIATVQAVRG

>AtMED21

MDIISQLQEQVNTIAAITFNAFGTLQRDAPPVQLSPNYPEPPATTTVTDDATPFPEQPKQLSAGLVKAAKQFDALVAALPLSEGGEGAQLKRIAELQVENDLVGQELQKQLEAAEKELKQVQELFGQAADNCLNMKKPE

>AtMED25

MSSEVKQLIVVAEGTAALGPYWQTIVSDYLEKIIRSFCGSELNGERNPVSTVELSLVIFNSHGSYCACLVQRSGWTRDVDIFLHWLSSIQFGGGGFNEVATAEGLAEALMMFSPPSGQAQPSNDLKRHCILITASNPHILPTPVYRPRLQNVERNENGDAQAESRLSDAETVASYFAKCSVSLSVVCPKQLPTIRALYNAGKPNQQSADLSIDTAKNTFYLVLISENFVEACAALSHSATNLPQTQSPVKVDRATVAPSIPVTGQPPAPVSSANGPIQNRQPVSVGPVPTATVKVEPSTVTSMAPVPSFPHIPAVARPATQAIPSIQTSSASPVSQDMVSNAENAPDIKPVVVSGMTPPLRTGPPGGANVNLLNNLSQVRQVMSSAALAGAASSVGQSAVAMHMSNMISTGMATSLPPSQTVFSTGQQGITSMAGSGALMGSAQTGQSPGPNNAFSPQTTSNVASNLGVSQPMQGMNQGSHSGAMMQGGISMNQNMMSGLGQGNVSSGTGGMMPTPGVGQQAQSGIQQLGGSNSSAPNMQLSQPSSGAMQTSQSKYVKVWEGNLSGQRQGQPVLITRLEGYRSASASDSLAANWPPTMQIVRLISQDHMNNKQYVGKADFLVFRAMSQHGFLGQLQDKKLCAVIQLPSQTLLLSVSDKACRLIGMLFPGDMVVFKPQIPNQQQQQQQQLHQQQQQQQQIQQQQQQQQHLQQQQMPQLQQQQQQHQQQQQQQHQLSQLQHHQQQQQQQQQQQQQHQLTQLQHHHQQQQQASPLNQMQQQTSPLNQMQQQTSPLNQMQQQQQPQQMVMGGQAFAQAPGRSQQGGGGGQPNMPGAGFMG

>AtCdk8-1

MGDGSSSRSNSSNSTSEKPEWLQQYNLVGKIGEGTYGLVFLARTKTPPKRPIAIKKFKQSKDGDGVSPTAIREIMLLREISHENVVKLVNVHINFADMSLYLAFDYAEYDLYEIIRHHRDKVGHSLNTYTVKSLLWQLLNGLNYLHSNWIIHRDLKPSNILVMGDAEEHGIVKIADFGLARIYQAPLKPLSDNGVVVTIWYRAPELLLGSKHYTSAVDMWAVGCIFAELLTLKPLFQGAEAKSSQNPFQLDQLDKIFKILGHPTMDKWPTLVNLPHWQNDVQHIQAHKYDSVGLHNVVHLNQKSPAYDLLSKMLEYDPLKRITASQALEHEYFRMDPLPGRNAFVASQPMEKNVNYPTRPVDTNTDFEGTTSINPPQAVAAGNVAGNMAGAHGMGSRSMPRPMVAHNMQRMQQSQGMMAYNFPAQAGLNPSVPLQQQRGMAQPHQQQQLRRKDPGMGMSGYAPPNKSRRL

>AtCycC-1

MFLIDSFCGFYFHSKELKDPEEVNVVHPLDAQRGISVEDFRLIKLHMSNYISKLAQHIKIRQRVVATAVTYMRRVYTRKSLTEYEPRLVAPTCLYLACKAEESVVHAKLLVFYMKKLYADEKFRYEIKDILEMEMKVLEALNFYLVVFHPYRSLPEFLQDSGINDTSMTHLTWGLVNDTYRMDLILIHPPFLITLACIYIASVHKEKDIKTWFEELSVDMNIVKNIAMEILDFYENHRLFTEERVHAAFNKLATNP

>AtCycC-2

MAANFWNSSHYKQLLDPEEVDVVHDLDKERGISIDDFKLIKFHMSNHIMKLAQHIKVRQRVVATAITYMRRVYIRKSMVEFEPRLVALTCLYLASKAEESIVQARNLVFYIKRLYPDEYNKYELKDILGMEMKVLEALDYYLVVFHPYRSLSEFLQDAALNDVNMNQITWGIVNDTYKMDLILVHPPYRIALACIYIASVHREKDITAWFEDLHEDMNLVKNIAMEILDFYENYRTITEEKVNSAFSKLALKL

>MedtrMED2

MDSVVDSLKNAYQDFVDAAATVLEASNISGALDTAATDTALKSFKQKWELFKVACDQAEEYVQSVKQRVESESLVVDAEMLLESIEKLHN

>MedtrMED3

MQQTQQATAATFSTPTPPPSSTAEAPPKQVALAMDKLGQAERIIADIRIGADRLLEALFIAAGQPHQGNKPLQVFVKENASMQQHFKDLRSLGKELEEAGVLSETARSRKDFWGLHMPLVCPDGAVVAYAWKRQLAGQAGASAVDRTRLALKAFTDQKRRFFPHLDDGVEANESDSKKRCGSEEVTFEPKEVMSFLRTLPDVLQSLEKEVPNVKISTFERLDWLKCASTLTSSPNESSEEHNYRGSSKRKLGSMGMVAPEKVAVIELLCPSIFRVVISLHPAGSIDPDAVAFFSPDESGSYVHARGVSVHHVYRHITEYATIALQYFLGNQAETSLYSLVHWICSYQTLFSRPCSKCKKLLAMDKQSNLLLAPVHRPYWKFSFSKILSTISSKDQNSDTTMAYHIGCISEEV

>MedtrMED4-1

MLQHQVVQSPARLGLTNPNSPLIPNPTPPKLPPLQTTNHHQDRHLATPSPALLSLLPPLPRAQALLQQMASLSTKLFEVSPNKSLWVSAFRGSLPTFLSSQGQPRSSASLDSSPSTTKEILSLFTNLQTQMFEAVSELQEVLDQKDAKQKIDQEICSKDSALLAFANKLKDAERVLDILVDDYSDYRSKTKRLKLGDGSEDVSLTTSTVSSQLKLSDILSYAHRISYTTFAPPEFGAGQAPLRGALPPAPQDEQMRASQLYNFADLDIGLPKSAETTEKTIEAIIEPPPLQPVDANSLANLPGIQGLLPPNFTVPAGWKPGMPVQLPIDIPIKPPPGWKPGDPVALDSLSIPRIEEQQLHPHVPQPKLPEIIQVAPVNLDLGESDSSDYSSDDASTDDED

>MedtrMED4-2

MLQHQIVQSPARLGLTNPNSPSILNPNPQKLPPSQTNHHQDRHSATPSAALISLLPPLPRAQALLSQMASLASKLFEVSPNRSVWVTAFRGSLPTFLSSQTQPHSFSSLESSLPSTTKEIISLFTTLQTQIFEAVSELQEIIDLQDAKKKIDGEIRSKDSALLAFANKLKDAERELDILVDDYSDYRRSIKRLKSGDGSEDDSLTTSTVSSQLKLSDILSYAHRISYTTFAPPEFGAGTAPLRGAMPPAPQDEQMRASQLYNFADLDIGLPKAVETKEKTVEAIIEPPPSVDTNPLGNLSAIQGMLPPNFAIPPGWKPGMPVQLPIDMPIKPPPGWKPGDPVALPPIDSLPVPRFEEQKLPPHIPQPRQPEIIQVQHVNLDLGGESDSSDYSSDEASSDDED

>MedtrMED6-1

KMIGIEYVLSEVMEPHLFIMKKQKRDSPDKVTPMLAYYILDGSIYQAPQLSNVFAARIGRALYYIEKAFTTAASKLEKIGYVDSENETTIPEPKAAKETIDLKRN

>MedtrMED6-2

MATPGMGMLDGGVPTAQPPGTDMTGICFRDQLWLNTYPLDRNLVFDYFALSPFYDWTSNNEQLRMRSHHPLDSSQLTKMIGIEYVLSEVMEPHLFIMKKQKRDSPDKVTPMLAYYILDGSIYQAPQLSNVFAARIGRALYYIEKAFTTAASKLEKIGYVDSENETTIPEPKVAKETIDLKEIKRVDHILASLQRKLPPAPPPPPFPEGYVPPSTAETEKGPETQEAAESLAPTVDPILDQGPAKRMKF

>MedtrMED7-1

MATATYPPPPPYYRLYKDYVQDPESAPEPPPPIEGTYICFGGSYTTSDVLPSLEEQGVRQLYSKGPNIDFKKELRSLNGELQLHILELADILIERPSQYARRVEEISTVFKNLHHLLNSLRPHQARATLIHILELQIERRKQAAEDINRRREEARRILNESLATLDGH

>MedtrMED7-2

MATATYPPPPPYYRLYKDYVQDPESAPEPPPPIEGTYICFGGSYTTSDVLPSLEEQGVRQLYSKGPNIDFKKELRSLNGELQLHILELADILIERPSQYARRVEEISTVFKNLHHLLNSLRPHQARATLIHILELQIERRKQAVEDINRRREEARRILNESLATLDGH

>MedtrMED10

MDSSQSPAAGGNGTLISHGNDAAASASGADDSMQNLSQISNSIEKTLGLIHQLSLTVSTFNSALQMPLLQRINGLVAELDNMVKLAEKCNIQVPMEVVNLIDDGKNPDEFTKDVINNCIAKNQITKGKTDALKDLRKHLLEELEQNFPDEVETFRENRAAAAAELKRLAQAPSVLPNGDARAKVEH

>MedtrMED11-1

MDSQGQTTSLQRLQNVEKRIVKVLELAGGVMDELASPVGPRKDLVQNHCLEFMQLIKDIQVTLRDEIKSACEYRPFEKCDYGPRIANEIGFKKVEYVMSQLEAMKQTINEYNAAA

>MedtrMED11-2

MDSQGQTTSLQRLENVEKRIVKVLELAGGVMDELASPVGPRKDLVQNHCLEFMQLIKDIQVALRDEIKSACEYRPFEKCDYGPRIANEICFKKVEYVMSQLEAMKQTIDEHNAAA

>MedtrMED12-1

MQRYHAGSCTSAVNNSTIGGPSSRDTGRSDSSFPAHFPVNSRSSIHKSNPDLITMGSRSKQNIKGQPKRFPIMRRQPQLNINPYKLKCDKEPLNSRLGAPDFLPQTPNCPEETLTKEYLQSGYRDTVEGLEEAREISLTQVPHFNKPIVHNCREAIKKRLRAINESRAQKRKAGQVYGVPLSGLQLAKPGIFPELRPCGEDFRKKWIEGLSQPHKRLCTLADHVPHGYKRSSLLEVLIRNNVPLLRATWFVKVTYLNQVRPGSVGISSGTADKIQPSRTEIWTKDVIHYLQTLLDEFFSKNNSHSTLQNRERSPQMPYAGTLLHKSDPLSSFSAGEEPSSHFRWWYIVRLLQWHHAEGLILPSLVIDWVLNQLQEKDLLEVWELLLPIIYGFLEIIVLSQSYVRALAGLTLRVIRDPAPGGSDLVDNSRRAYTTYALIEMLQYLILAVPDTFVALDCFPLPSSVVSHTMNDGSFVLKSSEAAGKIKNSSDYFGRIVLCIQKRAEDLAKAASPGNPSHCLAKVAKALDNSLVLGDLREAYKFLFEDFCDGTVSESWIVKVSPCLRLSLKWFGTVKTSLIHSVFFLCEWATCDFRDFCTTPPCDIKFTGRKDLSQVHIAVRLLKMKLRDVKSSLRRAKRSIHRASYAAKHESQRHNQNYITTGSSVISESPGPLHDIIVCWIDQHVVHKGEGLKRLHLFIVELIRAGIFYPLAYVRQLIVSGIMNMDVNVVDVERRKRHSHILKQLPGHFMRDALTESGIAEGPQLIEALQTYLTERRLILRNSPSDPRDDANANNAKLPTLKRKRNPASSKDGTSTVSTDQWKTVQSTASSKSAKDGASMEELKETISVLLQLPNSLSNLNSTGCDESESSVRKPTWPHYGKTEPVEGTPGCEECRRAKRQKLSEERSSSVPADDDTWWVKKGLKSTEPLKVDQPQKTTKQVTKSRQKNVRKMSLAQLAASRIEGSQGASTSHMCENKVSCPHHRTPVDGDALRSVDSMRTCDSRDIVFIGKALKKLRFVEKRAVAAWLLTVVKQVIEETEKNIGKVGQFGRAYSMVDDRNSIRWKLGEDELSAILYLIDISDDLVSAVKFLLWLMPKVLSSPNSTIHSGRNVLMLPRNAENQVCDVGEAFLLSSLRRYENILVAADLIPEALSSTMHRAATLIASNGRVSNSGATAFTRYLLKKYSNVASVIEWEKTFKSTCDARLSAELESVRSVDGELGLPFGVPAGVEDPDDFFRQKISGSRLPSRVGAGMRDIVQRNVEEAFQYLFGKDRKLYAAGIPKGHALEKWDNGYQIAQQIVMGLIDSIKQTGGAAQEGDPSLVLSAVSAIVGSVGPTLAKMPDFSSSNNQSNIMSLNYARCILRMHITCLRLLKEALGERQSRVFDIALATEASNVFAGVFAPSKASRAQFQMSSAEVHDTSATNSNELGNNSIKTVVTKATKIAAAVSALVVGAVIYGVTSLERMVTILRLKEGLDVIQCIRTTRSNSNGNVRSVGAFKADSSIEVHVHWFRLLIGNCRSLCEGLAVDLLGEPSISALSRMQRMLPLSLIFPPAYSIFAFVRWRPFILNANVAVRDDMNQLYQSLTMAIADAIKHSPFRDVCFRDCQGLYDLMVADGSDAEFAALLELNGSDMHLKSMAFVPLRSRLFLDAMIDCKMPPSIFTKDDVNRVSGPSESKTKLANGDSKLPDKLVHVLDTLQPAKFHWQWVALRLILNEQTLIEKLDTHDASLSDAIQLASPGPDKAASEKESNFIEILLTRLLVRPDAAPLFSQLVHLFGRSLQDSMLLHAKWFLVGPDVLVGRKTVRQRLHNIAESKKLSSKTQYWEPWGWCSPCTDPGTIKGDKKKFDAASLEEGEVVDEGIDMKKSLKRLSHVSQVFDSESSRINQQHVTERALIELLLPCIYQSSDESRNSFANDMIKQLCSIEGHIAAVTGGVKPVGSTPPGVEGQTNKVNTKKGIRGGSPGLARRPAGATDSSPPSPAALRVSVSLRLQLLLRFLPILCTEREPSVRNMRNSLAPVILRLLGSRVVYEDAYILSNAVHSKKDLELSSEAAASAFVDFSAEGLFDRLLLVLHALLSSYPASWLRLKPGSKSINEPTKEVSGFDREFLERMQNDLDRMQLPDTIRWRIQAAMPVLFPSTQSSFSCQPPLVPTSAVVCLQASNTIPGFNSSSSANPPLRSPVLSRVAANTSAKSKQQDSELDIDPWTLLEDGAGSCPSASNIASIGGGDHTNVRAASWLKGAVRVRRTDLTYVGAVDDDS

>MedtrMED12-2

MQRYHAGSCTSAVNNNTIGGPSARDIGRIDSSSLPPNFPVSSRRLPPPFTLYKLKCDKEPLNSRLGPPDFNPQTPNCPEETLTKEYLQSGYRDTVEGLEEAREILLTQIPHFNKTIVLNCKEAIRKRLRAINESRVQKRKAGQVYGVALSGSQLSKPGVFPEQRPCSEDFRKKWIEGLSQQHKRLRSLADHVPQYRRKSVLEVLIRNNVPLPRATWFVKVTYLNLVRSGSASVPSGGTNDKTQLSCSELWTKDIIEYLQTLLDDFFSKNTSHSIHNRDRSPQIPYMTSVKHRSNQLLPVSNGEEPSLHFRWWYVVRLLQWHHAEGLLLPSLVIDWVLRQLQEKQLLEIWQLLLPIVYGFLEIVVLSQTYVRTFAGVALRIIRDPAPGGSDLVDNSRRAYTTAALIEMLRYLILAVPETFVALDCFPLPSSVVSLAINDGNFAPKAIEAADKIKSSSADVCIFRSKGLDVRYESLAFDRVISSIQKHAENLTKAVSPGYPGHCLAKAAQALDKSLVLGDLCEAFKYLFEDLYDEPASDDWVAKVSPCLRLSLKWFANVNTSLIYSVFFLCEWATCDFRNFRTAPPCDIKFTGKKDISQVHIAVRILKMKLRNMHTLSTQMNGSTHHGAGYLAKCSSQQNNWNYGCKIKSSSKTMNQIIRSSIAFESPGPLHDIIVCWIDQHIVHKGEGLKRLHLFIVELICAGIFYPLAYVRQLIVSGIMDTSVNMVDLERQKRHRRIVKQLPGNFIRHALEESKIIEGPLLIEALHDYLNERRLILRGSFSENHDNASSANGFAVNQKHCTSSAKDGSSTVSIDQRKTIPSSKISYKAEKDGNGVDDLKKAISVLLQLPKSLSNLTITGLGESQGSVKRPFRCHNKIDVMEATPGCEECRRAKKQKLSEERSSFVQAHFPVLSDDEDTWWVKKELKPLEPLKVEQPLKTTKQVAKSRQKTVRKTQSLAQLAASRIEGSQGASTSHVCDIKVNCPHHRTAMDGDTTKFVDGIQTSQFEDIVSTGRALKRLRFVEKREITVWLMTVIRQLIGDTEKSIGKVGQFGRPVTTVDDRSSIRWKLGEDELSAILYLMDISDDLVPAIKFLLWLLPKVCSSPNSTSHSGRNVSMLPRNVDNQVCNVREAFLLSSLRRYENILATSDLIPEALSSVVQRATTIIASNGRVSGSGALAFARYLLKKYSNVVSVIEWEKNFKTTCDKRLASELEFGGRLVDAECGLPLGVPAGVEDPDDYFRQKISGGRLPSRVASGMRDVVQRNVEEAFHYLFGKDRKLFAAGTPKGPTLEKWDNGYQIAQQIVMGLMDCIRQTGGAAQEGDPSLVTSAVSAIVGSVGPSLAKLPDFSAGNNHPNASLATSSLSYAKCIMRMHITCLCLLKEALGERQSRVFDIALATEASNTLAGVFAPSKASRNQFQMSPETHDTSATMSNDAVNSSSKIVLARTTKIAASVSALIVGAIIYGVTSLERMVTVLRLKEGLDVIQFVRSSRSNSNGSARSVGAIKVDCSVEVHLHWFRLLVGNCRTICEGLVVDLLSEPSIVALSRMQRMLPLSLVFPPAYLIFAFVMWRPFIMNANVAIREDTNQLYQTLTTGINDAIKHLPFRDACLRDSQGLYDLMAADTSDLEFATFLELNGSDMRLNSTAFVPLRARLFLNAIIDRKMPQSIYTKDGGSQISGHGESKIQFTDSKSKLEDKLVDVLEALQPAKFHWQWVELRLLLNELSLIEKLQTHDMSLAKAIQLSSPSPEKAAASENENDFIQIILTRLLVRPDAAPLFSELIHLFGKSLVDSMLSQAKWFLVGQDVLVGRKTIRQRLINIAESQRFSIKPQFSEPWGWCSPCTDPITIKRDKRKVDSLSLEEGEVAEGVDVKRPLKGFSQVFDSEGSTIKQLHETERAFLELILPCIDQSSDESRYSFASDLINQLCSIEKLIAAVTRGPGKLAVSSPVTEGLTNKVNTRKTIKGGSPGLARRPTSSTDSSPPSPAALRASLSLRIQLIMRFLPILCTDREPSVRKMRNTLASVILRLLGSQVVLEDANILVNATHSSHSKRDVESPSNVACVAFLDSSVEGLFDQLLLILHGLLSSSPPCWLRLKAASKTANEPTRELLETLQNHLDCMQLPDSIRLRIQAAMPVLPPSTRCSFSCQPPSVANSSLASLQSNIKNSGSNSGSLATSQRSPVPLSRTAASGKSKQQDNDFEVDPWTLLEDGAGSCPSASNVGSGDRVNIRAASWLKGAVRVRRTDLTYVGPVDEDS

>MedtrMED13

MWTNVFKIGSLHQISWFQFLPHEPDLNPLPDKSVKADQKDAAMLVVLSSHLQLQKEGFLSAWTNSFVGPWDPSQGLHNPDEKIKLWLFLPGRHLTVSETAQPALTGLRAVASGLWLAPGDSEEVAAALSQALRNCIERALLGLYYMRFGDVFLKVHQFQSEELLRRGHPAFEFVFAATEEAILIHVIVSSKNIRMLSSGDLEKLLKHSMETTYTLPVIVSPHGIRGNLTGCSSSDLVKQSYFSSAKFRVSNGIIGLPYHVSQGVGCQLRGQNCFVEVSLGFPRSETDKALQSNKNIRNLLKSPVTGHNDGKGSPDHLSDNEKTFLYPAEAVLVPVFQTSLARSSLRRFWLQNWMGPSLAGSSSFIHCAGNVESTEDPWAEYNGSRTQNSYDSSSNSNSSSISSLSASSSDSDYKTTGPSELEADADSLACRQSMISSADRLGSDAKSGSKRSRTGLVQSLSTTTNIPGQDAYMSDFGSMEVNNSAITRVGNEPTGSYWDWDDNDEENRGMELNMEALLKEFGRGFDDFFVGDELPFGEPPGTAESQALMVYAPDCGDVNSSPVGADVMDVSDQMILPIGFSSFESFNPTSPAVMEECLNKDQDNLNNSMSTGQTNQTQMLYTGEFDHIMKAEAMITFAPEFGAVEAPTSGLSTTLFRSPYFPKFQKAESSNSCSNNYLYGAEPPSSPYIEGSEGKNGMVINTKTCSGKHDTSMTLHSKNYYTFVESRKDMNEKKPVTCIANNIAKSEGIVQPPFSSVGSNVSVKSVLRKMTEDPKDAERFTPLSAKTLLATDVTCAMLQASMCRLRHTLLSSGNNLVPVGLNRSTGVTFSNQLPTDPSTTTDNISGKYEVKKKENIPVRIAGDFDGGMLDGHLNAPVGVWRSVGTSKVVKPSNSPNMEVGPSFSHNSFNEEGILSYAQRKPLQELLDGIALLVQQATSFVDLALDADCGDGPYGLLALQEQWRRGFCCGPSMVHAGCGGTLASSHSLDIAGLELVDPLSSDVHASTVISLLQSDIKTALKSAFTNLEGPLSVTDWCKGRSQLVDPGSMVDGVSAESSISECRDSSEPLSPSQSSVCGSSSFKVSSLTGQDMCNSESEQQPCSRLKPTLIAVPFPSILVGYQDDWLKASANCMQHWEKAPLEPYALQKPIAYHVVCPDIDPLTSAAADFFQQLGTVYEMCKLGTHSPLVLGNQMETESAKLSSCGFVLLDCPQSMKIESSNASLVGSVSDYFLSLSNGWDLTSYLKSLSKALRALNLSSCFSANPTEGSNSSCLVIYVVCPFPDPSAILQTIIESSVAIGSVIQQSDRERRSNLHSQVVKALSGLATVDEASASNIPVLSGFSIPKLVLQIVTVDAIFRVTSPSVSELVILKETAFTVYSKARRISRGISSDSAQLAFSSRSQSVLPQMPSPISGMWKDCGGPRMAGHSLPRDGDIDTSLRPGNWDNSWQPTRSGVLNCDPSRTGDIFLHDEIRYMFEPLFILAEPGSPEHGISVVGSPGSEASKALADDSSGNHVQSTSTSGSVDSASSIDGSGSDQKTHPSLHCCYGWTEDWRWLVCIWTDSRGELLDSNIFPFGGISSRQDTKGLQCLFVQVLQQGCLILQSCDPGLAKPRDFVIARIGGFYELEYLEWQKAIYSVGGSEMKRWPLQLRKSLSDGVSSTSNGSSLQQPDMSLIPERTLPSSPSPLYSPHPKPTGFIKGNLGQSAGRKQMMGGHSTVDNSRGLLHWAQSISFVAVSMDHTLQPVLPADSSSPGYVEGFTPVKSLGSASSAYILIPSPSMRFLPPTALQLPTCLTAESPPLAHLLHSKGSALPLSTGFVVSKTVASMRKDYRSNLKEEWPSVLSVSLIDYYGGSSIPQEKNVRGINKQGGRSLNWEAKDFETETHLVLESLAAELHALSWMTVSPTYLERRTALPFHCDMVLRLRRLLHFADKELSKQSDKS

>MedtrMED14-1

MATAELGQQTVELSTLVTRAAQDSYNSLKELVDKCRSTELSDSDKKISMLKFLTKTQQRMIRLNVLSKWCQQVPLIQHCQQLSSTVSNHDMCFTQAADSLFFMHEGLQQARAPVYDVPSAIEILLTGSYERLPKCIEDVGSQYALTEDKQKPALKKLDMLVRSKLLEVSLPKEISDIKVSDGTAMVRVNGEFQVLLTLGYRGHMSLWRILHLELLVGEKNKTVKLEELRRHVLGDDLERRMAAAENPFSILYTVLHELCVALVMDTVIRQVQFLRQGRWKDAIRFELISDGGSGHGASSSSVQNPDGESDSSGLRTPGLKIIYWLDFDKNAGVSDSGACPFIKIEPGPDLQIKCTHSIFVIDPLAGKEAEFFLDQNCIDVERLLLRAICCNRYTRLLEIKTELIKNVQVFRTADDVVLQSHMGEPDIEYKQKDNKRCDKDSEGNEVLHVRAYGSSFFTLGISIRNGRFLLQSSQNIVVSSALLECEEALNQGSITAAEVFLSLRSKSMLHLFASIGRVLGLEVYEHGLNTVKTPKTLSNGSTVLMMGFPDCGSSYFLLMQLDKNFKPLFKLLETEPDPSGKDKIFGDLNQVLRFKKIDIAQMQVLEDEMNLSLVDWAKLHSILPNAACPNQMSGHGLYSDTRLQNSMHTARGHHPSGFSSLVDDVFGLEKGSSAPPFPVQNISSPLNSSLPSHYGSLPINSQSLKAGNIHYNSSLFSSGNVKGPVQSSSVGSVPTGHGRSAVGKKLSASKSEQDLASVKSPHSVDISSSTPMDEDTANDALSGSRSSLLSPSWPINSRMSAPSSRPNGSSSCATTLVSQGLDTVNFSTSEDVISEQDKKSRKRTASDMLNLIPSLQEFVKNQGICKRRKILDACSSQLALPQSSITPEIIPKAEGCSYGSLIAEANKGNAPSSIYVAALLHVVRHSSLCIKHARLTSQMDALEISYVEEVGLRRVSSNIWFRLPFARGDSWQHIFLRLGRPGCMYWDVKIDDQHFRDLWELQKGSSNTSWGSGVRIANTSDIDSHIHYDPDGVVLSYQSVEKDSIKKLVADIQRLANARTFSIGMRKLLGIRADEKSEEFLTNSDVKISGVKTASDTADKLQMRRAFRIEAVGLMSLWFSFGSGVLARFVVEWESGKEGCTMHVSPDQLWPHTKFLEDFINGAEVSSLLDCIRLTAGPLHALAAATRPARAGPVPGVAAIISSFPKQTGYTSSQGLLLGSSTSATNVGQPASGLGANTSVSNASGISNQNLSMLAAAGRTGPGIVPSSLLPFDVSVVLRGPYWIRIMYRKQFAVDMRCFAGDQVWLQPATPPKEGRPSGGSLPCPQFRPFIMEHVAQELNGLDPSFTGQQAGGLTSSNSPNPNSGTQSMAANGNRINSAAMSRTGNQVASLNRMGNALAGSSNLALMTSAVPLRRPPGTVVPAHVRGELNTAIIGLGDDGGYGGGWVPLVALKKVLRGILKYLGVLWLFAQLPDLLKEILGSILKDNEGALLNLDPEQPALRFFVGGYVFAVSVHRVQLLLQVLSVKRFHHQQQQQQQNSNPAPEELSPSEISEICDYFSRRVASEPYDASRVASFITMLTLPIPVLREFLKLIAWKKGLSQAQVGDVVSAQKPRIELCLENHAGLNADENSESSSAFRSNIHYDRLHNSVDFALTVVLDSAHIPHVNAAGGAAWLPYCVSVRLRYSFGESLNVSFLGMSGSHGGRACWSRVDDWEKCKQRVARTVEVSASSAADVSQGRLKLVADSVQRNLHMCIQGLRDGSGATASSGAT

>MedtrMED14-2

MATAELGQQTVELSTLVTRAAQDSYNSLKDLVHKCSCSTELSDTDKKISMLKFLTKTRQRMIRLNVLSKWCQQLPLIQHCQQLSSTVSNHDMCFTQAADSLFFMHEGLLQARAPVYDIPSAIEILLTGSYECLPKCIDDVGSQYALTQDKQKPALKKLDMLVRSKLLEVSLPKELSDIRVSDGTAMVKVDGEFQVLLTLGYRGHMSLWRILHLELLVAEKNKPVKLEELRRHVLGDDLERRMAAAENPFSILYSVLHELCVVLVMDTVIRQVQFLRHGRWKDNPDGESDSSGLRTPGLKIIYWLDFDKNADVADSGACPFIKIEPGSDLQIKCTHSIFVIDPLTGKEAEFLLDQNCIDVERLLLRAICCNRYTRLLEIKTELLKNVQVFRTADDVVLQSRMGEPDIEHKQKDDKRCNKDSEAHEVLHVHAYGSSFFTLGISIRNGRFLLQSSQNIAVSSALLECEEALNQGSMTAAEVFLSLRSKSMLHLFASIGRVLGLEVYEHGLNTVKNPKTFFNGSTMLMMGFPDSGSSYFLLMQLDKKFNPLFKLLETEPDPSGKDNIFGDLNQVLRFKKIDIAQMQVLEDEMNLSLVDWEKLHSILSNTACPNQMSGHGLYSDIRLQNSIHTARGHHASGFSSLVDDVFGLEKGSSVPPFPVQNISSPLNTSLPFHYGSLPKAGNIQYNGSLFSSGGVKGLVQSSSVGSLLTGQGRSTVGKKLPALKSEQDLTSVKSPHSVDISSYTAMDEDTANDALSGSRPSLLSPPWPISSQMSSPSSRPNATTPVSQGPDTVNFSSSEDVISEHDKRSRKRTTSDMLNLIPSLQGFVKNQGICKRRKISDPCGSQLALRQGSITPEMIPRAEGCSYGSLIAEANKGNAPSSIYVAALLHVVRHCSLCIKHARLTSQMDALEISYVEEVGLRRESFNIWFRLPFARGDSWQHIFLRLGRPGCMYWDVKIDDQHFRDLWELQKGSSNTPWGSGVRIVNTSDIDSHIRYDPDGVVLSYQSVEEDSVKKLVADIQRLANARTFSIGIRKLLVIRADEKSEEFHTHSDVKISGVKTASDSADKLQMRRAFRIEAVGLMSLWFSFSSGVLARFVVEWESSKEGCTMHVSPDQLWPHTKFLEDFINGAEVSLLLDCIRLTAGPLHALAAATRLARAGPVPGVAAALSSFPKQAGYISLQGLLLGSLSSTANVGHPASGLGANTAVSNASGIANQTLSMLAAAGRGGPGIVPSSLSPFDVSVVHRGPYWIRIMYRKQFAVDMRCFAGDQVWLQPATPPKEGRPSGGSLPCPQFRPFIMEHVAQELNGLDPSFTGQQAGGRTSSNSPNSGTQSMAANGNRINSAAMSRTGNQVASLNSMGNALAGSSTLALTTSAVPLRRPPGTVVPAHVKGGLNTAIIGLGDDGGYGGGWVPLDALKKVLRGILKYLGVLWLFAQLPDLLKEILGSILKDNEGALLSLDPEQPALRFFVGGYVFAVSVHRVQLLLQVLSVKRFHQQQQQQQQNSNPAPEELSSSEISEICEYFSRRVASEPYDASRVASFITMLTLPIPVLREFLKLIAWKKGLSQAQVGDVVSAQKPRIELCLENHAGLNADENSKSSSAFRSNIHYNRLHNSVDFALTVVLNSAHIPHVNAAGGAAWLPYCVSVSLRYSFGESLNVSFLGMSGSHGGRACWPRVDDWEKCKRRVARIVEVSASSTADVSQGRLKLVADSVQRNLHMCIQGLRDGSGATTSSGAT

>MedtrMED17

MEEGMELQLSLDKLPIKRLDSIEENGNERFPLDVDYDEKRVSLIRRIDFAWAIEKDEEKKKQKKSSKETTPWQWQGMVENLQLAHQELSVIIDFINTVETNDAVTVASMTRPKSLPNEALSDLAVSAATKLQCYRQVGKYFKQSAKAFEQQVAREARFYGALIRLQQNWKVKRQRQTSLVPGNEGFTFDLFDNSYDQGAIVRSSSMSTVRVNHDAAGMLAINVSPELCHSLQFGFVSAQPDDMQKKSNENQSHLLGEDCLGETGIESSSDEECVKKTHSLLRDVHQAIFNEQVFDLVNREAFNTSTGFTLTGIRENYLQLSLGQGTSVYLSLVSTGQDNPTVEGELTNNADDNAFSPLESSDVLMHDAQQNTLKKKGRHSNSTCYEIYIQQIYHEHIFGRGSEKPISSGNRLSGAQAKDGSYLLSHFFMSLAHRIFSTKILAELENVVFKVPYLQLISNPTWHSRGSSWTLFMEVPPSILRGCQVKTSDFENNAIKRQFWTKVVVIDDCISVKAEGSPNVSGLFKGKSEDTHSINKYDCNLADLPVIILQQVASQIINWLYHEALMVGIKANRDFLCLSFELEQGETLGLVANVDPKDSDGCISWSLVMEDSFAEVQKLHTNLTDGASEYRKFLGPLSLELLYATLIDLIAFVSGGGGGQ

>MedtrMED18

MECVVQGIIETQHVEALEILLQGLCGVQRERLRIHELCLKNGPHLGPVSSEVRLLCDLEQTEPSWTVRHVGGAMRGAGADQISVLVRTMVESKVSKNVLRMFYTLGYKLDHELLRVGFSFKFNRGAQITVRVSSVNKMLKLHATDEAVPVTPGIQMVEVTAPASDENYAEVAAAVQSFCEYLAPLLHLSKPGVSTGVVPTAAAAAASLMSDGGGTPL

>MedtrMED20-1

MPVRWILHWQPSQGAVVNSHILNEISQCVENLNGVKDGRTKATLTFYRPNLRDQSLSSEFPRDFLGISLMEQPNKYYFIIRGHKLAVEADSSILTIMEKLQSYKSKVTLNFEGTQYKLGDFQVRMIKVVPNQAENLRGILMEIEYLPNSSVEKVKPIMEEFIEIWRDVLSKKSLPGQFMRTEPIFVEYGLSDHYTWQHTAVQYAAALAQLIASVQVRN

>MedtrMED20-2

MPVRWILHWQPSQGTTVNSHILNEISQCVENFNGVKDGRCKTTITFYKPNLKDQSMSAQFPRDFLGISLMEQPNKYYLIIRDNKLVAEADSSILTIMEKLQSYKSKVALNCEGLQYNLGDFQMRLIKVVPNQAESLRGILMEIEYLPISSLENAKPIMEEFIEIWREVLSKKSLPGQFMRAEPIFADYGLSDNYSLQHTAVQYAAALPQLFASVQLRS

>MedtrMED21-1

MDIISQLQEQVNLIANLALNTVGTLQRDAPPNRLSPNYPEPPPHPTEDGANFSEEPKLMGASLVKAAKQFDLLVASLPISETGEEAQLKRIAELQAENDAVGQELQKQLEAAEKELNQVQELYRQATDNCLNLKKPDVN

>MedtrMED21-2

MDIVSQLQEQVNLIAHLASNTVGTLQRDAPSSQLSPNYPEPPAHTTSMDSANFSEQPKLMASTLMKAAKQAPFPVGDENRSPTVGDIKKW

>MedtrMED22

MNKGGPGGGGPTAAAAAAAAQKQKTLLQRVEGDIANIVDNFSHLVNVARVNDPPVRNSQEAFMMEMRAARMVQAADSLLKLVSELKQTAIFSGFASLNDHVEQRRLEFNQLAEKTDHTLSTIGEEAAASLKELESHYSSSAQKTIQDVQP

>MedtrMED23

MDQSQNQRSITTTTSQSRSLQFHPARVPILDLFNLYLGLGRNSRNKPDDSIREPPNKTQKRVHAINRELPPPNEQFIVDFEQLQSQFPDHEQLRSVTEAILIPLVVQCSGHGPRSDFLLFVLRSLCGIGCINWDTFLPALVSSVSSAEIPVGQTSQAVSTVTSSSLSQSGMLPPPSTIANSSNFQSSNPASPLTSVHTIGSPAQSSIEPLSCAALSPVKSSDISSAGQQSKQRGSPSVRNNDISYSSLRQLCCKIILTGLEFSLKPMTYAEIFNHMLNWLVNWDQGQQGVDESDNQKSWRPDNALVAWLHSCLDVIWLLVDEGKCRVPFYELLRSDLQFMENIPDDEALFTLILEIHRRRDMMAMHMQMLDQHLHCPTFGTHRILNQTTPTISGEGIAHLRLSPISYLSVLGEPLNGEDIAISIQKGSLDWERAVRCIRHALRTTPSPDWWRRVLVLASPYRPSAQGPTAGAVFSTEMICEATIDRIVELLKLTNSEINCWQEWLVFSDIFYFLMKSGCINFVDFVDKLVSRLAEGDHHTLKTNHVTWLLAQIIRIELVMTALNSDSRKVETTRKVLSFHREDRSSDPNSPQSILLDFVSSCQNLRIWSLNTSAREYLNNEQLQKGKQIDEWWRQASKGDRMMDYMNMDERSIGMFWVVTYTMAQPALETVMNWLTSAGVIDLAPATNLQPPERLVCTREVNPLPMSLISGFSMNLCLKLSYQMEDSLFSGQVLPSIAMVETYTRLLLIAPHSLFRSHFIHLGQKSPSLLSRPGVTLLVLEILNYRLLPLYRYQGKSKALMYDVTKIISALKGKRGDHRVFRLAENLCLNLIFSLRDFFLVKREGKGPTEFSETLNRVTVVTLAILIKTRGIADADHLPYLQNMLEQIMATSVHTWSEKTLRYFPSVLREALSGRPDKRSLAIQTWQQAEQTVIHQCNQLLSPSADSNYVMTYINHSFPQHRQYLCAGALILMQGHAENINSGNLGRVLREFSPEEVTSNIYTMVDVMLHHMQIELQQGHLIQDLMLKAISSLAFFVWTNEILPLDILLLALTDRDDDPHALRIVISLLDRQELQQRVKLFCLTRGHPEHWLYTGMFKRVELQKALGNHLSWKDRQPVYFDDIAARLLPIIPLIIYRLIENDAMDTAERLLALYSPLLAYYPLRFTFVRDILAYFYGHLPGKLIVRILNVLDISKIPFSESFPQQISSPNPAMCPPHDYFTTLLLGIVNNVIPPLHNNSKSGSIGDSSNGAQRTSQSKPPAVSQSGPANASESQKAFYQIQDPGTYTQLVLETAVVEILSLPVSASQIVQSLVQIVVNIQPTLVQSNDSFNGSSNGVGQGSVLPTSPSGGSTDSIGASRSTPSTSGVNTTNFASRSGYTCQQLSCLLIQACGLLLAQLPSDFHSQLYLETTRIIKENWWLTDVKRSLAEIDSAVGYALLDPTWAAQDNTSTAIGNVVALLHSFFSNLPQEWLEGTNVIIKQLRPVTSVAMLRIAFRIMSPLLPKLANAHALFNKTLSILLSVLVDVFGKNSQTSTAVDASEIADIIDFLHHVIHYEGQGGPVQASSKPRPDVLALIGRAAESLRPDIQHLLSHLNTDVNSSVYAASHPKLVPNPT

>MedtrMED25

MGLSYWAIIRSGTVLKRYFTYFVNFEGLKGERAQANGSITTGMPQQEVESDMQPLENDATAAQQTASSAQSKYTRFWKGSLTELRQGQRVLITKLECSRSSSASKTLTTNWPSDMQIVRLISQERMTIHKQHARKEDLLVFRPVNPGRSLSHLKEKKLGAVIQLPSQTLLLFVSEKPNQLIGMFIPQVTYSR

>MedtrMED26-1

MGYEDNPYRDEDGEPLMDYDDVQSDGEGSPEPQQLDDFEEEDVDNFHDRARSQTPVYENDSSKSKPRKRLIKKSDTGKQSMALPSELEDELEEEDEGRKRKKGKDGGSGKKEKRLKGGSSSSGKGGSRFGGSKRGVGGKSGNDREGEVNEMWDALAGNSEDDNEGARNMDDDNFIDDTGVEPALYGYDEPRSPGDAPQAEEGEEDDEIKDLFKMGKKKKKNERSPAEIALLVENVMAELEVTAEEDAELNRQHKPAVNKLKKLPLLIEVLSKKQLQLEFLDHGVLNLLKSWLEPLPDGSLPNINIRTAILKILNDLPIDLEHYDRREQLKRSGLGKVIMFLSRSDEEINVNRRLAKDLVDKWSRPIFNKSTRFEDMRNTEDDRVPYRRPSVKKPAAKAAGMQSRDGDLDLDLSQPRSGESSSRQHASRPEATPLDFVIRPQSKIDPDEIRARAKQATQDQHRMKMNKKLQQLRAPKKKQLQATKLSVEGRGMAKYF

>MedtrMED26-2

MTQMKSESLDQWRSYFRSSNSDIFDIIDHAIVVAASDCPKEFKSRRDGIAERLFSVMLNRCKGCEKVELSVPDDDNGDELCKRSSVREGASKESKVDCSREENGVMDANPISNYSFGEAEALTDELEEQSQLVAEVTRIKEILNNHEDESDSVLFESLRRLQLMQLCVDLLKSTEIGKAVNHLRKHGSKDIRQLARTLIDGWKELVNAWVKATTTTPVAASEEGTPDSRNPSVVENEEEEEGGLPSPPLDEGAFFVAPTGTIELSQFFDGMDDDGNIRKSAPFNKNRDNGRKPALGTQTKDKRNFPASNETAVTAKDNKSQHMKKNEAAARPNKPMAADTGRARPLNSNIQRKANVEPKMPPKIENSTVPKRPLNAQQDKSKCSDDLAKLEATKRRLQESYQQAENAKRQRTIQVMEINDLPKQGSVQRNTHFKPGNHNRQWGNGRR

>MedtrMED26-3

MYGFGGEKKWKHNRHMWPVPSNPTTVVTVSSPSKFICKDGRKIRVGDCALFKPPQDSPPFIGIIRKLIFNKEESPSLEVNWFYRPADLKLSKGIAVEAAPNEVFYSFHKDETHAASLLHPCKVAFLRKGVELPSGISAFVCRRVYDIDNNCLRWLTDKDFVNDQQEEVDHLLDKTKLEMHGAVQSGGRSPKPLNGPTSTQSLKSSSDNIQNSSSFGVQGKGKKRERGDQGSDSSKKERLFKVEVGDSGQFRLEMLRTEIARITEKGGLVDFEGVEKFVQLMQPDSADKKIDLAGRTMLVDVISLTEHYDCLGWFVQLRGLPVLDEWLQEVHKGKIGDGNVDKSEEFLLALLRALDKLPVNLHALQTCNVGKSVNNLRSHKNSEIQRKARSLVDTWKKRVEAEMNMTDTKPGSTRAVSWPTKPSPSDVSHSGNRRTGGSSENVAKSSPIQPSASKNSQSKHNSGEVLSKFSSSPGSTKSMTASVGSNTKDQNVKLIVGATTSDLPLTPIKEERSSSSSQSQNNSRSCSSEHAKSSTAGSISGSKIPGNASRSRKSSNGIHGAGVAVVLKDHSSAKNSTRNSPSDKVSPTRMSHEKPSDQPLSDQGHNQRLIVRLPNTGRSPSRVASGGTFEEPAITCGKTSPPADKNENQDRRVKAKTDCLQTHVSNVMNDASNAKEITGCDEAKCSPRVDERCRANEDGDKVAETSKTTSSSTGFVSRSGQTYDASLSPMNALVESCVKFSEASSSVSPGDDGMNLLASVAAGEMSRCENVSPLPSPERKSPAADESSSGNDRHSFEAAGRTQRQSDGGATGEHPVNTLQFKNNSRHLVTMVSRDFSDGEAVSSSCVEKTGDGKKQVNFSTTDAIQNTEGSCLRPDTKEDTSETVFPGRKESHAEAGGAEGFHERRESGTQWPKNSTSPGSKKLRTSSFDDDQKTDNKDGGLTEHGKMLVSETVASGMIENEPGKISPELSSGVDSKSQISAEKVTGIIPVQKGSPVADTCESIDVKREDVMLPASGSALTVSRDENTNNVMAVESKPSEKRMDLDSAVADGVDERCEENSVRKELIGSSSLNSDIPITSEKENEVPETCDSNIEGKKSVVAAELNAGNANTSPIASGSDAAVKLDFDLNEGYPVEDAGQGDIVRQEDPTSSSAVHVPCPLPFPIPSLSGAFHASITVASATKGPVVLPENPLRSKGELGWKGSAATSAFRPAEPRKNAEMPSNTGDIPSVETTSVKQGRAPLDFDLNVADEIGFEDVGYRGSLESGAHDRSAVGLDLDLNRLDETPEAGSFAMGKMDIPSLPSKQPSLSSGLSNGGSVSRDFDLNNGPGLDEVSTEVPARSLQMKGPVPFSSSVHGTRTNNAEFGNYSSWFPPGNSYSAITVPPLLPGRGEQSYVGASGPQRIIGSTGSSPFSPEMYRGPVLSSSPAVAYPPTTPFPYPGFPFETNFPLSSNAFSGCSTPFMDSSTVSGLCFPTMPSQPVGPGGIVSSTYPRPYVMSLPGSTSNVIPDSRKWGSQSLDLNSGPGGTDAERRDDRLPSGLRQMPVPSPQALMEDHLKMFQMAGALKRKEPDGGWDGTDRFSYKHPPSWQ

>MedtrMED26-4

MDLDDFRSILHTAGVDVWMFIDTAISVAAQDNAGELKRRRDGIVERLYAASTEGIPMCQNCDGGQRLVTNDNQIKKENSPSLSPERQPRRGGASSPPTPQSEGNEDGEDEEIDPYGGLFDDEQKKILEIKELLEDPHQSEDTLMELLQNLVDIDITFQELKETDIGRNVNQLRKHPSSDVRRLVKLLVKKWKEIVDDWVKQNPQRGKSTLMADGDSPLQKTTPNGHNHQIPDFAYSPNPHNGSSGSDRNTSEAEPKPKPKSVPRKDPPPKPRPSPPVTAPTSAPQNRQREQKESNFDAERLAAARKRLQANYKEADNAKKQRTIQVMDIHELPKSKAKSGYFAKHKGGGSSQGRQHW

>MedtrMED26-5

MEKEKEKELVELYEAAKKAADASTSTDNSPSEETRCLDALEQLKNFPVNYKILVNTQVGKHLKTLTKHPRENIRAFAVDLIAIWKDVIIKETSKNKNGASDSKVESTNGERAKAGKLQKSPSVKVEKGESAKVEKVNGNGSSKLSSGNVKAQNVDVKIEKTDRTSNIKAKEEKPVSAAKKISSSAAAPPKLKTMIKSNDSARDKIRELLRDALAKVFEEADEDMMDEVNACDPIRVAVTVESVLFENWGPSNGAQKVKYRSLMFNLKDQKNPDFRRKVLLGTVEPQRLAVMSSAEMASEQRKQENEKIEQKALFDCERGLQPKATTDQFKCGRCGQRKTTYYQMQTRSADEPMTTYVTCVNCNNRWKFC

>MedtrMED26-6

MTLEDFFTLTEMKDGLTTPSRVQELVSVMKKEQDSIVKNTGDAIRQWAAVASTIAATENKDCLDLFIQLDGPWFIDRWLNDAQKLGGGTNDSVMEESITAMLRAVEKLYQDSEKLISSGMWATVSNLLGHHSSKVQDRARALFDKWKEVGNGDAKSHDMDTGQRNHMIDKNLKEEGQLSSVSGASNDNVHVLRLEGGEKSVLRSSDTQIPDKAANVKKESSDNAHQSSASLNCEELKERSNHLTTVLTSVQESASASESELTSSGICNLPVPKQGSFKDQPDDLQLNDLSMKEEQELNDNGPPEKLGAPINPKPESVSVGASEAQVKPVPAPIVPESSLEHDVKSSEVGICDKVIVSGSMKTPASDKMSVVDGARATDSSNPQLSKASMEEEGNSQVSNHVDDTSNGSDSFKQRKDPTSPNIIDKSSDMELDYGIVDALDVARQVAEEVTQVSDQDDDTSNSSDSFKQSKVSRSANIVNKNSEIELDYGMVDALQVARQVAEEVEREINNSSSEKSSEGGTRQAGSPESVGKNDDLACALPEVSSRQSNSAEACPEERHMSVSDDVVAEPECIPDLESSQLTEAAQDPGGNSEKSLCTFDLNEEYGSDDMNVSANTISTTPIPVVSASKPAQTSGLPTAPLQFEGTLGWKGSAATSAFRPASPRKNADNQKNVSAGGNSDISKQRQDFLDFDLNVAGGEDELVKQIGESSGLPSGQSSVEHSPKRSKRFELDLNSIGDDGDTQPSDQRMEGQLFFGRNGYWSPSPASSSSSMQPSVRNIDLNDRPYFQTDLLDQGPTKSSSSIEVYGLSKSDAPAISILGAKVEVGRKEPVPQIWSLPNGKAVEPAIDLTMMPGSGGVSGMGPAVSYNHSTFLGYNGLTSMPPLSFSPAVYGSGGTIPYMVDSRGAPVVPQVGGSSSNVLSSYAQPPYIMSMAGPQLGLNGVGPSRPNFDLNSGFMIDGGNRDALTARPFFFPGQSRAMEDRTLQQSSSSGVGGKRKEPDGSGWETYPFGYKHQQQPPWK

>MedtrMED30

MEEQSINGTMSTKTTQDLAIEGHKYLEETIQHAFKILSSMNDELCNPVWWSTSPSSATSPNAPSSNGDANSENSGQHADGAAPSGGAGGALDEARLRYKDAVAGLRSVLAAIPNSQKANTFDGGSADSPMDEAEIEKLEEQASSLRKELGNKNLHLKILIDQLRELITDISTWQSPFST

>MedtrMED31

MGKNKFSTTSSLLILREIFKGDLGHIKVPSSNPANYISLNRCVFVLVVEDSVSMASKTESGSPRDTPPSPPKSIYKDPDDGRQRFLLELEFVQCLANPTYIHYLAQNRYFEDEAFIGYLKYLQYWQRPEYIKFIMYPHCLYFLELLQNANFRNAMAHPTNKELTHRQQFYFWKNYRNNRLKHILPRSLAEPSAALPAPASTQPQPPVPALPPVPATSVAVTTSSSQAPSPMPYGIPPGSGIAKNDMRNTSADRRKRK

>MedtrMED36-1

MAPPVRGRGGGGFRGGRGGDRGGRGGGRGGFGGRGGDRGTPFKARGGGRGGGGRGGRGGGRGGGRGGGMKGGSKVIVEPHRHEGIFIAKGKEDALVTKNLVPGEAVYNEKRVTVQNEDGTKTEYRIWNPFRSKLAAAVLGGVDNIWIKPGAKVLYLGAASGTTVSHVSDIVGPTGVVYAVEFSHRSGRDLVNMAKKRTNVIPIIEDARHPAKYRMLVGMVDVIFSDVAQPDQARILGLNASYYLKAGGHFVISIKANCIDSTVPAEAVFSAEVNKLKADQFKPMEQVTLEPFERDHACVVGGYRVPKKKKDAE

>MedtrMED36-2

MAPPRGRGGFGGRGGDRGGRGGGGGRGFGGGRGGDFKPRGGGRGFGGGRGARGGGRGRGGGRGGMKGGSKVVVEPHRHEGIFIAKGKEDALVTKNLVPGEAVYNEKRVSVQNEDGTKVEYRIWNPFRSKLAAAVLGGVDNIWIKPGAKVLYLGAASGTTVSHVSDIVGPTGVVYAVEFSHRSGRDLVNMAKKRTNVIPIIEDARHPAKYRMLVGMVDVIFSDVAQPDQARILGLNASYYLKAGGHFVISIKANCIDSTVPAEAVFSSEVNKLKADQFKPFEQVTLEPFERDHACVVGGYRMPKKKKDAE

>MedtrMED36-3

MLDLTAMASGESKLPLETQRLQIYSKPNSGVSPFWREKYEREAKKYWDVFYKHHKDKFFKDRHYLDKEWGDYFSGGGKKVILEVGCGAGNTIFPVIASYPDAFVYACDFSRRAIELVKMHEDFKESHVHAFVADLTADDLCKEIIPSSVDIVTMIFMLSAVSPEKMPIVLQNIKKVLKPNGYVLLRDYATGDLAQERLSGKDQKITDNFYVRGDGTRAYYFSNEFLTNLFKENGFDVHKLDVCCKEVENPLKGVDNETVNTLYHLRWVQAVFCVSDGSNSSSKETEVNHLDSDNNIGTEIEKNNCGSITDTVIDMSEGVGADMFGVLPSDEYEIMEINLRGWNFKINLLSKEYQHTCKSTGLMLWESARLMASVLVENPNIVSGKRVLELGCGSGGICSMIASRHADRVVATDGDDFSLDLLAKNVASNIEQPLLTKLTTKKLEWGNKDHIESIKELSDRGFNVIIGTDVTYVAEAILPLFATAKELIAPSESNKDENVPVLILCHIFRRVDEPTLLSAAVQFGFRLVDKWPTGNSPETSRSVIDNWFMDNDLKDDLPNSALNILVFSME

>MedtrMED36-4

MRGGSKGVIIPHRHEGVFIFKTSNSDALLTKNLVPGESFHIEIIAHNVFISIMNE

>PhvulMED2

MDSVVDSLNNAYQDFVAAAANVLEAKESAGSVKTTATDTALENFKQKWELFRVACDQAEEFVESVKQRIGSECLVDEATRPVAGKPGQATMTGLPPISAVRLEQMSKAVRWLVIELQHGSGASSANSALSHPSAAFDARFSEDATQ

>PhvulMED3

MQMQIQQTQQQATTAPISTSTPPPSSGGSASEAPPKQVAQAMDKLGQAERIIADIRIGADRLLEALFVAAAQPHQGNKPLQIFLKEDACMRQYLQDLRSLGKELEESGVLSESLRSRKDFWGLHMPLVCPDGAVVAYAWKRQLAGQAGASAVDRTRLALKAFTDQKRRFFPHLDDGLETSDLASKKSCGSEEITVDPKEEIRFLRTLPDVLKSVEKDVPILKILTFERLDWLKRASTLTSLANESSLEHNYHGSNKLRLGSVGTVAEEKVAVIEMLFPSVFRAVISLHPAGSTDPDAVAFFSPYESGSYVHARGFSVHHVFRHITEYAATALQYFLGNQTETSLYCLLHWICSFQTLFSKPCSKCSRLLAMDKQSTLLLPPVHRPYWQFSFSKILLSISSKDQNSDTKTYHIGCLSEEI

>PhvulMED4

MHGCAGDQWKHNRHMWPVPANATTVAIDSSPSQFICKDGRKIRAGDCALFKPPRDSPPFIGIIRKLSYDKQESPSLEVHWLYRPADLKLAKGIVLEAAPNEVFYSFHKDETPAASLLHPCKVAFLRKGVELPSGISAFVCRRVYDIENNCLWWLTDKDYLNEQQEEVNQLLDKTKLEMHGAVQSGGRSPKPLNGPASTQSLKSGSDNIQNSSSFGAQGKGKKRERDQGSDSSKKERLFKIEDGDSGQFRPESMLKSEIAKITDKGGLVDFEGVEKLVQLMQPDSADKKIDLPGRIMLVDVIALTDRYDCLGWFVQLRGLPVLDEWLQEVHKGKIGDGNMPKESDKSVDEFLLALLRALDKLPVNLHALQTCNVGKSVNHLRTHKNAEIQRKARSLVDTWKRRVEAEMNMNDSKSGSNRAVSWPAKPANSESPHVGNRKTGGSSDNVVKSPAIQPSLSKSSQSKLSSGEALSKSSSPGSTKSLTTSVGMNSKDQNSKVFVGAATADLPLTPIKEERSSSSSQSQNNSITCSSEHAKTIGSCREDAKSSTAVSMSASKIHGCASRTRKSSNGIHGPGAVGQKEHNSAKISTRNSPTEKVSPTRASHEKSVDQPLADQGNNQRLILRLPNTGRSPSRGASGGSFEEPATTSSKALSPADLNDNQDRRLKTKTECLLTHVSNMINESCDANEALIGDENKGTPIVDERCRAIEDSDKVLETSKPTSLPSGFVSRSGQTYDASLSPMNALVESCVKFSEASSSVSHGDDGMNLLATVAAGEISRSENASPVASPERKSSPAGDEQCSGNDLKLKHSGEAAVRTLSELNGRATGEHPLNTVDSSQIKNELRHPAMTVSRDFPGDGETISSSHDTRINVSSTDLLQNVEGPCLRPETIEDASVTILTPKKESNADAGVSDSKLKPRASSFDDDQKVDHMKEEIIENEKMLVSKPFTNVESENESGEKKPDLTSSVDNENHISVEKATGTGILVQKTSPTAENSESIYLKKELPASGNALMVPMDENADDMKSVVIEPDERRREQDSSSPDDSNDCAEDNMGRKEAIGQCSGSSSVQPDLQTMSRKENEVSKSCEQKLDANPSEVSGERHAYSASGADATVKLDFDLNEGFPFDDASQGEIARQEDPITSSAVHVPCPLPFPISSISGGFHPSITVASAAKGPVIPPENPLRMKGELGWKGSAATSAFRPAEPRKNAEMQSSTNDITSVEVTSIKQSRAPLDFDLNVADERCFEDVGSHGSLESGPHDRSVGLDLDLNRVDDTPEIGSFSISKLDIPSLPSKPSLSSGLSNGGSVSRDFDLNNGLGLEEVGSEVPARSQLMKNSVPFPSAVHSTRTNNAEYGNYSAWFPPGNSYPAITVPPLLPGRGEQSYVSGAGAQRIMGPTGSSPFGPEIYRGSVLSSSPAVAYPSTTAFPYPGFPFETNFPLSSNSFSGSTAFMDSSNVGGLCFPTMTSQPVGPGGVVSSTYPRPYVMSLPGGTSNVIPDSRKWGSQSLDLNSGPGVADTERRDDRLSSGLRQMSVPNTQASIEDHLKMLQMAGAALKRKEPDGGWDAERFGYKQHSRQ

>PhvulMED6

MATAPGNQMMEGGPPPLPGTDMTGICFRDQLWLNSFPLDRNLVFDYFALSPFYDWTCNNEQLRMRSVHPLDLSQLSKMTGTEYMLSEVMEPHLFVIRKQKRDSPDKVTPMLAYYVLDGSIYQAPQLCNVFAARIGRALYYIQKAFTTAASKLEKIGYAASVDSENETALVESKTAKETIDIKEVKRVDHILASLQRKLPPAPPPPPFPEGYVPPPTAEAEKGTETQEAAETQAPTADPIIDQGPAKRMKF

>PhvulMED7

MATATYPPPPPFYRLYKEYLQDPKSAPEPPPPIEGTYVCFGGNYTTSDVLPSLEEQGVRQLYSKGPNVDFKKELRSLNGELQLHVLELADILIERPSQYARRVEEISTVFKNLHHLLNSLRPHQARATLIHILELQIQHRKQAVEDIKRRREEARRLLNESLATLDGH

>PhvulMED9

MLRMNKGSLDYWRNYFGAANSDIFGIIDHAIMVAASDCPEEFRMRRDGIAERLFSCRLSRCLGCERVELAVSVDDDGGEGGGGCKSGVDGAGAEFESEAGASKESKVNSARDDDPGEMNTNRVSNYSYGEAEALTDEIEEESQYVEEVFRIKDVLLNYEEASDSVLFDSLRRLQLMELTVDLLKATEIGKAVNPLRKHGSSDICRLARTLIDGWKEMVDTWVKATTAFAGSEGTPDSVNPSVVEDEDEEGLPSPPMDEGALFAAPTGSMELSQFFDGMDDDGNPRHSGEFIRNRDHGRSPTLNSKNTAKRKPQASNEANIIAKDSKGQQAKKNEAAVRPNQPVIGDSGPGRPPKSTMQKKSHIEPQVQQNTVKSTIPKNPYVRQLDKPRCSDDASVQVKLEASKRKLQERYQQVENAKRQRTVQVMELHDLPKQSIGHRNPHVKPGNQRKQWGQGRR

>PhvulMED10

MDSSQSTVLGGNGGSGGNGTLISQTNDITASTAGADDSMQKLNQVSNSIQKTLGLIHQLYLTVSTFNAAFQMPLLQRINGLVVELDNMVKLAEKSNIQVPMEVVNLIDDGKNPDEFTKDVINSCIAKNQITKGKTDALKNLRKHLLEELEQNFPDEVETFRESRAAAAAELKRQAQAQSALPNGDVRVKSEH

>PhvulMED11

MASLAMVQGGGVDERCVQKSYWIEHTTELSVESMMLDSNASHLDKEERPEVLSLLPAYEGKSVLELGAGIGRFTGELAKKAGQLLAVDFIESAIKKNENINGHHKNVKFMCADVTSPNLQISEGSVDLIFSNWLLMYLSDKEVENLAGKMMKWLKVGGYVFFRESCFHQSGDSKRKYNPTHYREPRFYTKVFKECHKSDDRGNSFELSLIGCKCIGAYVRNKKNQNQICWIWQKVRSEDDRGFQRFLDSVEYNHKDILLYETVFGQGFVSTGGLETTKEFVAKLGLKAGQKVLDVGCGTGGGDIYMAENFDVEVVGIDLSINMISLAIERVIGLKCSVEFECADCTKKSYPENTFDVIYSRDTLLHIKDKPSLFRSFYKWLKPGGTLLITDYCKSVGSLSVGYANYIQKGGYYIHEMKTYSQMLEFAGFNDVIAMDQSNLFMKTLQQELNDLESKKDDFIDYFSKEDYNKISERWKAKQMRGADGEQIWGLFIAKKK

>PhvulMED12-1

MEAREISLNQVPHFNKNVVLNCKEAIRKRLRAINESRAQKRKVGQVYGVALSGSQLSRSGIFPELRPCGEDFQKKWNEGLSQQHKRLRSLADHVPHGYKRASLLDVVMRNNVPLLRATWFIKVTYLNQVGML

>PhvulMED12-2

MQRYHAGSCTSAVNNSAIGGPSARDTGRSDSPSLPANFSVSSRRQPPLNSYKLKCDKEPLNSRLGAPDFHPQTSNCPEETLTREYLQSGYRDTVEGLEEAREISLTQVPHFNKNVVLNCKEAIRKRLRAINESRAQKRKAGQVYGVALSGSQLSRSGIFPELRPCGEDFQKKWIEGLSQQHKRLRSLADHVPHGYKRASLLDVLIRNNVPLLRATWFIKVTYLNQVQPGSVGISSGTADKIQLSRSDVWTKDVINYLQALLDEFLSKNVSHSASHARERSPQMPGSLQNKSDPLSSVSDGEGPSLHFRWWYIVRLLQWHHAEGLLHPSLAIDWVFNQLQEKDLLEVWQLLLPIIYGFLETIVLSQTYVRTLAGLALRVIRDPAPGGSDLVDNSRRAYTTCAVIEMLRYLILVVPDTFVALDCFPLPSSVISHAMNDGNFVLKSTEAAGKVKNSSDDFGHIISCIQKHTEDLAKASIPGAPGHCLAKVAKALDKALVLGDLRVAYKFLFEDLCGGTVSEGWVAKVSPCLRLSMKWFGTVSTSLIYSVFFLCEWATCDFRDFRGTRPRDIKFTGRKDISQVHVAVRLLKMKIRDVKISLKQTNEYHGASRFAKTNQQPNWNYVGKVSRLKSSSKSTGSSVIFESPGPLHDIIVCWIDQHVVHKGEGSKRIQLFIVELIRAGIFYPLAYVRQLIVSGIMDGNVNLVDMERRRRHYHILKQLPGCFIHDVLEESGIVEGAQLKVALQIYLNERHLILRGPLSESHDDASGSNLSALKRKKYPASMKDEASGMAIDQRNVISITKNTKNNANIEELRTAISVLLQFPNCSSNLSATGCDESEGSVRRPIGSQYSKNDPVEGTPGCEECIRTKRQKLSEERNSFVQGNSPVQSDDDDTWWLKKGMKSPEPLKVDQPQKSTKLVTKSRQKNVRKTQSLAQLAASRIEGSQGASTSHVCGSKVSCPHHKTAMDVDGQRSVDSIRTSHFGDIVSIGKALKQLRFVEKRAIAIWLLTVVRQVIEEMDKNVGKVGQFGRPFSVADDKSSIQWKLGEDELSAILYLMDISHDLVSAVKFLLWLLPRVLNSPNSTIHSVRNVLMLARNVENQVCDVGEAFLLSSLRRYENILVAADLIPEALSSAMRRAATIIASNGRVSGSGALAFARYLLRKYSTVASVIEWEKTFKATCDARLSSELDSCRSVDGELGLPLGVPAGVEDHDDFFRQKISGGRLPSRVGAGMREVVQRNVEEAFHCLFGKDRKLFAAGTLKGLPPVEKWDNGYQIAQQIVMGLIDCIRQTGGAAQEGDPSLVSSAVSAIVGSVGPTLAKMPDFSSGNNHSNITSASNLLNYARCILRMHITCLGLLKEALGERQSRVFDIALATEASTALAGVFTPSKASRAQFQTYPEVHESSNTISNDMGNNSNKVVVAKTTKIAAAVSALFVGAIIHGVTSLERMVTVLRLKEGLDAVQFVRSTRSNSNGNARSVMAFKMDNSIEVHVHWFRLLVGNCRTICEGLVVELLGEPFIMALSRMQRMLPLNLVFPPAYSIFAFVRWRPFILNATVREDMNQIYQSLVVAITEAIKHLPFRDVCFRDCQGLYDLMAADNSDSEFASLLEFNGSDMHLKLTAFVPLRSRLFLNAIIDCKMPQSIYAKDDGSRISGPGESKVQLTDSGSKLQDMLVHVLDTLQPAKFHWQWVVLRLLLNEQALIEKVENHDVPLSDAIKLSSPSPEKAASASENENNFIQILLTRLLVRPDAAPLFSELIHLFGRSVEDSMLLQAKWFLGGQDVLFGRKAIRQKLHNIAVNKKLSVKTQFWEPWGWCSPSTDSSTIKGENKKFDSTSLEEGEVVEEGTDLKRCQQQVIERALIELLLPCIDQSSDEAHNSFATDLVKQLSFIETHITAVTGGSKPVGSAPPGVEGQPNKVNNRKNMRTGSTALARRPTVAADSSPPSPAALRASMSLRLQLLLRFLPILCTDREPSVRSTRQFLASVIFRLLGSRVVHQDAGISANAVPLPMREAESSSEVASVDSSSQSLFDRLLLVLHGLLSSYPPSWLRPKPSKTSNEPTIDREWLETLQNDLDRMQLPDTVRWRIQAAMPILIPSMRCSLSCQPPSVSNSALMCIQPSTTNPGVNSSSSTIPQRNPALSRVASNASGKPKRQDNDLEIDPWTLLEDGAGSFPLPGNTASIGSGDHVNIRAASWLKGAVRVRRTDLTYVGAVDDDS

>PhvulMED12-3

MTLEDFFTLTEMKDGLTAPSRVQELVSVMQKEKSCEVKNSADAIRQWAAVASTIAATENKDCLDQFIQLDGLCFINRWLKDTQKFEVDAKDSFVEESITTLLRAVEKLHLDREKSMSSGIRITVSNLLGHHSTKVQDRARTLFDSWKGAENADTESHDVELAKADNASNEIVRDEGQPSAVNEAGNDNDNASELNGTVNSLLKSSDNLPVHSSANVCHSSSSLECDDVKEGSVNHVDGVPSSAQVELPLCPADETTSVATSNFSLHNQGSFEGQSDMVQLIDLAKREKQEQNVNDPPEKFGAPEICSVSSEPELESVSIVCSEAKAPESVKGPALERNVEHNEEDVCHNLPISTCIRTPSSDRRTGEDDVRTVTSFTQVFRAAENDKDCSNALQDTSVSDSNLGKTEVPDMSVCGAGSVTPSKEGKGHIYNNKDVTSIGSDSSKPEIDFRRSNIVDNRGSGNELDCGIVDPLEFARQVAQEVNREVSSSSEKISYGRIRQPCSPDSVRKEDVLTPVPPEEVSSRHSLATDNTEAEPECRPDVVCLEVTEVVQDSEGNSEKRPCGFDLNEVGFDDMDVCLNTTSTPIPVVSASRPTPTPGLPGAPLQFEGTLGWKGSAATSAFRPASPRKYCDSERNLSVDMNSDTSRQRQRQDWLDFDLNVAEGEEGNAEPVAESSGGLSGQSTVEFSSKRSSMLGFDLNSTGDDVHIQPSDHRMDGQLFLGRNGYWSPSPTSSSSSMQPYVRNIDLNDRPCLQTDLVDLGHGKSSHIINGYDCSKSLDAPVISLLGAKVEVGKKERVPQSFFPNGKAVEPAIDLTMPRAGGIIGMAPAVSFNPSSGFGYNGVPSASAAPTMPFPSAMYGSGGTIPYMVDSRGSPAVPQVGGPSLNILPSSYSQPPPFFMNMTGTQLGLNGFGPVRPNFDLNSGFMTEGGNRDTLAARQFFFPGQGRAVEEQVRTMPQPSSSGVGGKRKEPDSGWEPYPYSYKHSQPPWK

>PhvulMED13

MWTNVFKIGSLHQISWFQFLPHEPDLNPLPDKSVKVDQKDVANLLVLSSHLQLQKEGFLGTWTNSFVGPWDPSQGLHNPDEKIKLWLFLPGRHSSVVESAQPAVSRLRVVASGLWLAPGDSEEVAAALSQALRNRIERALLGLYYMRFGDVFSKFHQFQSEELFRRGQPAVEFAFAATEEAIFIHVIVSSKHIRMLTTSDLEKVLKHSTESACRLPVIVSPHGIRGSLTGCSPSDLVKQSYFSSTKFRVSNGIIGLPYNVSQGVGCQLRGQNCYVEVSLGFPRSGTDNSLQPNRTSARNLPTLHVAESPITGRSDHKGSADHLSDYEKTFLYPAEAVLVPVLQTSLARSSLRRFWLQNWMGPSLPGSSSLIHCAGNVDCCEDPWTEINGARTQNSYDSSSNSNSSSISSLSASSSDSDYKTTGPSELEADADSLTCRQSMVSSADQLESDGPKLGSKRSRTGVTESLSTAANIPVQDTYMSDFGSVEVNNSAITGVGNEPIGSYWDWDDDDRGMEMDIQALLSEFGDFGDFFENDVLPFGEPPGTAESQALMFSSPDYGDVNSSPGGVIDVPDQILLPVGFPSFESFNPPPSTSVEECLNKSQDNLNNSMSLGPTNQTQMLYTREFDHIMKAEAMMTFAPEFGAVETPTYELSTTLFRSPYFPKCRKAESSNSSSNNYLYGAAPPSSPCTEGSEGKNGMSFNTKTGSGKHDSGTSLHSKHYYTFVESRKEKNDKNPVVCNDNSIAKSDGILSLSNIGSNAIVKSSLRKTAEGTHEPEHALLSAKTLLATDITCVMLQASVCRLRHVLLSSGNLMPVGFSRSTGVSFFNQLPSEPSTTTDNISGKYDVKKKENIPIRIAGDIDGGMLDGHLNAPVGVWRTLGASKVVKPSNSPNMEVGPSFSHNSFNEEGILSYGQRKPLQELLDGIALLVQQAISFVDLALDADCGDGPYGLLAMQEQWRRGFCCGPSMVHAGCGGTLASSHSLDIAGLDLVDPLSADVHASTVISLLQSDIKTALKSAFSNLEGPLSITDWCKGCNPLVDTGSIVEGVSVESTNNECRDSSEPMSPSQSSVGGSSSIKVSNIMDGAKGDETSQRRSGHDLCNTESEQQTCARLKPTLIALPFPSILVGYQDDWLKTSANSLQHWEKAPLEPYALQKPITYHVVCPDIDPLTSAAADFFQQLGTVYETCKLGTHSPQGLGNQMEIESSKLSSCGFVLLDCPQSMKIESSNASLVGSVSDYFLSLSNGWDMTSYLKSLSKALRGLKIGSCFSTNPSEGSNSSCLVIYVVCPFPDPTAILQTVIESSVAIGSVVQQLDRERKSSLHSQVVKALSGLATVDEASPSNILVLSGFSIPKLVLQIVTVDAIFRVTSPSVSELVILKETAFTVYCKARRISRGISSDFAQSAFSGRSHSVLTQLPSPISGMWKDCVGPRMAGHSLPREGDIDASLRPGTWDNSWQPTRTGGLNCDPSRTGDIFLHDEIRYMFEPLFILAEPGSLDNGISVIGSPTSESSKALVDDSSGNYVQSTSTTGSVESASSADGSGSDLKTPPSLHCCYGWTEDWRWLVCIWTDSRGELLDCNIFPFGGISSRQDTKGLQCLFVQILQQGCLIIQSCDPGLAKPRDFVIARIGGFYELEYLEWQKAIYSVGVSEMKRWPLQLRKSMSDGLSATSNGSSLQQPDMSLIPERTLPSSPSPLYSPHTKSTGFMKGNLGQPAARKQLMGGHSMVDNSRCLLHWAQSISFVAVSMDHTLQLVLPADSSTPSYIEGFTPVKSLGSTSSAYILIPSPSMRFLPPTVLQLPTCLTAESPPLAHLLHSKGSALPLSTGFVVSKAVPSMRKDYRSNLKEEWPSILSVSLIDYYGGTNIPQEKVVRGINKQVGRSLSWEAKDFEIETHLVLESIAAELHALSWMTVSPTYLERRTALPFHCDMVLRLRRLLHFADKELSKHSDKS

>PhvulMED14

MDIISQLQEQVNLIAHLAFNTIGTLQRDAPPNRLSPNYPEPPAHPTEEGTNFSEQPKLMSSTLVKAAKQFDALVAALPISESGEEAQLKRIRELQAENDAIGQELQKQLEAAEKELNQVQELFSQASDNCLNLKKPDDN

>PhvulMED17

MLQHQIVQSPARLGLANPNSPSIPNPTPPKLPPTQTQQPQDRNSSTPSSALLSLLPPLTRAQALLHQMASLASKLFEVSPNRSLWVTAFRGSPTFLSSQSSTPLDSSPSTAKEIISLFTVLQTQIFEAVAELQEILDLQDAKQKMDREIRSQDSMLLAFANKLKEAECCLDILVDDYSDYRRSKRSKSGDDDSMTSSTVSSQLKLSDILSYAHKISYTTFAPPEFGAGQAPLRGAMPPAPQEEQMRASQLYNFADLDVGLPKEVETKEKIVEAIVEPPSQVDTNAVPNLSAFQGLLPPVPPGWKPGMPVQLPIDLPLPPPGWKPGDPVPLPPMDSLRFAEQQMQPHIPQPKQPEVIQVQPVNLDLGGSDTSDYSSDDVSSDDED

>PhvulMED18

MDDNMDLQISLDKLPIKRLDSIEENGMERFPPDVDYDEKRLSLIRRIDFAWAIEKDEEKKKQKKSSKETSTPWQWQSMVENLQLAHQELSVIIDLINTVEANDAVTVASMTRPKLQPNEALSDLAVSAASKLQCYRQVGKYFKQSAKAFEQQVAREARFYGALIRLQQNWKVKRQRQAAIVPGNEGFTFDLFDNSYDQASIIRSLSMSTVRVNHDAAGMLAINMSPDLCRSLQFGFVGAQSDDIPRNSKGNKSYFSVEHSLGETGKESLTDEECVKKTHALLREVHEAIFNEHVFDLVNREAFTTVTGVSVTGIRENYLELSLGRGTSVYLSLVSNGQDHSTVENELTNNAENAMLPPESSDEMMLEAKQNAPKKGLFSNSICYEIYIQQIFHEHIFGKGGEKPISSGNRLSGVQAKETKDGGSNLLGHFFMSLSHRIFSTKVLAELENVVSKVPYLQLISNPTWHSRASSWTLYMEVPQSILRGSQIKTSDYFEKNAVKRQFWIKAVVNDNCINVKAEGSPNVAGLFKGKIEETHSINKYNCNLADLPVIILQQVASQIINWLYQEALMVGIKANRDFLCLSFELEQGETLGLVASVDPEDSEGCISWWLVMEDSFAEEQKLHMNITDGASEYRKFLGHLSLDLLYATLIDLVGLCSGGTSQ

>PhvulMED20

MPIRCILHWQPNQGTMVNSQILNEISQCVESLNGVKEGRCKASLTFYRPNLRDPSTAIDFPRDFLGISMLEQPNKYYFIIRGHKLVVEADYSILTIMEKLQSYKSKVALHFEGALYKLGDFQVRVIKVVPNQAESLRGIMIEIEYLPISSVEKSKPIMEDFIDLWKEVVSKKSLAGQFIHTEPNYAEYGLSDNYTSQHTAVQYAAALAQLIQSSQLRN

>PhvulMED21

MASKNESDNSTDTSPSSPKNIYKDPDDGRQRFLLELEFVQCLANPTYIHYLAQNRYFEDEAFIGYLKYLQYWQRPEYIKFIMYPHCLYFLELLQNANFRNAMAHPTNKELAHRQQFYFWKNYRNNRLKHILPRSLPEPSATSAVPAPVSTTTQAPVSALPPVPATSVAVTSTPAQAPSPMPYGMPPGSGLAKNDMRNPTVDNRRKRK

>PhvulMED22

MAAELGQQTVELSTLVTRAAHDSYASLKELVDKCRSSELSDTDKKISILKFLSKTQQRMIRLNVLSKWCQQVPLIQHCQQLASTVSNHDMCFTQAADSLFFMHEGLQQARAPVYDVPSAIDILLTGSYQRLPKCVEDVGTQYALTEDQQKPALKKLDTLVRSKLLQVSIPKEFSDIKVSDGTAMLRVVGEFKVLITLGYRGHLSLWRILHLELLVGEKNKTVKLEEMRRHVLGDDLERRMAAAENPFSVLYSVLHELCVALVMDTVIRQVQVLRQGRWKDAIRFELISEGHGASSSSAQNPDGESESSALRTPGLKIVYWLDFDKSANVSESGTCPFIKIEPGSDLQIKCLHSSFVIDPLTGKEAEFVLDQSCIDVERLLLRAICCNKYTRLLEIKRELVKNVQVCRTVDDVVLQSRMGEPDIEYKQKDDKCCSKDSEGHEVLCVRAYGSSFFTLGINIRNGRFLLQSSQNIVVSSALIECEEALNQGSMTAAEVFISLRSKSILHLFASIGRVLGLEVYEHGFNIVKIPKDASNGSAMLVMGFPDCGSSYFLLMQLDKDFKPLFKLLETQPDPSGTDNLSGGDLNQVLRIKKIDIGQMQVHEDEMNLSLVDWGKLRSVLPNAIGPNQTSAHEFFSDIRLENSVQIARGHPSGFSSLVDEVFGLEKGSSVAPLSVQNVPSSGNTSLPSQYGSVPMNIHSLKAGSPSPKWEGGMQMAQVNNVTKASGATSLYSGSLFSSGSVKGPVQSSSVGSIPTGHVRNTAGKKLSASKSEQDLASPKSPHSVDISSSIAIDEEQLRVLNDTSNEALSGSRSSRLLSPPRPTGSRMSIPNSRPNGPQADSFKVIGSASCATTPVSQTLESTVSYIAGEDVTSKNDKKSRKRTASDMLTLIPSLQGVENNPGICKRRKISDSSGCQLSLPQGAMSAEMIPKTEGYSYGSLIAEVNKGTVPSSIYIASLLHVVRHCSLCIKHARLTSQMDALDISYVEEVGLRSGSSNIWFRLPLARGDSWQHICLRLGRPGCMYWDVKINDQHFRDLWELQKGSNNTPWGSGVRIANTSDIDSHIHYDPDGVVLSYQSVEVDSIKKLVADIQRLANARTFALGMRKLLGVRAEEKSDELVTSTDSKIPSTKVASDTADKLSEQMRRAFRIEAVGLMSLWFSFGSSVLARFVVEWESGKEGCTMHVSPDQLWPHTKFLEDFINGAEVSSLLDCIRLTAGPLHALAAATRPARAGPVPGVAAALSSIPKQSGGYISSQGLLLGNSTTNVGQPASGPGANTVMPTASGPTNQTLSMLAAAGRGGPGIVPSSLLPIDVSVVLRGPYWIRIIYRKQFSVDMRCFAGDQVWLQPATPPKEGRLSGGSLPCPQFRPFIMEHVAQELNGLDPSFTGQQAGGLTNSNNPNPGSGSQMMAANGNRINLPISAAMSRTGNQVASLNRVGNALAGSSNLALMTSPVSLRRPPGAVVPAHVRGELNTAIIGLGDDGGYGGGWVPLVALKKVLRGILKYLGVLWLFAQLPDLLKEILGSILKENEGALLNLDPEQPALRFFVGGYVFAITVHRVQLLLQVLSVKRFHQQQQQQQQNSNPAPEELSPSEISEICDYFSRRVASEPYDASRVASFITMLTLPVSVLREFLKLIAWKKGLSQTQVGDVVSAQKPRIELCLENHSGLNVDENSESSSAFRSNIHYDRVHNSVDFALTVVLDSSHVPHVNAAGGAAWLPYCVSVRLRYSFGESSNVSFVAMNGSHGGRACWLRVDDWEKCKQRVARAVEVNGSSAADVSQGRLKLVADSVQRNLHMCIQGLRDGNGVTTSSGAT

>PhvulMED23

MDQSQNPRAATSTTPSRSFQFHPARGPILDLFNLYLGLGRNSRNKPEDSLRDPPNKTQKRVHALNRELPPPNEQFILDFEQLQSQFPDQDQLRSVTEAILISLVVQCSGHGPRADFLLFVLRSLCGIGCINWDSLLQSLLSSVSSAELPVGQLNQAVPTVSSSSLSQTGMLPPPSTIANSSNFQSSNPASPLTAVHTIGSPAQSTIESLSCAAMSPVKSSDISSAGQQSKLRGSSAIRNNDISNSSLRQLCCKIILIGLEFSLKPVTYAEIFNHMLNWLVNWDQRQQGMDESDVIKSWRPDKAVIAWLHSCLDVIWLLVDEGKCRVPFYELLRSDLQFIENIPDDEALFTLILEIHRRRDMMAMHMQMLDQHLHCPTFGTHRILSQTTHVSGETHMRLSPITYSSVLGEPLHGEDIASSIQKGSLDWERAVRCIRHALRTTPSPDWWRRVLVLAPCYRPSSQMPTAGAVFSSEMICEATINRIVELLKMTNSEINCWQDWLVFSDIFYFLIKSGCIDFVDFVDKLVSRLSEGDHHILKTNHVTWLLAQIIRIEQVMNALNSDPRKVETTRKILSFHREDRSADPNNSQSILLDFVSSCQNLRIWSLNSSTRDYLNNEQLQKGKQIDEWWRQASKGDRMVDYMNMDERSIGMFWVVTYTMAQPACETVMNWLNSAGVADLLPGTNLQPAERLMATREVSPLPMSLLSGFSINLCVKLSYQMEDSLFSGQVIPSIAMVETYTRLLLLAPHSLFRSHFNHLVQRNPSLLSKPGVTLLVLEILNYRLLPLYRYQGKSKALMYDVTKIISAIKGKRGDHRVFRLAENLCLNLIFSLRDFFLVKREGKGPTDFTETLNRVTVITLAILIKTRGIADAEHLLYLQNMLEQIMATSHHTWSEKTLHHFPSVLREALSGRIDKRSLDIQTWQQAETTVIHQCNQLLSPSADPSYVMTYLGHSFPQHRQYLCAGALILMHGHAENINSGNLGRVLREFSPEEVTSNIYTMVDVLLHHMQIELQQGHSLQDLMLKASASLAFFVWTNELLPLDILLLALIDRDDDTHALRIVISLLDRQELQQRVKLFCMTRGHPEHWLYSGIFKRVELQKALGNHLAWKDRYPVFFDDIAARLLPVIPLIIYRLIENDAMDTAERVLAMYTPLLAYYPLRFTFVRDILAYFYGHLPGKLIVRILNVLDVSKIPFLESFPLQISLTNPVMCPPLDYFTTLLLGIVNNVIPPLHNNSKSGSMGEASNNAQRTTQSKPAVVSQSGPANASEGQKAFYQIQDPGTYTQLVLETAVIEILSLPVSAAQIVQSLVQIVVNIQPTLIQSSNALHGGSNSVGQGSVLPTSPSGGSTDSLGASRSTPSVSGINTSNFASRSGYTCQQLSCLLIQACGLLLAQLPSDFHSQLYLETTRIIKENWWLKDGTRSLGEIDSAVGYALLDPTWAAQDNTSTAIGNVVALLHSFFSNLPQEWLEGTNVIIKQLRPVTSVALLRIAFRIMGPLLPKLANAHALFNKTLSSLLSILVDVFGKNSQTTIAVDASDIADIIDFLHHIVHYEGQGGPVQAISKPRADVLALIGRASENLRPDIQHLLSHLNPDVNSSVYAASHPKLVQNPT

>PhvulMED26-1

MGYEDNPYRDEDGEPLMDYDDVQSDREPSPEPRHQLDDYEDDVNDEWRGRDRSQTPVYDNDAARSKPRKRLIKKSVAGKQSVAAELEDEEEEGYVPEARFDEEDGRKRKKGKEVGSGKKEKRLKGEQRFGSGDGSKFGGSKKGFGGKAGKDHDGEVKEMWDTIAGGDSEDDHDGVRDLDDDNFIDDTGVEPAYYGSDEPRSPGDAPQAEEGEEDSEMKDLFKIGKKRKKNERSPAEIALLVENVMAELEVTAEEDADLNRQGKPAINKLKKLTLLTEVLSKKQLQLEFLDHGVLTLLKNWLEPLPDGSLPNINIRMEILRILNEFPIDLEQYDRREQLKKSGLGKVIMFLSKSDEEISVNRKLAKELVDKWSRPIFNKSTRFEDMRNVEDDRVTYRRPSVKKPTNKVAGISRDSDLDLEISQPTTAGQSSSRQHASRPEATPLDFVIRPQSKIDPDEIRARAKQAAQDQQRMKMNKKLQQLRAPKKRQLQATKLSVEGRGMVKYL

>PhvulMED26-2

MHGRGCEKGTRHMWKAPTRGDSSLNADVSSSSSSSSSTVKLFFKDRRKISVGECALFKVSEDCPPFIGIIRYLTIGKDKKLKFGVSWLYRSIEVKLSKGVPLEAAPNEIFYTFHKDEIDAEALLHPCKVAFLPKGAELQPGISSFLCRRVYDIANKCLWWLNDQDYINDCQEEVDKLLYRNCVEMHATVQPGGRSPKPMSSPTSTSQLKSASDSVQNSTSSFPSHIKGRKRERADQGSESVKRERSIKAEDGDSGNFRHDNIFKTEIAKITEKGGLVDGEGVEKLVQLMVPDRNEKKIDIASRSLLAAVIAATDKLDCLSQFVQLRGLPVFDEWLQEVHKGKIGDGDKSAEEFLLVLLRALDKLPVNLQALQTCNIGKSVNHLRTHKNTEIQRKARGLVDTWKKRVEAEMNINDAKSGSGPNVHWPAKSRPSDVGQGGNRHSGASSDVGMKSSVTQLSASKTASVKIVQGENITRSALTSAFPGPAKSAPSPAAVTANLKDGQPRIVAVNGGSDLPMANARDEKSCSSSQSHNNSQSCSSDHAKTGGHSVKEDARSSTAMSVNKISGGSSRHRKSINGFSGSTPSGGQRETGSSRNSSLHKNLTSEKISPPGLMDKAVDGTSLDGNIPKLIVKIPNQGRSPAQSVSAGSFDDPTIMNSRASSPVLPDKHDQSDHSPKEKSDLYRVNIGSDINTESWQSNDFKDVLTGSDEGDGSPAAVTDEEHCRTGNDCKKALEVSKAASSSSGNEHKAGNMQDASYSSINALIEGVKYSEADDGGMNLLASVAAGEILKSELLTPAGSPERNTTAVEQSCTDNGVNKSSEENLVRDECHSNNGLDGEHKNLASVTGDLGANDESDSDFQASGGKAARELNKRVNACSMDLQQVTETTLESKGKLNEKSGPTSLGGLAENSVQEAGDADRSKQLQEVVQGVNAGETHDKVSCVAEVEAEAAKKLLHTAVEVDAQSDNCTAEGSSGCGQLVKKPPAILVQSDLASGKDDNALHSSGYSVDEVPKDFTDRESEKTDDVDAENHVSQSKNKRNESESDALTMPENKGLCSVVTGLVAEHVEENLEAKEVRDQPAREDPPEDSPSVRSQEIDKHLDSKRLKLTSTETEEAEECTSTTADASSMSAAAVSDVDAKVGFDLNEGLNADDGRCEFNSIVTSGCAPAGQLISPVPFPASSMSGILAPVTVASAAKGHFVPPEDLLRSKGEIGWKGSAATSAFRPAEPRKVMEMPLGTSATPIADAPAGKQSRAPLNIDLNVADERILDDISCARHTNSISLATDCHDPVCSKIPSPVRSSGGLGLDLNQADDASDIDICLSSNHKIDVPTMQGKSSLGGPPNREANVHRDFDLNNGPSVDEVTTESSFFSQYARSSVPSQLPVSGLRVTTAEPGNFSWLPSSGNTYSAVTISSIMPDRGDQPFSVVTPNGPQRLLTPAAGGNPFGPDIYRAPVLSSSPAVSYPSAPFEYPVFPFNSSFPLPSASFSAGSTAYVYPTSANRLCFPAVNSQLMGPAGTVSSHYPRPYVVGLTEGSNSGSAETSRKWTRQGLDLNAGPGCSDMEGRDESSPLPSRQLSVASSQALAEEQARIQLAGSVRKRKEPDGGWDGYNQSSWQ

>PhvulMED26-3

MGYEDNPYRDEDGEPLMDFDDVQSDREPSPQPRHQLDDYEDDVNDEWRGRDRSQTPVYDNDAARSKPRKRLIKKSDAGKQSVAPELEDEEEEGYVPEARFDEEDGRKRKKGKEVGSGKKEKRLKGEPRFGSGGGSKFGGSKKGFGGKAGKDHDGEVKEMWDTIAGGDSEDDHEGVRNLDDDNFIDDTGVEPAYYGSDEPRSPGDAPQAEEGEEDAEMKDLFKIGKKRKKNERSPAEIALLVENVMAELEVTAEEDADLNRQGKPAINKLKKLTLLTEVLSKKQLQLEFLDHGVLTLLKNWLEPLPDGSLPNINIRTEILRILNDFPIDLEQYDRREQLKKSGLGKVIMFLSKSDEEISVNRKLAKELVDKWSRPIFNKSTRFEDMRNVEDDRVPFRRPSVKKPTNKAAGISRDSDLDLEISQPRSGESSSRQHASRPEATPLDFVIRPQSKIDPDEIRARAKQAAQDQQRMKMNKKLQQLRAPKKRQLQATKLSVEGRGMVKYL

>PhvulMED26-4

MAPPRGGFSGRGGSGGGFRGGRGDRGRGRGGGGRGGDRGTPFKARGGGRGGGGRGGGRGGGRGGGRGGMKGGSKVVVQPHRHEGIFIAKGKEDALVTKNLVPGEAVYNEKRITVQNEDGSKDEYRIWNPFRSKLAAAILGGVDNIWIKPGARVLYLGAASGTTVSHVSDVVGPTGVVYAVEFSHRSGRDLVNMAKKRTNVIPIIEDARHPAKYRMLVGMVDVIFSDVAQPDQARILGLNASYYLKAGGHFVISIKANCIDSTVPAEAVFESEVNKLKADQFKPFEQVTLEPFERDHACVVGGYRMPKKKKDVA

>PhvulMED26-5

MDSKDFRSILESAGIDIWMLMDVAIAVASVDHSDELRRRRDGIVERLYATSSVSSPPCPNCDVDDVREIEMKSNVNPSVEEEKETCEVLFDDEQKKILEIKEQLEDPHQSIDSLLELLQNLADMDITFQALEETDIGRHVNRLRKHSSNDVKKLVKLLVRKWKEIIDEWMKLKTPGEATTTVVADEDSPQEKILQNGHRQIPDFADSPNALNESFGSEHNNIEPQQRKPKAIPRKESPPQPSASRQRESNFELDKLASARKRLIENYKEVANAKKQRTIQVMDLHELPKPKNAFFGKNKGGTSQRKHW

>PhvulMED26-6

MDSQGQTTSLQRLQNVEKRIVKVLELAGGVMDELASPVGPRKDVVQNHCLEFMQLIKDIQVALRDEIKSACEYRPFEKCDYGSRIANEICHKKVEFIMSQLDAMKETIDEYHSAV

>PhvulMED26-7

MRSGIELSISCSSFGSARMKSWSVDEWREYFVSSKSDIFEIIENAIIVAASDCPKEFRVRRDTIAERLFCSSLTRCAGCDRVQLAARGVGKESDDGGCKNAVERDGGEFAGASKESKVNERGDEDAEIDVNGVSNYSFGEAEALTDEMDEETQYVGEILRIKGVLLNRDEESESVLFESLRRLQLMELTVDCLKATEIGKAVNPLRKRGSKDIRQLAKTLIDGWKQMVDEWVKATATTAIAASDEGTPDSVNPSVVDDEEEEGLPSPPMDEGAFFVAPTGSIELSQFFDGMDDDGNPRPSGPSHKNRDSNSRKPAIPNREDKSQQAKRNDADVPVRPNKPVTSNSGPGRPLQYNMERKSNVEPKIQQKVENNSVTRRPPIGHLDKPMHSDDAKLEATKRRLQESYQQAKNAKRQRTIQVMEINDLPKHVNYRNPNSKPGYHNRNRAITRR

>PhvulMED26-8

MECVVQGIIETQHVEALEILLQGLCGVQRERLRIHEICLKSGPHLGSVTSEVRLLCDLEQAEPSWTVRHVGGAMRGAGAEQISVLVRSMVESKTSKNVLRLFYTLGYKLDHELLRVGFSFNFYRVAQITVTVSSINKMLKLHATDEAVPVTPGIQMVEVTAPAAAETYTEVAAAVSSFCEYLAPLLHLSKPGISTGVVPTAAAAAASLMSDGGGTTL

>PhvulMED26-9

MQRYHAGSCTSAVNNTAIGGQSTRDIGRTDSSSLPANFPLSSRRQPLLTPYKLKCDKEPLNSRLGPPDYHPQTPNCPEETLTREYLQSGYRDTVEGLEESREISLTQVPNFNKAIVLNCKEAIKKRLRAINESRVQKRKAGQVYGVALSGSQLAKPGVFPEQRPCSEDIRKKWIEGLSQQHKRLRSLADLVPHVRRKSLLEVLIRNNVPLLRATWFIKVNYLNLVRPGSASIPSGTGDKTQLTCSELWTKDVIEYLQTLLDEFFSKNTSHFTPHNRDRSPQVPYTASHQHRSDQLSVSDGEEPSLHFRWWYIVRLLQWHHAEGLLIPSLIIDWVLRQLQEKQLLEIWQLLLPIVYGFLEIVVLSQTYVRTLAGVALRVIRDPAPGGSDLVENSRRAYTTSALIEMLRFLILGAPETFVALDCFPLPSSILSYTINDGNFILKATEAAGKIKNSSEDVVCLFKSKGFDAQYQSLAFDHVISCIQERVQDLTKAVKPGYPGQYLAKAAQALDKSLVLGDLHGAYTFLFEDLCDETVSEGWVVKVSHCLRLSLKWFRTVNTSLIYSVFFLCEWATCDFRDFRTAPCDVKFTGRKDLSQVHIAIRLLKMKLRDMEVSPRQKSGNTRGRGVSYLGKCSGQQSNRNIVKNVSKTKSSSRSMDQNICSSAIFESPGPLHDIIVCWIDQHMVHKGGGLKRLHLLVVELIRAGIFYPLAYVRQLIVSGIMDMNVIDLEKQKRHCRILKQLPEKFVRDALVESGVNAGPQLTEALQIYLNERRLILRCSLWENHGNASNVNISSLKQNQCISSTKDRASTVSTDQWKSVLSSKTASKNGKDDNGVEDLKTFISALLQLPKSLSNLSSTGTDESQGNVRKPIGSQSKIDLVETTPGCEECRKSKRQKLSAERSLFVQAPSPVLSDDEDTWWAKKGLKSSEPLKVDQPLKPIKQVTKTRQKTVRKTQSLAQLAASRIEGSQGASTSHMCDNKVSCPHHRTAMNGDTARCVDGIQSIECEDIVSIGKALKQLRFVERKEITLWLLTVIRQLIEESEKVVGKVSQFGRPFATVDDKSSIRWKLGEDELSALLYLMDVSDDLVSAVKFLLWLLPKVYSSPSTTIHSGRSVLMLPRNVENQACDVSEAYLLSSLRRYENILAAADLIPEALSSIMHRAAAIMASNGRVSGSGALAFGRHLLKKYGNVVSVSEWEKNFRSTCDKRLASEIESGRSVDGELGLPLGVPAGVEDPDDFFRQKISGGRLPSRVGSGMRDVVQRNVEEAFHYLFGKDRKLFAAGTPRGPAFEKWDNGYQIAQQIVVGLIDCIRQTGGAAQEGDPSLVTSAVSAIVGSVGPTLAKLPDFSAGSNHSTMSLATSSLNYAKCILRMHITCLCLLKEALGERQSRVFEIALAMEASTALAGVFAPSKASRAQFQMSPETHDTGTIPGDVSNNSSKIVVARTTKISAAVSALVVGAIISGVMSLERMVTILRLKEGLDVVQFVRSSRSNSNGSVRTVGAFKVDSSVEVHVHWFRLLVGNCRTICEGLVVDLLDEPSIVALSRMQRMLSLSLVFPPAYSIFSFVMWRPFVMNANVAVREDMNQLYQSLTMAISDALKHLPFRDVCLRDCQGLYDLMAGNTTDAEFATLLELNGSDIHSKSVAFIPLRARHFLNAMIDCKMPQSVYTKDEGSRNSGHGESKIDFTDSESTLQDKLVNVLDALQPAKFHWQWVELRLLLNEQALIEKMKMHDISLADAIQLSSPSLEKSGASENENNFIEIILTRLLVRPDAAPLFSEVVHLFGKSLEDSMLLQAKWFLAGQDVLFGRKTIRQRLINIAESKRFSVKTQFSEPWGWCSPCKVPVTLKGNKKKVDSMPLEEGEVVEEGMDVKRSIKGFYPMFESESSTSKQQHGTERALLELILPCIDQSSDESRNSFASDLIKQLNYIEQQIAVVTRGPTKPVNTPVTEGQTNKVNSRKTIRSGSPGLARRPTPAPDSSPLSPAALRASISLRVQLLMRFLPIICTDGESSVRSMRYTLASVLLRLLGSRVVHEDAMVNAMQYSPLRKEAESPAEAAFVDSSVECLFDRLLLILHGLLSSSLPSWLRSKHVTKTANEPAREFSGFDREPLEALQNHLDNMQLPDTIRWRIQAAMPVLPPSIRCTFSCQLPTVPTSALASLQPNTTNSWFNSSSSTVPQRNLVPSSRTTSSGKSKQQDNDLDIDPWMLLEDGAGSCPSANNTNIIGSGDRVNIRAASWLKGAVRVRRTDLTYVGAVDEDS

>PhvulMED26-10

MDLDDFRSILDTSGVDVWMFIDAAIAVASADCAAELKRRRDSIVESLYSATAAPPRCRNCDDGHLLRTNGHQITKQNSPSPSPVRQPHRRRAAEAANSPATPQSLENDDGGEDLDPYGGLFDDEQKKILEIKEQLEEPDQSEDSLVELLQSLADMDITFQALKETDIGRHVNRLRKHPSNDVRRLVKLLVRKWKEIVDEWVKLKPQGGRDTLMADGDSPVQKTTQNGHHHQIPDFAYSPNPHNGSSGSDRNNSEAEHKPKVIPRSEPRPKPTPAPSISTPASASQNRQRDSSFDAERLASARRRLQENYKEAENAKRQRTIQVMDINELPKSKPKNAFFGKNKGGGGSQGRHW

>PhvulMED26-11

MEKDLVELYDAAKKAADAVLSGDAEHEESRCIDALEQLKKFPVNYKILVNTQVGKHLKVLTKHPRQTIRAFAVDLIEIWKGIIIKETSKNKNGGIDSKVESANGERSKSGKMQKSPSVKIEKGETVKAERHDRNGTSKSGAESMKKVQNDVKNEKTDRAASVKVEKSAKEVKPVSGAKKISSSSATPPKLTTMIKSNDATRDKIRELLHEALAKVSGEADEDLVAIVNDTDPIRVAVTVESVLFEKWGPSNGAQKVKYRSLMFNLKDSNNPDFRRKVLLGVVEPEQLINMSSAEMASEQRKQENQKITEKALFECERGGQPKATTDQFKCGRCGQRKCTYYQMQTRSADEPMTTYVTCCVCNNRWKFC

>PhvulMED26-12

MTLEDFFTLTEMKDGLTAPSRVQELVSVMKKEQDRVVKNAGDATRQWAAVASTIAATENKDCLDLFVQSDGLWFINRWLKDAQNLGADNTNDGFIEESITAMLRAVEKLYLDSEKSISSGISVTVNNLLGHHSSKVQDRARALFEHWKGGGLGDADNSDLDRLNNESDKIVREEGQPSCVNEAGNDNHLPSQLAGDEKSLLGGSNSQLQEKVSSIQIQSIDNALQSSVSLDCEDIKEKSSHVASAQESTLREGETTLAGTCSLPITKQGSFKGQQDDLQLSDLSKKEKQDQDVNGPPEELRASDISSESAKPDPEPASTGDSEAKALESVKEEPALEHNVESNEKVVFPKISVSGSMRTPESDGMSEMDDFRATSSSNPQLPKASETDDDSCSKMLPDLSVTGSNLEKPEMSFIKSEYIIAVKEIKDQESDQDDDTSNGSDSFNQGKGHRSPNINDKSSDMEIEYGNVDALEVARLVAQEVERECVSPVKEGNDHGSNRTTNGSDSFKWRNGPKSPNVIGKSCEIELEYGMVDALEVARQVAQEVEREVSSSSEKISKGGIRQRGSLDSVGRKDEVTRILPEEVSSRQSNSAEVCSEEVGHMTVSDNVEAGPDDLDSSQVTEAAQDPGGNSEKSLCTFDLNEEVVSDDMDVSVNAMSTVPIPVVSASKPAQSSGLPMAPLQFEGTLGWKGSAATSAFRRASPRRNSDGEKNVSIGRNSEISKQRHCCLDFDLNVAEGEEGLVKQIGESSGLPSGQSSVELSPKRTNRFELDLNSIGDDGDAQPSDQRMEGPLFSGRNGYWSPSPASSSSSMQPSVRNIDLNDRPYFQTDLVESSYKRSKLDASVISILGAKVEVGRREYVPQTLSLPNGKAIEPAMDHPLSGAGGILGMGPPLSYNHSNAFGYNGLTSVPALSFSSAMYGPSGGPIPYMVDSRGTPVMPQVGGSSSTVLSSYTQPPFIVSMTRTQFGLNGVGSSHPNFDLNSGFTIDGPNRDMLTARQFFFPAQGRAIEEHVRTLPQSSSSGVSVKRKEPDGAWETYPRSYKHQQPPWK

>PhvulMED31

MACRSVVSSSLHMTNFMAVKGVLSFAPNPPLFHHLPKVVTFVSLSASVKLPTKSFQVRSVVGTTEAASEFDEMVSGTKRKYYMLGGKGGVGKTSCAASLAVKFANNGHPTLVVSTDPAHSLSDSFAQDLTGGALVPVEGPDFPLFALEINPEKSREEFRNAAQKNGGTGVKDFMDGMGLGMIADQLGELKLGELLDSPPPGLDEAIAISKVMQFLESQEYSMFTRIVFDTAPTGHTLRLLSLPDFLDASIGKILKLRQKIASATSAIKSVFGQENTQQNSADKLEKLRERMLKVRELFRDTDSTEFVIVTIPTVMAVSESSRLSASLKKENVPVKRLVVNQILPPSPSDCKFCAMKRKDQMRALDLIQNDPELSSLLMIQAPLVDVEIRGVPALKFLGDIIWK

>PhvulMED36-1

MAPPRGRGGGSYSGGGSFSGGGGSGGFRGGRGDRGRGRGGGGRGGDRGTPFKARGGGRGGRGGGRGGGRGGGRGGGMKGGSRVVVQPHRHEGIFIAKGKEDALVTKNLVPGEAVYNEKRITVQNEDSSKVEYRIWNPFRSKLAAAILGGVDNIWIKPGARVLYLGAASGTTVSHVSDIVGPTGVVYAVEFSHRSGRDLVNMAKKRTNVIPIIEDARHPAKYRMLVGMVDVIFSDVAQPDQARILGLNASYYLKTGGHFVISIKANCIDSTVPAEAVFESEVNKLKADQFKPFEQVTLEPFERDHACVVGGYRMPKKKKDTA

>PhvulMED36-2

MKDVFKIGKKRKKNERSPAEIALLVANVMAGLEVTAQEDADLNRQGKPAINKLKQLPLLTQVLSKKQLQLEFLDHGILTLLKNWLEPLPDGSLPNINIRMEILRILNEFPIDLEQCDRREQLKKSGLGRVIMFLSKSDEETRLNRKLAKELVDKWSRPIFNNSILFEDIRKKKKCRR

>PhvulMED36-3

MASGNENGVNERDMQKLYWVQHSADLSVEAMMLDSKAAHLDKEERPEVLSLLPPCEGKSVIELGAGIGRFTGELAQKAGQLLAVDFIESAIKKNESINGHHKNVKFLCADVTSPNMSSSVSEGSVDVIFSNWLLMYLSDNEVEKLAERMIKWLKDGGYIFFRESCFHQSGDSKRKHNPTHYREPRFYTKVFKECHMSDNTGNSFELSLIGCKCIGAYVRNKKNQNQICWIWQKVKSVDDRGFQRFLDRVEYSHKSILRYEQMYGPGFVSTGGLETTEEFVAKLGLKPGQKVLDVGCGVGGGDFYMAENFDVEVVGIDLSINMISLAIERAIGLNYAVEFDCADCFKKSYPDNTFDVIYTRDTMLHVKDKPTLFRSFYKGLKPGGKILITDYCKSAQSPSSEFAEYIKKGGYYLHDMKAYEKMLEDAGFDDLIAEDRTDQFVKTLQKELDALENKKDDFIRDFSEITMKLWKDGRQSRAGVHLKSRCGACSLPRKIDMLCCCHLSCSIIFSLCVTLMF

>PhvulMED36-4

MNKGGGAGLGGGAGAGSGPTAAAASAAAQKQKTLLQRVEGDIANIVDNFSHLVNVARVNDPPVRNSQEAFMMEMHSARMVQAADSLLKLVSELKQTAIFSGFASLNDHVEQRRIEFNQLAEKTDHALSRVGEEAAANLKELESHYTSSAQKTMQNLQP
